# Spiroketal Synthesis Via Uncatalyzed Inverse Electron-Demand Hetero-Diels-Alder Reactions at Ambient Temperature

**DOI:** 10.1021/jacs.6c09678

**Published:** 2026-09-17

**Authors:** Emma J. Greene, Matthew C. Raeside, Marcelo A. Mazariego, Andy Y. Song, K. N. Houk, Patrick G. Harran

**Affiliations:** Department of Chemistry and Biochemistry, 8783 University of California, Los Angeles, 607 Charles E. Young Dr., Los Angeles, California 90095, United States

## Abstract

Vinyl vicinal tricarbonyl reagents have been used as flexible bis-electrophiles in a variety of condensation reactions. Here, we show the enone component of the molecule participates in uncatalyzed inverse electron-demand hetero-Diels-Alder reactions with α-methylidene furans, pyrans and oxepanes. This previously unrecognized reactivity mode enables direct, diastereoselective access to 5,6-, 6,6- and 7,6- spiroketal substructures using stoichiometric reactants at ambient temperature – and without the use of Lewis acidic additives or catalysts. Broad substrate scope is demonstrated using diverse cyclic enol ethers, with substrate-dependent competition between Diels-Alder and carbonyl-ene pathways occurring for specific ring systems. Density functional theory calculations reveal that both pathways proceed through polar, stepwise mechanisms involving zwitterionic intermediates, with subtle energetic differences governing pathway selectivity. The unsaturated α-keto ester present in cycloaddition products can be manipulated in a variety of useful ways. A selection of enabling transformations is demonstrated, as is a concise synthesis of a reveromycin B subunit.

## Graphical abstract

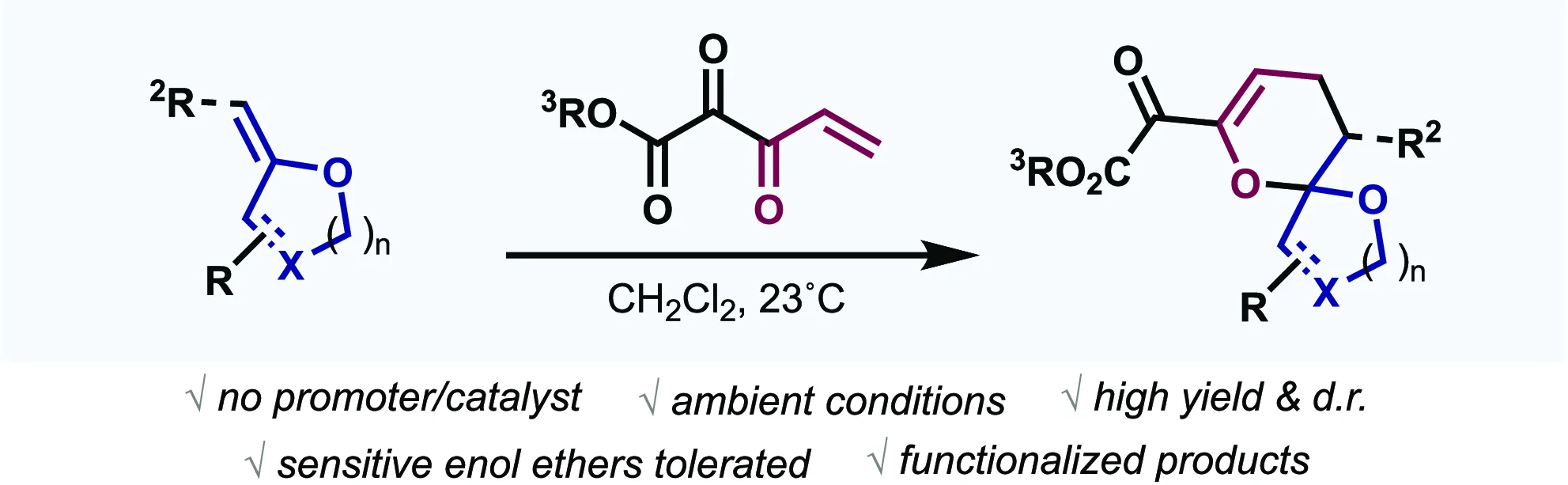

## Introduction

The spiroketal motif is prominent in natural products, especially in those of polyketide origin. Desiccation of linear keto diol precursors forms conformationally constrained scaffolding for a range of bioactive pharmacophores.^1^ Methods to form spiroketals synthetically (Figure 1A) include a range of protic and Lewis acid-catalyzed protocols for internal ketalization and transketalization.^2–5^ Transition metal catalyzed tandem hydroalkoxylation of alkynyl diols also generates spiroketals, as does oxygenation of α-ethereal methine hydrogens.^2,3,6^ Yield and diastereoselectivity tend to be moderate in the latter. Relevant to the current study are [4+2] cycloaddition reactions between alkylidene cycloethers and unsaturated carbonyl compounds (Figure 1A). Such inverse electron-demand hetero-Diels-Alder (IED-HDA) reactions can occur with high regio- and stereocontrol. When using enone derivatives as the diene component, Lewis acid catalysts, transition metal catalysts or high temperatures are required to promote spiroketal formation.^7–13^ Because exocyclic alkylidene ethers are labile, olefin isomerization can compete with cycloaddition under these conditions.^7,14^ This is especially true for methylidene tetrahydrofurans used to construct dioxaspiro[4.5]­decanes (5,6-spiroketals). In that case, participating substrates often require substituents that block isomerization (Figure 1B) for efficient reactions.

**Figure 1:**
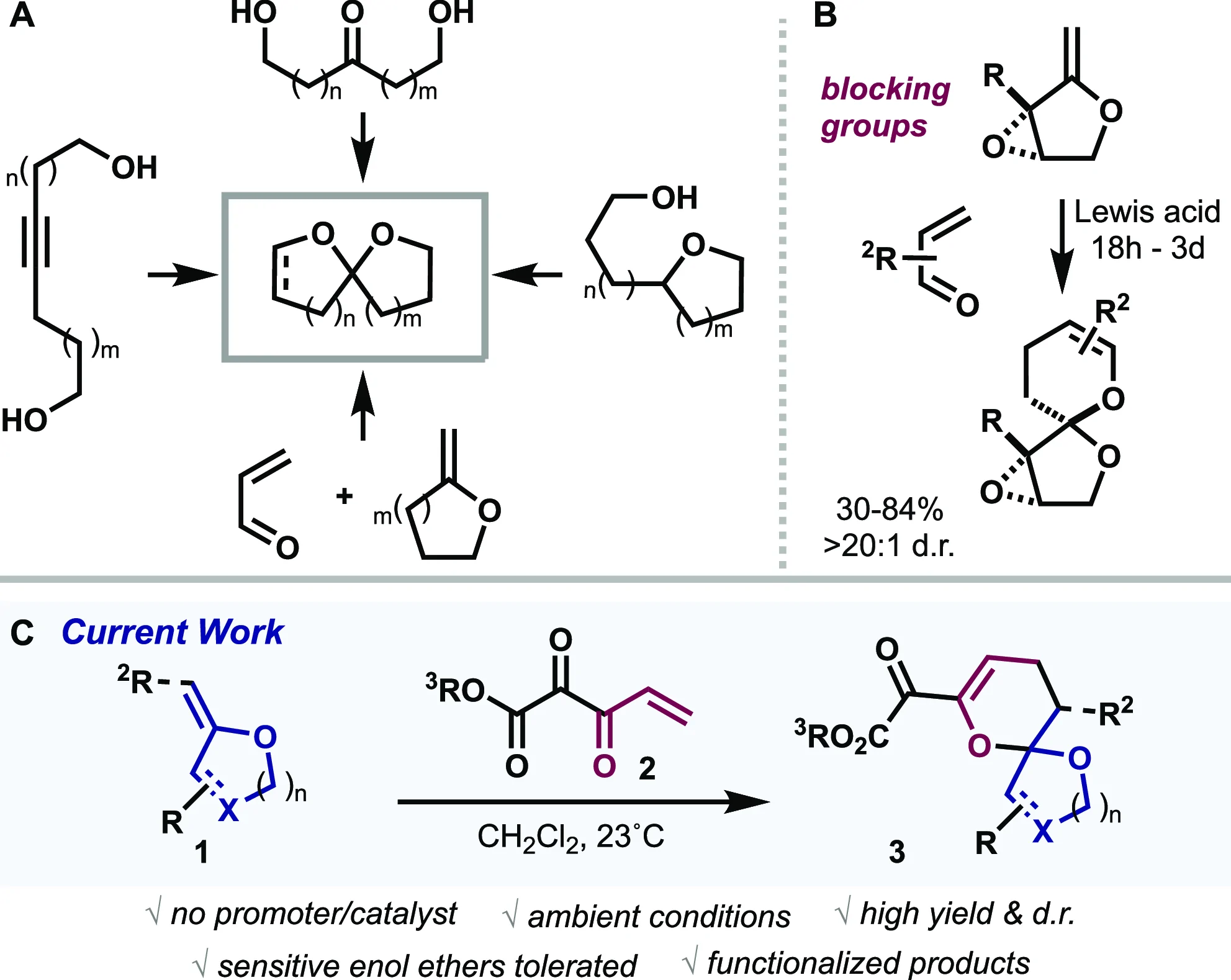
(A) General methods for spiroketal synthesis. (B) Lewis acid catalyzed IED-HDA reactions using methylidene tetrahydrofurans typically require substituents to block competing alkene isomerization (see ref 14). (C) Vinyl vicinal tricarbonyl reagents **2** spontaneously cycloadd to methylidene furans, pyrans and oxepanes to afford spiroketals at room temperature.

During studies aimed at synthesizing the natural product portimine A, we had occasion to prepare heterodendralene **4** (Scheme 1). The carbonyl group in this novel oxoacetyl pyrroline is overtly electrophilic and hydrates readily. In desiccated (keto) form, the molecule reacts with methylidene tetrahydrofurans via a carbonyl-ene reaction to afford dihydrofuran adducts in good yield. The reaction occurs spontaneously when equimolar amounts of reactants are mixed in CH_2_Cl_2_ at room temperature. Notably, before we had developed a viable synthetic route to **4**, we attempted to use Wasserman’s vinyl vicinal tricarbonyl reagent **2a** ^15,16^ as a surrogate for **4** to test the feasibility of the ene reaction. However, when the hydrate of **2a** was desiccated and treated with 1.1 equiv of enol ether **1c**, no ene product was detected. Instead, a single diastereomer of spiro cycloadduct **3c** was isolated in good yield. Wasserman had used **2a** in a variety of creative annulations in which the enone segment of the molecule functioned as a Michael acceptor and subsequent ring closures occurred at the central carbonyl group. With an alkylidene ether as the nucleophile, it appeared the enolate ion pair formed from Michael addition collapsed directly to **3c**, leaving the central carbonyl intact. In contrast, the s-*trans* vinyl imine in **4** appeared not to engage in a Michael addition (or it was reversible). Instead, the carbonyl ene pathway predominated. The latter was advantageous for portimine studies. The former represented a new, selective method for generating complex spiroketal fragments under exceptionally mild conditions, ones that tolerate even the most sensitive of enol ether reactants. Here we explore the scope of the cycloaddition, computationally probe its mechanism and demonstrate the utility of its reaction products.

**Scheme 1:**
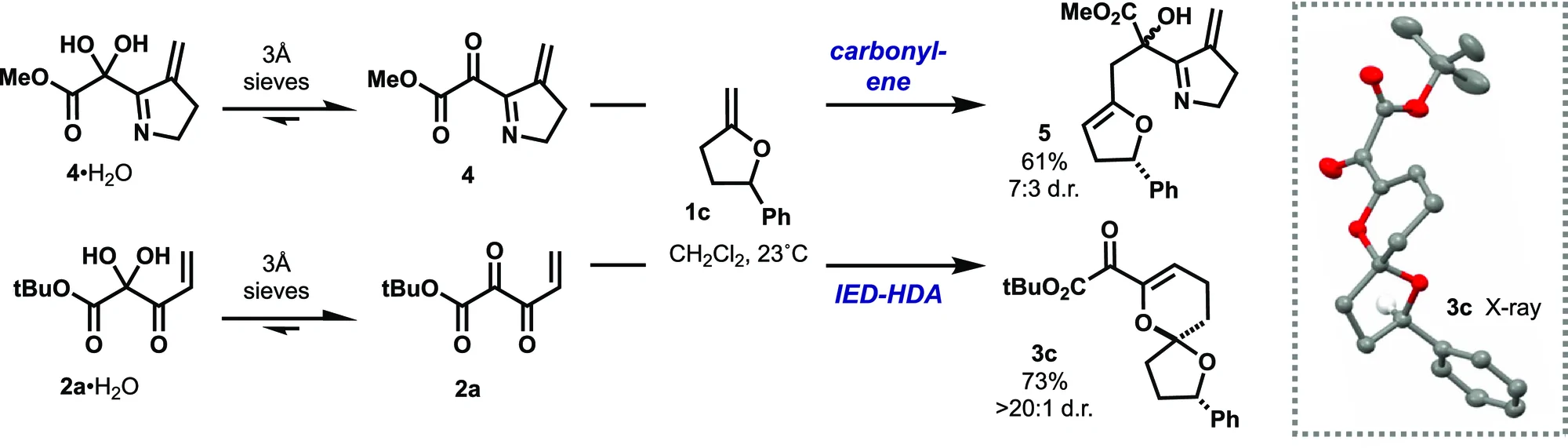
Divergent Reactivity of Heterodendralenes **4** and **2a**

## Results and Discussion

Methylidene tetrahydrofurans, pyrans and oxepanes used in this study were prepared either by base-mediated elimination of HX from halohydrin ethers,^17^ or by dimethyltitanocene mediated methylenation of lactones according to the Petasis protocol and modifications thereof (see Supporting Information).^18–20^ Known compounds **1a** and **1p** were prepared according to established protocols.^21,22^ Enol ethers of this type are prone to isomerization and/or hydrolysis. They were handled as distilled liquids or as crude supernatants following oxo titanium byproduct precipitation. As shown in Figure 2, sensitive mono or poly-substituted methylidene tetrahydrofurans (**1a-m)** reacted with **2a** in CH_2_Cl_2_ at room temperature to afford spiroketal products in moderate to excellent yields (50–89%). Even methylidene dihydroisobenzofuran reacts efficiently to form spirocycle **3j**. Alkylidene phthalans are sufficiently labile that catalytic acetic acid can generate isobenzofuran prototropisomers in situ and these react with enones as the diene component in Diels-Alder reactions.^23^ In contrast, under neutral conditions with no catalyst, **1j** reacts with **2a** selectively as a dienophile. Products derived from isomerization and/or hydrolysis of the enol ether were not detected. The same was true when reacting uridine-derived enol ether **1k**,^24^ gluconolactone-derived enol ether **1l**,^25^ glycolate-derived methylidene dioxolane **1n** and methylidene oxepane **1o**. Free hydroxyl groups, including vicinal diols, and polar heterocycles are tolerated in the reactions. Interestingly, methylidene pyrans behaved differently. Reaction of 5-butyl-2-methylidene tetrahydropyran **1q** with **2a** gave cycloadduct **3q** in 31% yield, but the major product was ene adduct **5q**. The same result was seen with 2-methylidene tetrahydropyran (**1p**), wherein ene product **5p** was formed as the major product in a 1.6:1 mixture with cycloadduct **3p**. For the 5, 6 and 7-membered ring nucleophile series **1a**–**q**, only the pyran showed this behavior – and the ratio of products in that case was solvent dependent. The ene product predominated in CH_2_Cl_2_, the cycloadduct was favored in THF and there was no selectivity in toluene.

**Figure 2:**
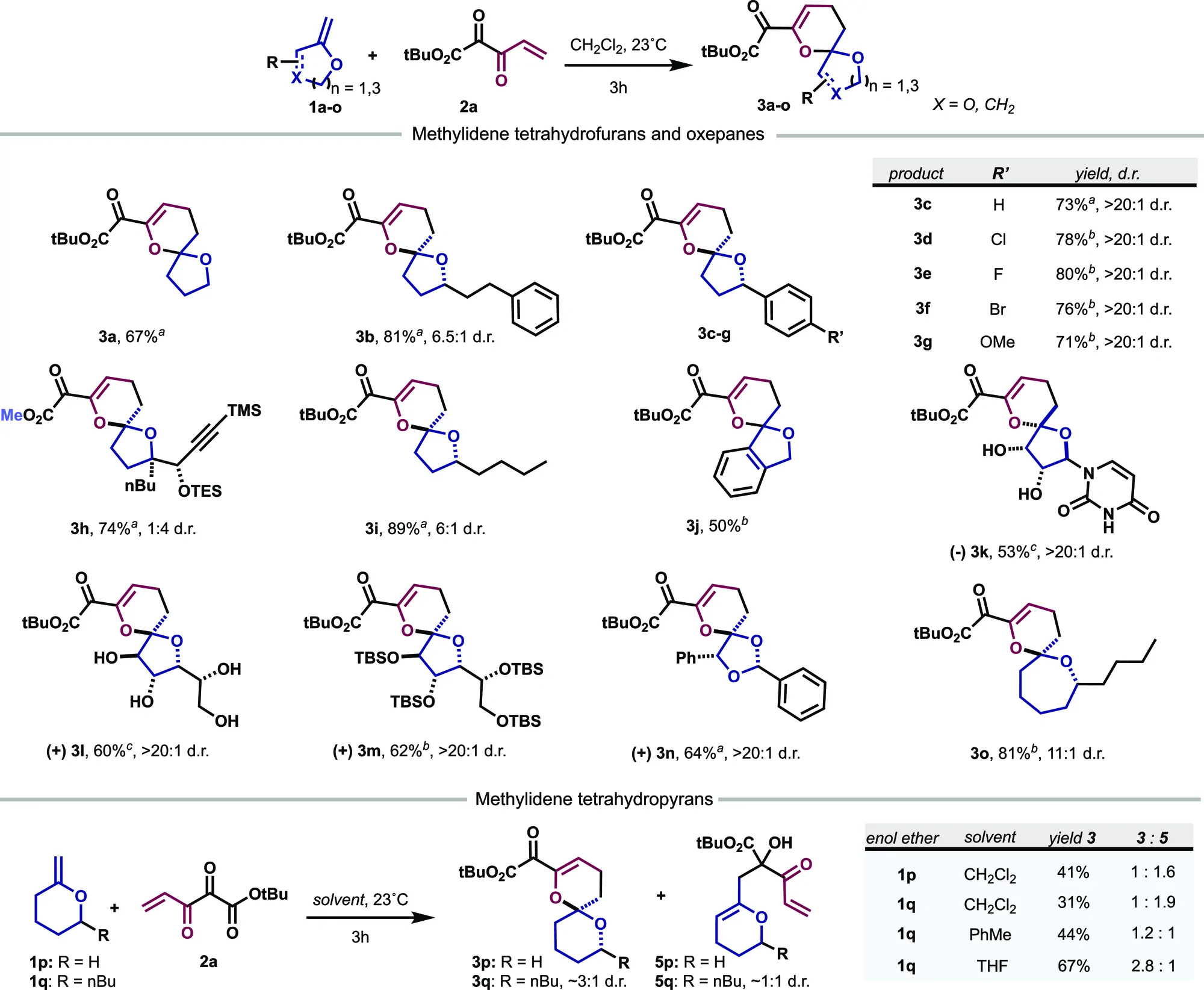
Initial substrate survey. All yields are isolated. ^*a*^ Reaction conditions: enol ether **1** (1.0-1.2 equiv), enone **2** (1.0 equiv), CH_2_Cl_2_ (0.3 M), 23 °C. ^*b*^ Reaction conditions: enol ether **1** (1.0 equiv), enone **2** (1.0-1.7 equiv), CH_2_Cl_2_ (0.3 M), 23 °C. ^*c*^ Reaction conditions: enol ether **1** (1.0 equiv), enone **2** (1.1 equiv), THF (0.1 M), 24 h, 23 °C.

### Computational Studies

To understand the likely subtle effects underpinning these observations, Density functional theory (DFT) calculations were used to probe the mechanism of competing hetero-Diels-Alder (HDA) and carbonyl-ene pathways for enol ethers **1i, 1o and 1q** under CH_2_Cl_2_ solvation. Figure 3 shows energy surfaces calculated at the M06-2*X*/6-311+g­(d,p) /SMD (CH_2_Cl_2_) level of theory for reactions with **2a**. Both the IED-HDA pathways and the carbonyl-ene pathways involve zwitterionic transition structures. The IED-HDA reactions are all highly asynchronous, concerted pathways with activation energies of ∼18 kcal mol^–1^. The forming CC bond is about 2.1Å, while the forming CO bond is 3.2-3.3Å. The zwitterionic nature of the TS is reflected by the values of δq in Figure 3, which are the amount of charge donated from the enol ether to the tricarbonyl compound in the transition states.

**Figure 3:**
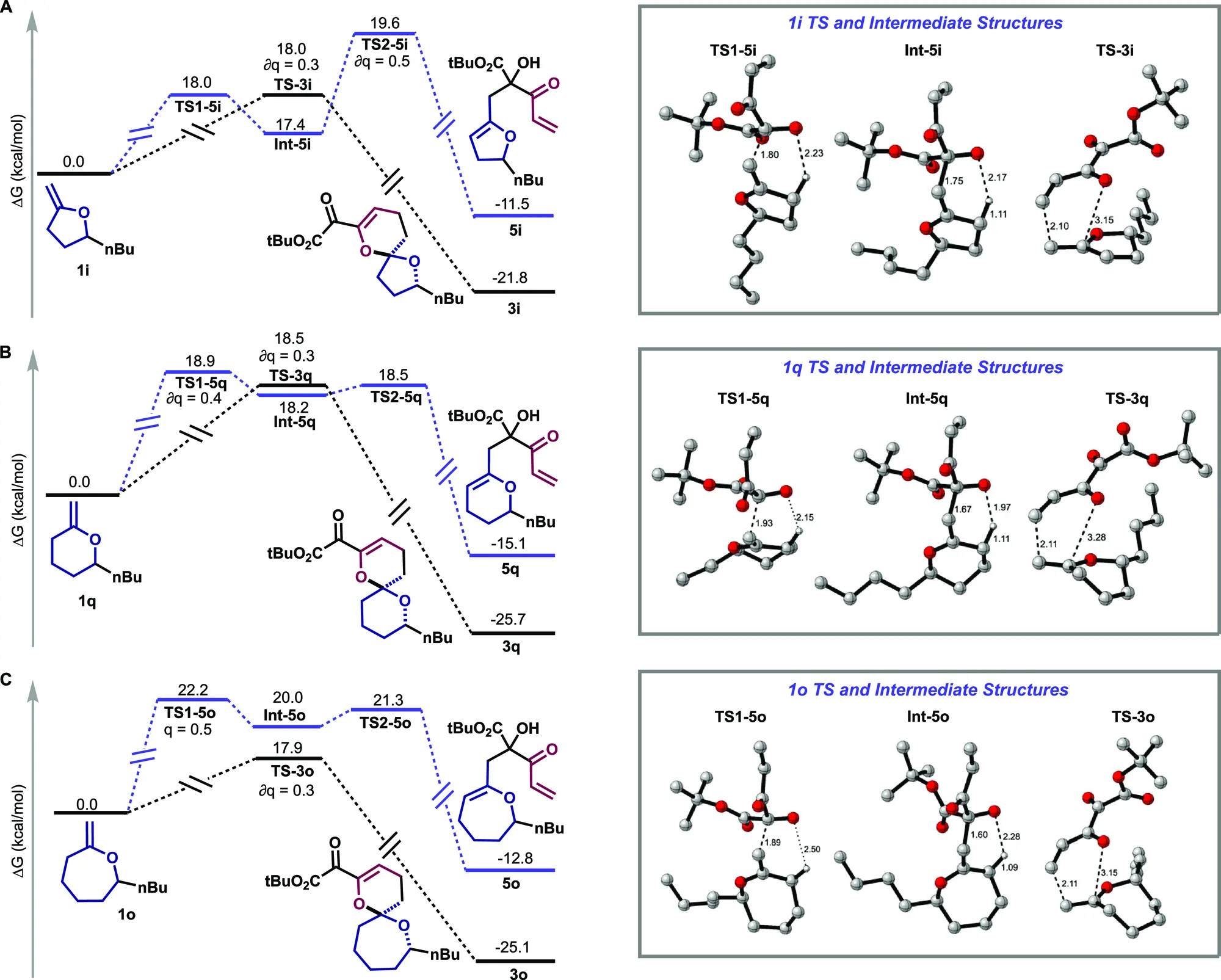
Reaction pathways and carbonyl-ene transition state structures. (A) Pathways, TS 1s and intermediates for **1i**. (B) Pathways, TS 1s and intermediates for **1q**. (C) Pathways, TS 1s and intermediates for **1o**.

The carbonyl-ene reaction pathways involve zwitterionic intermediates **Int-5i**, **o**, and **q**, which undergo subsequent proton transfer to afford the homoallylic alcohol products. The potential energy surfaces around the transition states and zwitterionic intermediate are quite flat, with minimal energy differences between various TSs and intermediates. The formations of the zwitterionic intermediates, with partial CC bond formation of 1.8-1.9Å, occur with barriers of 18-22 kcal mol^–1^, while the second barriers occur at energies of 18-21 kcal mol^–1^. The energies of the transition states, **TS2-5i**, **o**, and **q**, are inversely related to the distances from the O^–^ to the proton that is transferred in **Int-5i**, **o**, and **q**. That is, the shortest distance, 1.97Å in **Int-5q** corresponds to the lowest energy, 18.5 kcal mol^–1^, for **TS2-5q**. The longest distance, 2.28Å, for **Int-5o**, corresponds to the highest energy, 21.3 kcal mol^–1^, for **TS2-5o**. **TS2i** is intermediate in energy.

The differences in rate-determining steps for formation of IED-HDA and carbonyl-ene products are 1.6-4.3 kcal mol^–1^ for reactions of five- and seven-membered enol ethers, **1i** and **1o**, consistent with the observation of only IED-HDA products for these reactions. For the six-membered enol ether, **1q**, this difference drops to 0.4 kcal mol^–1^, consistent with the experimental observation of formation of both IED-HDA and carbonyl-ene products. The rate-determining TS for carbonyl-ene product formation is more polar than that for the IED-HDA product, consistent with the greater amount of carbonyl ene product in CH_2_Cl_2_ than in THF.

### Additional Scope

To probe the effect of proximal substitution on stereochemical and path selectivity, and to anticipate scope in more complex settings, reacting partners **1i/q** and **2** were incrementally methylated. The syn C-3 methyl congener of **1i (1r**, Table 1) underwent cycloaddition to **3r** efficiently and with increased diastereoselectivity (>20:1 d.r.). Enhanced stereoselection was also observed with pyranyl substrate **1s**. Notably, in this case methylation caused a reversal in path selectivity. Cycloadduct **3s** formed in preference to the carbonyl-ene product **5s** (87:13). One carbon homologation of the enol ether was also tolerated. Ethylidene tetrahydrofuran **1t** cycloadded to **2a** to afford a single diastereoisomer of spirocycle **3t** in good yield.

**Table 1:**
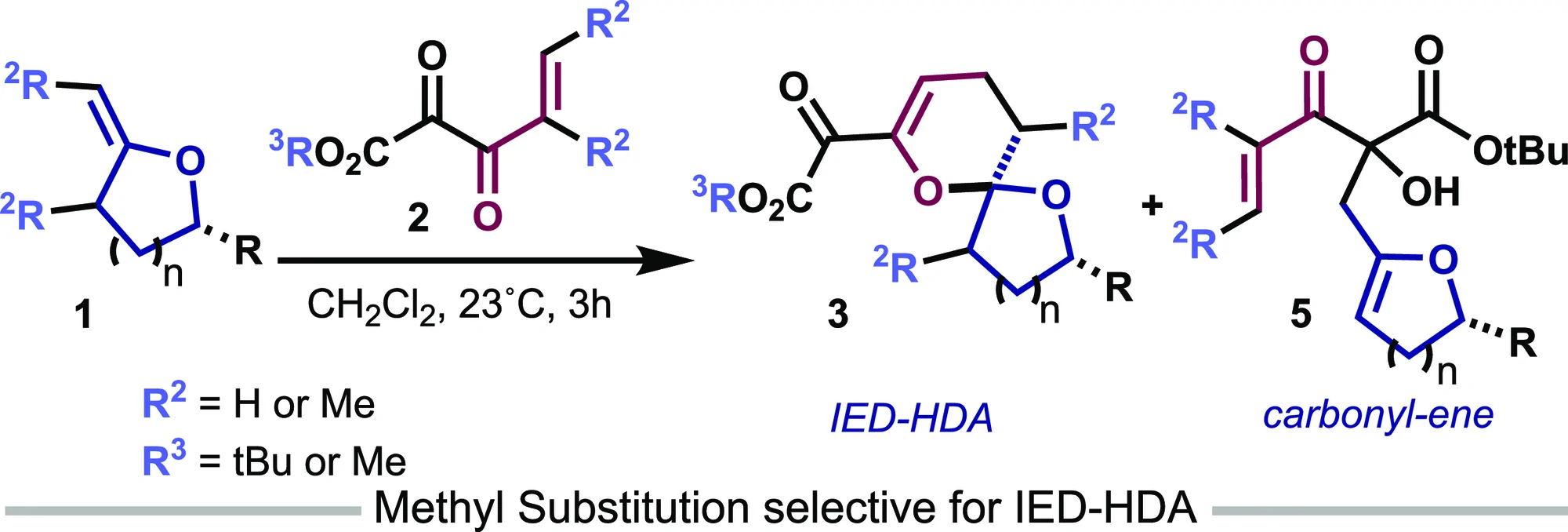

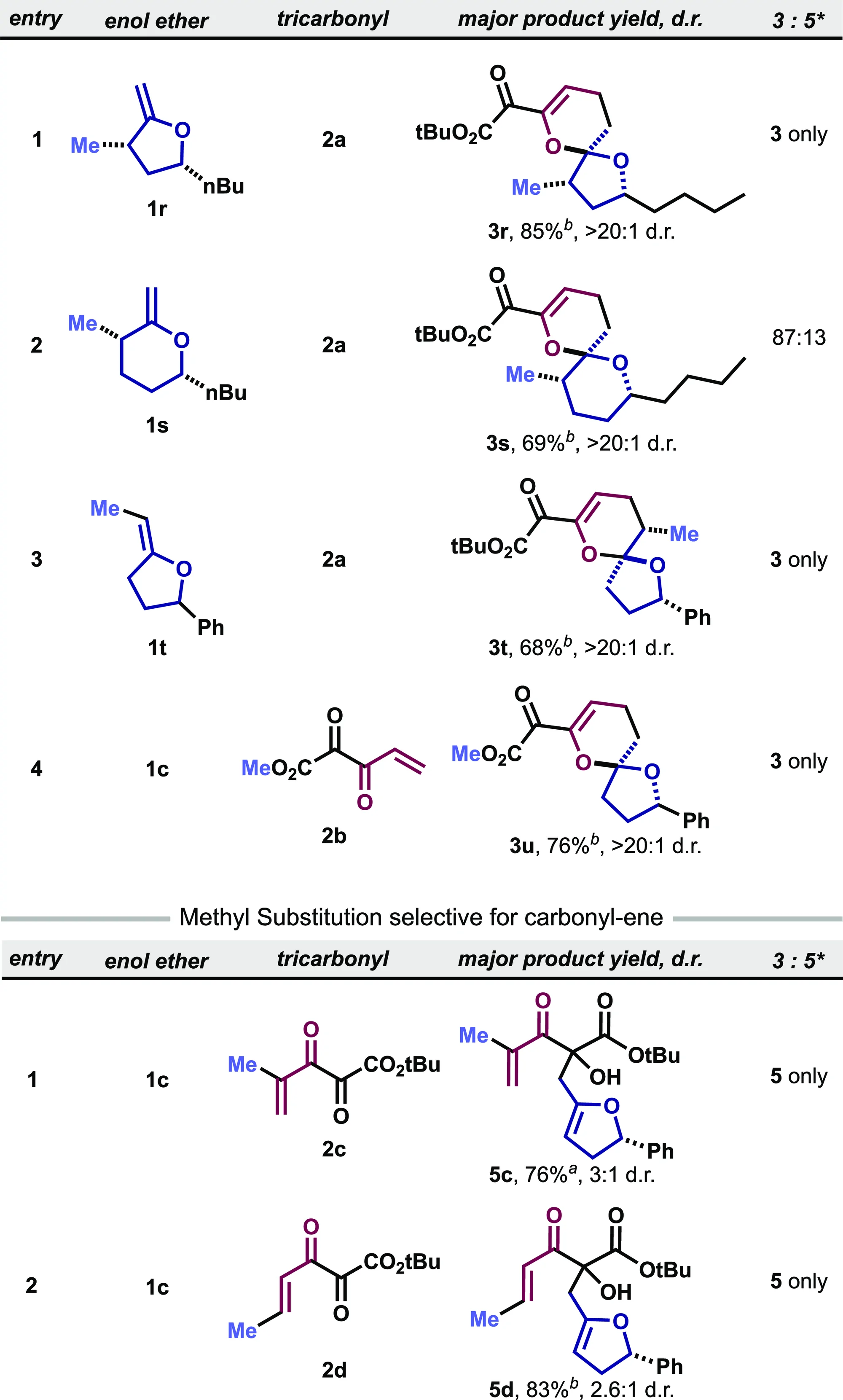
Reactant Methyl Scan

All yields are isolated.

a Reaction conditions: enol ether **1** (1.0-1.2 equiv), enone **2** (1.0 equiv.**)**, CH_2_Cl_2_ (0.3 M), 23 °C.

b Reaction conditions: enol ether **1** (1.0 equiv), enone **2** (1.0-1.7 equiv), CH_2_Cl_2_ (0.3 M), 23 °C. *As detected by 1H NMR.

Turning to the tricarbonyl component, changing the ester substituent from *tert*-butyl to methyl (i.e., **2b**) had no measurable impact on IED-HDA reactivity/selectivity, despite reduced steric congestion near the central carbonyl group. On the other hand, replacing either vinyl hydrogen in **2a** with a methyl group completely suppressed cycloaddition reactivity. Mixing homologs **2c** or **2d** with enol ether **1c** gave ene products **5c** and **5d**, respectively, in high yield. Cycloadducts were undetectable in the crude reaction mixtures. Taken together, data indicates **2a** and its ester congeners will participate in diastereoselective cycloadditions with a range of alkylidene cycloethers, while even modest perturbations of the vinyl group will shift reactivity fully towards carbonyl ene adducts.

### Manipulations and Utility of Cycloaddition Products

The α-keto ester segment of **2** that enhances heterodiene reactivity of its enone component also facilitates functionalization of cycloaddition products. Numerous manipulations are possible and these can be exploited for synthesis. The ketone remains reactive and readily accepts nucleophiles. For example, the oxygenated enone in **3c** participates in selective 1,2-additions with alkyl nucleophiles and hydride (see **12** and **13**, Scheme 2), even under conditions that would normally favor conjugate addition. The keto ester in **3u** can be cleanly reduced to diol **7** using LiAlH_4_, and the diol is efficiently cleaved to aldehyde **8** by NaIO_4_. One can envision myriad ways to elaborate enal **8** further. If NaBH_4_ is used in place of LiAlH_4_ for the reduction of **3u**, and the solvent is changed to MeOH, mildly acidic workup provides spirocyclic oxepanone **6**, presumably via ring expanding rearrangement of a boron-complexed form of **7**. This provides ready access to a new series of 7,5-spiroketal ring systems. The ester in **3u** can also be saponified and the incipient keto acid is stable. α-keto acids are linchpins that participate in a variety of decarboxylative acylation reactions.^26–29^ The example shown in Scheme 2 engages the crude sodium carboxylate derived from **3u** in a mild KAHA ligation^27,28^ with *N*-methyl hydroxylamine hydrochloride to afford enamide **11** in high yield. The keto ester functionality in cycloaddition products allows for direct conjugations that leave the unsaturated spiroketal intact.

**Scheme 2:**
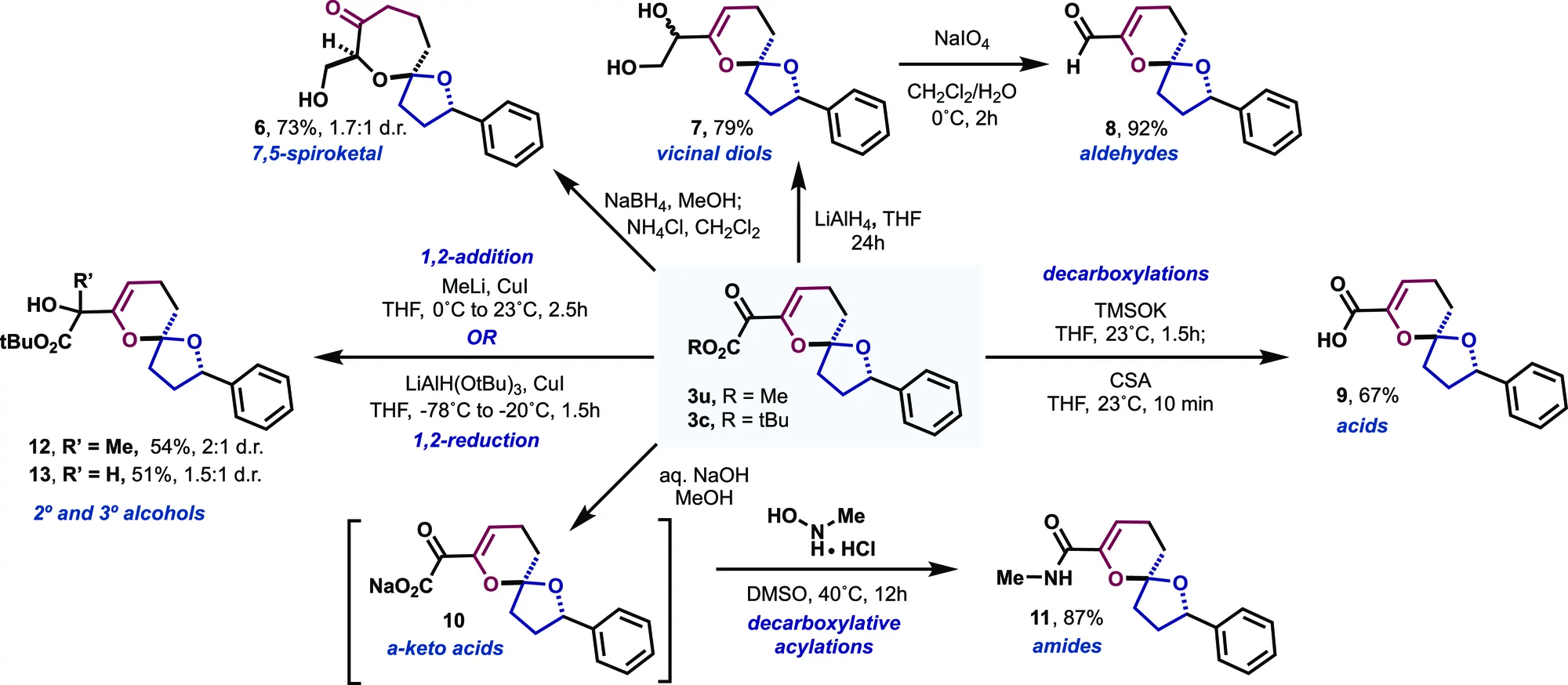
Manipulation of Cycloaddition Products^a^

a All yields are isolated. See Supporting Information for full experimental details.

Lastly, during attempts to generate the keto acid derivative of **3u** under anhydrous conditions, a novel reaction was discovered. Treatment of **3u** with 1.2 equiv of KOTMS in THF at room temperature followed by neutralization with CSA affords carboxylic acid **9** directly. One carbon degradation of α-keto acids to carboxylic acids is a well-established transformation under oxidative conditions. But, to our knowledge, the direct degradative conversion of α-keto esters to carboxylic acids is without precedent and the KOTMS/THF procedure is non-oxidative. The same reaction run on methyl pyruvate affords, after workup, α-methyl fumaric acid. In contrast, treatment of tetramethyl pyruvate with KOTMS in THF affords only the corresponding α-keto acid (see Supporting Information). Further studies are underway to understand the unexpected reactivity of unsaturated α-keto esters with KOTMS.

To demonstrate the utility of the uncatalyzed IED-HDA for complex molecule synthesis, we chose a system where comparison of related strategies was possible. During their synthesis of reveromycin B, Rizzacasa et al. had designed a thermal alkylidene pyran/enal based IED-HDA to generate spiro-bispyran **14**. Subsequent reactions led to that substrate being functionalized and ring contracted *en route* to target 5,6-spirocycle **15**.^10^ The mildness of uncatalyzed IED-HDA reactions using **2a/b** and their compatibility with sensitive methylidene tetrahydrofurans allowed more direct access to structures of type **15** (Scheme 3). ZnBr_2_ promotes the Mukaiyama aldol^30,31^ addition of butylated siloxyfuran **16** to 3-trimethylsilylpropynal (**17**) to afford butenolide **18**. Silylation of the secondary alcohol, conjugate reduction of the enoate and Petasis methylenation of the lactone gave enol ether **1h**. Mixing this material with an equivalent of **2b** in CH_2_Cl_2_ at room temperature gives cycloadduct **3h** efficiently, albeit with lowered diastereoselectivity. 5,5′ geminal disubstitution in **1h** renders the diastereotopic faces of the alkene less sterically differentiated than other substrates in this study. Nonetheless, the target spirocycle was accessed in six steps from commercial materials. There is potential for equilibrating the stereochemistry at the spiro center in **3h** under acidic conditions. Moreover, the alkene and α-keto ester groups provide numerous options for pyran functionalization and side chain elaboration.

**Scheme 3:**
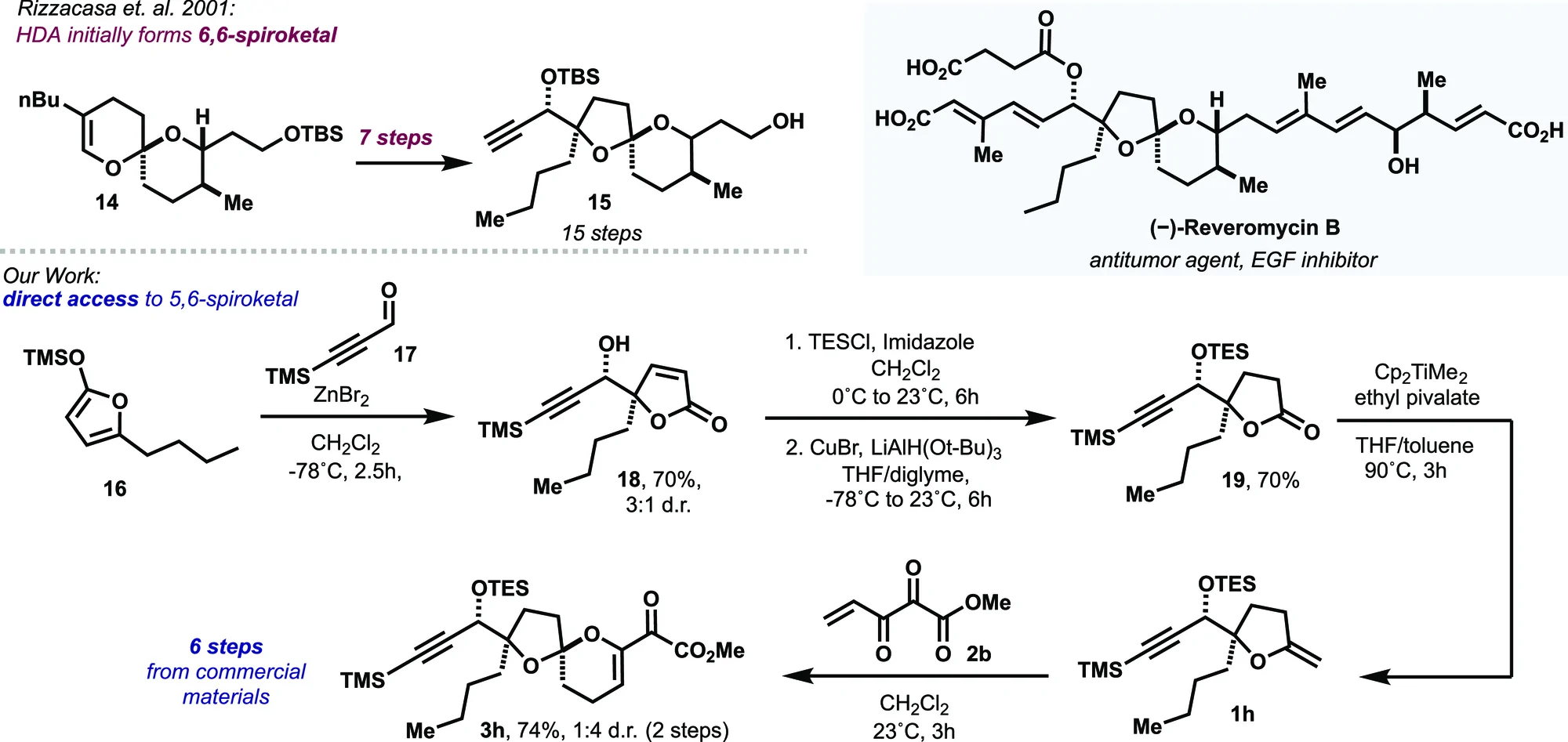
Assembly of a Reveromycin B Subunit^a^

a All yields are isolated. See Supporting Information for full experimental details.

## Conclusion

We have discovered an uncatalyzed inverse electron-demand hetero-Diels-Alder reaction that enables diastereoselective synthesis of spiroketals under exceptionally mild conditions. The reaction reveals a previously unrecognized reactivity of heterodendralenes **2** and is compatible with even the most sensitive of enol ether substrates. The method tolerates variations in enol ether substitution and ring size, providing direct access to 5,6, 6,6 and 7,6-spiroketal frameworks.

DFT calculations indicate the mechanisms of the IED-HDA cycloadditions are highly asynchronous, concerted processes with rather zwitterionic transition states and activation free energies around 18 kcal mol^–1^. The competing carbonyl-ene pathways proceed through stepwise mechanisms involving zwitterionic intermediates that have very low barriers to proton transfer. There are small energetic differences between these pathways, modulated by substrate structures and polarities of transition states. These lead to the carbonyl-ene reaction competing with the IED-HDA in only the case of six-membered enol ethers, where the intermediate is formed with close proximity of alkoxide and the proton that is subsequently transferred.

The spiroketal cycloaddition products in this study carry functionality that can be manipulated in a variety of useful ways, providing access to building blocks of potential value in complex molecule synthesis.

## Supplementary Material



## Notes

Detailed experimental procedures; spectroscopic data; X-ray crystallographic data; and computational details (PDF)

## References

[1] Zhang F.-M., Zhang S.-Y., Tu Y.-Q. (2018). Recent
Progress in the Isolation,
Bioactivity, Biosynthesis, and Total Synthesis of Natural Spiroketals. Nat. Prod. Rep..

[2] Perron F., Albizati K. F. (1989). Chemistry of Spiroketals. Chem.
Rev..

[3] Boivin T. L. B. (1987). Synthetic
Routes to Tetrahydrofuran, Tetrahydropyran, and Spiroketal Units of
Polyether Antibiotics and a Survey of Spiroketals of Other Natural
Products. Tetrahedron.

[4] Miyakoshi N., Aburano D., Mukai C. (2005). Total Syntheses
of Naturally Occurring
Diacetylenic Spiroacetal Enol Ethers. J. Org.
Chem..

[5] Dias L. C., de Oliveira L. G. (2004). Short Synthesis of the 6,6-Spiroketal Cores of Spirofungins
A and B. Org. Lett..

[6] Crimmins M. T., O’Mahony R. (1990). Synthesis
of Spiroketals: A General Approach. J. Org.
Chem..

[7] Marsini M. A., Huang Y., Lindsey C. C., Wu K.-L., Pettus T. R. R. (2008). Diastereoselective
Syntheses of Chroman Spiroketals via [4 + 2] Cycloaddition of Enol
Ethers and o-Quinone Methides. Org. Lett..

[8] Zhou G., Zhu J., Xie Z., Li Y. (2008). An Efficient Synthesis of Highly
Functionalized [5,6] Aromatic Spiroketals by Hetero-Diels–Alder
Reaction. Org. Lett..

[9] Lindsey C. C., Wu K. L., Pettus T. R. R. (2006). Synthesis
of Electron Deficient 5,6-Aryloxy
Spiroketals. Org. Lett..

[10] Cuzzupe A. N., Hutton C. A., Lilly M. J., Mann R. K., McRae K. J., Zammit S. C., Rizzacasa M. A. (2001). Total Synthesis
of the Epidermal
Growth Factor Inhibitor (−)-Reveromycin B. J. Org. Chem..

[11] Zhou L., Yu S.-Y., Tang Y., Wang L. (2019). Facile Stereoselective
Approach to Diverse Spiroheterocyclic Tetrahydropyrans: Concise Synthesis
of (+)-Broussonetine G and H. Angew. Chem.,
Int. Ed..

[12] Boger D. L., Robarge K. D. (1988). Divergent de Novo Synthesis of Carbohydrates Based
on an Accelerated Inverse Electron Demand Diels-Alder Reaction of
1-Oxa-1,3-Butadienes. J. Org. Chem..

[13] Wasserman H. H., Blum C. A. (1994). The Chemistry of Vicinal Tricarbonyls. Use of Vinyl
and Acetylenic Derivatives in the Synthesis of Substituted Indoles. Tetrahedron Lett..

[14] Pale P., Chuche J. (1988). A Two Step Synthesis
of Spiroketals. Tetrahedron Lett..

[15] Wasserman H. H., Parr J. (2004). The Chemistry of Vicinal Tricarbonyls and Related Systems. Acc. Chem. Res..

[16] Wasserman H. H., Cook J. D., Vu C. B. (1990). The Chemistry
of Vicinal Tricarbonyls.
New Routes to Indolizidines. J. Org. Chem..

[17] Lee A. S.-Y., Tsao K.-W., Chang Y.-T., Chu S.-F. (2007). L-Proline-Catalyzed
Intramolecular Cyclization of 5-Hydroxypentene to β-Halogenated
Tetrahydrofuran. Tetrahedron Lett..

[18] Petasis N. A., Bzowej E. I. (1990). Titanium-Mediated Carbonyl Olefinations. 1. Methylenations
of Carbonyl Compounds with Dimethyltitanocene. J. Am. Chem. Soc..

[19] Pine S. H. (1993). Carbonyl
Methylenation and Alkylidenation Using Titanium-Based Reagents. Org. React..

[20] Payack J. F., Huffman M. A., Cai D., Hughes D. L., Collins P. C., Johnson B. K., Cottrell I. F., Tuma L. D. (2004). Dimethyltitanocene:
From Millimole to Kilomole. Org. Process Res.
Dev..

[21] Guerra
Faura G., Nguyen T., France S. (2021). Catalyst-Controlled
Chemodivergent Reactions of 2-Pyrrolyl-α-Diazo-β-Ketoesters
and Enol Ethers: Synthesis of 1,2-Dihydrofuran Acetals and Highly
Substituted Indoles. J. Org. Chem..

[22] Aumann K. M., Healy P. C., Coster M. J. (2011). Arylation of [6,6]-Spiroacetal Enol
Ethers: Reactivity and Rearrangement. Tetrahedron
Lett..

[23] Meegalla S. K., Russell R. (1989). Alkyl Isobenzofurans from Phthalides;
Inter and Intramolecular
Diels-Alder Reactions. Synthesis.

[24] Xiang L., Hu T., Xue H., Pan W., Xie Y., Shen J. (2023). Synthesis
and Evaluation of NHC Derivatives and 4′-Fluorouridine Prodrugs. Org. Biomol. Chem..

[25] Murphy P. V., McDonnell C., Hämig L., Paterson D. E., Taylor R. J. K. (2003). Confirmation
of the Structure of a Glucono-1,4-Lactone Derivative Obtained from
Silylation of Glucono-1,5-Lactone. Tetrahedron:
Asymmetry.

[26] Penteado F., Lopes E. F., Alves D., Perin G., Jacob R. G., Lenardão E. J. (2019). α-Keto Acids: Acylating Agents
in Organic Synthesis. Chem. Rev..

[27] Pusterla I., Bode J. W. (2012). The Mechanism of the α-Ketoacid–Hydroxylamine
Amide-Forming Ligation. Angew. Chem., Int. Ed..

[28] Pattabiraman V. R., Ogunkoya A. O., Bode J. W. (2012). Chemical Protein Synthesis by Chemoselective
α-Ketoacid–Hydroxylamine (KAHA) Ligations with 5-Oxaproline. Angew. Chem., Int. Ed..

[29] Deng R., Han T.-J., Gao X., Yang Y.-F., Mei G.-J. (2022). Further
Developments of β,γ-Unsaturated α-Ketoesters as
Versatile Synthons in Asymmetric Catalysis. iScience.

[30] Von
Der Ohe F., Brückner R. (1998). Z- or E-Configurated γ-Alkylidenebutenolides
from a 2-(Trimethylsiloxy)­Furan and Iodomethacrolein - Stereoselective
Synthesis of Z- and E-Freelingyne. Tetrahedron
Lett..

[31] Singh R. P., Foxman B. M., Deng L. (2010). Asymmetric Vinylogous
Aldol Reaction
of Silyloxy Furans with a Chiral Organic Salt. J. Am. Chem. Soc..

