# Supplementary material for: Spiroketal Synthesis Via Uncatalyzed Inverse Electron-Demand Hetero-Diels-Alder Reactions at Ambient Temperature
Source: J Am Chem Soc. 2026 Sep 17;148(38):40959–66. doi: 10.1021/jacs.6c09678 (PMC13632720; doi:10.1021/jacs.6c09678)
Supplement: Supplementary file 1 [file ja6c09678_si_001.pdf]

Spiroketal Synthesis Via Uncatalyzed Inverse Electron-Demand Hetero-Diels-Alder Reactions at Ambient Temperature

Emma J. Greene, Matthew C. Raeside, Marcelo A. Mazariego, Andy Y. Song, K. N. Houk*, and Patrick G. Harran*

Supplementary Information

Table of Contents

1. General Information....4

2. Experimental Procedures....5

2.1. Preparation of exocyclic enol ethers....5

2.1.1 Synthesis of **1c-1g** from iodohydrin ethers....5

2.1.2 Cp_2TiMe_2 mediated methylenation....7

2.1.3 Preparation of lactones **S8o**, **S8r**, and **S8s**....9

2.1.4 Preparation of 1-methylene-1,3-dihydroisobenzofuran **1j**....12

2.1.5 Preparation of uridine methylidene tetrahydrofuran **1k**....13

2.1.6 Preparation of 2,5-Anhydro-1-deoxy-D-gluco-hept-1-enitol **1l**....14

2.2. Preparation of vinyl vicinal tricarbonyl hydrates....15

2.2.1 Preparation of methylated vinyl vicinal tricarbonyl hydrates....15

2.3. Preparation of spiroketals and ene adducts....16

2.3.1 Complete screen of conditions for cycloaddition of **1q**....24

2.4. α-Keto ester derivatizations....24

2.4.1 Preliminary studies of keto ester degradation to carboxylic acid....28

2.5. Synthesis of **20**....28

3. References....31

4. NMR spectra....33

5. Crystallographic data....117

6. Computational Methods....132

6.1. Energies of 1i,o,q, 3i,o,q, 5i,o,q Transition States and Intermediates....132

6.2. Atomic Coordinates of 1i,o,q, 3i,o,q, 5i,o,q Transition States and Intermediates....133

6.3. References....175

1. General Information

Unless stated otherwise, reactions were performed in flame-dried glassware under positive pressure of argon at room temperature. Commercially obtained reagents were used as received unless otherwise specified. Anhydrous solvents were dried using an activated alumina solvent drying system. Methanol (MeOH) was dried over activated 3Å molecular sieves. Thin layer chromatography (TLC) was performed on pre-coated plates from Sorbent Technologies, silica gel 60 PF_{254} (0.25 mm). TLC were visualized with UV light (254 nm) and/or stained using $KMnO_4$ or cerium ammonium molybdate (CAM). Flash chromatography was performed on silica gel 60 (240-400 mesh). NMR spectra were recorded on a Bruker Advance (500 MHz or 600 MHz) spectrometer using $CDCl_3$, DMSO-d_6, or MeOD-d_4 as solvent and referenced relative to the residual solvent peak. Chemical shifts are reported in ppm and coupling constants (*J*) in Hertz. ^{13}C NMR and APT spectra were recorded on the same instruments (125 or 151 MHz) with total proton decoupling referenced relative to the residual solvent peak. HSQC, HMBC, COSY and NOESY NMR experiments were used to aid assignment of NMR peaks when required. Diastereomeric ratios (d.r.) were determined by ^{1}H NMR analysis of the crude reaction mixtures. High-resolution mass spectra were recorded on a Thermo Scientific Exactive® Mass Spectrometer with DART ID-CUBE Waters GST Premier or Agilent 6545 LC-QTOF. Optical rotations were measured with a Rudolf Autopol III Automatic Polarimeter.

2. Experimental Procedures

2.1. Preparation of exocyclic enol ethers

2.1.1 Elimination of iodohydrin ethers

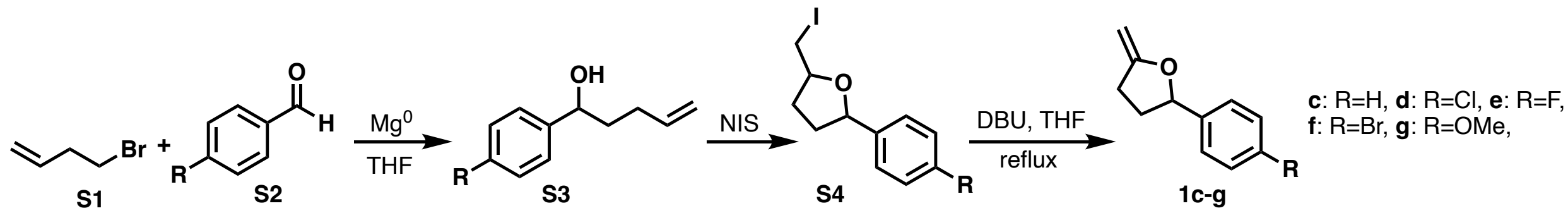

General Procedure 1: Elimination of iodohydrin ethers

Iodohydrin ethers were prepared according to literature procedures.[1] 2-iodomethyl tetrahydrofurans (1.0 equiv.) were dissolved in THF (0.4M). To this mixture, DBU was added dropwise, and the mixture was refluxed for 12 h. The mixture was then cooled to room temperature, filtered, and concentrated. The crude yellow oils were distilled to give pure methylene tetrahydrofurans as clear oils.

2-methylene-5-phenyltetrahydrofuran **(1c)**

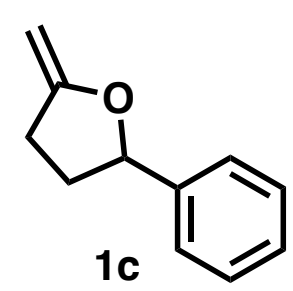

Prepared according to **General Procedure 1** using 2-(iodomethyl)-5-phenyltetrahydrofuran (**S4c**) (65:35 trans:cis) (18.8 mmol) and DBU (3.12 mL, 1.1 equiv, 20.7 mmol) in THF (47 mL). Distilled at 110 ºC and 20 mbar to give **1c** as a clear oil (16 mmol, 86 %). Spectral data was consistent with literature reports. **^{1}H NMR** (500 MHz, $CDCl_3$) δ 7.31 – 7.20 (m, 4H), 7.23 – 7.15 (m, 1H), 5.17 (dd, *J* = 7.9, 6.2 Hz, 1H), 4.25 (t, *J* = 1.8 Hz, 1H), 3.83 (t, *J* = 1.6 Hz, 1H), 2.59 (td, *J* = 7.5, 1.8 Hz, 2H), 2.32 (dq, *J* = 12.6, 6.3 Hz, 1H), 1.87 (dq, *J* = 12.3, 8.4 Hz, 1H). **^{13}C NMR** (126 MHz, $CDCl_3$) δ 162.74, 141.43, 128.27, 127.54, 125.38, 83.00, 79.09, 33.71, 29.15. **HRMS (ESI-TOF) m/z**: [M+H]+ Calcd. for $C_{11}H_{13}O$ 161.096; found 161.0939

2-(4-chlorophenyl)-5-methylenetetrahydrofuran **(1d)**

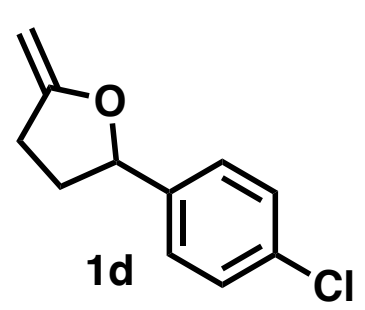

Prepared according to **General Procedure 1** using 2-(4-chlorophenyl)-5-(iodomethyl) tetrahydrofuran (19.5 mmol) (**S4d**) (63:37 trans:cis) and DBU (2.80 mL, 0.95 equiv, 18.6 mmol) in THF (48.83 mL). Distilled at 120 ºC and 20 mbar to give **1d** as a clear oil (14.9 mmol, 76 %). **^{1}H NMR** (500 MHz, $CDCl_3$) δ 7.24 – 6.90 (m, 4H), 5.05 – 4.97 (m, 1H), 4.13 (d, *J* = 1.7 Hz, 1H), 3.71 (d, *J* = 1.6 Hz, 1H), 2.48 – 2.42 (m, 2H), 2.19 (dq, *J* = 12.6, 6.3 Hz, 1H), 1.69 (dq, *J* = 12.3, 8.4 Hz, 1H). **^{13}C NMR** (126 MHz, $CDCl_3$) δ 162.61, 140.07, 133.43, 126.94, 82.40, 79.72, 33.82, 29.18. **HRMS (ESI-TOF) m/z**: [M+H]+ Calcd. for $C_{11}H_{12}ClO$ 195.0577; found 195.0568

2-(4-fluorophenyl)-5-methylenetetrahydrofuran **(1e)**

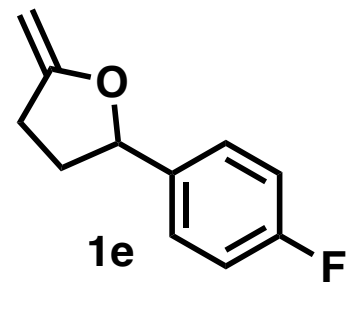

Prepared according to **General Procedure 1** using 2-(4-fluorophenyl)-5-(iodomethyl) tetrahydrofuran (22.0 mmol) (**S4e**) (74:26 trans:cis) and DBU (3.7 mL, 1.1 equiv, 24 mmol) in THF (56 mL). Distilled at 110 ºC and 20 mbar to give **1e** as a clear oil (16 mmol, 71 %). ^{1}H NMR (500 MHz, $CDCl_3$) δ 7.33 – 7.27 (m, 2H), 7.03 (t, *J* = 8.7 Hz, 2H), 5.21 (dd, *J* = 8.1, 6.2 Hz, 1H), 4.33 (s, 1H), 3.91 (s, 1H), 2.66 (ddt, *J* = 8.3, 6.2, 1.7 Hz, 2H), 2.37 (dq, *J* = 12.5, 6.2 Hz, 1H), 1.89 (dq, *J* = 12.2, 8.6 Hz, 1H). **^{13}C NMR** (126 MHz, $CDCl_3$) δ 163.31, 162.66, 137.24, 137.22, 127.30, 115.21, 82.55, 79.55, 33.86, 29.29. **HRMS (ESI-TOF) m/z**: [M+H]+ Calcd. for $C_{11}H_{12}FO$ 179.0867; found 179.0862

2-(4-bromophenyl)-5-methylenetetrahydrofuran **(1f)**

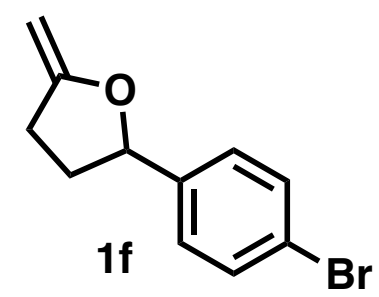

Prepared according to **General Procedure 1** using 2-(4-bromophenyl)-5-(iodomethyl) tetrahydrofuran (18.0 mmol) (**S4f**) (66:34 trans:cis) and DBU (3.1 mL, 1.1 equiv, 20 mmol) in THF (46 mL). Distilled at 125 ºC and 1 mbar to give **1f** as a clear oil (11.0 mmol, 61 %), 94.9% purity. **^{1}H NMR** (600 MHz, $CDCl_3$) δ 7.52 – 7.47 (m, 2H), 7.26 – 7.21 (m, 2H), 5.23 (dd, *J* = 7.8, 6.3 Hz, 1H), 4.36 (d, *J* = 1.7 Hz, 1H), 3.94 (d, *J* = 1.5 Hz, 1H), 2.68 (tq, *J* = 7.1, 1.8 Hz, 2H), 2.46 – 2.38 (m, 1H), 1.91 (dq, *J* = 12.3, 8.4 Hz, 1H). **HRMS (ESI-TOF) m/z**: [M+H]+ Calcd. for $C_{11}H_{12}BrO$ 239.0066; found 239.0097

2-(4-methoxyphenyl)-5-methylenetetrahydrofuran **(1g)**

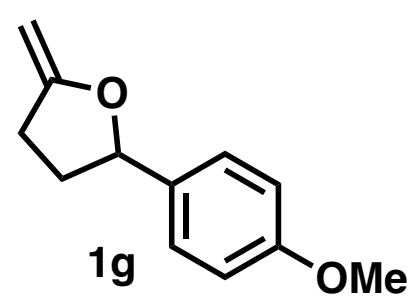

Prepared according to **General Procedure 1** using 2-(iodomethyl)-5-(4-methoxyphenyl) tetrahydrofuran (24.3 mmol) (**S4g**) (53:47 trans:cis) and DBU (3.48 mL, 0.95 equiv, 23.08 mmol) in THF (60.7 mL). Distilled at 110 ºC and 1 mbar to give **1g** as a clear oil. 63% yield (15.2 mmol), 82.4% purity (co-distilled with residual DBU and 10.7% endo isomer). **^{1}H NMR** (600 MHz, $CDCl_3$) δ 7.27 (d, *J* = 7.0 Hz, 2H), 6.89 (d, *J* = 8.7 Hz, 2H), 5.20 (t, *J* = 7.2 Hz, 1H), 4.30 (s, 1H), 3.89 (s, 1H), 3.80 (s, 3H), 2.71 – 2.64 (m, 2H), 2.35 (dq, *J* = 12.4, 6.2 Hz, 1H), 2.01 – 1.91 (m, 1H). **^{13}C NMR** (151 MHz, $CDCl_3$) δ 162.89, 159.30, 133.33, 127.06, 113.87, 83.10, 79.16, 55.31, 33.71, 29.51. **HRMS (ESI-TOF) m/z**: [M+H]+ Calcd. for $C_{12}H_{15}O_2$ 191.1067; found 191.1056

Preparation of 2-ethylidene-5-phenyltetrahydrofuran **(1p)**

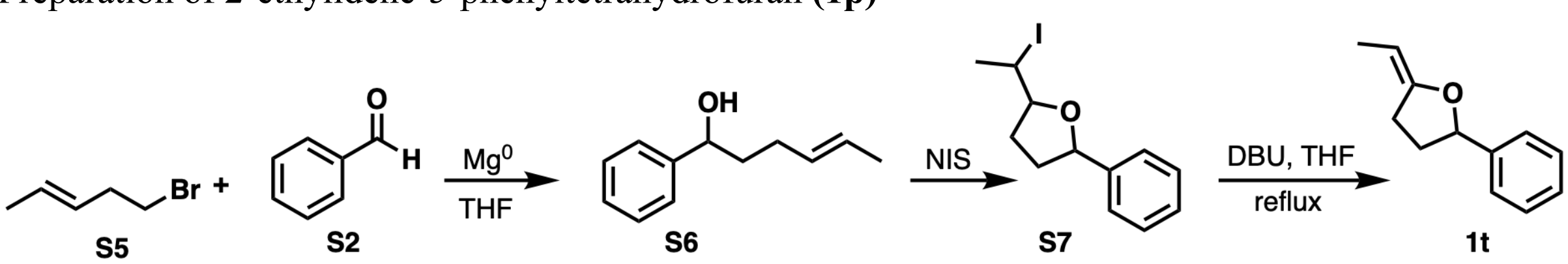

(E)-1-phenylhex-4-en-1-ol **(S6)**

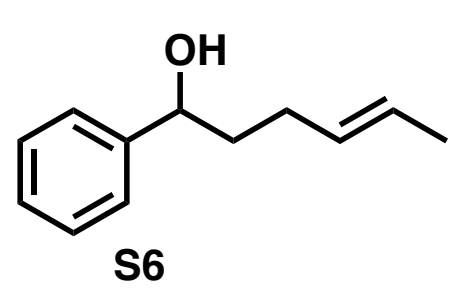

Magnesium turnings (1.3 equiv, 7.93 mmol) was charged into a two-necked round flask equipped with a condenser and a dropping funnel. To magnesium in Et2O was added a small bead of iodine and a small amount of **S5** in THF. The mixture was heated with heat gun until the color disappeared. A solution of (E)-5-bromopent-2-ene (prepared according to literature procedure)[2] (1.1 equiv, 6.71 mmol, 1.25M) (**S5)** in THF (5.37 mL) was added dropwise to the mixture, maintaining a gentle reflux. Once reaction mixture was cooled to room temperature and a solution of benzaldehyde (1.0 equiv, 6.10 mmol, 1 M) in THF (6.1 mL) was added dropwise to the mixture. The resulting mixture was stirred at room temperature for 1 h. The reaction was quenched with 1N HCl (20 mL) and the aqueous phase was extracted with EtOAc. The combined organic phase was washed with brine and dried over Na2SO4. After removal of the solvent, the resulting crude mixture was purified by silica gel column chromatography using 10-20% EtOAc in hexane to give (E)-1-phenylhex-4-en-1-ol (**S6)** as a pale yellow oil in 67% yield (4.08 mmol). **^{1}H NMR** (500 MHz, $CDCl_3$) δ 7.38 – 7.28 (m, 4H), 7.27 (dt, *J* = 7.7, 2.8 Hz, 1H), 5.45 (m, 2H), 4.72 – 4.64 (m, 1H), 2.14 – 1.98 (m, 2H), 1.91 – 1.81 (m, 1H), 1.81 – 1.70 (m, 1H), 1.65 (d, *J* = 3.7 Hz, 3H). **^{13}C NMR** (126 MHz, $CDCl_3$) δ 144.72,

130.62, 128.45, 127.52, 125.93, 125.59, 74.13, 38.75, 28.94, 17.96. **HRMS (ESI-TOF) m/z**: [M+H]+ Calcd. for $C_{12}H_{17}O$ 177.1274; found 177.1295

2-(1-iodoethyl)-5-phenyltetrahydrofuran **(S7)**

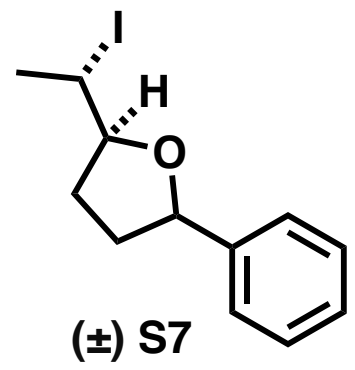

(±) S7

To a solution of (E)-1-phenylhex-4-en-1-ol (1.0 equiv, 2.04 mmol) in dry DCM (2.32 mL, 0.88M) at 0ºC was added N-iodosuccinimide (1.1 equiv, 2.25 mmol) portionwise and the reaction was warmed to room temperature for 2h. The solvent was then removed in vacuo and remaining oil containing both diastereomers (trans: cis = 4:1) was purified by flash column chromatography using 10% EtOAc in hexanes to yield 2-(1-iodoethyl)-5-phenyltetrahydrofuran (**S7**) as a light yellow oil in 85% yield (1.74 mmol, 54:46 trans:cis*, 83% purity). **^{1}H NMR** (500 MHz, $CDCl_3$) δ 7.42 – 7.21 (m, 5H), 5.16 – 5.06 (m, 1H), 4.30 (h, J = 6.8 Hz, 1H), 3.97 (q, J = 7.1 Hz, 1H), 2.45 – 2.15 (m, 2H), 1.97 (dd, J = 17.9, 6.9 Hz, 3H), 1.93 – 1.71 (m, 2H). **^{13}C NMR** (126 MHz, $CDCl_3$) δ 142.79, 142.29, 128.41, 128.39, 128.36, 127.60, 127.48, 127.39, 125.99, 125.81, 125.67, 84.22, 83.86, 82.37, 81.61, 80.06, 79.68, 38.89, 37.28, 35.49, 34.28, 34.12, 33.76, 33.02, 32.96, 31.72, 24.98, 24.92, 22.26. **HRMS (ESI-TOF) m/z**: [M+H]+ Calcd. for $C_{12}H_{16}IO$ 303.0240; found 303.0244

** trans:cis ratio refers to the relationship between the substituents on the THF ring. The iodo group is always syn to it's alpha methine proton.*

(E)-2-ethylidene-5-phenyltetrahydrofuran **(1t)**

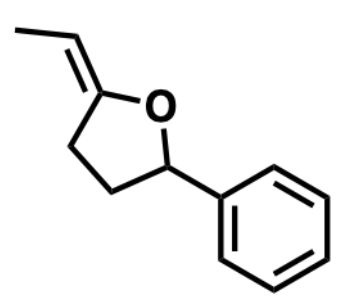

Prepared according to **General Procedure 1** using 2-(1-iodoethyl)-5-phenyltetrahydrofuran (1.74 mmol) (**S7**) (54:46 trans:cis) and DBU (0.29 mL, 1.1 equiv, 1.91 mmol) in THF (46 mL). Distilled at 95 ºC and 1 mbar to give **the product** (0.88 mmol, 51% yield, 88% purity) as a light-yellow oil that co-distilled with residual DBU. The expected E-configuration of the ethylidene ether was confirmed via 1H-1H NOSEY. **^{1}H NMR** (500 MHz, $CDCl_3$) δ 7.34 (d, J = 4.5 Hz, 4H), 7.28 (q, J = 4.6 Hz, 1H), 5.17 (t, J = 7.1 Hz, 1H), 4.85 (qd, J = 7.0, 3.6 Hz, 1H), 2.70 – 2.51 (m, 2H), 2.45 – 2.35 (m, 1H), 1.94 (dq, J = 12.3, 8.5 Hz, 1H), 1.60 (d, J = 7.1 Hz, 3H). **^{13}C NMR** (126 MHz, $CDCl_3$) δ 156.26, 141.88, 128.43, 127.62, 125.62, 89.80, 81.99, 33.47, 25.90, 12.29. **HRMS (ESI-TOF) m/z**: [M+H]+ Calcd. for $C_{12}H_{15}O$ 175.1117; found 175.1122

2.1.2 Cp_2TiMe_2 mediated methylenation

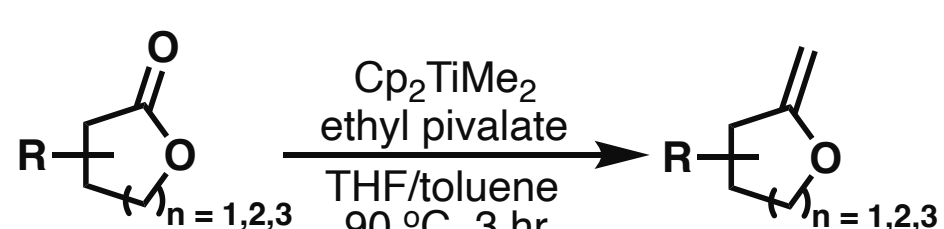

General Procedure 2: Cp_2TiMe_2 mediated methylenation of lactones

To a vial containing lactone **S8** (1.0 equiv.) was added a solution of Petasis reagent (2.0 equiv.) in THF/PhMe. Additional THF was added such that the concentration of the lactone was 0.2M. Ethyl pivalate (1.0 equiv.) was added to the mixture as a sacrificial ester. The vial was sealed, heated to 90ºC, and stirred at this temperature for 3h. After cooling to room temperature, the contents of the vial were poured into a flask containing at least 3x the volume of pentane, causing Ti residue precipitate as a yellow solid. After 1h of stirring at room temperature, the residue was filtered quickly through a small pad of celite and washed with additional pentane. If more precipitate is observed during concentration, the filtration may be repeated.

The filtrate was concentrated under reduced pressure at 23ºC to give crude enol ether **1** as an orange oil. **1** was dissolved in a known volume of DCM and carried forward crude. Yield was calculated based on concentration of **1** using ^{1}H NMR.

2-butyl-5-methylenetetrahydrofuran **(1i)**

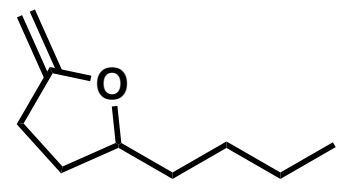

1i

Prepared according to **General Procedure 2** using commercial 5-butyldihydrofuran-2(3H)-one (**S8i**) (1.0 equiv, 1.05 mmol). Petasis reagent (2 equiv, 1.53 mL, 32.3% wt, 2.11 mmol), ethyl pivalate (1.0 equiv, 1.05 mmol) in THF (2.64 mL). Obtained as a crude yellow oil in 86% yield (0.91 mmol). **^{1}H NMR** (600 MHz, $CDCl_3$) δ 4.20 (p, *J* = 6.7 Hz, 1H), 4.16 (s, 1H), 3.77 (d, *J* = 2.4 Hz, 1H), 2.60 – 2.51 (m, 2H), 2.10 – 2.02 (m, 1H), 1.71 – 1.64 (m, 1H), 1.64 – 1.56 (m, 1H), 1.51 (tt, *J* = 12.2, 5.4 Hz, 1H), 1.45 – 1.40 (m, 1H), 1.35 (dd, *J* = 13.4, 6.6 Hz, 3H), 0.93 – 0.90 (m, 4H). **^{13}C NMR** (151 MHz, $CDCl_3$) δ 163.00, 82.67, 78.36, 34.98, 30.69, 29.39, 28.01, 22.69, 14.02.**HRMS (ESI-TOF) m/z**: [M+H]+ Calcd. for $C_9H_{17}O$ 141.1274; found 140.1306

2-butyl-6-methylenetetrahydro-2H-pyran **(1q)**

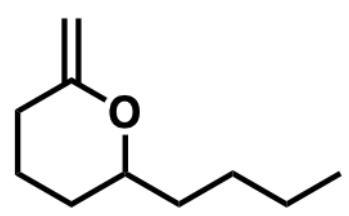

Prepared according to **General Procedure 2** using commercial 6-butyltetrahydro-2H-pyran-2-one (**S8q**) (1.0 equiv, 1.60 mmol). Petasis reagent (2 equiv, 1.53 mL, 27.4% wt, 3.20 mmol), ethyl pivalate (1.0 equiv, 1.60 mmol) in THF (4.00 mL). Obtained as a crude yellow oil in 87% yield (1.39 mmol). **1H NMR** (600 MHz, CDCl3) δ 4.30 (s, 1H), 4.04 (s, 1H), 3.57 (tq, *J* = 7.6, 2.6 Hz, 1H), 2.24 (dt, *J* = 13.8, 4.6 Hz, 1H), 2.16 – 2.08 (m, 1H), 1.81 (dq, *J* = 13.1, 4.2 Hz, 1H), 1.75 – 1.67 (m, 1H), 1.64 – 1.54 (m, 2H), 1.50 – 1.39 (m, 3H), 1.33 (dp, *J* = 15.6, 4.4 Hz, 3H), 0.91 (d, *J* = 7.1 Hz, 3H). **^{13}C NMR** (151 MHz, $CDCl_3$) δ 160.63, 90.88, 79.24, 45.53, 35.76, 30.33, 29.03, 27.52, 22.69, 14.09. **HRMS (ESI-TOF) m/z:** [M+H]+ Calcd. for $C_{10}H_{19}O$ 155.1430; found 155.1469

2-butyl-7-methyleneoxepane **(1o)**

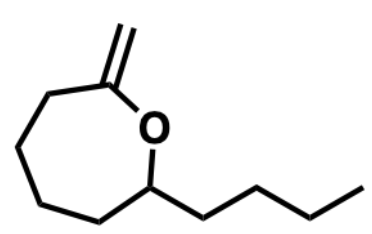

Prepared according to **General Procedure 2** using 7-butyloxepan-2-one (**S8o**) (1 equiv, 1.18 mmol), Petasis reagent (2 equiv, 1.37 mL, 32.3% wt, 2.35 mmol), ethyl pivalate (1.0 equiv, 1.18 mmol) in THF (2.97 mL). Obtained as a crude solution in DCM (1.9 M) in 75% yield (148 mg). **1H NMR (**500 MHz, $CDCl_3$) δ 4.10 (s, 1H), 3.86 (d, *J* = 1.3 Hz, 1H), 3.83 (td, *J* = 8.4, 4.0 Hz, 1H), 2.48 – 2.41 (m, 1H), 2.30 (ddt, *J* = 15.3, 11.8, 1.7 Hz, 1H), 1.91 – 1.81 (m, 2H), 1.78 (dddd, *J* = 14.2, 5.5, 2.5, 0.9 Hz, 1H), 1.63 – 1.57 (m, 1H), 1.47 – 1.38 (m, 5H), 1.36 – 1.30 (m, 4H), 0.90 (t, *J* = 7.2 Hz, 3H). **HRMS (ESI-TOF) m/z:** [M+H]+ Calcd. for $C_{11}H_{21}O$ 169.1587; found 169.1578

(2R,5R)-4-methylene-2,5-diphenyl-1,3-dioxolane **(1n)**

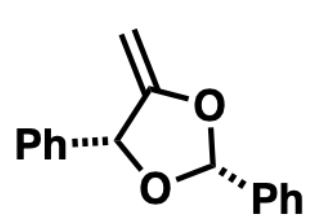

Prepared according to **General Procedure 2** using (2R,5R)-2,5-diphenyl-1,3-dioxolan-4-one (**S8n**) (1.0 equiv, 0.832 mmol), Petasis reagent (2 equiv, 0.944 mL, 38.4% wt, 1.66 mmol), ethyl pivalate (1.0 equiv, 0.832 mmol) in THF (2.77 mL). Obtained as a crude solution in DCM in 88% yield (0.734 mmol). **^{1}H NMR** (500 MHz, $CDCl_3$) δ 7.65 – 7.33 (m, 8H), 7.25 – 7.11 (m, 2H), 6.24 (s, 1H), 5.58 (s, 1H), 4.44 (d, *J* = 2.3 Hz, 1H), 3.68 (d, *J* = 2.2 Hz, 1H).

cis-5-butyl-3-methyl-2-methylenetetrahydrofuran **((±)-1r)**

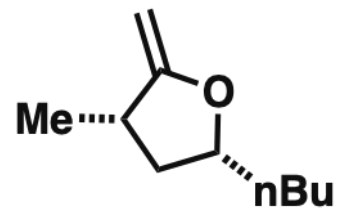

Prepared according to **General Procedure 2** using *cis*-5-butyl-3-methyldihydrofuran-2(3H)-one (**S8r**) (1.0 equiv, 960 μmol), Petasis reagent (2 equiv, 1.09 mL, 38.4% wt, 1.09 mmol), ethyl pivalate (1.0 equiv, 960 μmol) in THF (3.20 mL). Obtained **product** as a crude yellow oil in 61% yield (0.58 mmol). **^{1}H NMR** (600 MHz, $CDCl_3$) δ 4.16 (q, *J* = 1.5 Hz, 1H), 4.14 – 4.07 (m, 1H), 3.70 (t, *J* = 1.7 Hz, 1H), 2.84 – 2.72 (m, 1H), 2.23 – 2.14 (m, 1H), 1.74 – 1.65 (m, 1H), 1.56 – 1.48 (m, 1H), 1.46 – 1.39 (m, 1H), 1.37 – 1.32 (m, 2H), 1.28 – 1.22 (m, 2H), 1.18 (dd, *J* = 6.8, 1.3 Hz, 3H), 0.91 (t, *J* = 7.0 Hz, 3H). **^{13}C NMR** (151 MHz, $CDCl_3$) δ 168.07, 80.48, 77.10, 39.96, 36.44, 35.04, 27.99, 22.71, 17.40, 14.01. **HRMS (ESI-TOF) m/z:** [M+H]+ Calcd. for $C_{10}H_{19}O$ 155.1430; found 154.1449

cis-6-butyl-3-methyl-2-methylenetetrahydro-2H-pyran **((±)-1s)**

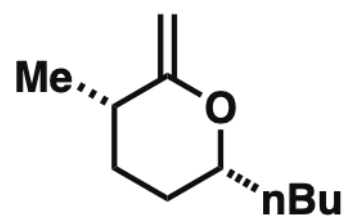

Prepared according to **General Procedure 2** using *cis*-6-butyl-3-methyltetrahydro-2H-pyran-2-one (**S8s**) (1.0 equiv, 587 μmol), Petasis reagent (2 equiv, 0.67 mL, 38.4% wt, 1.17 mmol), ethyl pivalate (1.0 equiv, 587 μmol) in THF (1.96 mL). Obtained as a crude yellow solution in DCM in 84% yield (0.49 mmol).**^{1}H NMR** (600 MHz, $CDCl_3$) δ 4.28 (s, 1H), 4.08 – 4.03 (m, 1H), 3.79 – 3.67 (m, 1H), 2.42 (q, *J* = 6.5 Hz, 1H), 1.65 (dd, *J* = 6.9, 3.4 Hz, 1H), 1.61 (d, *J* = 5.4 Hz, 1H), 1.52 – 1.46 (m, 1H), 1.42 (d, *J* = 9.5 Hz, 2H), 1.33 (s, 3H), 1.25 (d, *J* = 6.9 Hz, 1H), 1.13 (d, *J* = 6.9 Hz, 3H), 0.91 (t, *J* = 7.1 Hz, 3H). **HRMS (ESI-TOF) m/z:** [M+H]+ Calcd. for $C_{10}H_{21}O$ 169.1587; found 169.1584

2.1.3 Preparation of lactones S8o, S8r, and S8s

Preparation of caprolactone **(S8o)**

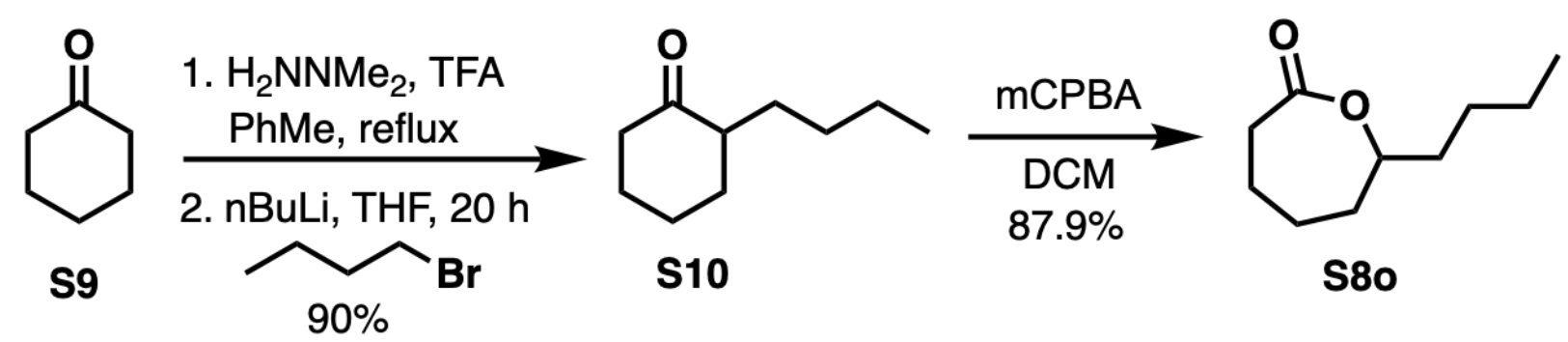

2-butylcyclohexan-1-one **(S10)**

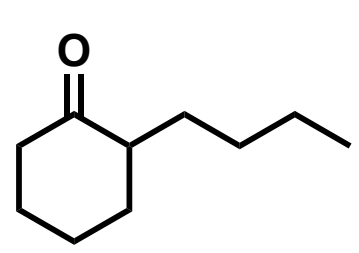

Cyclohexanone (1.0 equiv, 51 mmol), 1,1-dimethylhydrazine (1.20 equiv, 61 mmol), 2,2,2-trifluoroacetic acid (27 μL, 0.36 mmol), and toluene (20 mL) were added to a flask equipped with a dean-stark apparatus. The mixture was heated under reflux for 5 h and then cooled to room temperature. The reaction mixture was diluted with ether (100 mL) and water (25 mL). The organic layer was washed with brine and dried over Na_2SO_4. The filtrate was carefully concentrated under reduced pressure. Without further purification, A solution of cycloalkanone 2-cyclohexylidene-1,1-dimethylhydrazine (1.0 equiv, 3.57 mmol) in THF was prepared (1.78 mL) and *n*-BuLi in hexane (1.1 equiv, 3.92 mmol, 2.65 M, 1.5 mL) was added at −5 °C under an argon atmosphere. After the mixture stirred for 1h at this temperature, 1-bromobutane (1.05 equiv, 3.74 mmol) was added, and stirring was continued for at room temperature for 20h. Upon completion, aqueous 2M HCl was added to the reaction mixture. After stirring for 1 h, the mixture was extracted 3x with EtOAc. The organic layer was washed with water and brine and dried over Na_2SO_4. The crude mixture was purified via silica gel column chromatography using 5% EtOAc in hexane to yield 2-butylcyclohexan-1-one (**S10**)

as a clear oil (90% yield, 3.21 mmol). Spectral data was consistent with literature reports.[3] **^{1}H NMR** (600 MHz, $CDCl_3$) δ 2.41 (dtd, J = 13.5, 4.3, 1.4 Hz, 1H), 2.36 – 2.23 (m, 2H), 2.17 – 2.09 (m, 1H), 2.09 – 1.98 (m, 1H), 1.91 – 1.85 (m, 1H), 1.85 – 1.76 (m, 1H), 1.76 – 1.63 (m, 2H), 1.47 – 1.16 (m, 6H), 0.91 (t, J = 7.2 Hz, 3H).

7-butyloxepan-2-one **(S8o)**

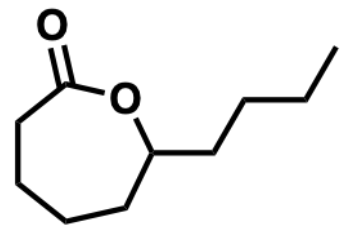

To a solution of an 2-butylcyclohexan-1-one (**S10**) (1.0 equiv, 3.21 mmol) in DCM (16.0 mL) was added *m*CPBA (75% wt, 2.0 equiv, 6.42 mmol) at 0 °C. The solution was allowed to warm to room temperature and stirred for 2d. Then, the reaction mixture was poured into aqueous Na_2SO_3 (1 M). The aqueous layer was extracted 3x with DCM. The combined organic extracts were washed with sat. aq. $NaHCO_3$ and brine and dried over Na2SO4. The crude mixture was concentrated and purified by silica gel flash chromatography 20% Et_2O in pentane to give a colorless oil (88% yield, 2.82 mmol). Spectral data was consistent with literature reports.[4] **^{1}H NMR** (600 MHz, $CDCl_3$) δ 4.25 (td, J = 8.2, 4.7 Hz, 1H), 2.73 – 2.58 (m, 2H), 2.01 – 1.88 (m, 3H), 1.79 – 1.70 (m, 1H), 1.68 – 1.61 (m, 2H), 1.59 – 1.52 (m, 2H), 1.52 – 1.45 (m, 1H), 1.40 – 1.31 (m, 3H), 0.97 – 0.90 (m, 3H).

Cis methyl substituted lactones **1r** and **1s** were prepared according to literature preparation of similar compounds.[5]

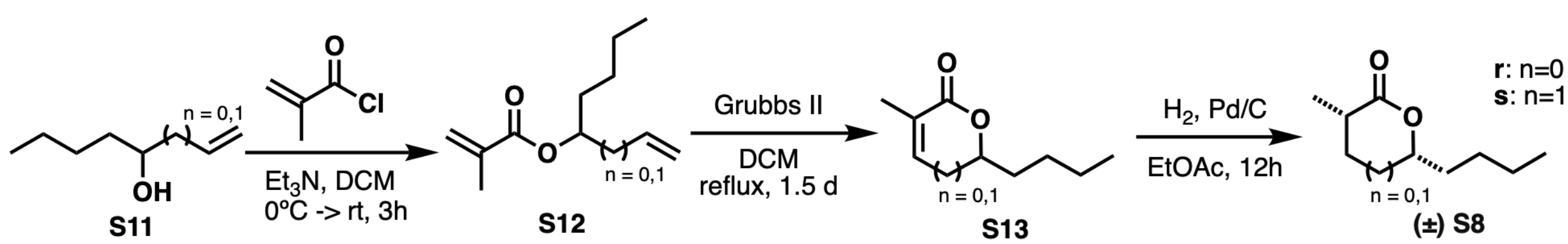

General Procedure 3: Methacrylate esters **S12**

A 0.3 M solution of the alcohol **S11** (1.0 equiv) and Et_3N (1.38 equiv) in dry DCM was cooled to 0 °C followed by the slow addition of methacryloyl chloride (1.0 equiv). The mixture was stirred at room temperature overnight and then quenched by the addition of an equal volume of 1 N HCl. The layers were separated and the aqueous layer was extracted three times with dichloromethane. The combined organic layers were washed with saturated aqueous $NaHCO_3$ and brine, dried over Na_2SO_4, and concentrated under reduced pressure. The residue was purified by column chromatography on silica gel (hexane/ethyl acetate 20:1) to yield the methacrylate ester **S12** as a colourless oil.

General Procedure 4: Ring-closing metathesis towards **S13**

Grubbs 2nd generation catalyst (0.05 equiv) was added to a solution of methacrylate ester **S12** (1.0 equiv) in dry DCM (0.05 M). The mixture was stirred under reflux for 2.5 d. The solvent was removed under reduced pressure and the residue was purified by silica gel column chromatography (pentane/diethyl ether 3:1) to give the **S13** as a colourless oil.

General Procedure 5: Hydrogenation of **S13** to **S8**

To a solution of the lactone **S13** (1.0 equiv) in ethyl acetate (0.15 M) Pd/C (10% Pd, 0.1 equiv) was added. The mixture was stirred in a hydrogen atmosphere (45 bar) for 4-12 h at 23°C. The catalyst was removed by filtration over a pad of celite, the solvent was evaporated under reduced pressure, and the

residue was purified by silica gel column chromatography (pentane/ diethyl ether 3:1) to yield the *cis* methylated lactone **S8** as a colourless oil.

hept-1-en-3-yl methacrylate **(S12r)**

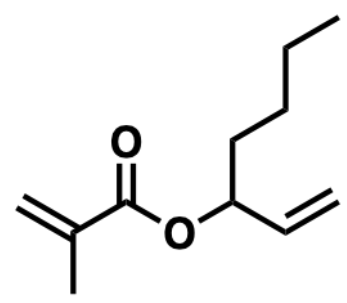

Prepared according to **General Procedure 3** using hept-1-en-3-ol (**S11r**) (1.0 equiv, 7.44 mmol), triethylamine (1.38 equiv, 10.3 mmol, 1.43 mL) and methacryloyl chloride (1.0 equiv, 7.44 mmol) in DCM (25 mL). 51.6% yield (3.84 mmol). **^{1}H NMR** (500 MHz, $CDCl_3$) δ 6.12 (d, *J* = 1.6 Hz, 1H), 5.81 (ddd, *J* = 17.0, 10.5, 6.2 Hz, 1H), 5.55 (t, *J* = 1.7 Hz, 1H), 5.33 – 5.20 (m, 2H), 5.16 (dt, *J* = 10.6, 1.2 Hz, 1H), 1.95 (s, 3H), 1.66 (dddd, *J* = 27.0, 14.2, 7.5, 3.1 Hz, 2H), 1.39 – 1.25 (m, 4H), 0.94 – 0.84 (m, 3H). **^{13}C NMR** (126 MHz, $CDCl_3$) δ 166.77, 136.69, 125.22, 116.37, 75.04, 33.94, 27.19, 22.48, 18.35, 13.98. **HRMS (ESI-TOF) m/z:** [M+H]+ Calcd. for $C_{11}H_{19}O_2$ 183.1380; found 183.1428

oct-1-en-4-yl methacrylate **(S12s)**

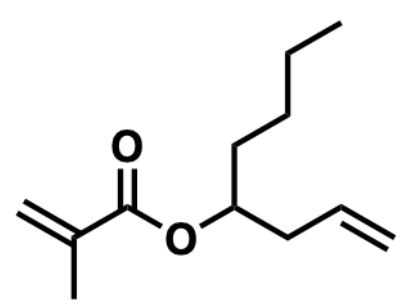

Prepared according to **General Procedure 3** using oct-1-en-4-ol[6] (**S11s**) (1.0 equiv, 11.0 mmol), triethylamine (1.38 equiv, 15.0 mmol, 2.1 mL) and methacryloyl chloride (1.0 equiv, 11.0 mmol) in DCM (36 mL). 65% yield (7.1 mmol). **^{1}H NMR** (500 MHz, C_6D_6) δ 6.08 (d, *J* = 1.7 Hz, 1H), 5.77 (ddt, *J* = 17.2, 10.2, 7.1 Hz, 1H), 5.53 (q, *J* = 1.7 Hz, 1H), 5.14 – 5.02 (m, 2H), 4.97 (p, *J* = 6.1 Hz, 1H), 2.42 – 2.30 (m, 2H), 1.93 (s, 3H), 1.60 (ddt, *J* = 10.9, 6.0, 2.7 Hz, 2H), 1.40 – 1.25 (m, 4H), 0.96 – 0.84 (m, 3H). **^{13}C NMR** (126 MHz, C_6D_6) δ 133.73, 124.91, 117.51, 77.20, 76.94, 76.69, 73.58, 38.51, 33.16, 27.37, 22.47, 18.28, 13.91. **HRMS (ESI-TOF) m/z:** [M+H]+ Calcd. for $C_{12}H_{21}O_2$ 197.1536; found 197.1516

5-butyl-3-methylfuran-2(5H)-one **(S13r)**

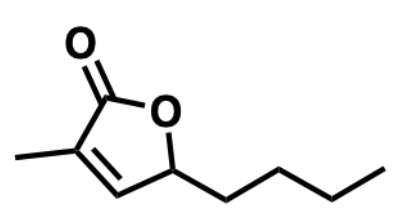

Prepared according to **General Procedure 4** using hept-1-en-3-yl methacrylate (**S12r**) (1.0 equiv, 3.57 mmol) and Grubbs 2nd generation catalyst (0.05 equiv, 178 µmol) in DCM (71.3 mL). 85.5% yield (3.05 mmol). **^{1}H NMR** (600 MHz, $CDCl_3$) δ 7.02 (q, *J* = 1.6 Hz, 1H), 4.87 (tq, *J* = 5.7, 1.9 Hz, 1H), 1.91 (s, 3H), 1.75 – 1.67 (m, 1H), 1.63 (ddd, *J* = 17.8, 9.0, 4.6 Hz, 1H), 1.47 – 1.31 (m, 4H), 0.94 – 0.88 (m, 3H). **^{13}C NMR (**126 MHz, $CDCl_3$) δ 174.36, 148.76, 129.89, 81.16, 33.20, 27.13, 22.45, 13.87, 10.65. **HRMS (ESI-TOF) m/z:** [M+H]+ Calcd. for $C_9H_{15}O_2$ 155.1067; found 155.1055

6-butyl-3-methyl-5,6-dihydro-2H-pyran-2-one **(S13s)**

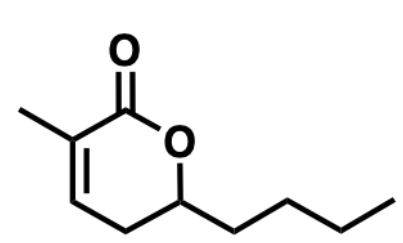

Prepared according to **General Procedure 4** using oct-1-en-4-yl methacrylate (**S12s**) (1.0 equiv, 6.6 mmol) and Grubbs 2nd generation catalyst (0.05 equiv, 0.33 mmol) in DCM (130 mL). 31% yield (2.08 mmol). **^{1}H NMR** (500 MHz, $CDCl_3$) δ 6.56 (tq, *J* = 3.3, 1.6 Hz, 1H), 4.36 (dq, *J* = 12.2, 6.4 Hz, 1H), 2.28 (ddt, *J* = 5.9, 3.9, 2.1 Hz, 2H), 1.91 (s, 3H), 1.82 – 1.73 (m, 1H), 1.61 (ddt, *J* = 13.3, 10.5, 5.0 Hz, 2H), 1.48 (tt, *J* = 8.6, 3.5 Hz, 1H), 1.35 (qq, *J* = 9.0, 4.4 Hz, 3H), 0.90 (t, *J* = 7.1 Hz, 3H). **HRMS (ESI-TOF) m/z:** [M+H]+ Calcd. for $C_{10}H_{17}O_2$ 169.1223; found 169.1261

cis-5-butyl-3-methyldihydrofuran-2(3H)-one **((±)-S8r)**

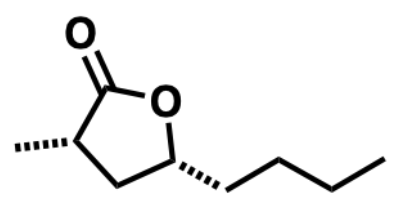

Prepared according to **General Procedure 5** using 5-butyl-3-methylfuran-2(5H)-one (**S13r**) (1.0 equiv, 3.05 mmol) and 10% wt. Pd/C (0.01 equiv, 30.5 μmol) in EtOAc (20 mL) under hydrogen atmosphere (45 bar) for 12 h. 96 % yield (2.9 mmol). **^{1}H NMR** (500 MHz, $CDCl_3$) δ 4.32 (ddt, *J* = 10.7, 7.6, 5.5 Hz, 1H), 2.65 (ddq, *J* = 11.9, 8.8, 7.1 Hz, 1H), 2.47 (ddd, *J* = 12.5, 8.5, 5.4 Hz, 1H), 1.79 – 1.69 (m, 1H), 1.59 (ddt, *J* = 15.3, 10.4, 5.2 Hz, 1H), 1.53 – 1.45 (m, 1H), 1.45 – 1.30 (m, 4H), 1.26 (d, *J* = 7.3 Hz, 3H), 0.90 (t, *J* = 7.0 Hz, 3H). **^{13}C NMR** (126 MHz, $CDCl_3$) δ 179.64, 78.72, 37.39, 35.94, 35.21, 27.40, 22.45, 15.14, 13.92. **HRMS (ESI-TOF) m/z:** [M+H]+ Calcd. for $C_9H_{17}O_2$ 157.1223; found 157.1245

cis-6-butyl-3-methyltetrahydro-2H-pyran-2-one **((±)-S8s)**

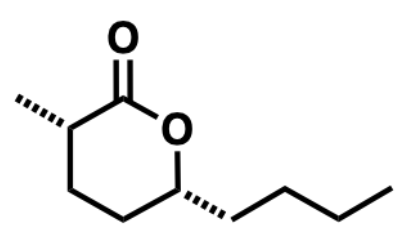

Prepared according to **General Procedure 5** using 6-butyl-3-methyl-5,6-dihydro-2H-pyran-2-one (**S13s**) (1.0 equiv, 2.08 mmol) and 10% wt. Pd/C (0.1 equiv, 208 μmol) in EtOAc (13.9 mL) under hydrogen atmosphere (45 bar) for 4h. 86.1% yield (1.79 mmol), 86% purity. **^{1}H NMR** (500 MHz, $CDCl_3$) δ 4.27 (ddq, *J* = 10.2, 7.2, 3.0 Hz, 1H), 2.66 – 2.52 (m, 1H), 2.12 – 2.02 (m, 1H), 1.95 – 1.84 (m, 1H), 1.74 – 1.65 (m, 1H), 1.65 – 1.44 (m, 5H), 1.38 – 1.31 (m, 3H), 1.21 (d, *J* = 6.8 Hz, 3H), 0.90 (t, *J* = 7.2 Hz, 3H). **^{13}C NMR** (126 MHz, $CDCl_3$) δ 176.48, 78.18, 35.05, 33.17, 27.26, 26.70, 25.62, 22.51, 16.20, 13.94. **HRMS (ESI-TOF) m/z:** [M+H]+ Calcd. for $C_{10}H_{19}O_2$ 171.1380; found 171.1412

2.1.4 Preparation of 1-methylene-1,3-dihydroisobenzofuran (1j)[7]

(2-((trimethylsilyl)ethynyl)phenyl)methanol **(S23)**

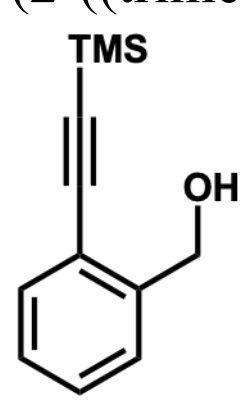

Prepared using a modified version of literature protocol. 2-iodo benzyl alcohol (562mg, 1eq., 2.4mmol) was dissolved in TEA and DMF (2mL, 5mL) and sparged with Ar. TMS acetylene (0.4mL, 1.2eq., 2.88mmol), CuI (23mg, 0.05eq., 0.12mmol), and $Pd(PPh_3)Cl_2$ (101mg, 0.06eq., 0.14mmol) were added and the mixture was stirred at 50˚C for 12h. The reaction was quenched with sat. NH_4Cl, extracted with EtOAc, washed with brine, dried, and concentrated. Purification on silica gel (0-10% EtOAc/Hex) lent the product in 95% yield (468mg). Spectral data was consistent with literature reports. **^{1}H NMR** (500 MHz, Chloroform-*d*) δ 7.47 (dd, *J* = 7.6, 1.5 Hz, 1H), 7.41 (d, *J* = 7.9 Hz, 1H), 7.33 (td, *J* = 7.6, 1.5 Hz, 1H), 7.24 (td, *J* = 7.9, 1.8 Hz, 1H), 4.82 (s, 2H), 1.65 (bs, 1H), 0.27 (s, 9H). **^{13}C NMR** (126 MHz, $CDCl_3$) δ 143.27, 132.57, 129.10, 127.51, 127.34, 121.31, 102.72, 99.74, 64.24, 0.07. **HRMS (DART) m/z**: [M+H]+ Calcd. for $C_{12}H_{17}OSi^+$ 205.1043; found 205.10400; 1.46 ppm mass defect.

(2-ethynylphenyl)methanol **(S24)**

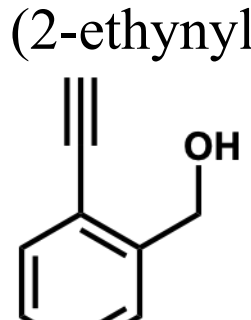

Prepared using a modified version of literature protocol. (2-((trimethylsilyl)ethynyl)phenyl)methanol (465mg, 1eq., 2.28mmol) was dissolved in MeOH (4.6mL, 0.5M). TBAF (1M in THF, 2.3mL) was added and the solution was stirred until complete. The reaction was quenched with brine, extracted with EtOAc, dried, and concentrated. Purification on silica gel (0-10% EtOAc/Hex) lent the product in 30% yield (90mg). Spectral data was consistent with literature reports. **^{1}H NMR** (500 MHz, Chloroform-*d*) δ 7.51 (dd, *J* = 7.6, 1.4 Hz, 1H), 7.45 (d, *J* = 7.7 Hz, 1H), 7.38 (td, *J* = 7.5, 1.4 Hz, 1H), 7.27 (d, *J* = 7.5 Hz, 1H), 4.84 (s, 2H), 3.34 (s, 1H), 1.64 (bs, 1H). **^{13}C NMR** (126 MHz, $CDCl_3$) δ 143.34, 133.03, 129.40, 127.62, 127.50, 120.39, 82.09,

81.40, 64.01. **HRMS (DART) m/z**: [M+H]+ Calcd. for $C_9H_9O^+$ 133.0648; found 133.06462; 1.35 ppm mass defect.

1-methylene-1,3-dihydroisobenzofuran **(1j)**

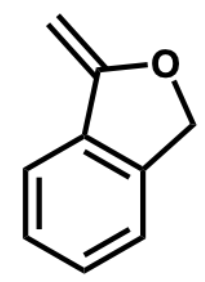

Prepared using a modified version of literature protocol. (2-ethynylphenyl)methanol (66mg, 1eq., 0.5mmol) was dissolved in DMSO (1mL, 0.5M). KOtBu (5.6mg, 0.1eq., 0.05mmol) was added and the solution was stirred at 80˚C for 5h. The reaction was quenched with brine, extracted with ether, dried, and concentrated. Quant. yield (66mg). Spectral data was consistent with literature reports. **^{1}H NMR** (600 MHz, Chloroform-*d*) δ 7.51 (d, *J* = 7.0 Hz, 1H), 7.35 (q, *J* = 6.6, 5.8 Hz, 2H), 7.30 (d, *J* = 7.0 Hz, 1H), 5.32 (s, 2H), 4.51 (s, 1H), 4.47 (s, 1H). **^{13}C NMR** (151 MHz, $CDCl_3$) δ 162.08, 140.20, 133.66, 129.17, 128.07, 121.36, 120.78, 77.89, 73.79. **HRMS (DART) m/z**: [M+H]+ Calcd. for $C_9H_9O^+$ 133.0648; found 133.06461; 1.43 ppm mass defect.

2.1.5 Preparation of uridine methylidene tetrahydrofuran (1k)[8]

1-((2*R*,3*R*,4*S*,5*S*)-3,4-dihydroxy-5-(iodomethyl)tetrahydrofuran-2-yl)pyrimidine-2,4(1*H*,3*H*)-dione **(S25)**

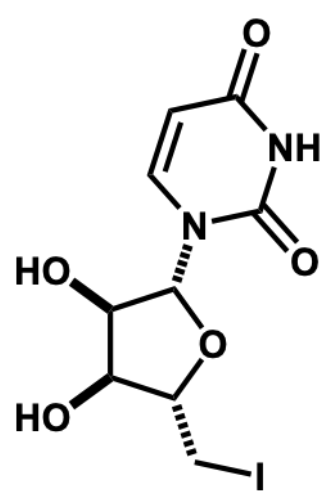

Prepared using a modified version of literature protocol. Uridine (244mg, 1eq., 1mmol), PPh_3 (380mg, 1.45eq., 1.45mmol), and imidazole (100mg, 1.45eq., 1.45mmol) were dissolved in THF (3.3mL, 0.3M). I_2 (267mg, 1.05eq., 1.05mmol) was added and the solution was stirred until complete. The reaction was quenched with water and concentrated. Purification on silica gel (5-7% MeOH/DCM) lent the product in quant. yield (354mg). Spectral data was consistent with literature reports. **^{1}H NMR** (600 MHz, DMSO-d_6) δ 11.38 (s, 1H), 7.67 (d, *J* = 8.0 Hz, 1H), 7.42 (s, 1H), 5.80 (d, *J* = 5.7 Hz, 1H), 5.68 (d, *J* = 8.0 Hz, 1H), 5.43 (bs, 1H), 4.19 (d, *J* = 6.0 Hz, 1H), 3.86 (d, *J* = 18.6 Hz, 2H), 3.55 (s, 1H), 3.40 (t, *J* = 8.7 Hz, 1H). **^{13}C NMR** (151 MHz, DMSO) δ 162.97, 150.71, 141.16, 102.20, 88.21, 83.09, 72.79, 72.26, 7.68. **HRMS (DART) m/z**: [M-I]+ Calcd. for $C_9H_{11}N_2O_5^+$ 227.0662; found 227.06602; 0.79 ppm mass defect.

1-((2*R*,3*R*,4*S*)-3,4-dihydroxy-5-methylenetetrahydrofuran-2-yl)pyrimidine-2,4(1*H*,3*H*)-dione **(1k)**

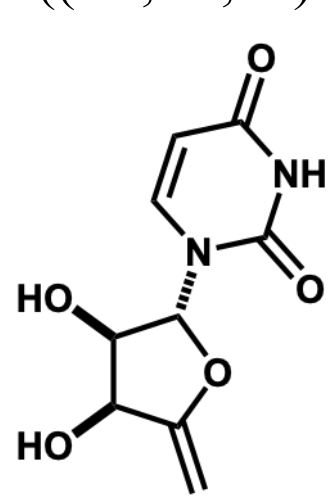

Prepared using a modified version of literature protocol. Sodium metal (35mg, 1.5eq., 1.5mmol) was added to anhydrous MeOH (2.6mL, 0.4M) at 0˚C. After the metal has been consumed, iodo uridine was added (354mg, 1eq., 1mmol) and the mixture was brought to reflux for 2h. The reaction was quenched to pH 8 with dry ice and concentrated. Purification on silica gel (0-100% NH_3 sat. MeOH/DCM) lent the product in 60% yield (136mg). Spectral data was consistent with literature reports. **^{1}H NMR** (500 MHz, DMSO-d_6) δ 9.28 (bs, 1H), 5.97 (d, *J* = 5.4 Hz, 1H), 5.66 (d, *J* = 8.1 Hz, 1H), 5.61 (bs, 1H), 5.47 (bs, 1H), 4.41 (d, *J* = 5.1 Hz, 1H), 4.35 (s, 1H), 4.26 (t, *J* = 5.3 Hz, 1H), 4.19 (d, *J* = 1.9 Hz, 1H). **^{13}C NMR** (126 MHz, DMSO) δ 162.97, 162.10, 150.66, 140.82, 102.40, 89.18, 84.98, 71.82, 69.30. **HRMS (DART) m/z**: [M+H]+ Calcd. for $C_9H_{11}N_2O_5^+$ 227.0662; found 227.06543; 3.39 ppm mass defect.

2.1.6 Preparation of 2,5-Anhydro-1-deoxy-D-gluco-hept-1-enitol (1l)[9]

2,3,5,6-Tetra-*O*-(*tert*-butyldimethysilyl)-D-glucono-1,4-lactone **(S26)**

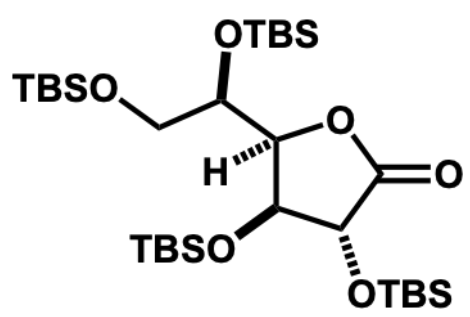

Prepared according to literature protocol. D-gluconolactone (250mg, 1eq., 1.4mmol) is dissolved in DCM (10mL, 0.14M) and cooled to 0˚C. 2,6-lutidine is added (1.30mL, 8eq., 11.2mmol), followed by TBSOTf (1.93mL, 6 eq., 8.43mmol), and the reaction is stirred at 0˚C for 2h. Warm to RT, quench with DI H_2O, wash with 5% aq. $CuSO_4$, followed by water, dry, concentrate. Purification on silica gel (0-2% Et_2O/Hexanes) lent the product in 78% yield (690mg). Spectral data was consistent with literature reports. **^{1}H NMR** (600 MHz, Chloroform-*d*) δ 4.60 – 4.54 (m, 2H), 4.43 (t, *J* = 6.8 Hz, 1H), 4.03 (td, *J* = 6.2, 3.0 Hz, 1H), 3.92 (dd, *J* = 10.3, 6.8 Hz, 1H), 3.64 (dd, *J* = 10.4, 5.6 Hz, 1H), 0.92 (s, 9H), 0.91 (s, 9H), 0.89 (s, 9H), 0.88 (s, 9H), 0.19 (s, 3H), 0.14 (s, 3H), 0.12 (s, 6H), 0.10 (s, 3H), 0.06 (s, 3H), 0.05 (s, 6H). **^{13}C NMR** (151 MHz, $CDCl_3$) δ 174.20, 79.01, 76.46, 74.55, 74.06, 64.05, 26.02, 25.96, 25.88, 18.36, 18.26, 18.13, 18.03, -4.24, -4.29, -4.31, -4.37, -4.39, -4.65, -5.14, -5.18. **HRMS (DART) m/z**: [M+H]+ Calcd. for $C_{30}H_{67}O_6Si_4^+$ 635.4009; found 635.39886; 3.21 ppm mass defect.

2,5-Anhydro-3,4,6,7-tetra-*O*-(*tert*-butyldimethylsilyl)-1-deoxy-D-gluco-hept-1-enitol **(1m)**

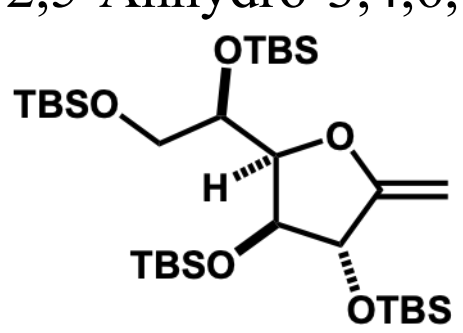

Prepared according to literature protocol. 2,3,5,6-Tetra-*O*-(*tert*-butyldimethysilyl)-D-glucono-1,4-lactone (526mg, 1eq., 0.83mmol) is dissolved in toluene (16.6mL, 0.05M). To that, a solution of Cp_2TiMe_2 (1.94mL, 2.7eq., 2.24mmol, 1.15M in toluene) is added and the reaction is stirred at 70˚C for 20h. After cooling, the mixture was concentrated. Purification on silica gel (100% hexanes, silica washed with 1%TEA/Hex) lent the product in 96% yield (503mg). Spectral data was consistent with literature reports. **^{1}H NMR** (500 MHz, Chloroform-*d*) δ 4.34 – 4.29 (m, 2H), 4.23 (d, *J* = 2.0 Hz, 1H), 4.07 – 4.02 (m, 2H), 3.98 (d, *J* = 1.7 Hz, 1H), 3.87 (dd, *J* = 11.1, 2.6 Hz, 1H), 3.73 (dd, *J* = 10.9, 5.9 Hz, 1H), 0.89 (s, 9H), 0.88 (s, 9H), 0.88 (s, 9H), 0.87 (s, 9H), 0.12 (s, 3H), 0.11 (s, 3H), 0.10 (s, 3H), 0.10 (s, 3H), 0.09 (s, 6H), 0.06 (s, 6H). **^{13}C NMR** (126 MHz, $CDCl_3$) δ 163.30, 89.85, 83.86, 83.83, 77.25, 71.99, 65.15, 26.13, 26.11, 26.00, 25.75, 18.57, 18.45, 18.18, 18.02, -3.43, -3.97, -4.08, -4.16, -4.38, -4.41, -5.21, -5.22. **HRMS (DART) m/z**: [M+H]+ Calcd. for $C_{31}H_{69}O_5Si_4^+$ 633.4217; found 633.42023; 2.32 ppm mass defect.

2,5-Anhydro-1-deoxy-D-gluco-hept-1-enitol **(1l)**

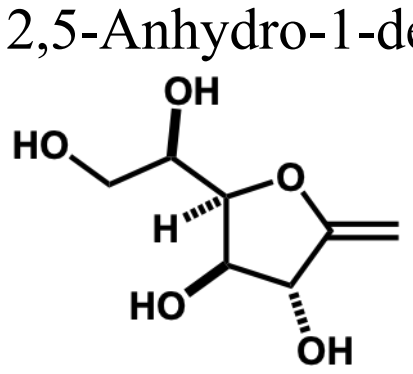

Prepared according to literature protocol. 2,5-Anhydro-3,4,6,7-tetra-*O*-(*tert*-butyldimethylsilyl)-1-deoxy-D-gluco-hept-1-enitol (380mg, 1eq., 0.6mmol) is dissolved in THF (6mL, 0.1M). To that, TBAF (2.4mL, 4eq., 0.6mmol, 1M in THF) is added and the reaction is stirred for 2h and concentrated. Purification on silica gel (8-10% MeOH/EtOAc, silica washed with 1%TEA/EtOAc) lent the product in 85% yield (90mg). Spectral data was consistent with literature reports. **^{1}H NMR** (600 MHz, Methanol-*d*$_4$) δ 4.32 (s, 1H), 4.26 (s, 1H), 4.22 (dd, *J* = 8.6, 3.1 Hz, 1H), 4.15 – 4.13 (m, 2H), 3.92 (ddd, *J* = 8.8, 5.9, 3.0 Hz, 1H), 3.79 (dd, *J* = 11.6, 3.1 Hz, 1H), 3.64 (dd, *J* = 11.6, 5.9 Hz, 1H). **^{13}C NMR** (151 MHz, MeOD) δ 165.13, 84.65, 83.45, 78.13, 76.50, 70.53, 65.08. **HRMS (DART) m/z**: [M+H]+ Calcd. for $C_7H_{13}O_5^+$ 177.0752; found 177.07555; 1.98 ppm mass defect.

2.2. Preparation of vinyl vicinal tricarbonyl hydrates

Known vinyl, vicinal tricarbonyl hydrate esters **2a•H₂O** and **2b•H₂O** were prepared according to literature procedures.[10–12]

tert-butyl 2,2-dihydroxy-3-oxopent-4-enoate **(2a•H₂O)**[11,12]

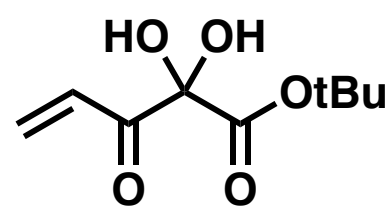

2a·H₂O

Spectral data was consistent with literature reports. **¹H NMR** (500 MHz, $CDCl_3$) δ 6.62 – 6.47 (m, 2H), 6.00 (dd, *J* = 8.4, 3.4 Hz, 1H), 4.99 (s, 2H), 1.46 (s, 9H). **¹³C NMR** (126 MHz, $CDCl_3$) δ 191.65, 168.24, 133.23, 129.05, 91.93, 85.14, 27.62.

methyl 2,2-dihydroxy-3-oxopent-4-enoate **(2b•H₂O)**[10,12]

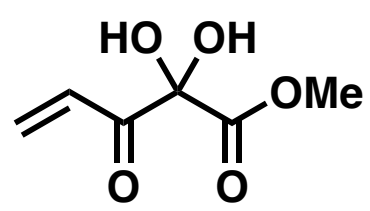

2b·H₂O

Spectral data was consistent with literature reports. **¹H NMR** (500 MHz, $CDCl_3$) δ 6.58 (d, *J* = 6.2 Hz, 2H), 5.99 (dd, *J* = 6.7, 5.2 Hz, 1H), 5.20 (s, 2H), 3.80 (s, 3H). **¹³C NMR** (126 MHz, $CDCl_3$) δ 188.91, 168.41, 128.28, 83.34, 54.39, 22.99.

2.2.1 Preparation of methylated vinyl vicinal tricarbonyl hydrates

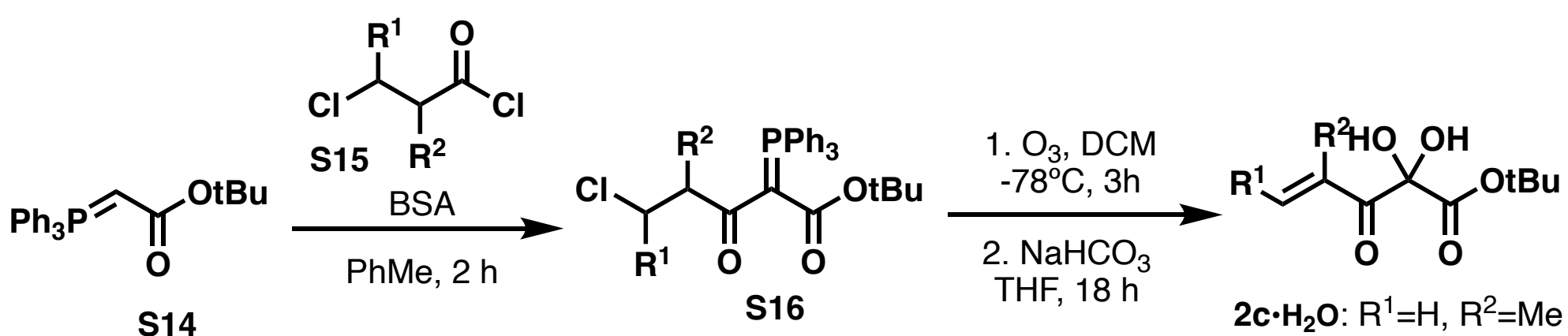

tert-butyl 4-methyl-2,3-dioxopent-4-enoate **(2c•H₂O)**

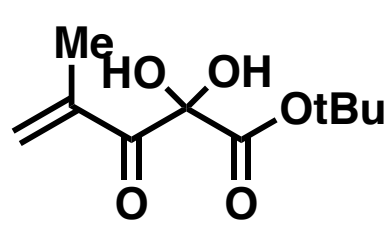

2c·H₂O

3-chloro-2-methylpropanoyl chloride (**S15c**) (1.0 equiv, 21.25 mmol) is added dropwise to a solution of tert-butyl 2-(triphenyl-l5-phosphaneylidene)acetate (**S14c**) (1.0 equiv, 21.25 mmol) and BSA (1.2 equiv, 25.50 mmol) in toluene (304 mL) at 10°C over 5 minutes, then was stirred at room temperature for 2h. Then, 100 mL of ether was added, and the resulting salt precipitate was removed by filtration and washed with ether. The combined filtrates were concentrated under reduced pressure, giving crude ß-chloroacyl ylide (**S16c**). Without further purification, **S16c** (15.59 mmol) was dissolved in DCM (31 mL, 0.5M) and cooled to -78°C. Ozone was then bubbled through the mixture until a green/blue color persisted (1.5 h). The excess ozone was excluded by argon gas. The solution was allowed to warm to 23ºC then concentrated. Half the residue (7.41 mmol) was dissolved in THF (46 mL) and treated with saturated aqueous $NaHCO_3$ (2.0 equiv, 3.0 M, 4.9 mL), and stirred at 25 °C until completion of the dehydrochlorination reaction. After diluting the reaction mixture with water, the aqueous solution was extracted 2x with EtOAc. The combined extracts were dried and concentrated in vacuo to yield the vinyl tricarbonyl as a yellow oil. Purification by silica gel flash chromatography (30% EtOAc in hexanes) yielded **2c•H₂O** as a pale yellow solid in 27% yield (3 steps) containing a a 3:1 ratio of **2c•H₂O** : **2c**. **¹H NMR** (600 MHz, $CDCl_3$) δ 6.25 (q, *J* = 1.3 Hz, 1H), 6.01 (q, *J* = 1.5 Hz, 1H), 5.14 (s, 2H), 1.99 (dd, *J* = 1.5, 0.9 Hz, 3H), 1.47 (s, 9H). **¹³C NMR** (151 MHz, $CDCl_3$) δ 193.38, 168.88, 139.14, 130.08, 91.38, 84.69, 27.58, 18.40. **HRMS (ESI-TOF) m/z:** [M+H]+ Calcd. for $C_{10}H_{15}O_4$ 199.0965; found 199.0967

tert-butyl (E)-2,2-dihydroxy-3-oxohex-4-enoate **(2d•H_2O)**

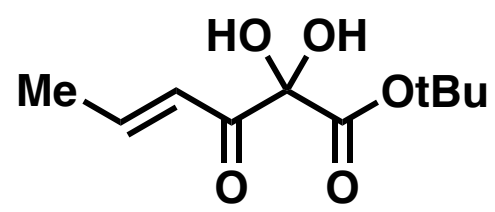

2d·H_2O

3-chlorobutanoyl chloride (**S15d**) (1.0 equiv, 34.53 mmol) is added dropwise to a solution of tert-butyl 2-(triphenyl-l5-phosphaneylidene)acetate (**S14d**) (1.0 equiv, 34.53 mmol) and BSA (1.2 equiv, 41.44 mmol) in toluene (493 mL) at 10°C over 5 minutes, then was stirred at room temperature for 2h. Then, 200 mL of ether was added, and the resulting salt precipitate was removed by filtration and washed with ether. The combined filtrates were concentrated under reduced pressure, giving crude ß-chloroacyl ylide (**S16d**). Without further purification, **S16d** (28.0 mmol) was dissolved in DCM (280 mL, 1.0M) and cooled to -78°C. Ozone was then bubbled through the mixture until a green/blue color persisted (1h). The excess ozone was excluded by argon gas. The solution was allowed to warm to 23ºC then concentrated. The residue was dissolved in THF (90 mL) and treated with saturated aqueous $NaHCO_3$ (1.0 equiv, 1.2 M, 11 mL), and stirred at 25 °C until completion of the dehydrochlorination reaction. After diluting the reaction mixture with water, the aqueous solution was extracted 2x with EtOAc. The combined extracts were dried and concentrated in vacuo to yield the vinyl tricarbonyl as a yellow oil. Purification by silica gel flash chromatography (30% EtOAc in hexanes) yielded **2d•H_2O** as a yellow solid in 23% yield (3 steps) containing a 5:1 ratio of **2d•H_2O : 2d**. **^{1}H NMR** (500 MHz, $CDCl_3$) δ 7.22 (m, J = 15.7, 7.0 Hz, 1H), 6.30 (d, J = 15.7, 1H), 5.06 (br s, 2H), 1.96 (d, J = 7.0 Hz, 3H), 1.45 (s, 9H). **^{13}C NMR** (126 MHz, $CDCl_3$) δ 191.02, 168.63, 148.90, 124.18, 91.89, 84.84, 27.63, 18.91. **HRMS (ESI-TOF) m/z:** [M+H]+ Calcd. for $C_{10}H_{15}O_4$ 199.0965; found 199.0962

2.3. Preparation of spiroketals and ene adducts

General Procedure 6:

Vicinal tricarbonyl hydrates **2a-d•H_2O** were dissolved in DCM (0.1-0.3M) over activated 3Å sieves and left to desiccate at room temperature for 3-12 h. The solution gradually turned yellow, yielding **2a-d** as solutions in DCM. **tricarbonyls can polymerize or decompose if concentration exceeds 0.3M.* To the solution of desiccated tricarbonyl **2** (1.0-2.0 equiv) was added enol ether **1** at room temperature (enol ethers that were thick oils were dissolved in a minimal amount of DCM for transfer). For enol ether substrates isolated in <100% purity, the quantity of substrate employed in each cycloaddition was corrected for purity; reported millimoles and all derived yields reflect the purity-corrected quantity of enol ether rather than the total mass of isolated material. Tricarbonyl component **2** was employed as the limiting reagent (1.0 equiv) relative to enol ether **1** (1.0–1.2 equiv) under standard conditions. In cases where **1** contained residual DBU or titanocene byproducts from its synthesis, these impurities were found to promote decomposition of **2** under the reaction conditions. Under these circumstances, **1** was instead used as the limiting reagent (1.0 equiv), with **2** employed in excess (1.2–1.7 equiv) to compensate for competing decomposition and drive the reaction to completion. The mixture was stirred for 3h and concentrated under reduced pressure. The crude residue obtained was analyzed by ^{1}H NMR spectroscopy to calculate d.r, then was purified by flash column chromatography using EtOAc/Hex to give pure spiroketal **3** and/or ene adduct **5**. **Silica gel was slurried with 2% Et_3N in hexane prior to use.*

tert-butyl (R)-2-oxo-2-(1,6-dioxaspiro[4.5]dec-7-en-7-yl)acetate **(3a)**

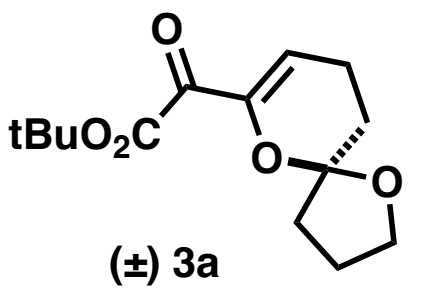

(±) 3a

Prepared according to **General Procedure 6** using tert-butyl 2,3-dioxopent-4-enoate (**2a•H$_2$O**) (0.380 mmol) and 2-methylenetetrahydrofuran[13] (0.418 mmol, 1.1 equiv.) in DCM (1.9 mL). Purified and isolated using 10% EtOAc in hexanes. 67% yield (0.250 mmol), white solid. **^{1}H NMR** (500 MHz, $CDCl_3$) δ 6.16 (ddd, *J* = 5.3, 3.1, 0.7 Hz, 1H), 4.05 (td, *J* = 8.1, 6.1 Hz, 1H), 3.97 (td, *J* = 8.2, 5.7 Hz, 1H), 2.51 (dddd, *J* = 19.6, 10.7, 7.8, 3.2 Hz, 1H), 2.36 – 2.14 (m, 3H), 2.04 – 1.95 (m, 1H), 1.95 – 1.87 (m, 2H), 1.87 – 1.76 (m, 1H), 1.55 (s, 9H). **^{13}C NMR (126 MHz, $CDCl_3$)** δ 182.67, 163.63, 146.48, 118.55, 106.90, 84.37, 68.36, 36.63, 28.79, 27.98, 23.83, 19.81. **HRMS (ESI-TOF) m/z**: [M+H]+ Calcd. for $C_{14}H_{21}O_5$ 269.1384; found 269.1386

tert-butyl 2-oxo-2-(-2-phenethyl-1,6-dioxaspiro[4.5]dec-7-en-7-yl)acetate **(3b)**

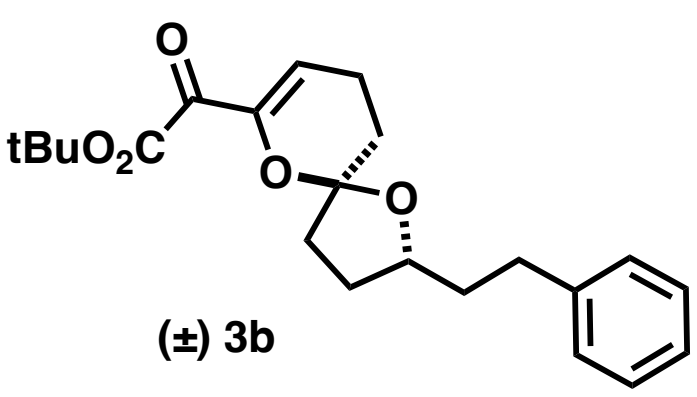

(±) 3b

Prepared according to **General Procedure 6** using tert-butyl 2,3-dioxopent-4-enoate (**2a•H$_2$O**) (0.217 mmol) and 2-methylene-5-phenethyl tetrahydrofuran (**1b**) (0.261 mmol, 1.2 equiv.) in DCM (1.1 mL). Purified and isolated using 10% EtOAc in hexanes. 81% yield (0.217 mmol), 6.5:1 d.r, yellow solid. **^{1}H NMR (600 MHz, $CDCl_3$)** δ 7.29 – 7.22 (m, 2H), 7.17 (m, *J* = 5.6, 2.7 Hz, 3H), 6.19 – 6.14 (m, 1H), 4.29 – 4.21 (m, 1H), 2.74 – 2.60 (m, 2H), 2.53 (dddd, *J* = 19.4, 9.5, 8.3, 3.2 Hz, 1H), 2.33 – 2.28 (m, 1H), 2.28 – 2.19 (m, 2H), 1.96 – 1.85 (m, 4H), 1.78 (dddd, *J* = 13.5, 9.8, 6.6, 5.8 Hz, 1H), 1.67 – 1.59 (m, 1H), 1.56 (s, 9H). **^{13}C NMR (151 MHz, $CDCl_3$)** δ 182.75, 163.73, 146.56, 141.83, 128.41, 128.36, 125.82, 118.82, 106.84, 84.36, 78.86, 37.15, 36.54, 32.19, 29.52, 29.38, 28.00, 19.85. **HRMS (ESI-TOF) m/z**: [M+H]+ Calcd. for $C_{22}H_{29}O_5$ 373.2010; found 373.2012

tert-butyl 2-oxo-2-(2-phenyl-1,6-dioxaspiro[4.5]dec-7-en-7-yl)acetate **(3c)**

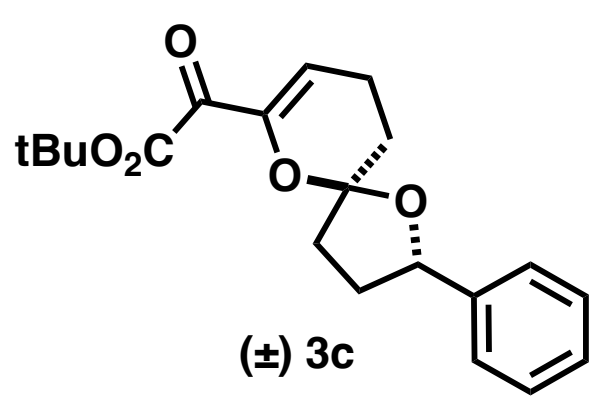

(±) 3c

Prepared according to **General Procedure 6** using tert-butyl 2,3-dioxopent-4-enoate (**2a•H$_2$O**) (1 equiv, 4.95 mmol) and 2-methylene-5-phenyl tetrahydrofuran (**1c**) (1.2 equiv, 5.44 mmol) in DCM (16.5 mL). Purified and isolated using 10% EtOAc in hexanes. 73% yield (1.2 g, 3.6 mmol), >20:1 d.r, white crystalline solid. **^{1}H NMR** (500 MHz, $CDCl_3$) δ 7.32 (ddd, *J* = 10.5, 5.8, 3.9 Hz, 5H), 6.19 (dd, *J* = 5.5, 2.9 Hz, 1H), 5.26 (t, *J* = 7.2 Hz, 1H), 2.69 – 2.54 (m, 3H), 1.57 (s, 9H). **^{13}C NMR** (126 MHz, $CDCl_3$) δ 182.66, 163.60, 146.50, 142.03, 128.40, 127.54, 125.73, 119.34, 107.18, 84.43, 80.78, 36.82, 33.16, 29.23, 28.01, 19.79. **HRMS (ESI-TOF) m/z**: [M+H]+ Calcd. for $C_{20}H_{25}O_5$ 345.1697; found 345.1738

tert-butyl 2-(-2-(4-chlorophenyl)-1,6-dioxaspiro[4.5]dec-7-en-7-yl)-2-oxoacetate **(3d)**

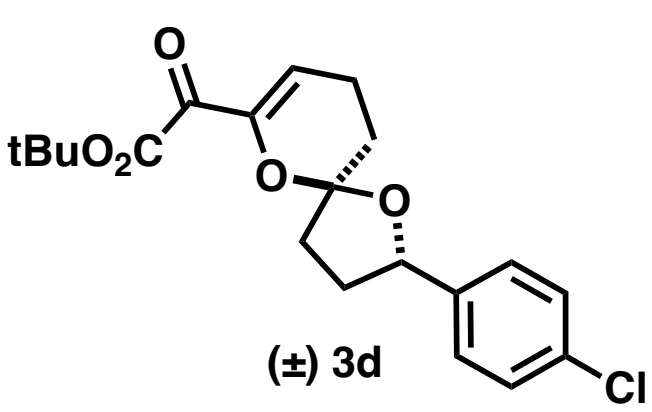

(±) 3d

Prepared according to **General Procedure 6** using tert-butyl 2,3-dioxopent-4-enoate (**2a•H$_2$O**) (1.2 equiv, 0.742 mmol) and 2-(4-chlorophenyl)-5-methylenetetrahydrofuran (**1d**) (1.0 equiv, 0.618 mmol) in DCM (3.7 mL). Purified and isolated using 10% EtOAc in hexanes. 78% yield (0.483 mmol), >20:1 d.r, white solid. **^{1}H NMR** (500 MHz, $CDCl_3$) δ 7.31 – 7.27 (m, 2H), 7.23 (m, 2H), 6.19 (dd, *J* = 4.5, 3.0 Hz, 1H), 5.22 (t, *J* = 7.2 Hz, 1H), 2.72 – 2.49 (m, 2H), 2.42 – 2.30 (m, 2H), 2.13 – 1.95 (m, 3H), 1.84 (dddd, *J* = 12.5, 10.1, 7.1, 5.5 Hz, 1H), 1.56 (s, 9H). **^{13}C NMR** (126 MHz, $CDCl_3$) δ 182.67, 163.65, 146.55, 140.69, 133.31, 128.66, 127.22,

119.47, 107.25, 84.61, 80.16, 36.85, 33.24, 29.28, 28.12, 19.86. **HRMS (ESI-TOF) m/z**: [M+H]+ Calcd. for $C_{20}H_{24}ClO_5$ 379.1307; found 379.1306

tert-butyl 2-(2-(4-fluorophenyl)-1,6-dioxaspiro[4.5]dec-7-en-7-yl)-2-oxoacetate **(3e)**

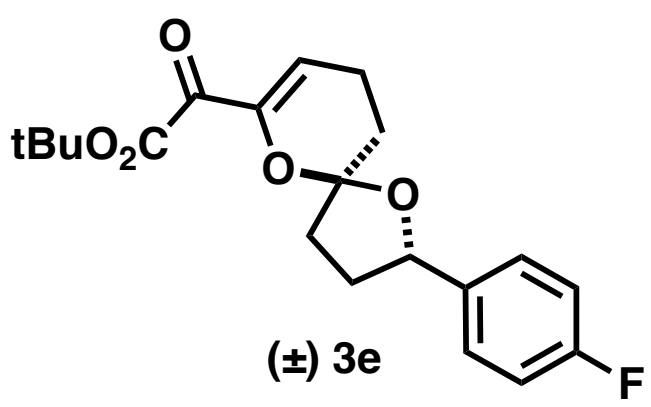

(±) 3e

Prepared according to **General Procedure 6** using tert-butyl 2,3-dioxopent-4-enoate (**2a•H₂O**) (1.2 equiv, 1.07 mmol) and 2-(4-fluorophenyl)-5-methylene tetrahydrofuran (**1e**) (1.0 equiv, 0.890 mmol) in DCM (5.3 mL). Purified and isolated using 10% EtOAc in hexanes. 80% yield (0.715 mmol), >20:1 d.r, white solid. ^{1}**H NMR** (500 MHz, $CDCl_3$) δ 7.32 – 7.25 (m, 2H), 7.05 – 6.97 (m, 2H), 6.19 (dd, *J* = 4.6, 3.1 Hz, 1H), 5.23 (t, *J* = 7.2 Hz, 1H), 2.66 – 2.53 (m, 2H), 2.40 – 2.32 (m, 2H), 2.10 – 2.03 (m, 2H), 2.03 – 1.96 (m, 1H), 1.85 (dd, *J* = 12.8, 10.2, 7.2, 5.7 Hz, 1H), 1.56 (s, 9H). ^{13}**C NMR** (126 MHz, $CDCl_3$) δ 182.57, 163.54, 146.45, 137.68, 127.47, 119.37, 115.32, 115.15, 107.07, 84.47, 80.16, 36.83, 33.22, 29.21, 28.00, 19.74. **HRMS (ESI-TOF) m/z**: [M+H]+ Calcd. for $C_{20}H_{24}FO_5$ 363.1602; found 363.1618

tert-butyl 2-(2-(4-bromophenyl)-1,6-dioxaspiro[4.5]dec-7-en-7-yl)-2-oxoacetate **(3f)**

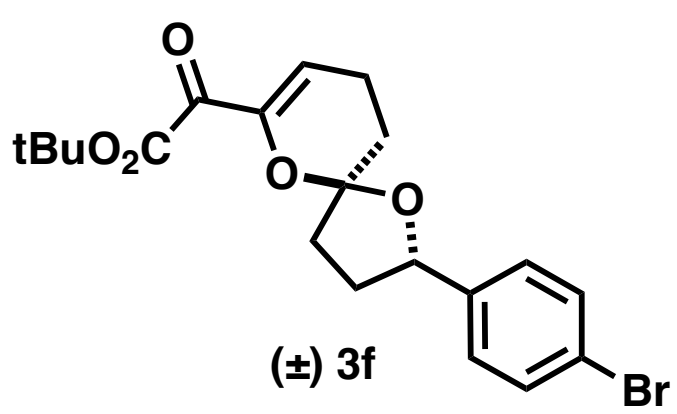

(±) 3f

Prepared according to **General Procedure 6** using tert-butyl 2,3-dioxopent-4-enoate (**2a•H₂O**) (1.1 equiv, 0.828 mmol) and 2-(4-bromophenyl)-5-methylene tetrahydrofuran (**1f**) (1.0 equiv, 0.753 mmol) in DCM (4.14 mL). Purified and isolated using 10% EtOAc in hexanes. 75.6% yield (0.569 mmol), >20:1 d.r, white solid. ^{1}**H NMR** (500 MHz, $CDCl_3$) δ 7.50 – 7.41 (m, 2H), 7.22 – 7.12 (m, 2H), 6.20 (dd, *J* = 4.5, 3.1 Hz, 1H), 5.21 (t, *J* = 7.2 Hz, 1H), 2.67 – 2.53 (m, 2H), 2.40 – 2.28 (m, 2H), 2.12 – 1.95 (m, 3H), 1.84 (dddd, *J* = 12.4, 10.1, 7.0, 5.5 Hz, 1H), 1.56 (s, 9H). ^{13}**C NMR** (126 MHz, $CDCl_3$) δ 182.53, 163.52, 146.42, 141.12, 131.49, 127.44, 121.28, 119.33, 107.13, 84.49, 80.05, 36.71, 33.07, 29.14, 28.00, 19.74. **HRMS (ESI-TOF) m/z**: [M+H]+ Calcd. for $C_{20}H_{24}BrO_5$ 423.0802; found 423.0805

tert-butyl 2-(2-(4-methoxyphenyl)-1,6-dioxaspiro[4.5]dec-7-en-7-yl)-2-oxoacetate **(3g)**

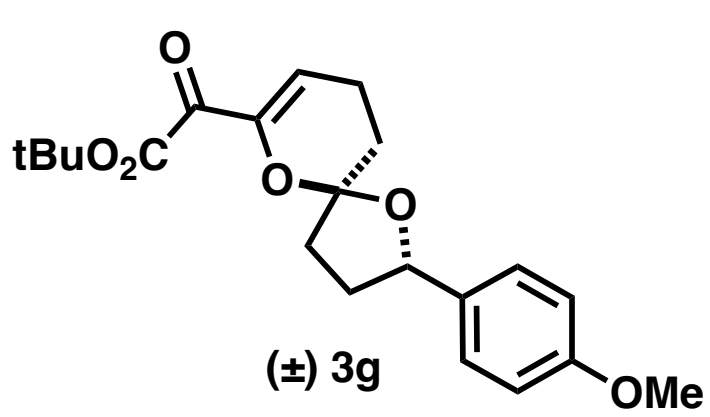

(±) 3g

Prepared according to **General Procedure 6** using tert-butyl 2,3-dioxopent-4-enoate (**2a•H₂O**) (1.7 equiv, 1.05 mmol) and 2-(4-methoxyphenyl)-5-methylene tetrahydrofuran (**1f**) (1.0 equiv, 0.618 mmol) in DCM (3.7 mL). Purified and isolated using 10% EtOAc in hexanes. 71.3% yield (0.441 mmol), >20:1 d.r, white solid. ^{1}**H NMR** (500 MHz, $CDCl_3$) δ 7.25 – 7.19 (m, 2H), 6.90 – 6.82 (m, 2H), 6.18 (dd, *J* = 4.5, 3.1 Hz, 1H), 5.21 (t, *J* = 7.2 Hz, 1H), 3.79 (s, 3H), 2.66 – 2.49 (m, 2H), 2.42 – 2.26 (m, 2H), 2.12 – 2.03 (m, 2H), 1.99 (ddd, *J* = 13.4, 11.7, 6.2 Hz, 1H), 1.88 (dddd, *J* = 12.4, 10.1, 7.3, 5.8 Hz, 1H), 1.56 (s, 9H). ^{13}**C NMR** (126 MHz, $CDCl_3$) δ 182.68, 163.62, 159.11, 146.51, 133.97, 127.17, 119.35, 113.80, 107.04, 84.41, 80.59, 55.31, 36.98, 33.10, 29.29, 28.00, 19.77. **HRMS (ESI-TOF) m/z**: [M+H]+ Calcd. for $C_{21}H_{27}O_6$ 375.1802; found 375.1817

tert-butyl 2-(2-butyl-1,6-dioxaspiro[4.5]dec-7-en-7-yl)-2-oxoacetate **(3i)**

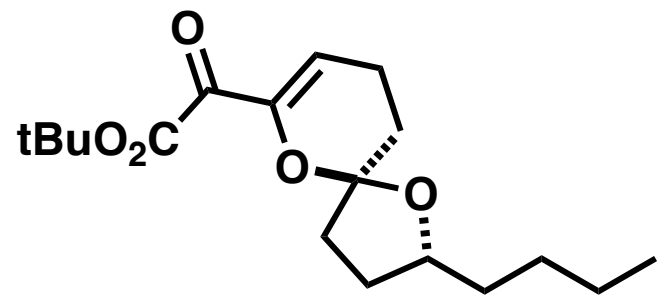

(±) 3i

Prepared according to **General Procedure 6** using tert-butyl 2,3-dioxopent-4-enoate (**2a•H_2O**) (1.0 equiv, 0.502 mmol) and 2-butyl-5-methylenetetrahydrofuran (**1i**) (1.2 equiv, 0.602 mmol) in DCM (2.5 mL). Purified and isolated using 10% EtOAc in hexanes. 89% yield (0.450 mmol), 6:1 d.r, light yellow solid. **^{1}H NMR** (500 MHz, $CDCl_3$) δ 6.14 (dd, *J* = 5.4, 3.2 Hz, 1H), 4.23 (p, *J* = 6.6 Hz, 1H), 2.52 (dddd, *J* = 19.5, 9.5, 8.1, 3.2 Hz, 1H), 2.36 – 2.14 (m, 3H), 1.95 – 1.81 (m, 3H), 1.56 (m, 9H), 1.56 (s, 9H), 1.49 – 1.39 (m, 2H), 1.37 – 1.21 (m, 4H), 0.89 (t, *J* = 7.0 Hz, 3H). **^{13}C NMR** (126 MHz, $CDCl_3$) δ 182.80, 163.74, 146.55, 118.60, 106.84, 84.31, 79.78, 36.52, 35.20, 29.43, 29.37, 27.99, 27.89, 22.74, 19.83, 14.05. **HRMS (ESI-TOF) m/z**: [M+H]+ Calcd. for $C_{18}H_{29}O_5$, 325.2010; found 325.2008

tert-butyl 2-(3',4'-dihydro-3*H*-spiro[isobenzofuran-1,2'-pyran]-6'-yl)-2-oxoacetate **(3j)**

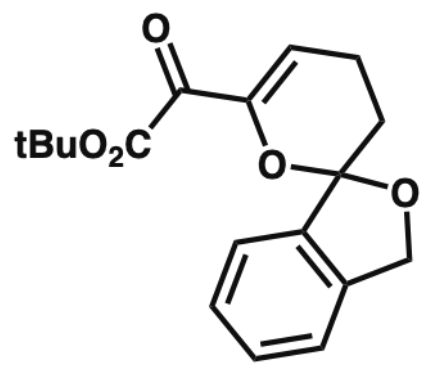

Prepared according to **General Procedure 6** using tert-butyl 2,3-dioxopent-4-enoate (**2a•H_2O**) (22mg, 1.1eq., 0.11mmol) and 1-methylene-1,3-dihydroisobenzofuran (13mg, 1eq., 0.1 mmol) in DCM (1mL, 0.1M). Purification on silica gel (0-10% EtOAc/Hex) lent the product as white crystals in 50% yield (16mg). **^{1}H NMR** (500 MHz, Chloroform-*d*) δ 7.40 (ddd, *J* = 7.8, 5.8, 2.9 Hz, 1H), 7.37 – 7.32 (m, 2H), 7.29 (d, *J* = 7.5 Hz, 1H), 6.28 (ddd, *J* = 6.0, 2.9, 1.2 Hz, 1H), 5.27 (d, *J* = 12.6 Hz, 1H), 5.05 (d, *J* = 12.6 Hz, 1H), 2.73 (dddd, *J* = 19.4, 12.7, 6.6, 2.8 Hz, 1H), 2.48 (dtd, *J* = 19.4, 6.0, 1.6 Hz, 1H), 2.31 (td, *J* = 13.0, 6.2 Hz, 1H), 2.10 (ddt, *J* = 13.3, 6.7, 1.5 Hz, 1H), 1.49 (s, 9H). **^{13}C NMR** (126 MHz, $CDCl_3$) δ 182.48, 163.66, 147.16, 140.17, 139.17, 129.77, 128.01, 122.37, 121.29, 119.17, 108.24, 84.63, 72.19, 29.10, 28.02, 19.14. **HRMS (DART) m/z**: [M+H]+ Calcd. for $C_{18}H_{21}O_5^+$ 317.1384; found 317.13733; 3.37 ppm mass defect.

tert-butyl 2-((2*R*,3*R*,4*S*,5*R*)-2-(2,4-dioxo-3,4-dihydropyrimidin-1(2*H*)-yl)-3,4-dihydroxy-1,6 dioxaspiro[4.5]dec-7-en-7-yl)-2-oxoacetate **((–)-3k)**

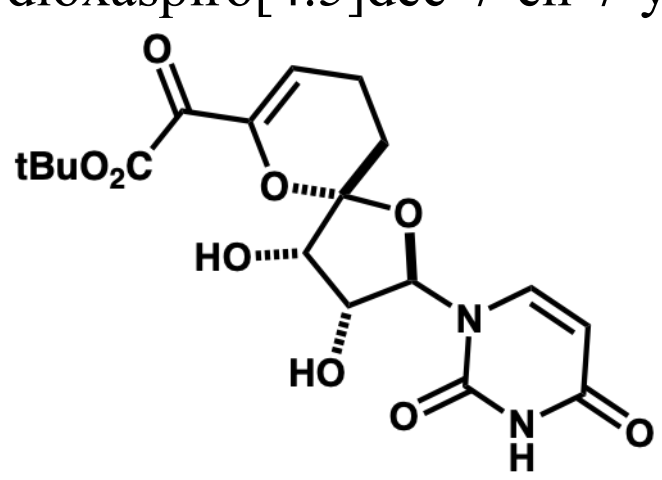

Prepared according to **General Procedure 6** using tert-butyl 2,3-dioxopent-4-enoate (**2a•H_2O**) (40mg, 2eq., 0.2mmol) and uridine methylidene tetrahydrofuran (23mg, 1eq., 0.1 mmol) in THF (1mL, 0.1M) and was stirred for 24h. Purification on silica gel (0-5% IPA/EtOAc) lent the product as an off-white powder in 53% yield (>20:1 d.r) (22mg). **^{1}H NMR** (500 MHz, DMSO-*d_6*) δ 11.40 (s, 1H), 7.68 (d, *J* = 7.5 Hz, 1H), 6.23 – 6.10 (m, 1H), 5.84 (d, *J* = 3.4 Hz, 1H), 5.63 (d, *J* = 8.0 Hz, 1H), 5.34 (d, *J* = 5.5 Hz, 1H), 4.81 (d, *J* = 7.8 Hz, 1H), 4.28 – 4.13 (m, 2H), 2.42 – 2.24 (m, 2H), 1.96 – 1.84 (m, 2H), 1.50 (s, 9H). **^{13}C NMR** (126 MHz, DMSO) δ 182.03, 163.32, 162.99, 150.34, 145.41, 142.20, 120.61, 104.32, 102.41, 91.05, 84.87, 73.10, 71.38, 27.53, 26.61, 18.40. **HRMS (DART) m/z**: [M-H]- Calcd. for $C_{18}H_{21}N_2O_9^-$ 409.1253; found 409.12354; 4.30 ppm mass defect. $[\alpha]^{20}_D$ = -14.00 (c=0.1, THF).

**spirocyclic relative stereochemistry was confirmed using ^{1}H-^{1}H COSY and ^{1}H-^{1}H NOESY correlations where the following key interaction was observed.*

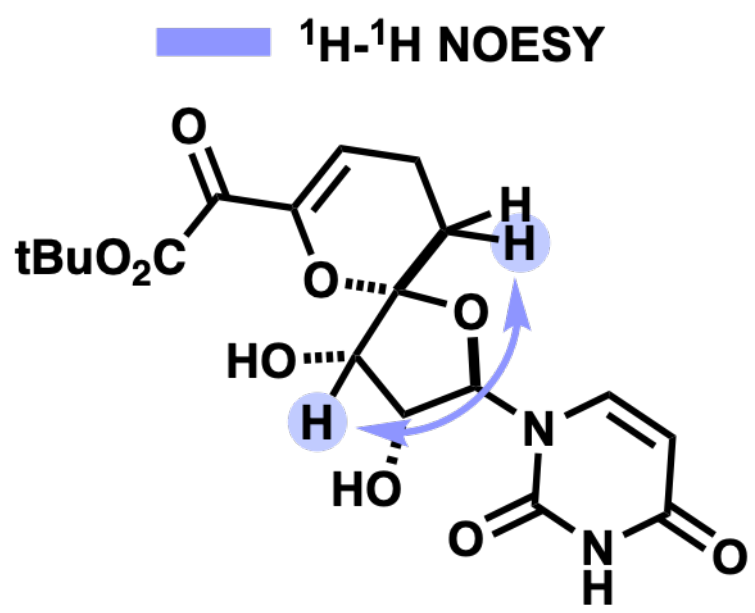

tert-butyl 2-((2*R*,3*R*,4*R*,5*R*)-2-((*R*)-1,2-dihydroxyethyl)-3,4-dihydroxy-1,6-dioxaspiro[4.5]dec-7-en-7-yl)-2-oxoacetate **((+)3l)**

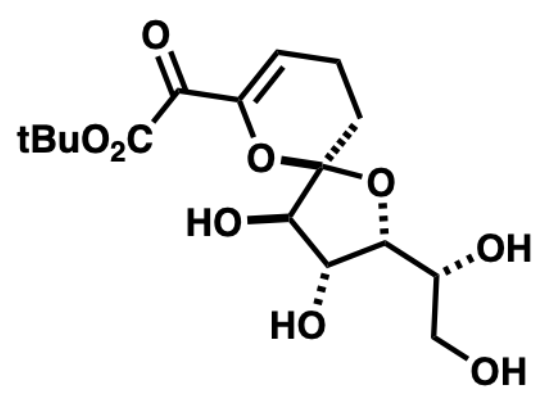

Prepared according to **General Procedure 6** using tert-butyl 2,3-dioxopent-4-enoate (**2a•H$_2$O**) (40mg, 2eq., 0.2mmol) and 2,5-Anhydro-1-deoxy-D-gluco-hept-1-enitol (18mg, 1eq., 0.1 mmol) in THF (1mL, 0.1M) and was stirred for 24h. Purification on silica gel (0-7% IPA/EtOAc) lent the product as a brown glass in 60% yield (>20:1 d.r) (22mg). **^{1}H NMR** (500 MHz, DMSO-*d*$_6$) δ 6.11 (t, *J* = 4.2 Hz, 1H), 5.33 (t, *J* = 6.2 Hz, 2H), 4.61 (d, *J* = 5.1 Hz, 1H), 4.49 (t, *J* = 5.7 Hz, 1H), 4.25 (q, *J* = 6.1 Hz, 1H), 3.98 (t, *J* = 6.4 Hz, 1H), 3.71 (t, *J* = 5.9 Hz, 1H), 3.67 (p, *J* = 5.2 Hz, 1H), 3.53 (dt, *J* = 10.2, 4.7 Hz, 1H), 3.37 – 3.34 (m, 1H), 2.37 – 2.28 (m, 2H), 1.85 (dt, *J* = 13.3, 8.1 Hz, 1H), 1.76 (dt, *J* = 13.6, 5.0 Hz, 1H), 1.51 (s, 9H). **^{13}C NMR** (126 MHz, DMSO) δ 182.53, 163.62, 145.66, 119.43, 102.62, 84.63, 81.28, 77.81, 75.46, 70.89, 62.51, 27.55, 26.50, 18.53. **HRMS (DART) m/z**: [M-H]- Calcd. for $C_{16}H_{23}O_9^-$ 359.1348; found 359.13309; 4.76 ppm mass defect. $[\alpha]^{20}_D$ = +70.00 (c=0.1, THF).

**spirocyclic relative stereochemistry was confirmed using ^{1}H-^{1}H COSY and ^{1}H-^{1}H NOESY correlations where the following key interaction was observed.*

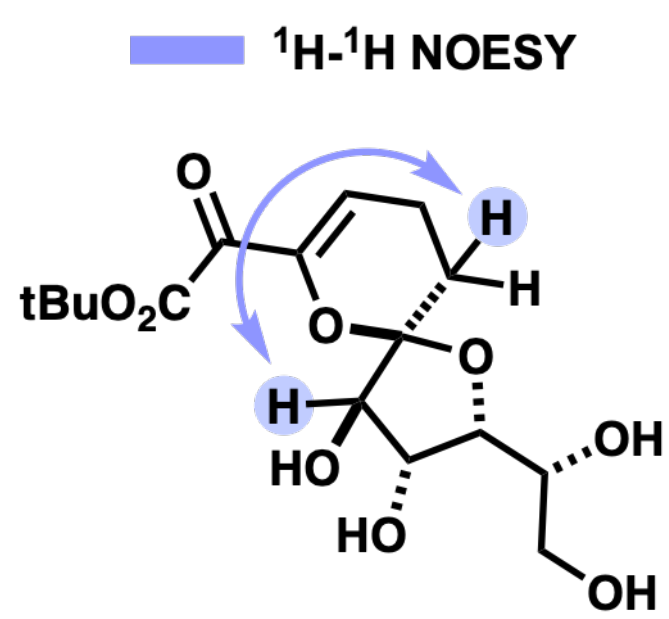

tert-butyl 2-((2*R*,3*S*,4*R*,5*R*)-3,4-bis((*tert*-butyldimethylsilyl)oxy)-2-((*R*)-2,2,3,3,8,8,9,9-octamethyl-4,7-dioxa-3,8-disiladecan-5-yl)-1,6-dioxaspiro[4.5]dec-7-en-7-yl)-2-oxoacetate **((+)-3m)**

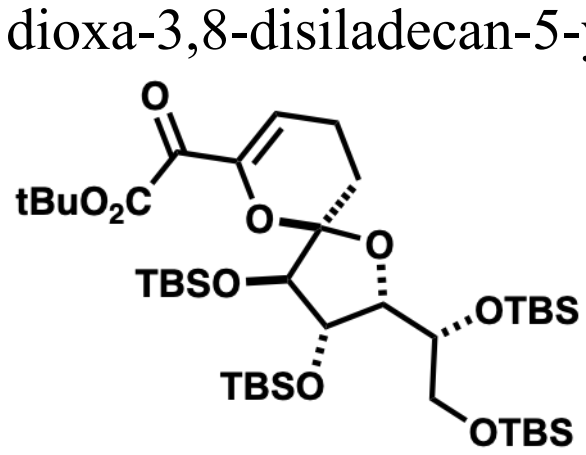

Prepared according to **General Procedure 6** using tert-butyl 2,3-dioxopent-4-enoate (**2a•H$_2$O**) (22mg, 1.1eq., 0.11mmol) and 2,5-Anhydro-3,4,6,7-tetra-*O*-(*tert*-butyldimethylsilyl)-1-deoxy-D-gluco-hept-1-enitol (63mg, 1eq., 0.1 mmol) in DCM (1mL, 0.1M). Purification on silica gel (0-3% EtOAc/Hex) lent the product as a clear oil in 62% yield (>20:1 d.r) (51mg). **^{1}H NMR** (500 MHz, Chloroform-*d*) δ 6.13 (ddd, *J* = 5.8, 2.9, 1.0 Hz, 1H), 4.31 – 4.25 (m, 2H), 3.95 (ddd, *J* = 7.0, 4.9, 2.4 Hz, 1H), 3.85 (d, *J* = 3.5 Hz, 1H), 3.82 (dd, *J* = 10.6, 2.4 Hz, 1H), 3.62 (dd, *J* = 10.6,

6.5 Hz, 1H), 2.48 (dddd, J = 18.9, 12.0, 6.4, 3.0 Hz, 1H), 2.21 (dtd, J = 19.1, 5.9, 2.3 Hz, 1H), 1.91 (dddd, J = 12.9, 6.4, 2.4, 1.2 Hz, 1H), 1.71 (td, J = 12.4, 5.9 Hz, 1H), 0.91 (s, 9H), 0.90 (s, 9H), 0.88 (s, 9H), 0.86 (s, 9H), 0.12 (s, 3H), 0.11 (s, 3H), 0.10 (s, 3H), 0.10 (s, 3H), 0.08 (s, 3H), 0.06 (s, 3H), 0.02 (s, 6H). **^{13}C NMR** (126 MHz, $CDCl_3$) δ 182.23, 163.73, 146.69, 118.92, 103.29, 84.21, 81.63, 78.03, 77.36, 72.99, 65.15, 28.50, 28.17, 26.16, 26.12, 26.08, 25.96, 18.88, 18.60, 18.44, 18.24, 18.12, -3.40, -3.84, -4.06, -4.33, -4.39, -4.56, -5.18, -5.39. **HRMS (DART) m/z**: [M+H]+ Calcd. for $C_{40}H_{81}O_9Si_4^+$ 817.4952; found 817.49243; 3.39 ppm mass defect. $[\alpha]^{20}_D$ = +28.00 (c=0.1, DCM).

**spirocyclic relative stereochemistry was defined via analogy with 31.*

tert-butyl 2-((2R,4R,5S)-2,4-diphenyl-1,3,6-trioxaspiro[4.5]dec-7-en-7-yl)-2-oxoacetate **((+)-3n)**

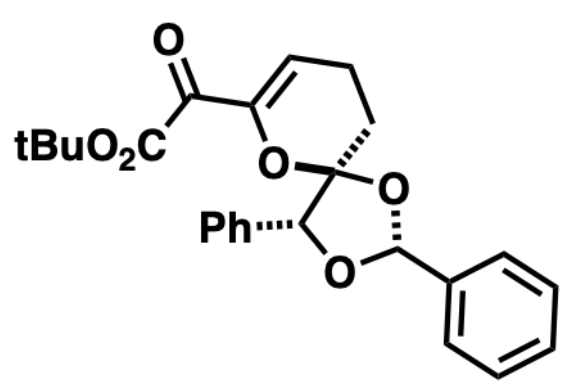

Prepared according to **General Procedure 6** using tert-butyl 2,3-dioxopent-4-enoate (**2a•H₂O**) (1.0 equiv, 0.420 mmol) and (2R,5R)-4-methylene-2,5-diphenyl-1,3-dioxolane (**1n**) (1.0 equiv, 0.420 mmol) in DCM (1.4 mL). Purified and isolated using 10% EtOAc in hexanes. 64% yield (0.269 mmol), >20:1 d.r, light yellow solid. **^{1}H NMR** (500 MHz, $CDCl_3$) δ 7.70 – 7.56 (m, 2H), 7.52 – 7.30 (m, 8H), 6.29 (s, 1H), 6.23 (dd, J = 4.7, 2.8 Hz, 1H), 5.41 (s, 1H), 2.46 (dddd, J = 19.5, 12.8, 6.6, 2.8 Hz, 1H), 2.21 (dt, J = 19.5, 5.3 Hz, 1H), 1.59 (s, 9H), 1.48 – 1.43 (m, 1H), 1.29 (dd, J = 13.3, 6.3 Hz, 1H). **^{13}C NMR** (126 MHz, $CDCl_3$) δ 181.98, 163.27, 146.48, 136.02, 135.66, 129.88, 128.58, 128.51, 128.49, 127.14, 126.78, 120.32, 105.80, 102.57, 87.00, 84.77, 28.04, 27.12, 19.06. **HRMS (ESI-TOF) m/z**: [M+H]+ Calcd. for $C_{25}H_{27}O_6$ 423.1802; found 423.1087. $[\alpha]^{23.6}_D$ = +82.60 (c = 0.02, EtOAc).

tert-butyl 2-(-8-butyl-1,7-dioxaspiro[5.6]dodec-2-en-2-yl)-2-oxoacetate **(3o)**

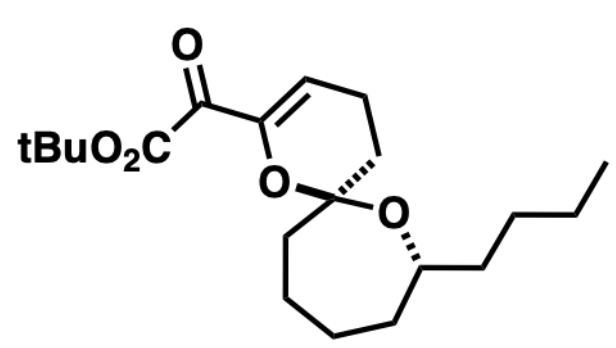

Prepared according to **General Procedure 6** using tert-butyl 2,3-dioxopent-4-enoate (**2a•H₂O**) (1.2 equiv, 1.06 mmol) and 2-butyl-7-methyleneoxepane (**1o**) (1.0 equiv, 0.880 mmol) in DCM (5.3 mL). Purified and isolated using 10% EtOAc in hexanes. 81% yield (0.450 mmol), 11:1 d.r, yellow oil. **^{1}H NMR** (500 MHz, $CDCl_3$) δ 3.89 (td, J = 9.5, 4.0 Hz, 1H), 2.44 (dddd, J = 19.3, 12.9, 6.5, 2.8 Hz, 1H), 2.19 – 2.01 (m, 3H), 1.96 (dd, J = 15.3, 7.9 Hz, 1H), 1.87 – 1.76 (m, 1H), 1.71 – 1.58 (m, 2H), 1.53 (s, 9H), 1.49 – 1.00 (m, 11H), 0.78 (t, J = 7.0 Hz, 3H). **^{13}C NMR** (126 MHz, $CDCl_3$) δ 182.85, 163.76, 146.51, 120.43, 111.99, 84.18, 72.85, 39.94, 36.93, 36.19, 30.71, 29.34, 27.96, 27.83, 22.57, 22.44, 18.92, 13.95. **HRMS (ESI-TOF) m/z**: [M+H]+ Calcd. for $C_{20}H_{33}O_5$, 353.2323; found 353.2326

tert-butyl 2-oxo-2-(1,7-dioxaspiro[5.5]undec-2-en-2-yl)acetate **(3p)**
tert-butyl 2-((3,4-dihydro-2H-pyran-6-yl)methyl)-2-hydroxy-3-oxopent-4-enoate **(5p)**

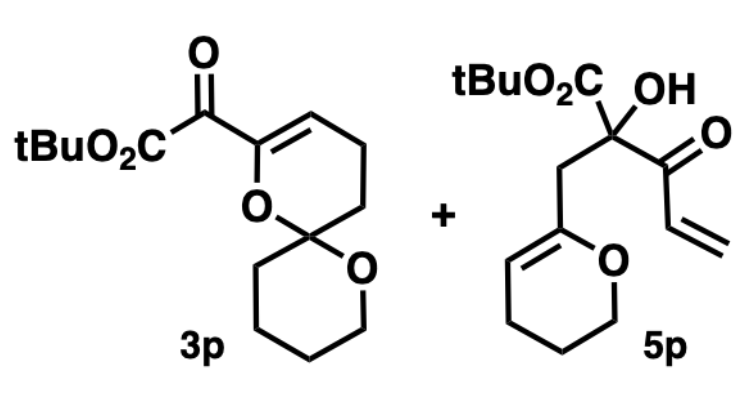

Prepared according to **General Procedure 6** using tert-butyl 2,3-dioxopent-4-enoate (**2a•H₂O**) (1.0 equiv, 0.890 mmol) and 2-methylenetetrahydro-2H-pyran[14] (2.0 equiv, 1.78 mmol, 30% wt.) in DCM (4.2 mL), giving a 1:1.6 ratio of **3p**:**5p**. Both compounds were purified and isolated using 10-20% EtOAc in hexanes. 41% yield (**3l**) (0.36 mmol), clear oil. **^{1}H NMR** (600 MHz, $CDCl_3$, **3l**) δ 6.28 – 6.13 (m, 1H), 3.80 (td, J = 11.8, 3.0 Hz, 1H), 3.70 – 3.54 (m, 1H), 2.43 (dddd, J = 19.4, 12.3, 7.0, 3.0 Hz, 1H), 2.17 (dt, J = 19.6, 5.2 Hz, 1H), 2.02 (dtd, J = 17.9, 13.2, 4.1 Hz, 1H), 1.85 (dtt, J = 11.4, 7.0, 1.6 Hz, 2H), 1.69 – 1.54 (m, 5H), 1.57 (s, 9H). **^{1}H**

NMR (600 MHz, $CDCl_3$, **5l**) δ 6.75 (dd, *J* = 17.2, 10.5 Hz, 1H), 6.41 (dd, *J* = 17.3, 1.7 Hz, 1H), 5.75 (dd, *J* = 10.5, 1.7 Hz, 1H), 4.60 (t, *J* = 3.8 Hz, 1H), 4.17 (s, 1H), 3.93 – 3.78 (m, 2H), 2.83 (d, *J* = 14.7 Hz, 1H), 2.60 (d, *J* = 14.7 Hz, 1H), 2.08 – 1.88 (m, 2H), 1.75 (dd, *J* = 11.0, 5.8 Hz, 2H), 1.43 (s, 9H). **^{13}C NMR** (151 MHz, $CDCl_3$, **5l**) δ 194.08, 169.86, 149.13, 130.47, 129.98, 100.36, 83.53, 81.56, 65.96, 39.86, 27.84, 22.16, 20.30. **HRMS (ESI-TOF, 3l) m/z**: [M+H]+ Calcd. for $C_{15}H_{23}O_5$, 283.1540; found. **HRMS (ESI-TOF, 5l) m/z**: [M+H]+ Calcd. for $C_{15}H_{23}O_5$, 283.1540; found 283.1525

tert-butyl 2-(2-butyl-4-methyl-1,6-dioxaspiro[4.5]dec-7-en-7-yl)-2-oxoacetate **((±)-3r)**

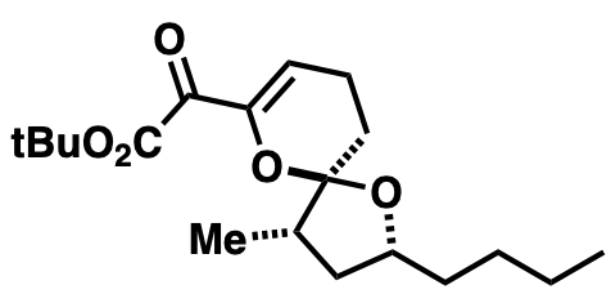

Prepared according to **General Procedure 6** using tert-butyl 2,3-dioxopent-4-enoate (**2a•H_2O**) (1.5 equiv, 0.778 mmol) and 5-butyl-3-methyl-2-methylenetetrahydrofuran (**1r**) (1.0 equiv, 0.519 mmol) in DCM (1.7 mL). Purified and isolated using 7.5% EtOAc in hexanes. 85% yield (0.441 mmol), >20:1 d.r., 85.5% purity, yellow oil. **^{1}H NMR** (500 MHz, $CDCl_3$) δ 6.15 (dd, *J* = 5.7, 3.0 Hz, 1H), 4.10 (p, *J* = 6.9 Hz, 1H), 2.53 – 2.39 (m, 2H), 2.28 (dtt, *J* = 18.0, 6.0, 3.0 Hz, 1H), 1.85 (dd, *J* = 12.8, 4.9 Hz, 1H), 1.77 – 1.59 (m, 3H), 1.56 (s, 8H), 1.49 – 1.41 (m, 2H), 1.30 (m, 3H), 1.21 – 1.11 (m, 1H), 1.01 (d, *J* = 7.2 Hz, 3H), 0.88 (t, *J* = 6.9 Hz, 3H). **^{13}C NMR** (126 MHz, $CDCl_3$) δ 182.84, 163.78, 146.54, 119.05, 108.51, 84.28, 78.04, 41.79, 39.01, 35.70, 28.10, 27.99, 25.84, 22.73, 19.34, 16.69, 14.04. **HRMS (ESI-TOF) m/z**: [M+H]+ Calcd. for $C_{19}H_{31}O_5$, 339.2166; found 339.2172

tert-butyl 2-((6R,8R,11S)-8-butyl-11-methyl-1,7-dioxaspiro[5.5]undec-2-en-2-yl)-2-oxoacetate **((±)-3s)**

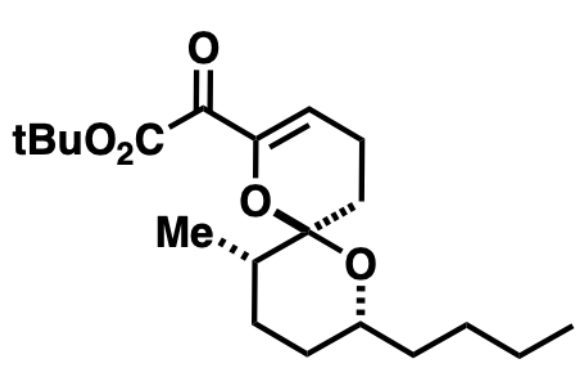

Prepared according to **General Procedure 6** using tert-butyl 2,3-dioxopent-4-enoate (**2a•H_2O**) (1.1 equiv, 0.490 mmol) and 6-butyl-3-methyl-2-methylenetetrahydro-2H-pyran (**1s**) (1.0 equiv, 0.446 mmol) in DCM (1.5 mL). Purified using 7.5% EtOAc in hexanes. 61% yield (0.272 mmol), >20:1 d.r., yellow oil, 89% purity **coelutes with some ene product* ***5s***. **^{1}H NMR** (500 MHz, $CDCl_3$) δ 6.20 (dd, *J* = 5.6, 3.0 Hz, 1H), 3.65 (ddt, *J* = 11.7, 7.7, 3.4 Hz, 1H), 2.47 – 2.30 (m, 2H), 2.22 – 2.13 (m, 1H), 1.98 (dd, *J* = 13.6, 6.9 Hz, 1H), 1.87 (dq, *J* = 7.7, 5.6 Hz, 1H), 1.56 (s, 10H), 1.53 – 1.45 (m, 2H), 1.39 (ddt, *J* = 10.1, 5.7, 2.5 Hz, 2H), 1.33 – 1.17 (m, 6H), 1.04 (d, *J* = 7.2 Hz, 3H), 0.84 (t, *J* = 6.9 Hz, 3H).**^{13}C NMR** (126 MHz, $CDCl_3$) δ 183.02, 163.89, 145.26, 119.22, 99.97, 84.24, 70.84, 35.62, 33.58, 28.03, 27.47, 25.66, 25.55, 22.46, 18.64, 14.67, 14.08. **HRMS (ESI-TOF) m/z**: [M+H]+ Calcd. for $C_{20}H_{33}O_5$, 353.2323; found 353.2317

tert-butyl 2-(10-methyl-2-phenyl-1,6-dioxaspiro[4.5]dec-7-en-7-yl)-2-oxoacetate **((±)-3t)**

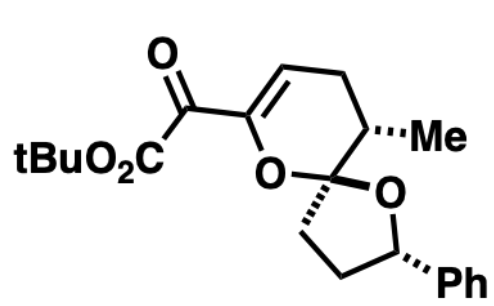

Prepared according to **General Procedure 6** using tert-butyl 2,3-dioxopent-4-enoate (**2a•H_2O**) (1.75 equiv, 0.51 mmol) and (E)-2-ethylidene-5-phenyltetrahydrofuran (**1t**) (1.0 equiv, 0.29 mmol) in DCM (0.98 mL). Purified using 7.5% EtOAc in hexanes. 68% yield (0.20 mmol), >20:1 d.r., white solid. **^{1}H NMR** (500 MHz, $CDCl_3$) δ 7.31 – 7.19 (m, 5H), 6.12 (dd, *J* = 5.6, 3.0 Hz, 1H), 5.23 (t, *J* = 7.2 Hz, 1H), 2.77 (ddd, *J* = 19.4, 6.7, 3.1 Hz, 1H), 2.59 (dt, *J* = 13.1, 7.8 Hz, 1H), 2.26 (ddd, *J* = 13.3, 8.7, 4.7 Hz, 1H),

2.19 – 2.08 (m, 1H), 2.06 – 1.92 (m, 2H), 1.84 (tt, *J* = 11.1, 5.5 Hz, 1H), 1.53 (s, 9H), 0.99 (d, *J* = 7.0 Hz, 3H). **^{13}C NMR** (126 MHz, $CDCl_3$) δ 182.47, 163.55, 145.56, 142.32, 128.39, 127.47, 125.67, 118.66, 110.15, 84.41, 80.75, 34.49, 32.91, 32.63, 28.00, 27.67, 16.39. **HRMS (ESI-TOF) m/z**: [M+H]+ Calcd. for $C_{21}H_{27}O_5$, 359.1853; found 359.1841

methyl 2-oxo-2-(2-phenyl-1,6-dioxaspiro[4.5]dec-7-en-7-yl)acetate **((±)-3u)**

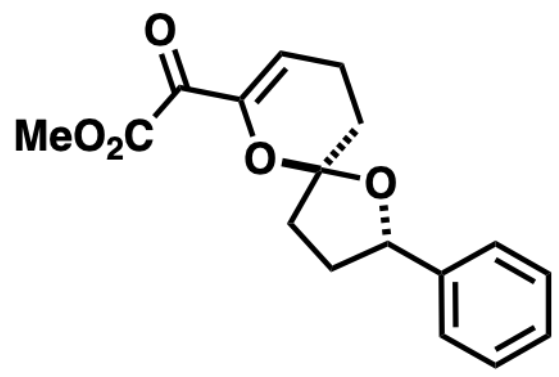

Prepared according to **General Procedure 6** using methyl 2,2-dihydroxy-3-oxopent-4-enoate (**2b•H_2O**) (1.3 equiv, 1.74 mmol) and 2-methylene-5-phenyltetrahydrofuran (**1c**) (1.0 equiv, 1.34 mmol) in DCM (5.81 mL). Purified using 20% EtOAc in hexanes. 76% yield (1.03 mmol), >20:1 d.r., white solid. **^{1}H NMR** (500 MHz, $CDCl_3$) δ 7.37 – 7.22 (m, 5H), 6.32 (dd, *J* = 5.6, 3.1 Hz, 1H), 5.25 (t, *J* = 7.2 Hz, 1H), 3.87 (s, 3H), 2.70 – 2.57 (m, 2H), 2.43 – 2.30 (m, 2H), 2.13 – 1.96 (m, 3H), 1.90 (dddd, *J* = 12.5, 10.0, 7.1, 5.6 Hz, 1H). **^{13}C NMR** (126 MHz, $CDCl_3$) δ 181.74, 163.98, 146.73, 142.07, 128.49, 127.65, 125.81, 121.31, 107.35, 80.94, 52.81, 36.92, 33.26, 29.30, 20.01. **HRMS (ESI-TOF) m/z**: [M+H]+ Calcd. for $C_{17}H_{19}O_5$, 303.1227; found 303.1226

tert-butyl 2-hydroxy-4-methyl-3-oxo-2-((5-phenyl-4,5-dihydrofuran-2-yl)methyl)pent-4-enoate **(5c)**

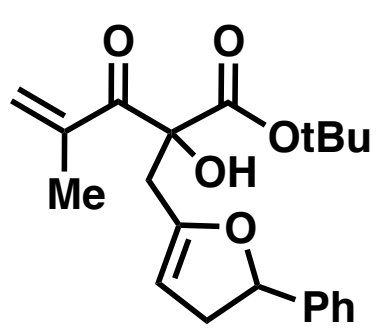

5c

Prepared according to **General Procedure 6** using tert-butyl 2,2-dihydroxy-4-methyl-3-oxopent-4-enoate (**2c•H_2O**) (1.0 equiv, 0.463 mmol) and 2-methylene-5-phenyltetrahydrofuran (**1c**) (1.1 equiv, 0.509 mmol) in DCM (2.31 mL). Purified using 7.5% EtOAc in hexanes. 76% yield (*mixture of diastereomers*) (0.352 mmol), 3:1 d.r., yellow solid. **^{1}H NMR** (500 MHz, $CDCl_3$) δ 7.39 – 7.27 (m, 5H), 6.10 (dd, *J* = 4.2, 1.0 Hz, 1H), 5.81 (dd, *J* = 3.8, 2.3 Hz, 1H), 5.43 (t, *J* = 10.1 Hz, 1H), 4.77 (dt, *J* = 3.0, 1.8 Hz, 1H), 4.28 (s, 1H), 3.09 – 2.94 (m, 2H), 2.88 (ddq, *J* = 14.6, 12.1, 1.4 Hz, 1H), 2.59 (m, 1H), 1.87 (s, 3H), 1.40 (s, 9H). **^{13}C NMR** (126 MHz, $CDCl_3$) δ 195.42, 195.37, 170.35, 170.26, 152.41, 152.31, 142.65, 142.56, 141.16, 141.12, 128.47, 128.43, 127.68, 127.66, 126.65, 126.64, 125.91, 125.80, 125.74, 98.61, 98.55, 83.46, 83.44, 83.35, 83.32, 80.76, 80.45, 39.01, 38.69, 35.08, 35.00, 31.60, 28.10, 27.71, 27.67, 22.67, 19.08, 19.04, 14.13. **HRMS (ESI-TOF) m/z**: [M+H]+ Calcd. for $C_{21}H_{27}O_5$, 359.1853; found 359.1823

tert-butyl (E)-2-hydroxy-3-oxo-2-((5-phenyl-4,5-dihydrofuran-2-yl)methyl)hex-4-enoate **(5d)**

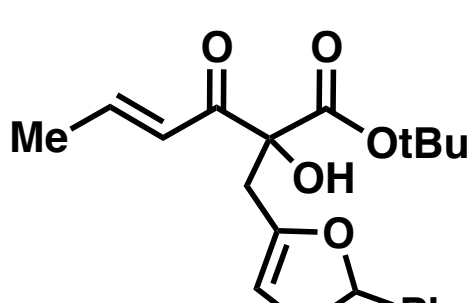

5d

Prepared according to **General Procedure 6** using tert-butyl (E)-2,2-dihydroxy-3-oxohex-4-enoate (**2d•H_2O**) (1.1 equiv, 0.515 mmol) and 2-methylene-5-phenyltetrahydrofuran (**1c**) (1.0 equiv, 0.468 mmol) in DCM (1.56 mL). Purified using 7.5% EtOAc in hexanes. 83% yield (*mixture of diastereomers*) (0.352 mmol), 2.6:1 d.r., light yellow oil. **^{1}H NMR** (500 MHz, $CDCl_3$) δ 7.39 – 7.27 (m, 5H), 7.08 (dq, *J* = 21.5, 7.0 Hz, 1H), 6.54 (d, *J* = 15.4 Hz, 1H), 5.45 (t, *J* = 9.8 Hz, 1H), 4.76 (d, *J* = 2.5 Hz, 1H), 4.29 (s, 1H), 3.11 – 2.96 (m, 2H), 2.82 (dd, *J* = 15.2, 4.5 Hz, 1H), 2.59 (ddd, *J* = 15.3, 11.8, 9.6 Hz, 1H), 1.91 (d, *J* = 5.9 Hz, 3H), 1.40 (s, 9H). **^{13}C NMR** (126 MHz, $CDCl_3$) δ 193.44, 193.35, 169.79, 169.69, 152.47, 152.41, 145.89, 143.01, 142.96, 128.42, 128.39, 127.55, 127.53, 125.73, 125.67, 124.88, 124.84, 97.78, 97.68, 83.67, 83.58, 82.95, 82.92, 81.41, 81.26, 39.01, 38.83, 33.51, 33.48, 27.71, 18.55. **HRMS (ESI-TOF) m/z**: [M+H]+ Calcd. for $C_{21}H_{27}O_5$, 359.1853; found 358.1828

2.3.1 Complete screen of conditions for cycloaddition of 1q

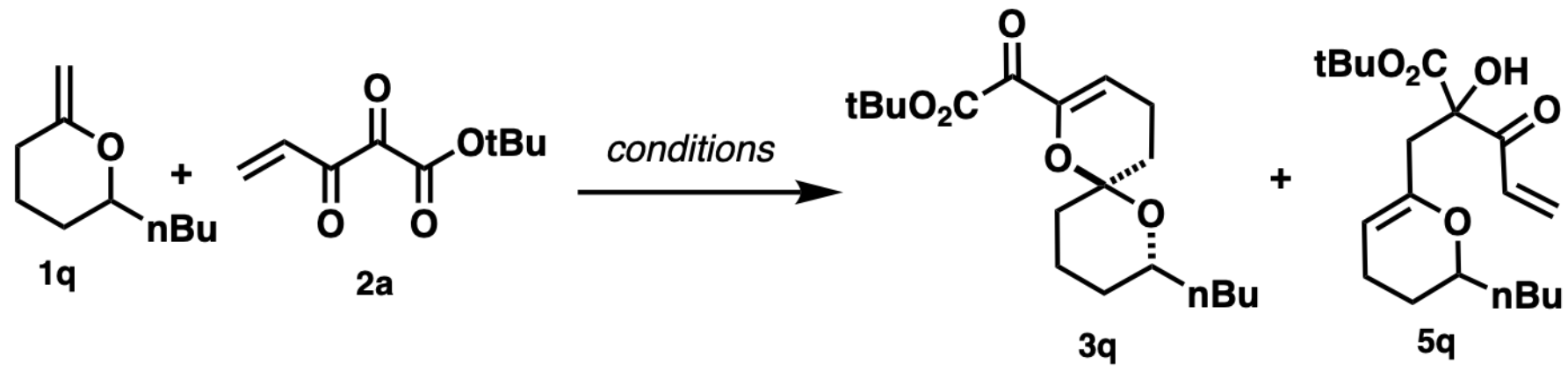

Entry	*Conditions*	*Result*
1	DCM, 23 ºC	31% yield, **3q:5q =** 1:1.6
2	DCM, 0 ºC	Slower progression, no increase in selectivity for **3q**
3	DCM, - 78 ºC	Slower progression, no increase in selectivity for **3q**
4	THF, 23 ºC	67% yield, **3q:5q =** 2.8:1
5	PhMe, 23 ºC	44% yield, **3q:5q =** 1.2:1
6	HFIP, 23 ºC	Decomposition of **2a**

tert-butyl 2-((6S)-8-butyl-1,7-dioxaspiro[5.5]undec-2-en-2-yl)-2-oxoacetate **(3q)**

^{1}H NMR (500 MHz, $CDCl_3$) δ 6.20 (dd, *J* = 5.6, 3.0 Hz, 1H), 3.72 – 3.64 (m, 1H), 2.48 (dddd, *J* = 19.3, 12.1, 6.9, 3.0 Hz, 1H), 2.16 (dt, *J* = 19.6, 5.8 Hz, 1H), 2.02 (qt, *J* = 14.5, 4.5 Hz, 1H), 1.88 – 1.80 (m, 2H), 1.71 – 1.59 (m, 3H), 1.56 (d, *J* = 0.9 Hz, 10H), 1.53 – 1.28 (m, 6H), 1.28 – 1.26 (m, 1H), 1.21 (d, *J* = 6.2 Hz, 1H), 0.84 (t, *J* = 6.7 Hz, 3H). ^{13}C NMR (126 MHz, $CDCl_3$) δ 183.03, 163.88, 145.25, 119.18, 97.15, 84.24, 70.75, 35.56, 33.86, 31.14, 30.96, 28.03, 27.46, 22.42, 18.58, 14.07, 1.03. **HRMS (ESI-TOF) m/z:** [M+H]+ Calcd. for $C_{19}H_{31}O_5$ 339.2166; found 339.2174

2.4. α-Keto ester derivatizations

7-(hydroxymethyl)-2-phenyl-1,6-dioxaspiro[4.6]undecan-8-one (**6**)

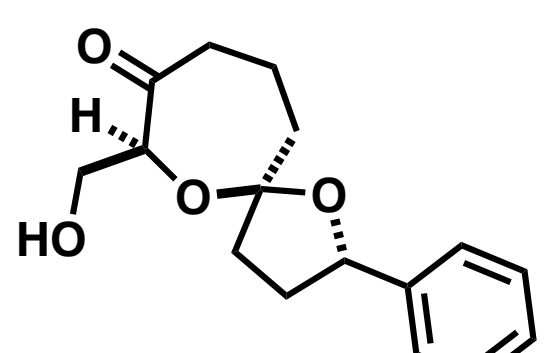

6

3u (1.0 equiv, 331 µmol) was dissolved in methanol (1.7 mL). Then, $NaBH_4$ (3.0 equiv, 992 µmol) was added portionwise. The reaction mixture was stirred at room temperature for 3h. Upon completion of the reaction, solid NH_4Cl was added, the MeOH was concentrated off. The residue was taken up in $CHCl_3$ and stirred with NH_4Cl in the absence of water overnight. Then, the mixture was filtered and concentrated under reduced pressure. The crude residue was analyzed by 1H NMR (1.7:1 d.r.) and purified using silica gel chromatography (40% EtOAc in hexanes) to give **6** in an overall yield of 73% (240 µmol, *major + minor diastereomer*, *48% isolated yield of the major diastereomer*). **^{1}H NMR** (500 MHz, $CDCl_3$) δ 7.39 – 7.26 (m, 6H), 5.15 – 5.09 (m, 1H), 4.34 (t, *J* = 5.0 Hz, 1H), 3.84 (dp, *J* = 10.6, 5.6 Hz, 2H), 2.82 (ddd, *J* = 14.2, 10.8, 5.2 Hz, 1H), 2.59 – 2.47 (m, 2H), 2.28 – 2.15 (m, 2H), 2.11 – 2.00 (m, 2H), 1.98 – 1.81 (m, 3H). **^{13}C NMR** (126 MHz, $CDCl_3$) δ 214.75, 142.25, 128.46, 127.55, 125.55, 110.15, 80.77, 76.35, 63.69, 42.00, 38.13, 38.10, 32.96, 18.43. **HRMS (ESI-TOF) m/z:** [M+H]+ Calcd. for $C_{16}H_{21}O_4$ 277.1434; found 277.1434

** assignments of structure and major diastereomer were confirmed using 1H-13C HMBC and 1H-1H NOSEY, as the following key interactions were observed*

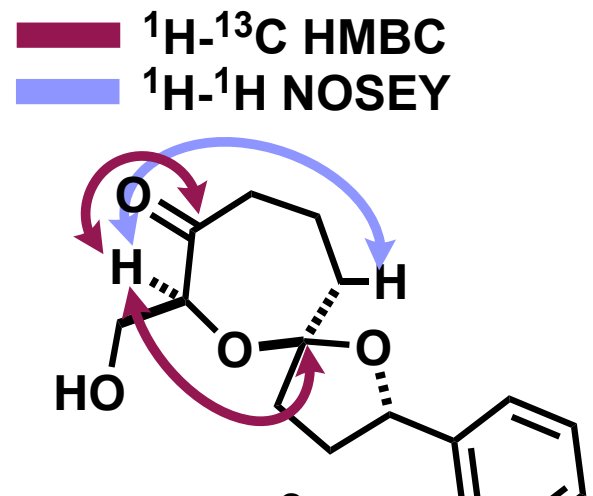

6

1-((2S,5R)-2-phenyl-1,6-dioxaspiro[4.5]dec-7-en-7-yl)ethane-1,2-diol (**7**)

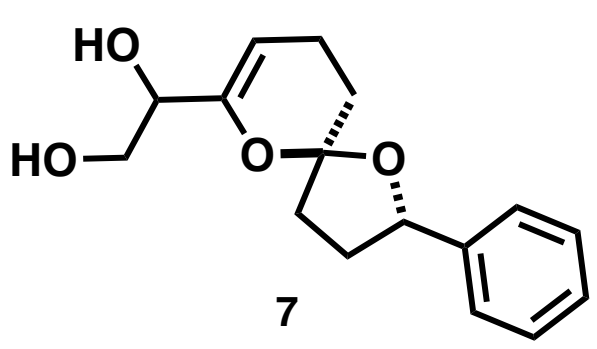

7

Lithium aluminum hydride (1.3 equiv, 2 molar in THF, 0.420 mmol) was cooled in an ice bath under argon. A solution of **3u** (1.0 equiv, 0.331 mmol) in THF (221 µL) was added dropwise to this mixture. The reaction was warmed to room temperature and then stirred for 18 h. The reaction was quenched by careful addition of water (100 µL) then 1 M sodium hydroxide (150 µL). The resulting mixture was refluxed for 1 h. The mixture was cooled to room temperature, diluted with THF (90 mL) and filtered. The filtrate was dried over Na_2SO_4 and the solvent was removed to give a red oil that was passed *quickly* through a short silica plug that was slurried with 5% Et_3N in hexane (50% EtOAc in hexanes) to give **7** as a clear oil. 79% yield (0.261 mmol), ~1:1 mixture of diastereomers at the secondary alcohol. **^{1}H NMR** (500 MHz, $CDCl_3$) δ 7.32 (q, J = 8.4 Hz, 3H), 7.26 (s, 2H), 5.22 (q, J = 7.1 Hz, 1H), 4.98 (dt, J = 7.9, 3.5 Hz, 1H), 4.09 (s, 1H), 3.80 – 3.64 (m, 2H), 2.55 (dq, J = 12.3, 7.7 Hz, 1H), 2.38 (dq, J = 16.2, 8.2 Hz, 1H), 2.24 – 2.09 (m, 2H), 2.07 – 1.88 (m, 4H). **^{13}C NMR** (126 MHz, $CDCl_3$) δ 149.80, 149.74, 142.36, 128.38, 127.46, 125.62, 107.25, 107.03, 98.74, 97.95, 80.75, 80.70, 72.65, 72.19, 64.97, 64.95, 36.68, 36.45, 33.24, 33.20, 30.19, 18.51, 18.33. **HRMS (ESI-TOF) m/z:** [M+H]+ Calcd. for $C_{16}H_{21}O_4$ 277.1434; found 277.1439

2-phenyl-1,6-dioxaspiro[4.5]dec-7-ene-7-carbaldehyde (**8**)

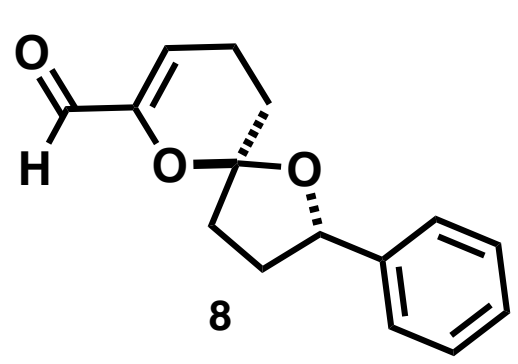

8

Diol **7** (1.0 equiv, 29 µmol) was dissolved in 30 µL of a 1:1 mixture CH2Cl2/H2O in an ice bath. $NaIO_4$ (1.0 equiv, 29 µmol) was added and the solution was stirred for 2 h at 0 °C. The layers were separated and the aqueous layer was extracted 2x with DCM. The organic layer was dried over Na_2SO_4 and concentrated under reduced pressure. The crude residue was purified by silica gel flash chromatography (30% EtOAc in hexanes) to give aldehyde **8** as a white solid in 92% yield (27 µmol). **^{1}H NMR** (500 MHz, $CDCl_3$) δ 9.15 (s, 1H), 7.36 – 7.23 (m, 5H), 5.98 (ddd, J = 5.4, 2.9, 1.2 Hz, 1H), 5.27 (t, J = 7.3 Hz, 1H), 2.76 – 2.58 (m, 2H), 2.48 – 2.33 (m, 2H), 2.18 – 2.06 (m, 2H), 2.01 (ddd, J = 13.4, 12.0, 6.3 Hz, 1H), 1.90 (dddd, J = 12.4, 10.0, 7.4, 6.0 Hz, 1H). **^{13}C NMR** (126 MHz, $CDCl_3$) δ 187.05, 151.16, 141.97, 128.39, 127.54, 125.71, 123.91, 107.04, 80.85, 36.92, 33.33, 29.64, 19.78. **HRMS (ESI-TOF) m/z:** [M+H]+ Calcd. for $C_{15}H_{17}O_3$, 245.1172; found 245.1172

2-phenyl-1,6-dioxaspiro[4.5]dec-7-ene-7-carboxylic acid (**9**)

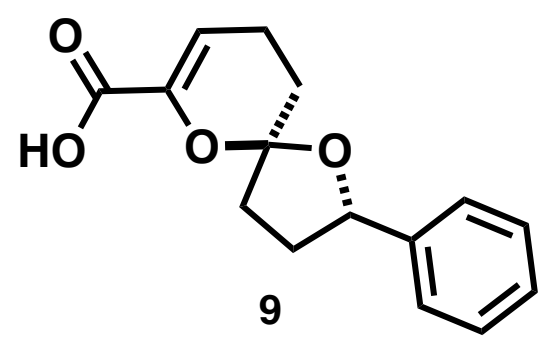

9

Methyl 2-oxo-2-(2-phenyl-1,6-dioxaspiro[4.5]dec-7-en-7-yl)acetate (**3u**) (1.0 equiv, 0.096 mmol) was dissolved in dry THF (0.60 mL). To the solution was added KOTMS (1.2 equiv, 0.115 mmol), and the mixture was stirred at room temperature for 1.5 h. Once complete, camphorsulfonic acid (1.2 equiv, 0.115 mmol) was added to the reaction, and the resulting mixture was stirred for 10 minutes. The THF solution was diluted with EtOAc, washed with water and brine, dried over Na_2SO_4, and concentrated under reduced pressure. The crude residue was passed through a short silica plug (60% EtOAc in hexanes) to give carboxylic acid **9** as a white solid in 67% yield (0.064 mmol). **^{1}H NMR** (500 MHz, $CDCl_3$) δ 7.38 – 7.26 (m, 5H), 6.33 (dd, *J* = 5.4, 3.0 Hz, 1H), 5.29 (t, *J* = 7.2 Hz, 1H), 2.69 – 2.49 (m, 2H), 2.42 – 2.23 (m, 2H), 2.18 – 2.04 (m, 2H), 2.04 – 1.87 (m, 2H). **^{13}C NMR** (126 MHz, $CDCl_3$) δ 165.01, 141.93, 140.90, 128.41, 127.56, 125.68, 113.73, 107.70, 80.90, 36.86, 33.20, 29.37, 19.28. **HRMS (ESI-TOF) m/z:** [M-H]- Calcd. for $C_{15}H_{15}O_4$, 260.1049; found 260.1036

sodium 2-oxo-2-(2-phenyl-1,6-dioxaspiro[4.5]dec-7-en-7-yl)acetate (**10**)

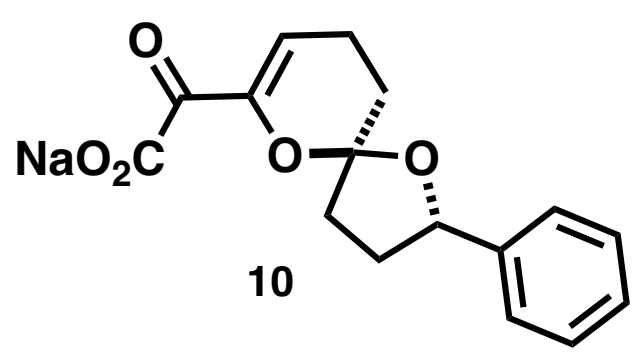

10

To a solution of **3u** (0.165 mmol) in MeOH (2.07 mL) was added a 4M solution of aq. NaOH (0.15 equiv, 0.174 mmol, 43 µL). The solution quickly began to turn yellow and was allowed to stir at room temperature for 1 h. Once complete, the mixture was diluted with EtOAc, dried over Na_2SO_4, and filtered. The resulting solution was concentrated en vacuo to give crude **10** as a yellow solid, which was carried forward without any further purification. **^{1}H NMR** (500 MHz, DMSO-d6) δ 7.40 – 7.21 (m, 5H), 5.89 (t, *J* = 4.0 Hz, 1H), 5.11 (t, *J* = 7.2 Hz, 1H), 2.39 – 2.27 (m, 2H), 2.18 (dddd, *J* = 35.5, 14.0, 9.0, 6.2 Hz, 2H), 2.01 (dddd, *J* = 28.5, 13.5, 6.6, 3.2 Hz, 2H), 1.87 – 1.74 (m, 2H). **^{13}C NMR** (126 MHz, DMSO-d6) δ 193.38, 168.76, 147.04, 142.39, 128.26, 127.32, 125.78, 114.49, 106.43, 79.86, 36.35, 33.01, 29.16, 18.83.

N-methyl-2-phenyl-1,6-dioxaspiro[4.5]dec-7-ene-7-carboxamide (**11**)

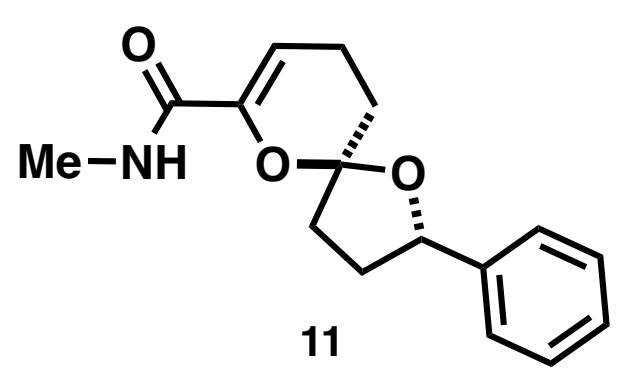

11

To a solution of keto carboxylate **10** (1.0 equiv, 0.160 mmol) in DMSO (805µL) was added N-methylhydroxylamine HCl salt (1.2 equiv, 0.193 mmol) as a solid, and the mixture stirred at 40 °C under Ar atmosphere until the reaction was complete by HPLC (12 h). The mixture was diluted with EtOAc and washed 3x with supersaturated brine to remove DMSO. The organic layer dried over Na_2SO_4, and concentrated under reduced pressure. The crude residue was purified by silica gel flash chromatography (60% EtOAc in hexanes) to give amide **11** in 87% yield (0.139 mmol) as a light yellow oil. **^{1}H NMR** (500 MHz, $CDCl_3$) δ 7.36 – 7.24 (m, 5H), 6.47 (s, 1H), 6.11 (t, *J* = 4.0 Hz, 1H), 5.27 (t, *J* = 7.0 Hz, 1H), 2.89 (d, *J* = 5.0 Hz, 3H), 2.58 (dq, *J* = 12.6, 7.5 Hz, 1H), 2.48 (dddd, *J* = 19.3, 9.8, 6.3, 3.2 Hz, 1H), 2.30 – 2.20 (m, 2H), 2.05 (dtd, *J* = 13.3, 7.1, 2.9 Hz, 2H), 1.94 (ddd, *J* = 12.9, 9.9, 6.2 Hz, 2H). **^{13}C NMR** (151 MHz, $CDCl_3$) δ 163.23, 143.96, 142.20, 128.54, 127.65, 125.69, 108.00, 106.46, 80.98, 36.69, 33.19, 29.93, 25.94, 19.12. **HRMS (ESI-TOF) m/z:** [M+H]+ Calcd. for $C_{16}H_{20}NO_3$, 274.1438; found 274.1464

tert-butyl 2-(8-methyl-2-phenyl-1,6-dioxaspiro[4.5]decan-7-yl)-2-oxoacetate (**12**)

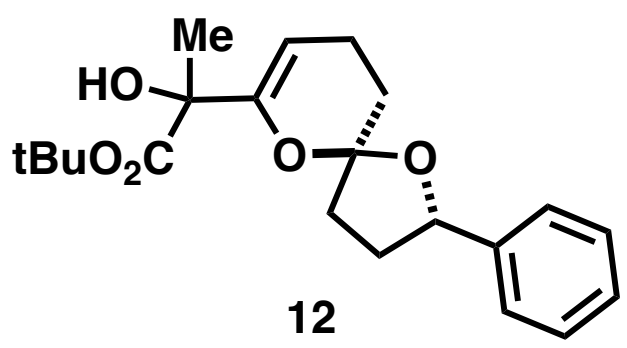

12

A 2.56M solution of MeLi in diethyl ether (2.2 equiv, 1.92 mmol, 749 µL) was added dropwise to a suspension of CuI (1.1 equiv, 958 µmol) in dry THF (2.18 mL) under argon at 0 °C. After stirring for 15 min, a solution of **3c** (1.0 equiv, 871 µmol) in dry THF (242 µL) was added dropwise. The reaction mixture was stirred at room temperature overnight, quenched with a sat. aq. NH_4Cl and extracted 4x with Et_2O. The combined organic layers were washed with brine, dried over Na_2SO_4 and concentrated under reduced pressure. The crude residue was analyzed by 1H NMR (2:1 d.r.), then purified by flash column chromatography on silica gel (15% EtOAc in hexane, **silica gel was slurried with 5% Et_3N in hexane*) to afford tertiary alcohol **12** as a yellow oil in 54% yield (470 µmol, 82% purity), **^{1}H NMR** (500 MHz, $CDCl_3$) δ 7.31 – 7.18 (m, 5H), 5.16 (t, *J* = 7.0 Hz, 1H), 5.00 (ddd, *J* = 4.9, 2.9, 0.8 Hz, 1H), 3.49 (s, 1H), 2.55 – 2.44 (m, 1H), 2.42 – 2.28 (m, 1H), 2.18 – 2.02 (m, 2H), 2.00 – 1.89 (m, 2H), 1.88 – 1.77 (m, 2H), 1.45 (s, 3H), 1.44 (s, 3H), 1.41 (s, 7H). **^{13}C NMR** (126 MHz, $CDCl_3$) δ 173.81, 173.78, 151.92, 151.84, 142.75, 142.73, 128.29, 128.28, 127.26, 125.63, 125.56, 107.25, 107.04, 96.12, 95.90, 82.17, 82.13, 80.45, 80.36, 74.77, 74.74, 36.47, 36.13, 33.18, 33.01, 29.86, 29.84, 27.90, 27.87, 27.80, 27.77, 27.75, 27.71, 22.18, 22.01, 18.70, 18.53. **HRMS (ESI-TOF) m/z:** [M+H]+ Calcd. for $C_{21}H_{29}O_5$, 361.2010; found 361.2021

tert-butyl 2-hydroxy-2-(2-phenyl-1,6-dioxaspiro[4.5]dec-7-en-7-yl)acetate (**13**)

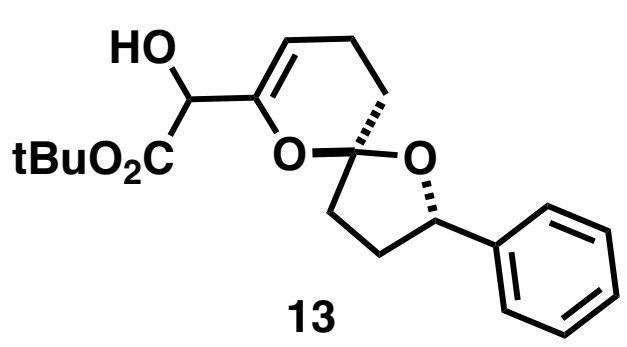

13

LithiumTri-t-Butoxyaluminohydride (0.5 molar, 7.30 equiv, 1.1 mmol) was added dropwise to a suspension of CuBr (3.82 equiv, 0.55 mmol) in THF (0.91 mL). The solution was maintained between 0 and -5 °C for 30 min, then the resulting dark brown suspension was cooled to -78 °C. **3c** (1.0 equiv, 0.15 mmol) was dissolved in a minimal amount of THF and was added rapidly via syringe. After 10 min, the mixture was allowed to warm up to room temp overnight. Then, methanol was added and the mixture was poured into sat. aq. NH_4Cl. The mixture was diluted with Et_2O and the blue (copper) layer was separated. The organic layer was washed with water, dried over Na_2SO_4, and concentrated. Using 1H NMR analysis, the crude indicated quantitative conversion of **3c** to **13,** 1.5:1 d.r. The crude residue was subject to silica gel flash chromatography (15% EtOAc in hexane, **silica gel was slurried with 5% Et_3N in hexane*), and **13** was isolated in 51% yield (76.5 µmol). **The loss of remaining yield was attributed to formation of **S17** during purification, a rearrangement product analogous to **6**.* **^{1}H NMR** (300 MHz, $CDCl_3$, **13**) δ 7.36 – 7.26 (m, 5H), 5.32 – 5.25 (m, 1H), 4.97 (p, J = 2.5 Hz, 1H), 4.34 (dd, J = 7.3, 2.8 Hz, 1H), 3.19 (dd, J = 11.1, 7.5 Hz, 1H), 2.54 (dddd, J = 11.7, 8.1, 5.8, 3.1 Hz, 1H), 2.40 (dtd, J = 16.1, 6.3, 3.2 Hz, 1H), 2.28 – 2.08 (m, 2H), 2.03 – 1.80 (m, 4H), 1.48 (d, J = 8.1 Hz, 9H). **^{13}C NMR** (126 MHz, $CDCl_3$, **13**) δ 170.98, 170.91, 148.75, 148.46, 142.30, 142.26, 127.86, 127.84, 126.82, 125.16, 125.09, 106.80, 106.68, 99.38, 99.12, 81.72, 81.68, 79.97, 79.94, 72.04, 71.96, 36.04, 35.83, 32.83, 32.61, 29.61, 29.55, 27.54, 27.51, 18.37, 18.21. **HRMS (ESI-TOF) m/z:** [M+H]+ Calcd. for $C_{20}H_{27}O_5$, 347.1853; found 347.1846

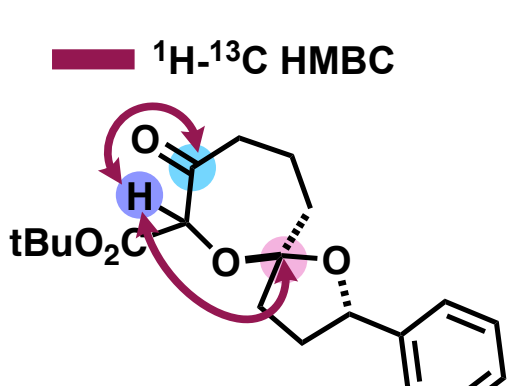

S17

assignment of rearrangement byproduct **S17 was based on comparison of spectral data to that of **6** and was confirmed by key interactions observed in 1H-^{13}C HMBC*

2.4.1 Preliminary studies of keto ester degradation to carboxylic acid

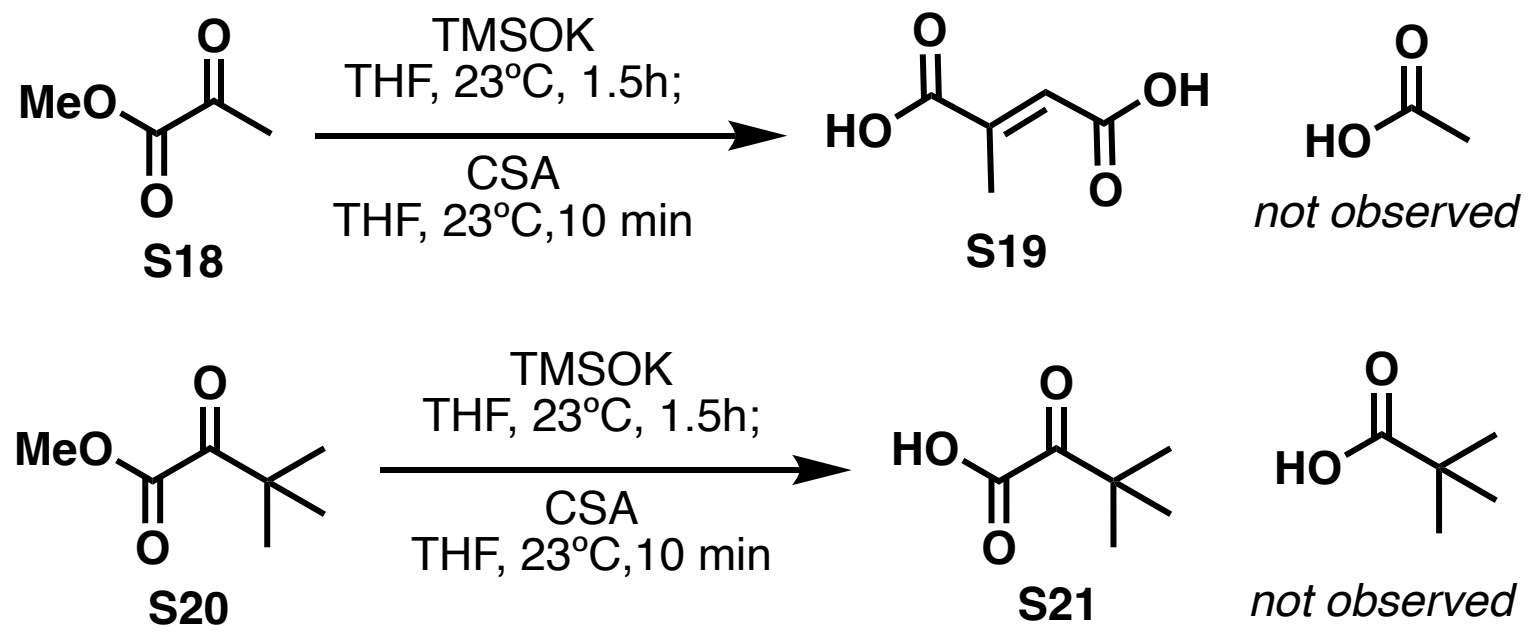

Study with methyl pyruvate (S18)

To evaluate the role of O_2 in the transformation, two reactions were ran concurrently using **S18** (1.469 mmol) and KOTMS (1.2 equiv, 1.763 mmol) in THF (9.2 mL). One was carried out under an O_2 balloon, and the other was freeze-pump-thaw degassed and kept rigorously oxygen free. After stirring for 2h at room temperature, camphorsulfonic acid (1.0 equiv, 1.469 mmol) was added to both reactions, and the resulting mixture was stirred for 10 minutes. An aliquot of each THF solution was analyzed by 1H and 13C NMR to reveal an identical outcome in both reactions: clean formation of the aldol/decarboxylation product to the diacid **S19**. Spectral data was consistent with literature reports.[15] **^{1}H NMR** (600 MHz, $CDCl_3$) δ 6.26 (s, 1H), 1.75 (s, 3H). **^{13}C NMR** (126 MHz, $CDCl_3$) δ 170.89, 169.00, 142.69, 118.87, 22.92.

Study with pivaloyl variant (S20)

To evaluate the transformation without the secondary aldol reaction, a model system without enolic protons (**S20**) was chosen. **S20** (1.0 equiv, 4.162 mmol) was dissolved in dry THF (26.1 mL). To the solution was added KOTMS (1.2 equiv, 4.994 mmol), and the mixture was stirred at room temperature for 1.5 h. Once complete, camphorsulfonic acid (1.0 equiv, 4.162 mmol) was added to the reaction, and the resulting mixture was stirred for 10 minutes. An aliquot of this THF solution was analyzed by 13C NMR to reveal only the saponification product **S21**. No pivalic acid decarboxylation product was observed. Spectral data was consistent with literature reports.[16] **^{13}C NMR** (151 MHz, $CDCl_3$) δ 203.75, 164.67, 42.43, 25.98.

2.5. Synthesis of 20

((5-butylfuran-2-yl)oxy)trimethylsilane (**16**)

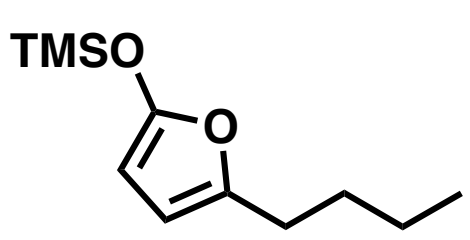

16

To a solution of 5-butylfuran-2(5H)-one (1.0 equiv, 36.88 mmol) in DCM (32.07 mL) at 0°C was added Et_3N (1.4 equiv, 51.63 mmol, 7.20 mL). Then, TMS-OTf (1.1 equiv, 40.57 mmol, 7.489 mL) was added dropwise to the resulting red/brown solution at the same temperature. The reaction was left to stir for 6 hr at room temperature. The mixture was then concentrated in vacuo, where the top layer was collected via pipette and transferred into a separate round-bottom flask. The residue was distilled (44-43 °C, 4 torr) to afford the compound as a colorless oil (5.65g, 72% yield). **^{1}H NMR** (600 MHz, CDCl3) δ 5.76 (d, J = 3.0 Hz, 1H), 4.97 (d, J = 3.0 Hz, 1H), 2.52 – 2.44 (m, 2H), 1.59 – 1.52 (m, 2H), 1.35 (h, J = 7.4 Hz, 2H), 0.91 (t, J = 7.4 Hz, 3H), 0.29 (s, 9H).**^{13}C NMR** (151 MHz, $CDCl_3$) δ 154.92, 146.00, 105.26, 83.15, 30.17, 27.69, 22.16, 13.83, -0.27. **HRMS (ESI-TOF) m/z:** [M+H]+ Calcd. for $C_{11}H_{21}O_2Si$ 213.1311; found 213.1319

(±)-5-butyl-5-(1-hydroxy-3-(trimethylsilyl)prop-2-yn-1-yl)furan-2(5H)-one (**18**)

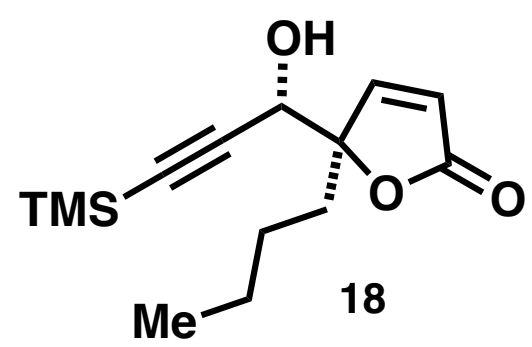

Compound was prepared according to the following literature procedure.[2] At -78°C, zinc bromide (1.0 equiv, 6.357 mmol) was added to a solution of ((5-butylfuran-2-yl)oxy)trimethylsilane (**16**) (1.0 equiv, 6.357 mmol) and 3-trimethylsilylprop-2-ynal (**17**) (80% wt, 1.3 equiv, 8.264 mmol) in DCM (6.357 mL). After stirring for 2.5 h at this temperature, $NaHCO_3$ aqueous solution (10 mL) was added and the reaction mixture allowed to warm to room temperature. The organic layer was separated, and the aqueous layer was extracted with DCM (3 x 20 ml). The combined organic DCM layers were dried over $MgSO_4$. The solvent was removed and the residue was purified by FCC (10% EtOAc in Hex) to give a clear, colorless oil (70% yield, 4.45 mmol, 3:1 dr). **^{1}H NMR (500 MHz, $CDCl_3$)** δ 7.37 (s, 1H), 6.18 (d, *J* = 5.7 Hz, 1H), 4.47 (d, *J* = 3.8 Hz, 1H), 2.26 (d, *J* = 5.6 Hz, 1H), 2.11 – 1.91 (m, 2H), 1.37 – 1.28 (m, 2H), 1.27 – 1.21 (m, 1H), 1.18 – 1.10 (m, 1H), 0.89 (t, *J* = 7.2 Hz, 3H), 0.17 (s, 9H). **^{13}C NMR (126 MHz, $CDCl_3$)** δ 172.12, 156.34, 123.10, 100.87, 93.60, 91.95, 67.03, 31.56, 25.02, 22.76, 13.82, -0.37. **HRMS (ESI-TOF) m/z:** [M+H]+ Calcd. for $C_{14}H_{23}O_3Si$ 267.1416; found 267.1425

(±)-5-butyl-5-(1-((triethylsilyl)oxy)-3-(trimethylsilyl)prop-2-yn-1-yl)furan-2(5H)-one **(S22)**

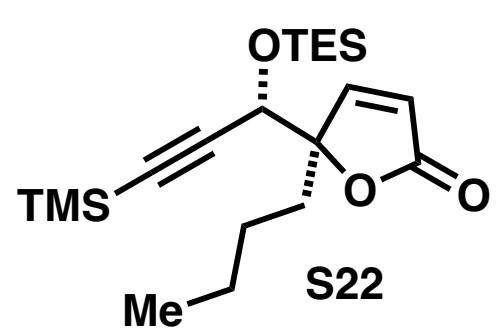

To a solution of **18** (1.0 equiv, 1.610 mmol) in DCM (3.22 mL) at 0°C was added imidazole (1.7 equiv, 2.738 mmol), followed by the dropwise addition of TESCl (1.2 equiv, 1.932 mmol). The reaction was monitored by TLC until disappearance of the starting material (20 min). Once finished, the reaction was quenched with water, diluted with Et_2O (10 mL), and extracted using Et_2O (3 x 10 mL). The solvent was removed in vacuo, and the residue was purified by FCC (2.5% EtOAc in Hex) to give a clear, colorless oil (80% yield, 1.29 mmol). **^{1}H NMR** (500 MHz, $CDCl_3$) δ 7.34 (d, *J* = 5.6 Hz, 1H), 6.15 (d, *J* = 5.7 Hz, 1H), 4.50 (s, 1H), 2.00 – 1.88 (m, 2H), 1.36 – 1.17 (m, 4H), 0.97 (t, *J* = 8.0 Hz, 9H), 0.87 (t, *J* = 7.2 Hz, 3H), 0.75 – 0.58 (m, 6H), 0.13 (s, 9H). **^{13}C NMR** (126 MHz, $CDCl_3$) δ 172.32, 156.60, 122.84, 102.03, 92.47, 91.71, 67.54, 32.06, 25.15, 22.80, 13.85, 6.71, 4.74, -0.46. **HRMS (ESI-TOF) m/z:** [M+H]+ Calcd. for $C_{20}H_{37}O_3Si_2$ 381.2281; found 381. 2294

(±)-5-butyl-5-(1-((triethylsilyl)oxy)-3-(trimethylsilyl)prop-2-yn-1-yl)dihydrofuran-2(3H)-one **(19)**

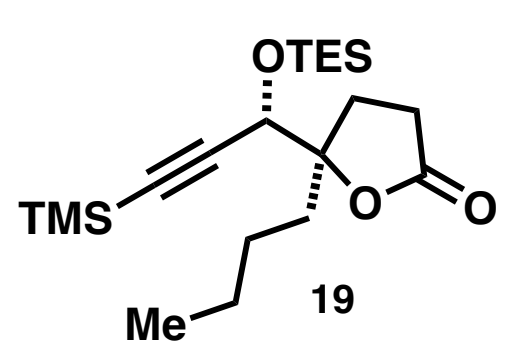

Lithium tri-tert-butoxyaluminum hydride (0.5M in diglyme, 7.30 equiv, 4.906 mmol) was added dropwise to a suspension of Cuprous bromide (3.82 equiv, 2.567 mmol) in THF (4.20 mL) maintained at 0 to -5°C. After 30 min, the resulting dark brown suspension was cooled to -78°C. **S22** (1.0 equiv, 0.672 mmol) was added rapidly via syringe and after 10 min, the mixture was allowed to warm up to 23ºC overnight. Then, methanol (5 mL) was added and the mixture poured into 25 mL of saturated aqueous ammonium chloride solution. Et_2O was added (25 mL) and the mixture was extracted with Et_2O (3 x 20 mL). The combined organic extracts were washed with water, dried, and purified via FCC (2% EtOAc in Hex) to give a clear, colorless oil (70% yield, 0.47 mmol). **^{1}H NMR** (500 MHz, $CDCl_3$) δ 4.39 (s, 1H), 2.67 (ddd, *J* = 17.4, 10.9, 7.4 Hz, 1H), 2.55 – 2.41 (m, 2H), 1.92 (ddd, *J* = 13.5, 10.8, 7.5 Hz, 1H), 1.81 – 1.67 (m, 2H), 1.33 (dd, *J* = 7.0, 3.4 Hz, 4H), 0.97 (t, *J* = 7.9 Hz, 9H), 0.93 – 0.87 (m, 3H), 0.67 (m, 6H), 0.15 (s, 9H).**^{13}C NMR** (126 MHz, $CDCl_3$) δ 177.18, 103.43, 91.48, 88.88, 67.51, 36.62, 29.74, 26.94, 25.10, 22.94, 13.93, 6.75, 4.86, -0.48. **HRMS (ESI-TOF) m/z:** [M+H]+ Calcd. for $C_{20}H_{39}O_3Si_2$ 383.2438; found 383. 2427

(±)- (3-(2-butyl-5-methylenetetrahydrofuran-2-yl)-3-((triethylsilyl)oxy)prop-1-yn-1-yl)trimethylsilane **(1h)**

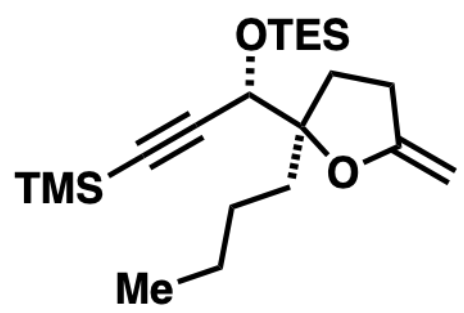

Prepared by following **General Procedure 2** using **19** (1.0 equiv, 392.0 µmol), Petasis reagent (26.8% wt, 2.0 equiv, 783.9 µmol), ethyl pivalate (1.0 equiv, 392.0 µmol) in THF (979.9 µL). After concentrating in vacuo at room temperature, a red oil was obtained and used in the next step without further purification (148 mg, quantitative yield). **^{1}H NMR (500 MHz, CDCl$_3$)** δ 4.35 (s, 1H), 4.14 (s, 1H), 3.70 (s, 1H), 2.74 – 2.63 (m, 1H), 2.62 – 2.52 (m, 1H), 2.25 (ddd, J = 13.2, 10.3, 6.3 Hz, 1H), 1.81 – 1.63 (m, 3H), 1.35 – 1.28 (m, 4H), 0.97 (dd, J = 8.4, 7.5 Hz, 9H), 0.89 (d, J = 7.0 Hz, 3H), 0.65 (ddt, J = 19.8, 15.1, 7.5 Hz, 6H), 0.13 (s, 8H). **^{13}C NMR (126 MHz, CDCl$_3$)** δ 163.62, 105.02, 90.80, 90.05, 78.44, 67.82, 45.47, 35.94, 29.72, 25.45, 23.20, 14.07, 6.79, 4.85, -0.38. **HRMS (ESI-TOF) m/z:** [M+H]+ Calcd. for $C_{21}H_{41}O_2Si_2$ 381.2645; found 381. 2663

(±)-methyl 2-butyl-2-(1-((triethylsilyl)oxy)-3-(trimethylsilyl)prop-2-yn-1-yl)-1,6-dioxaspiro[4.5]dec-7-ene-7-carboxylate (**3h/3h'**)

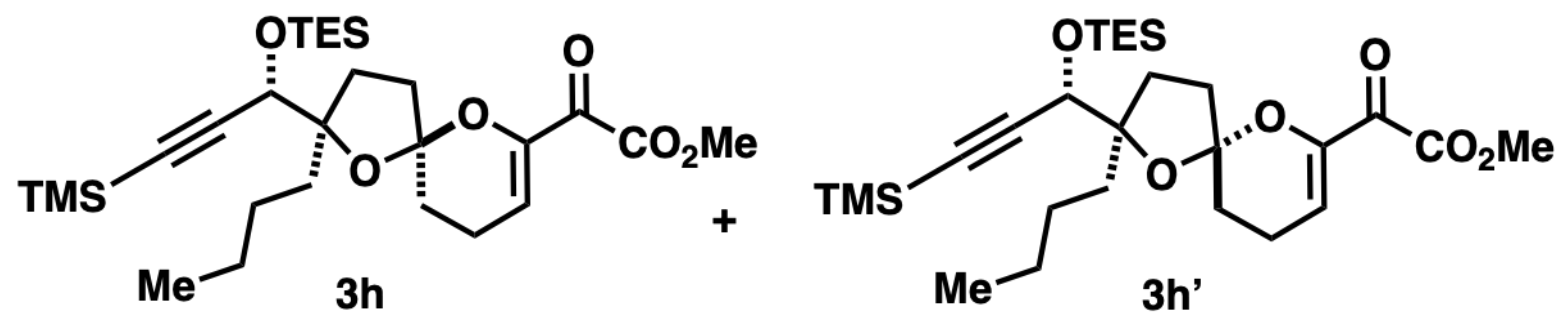

Prepared according to **General Procedure 6** using methyl 2,2-dihydroxy-3-oxopent-4-enoate (**2b•H$_2$O**) (1.0 equiv, 265.4 µmol) and **1h** (1.2 equiv, 318.5 µmol) in DCM. Purified via FCC (1% EtOAc in Hex w/ 5% Et3N) to give a slightly yellow oil. 74% yield, 4:1 d.r. **^{1}H NMR** (500 MHz, CDCl$_3$) δ 6.27 – 6.23 (m, 1H), 4.32 (d, J = 46.3 Hz, 1H), 3.87 (d, J = 9.2 Hz, 3H), 2.56 (dddd, J = 19.7, 11.4, 7.0, 3.4 Hz, 1H), 2.38 – 2.23 (m, 2H), 2.22 – 2.15 (m, 2H), 2.03 – 1.93 (m, 2H), 1.91 – 1.78 (m, 2H), 1.71 (ddd, J = 13.7, 11.7, 4.6 Hz, 1H), 1.35 – 1.26 (m, 2H), 1.23 – 1.16 (m, 2H), 0.94 (dt, J = 24.7, 7.9 Hz, 9H), 0.82 (t, J = 7.1 Hz, 3H), 0.72 – 0.55 (m, 6H), 0.14 (d, J = 10.1 Hz, 9H). **^{13}C NMR** (126 MHz, CDCl$_3$) δ 181.83, 164.15, 146.47, 121.04, 112.02, 107.94, 106.67, 90.28, 88.82, 66.43, 52.59, 38.05, 31.03, 28.81, 25.86, 23.04, 20.06, 14.01, 6.82, 4.92, -0.31. **HRMS (ESI-TOF) m/z:** [M+H]+ Calcd. for $C_{27}H_{47}O_6Si_2$ 523.2911; found 523.2922

** assignments of major and minor diastereomers were confirmed using 1H-1H NOSEY, as the following interactions were observed*

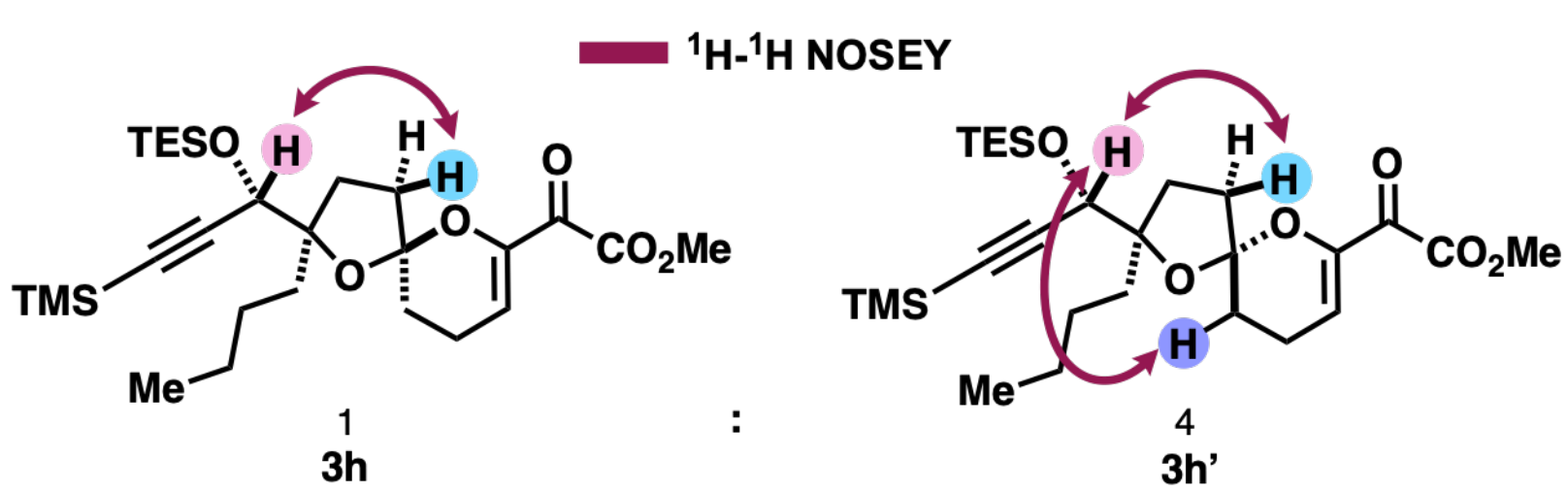

3. References

(1) Lee, A. S.-Y.; Tsao, K.-W.; Chang, Y.-T.; Chu, S.-F. L-Proline-Catalyzed Intramolecular Cyclization of 5-Hydroxypentene to β-Halogenated Tetrahydrofuran. *Tetrahedron Letters* **2007**, *48* (38), 6790–6793. https://doi.org/10.1016/j.tetlet.2007.07.069.

(2) Hornyánszky, G.; Rohály, J.; Novák, L. Facile Synthesis of Mill Moth's Sex Pheromone Components. *Synthetic Communications* **2008**, *38* (10), 1533–1540. https://doi.org/10.1080/00397910801929341.

(3) Malosh, C. F.; Ready, J. M. Catalytic Cross-Coupling of Alkylzinc Halides with α-Chloroketones. *J. Am. Chem. Soc.* **2004**, *126* (33), 10240–10241. https://doi.org/10.1021/ja0467768.

(4) Sigma-Aldrich Co. LLC. (Spectral Data Were Obtained from John Wiley & Sons, Inc.).

(5) Riclea, R.; Gleitzmann, J.; Bruns, H.; Junker, C.; Schulz, B.; Dickschat, J. S. Algicidal Lactones from the Marine Roseobacter Clade Bacterium Ruegeria Pomeroyi. *Beilstein J. Org. Chem.* **2012**, *8* (1), 941–950. https://doi.org/10.3762/bjoc.8.106.

(6) Hayashi, K.; Tanimoto, H.; Zhang, H.; Morimoto, T.; Nishiyama, Y.; Kakiuchi, K. Efficient Synthesis of α,β-Unsaturated Alkylimines Performed with Allyl Cations and Azides: Application to the Synthesis of an Ant Venom Alkaloid. *Org. Lett.* **2012**, *14* (22), 5728–5731. https://doi.org/10.1021/ol302608q.

(7) Li, D. Y.; Shi, K. J.; Mao, X. F.; Zhao, Z. L.; Wu, X. Y.; Liu, P. N. Selective Cyclization of Alkynols and Alkynylamines Catalyzed by Potassium Tert-Butoxide. *Tetrahedron* **2014**, *70* (39), 7022–7031. https://doi.org/10.1016/j.tet.2014.06.078.

(8) Xiang, L.; Hu, T.; Xue, H.; Pan, W.; Xie, Y.; Shen, J. Synthesis and Evaluation of NHC Derivatives and 4'-Fluorouridine Prodrugs. *Organic & Biomolecular Chemistry* **2023**, *21* (13), 2754–2767. https://doi.org/10.1039/d3ob00268c.

(9) Murphy, P. V.; McDonnell, C.; Hämig, L.; Paterson, D. E.; Taylor, R. J. K. Confirmation of the Structure of a Glucono-1,4-Lactone Derivative Obtained from Silylation of Glucono-1,5-Lactone. *Tetrahedron: Asymmetry* **2003**, *14* (1), 79–85. https://doi.org/10.1016/S0957-4166(02)00747-4.

(10) Wasserman, H. H.; DeSimone, R. W.; Boger, D. L.; Baldino, C. M. Singlet Oxygen Oxidation of Bipyrroles: Total Synthesis of d,l- and Meso-Isochrysohermidin. *J. Am. Chem. Soc.* **1993**, *115* (18), 8457–8458. https://doi.org/10.1021/ja00071a069.

(11) Wasserman, H. H.; Frechette, R.; Oida, T.; Van Duzer, J. H. The Chemistry of Vicinal Tricarbonyls. Preparation and Reactions of Acetylenic Tricarbonyls. *J. Org. Chem.* **1989**, *54* (26), 6012–6014. https://doi.org/10.1021/jo00287a007.

(12) Wasserman, H. H.; Parr, J. The Chemistry of Vicinal Tricarbonyls and Related Systems. *Acc. Chem. Res.* **2004**, *37* (9), 687–701. https://doi.org/10.1021/ar0300221.

(13) Guerra Faura, G.; Nguyen, T.; France, S. Catalyst-Controlled Chemodivergent Reactions of 2-Pyrrolyl-α-Diazo-β-Ketoesters and Enol Ethers: Synthesis of 1,2-Dihydrofuran Acetals and Highly Substituted Indoles. *J. Org. Chem.* **2021**, *86* (15), 10088–10104. https://doi.org/10.1021/acs.joc.1c00826.

(14) Aumann, K. M.; Healy, P. C.; Coster, M. J. Arylation of [6,6]-Spiroacetal Enol Ethers: Reactivity and Rearrangement. *Tetrahedron Letters* **2011**, *52* (10), 1070–1073. https://doi.org/10.1016/j.tetlet.2010.12.076.

(15) Sigma-Aldrich (Spectral Data Were Obtained from Advanced Chemistry Development, Inc.).

(16) *Diphenylparabanic Acid as a Synthon for the Synthesis of α-Diketones and α-Ketocarboxylic Acids | The Journal of Organic Chemistry*. https://pubs.acs.org/doi/10.1021/jo202304x (accessed 2026-05-01).

4. NMR spectra

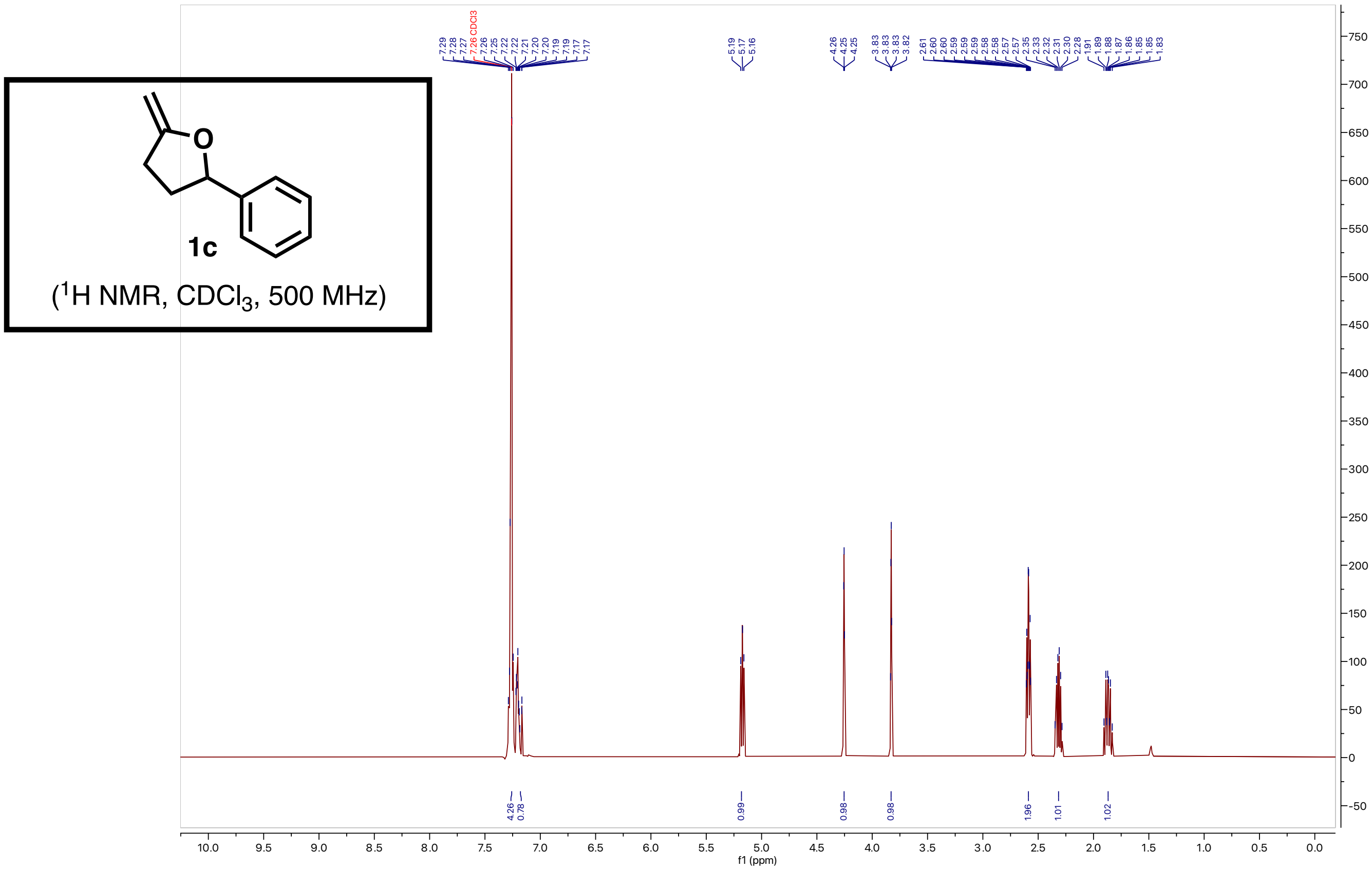

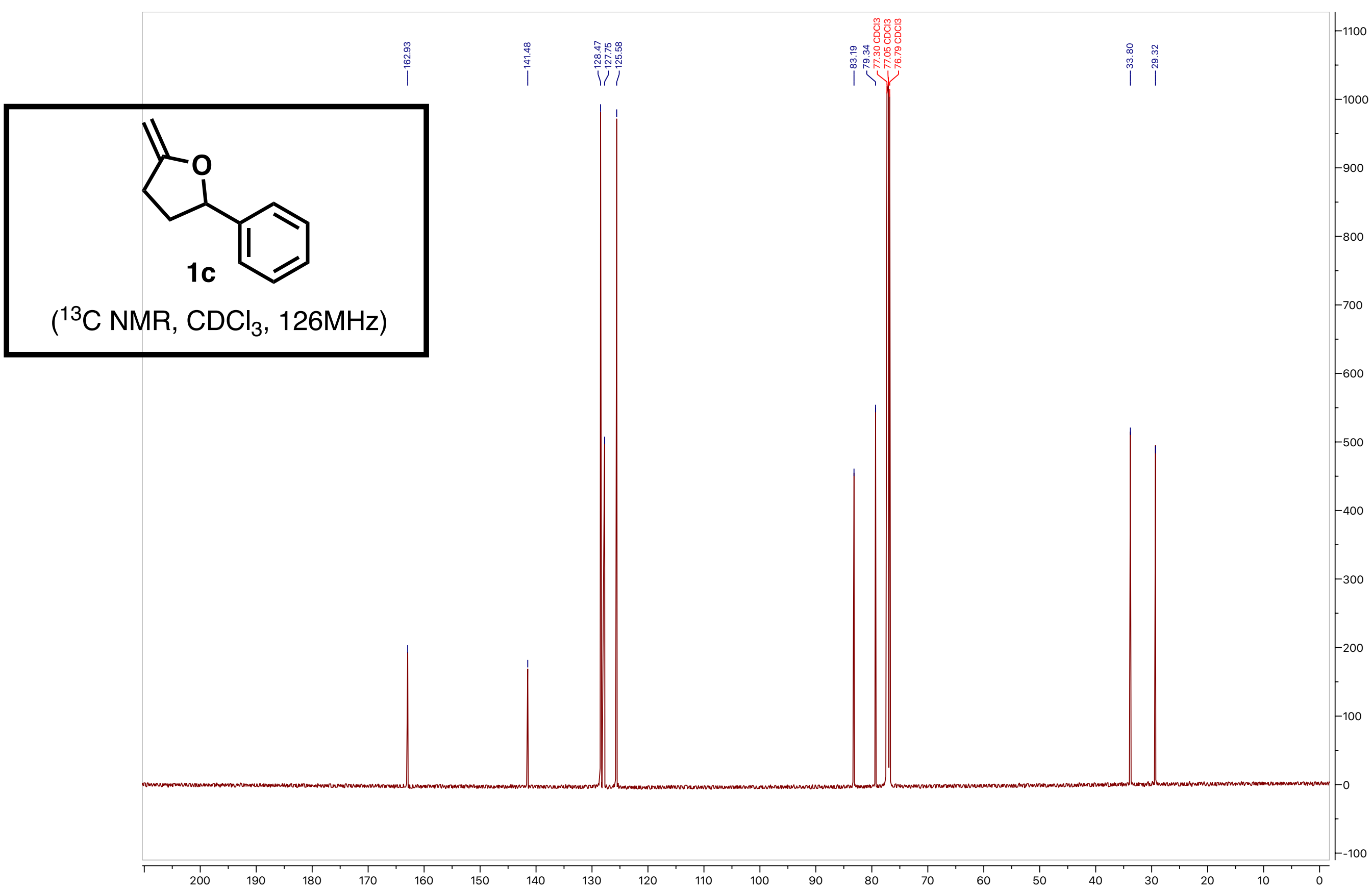

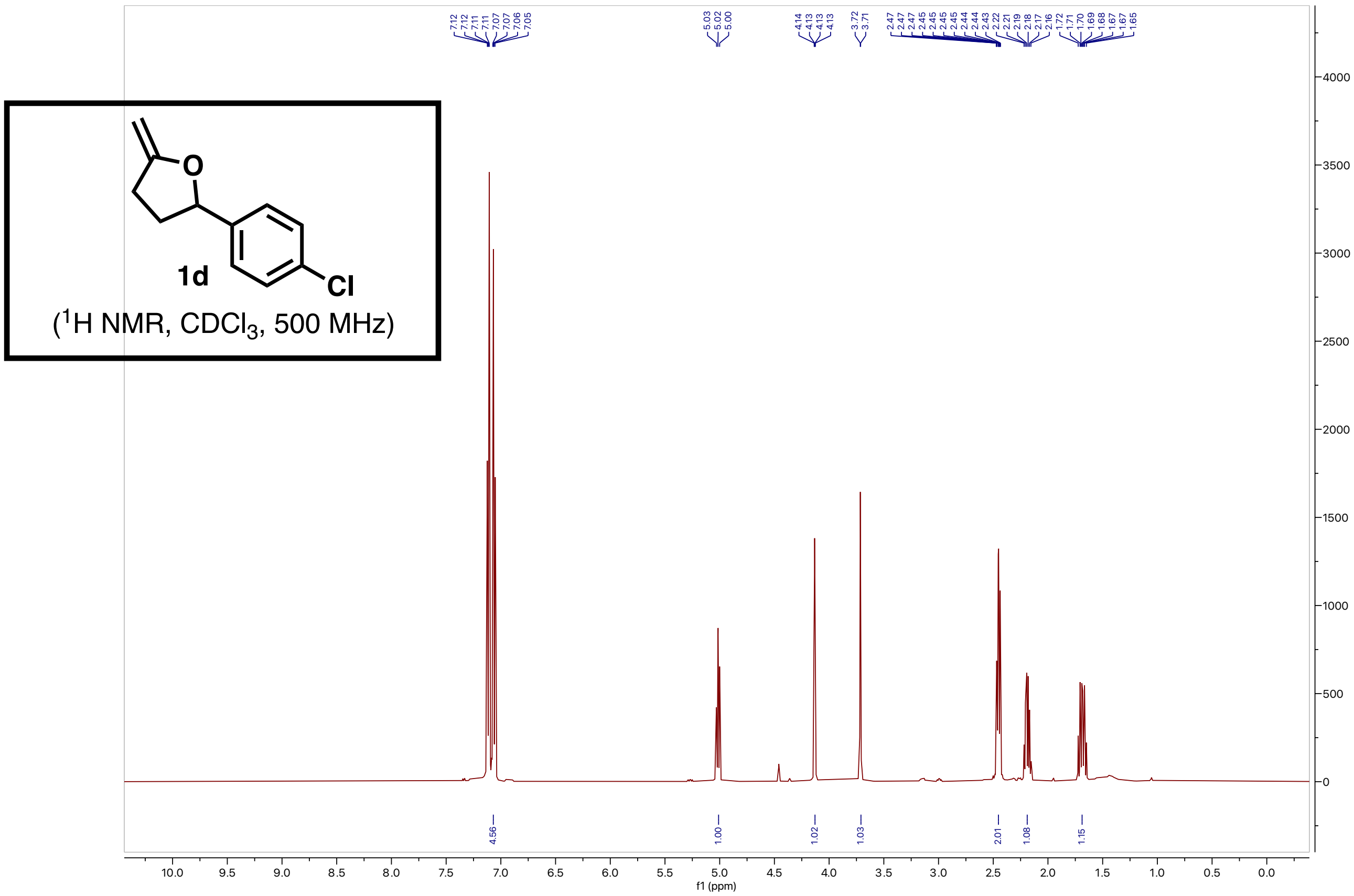
O
1d
Cl
(1H NMR, CDCl3, 500 MHz)
4.56
1.00
1.02
1.03
2.01
1.08
1.15
f1 (ppm)

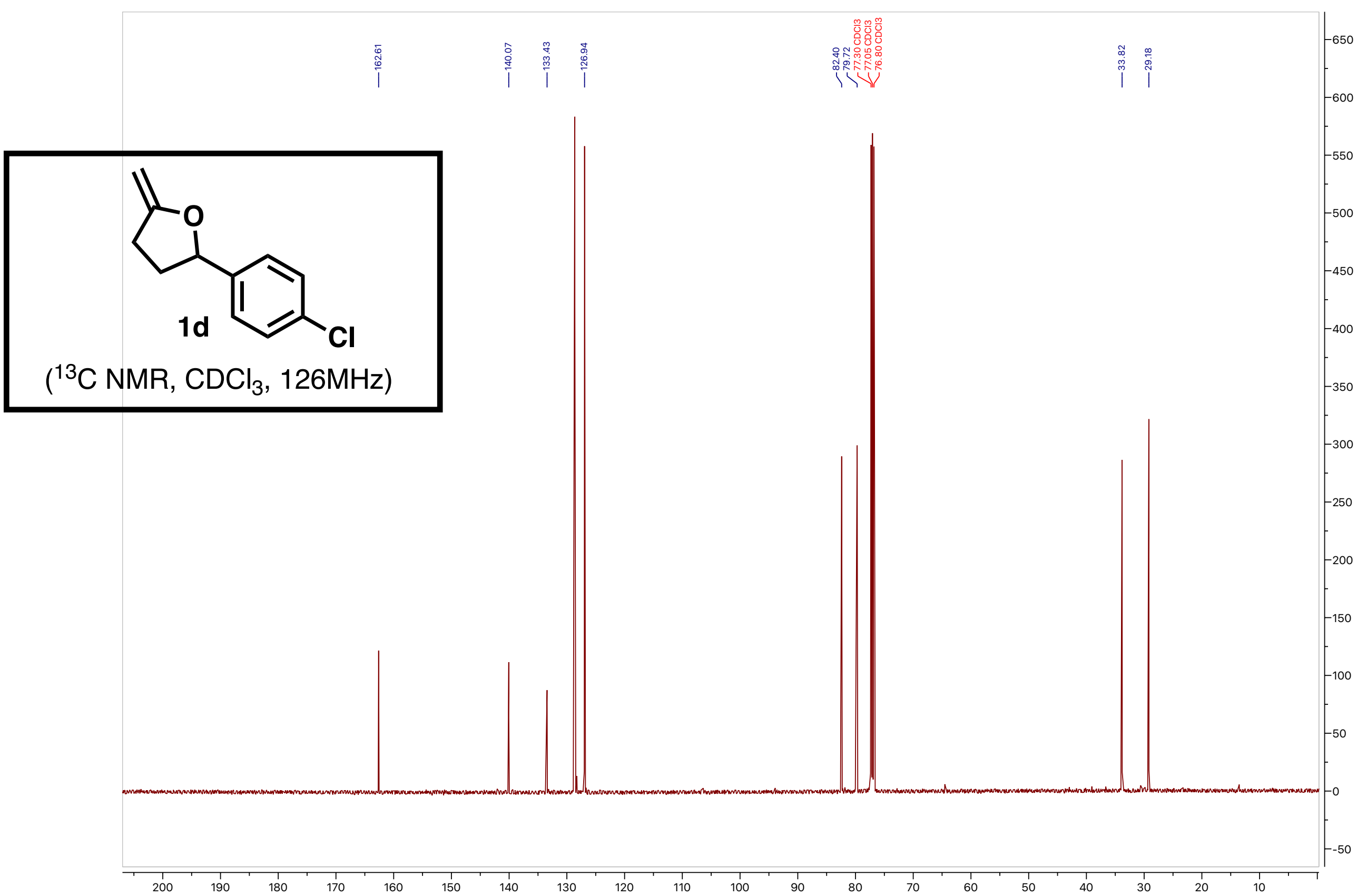
O
1d
Cl
(13C NMR, CDCl3, 126MHz)
162.61
140.07
133.43
126.94
82.40
79.72
77.30 CDCl3
77.05 CDCl3
76.80 CDCl3
33.82
29.18
f1 (ppm)

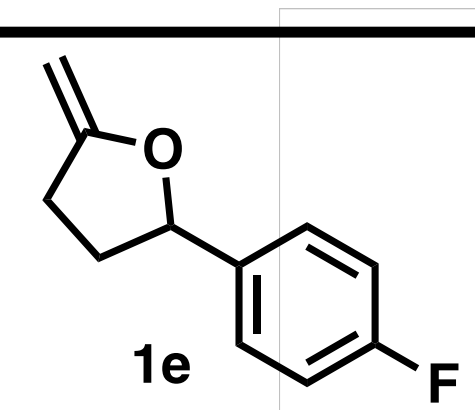

1e

(^{1}H NMR, $CDCl_3$, 500 MHz)
94.7% purity (DBU contaminant)

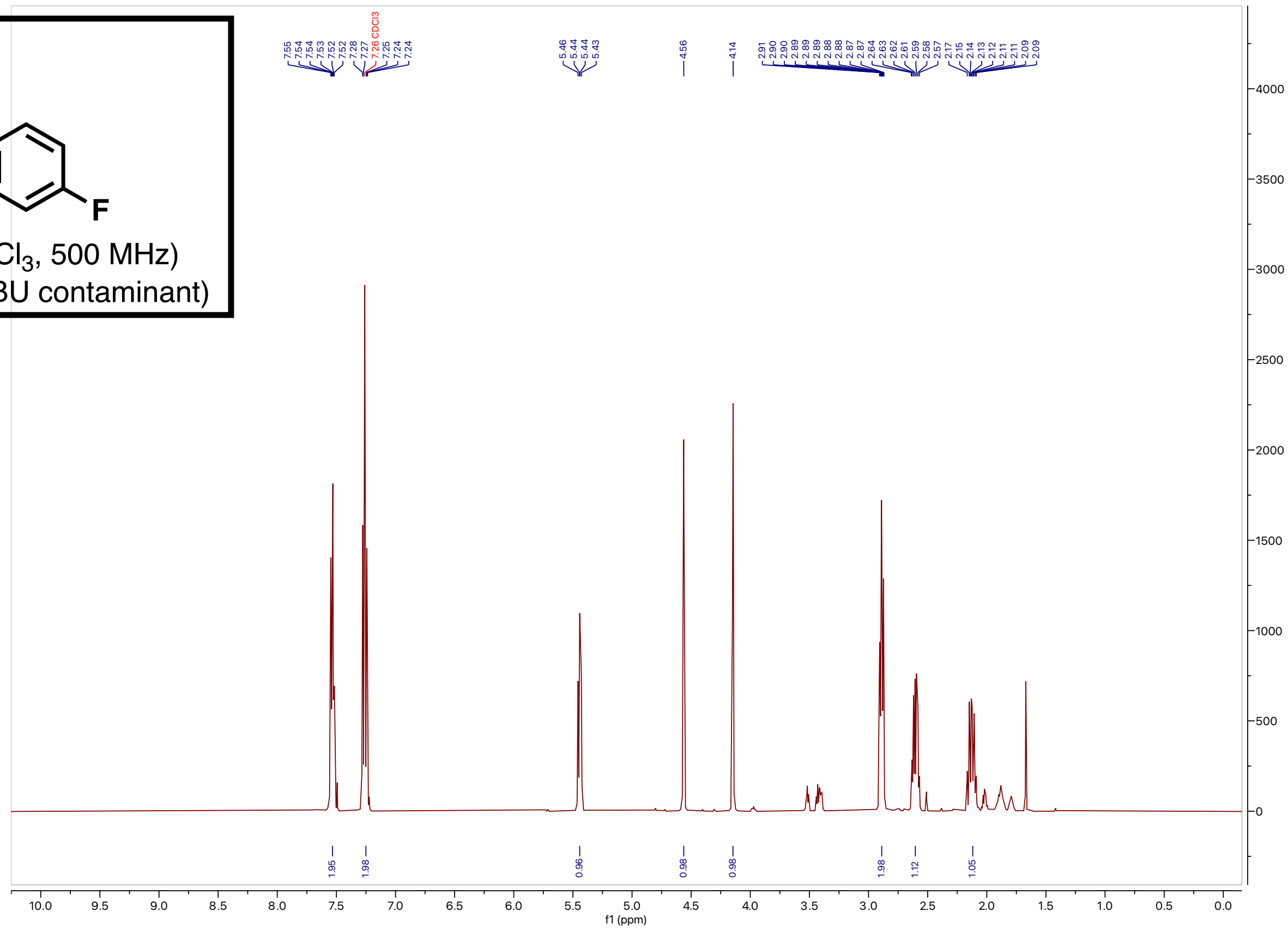

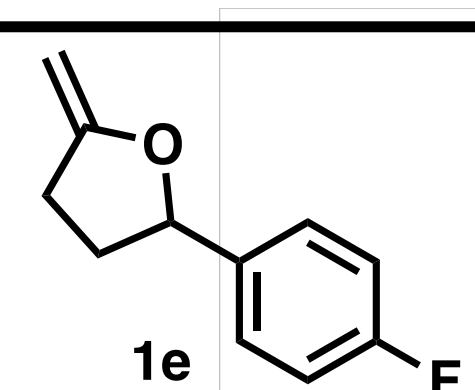

1e

(^{1}C NMR, $CDCl_3$, 126 MHz)
94.7% purity (DBU contaminant)

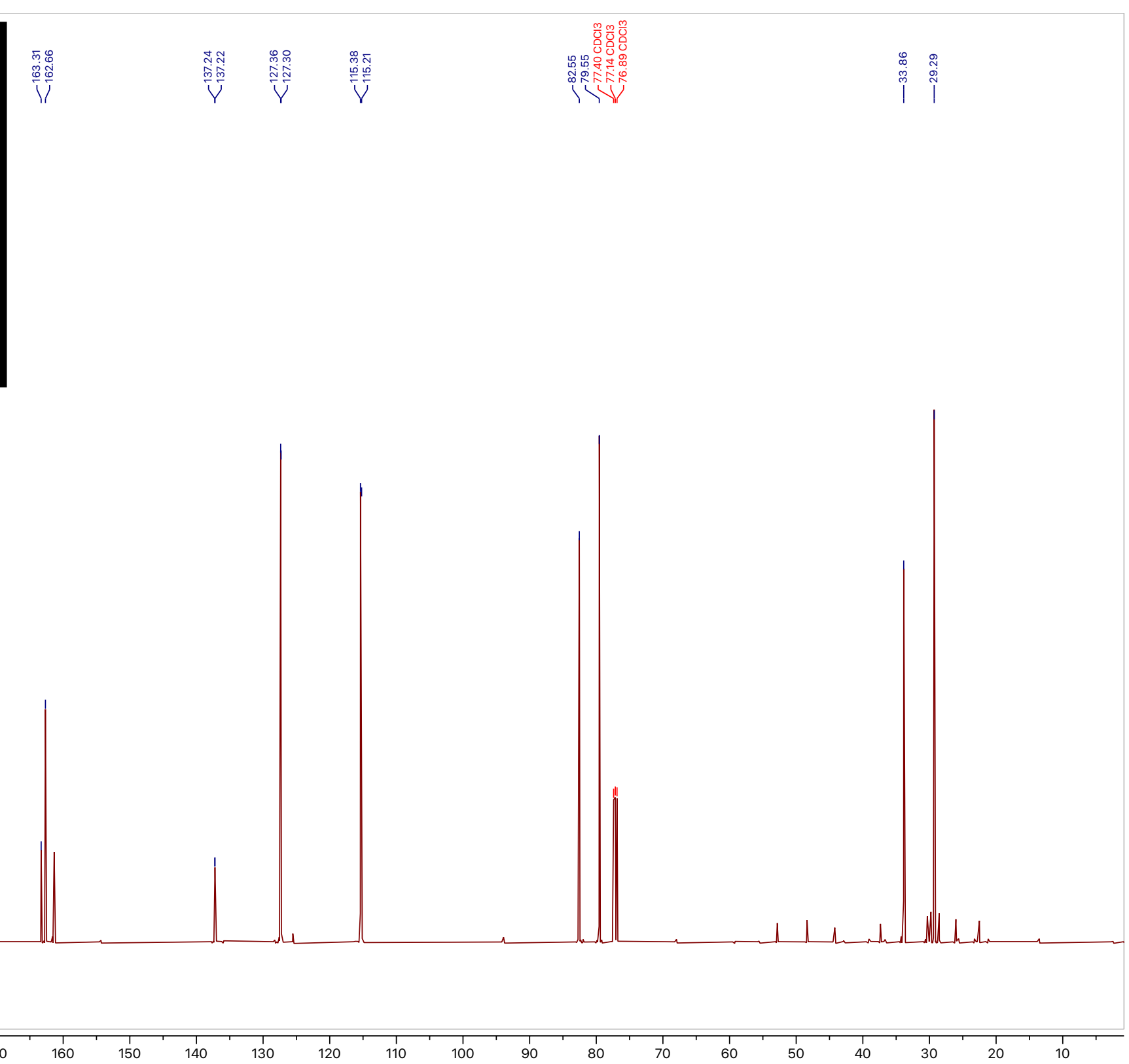

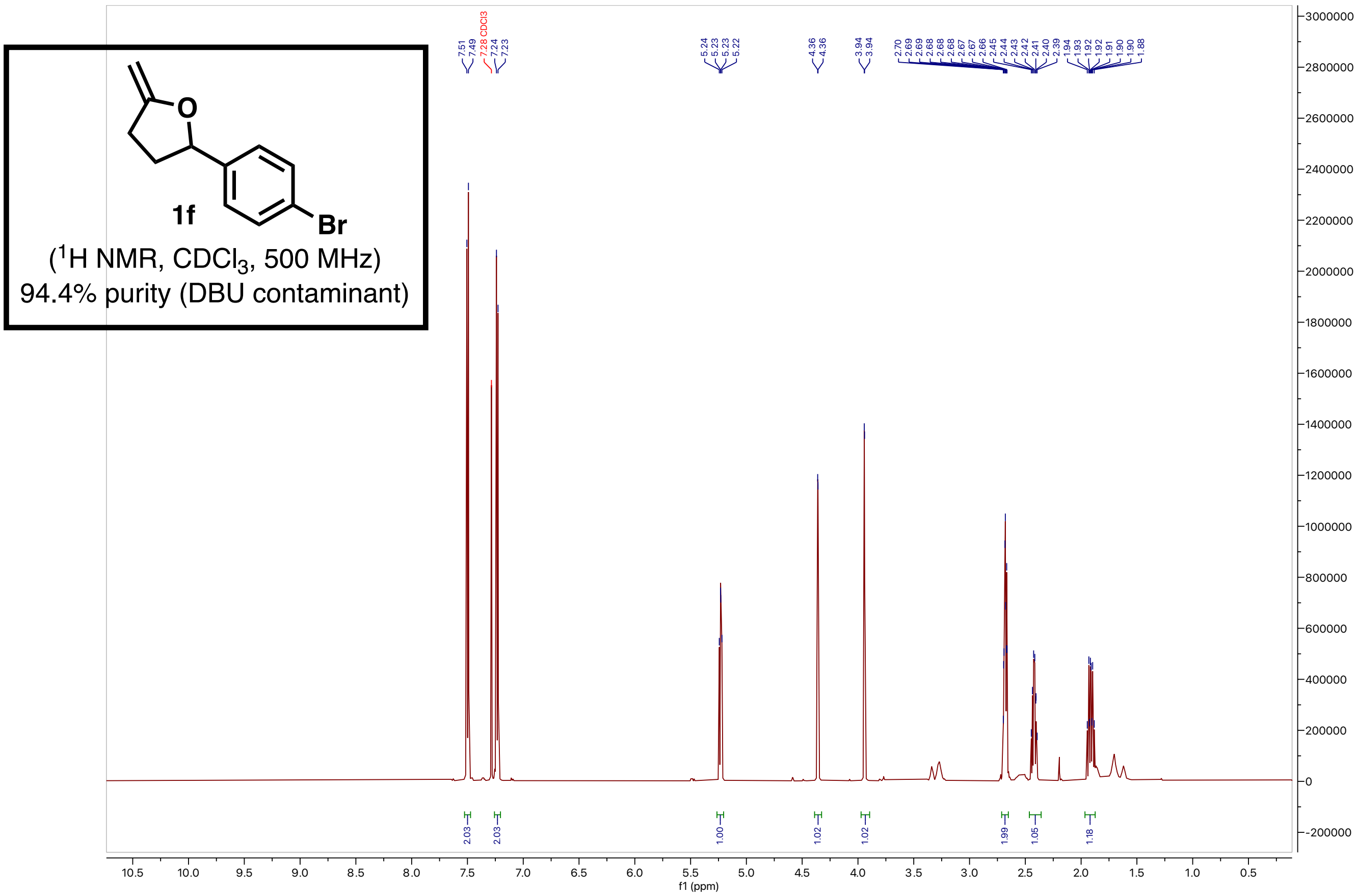
1f
(^{1}H NMR, CDCl$_3$, 500 MHz)
94.4% purity (DBU contaminant)
f1 (ppm)

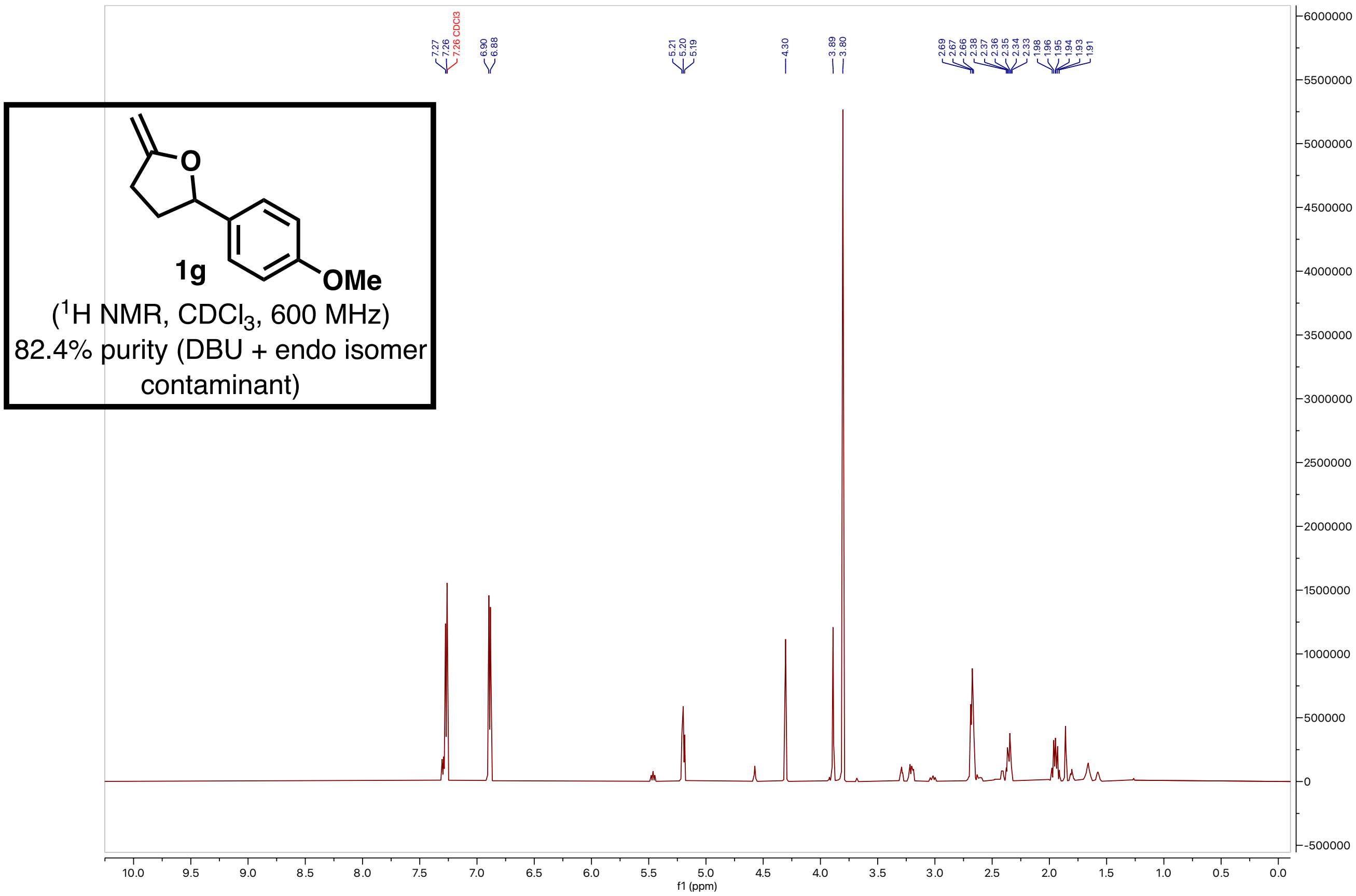
7.27
7.26
7.26 CDCl3
6.90
6.88
5.21
5.20
5.19
4.30
3.89
3.80
2.69
2.67
2.66
2.38
2.37
2.36
2.35
2.34
2.33
1.98
1.96
1.95
1.94
1.93
1.91
O
1g
OMe
(1H NMR, CDCl3, 600 MHz)
82.4% purity (DBU + endo isomer contaminant)
f1 (ppm)

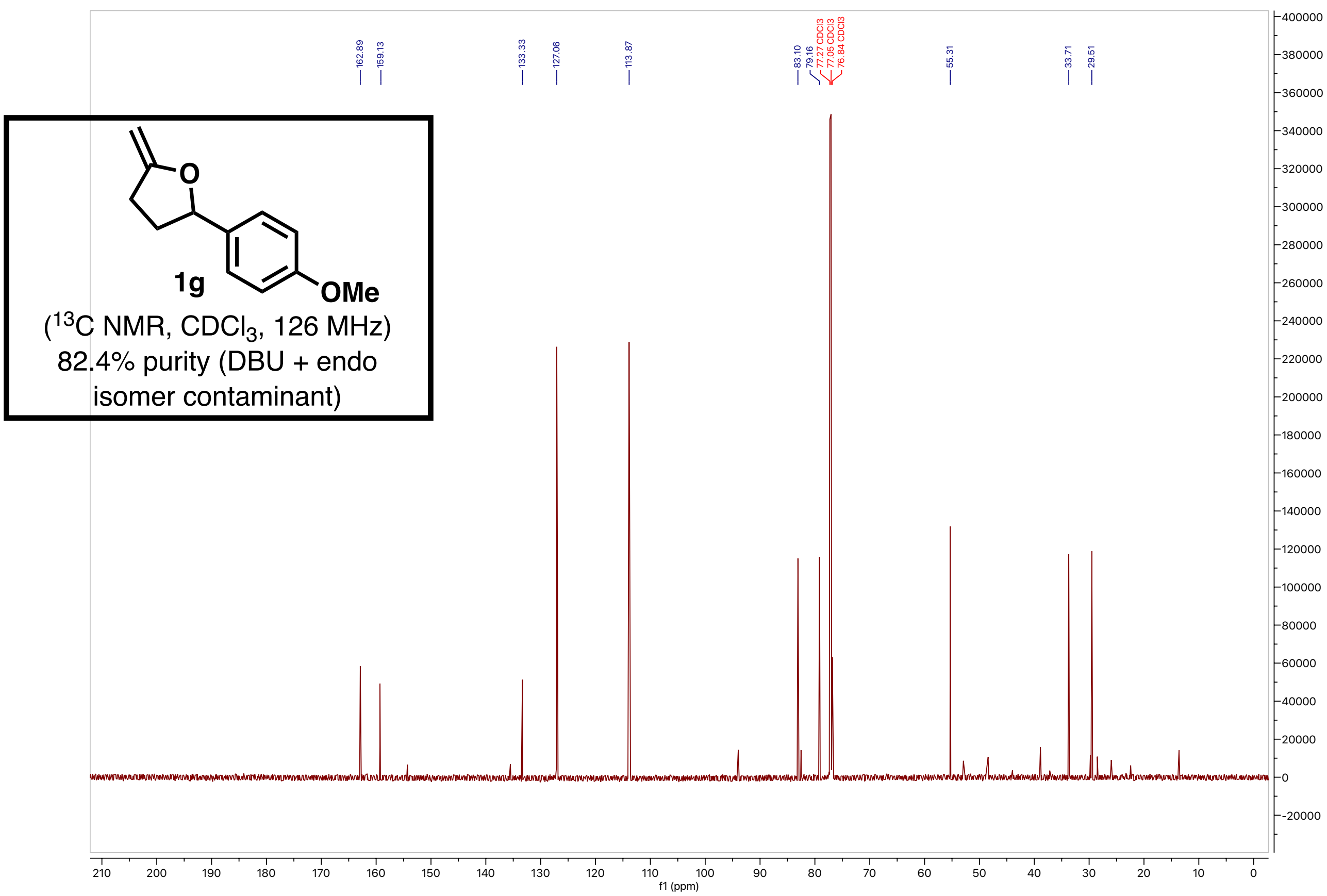
162.89
159.13
133.33
127.06
113.87
83.10
79.16
77.27 CDCl3
77.06 CDCl3
76.84 CDCl3
55.31
33.71
29.51
O
1g
OMe
(13C NMR, CDCl3, 126 MHz)
82.4% purity (DBU + endo isomer contaminant)
f1 (ppm)

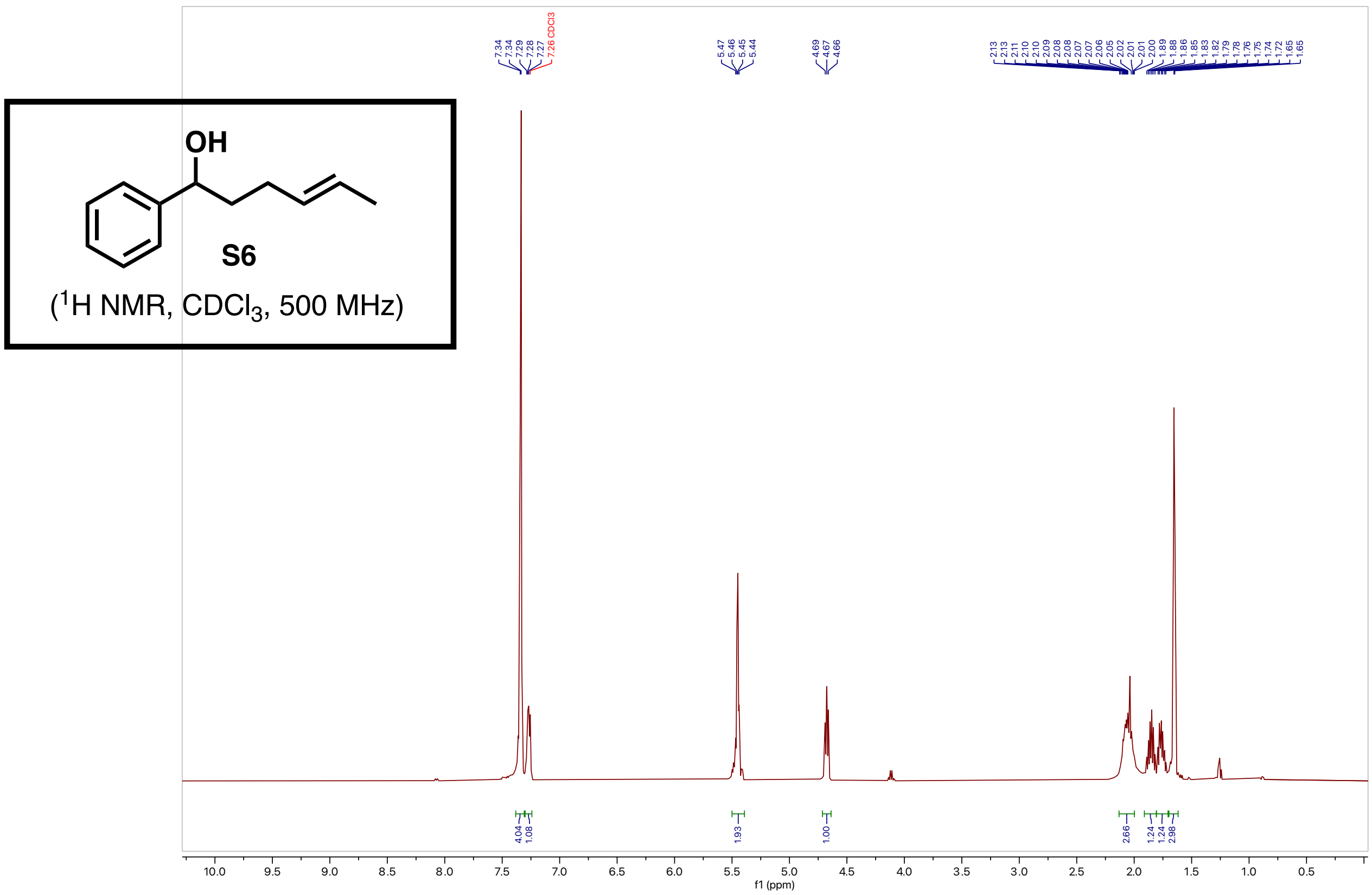
OH
S6
(1H NMR, CDCl3, 500 MHz)
7.34
7.34
7.29
7.28
7.27
7.26 CDCl3
5.47
5.46
5.45
5.44
4.69
4.67
4.66
4.04
1.08
1.93
1.00
2.66
1.24
1.24
2.98
f1 (ppm)

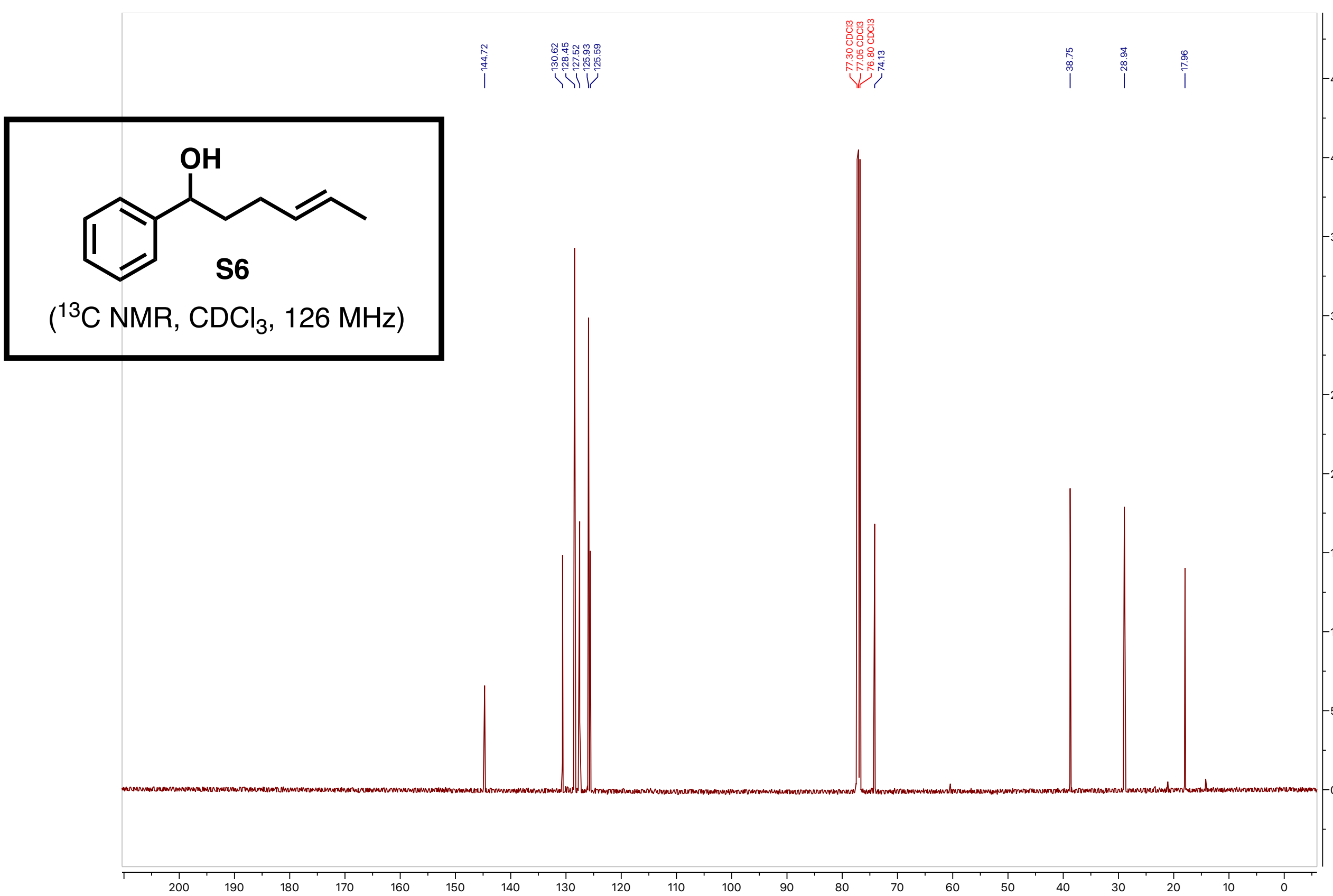
OH
S6
(13C NMR, CDCl3, 126 MHz)
144.72
130.62
128.45
127.52
125.93
125.59
77.30 CDCl3
77.05 CDCl3
76.80 CDCl3
74.13
38.75
28.94
17.96
f1 (ppm)

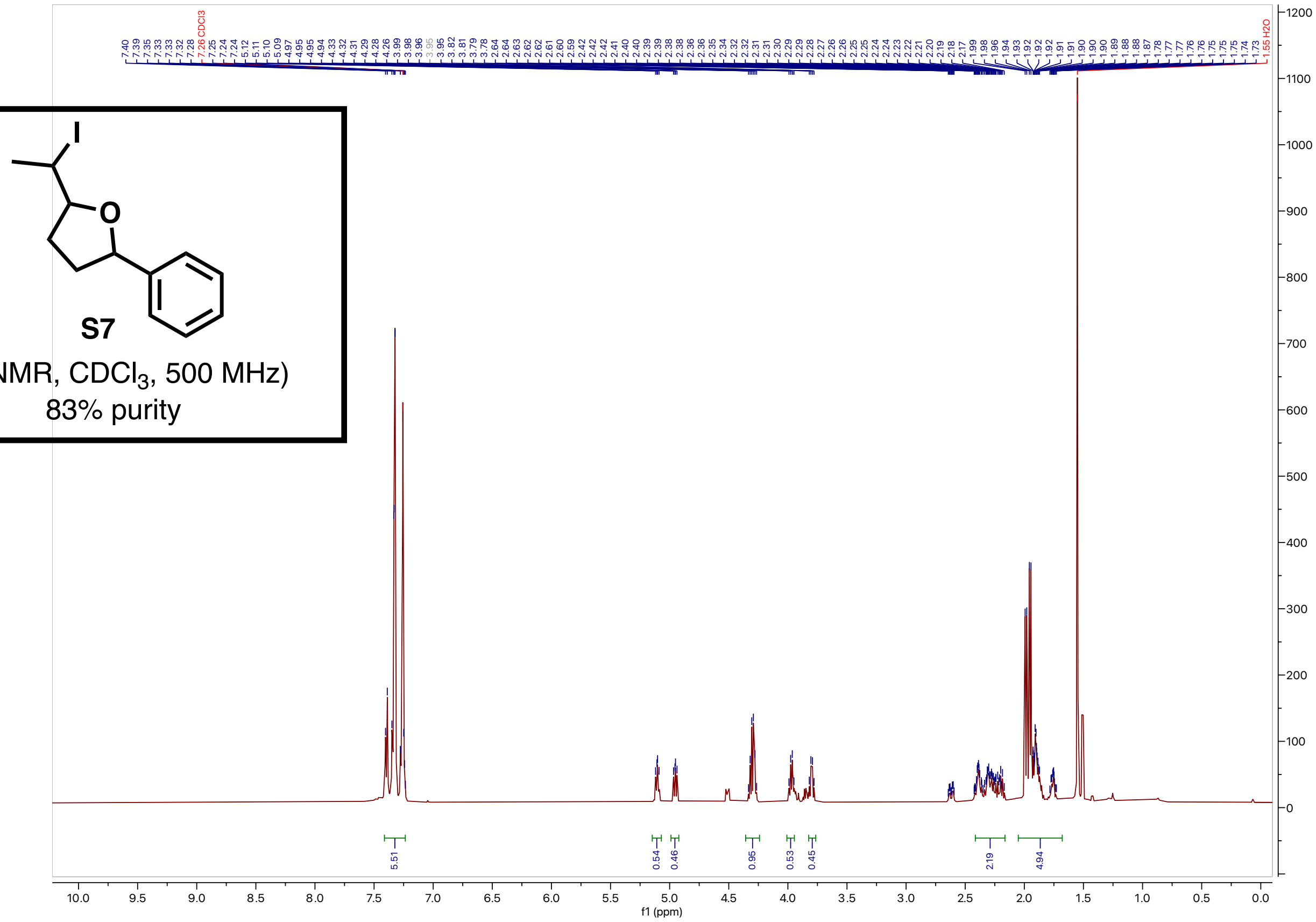

S7

(^{1}H NMR, $CDCl_3$, 500 MHz)
83% purity

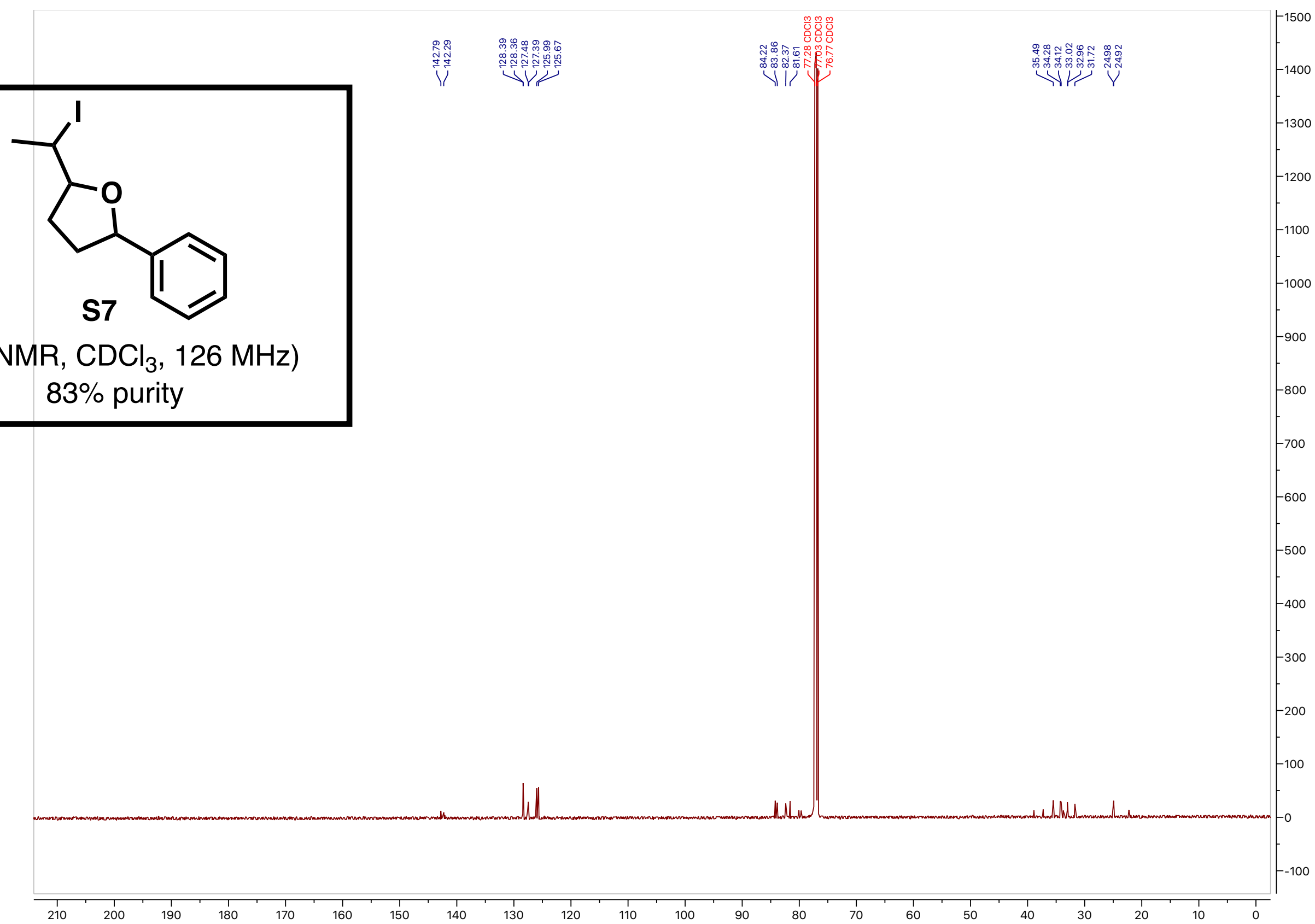

S7

(^{13}C NMR, $CDCl_3$, 126 MHz)
83% purity

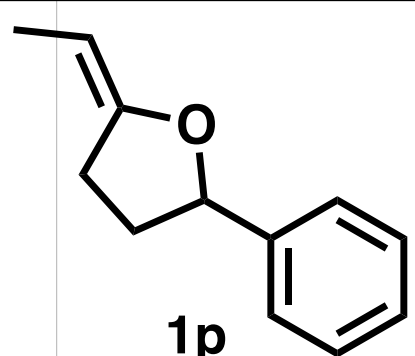

1p

(^{1}H NMR, $CDCl_3$, 500 MHz)
88% purity (DBU contaminant)

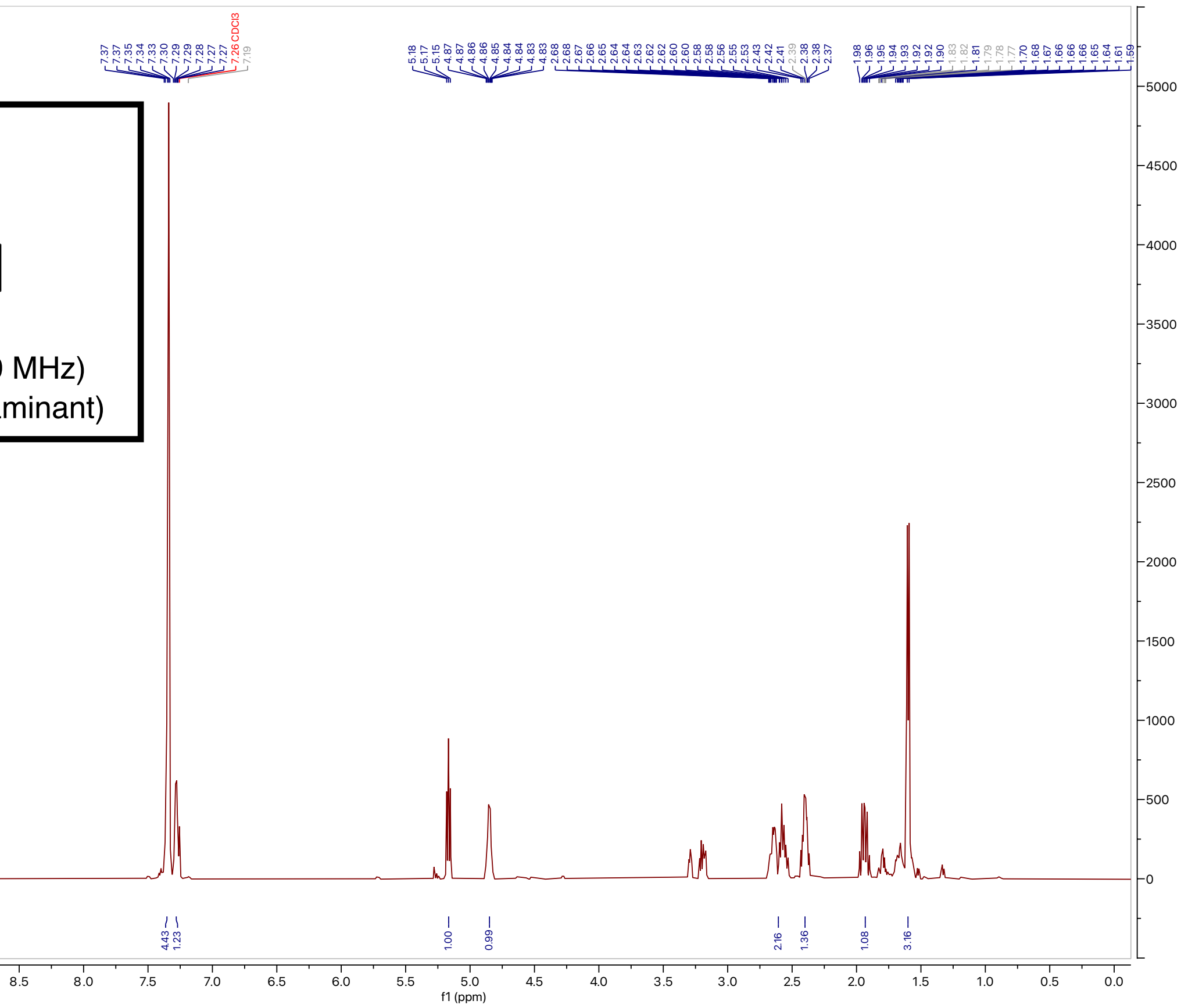

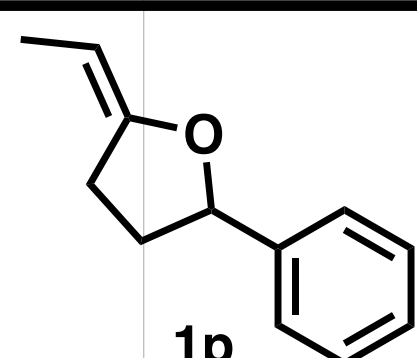

1p

(^{13}C NMR, $CDCl_3$, 126 MHz)
88% purity (DBU contaminant)

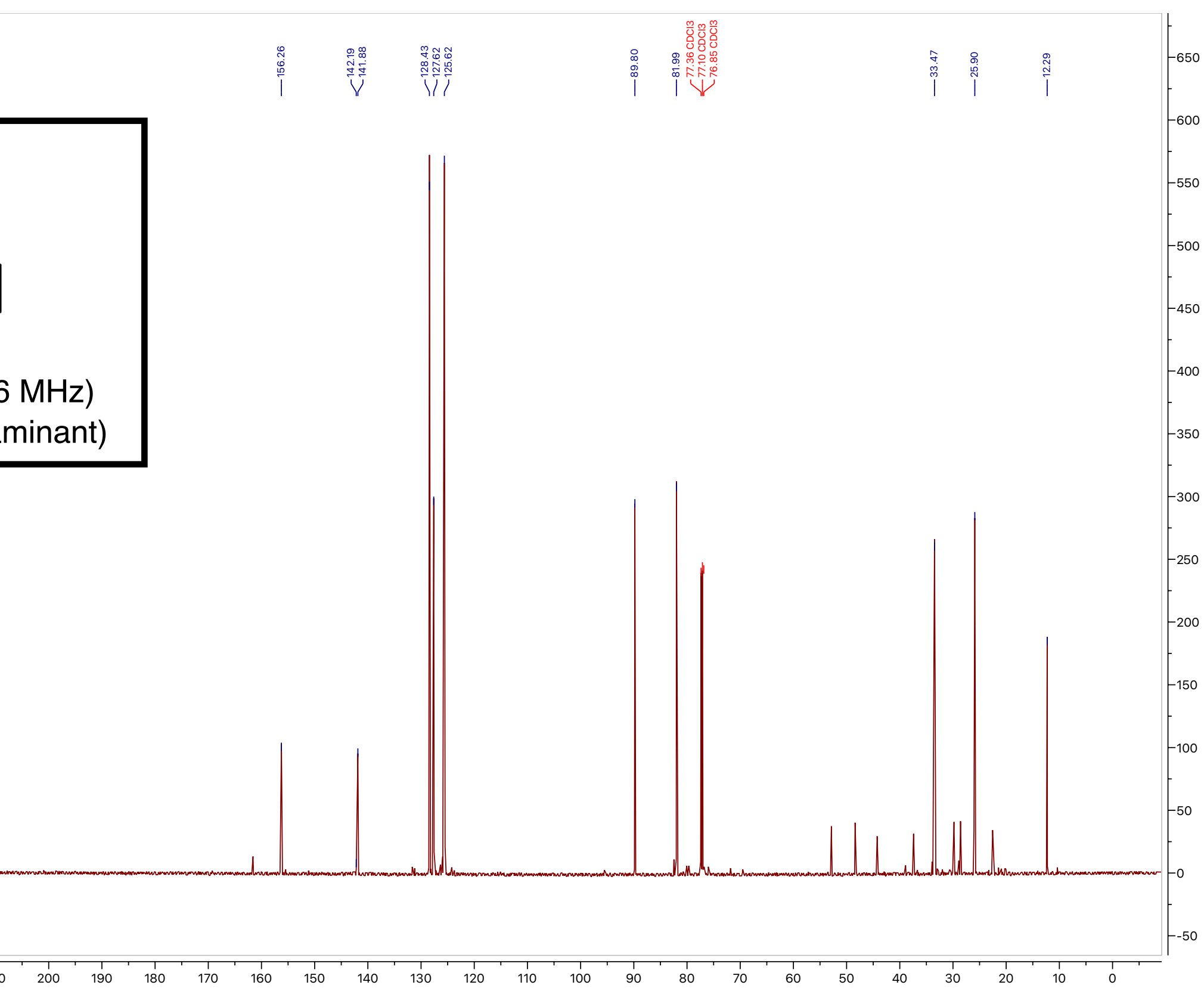

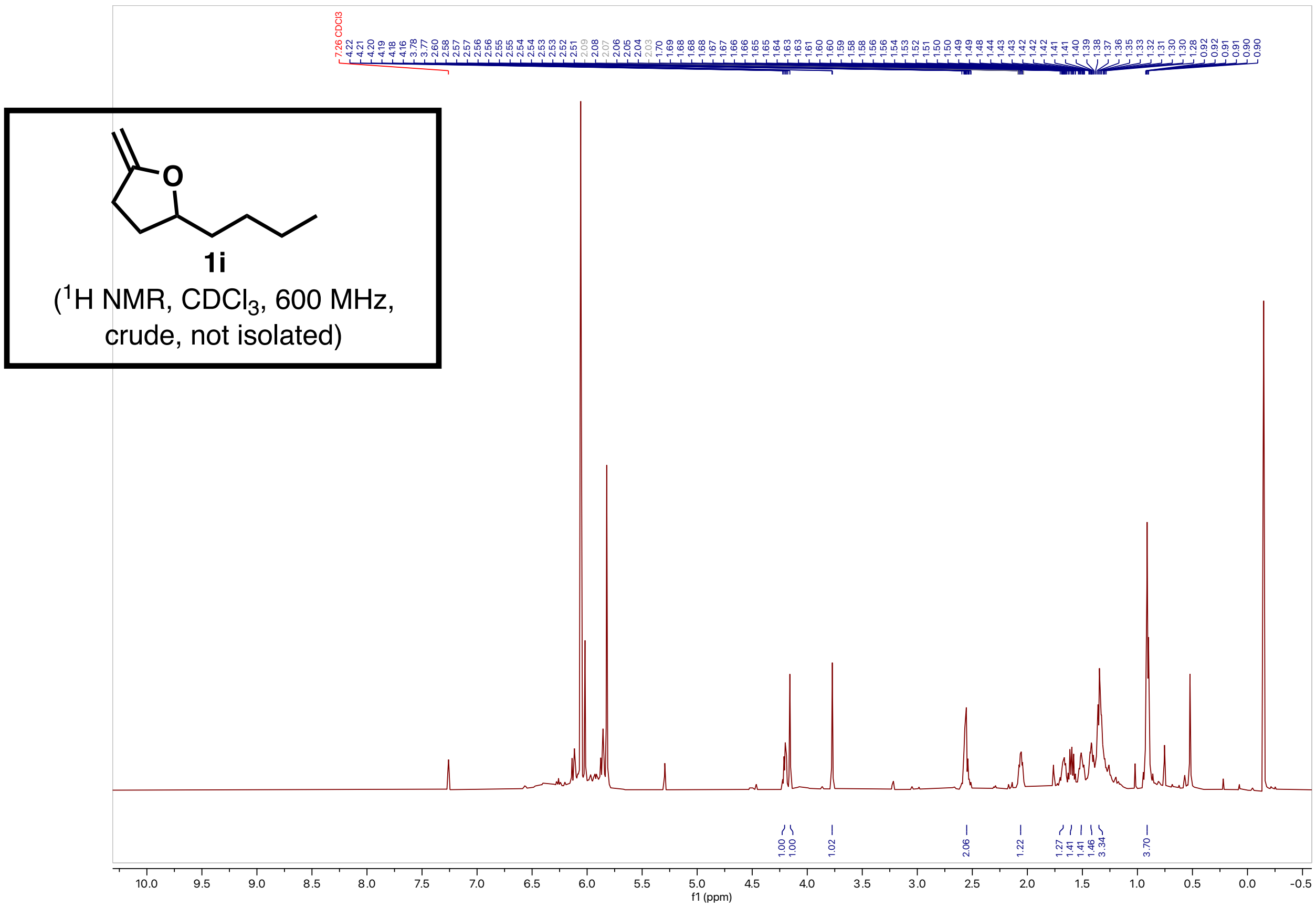

1i
(^{1}H NMR, $CDCl_3$, 600 MHz, crude, not isolated)

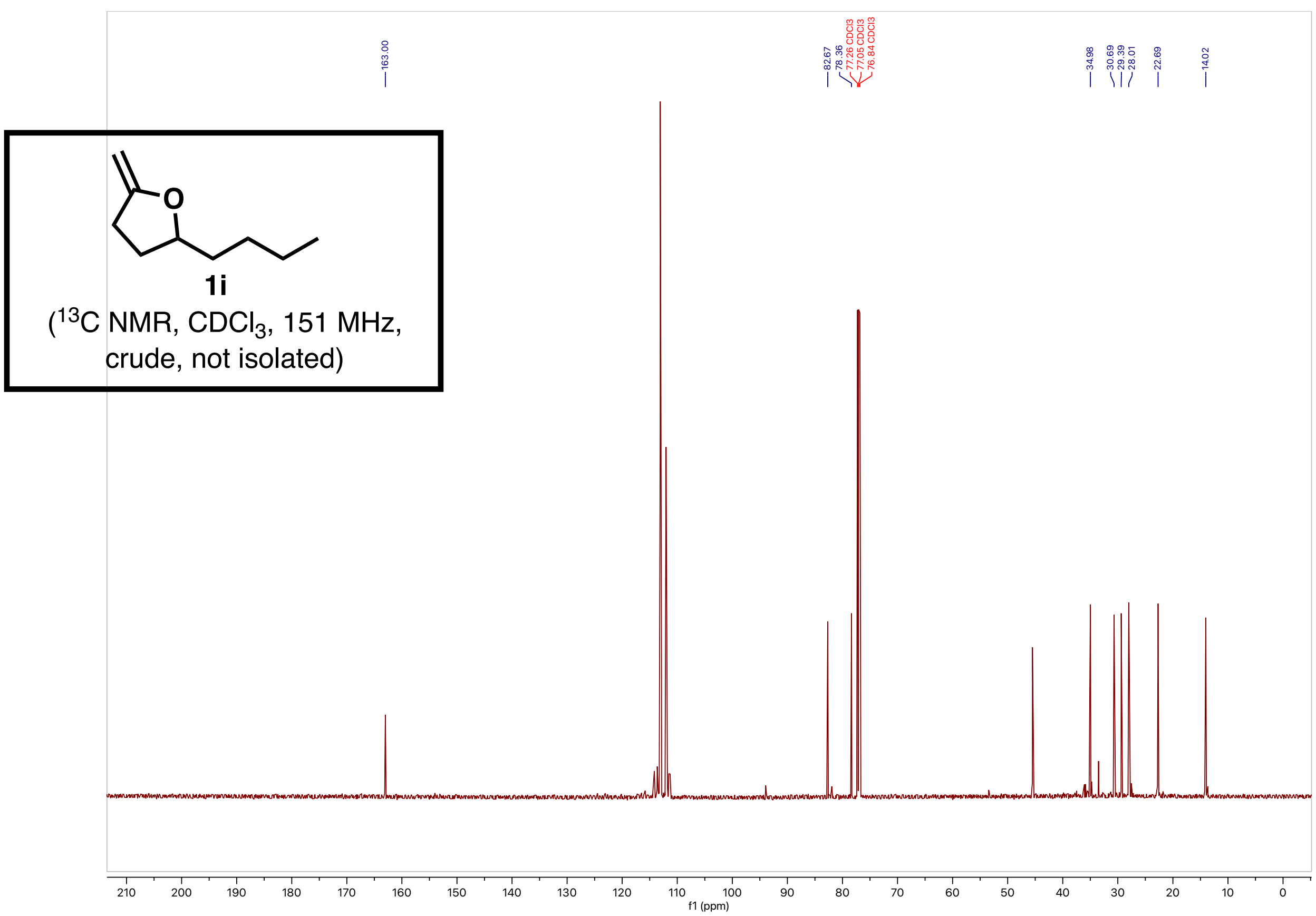

1i
(^{13}C NMR, $CDCl_3$, 151 MHz, crude, not isolated)

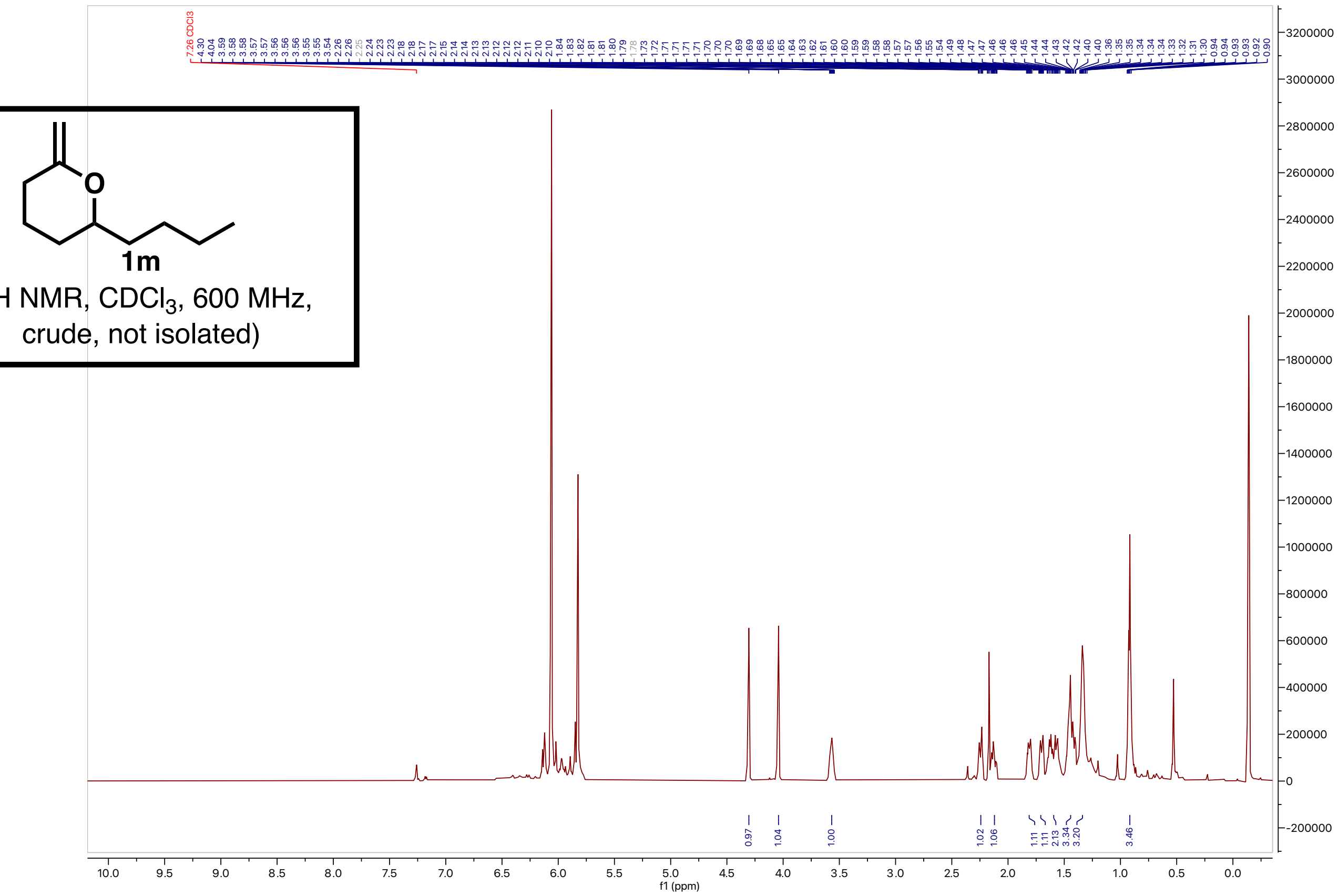

1m

(^{1}H NMR, $CDCl_3$, 600 MHz, crude, not isolated)

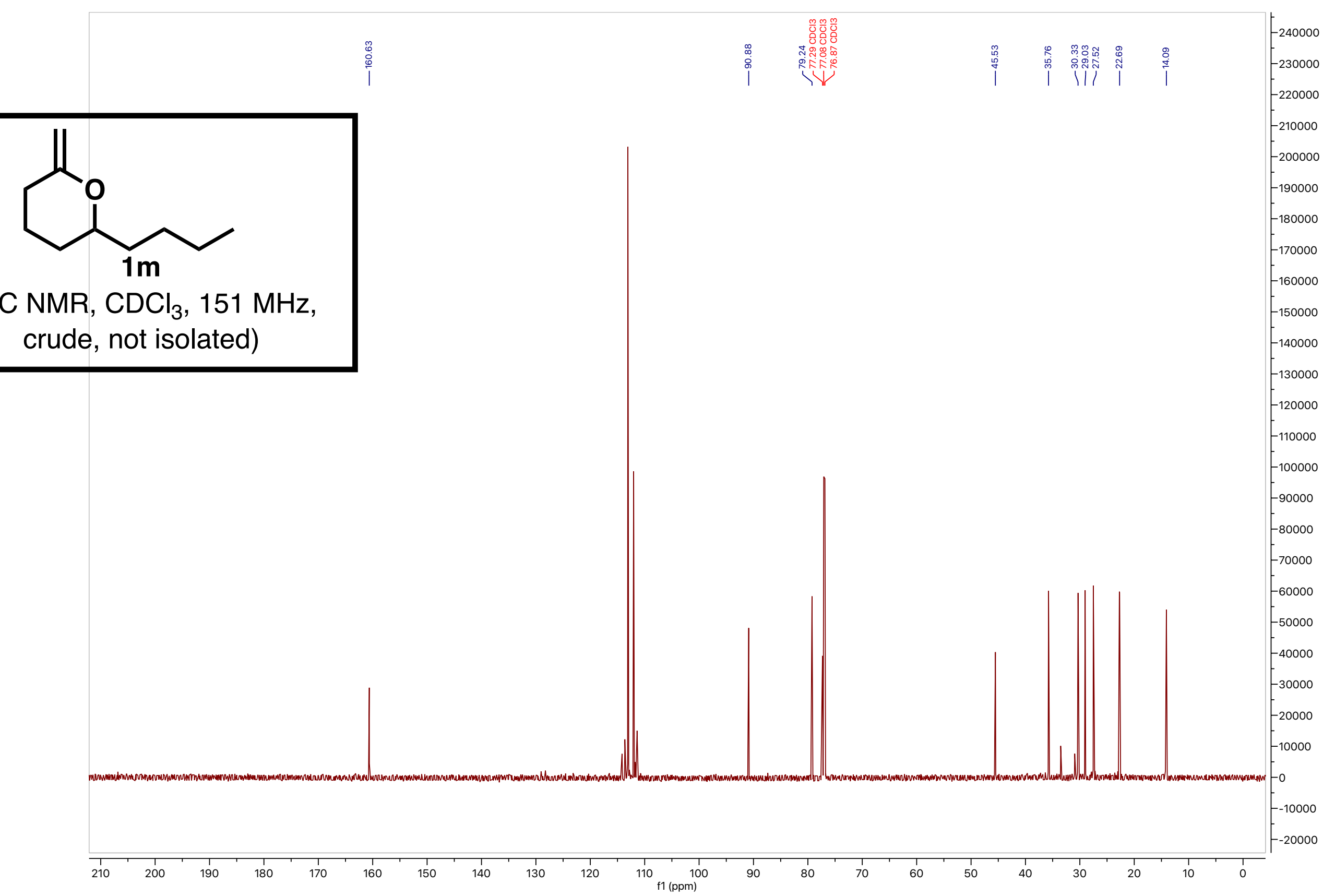

1m

(^{13}C NMR, $CDCl_3$, 151 MHz, crude, not isolated)

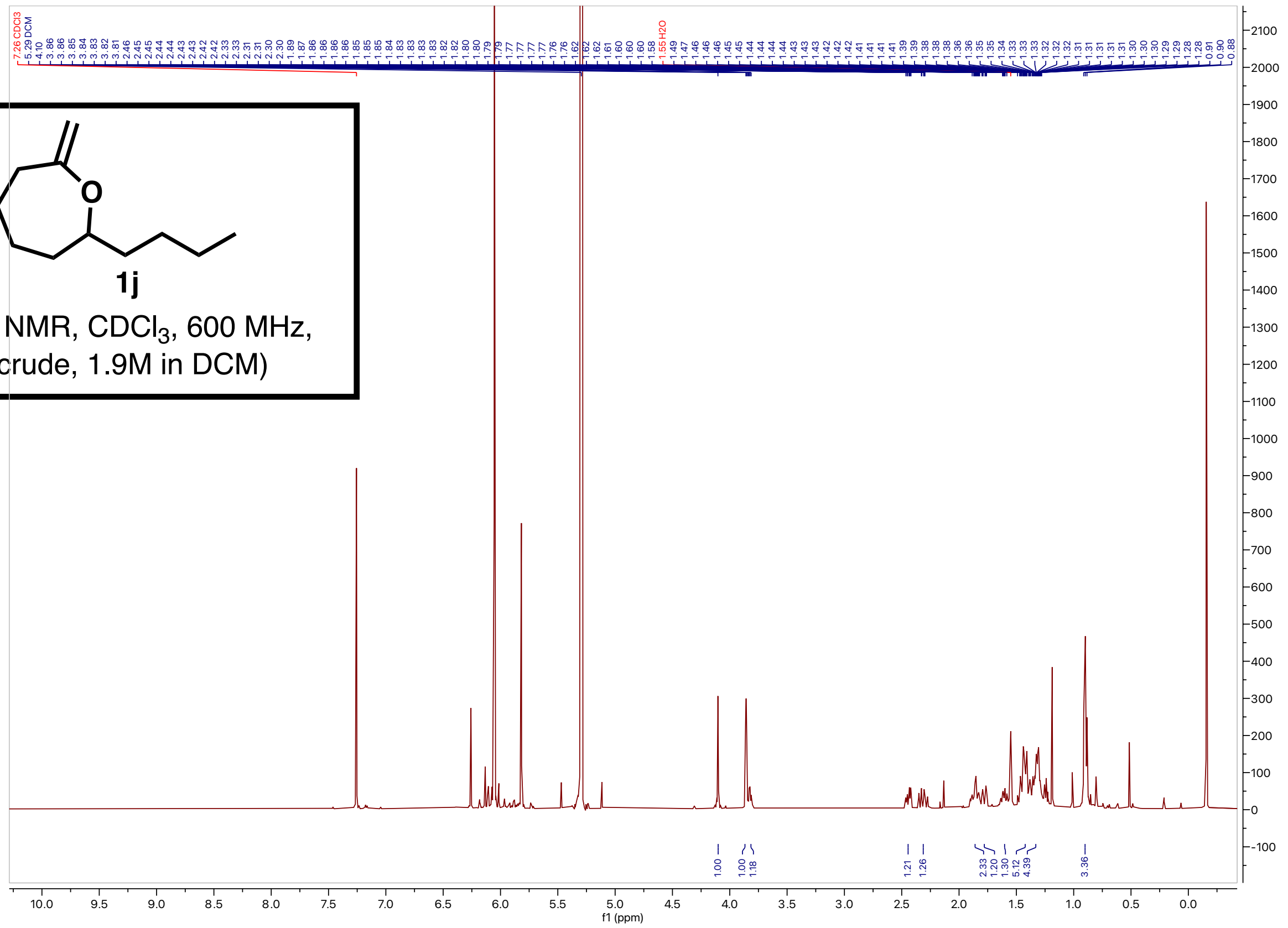

1j

(^{1}H NMR, $CDCl_3$, 600 MHz, crude, 1.9M in DCM)

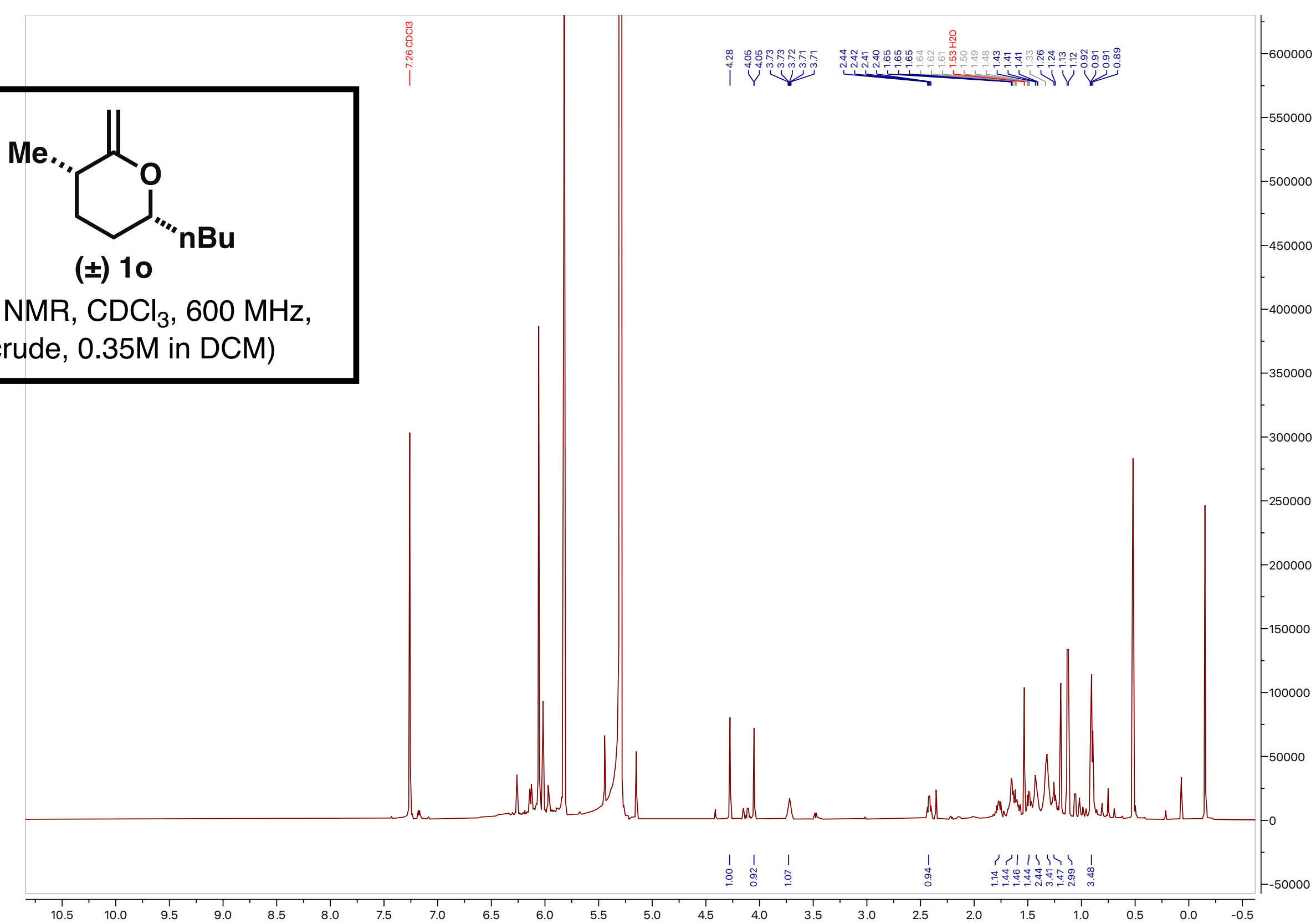

(±) 1o

(^{1}H NMR, $CDCl_3$, 600 MHz, crude, 0.35M in DCM)

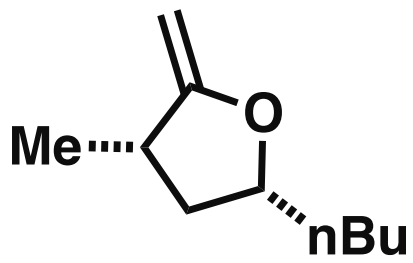

(±) 1n

(^{1}H NMR, $CDCl_3$, 600 MHz, crude, not isolated)

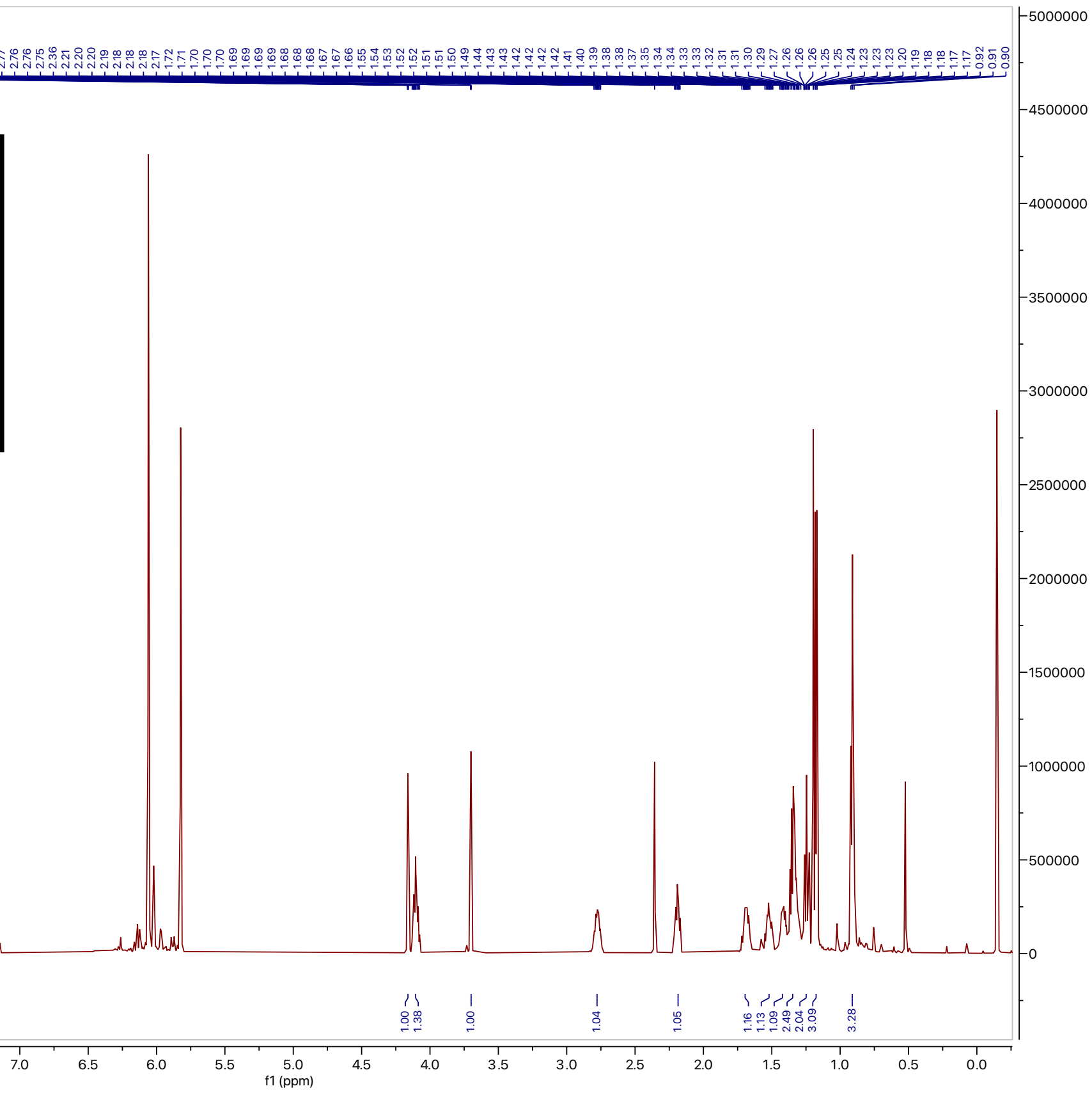

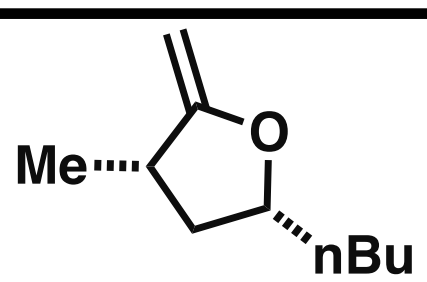

(±) 1n

(^{13}C NMR, $CDCl_3$, 151 MHz, crude, not isolated)

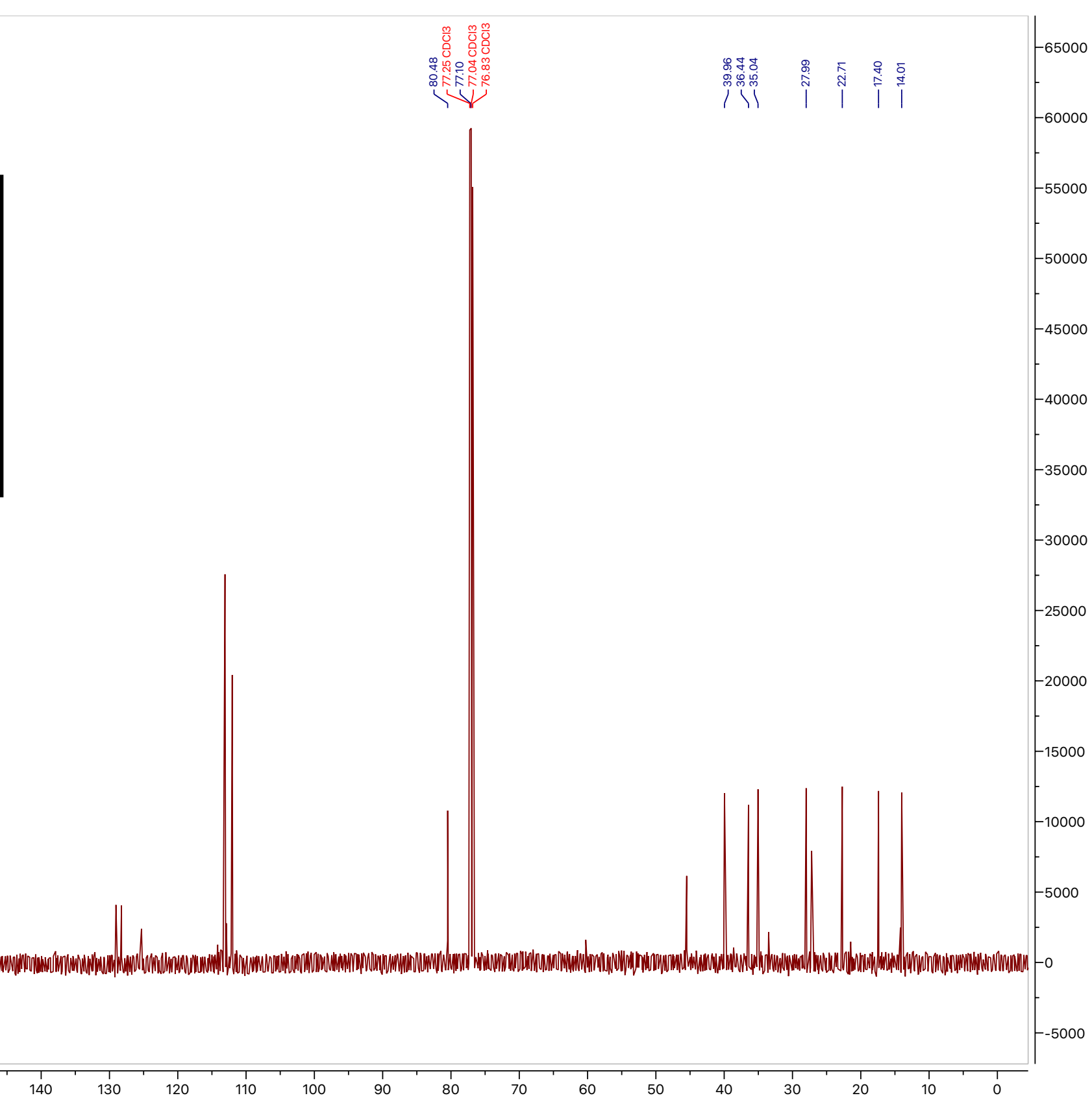

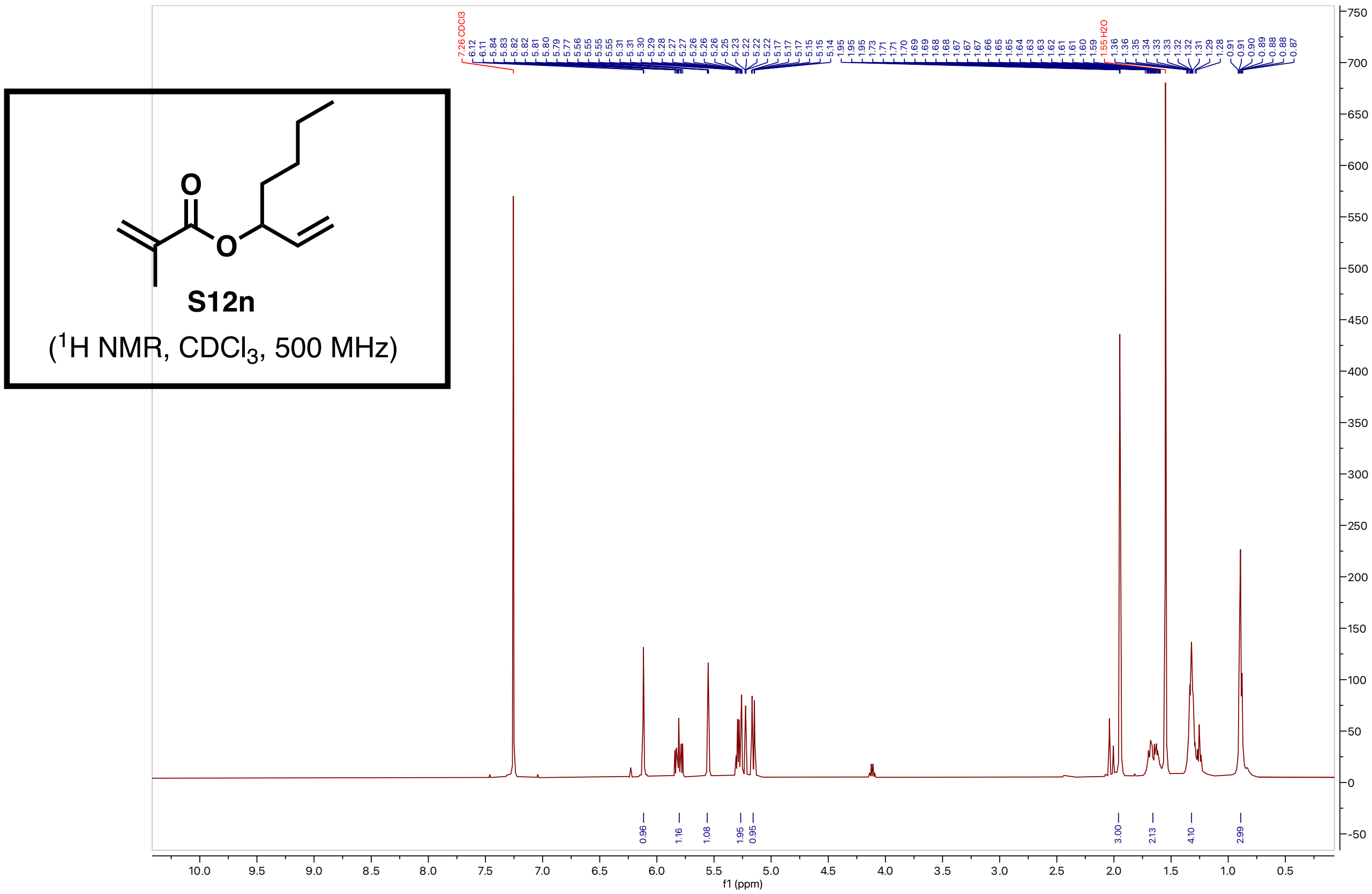
S12n
(1H NMR, CDCl3, 500 MHz)
f1 (ppm)

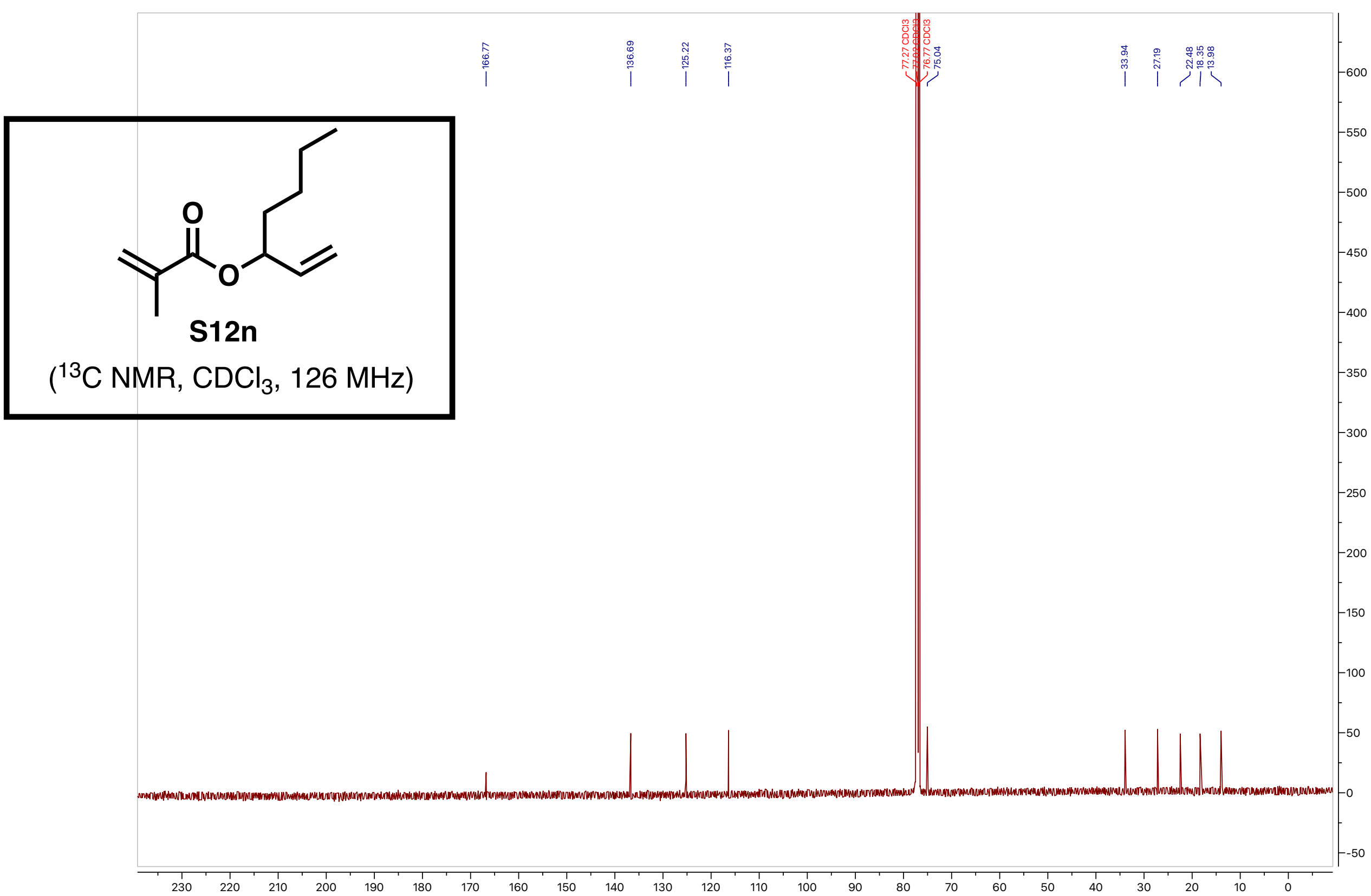
S12n
(13C NMR, CDCl3, 126 MHz)
166.77
136.69
125.22
116.37
75.04
33.94
27.19
22.48
18.35
13.98
f1 (ppm)

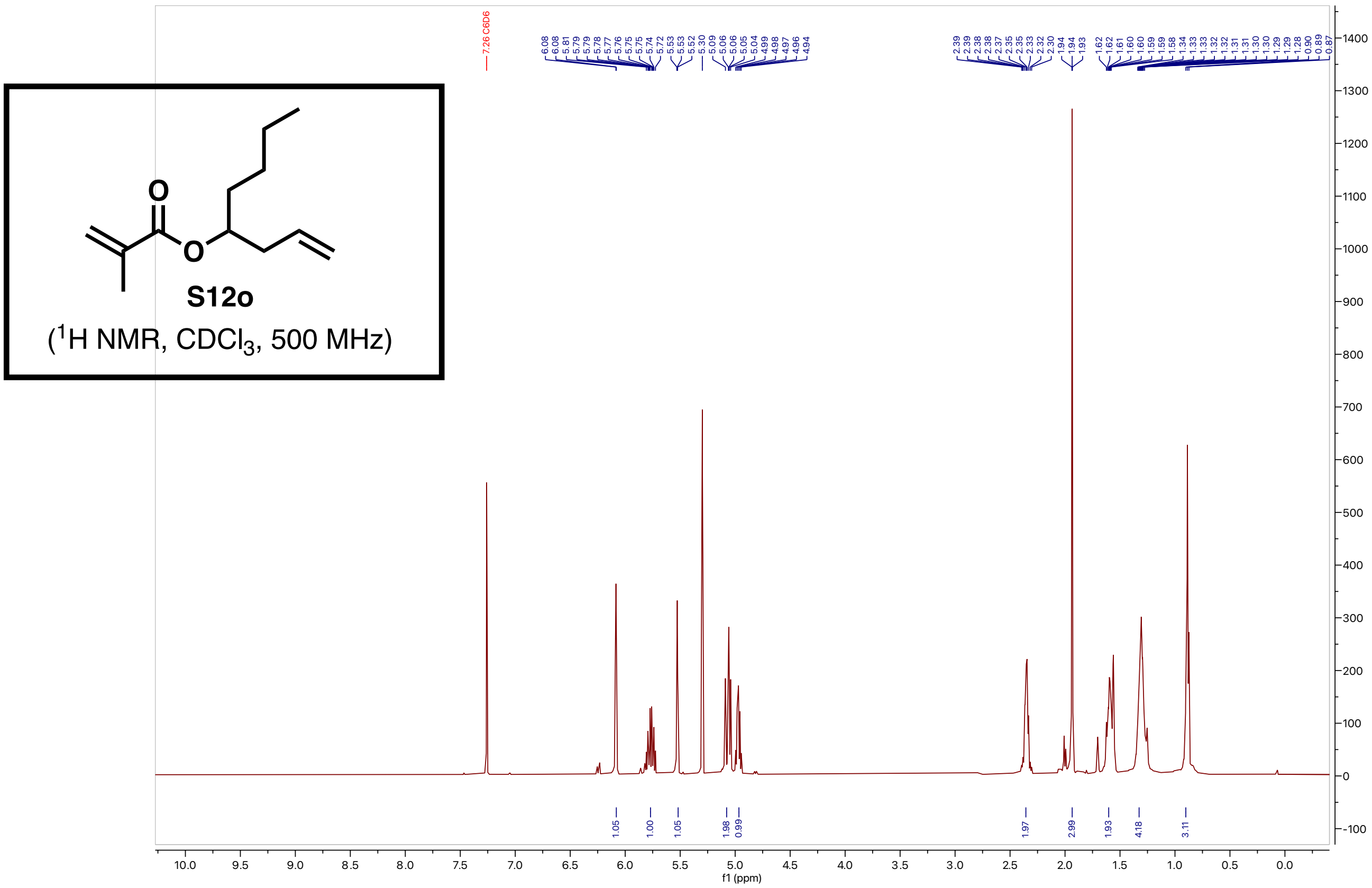
S12o
(1H NMR, CDCl3, 500 MHz)
7.26 C6D6
6.08
6.08
5.81
5.79
5.79
5.78
5.77
5.76
5.75
5.74
5.72
5.53
5.53
5.52
5.30
5.09
5.06
5.06
5.05
5.04
4.99
4.98
4.97
4.96
4.94
2.39
2.39
2.38
2.38
2.37
2.35
2.35
2.33
2.32
2.30
1.94
1.94
1.93
1.62
1.62
1.61
1.60
1.60
1.59
1.59
1.58
1.34
1.33
1.33
1.32
1.32
1.31
1.31
1.30
1.30
1.29
1.29
1.28
0.90
0.89
0.87
1.05
1.00
1.05
1.98
0.99
1.97
2.99
1.93
4.18
3.11
f1 (ppm)

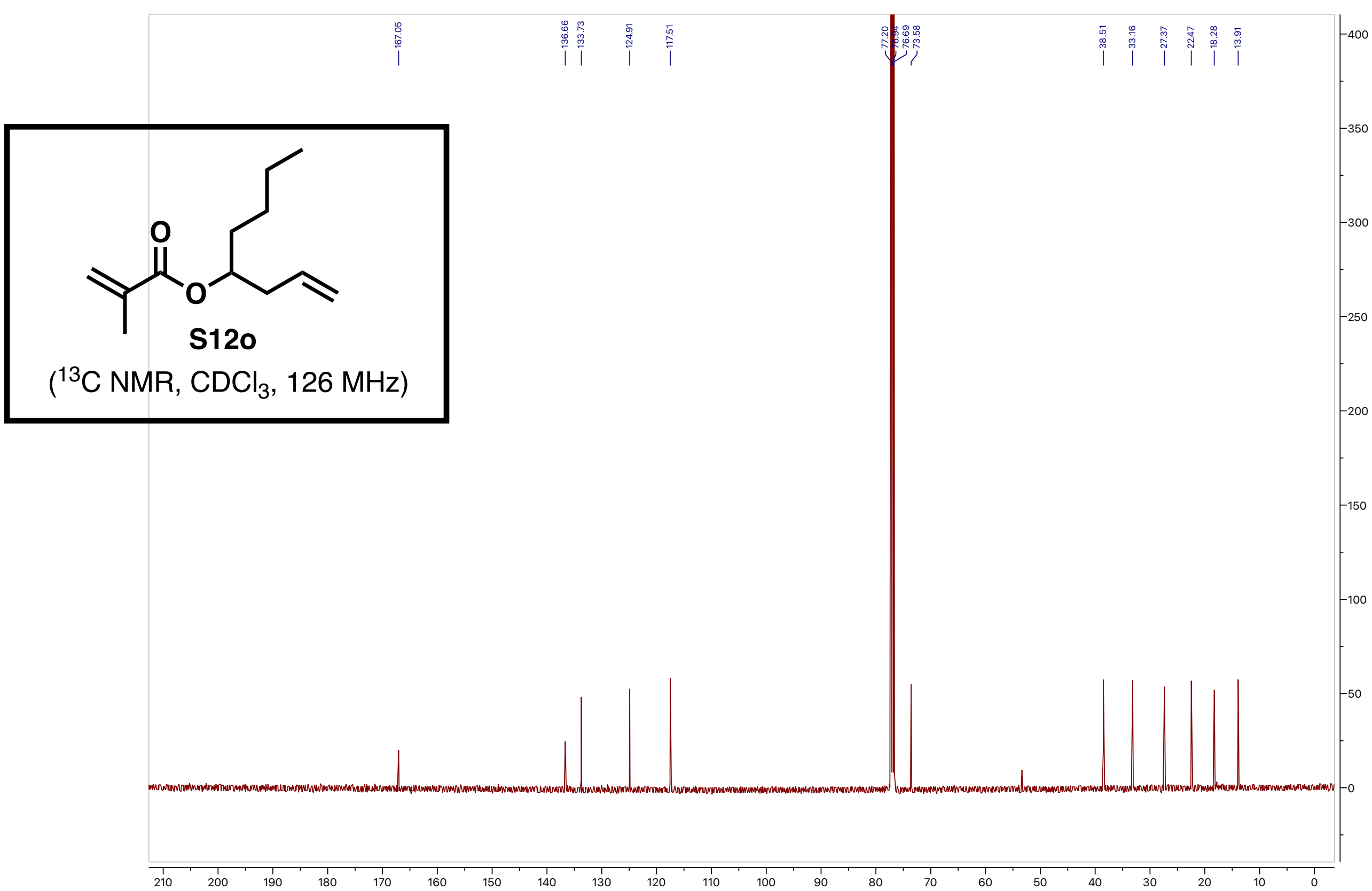
S12o
(13C NMR, CDCl3, 126 MHz)
167.05
136.66
133.73
124.91
117.51
77.20
76.94
76.69
73.58
38.51
33.16
27.37
22.47
18.28
13.91
f1 (ppm)

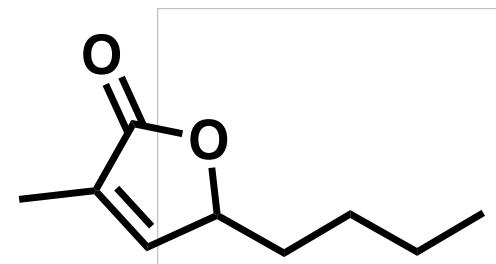

S13n

(^{1}H NMR, $CDCl_3$, 600 MHz)

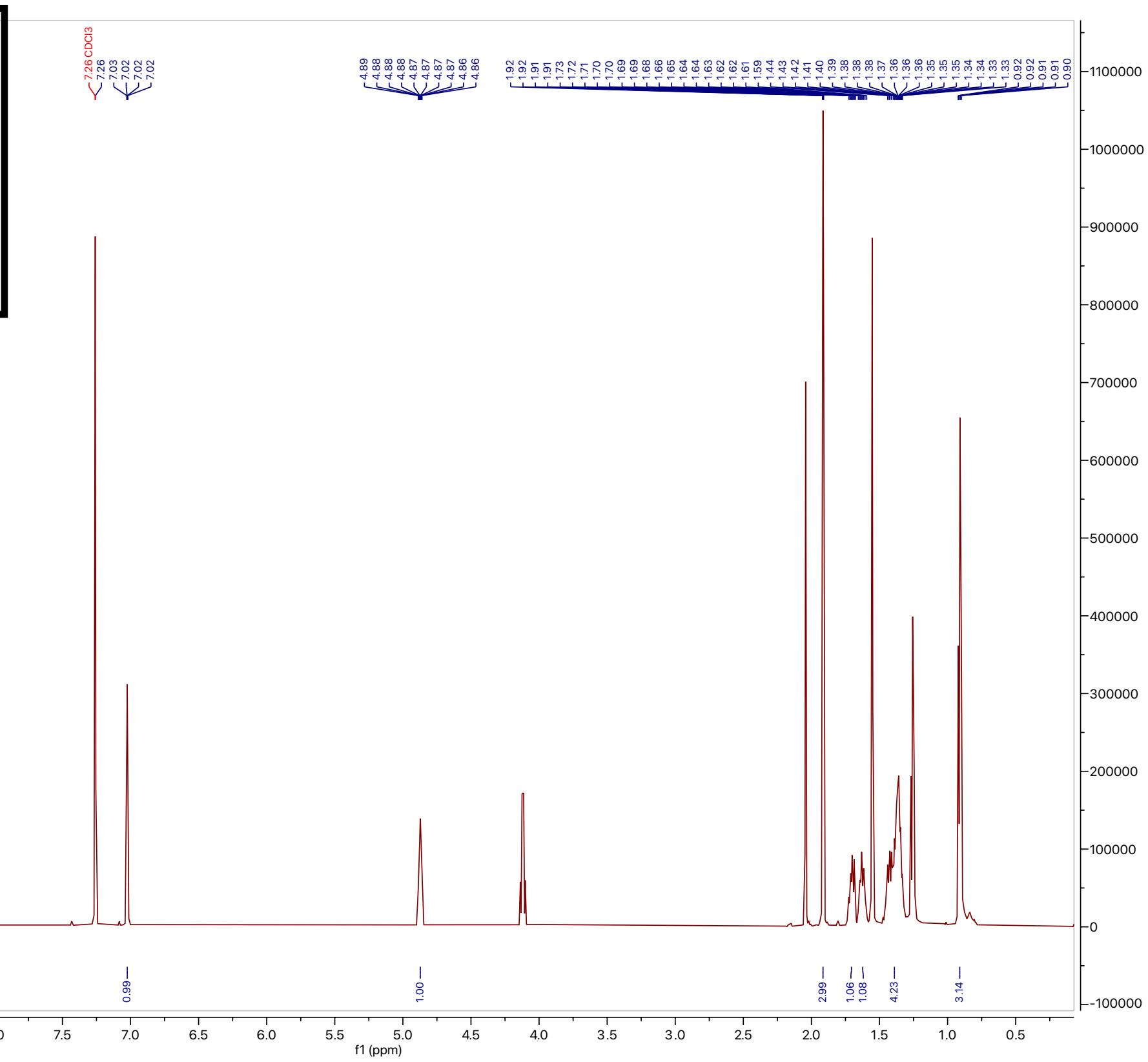

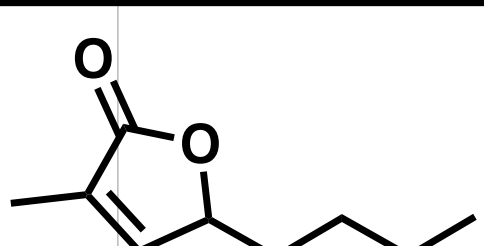

S13n

(^{13}C NMR, $CDCl_3$, 126 MHz)

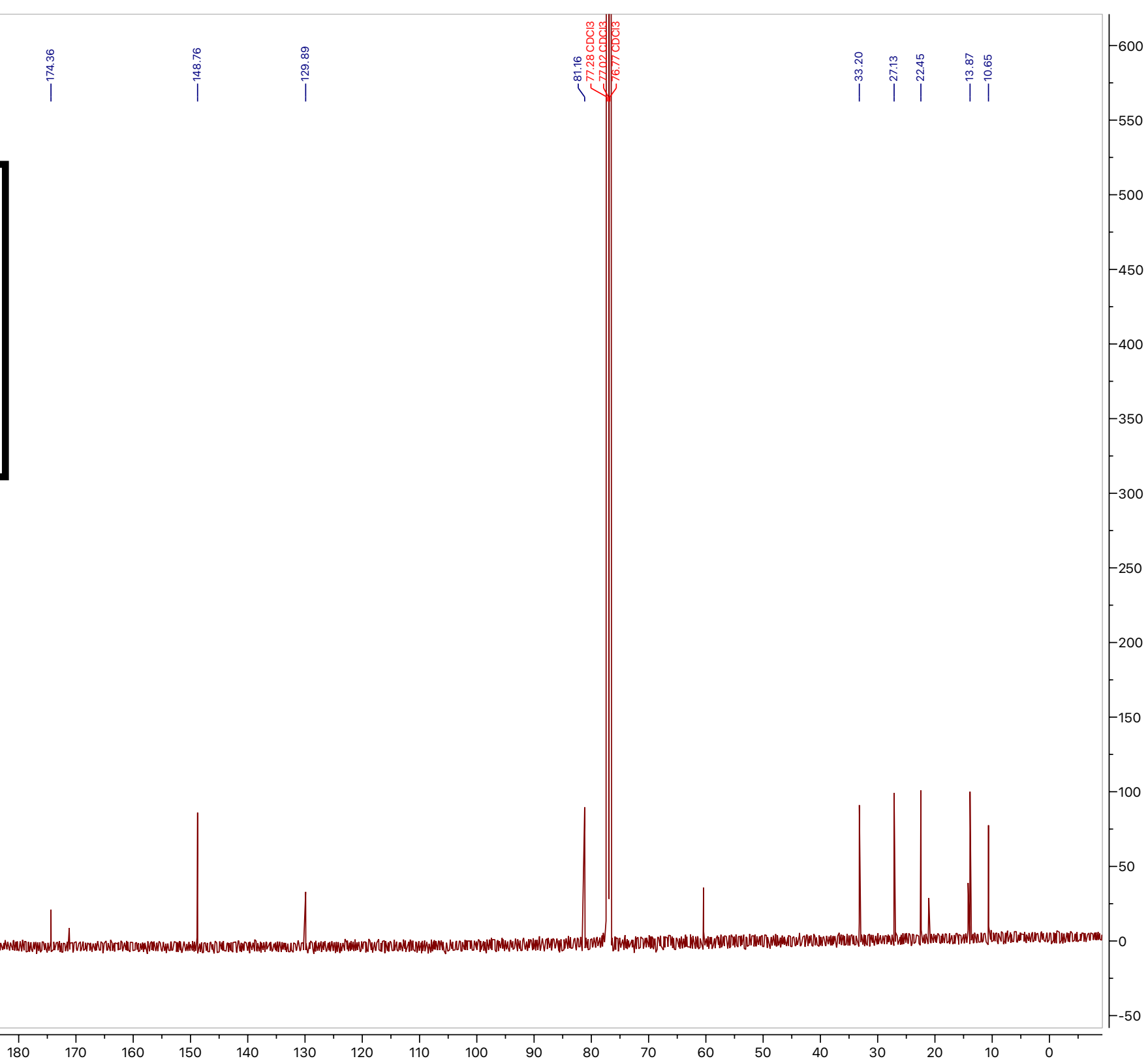

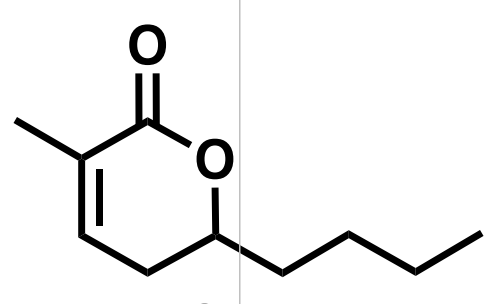

S13o

(^{1}H NMR, $CDCl_3$, 500 MHz)

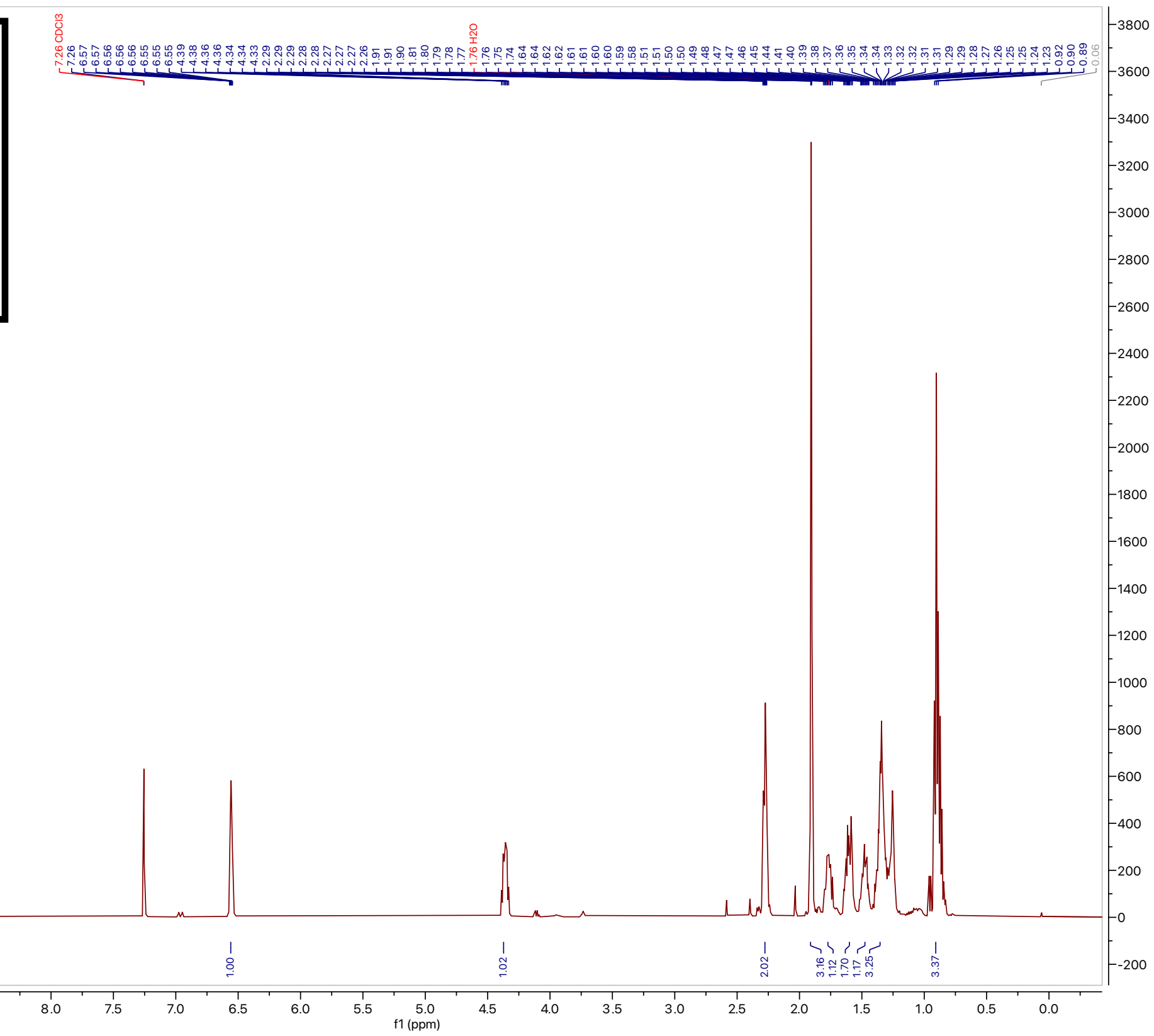

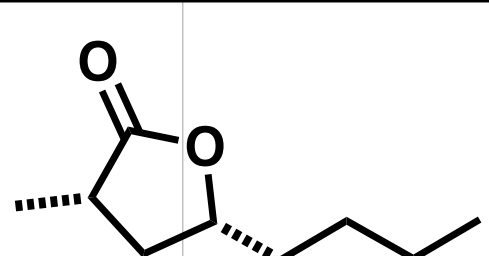

(±) S8n

(^{1}H NMR, $CDCl_3$, 500 MHz)

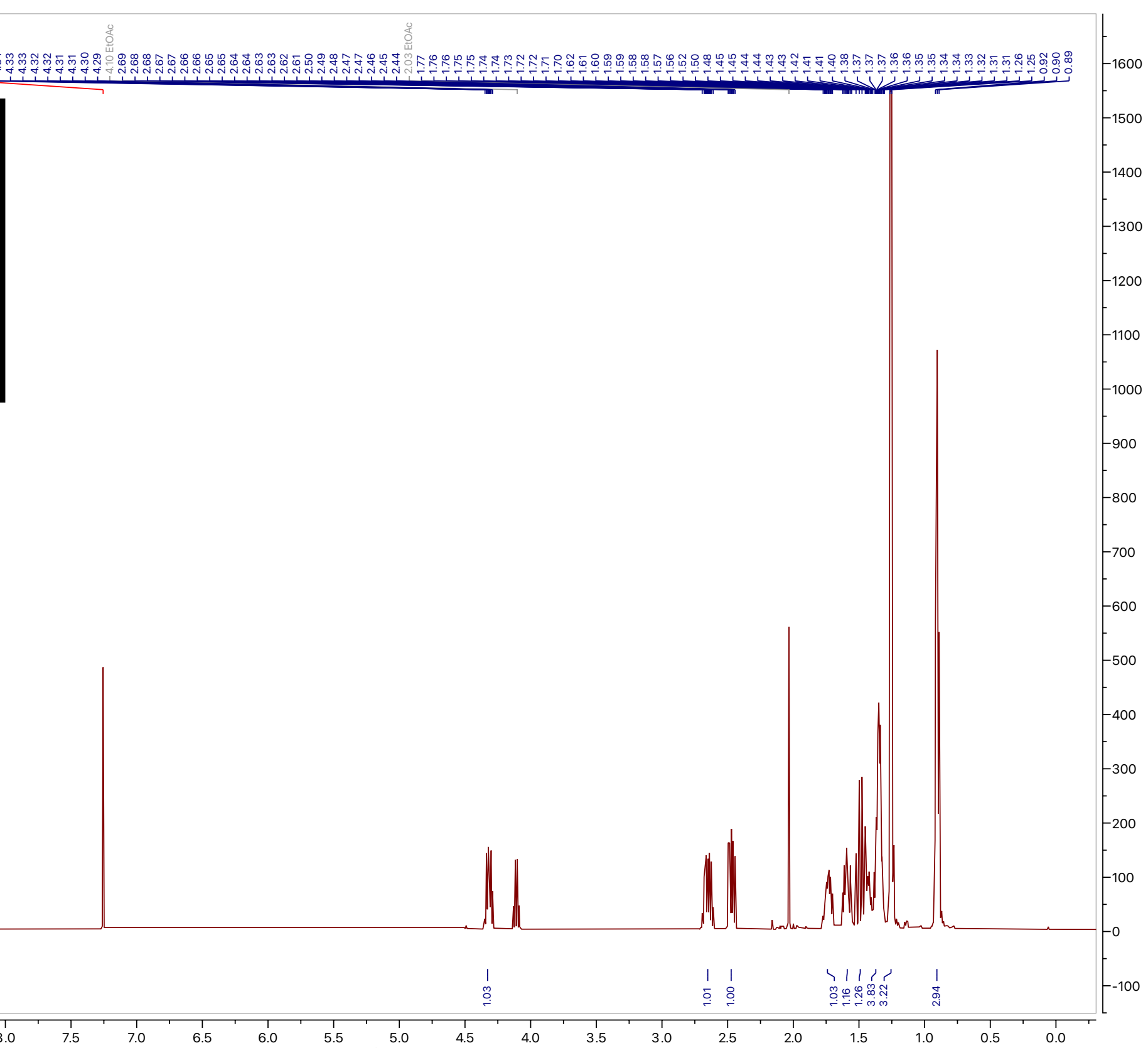

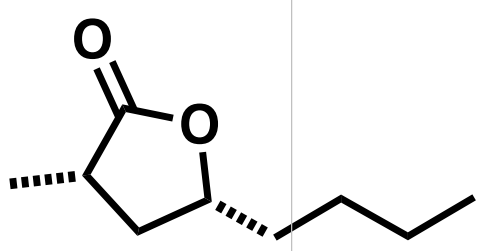

(±) S8n

(^{13}C NMR, $CDCl_3$, 126 MHz)

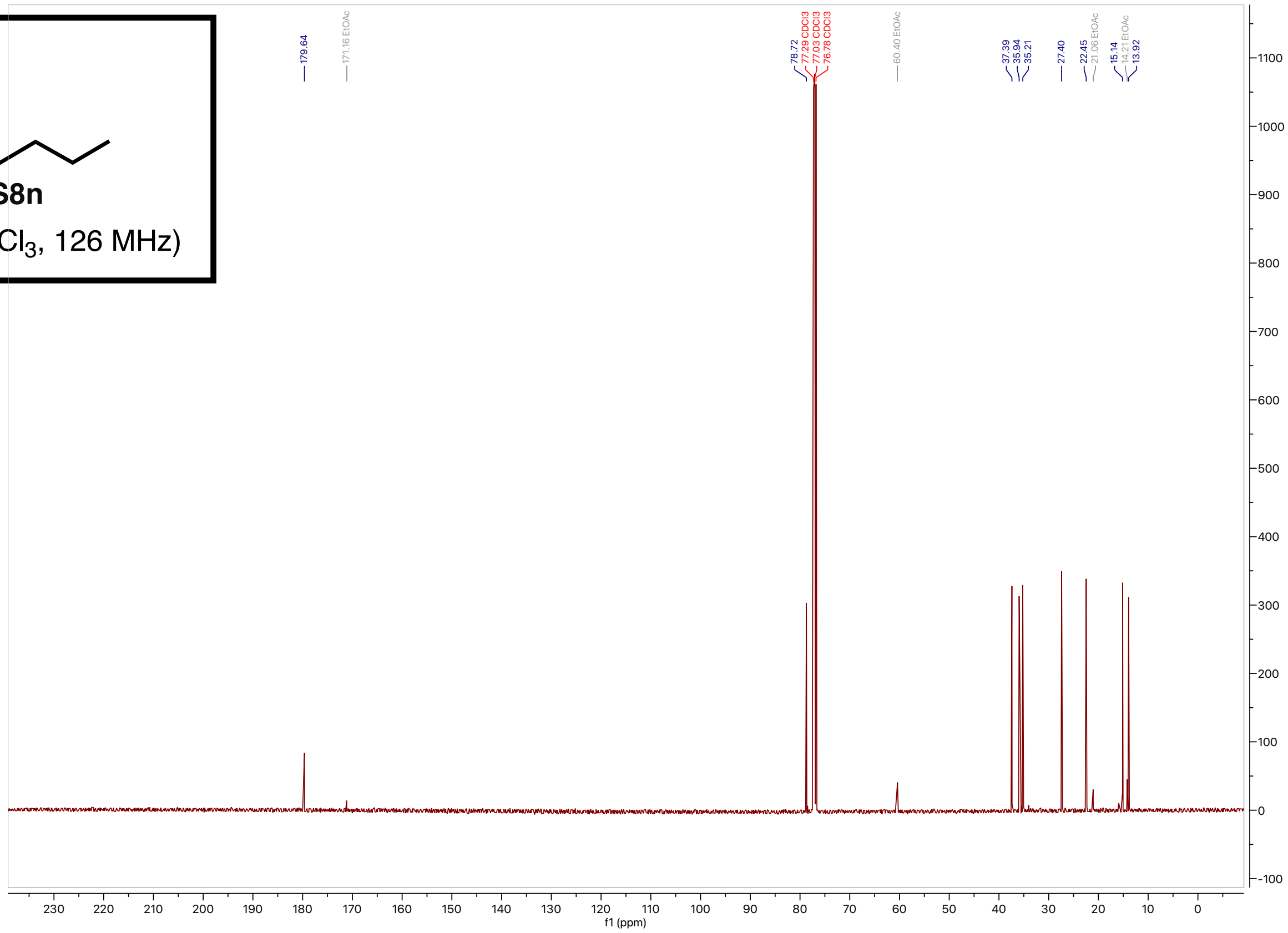

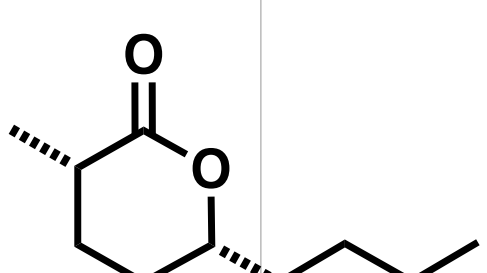

(±) S8o

(^{1}H NMR, $CDCl_3$, 500 MHz, 86% purity)

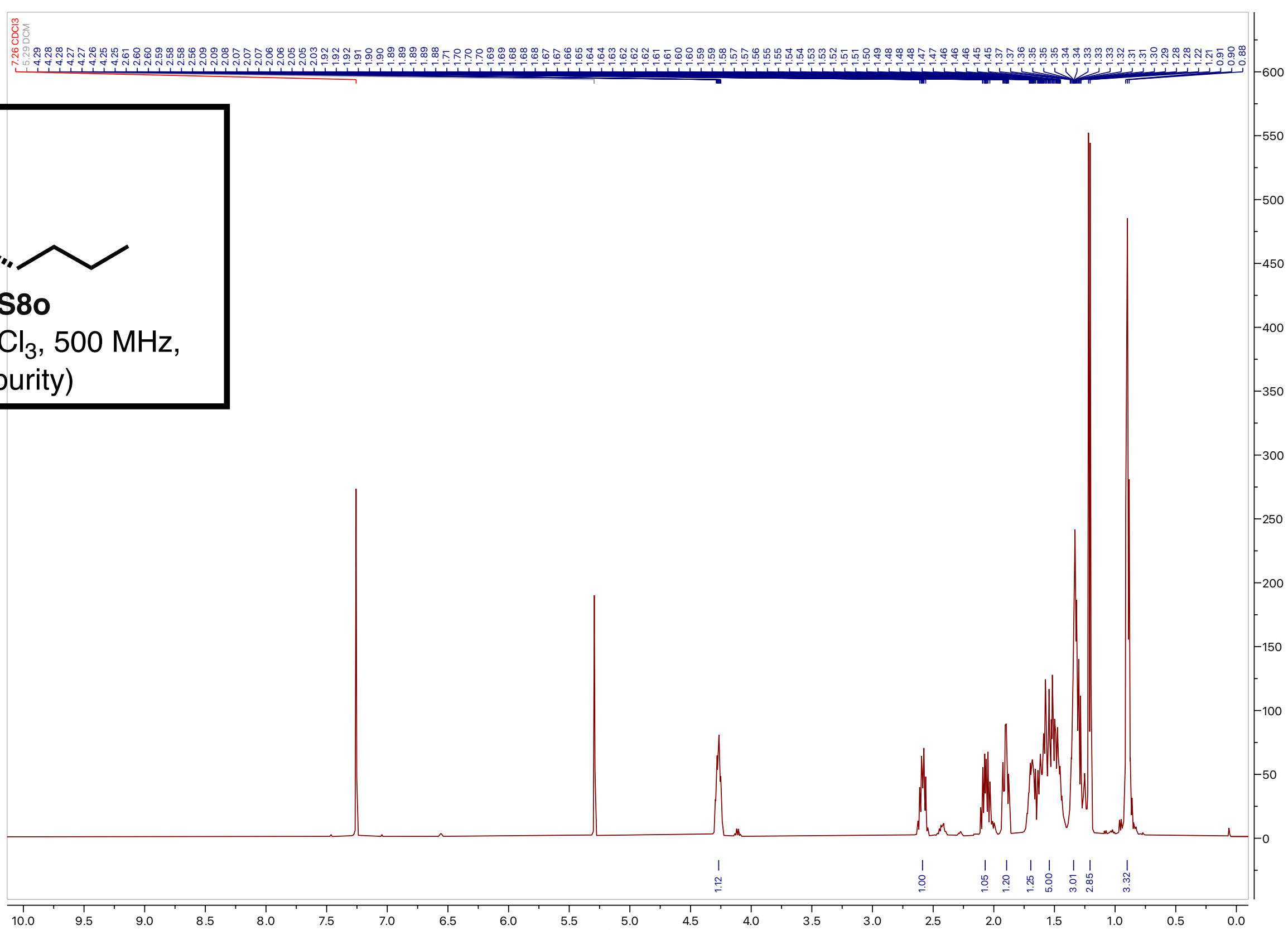

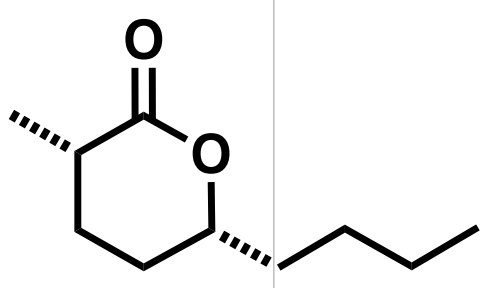

(±) S8o
(^{13}C NMR, $CDCl_3$, 126 MHz, 86% purity)

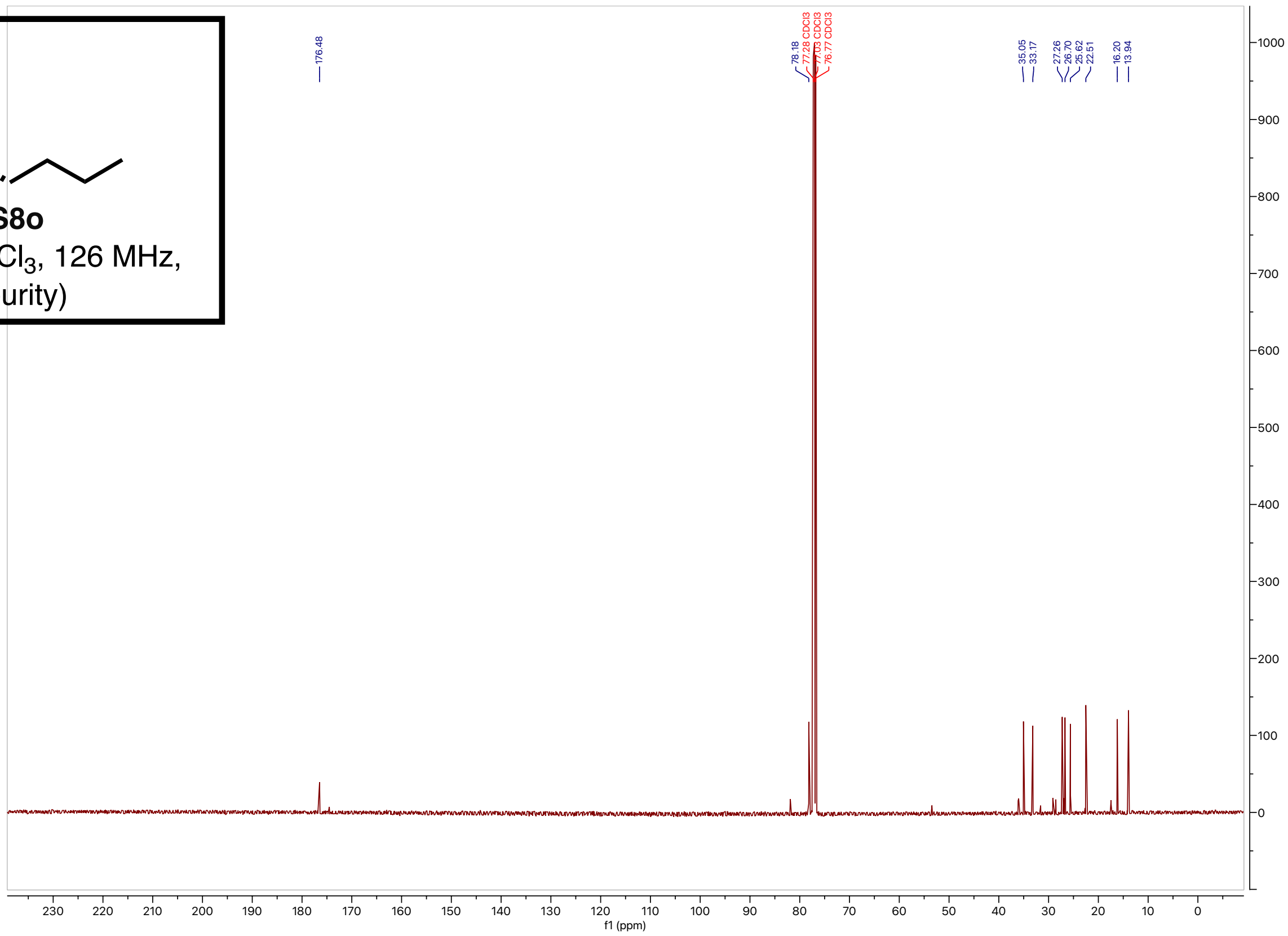

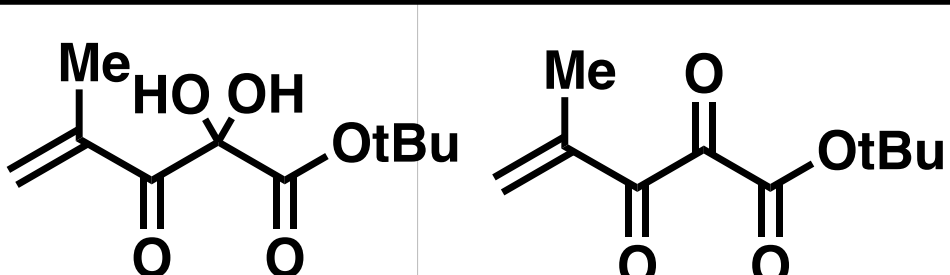

2c·H_2O : **2c**

2.6 : 1

(^{1}H NMR, $CDCl_3$, 600 MHz)

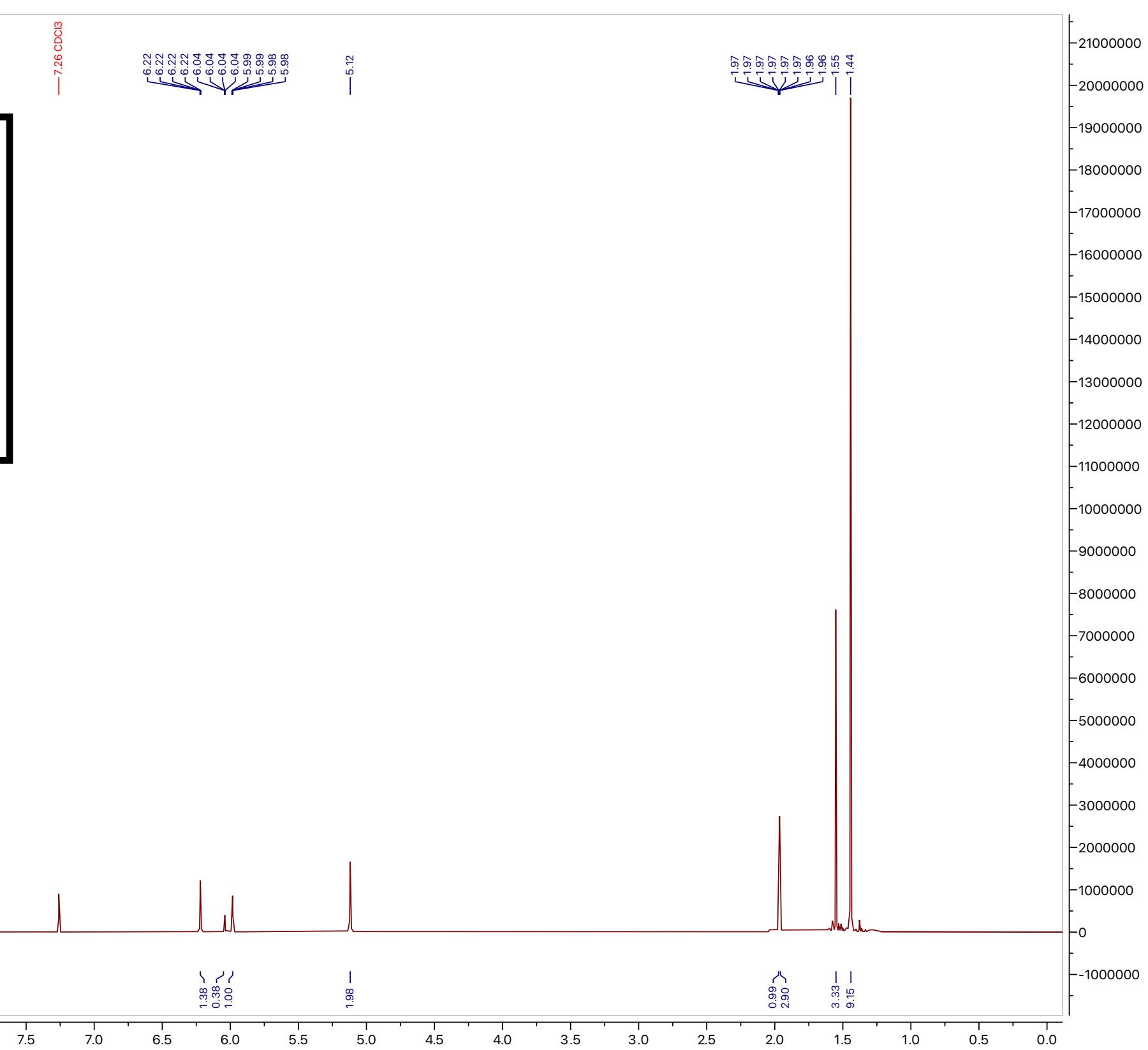

S23 ^{1}H NMR (500 MHz, Chloroform-*d*)

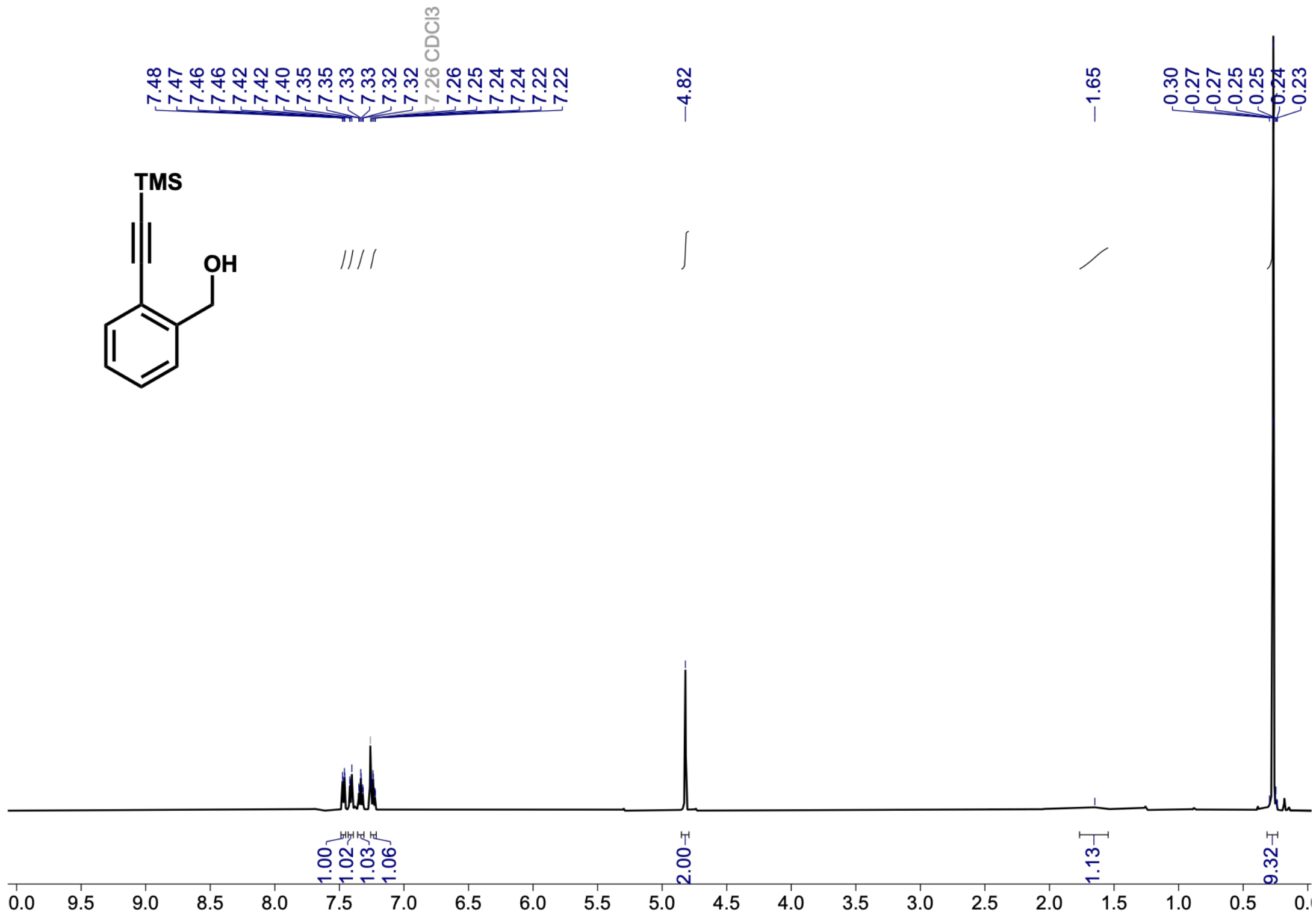

S23 ^{13}C NMR (126 MHz, $CDCl_3$)

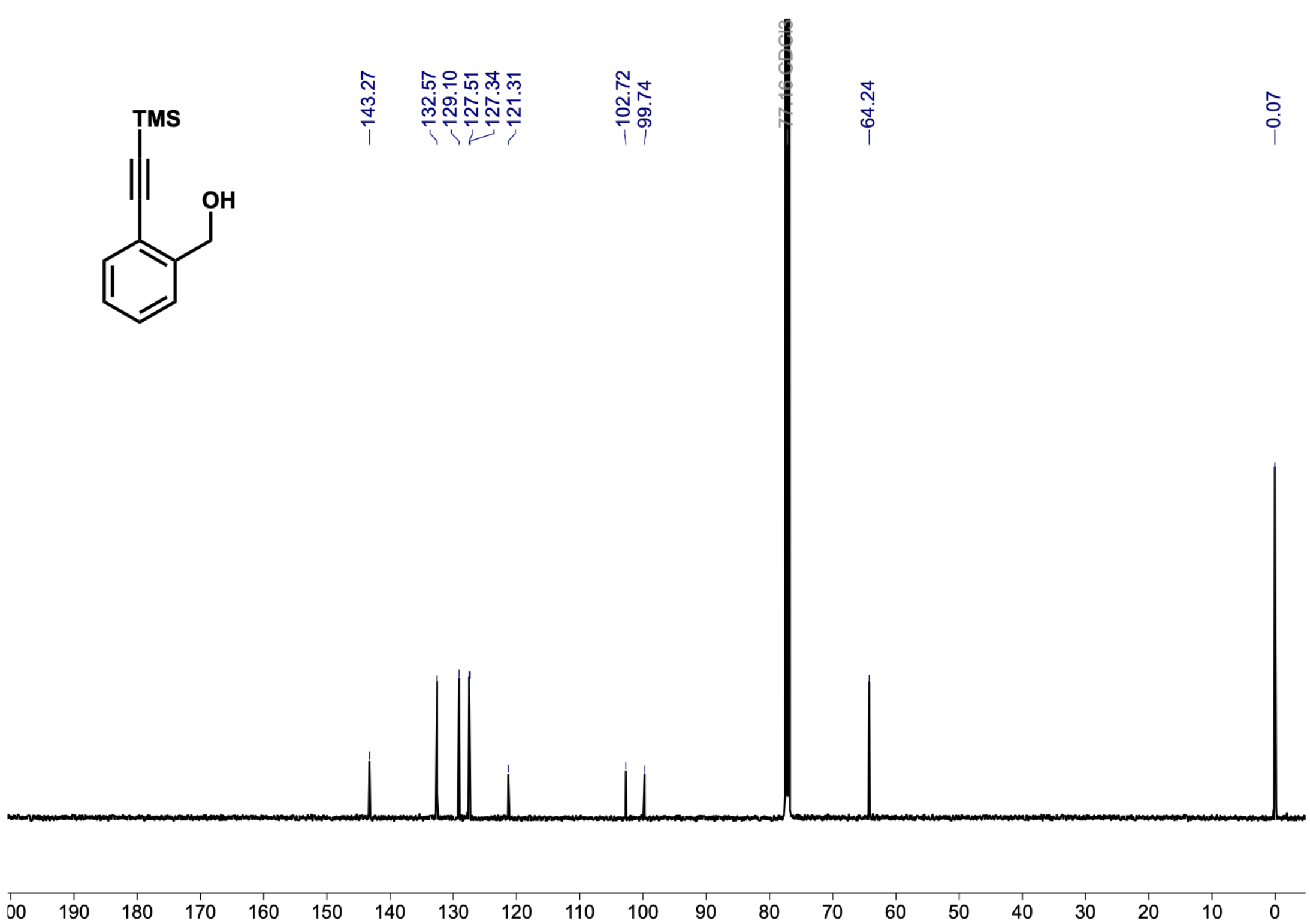

S24 ^{1}H NMR (500 MHz, Chloroform-*d*)

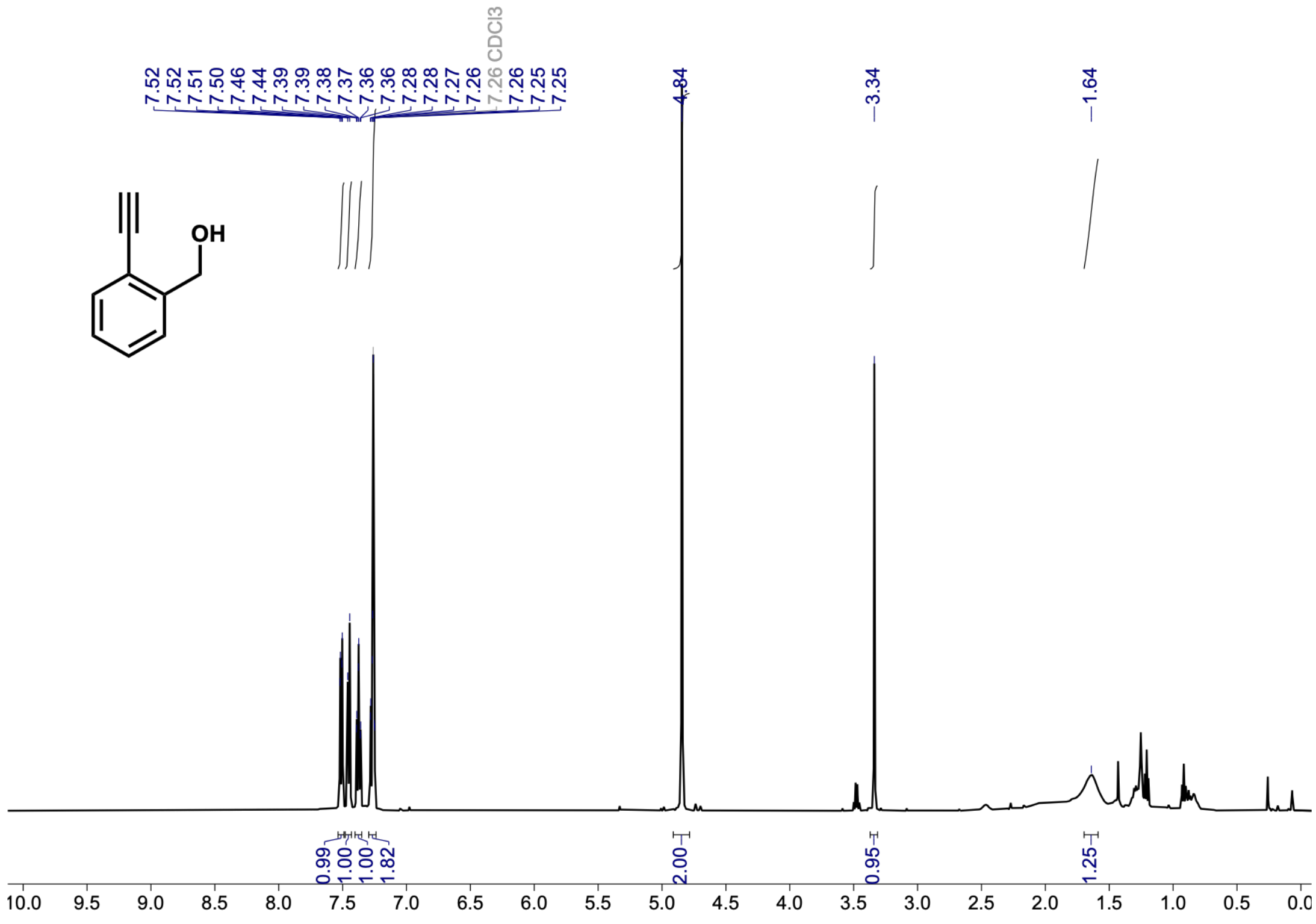

S24 ^{13}C NMR (126 MHz, $CDCl_3$)

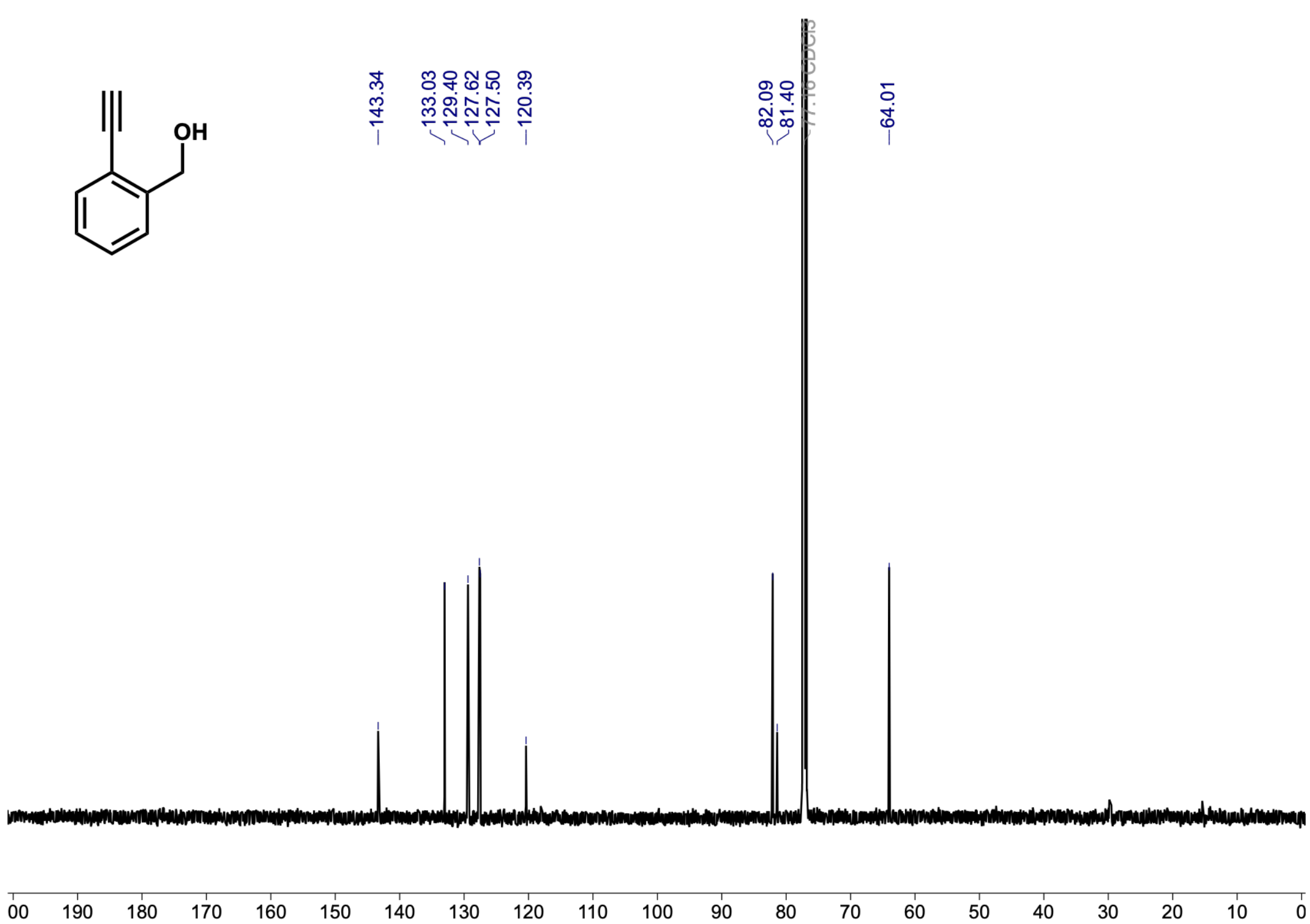

1j ^{1}H NMR (600 MHz, Chloroform-*d*)

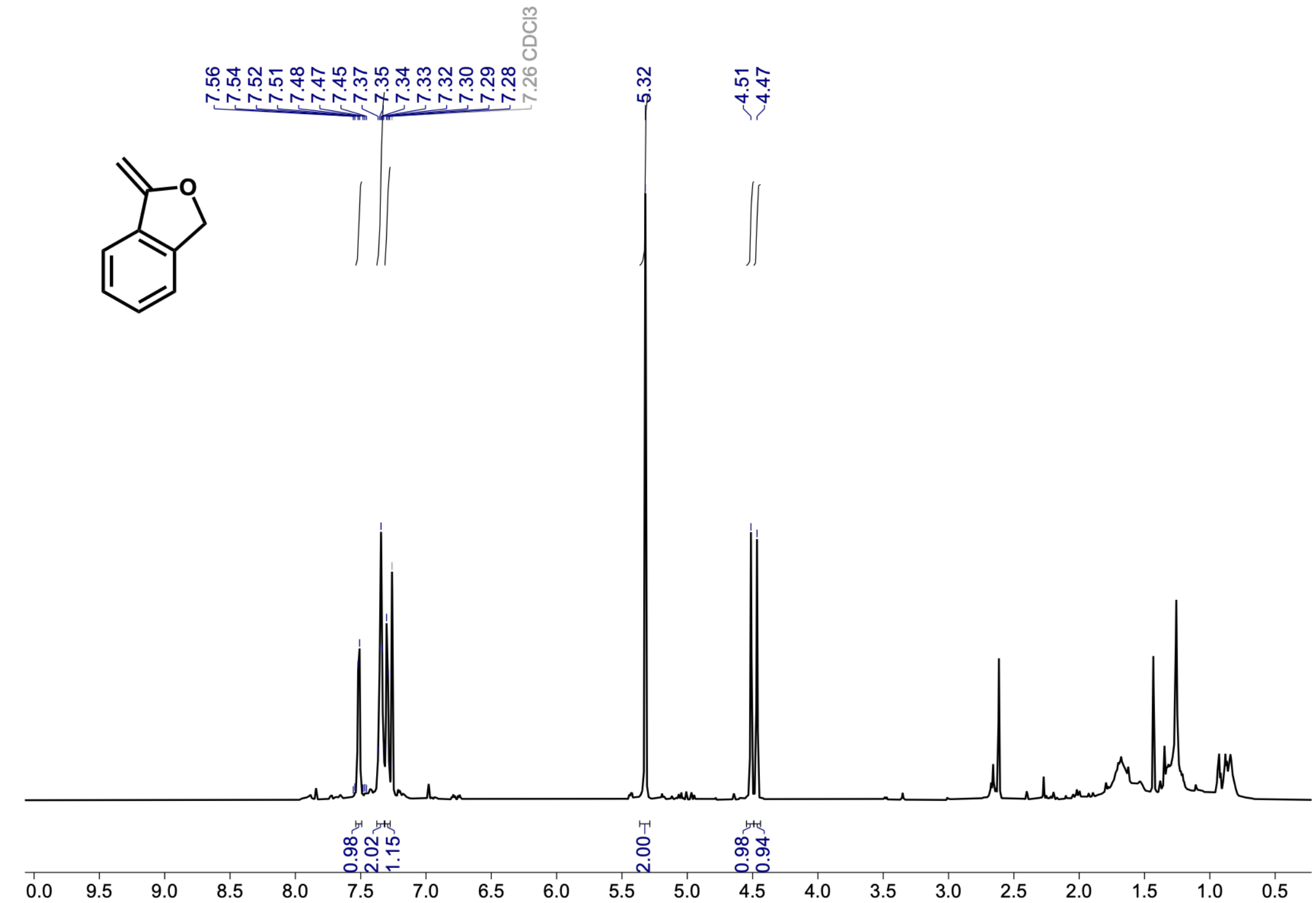

1j ^{13}C NMR (151 MHz, Chloroform-*d*)

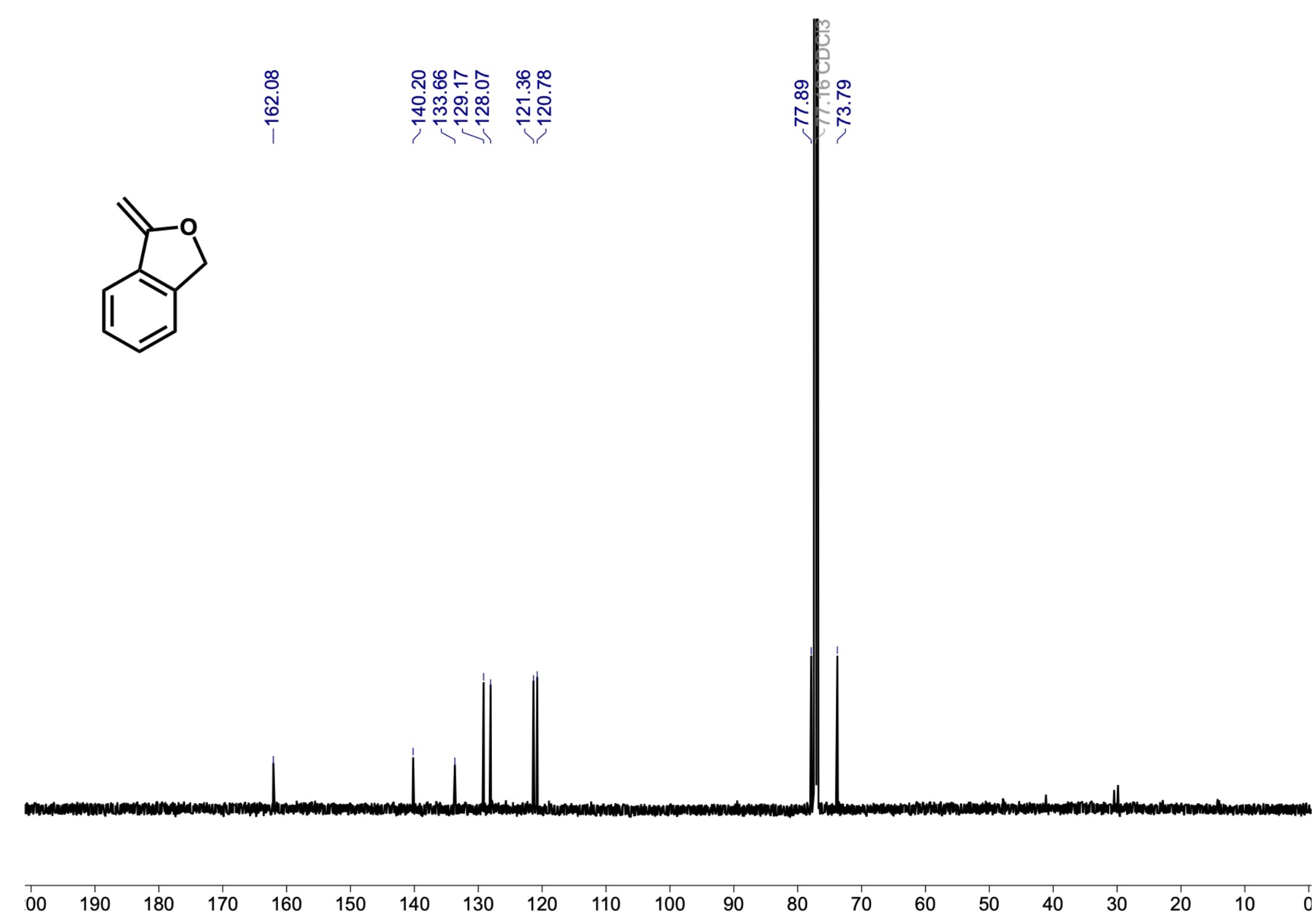

S25 ¹H NMR (600 MHz, DMSO-d_6)

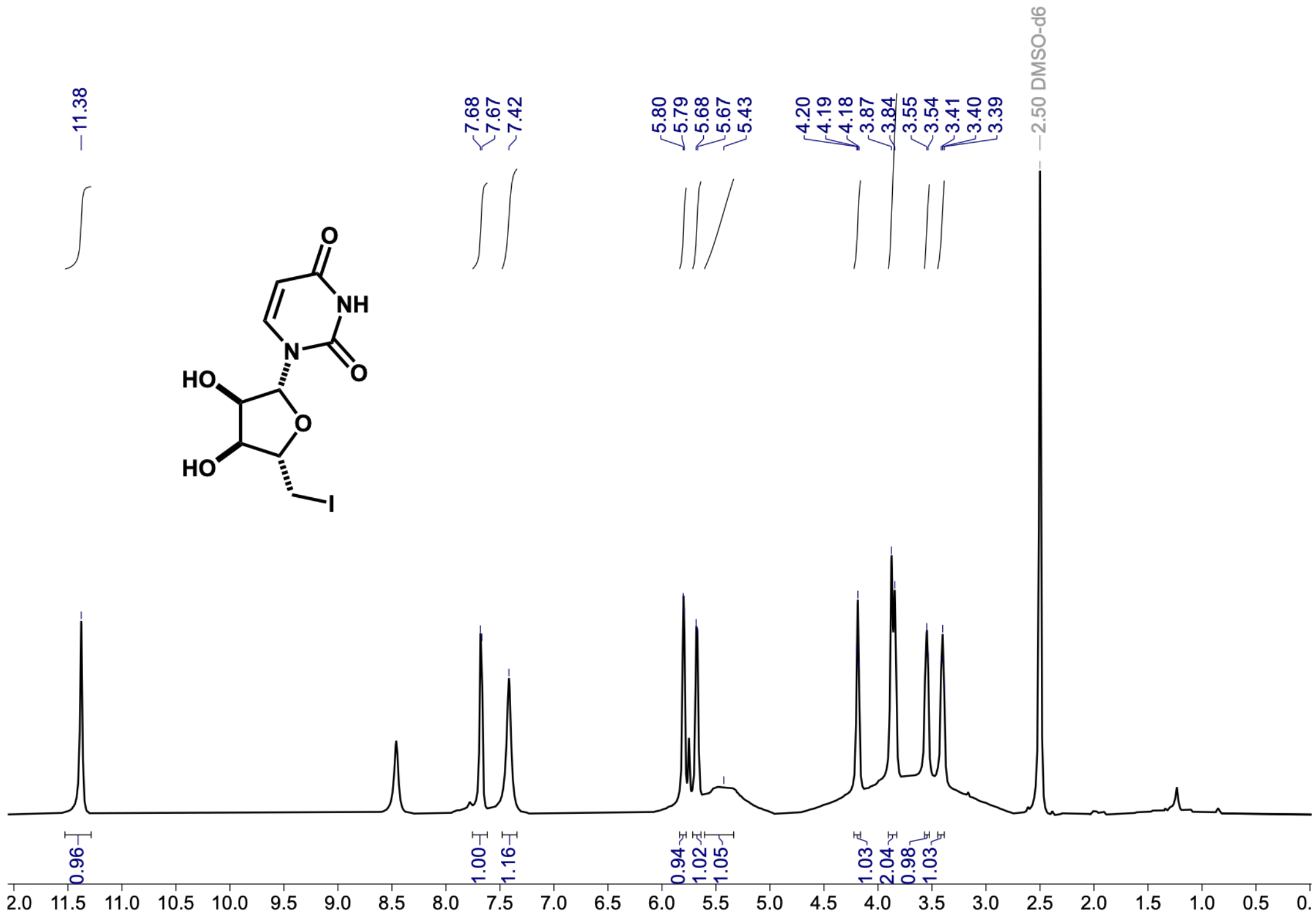

S25 ¹³C NMR (151 MHz, DMSO-d_6)

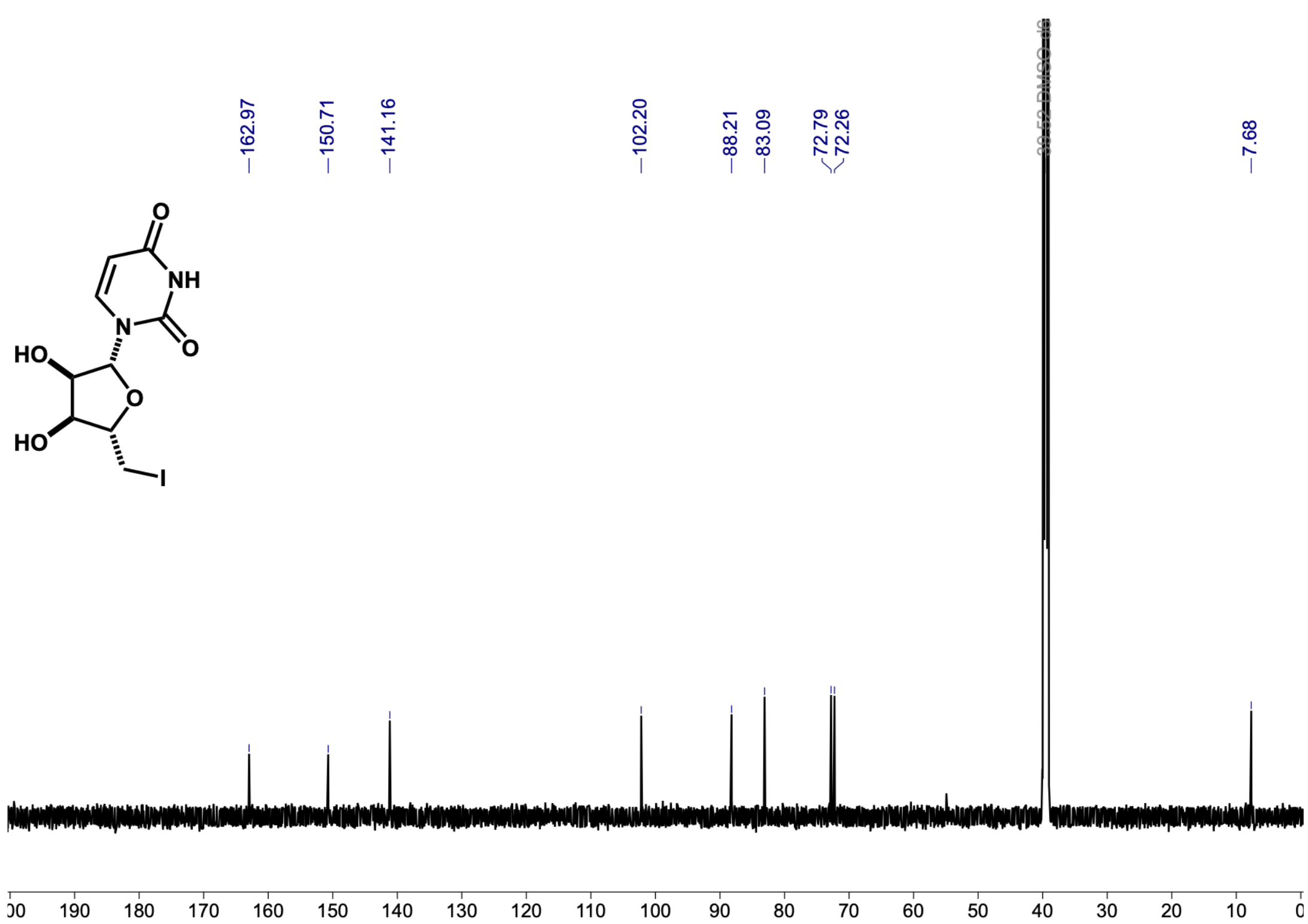

1k ^{1}H NMR (500 MHz, DMSO-d_6)

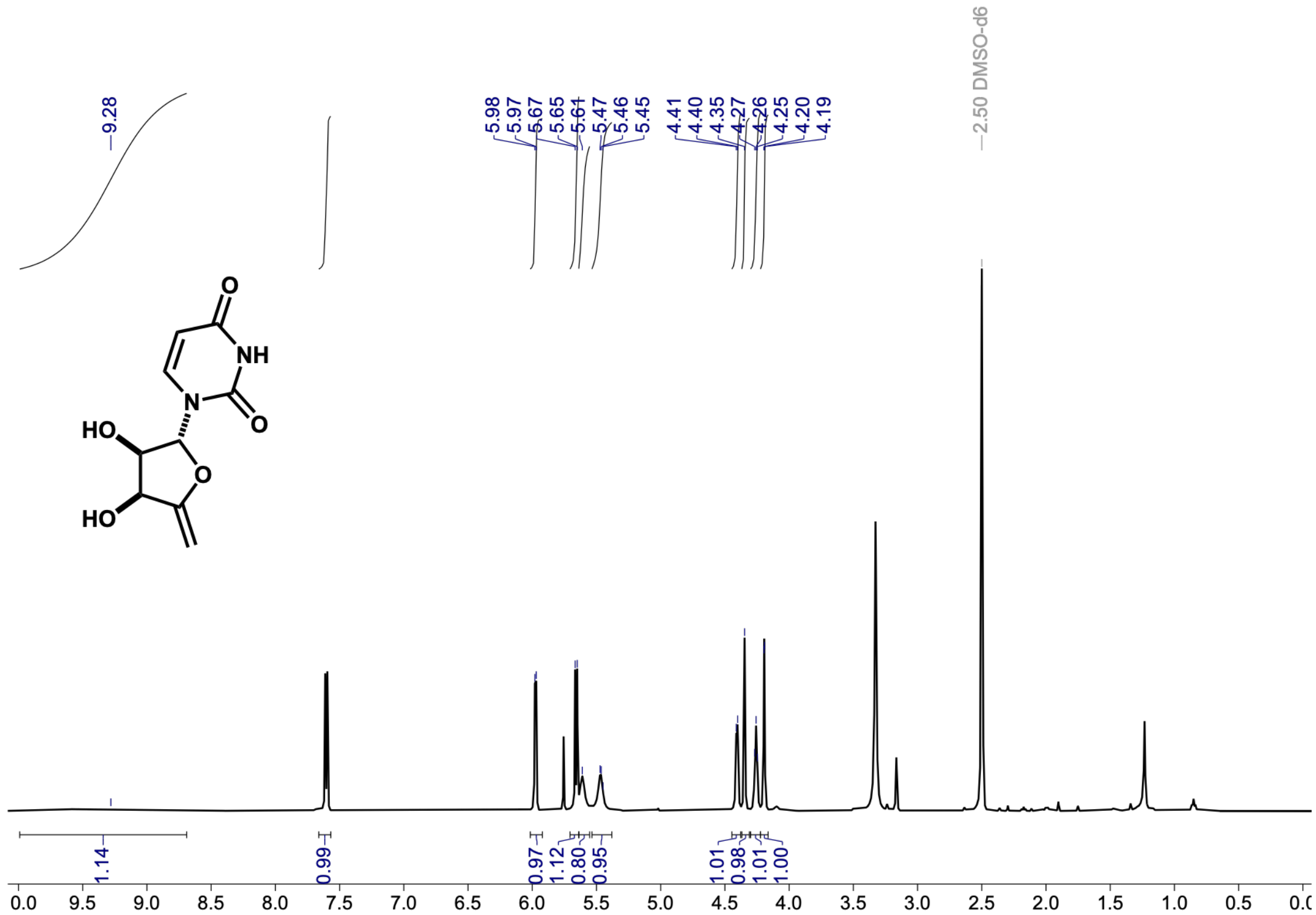

1k ^{13}C NMR (126 MHz, DMSO-d_6)

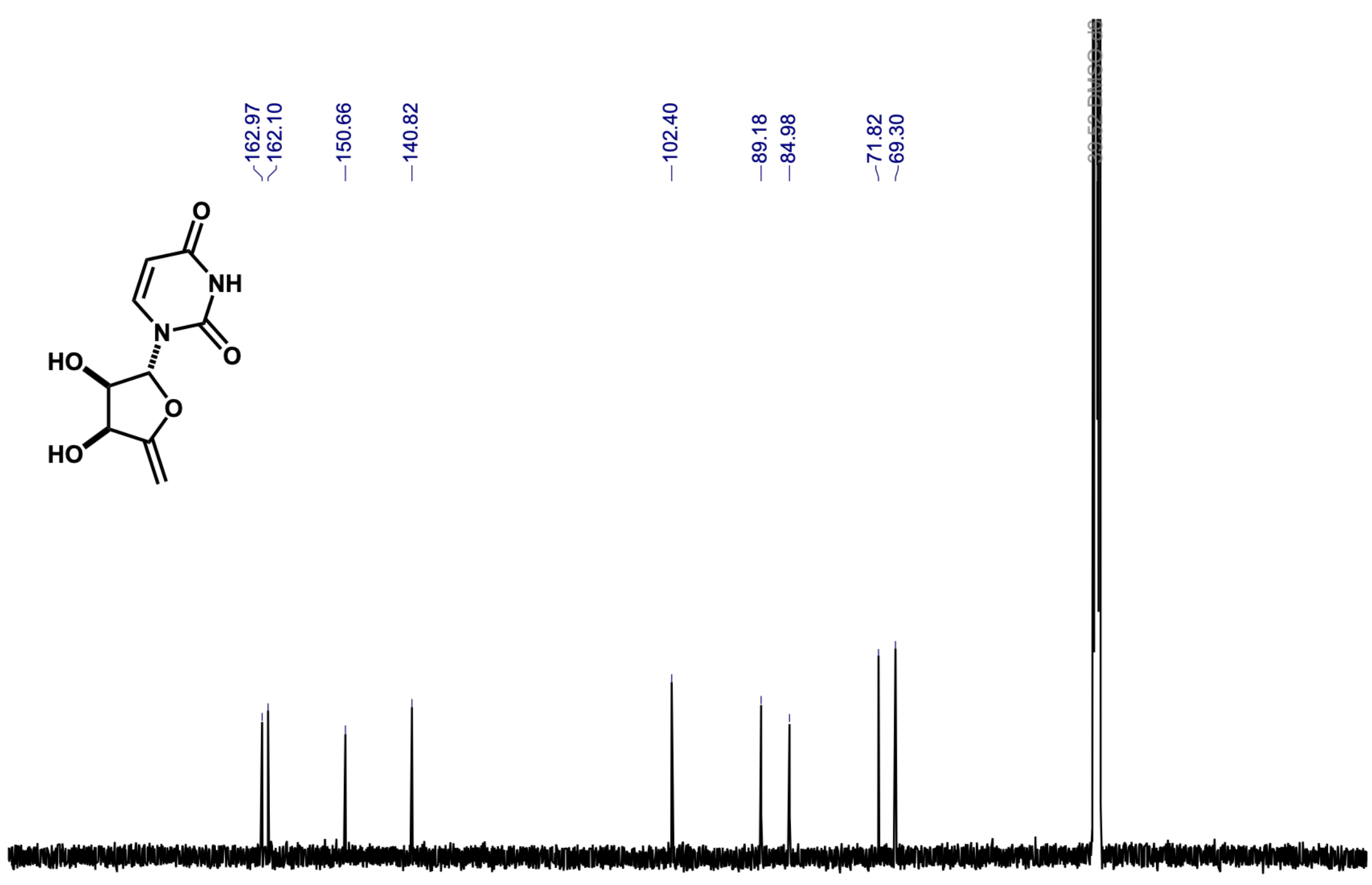

S26 ¹H NMR (600 MHz, Chloroform-*d*)

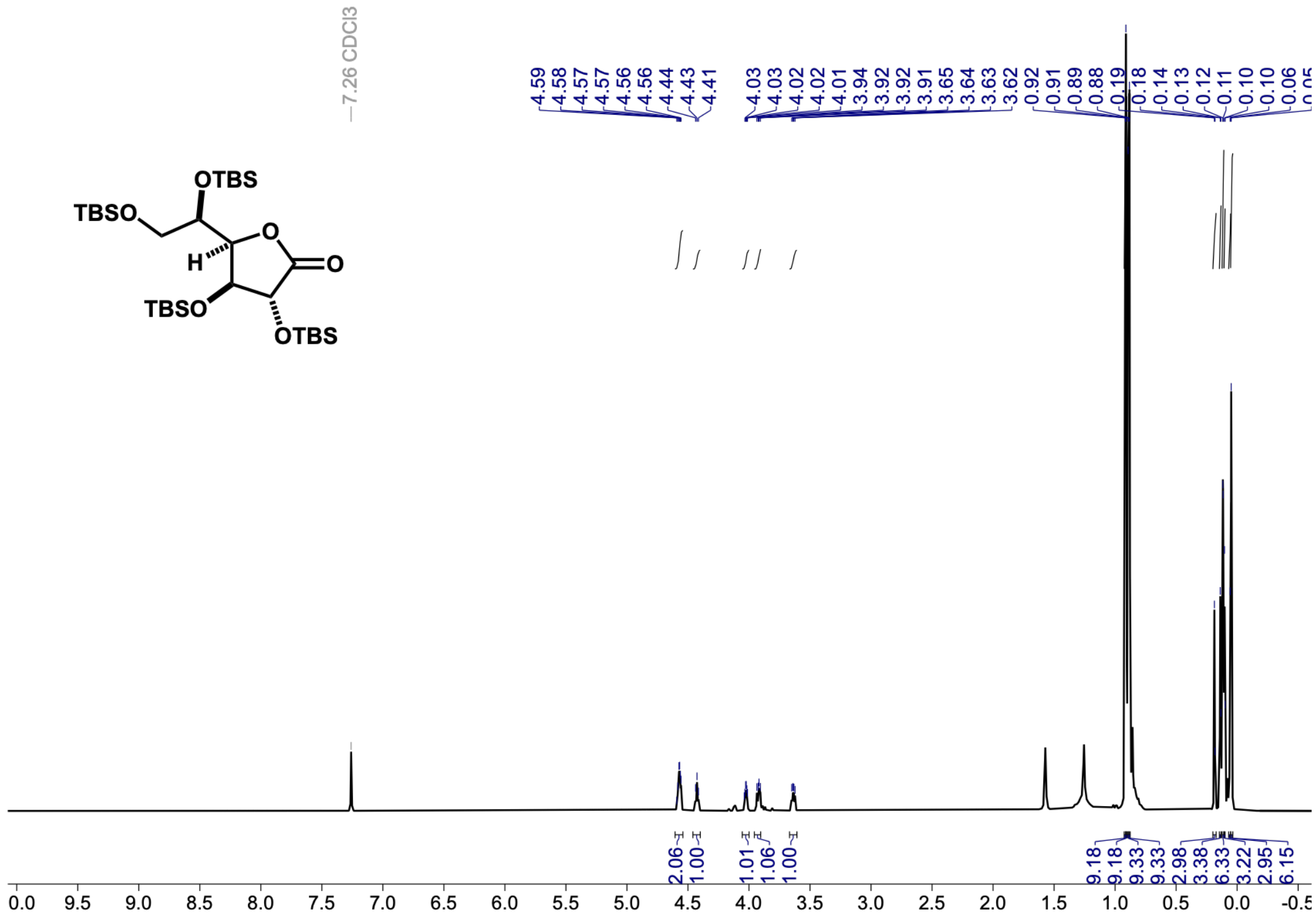

S26 ¹³C NMR (151 MHz, $CDCl_3$)

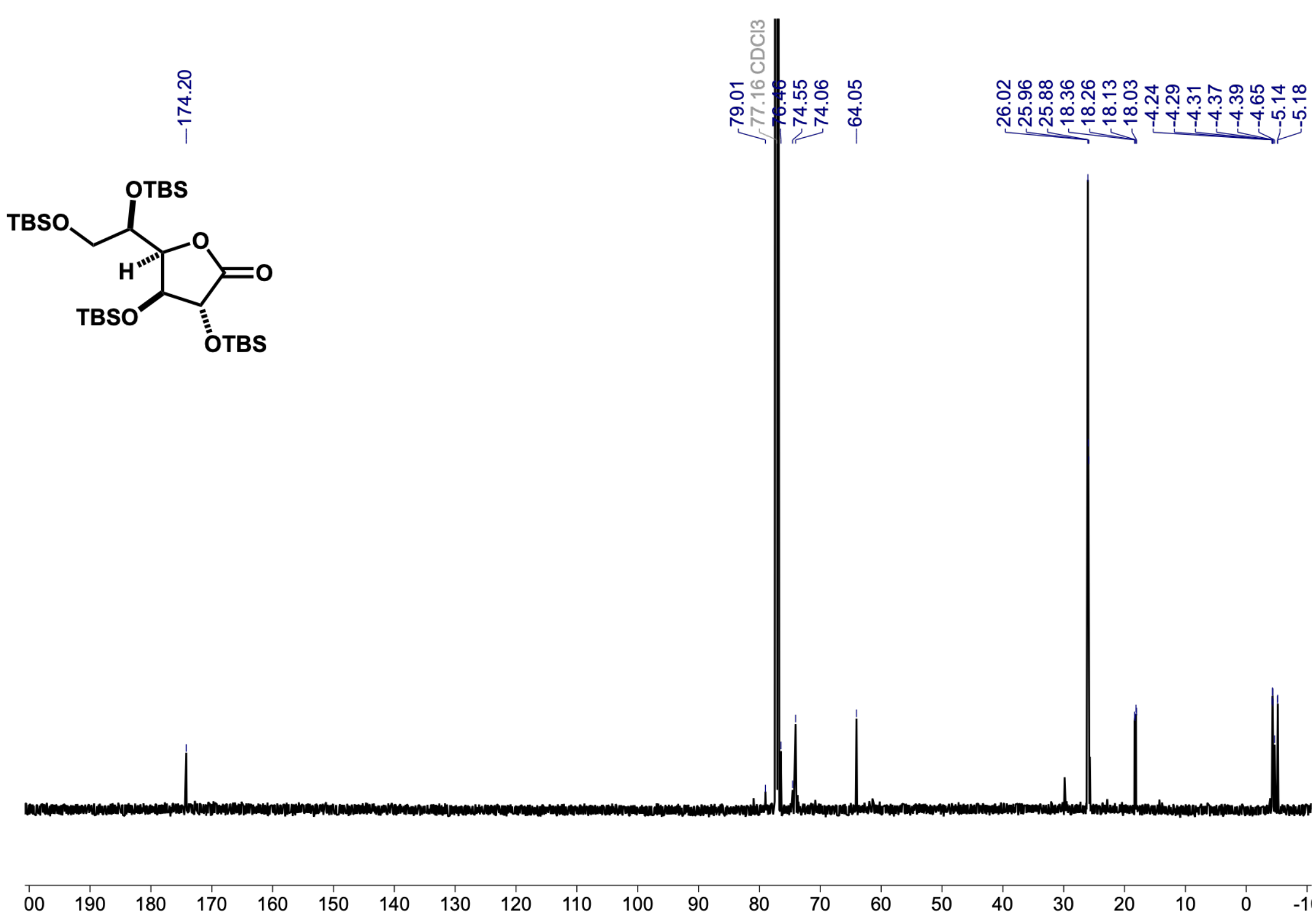

1m ^{1}H NMR (500 MHz, Chloroform-*d*)

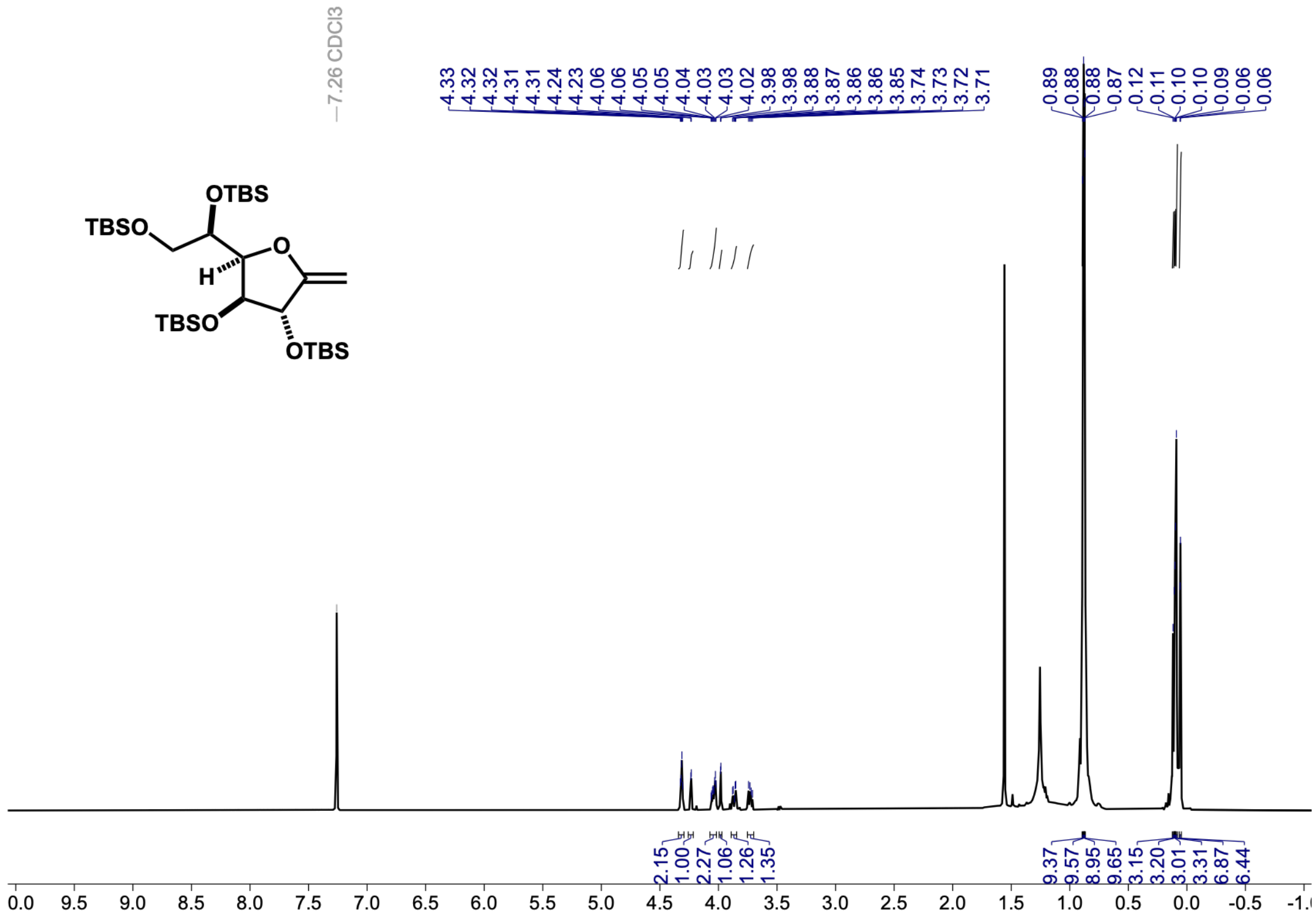

1m ^{13}C NMR (126 MHz, $CDCl_3$)

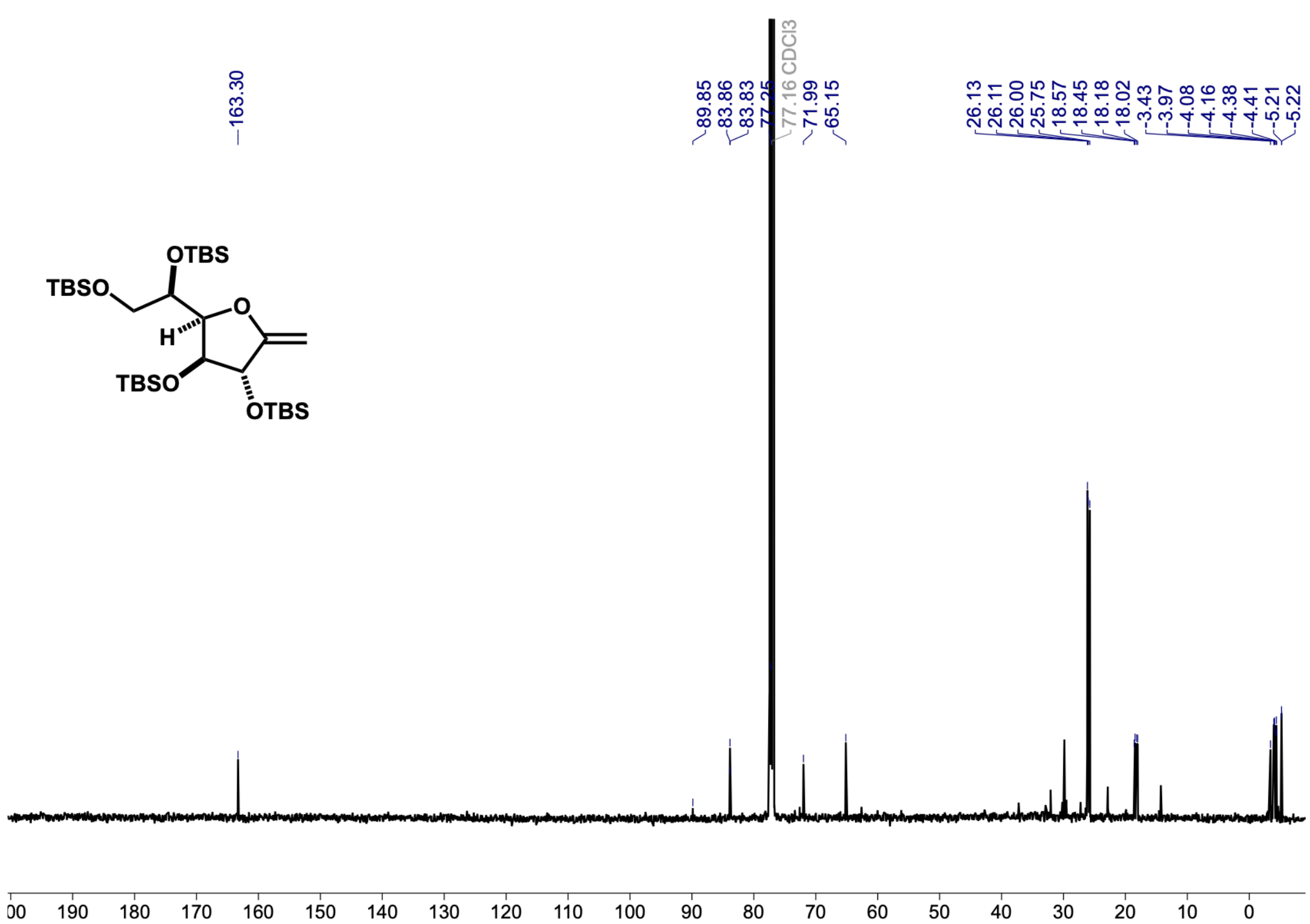

1l ¹H NMR (600 MHz, Methanol-d_4)

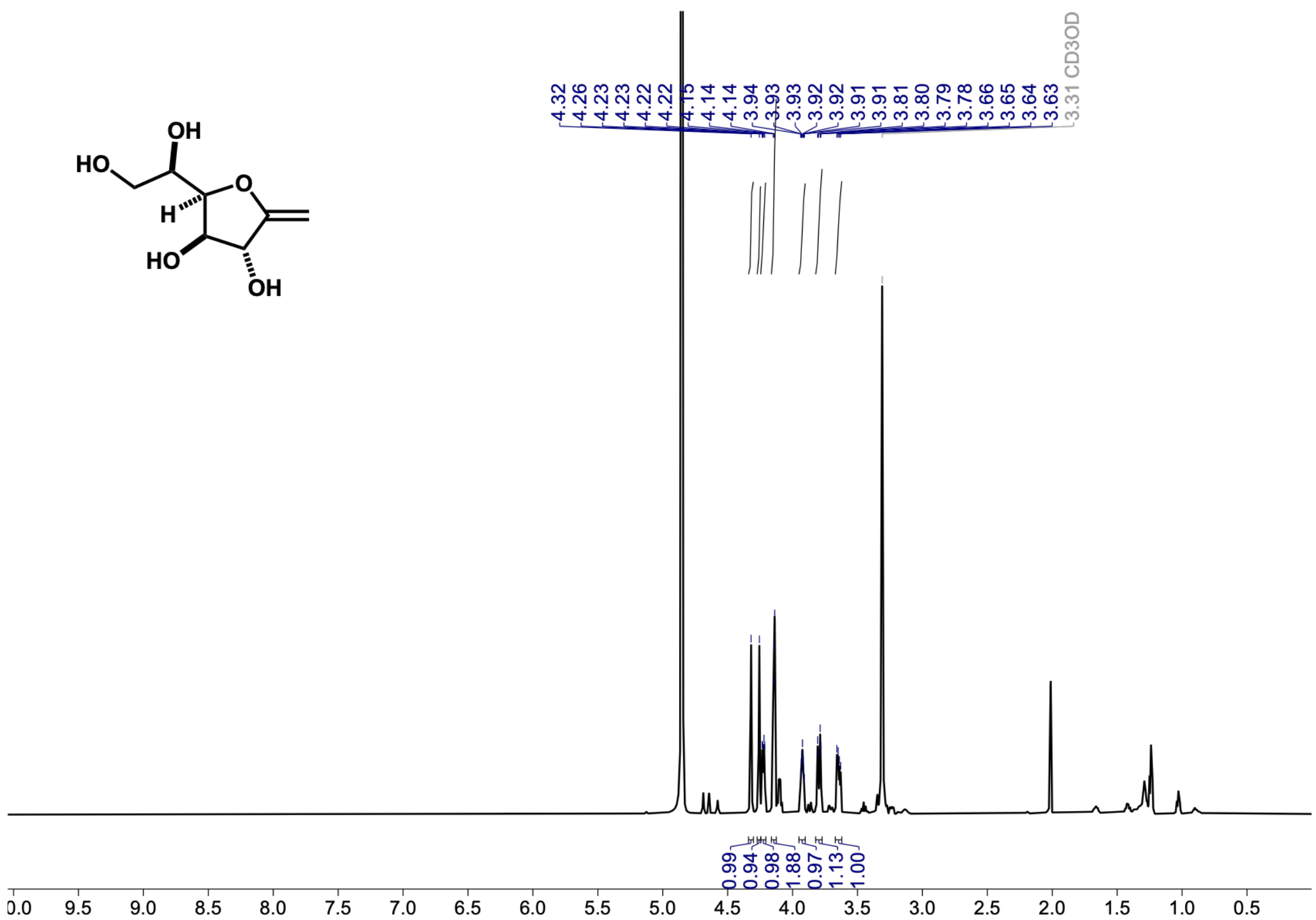

1l ¹³C NMR (151 MHz, MeOD)

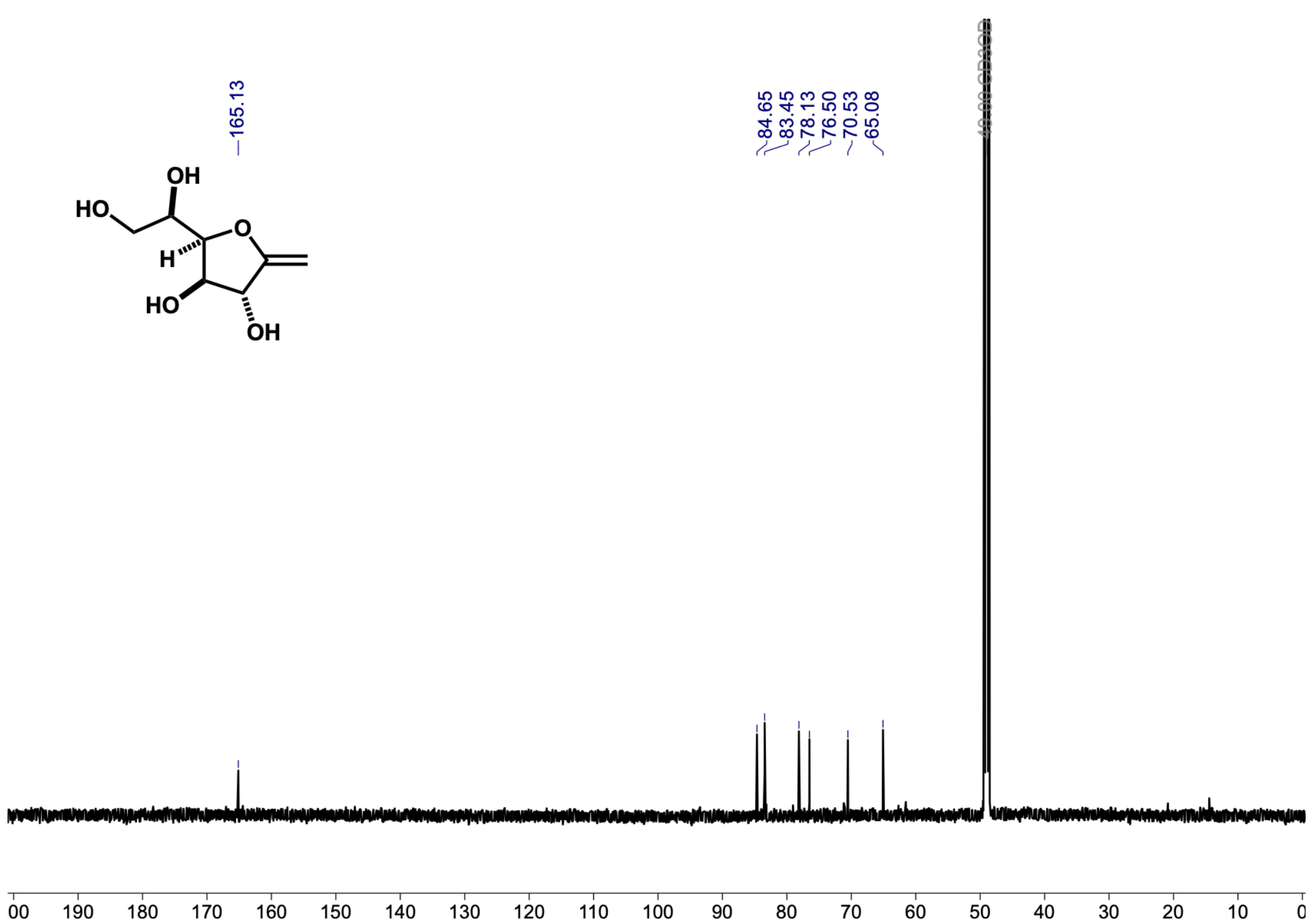

3j ^{1}H NMR (500 MHz, Chloroform-*d*)

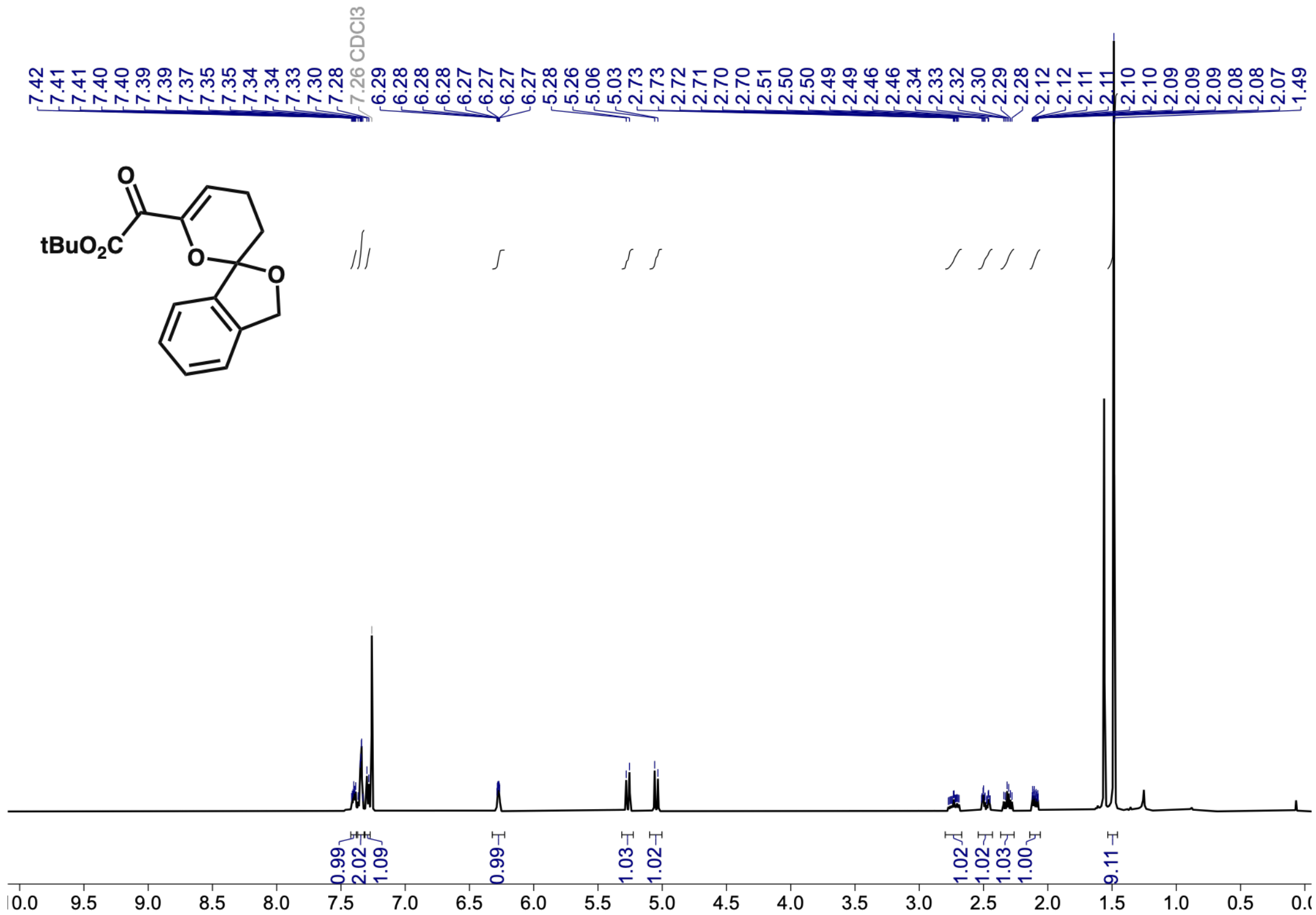

3j ^{13}C NMR (126 MHz, $CDCl_3$)

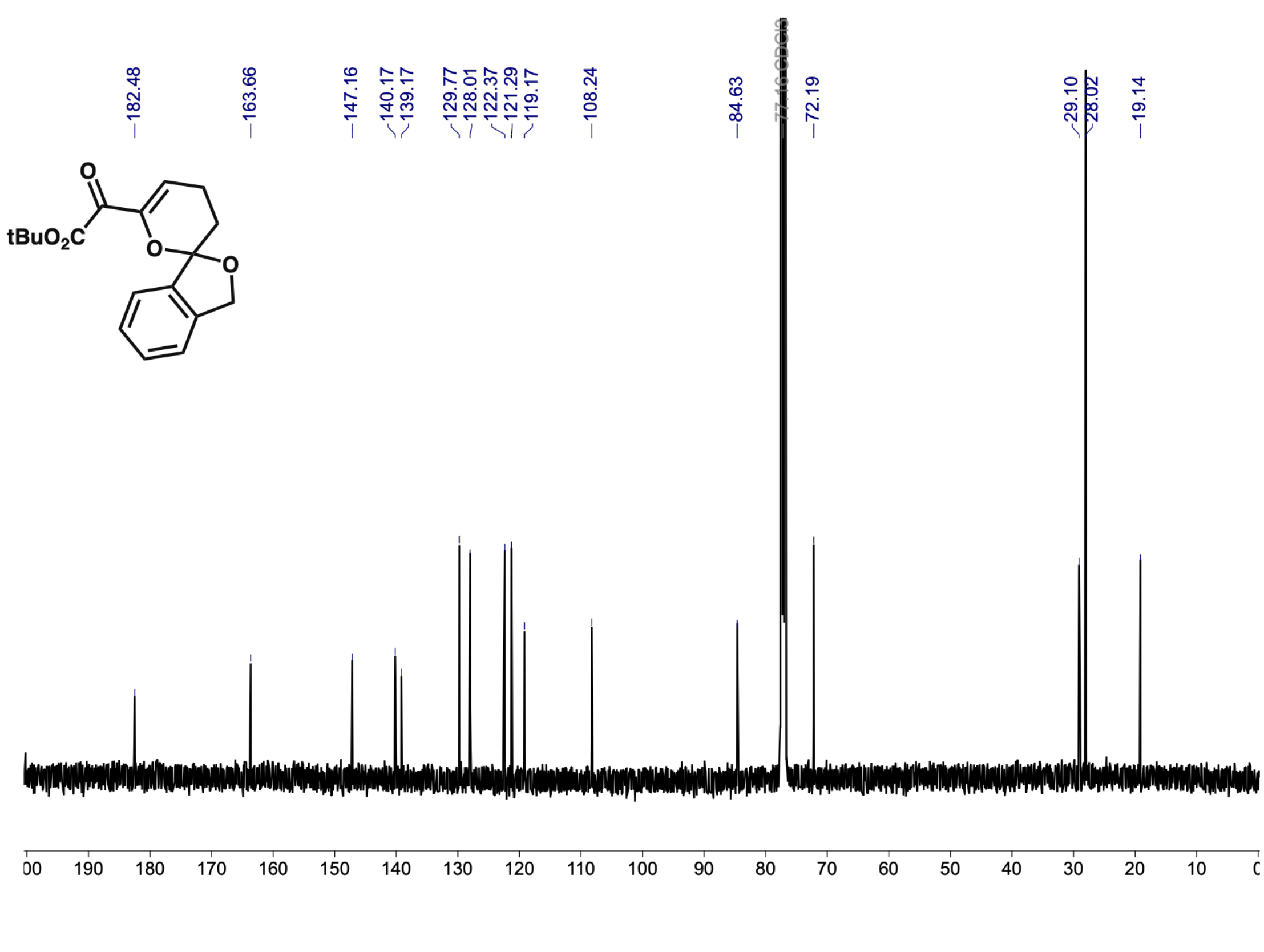

3k ^{1}H NMR (500 MHz, DMSO-d_6)

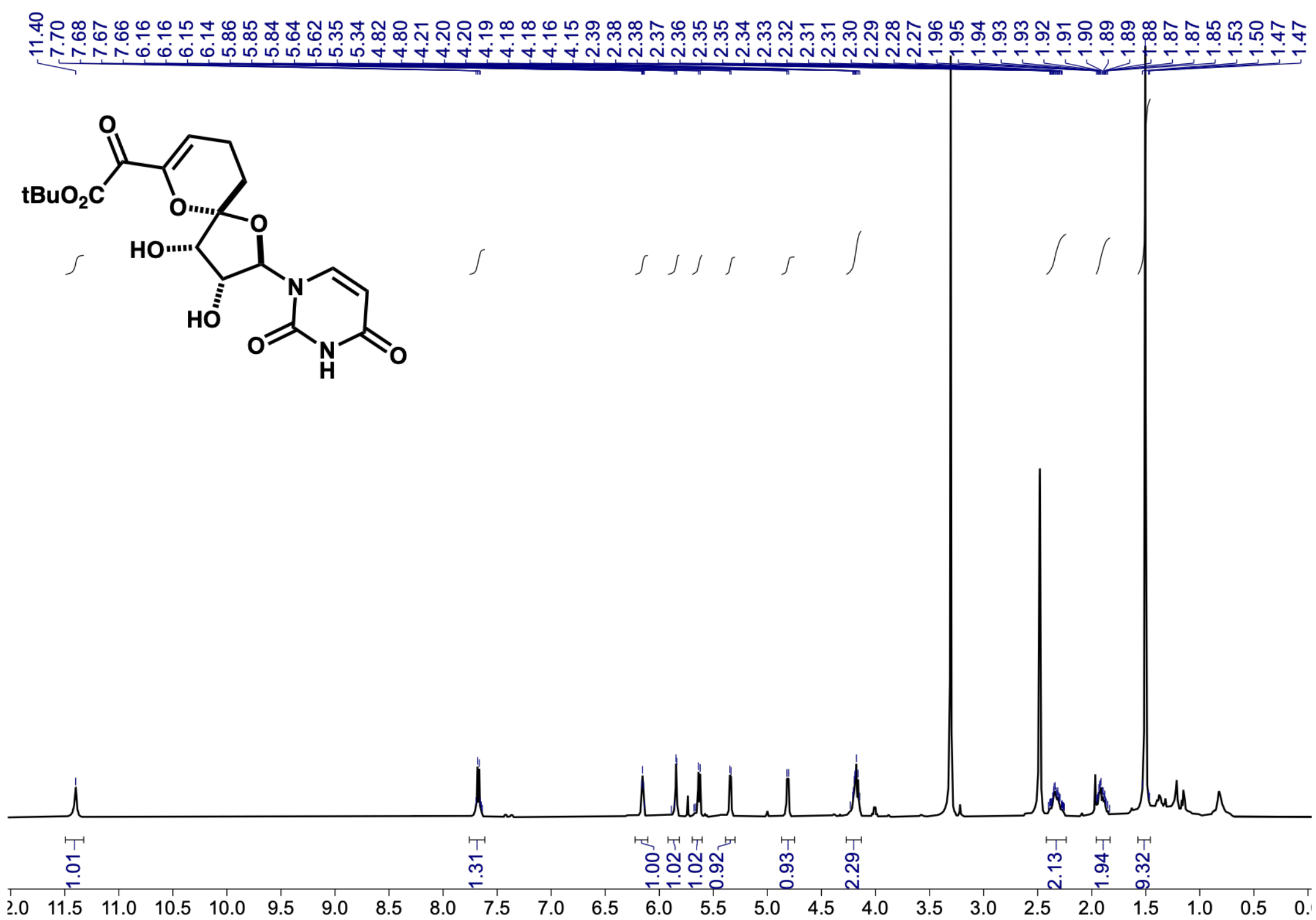

3k ^{13}C NMR (126 MHz, DMSO-d_6)

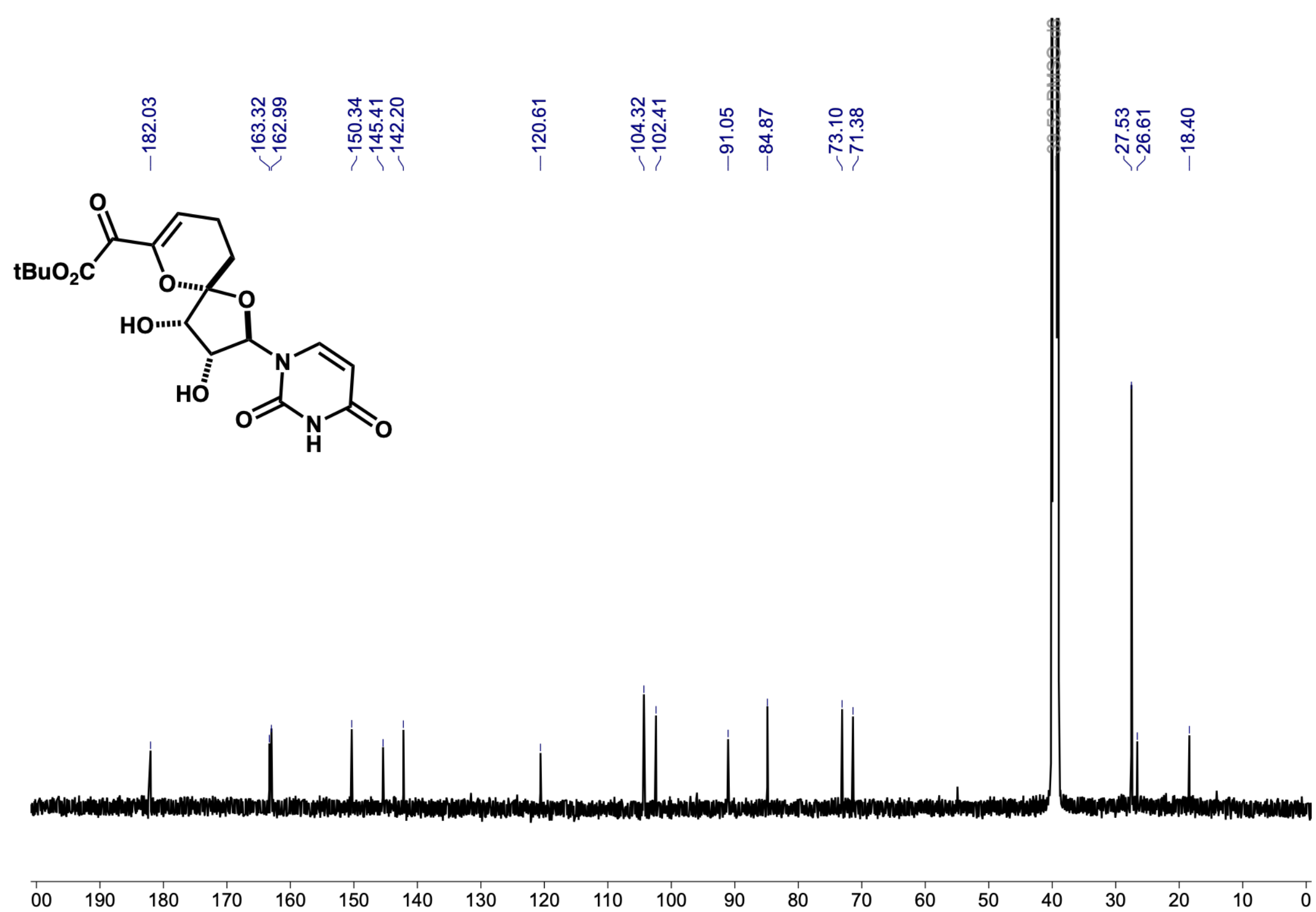

3k ¹H-¹H COSY (500 MHz, DMSO-d_6)

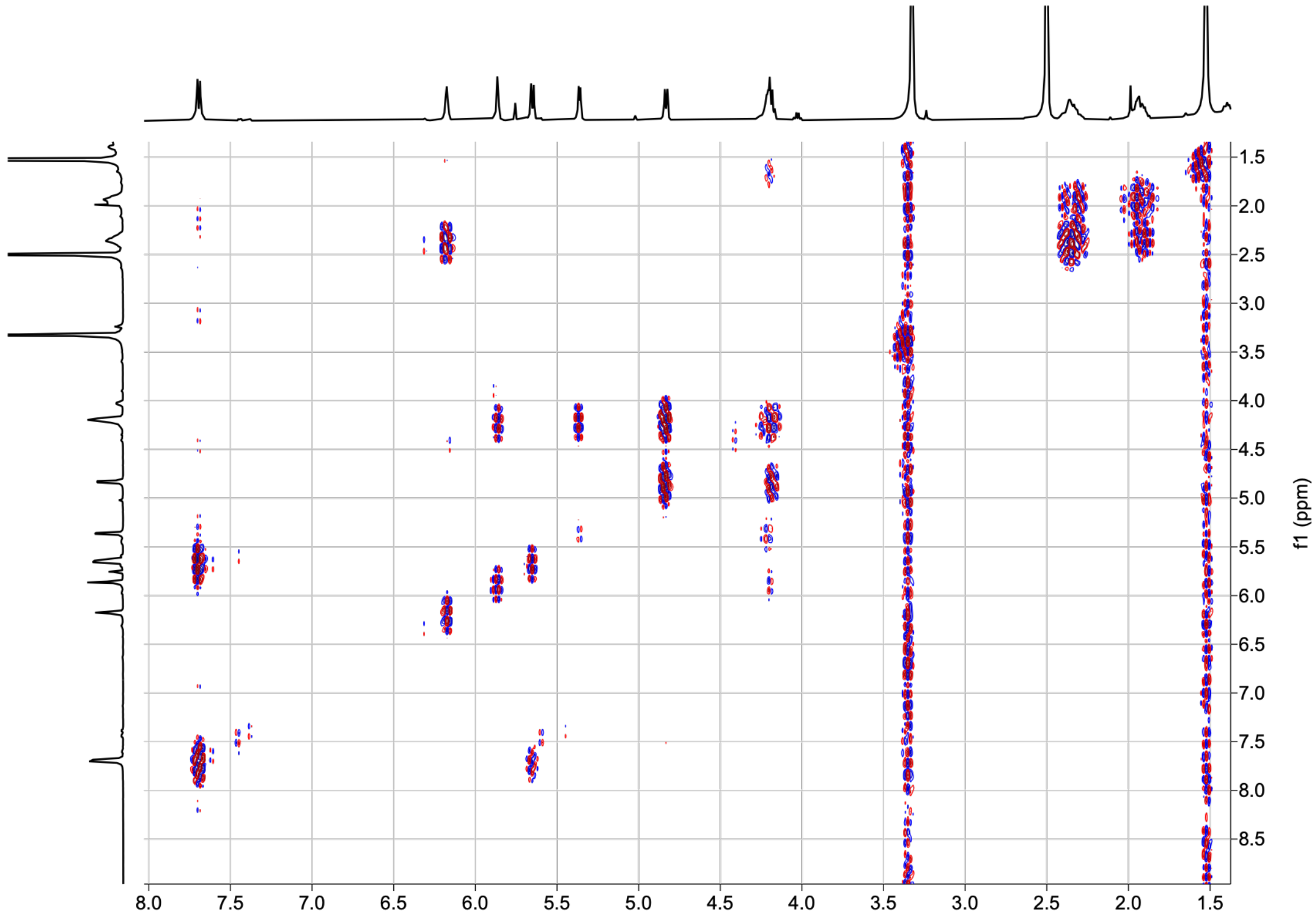

3k ¹H-¹H NOESY (500 MHz, DMSO-d_6)

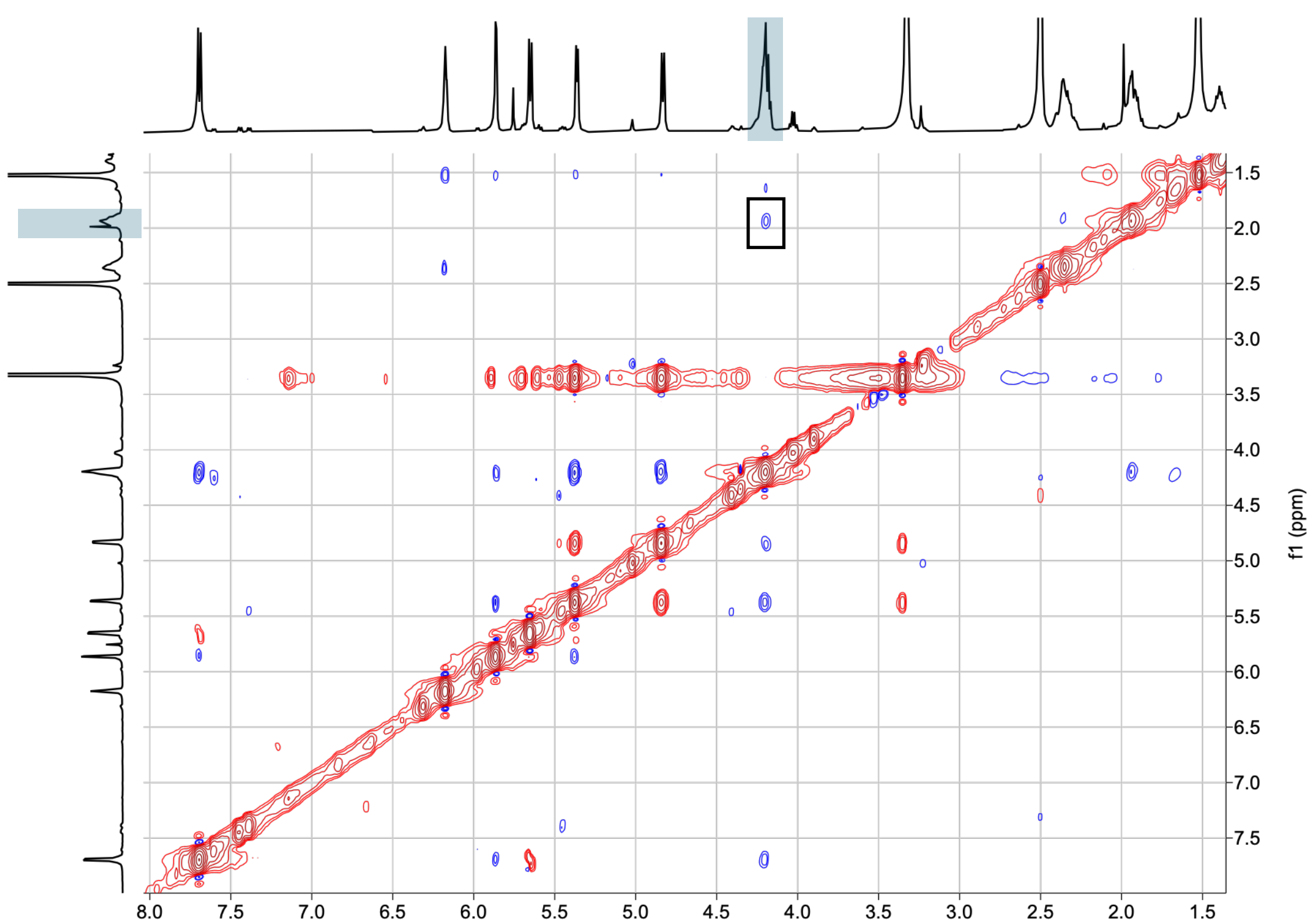

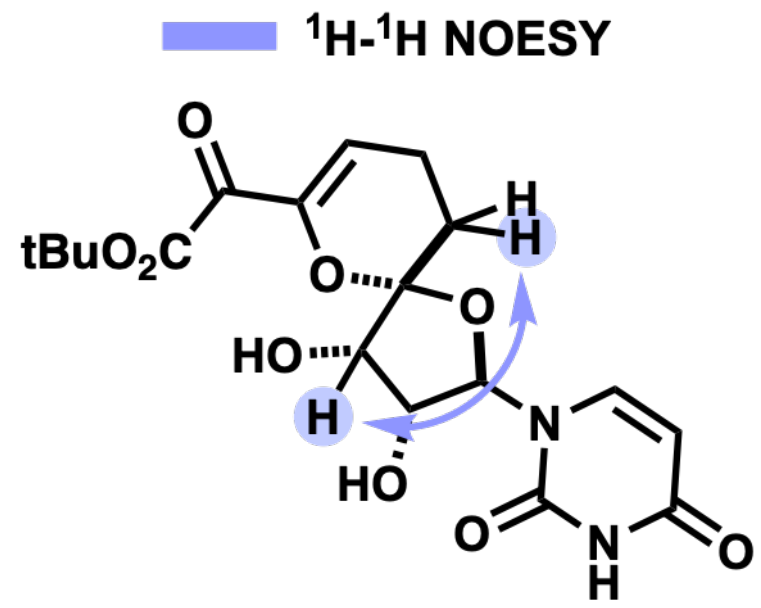

¹H-¹H NOESY
tBuO2C
O
HO
H
HO
N
NH
O

3l ^{1}H NMR (500 MHz, DMSO-d_6)

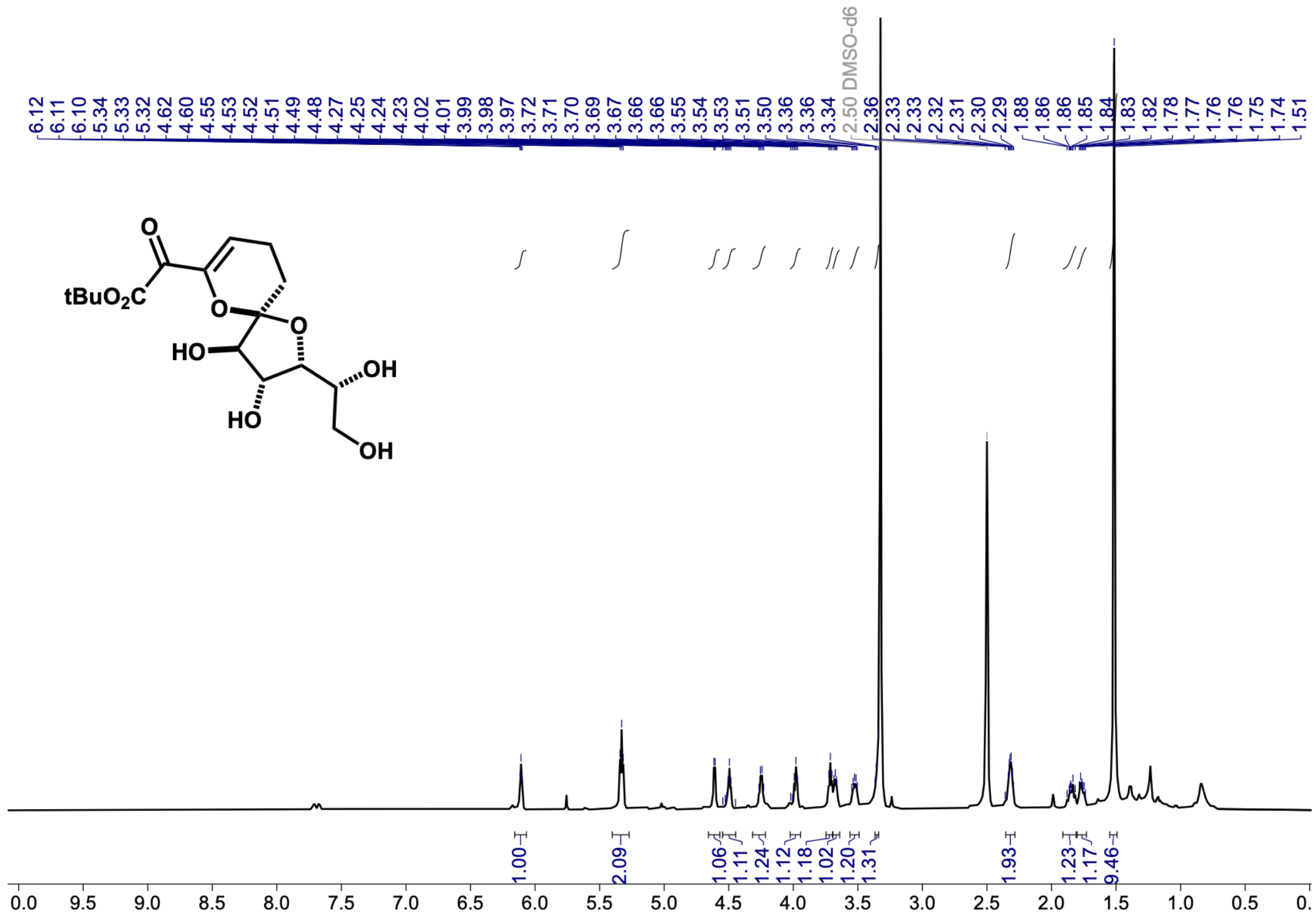

3l ^{13}C NMR (126 MHz, DMSO-d_6)

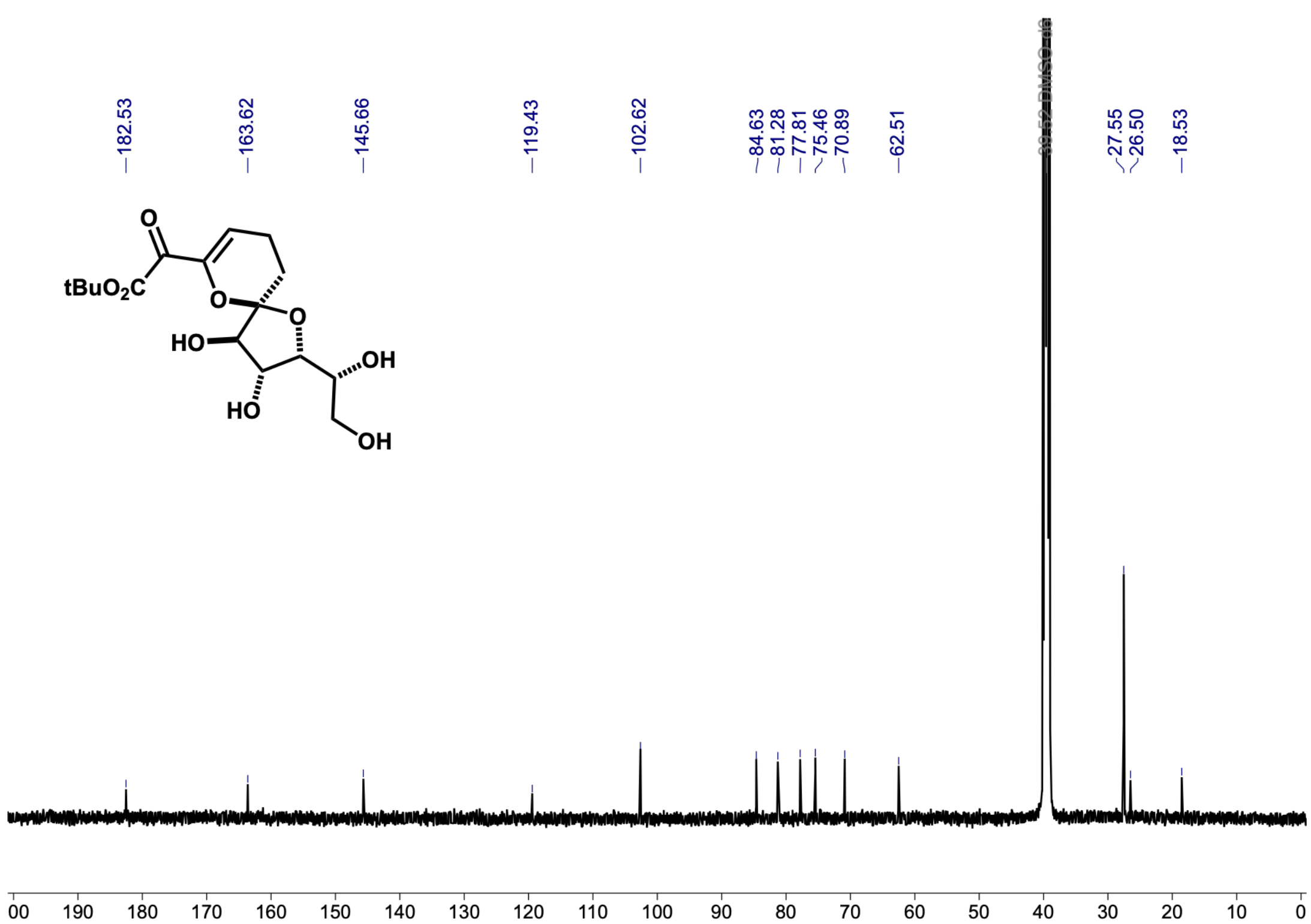

3l ^{1}H-^{1}H COSY (500 MHz, DMSO-d_6)

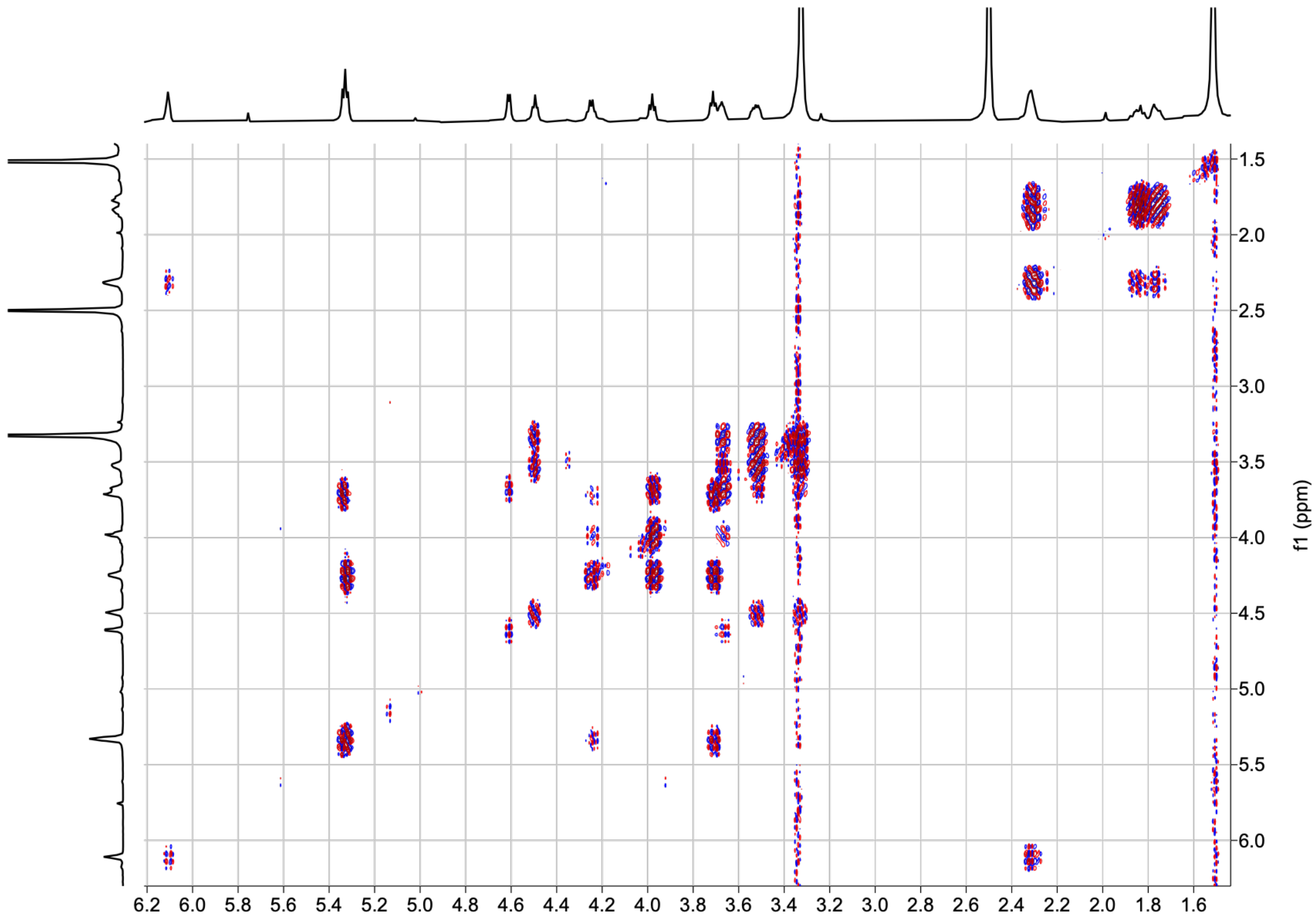

3l ^{1}H-^{1}H NOESY (500 MHz, DMSO-d_6)

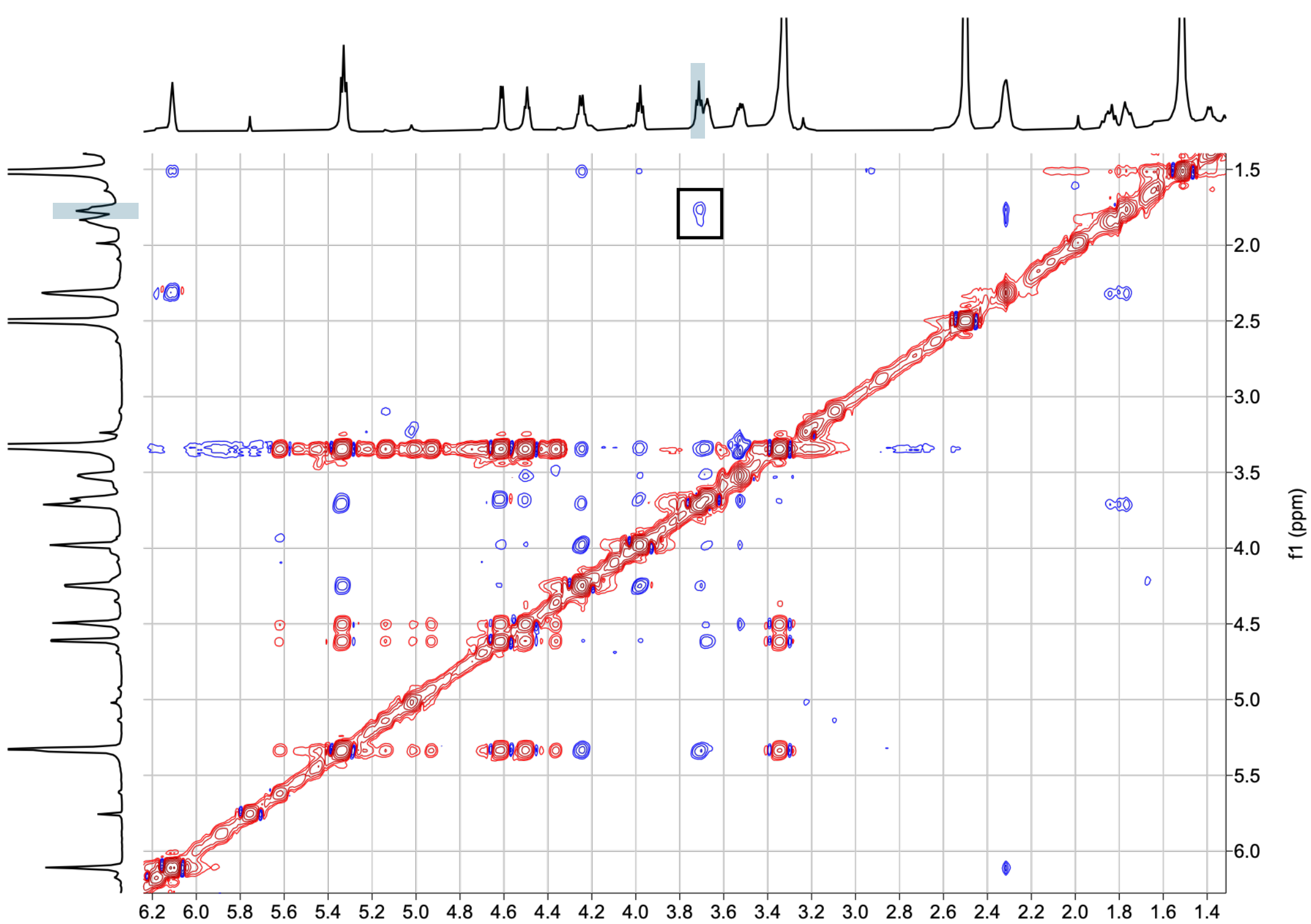

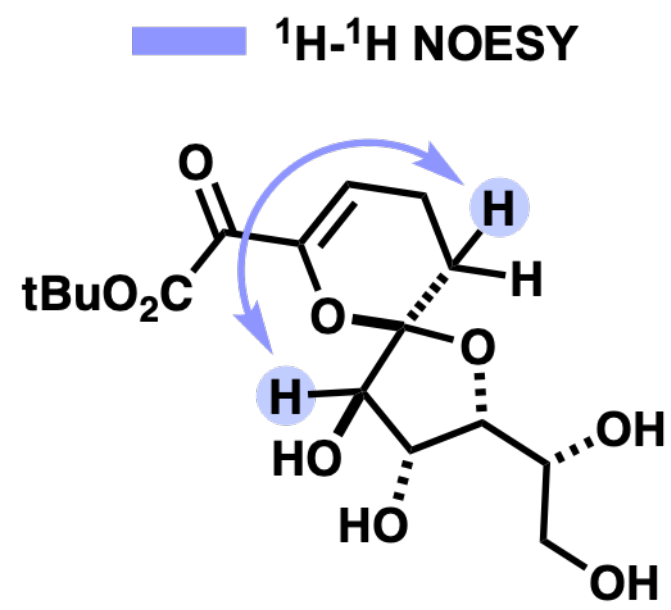
1H-1H NOESY
O
tBuO2C
O
H
H
O
H
HO
HO
OH
OH

3m ^{1}H NMR (500 MHz, Chloroform-*d*)

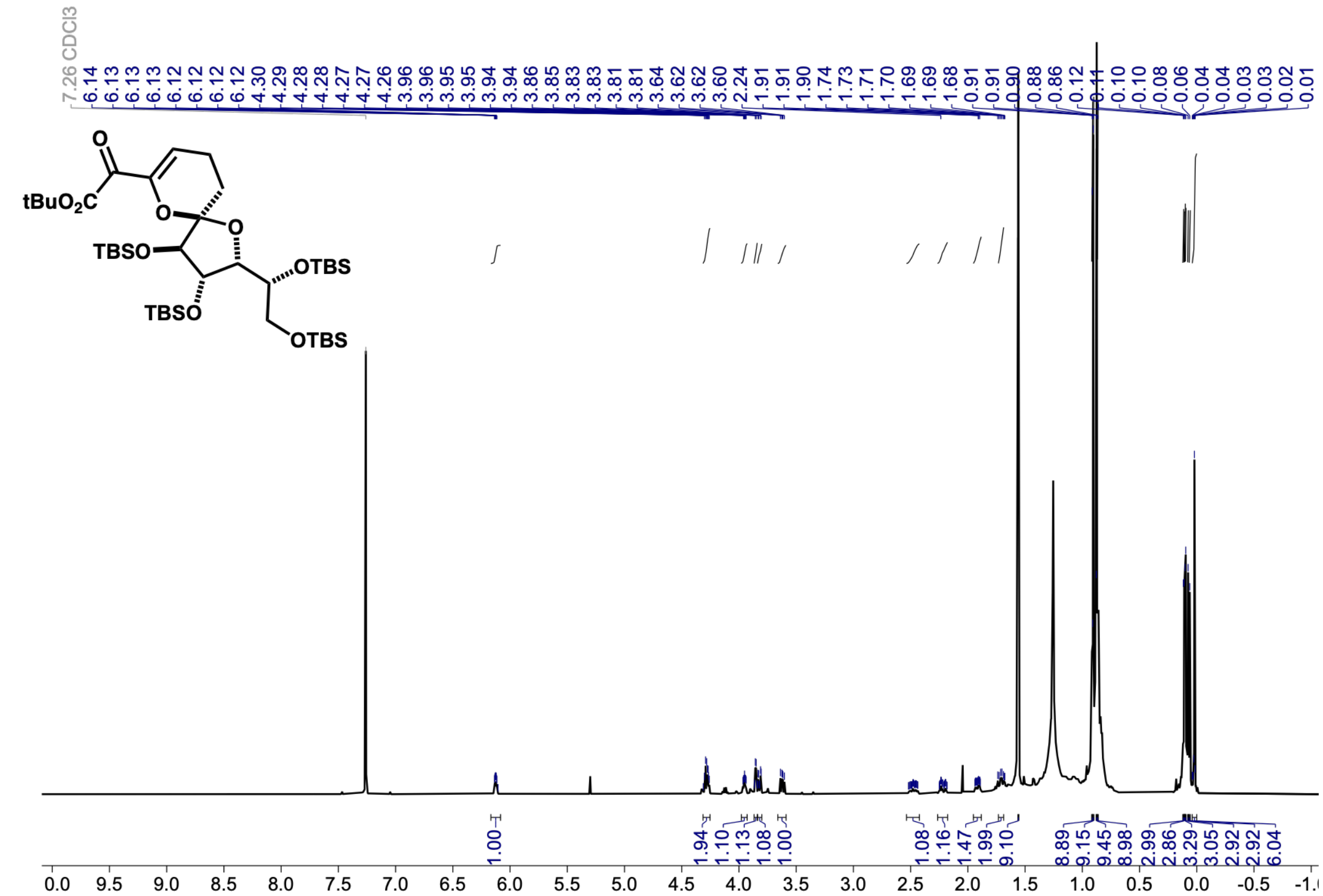

3m ^{13}C NMR (126 MHz, $CDCl_3$)

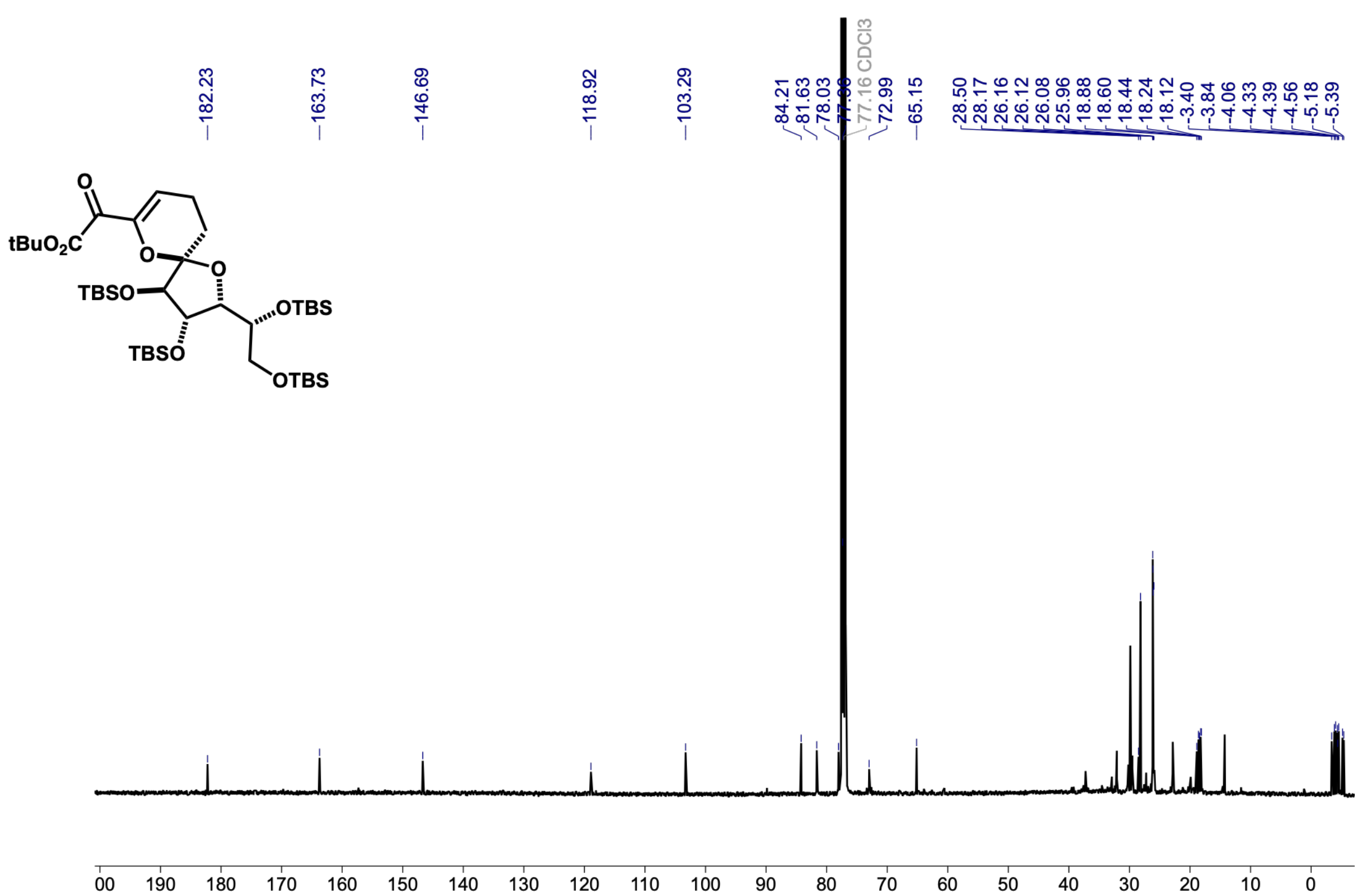

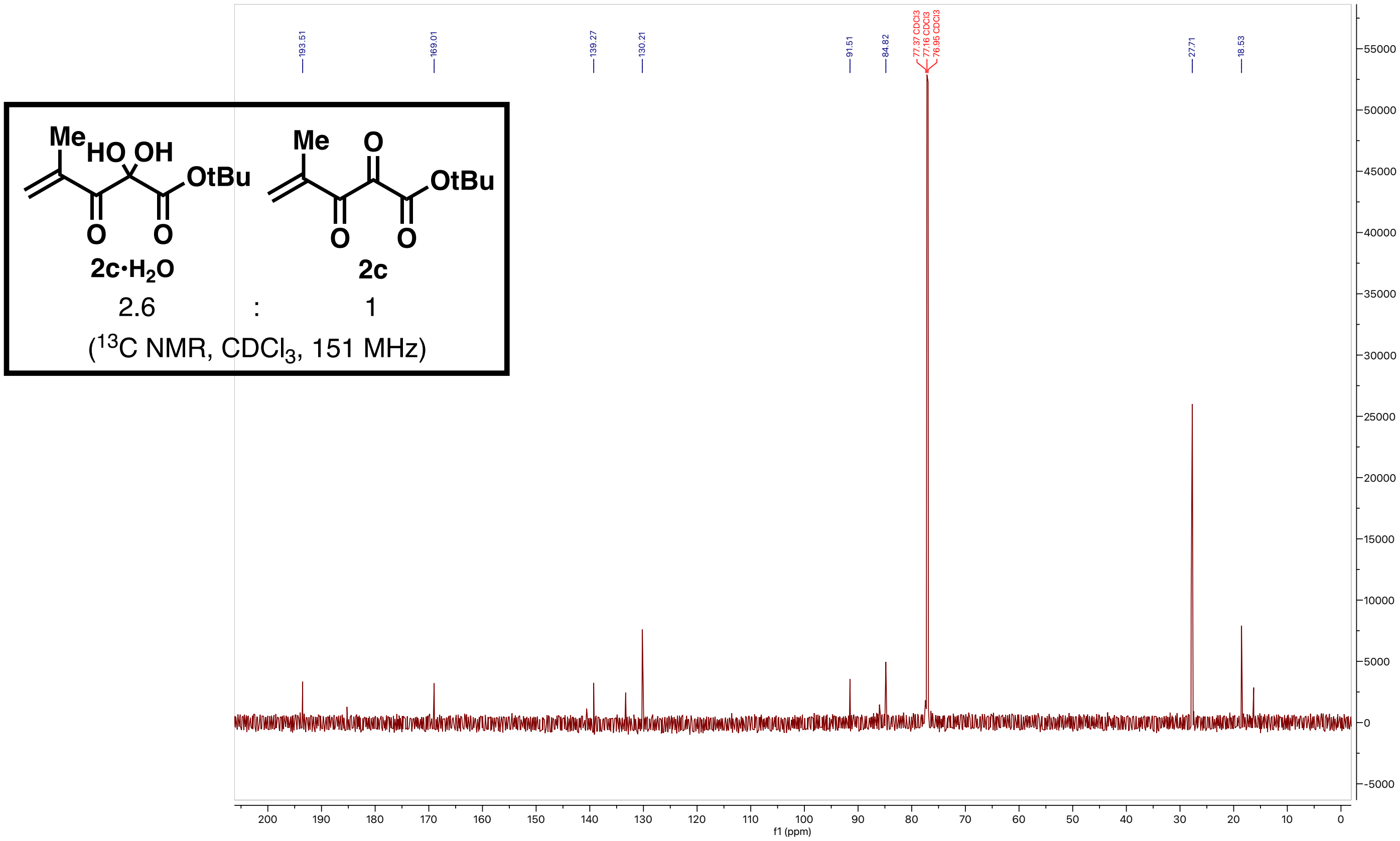
Me HO OH
OtBu
O O
2c·H2O
Me O
OtBu
O O
2c
2.6 : 1
(13C NMR, CDCl3, 151 MHz)
193.51
169.01
139.27
130.21
91.51
84.82
77.37 CDCl3
77.16 CDCl3
76.95 CDCl3
27.71
18.53
f1 (ppm)

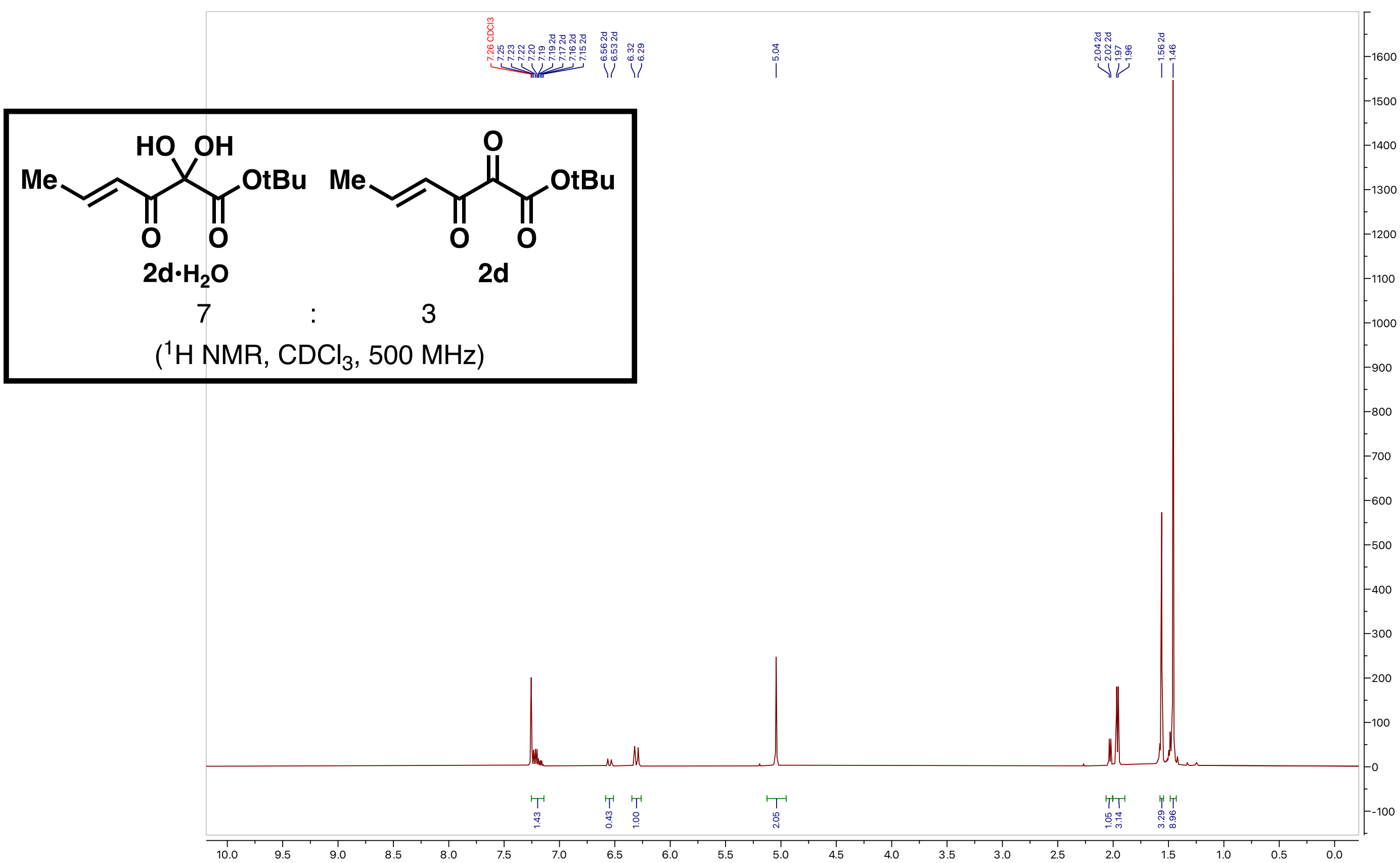
HO OH
Me OtBu
O O
2d·H2O
O
Me OtBu
O O
2d
7 : 3
(1H NMR, CDCl3, 500 MHz)
7.26 CDCl3
7.25
7.23
7.22
7.20
7.19
7.19 2d
7.17 2d
7.16 2d
7.15 2d
6.56 2d
6.53 2d
6.32
6.29
5.04
2.04 2d
2.02 2d
1.97
1.96
1.56 2d
1.46
1.43
0.43
1.00
2.05
1.05
3.14
3.29
8.96
f1 (ppm)

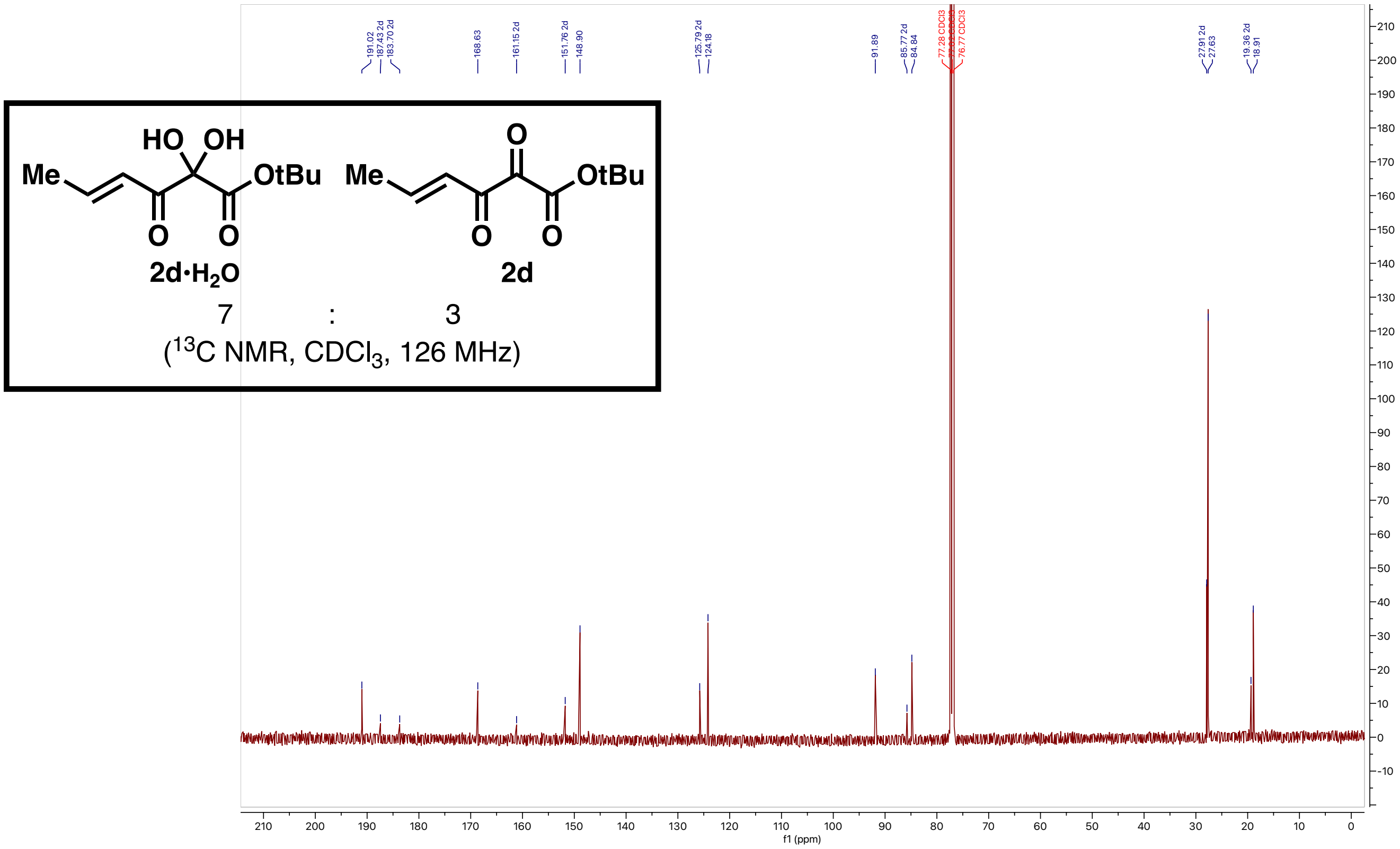
2d·H2O
2d
7 : 3
(13C NMR, CDCl3, 126 MHz)
f1 (ppm)

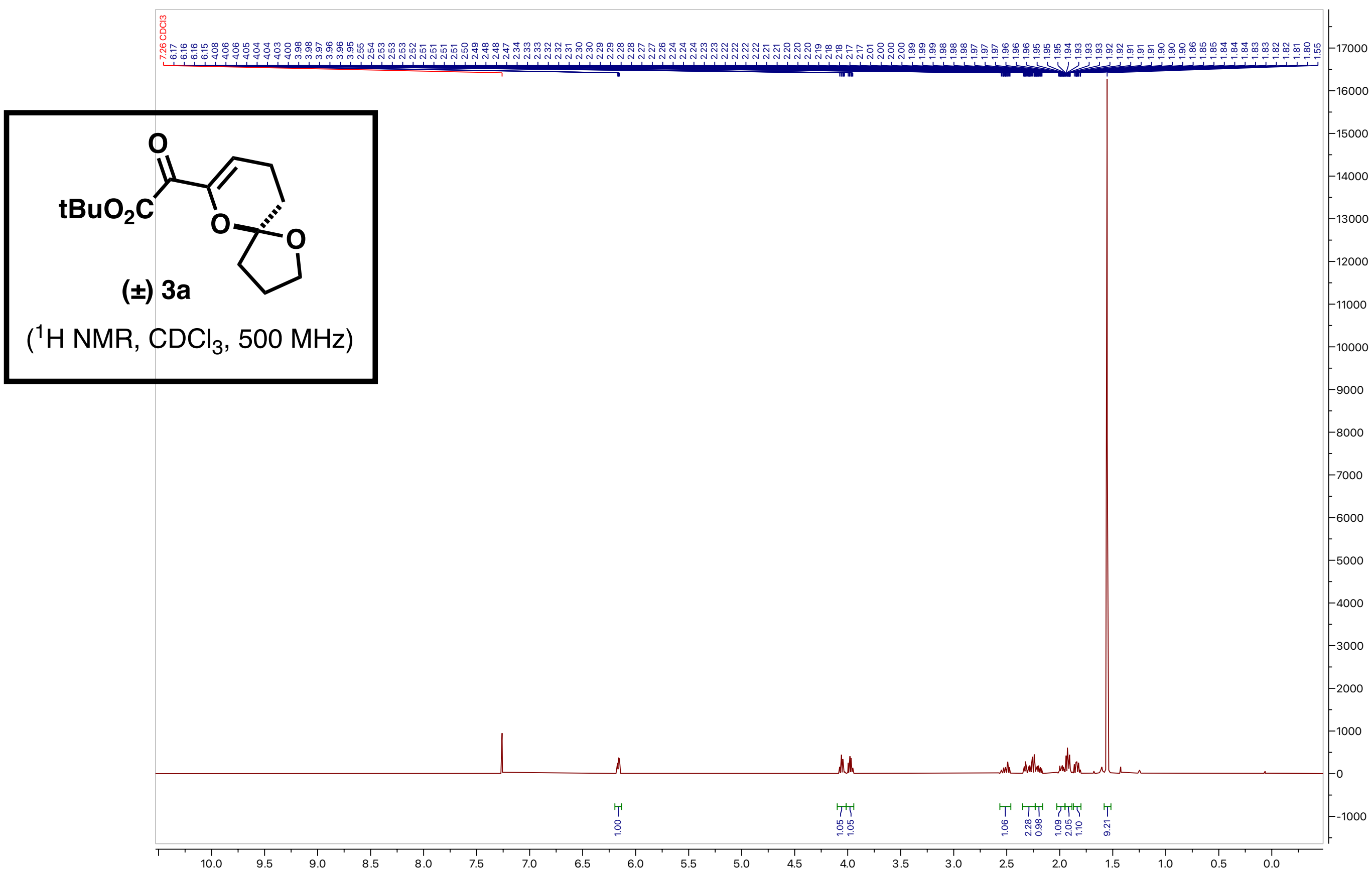
(±) 3a
(1H NMR, CDCl3, 500 MHz)
f1 (ppm)

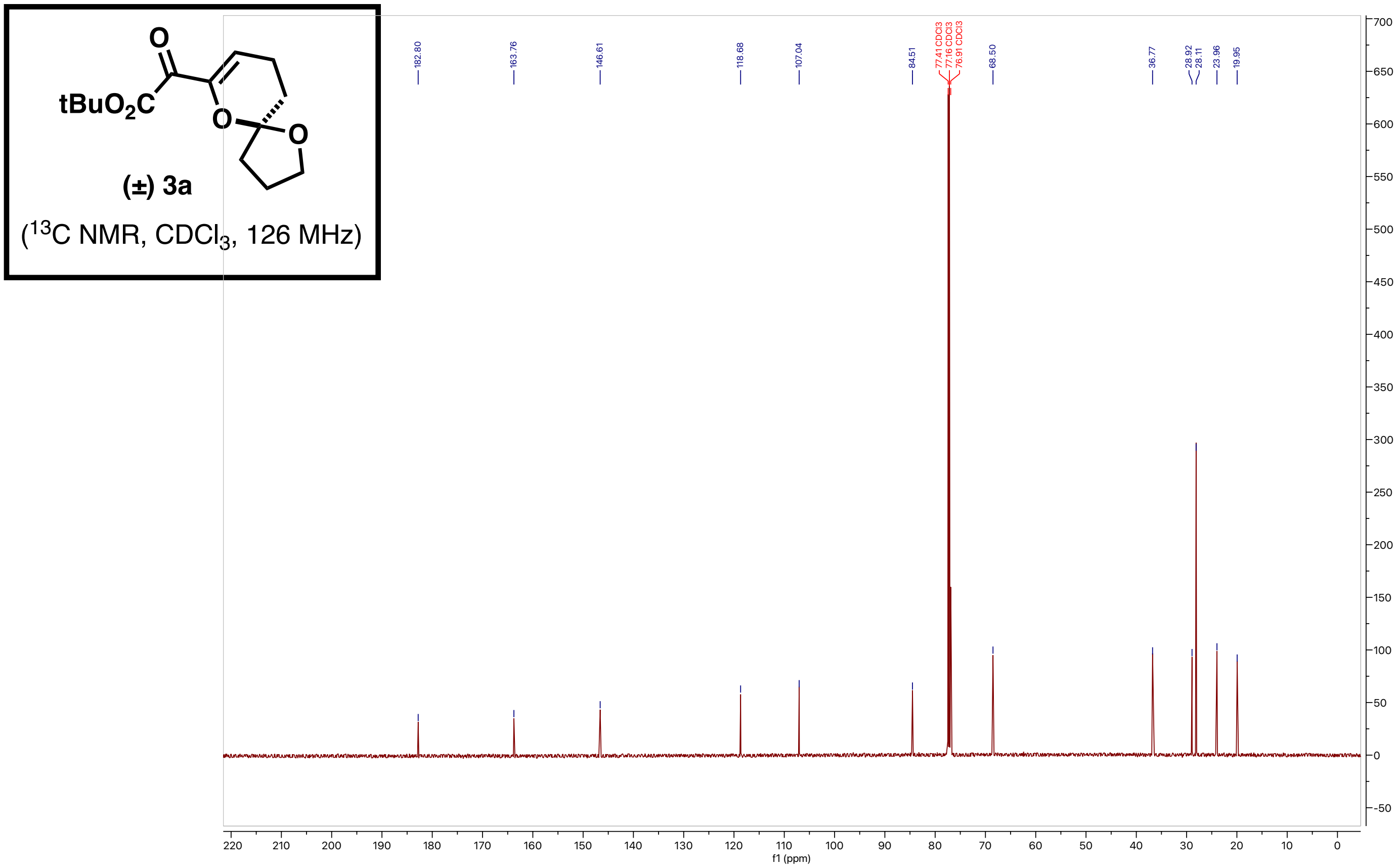
O
tBuO2C
O
O
(±) 3a
(13C NMR, CDCl3, 126 MHz)
182.80
163.76
146.61
118.68
107.04
84.51
77.41 CDCl3
77.16 CDCl3
76.91 CDCl3
68.50
36.77
28.92
28.11
23.96
19.95
f1 (ppm)

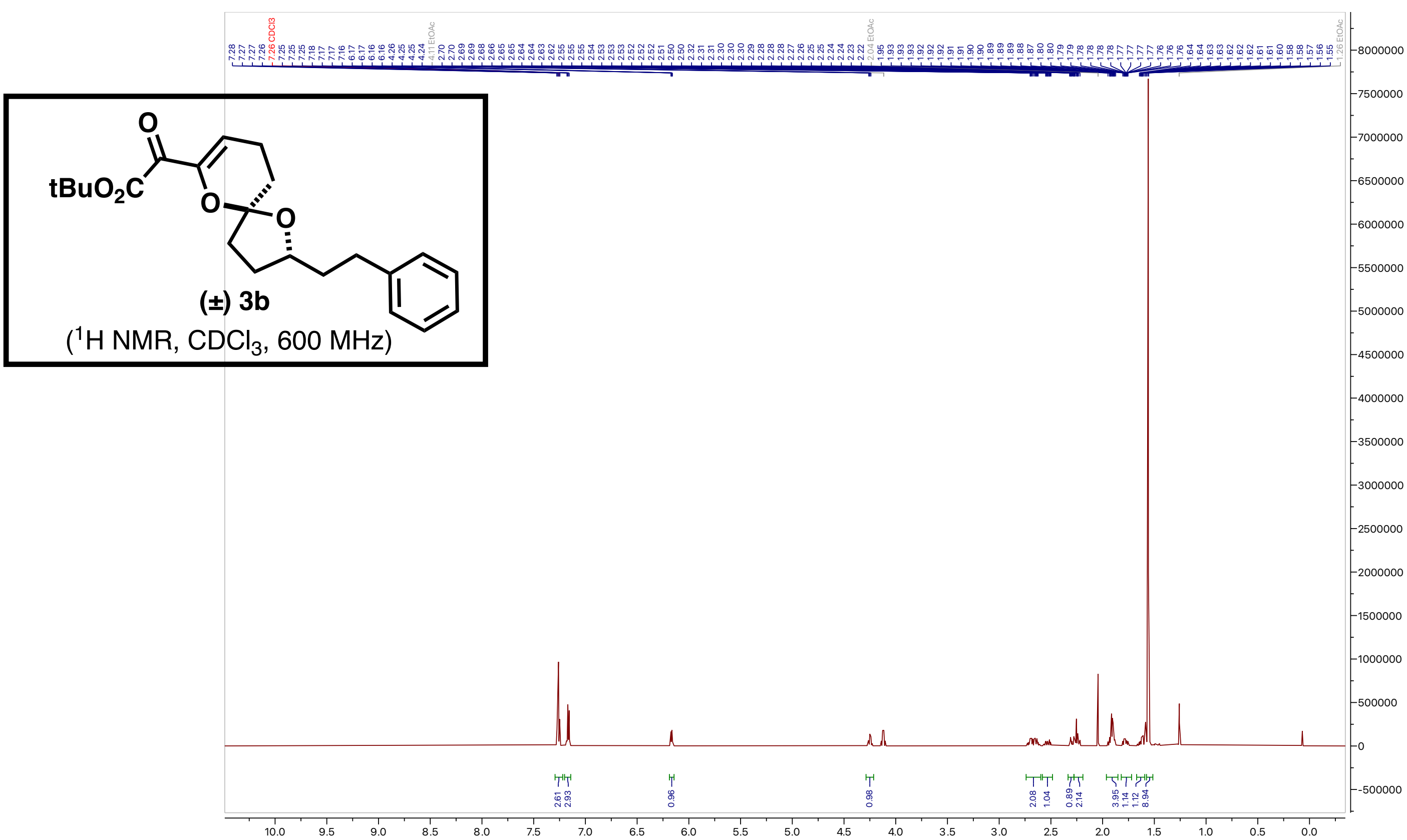
O
tBuO2C
O
O
(±) 3b
(1H NMR, CDCl3, 600 MHz)
2.61
2.93
0.96
0.98
2.08
1.04
0.89
2.14
3.95
1.14
1.12
8.94
f1 (ppm)

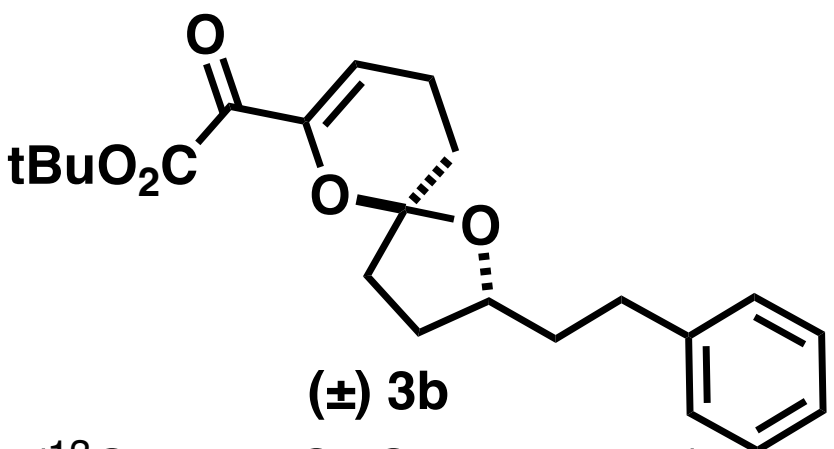

(±) 3b

(^{13}C NMR, CDCl$_3$, 151 MHz)

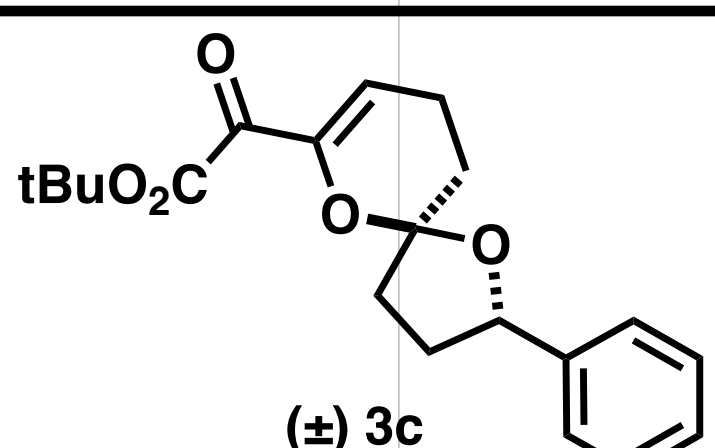

(±) 3c

(^{1}H NMR, CDCl$_3$, 500 MHz)

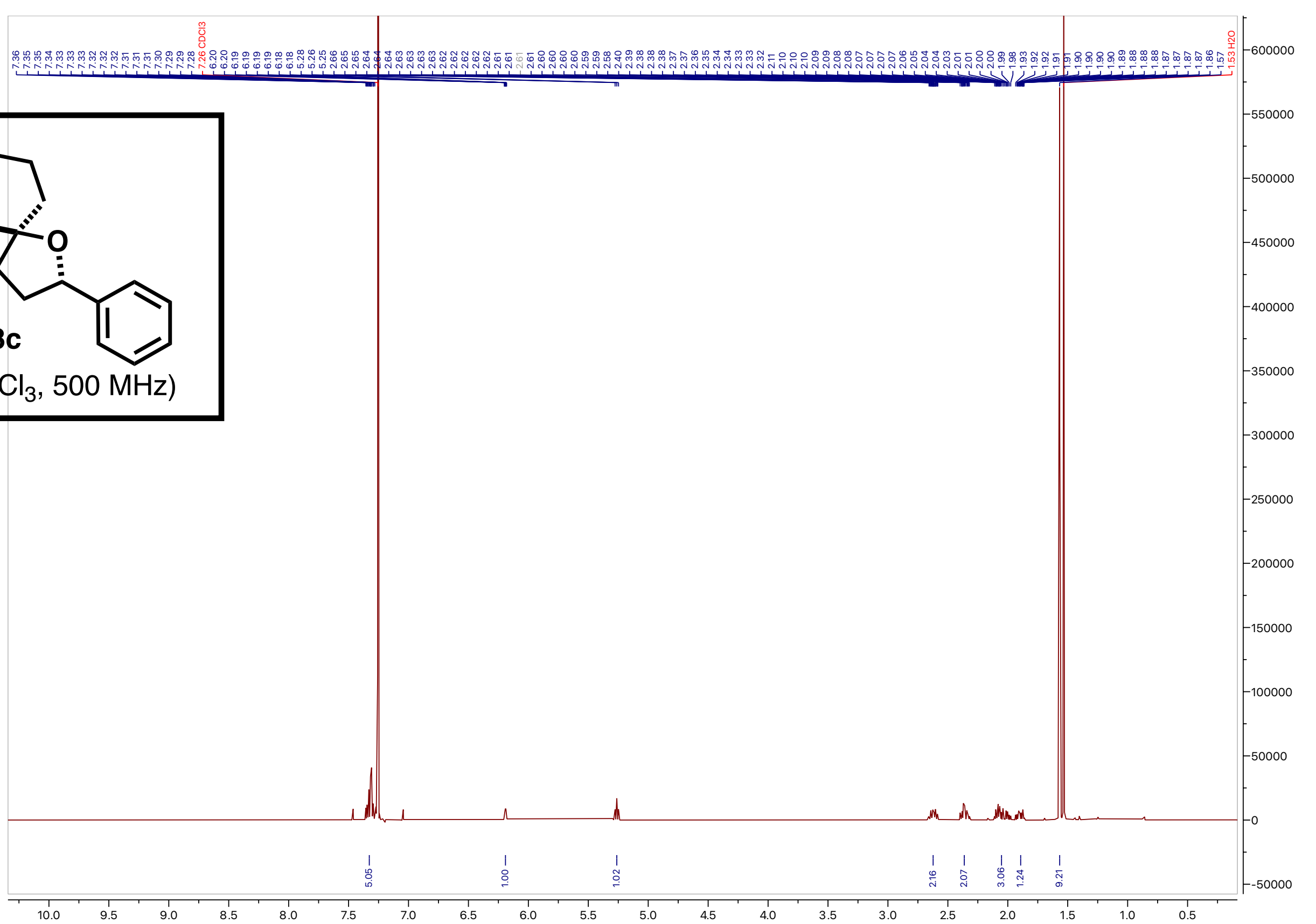

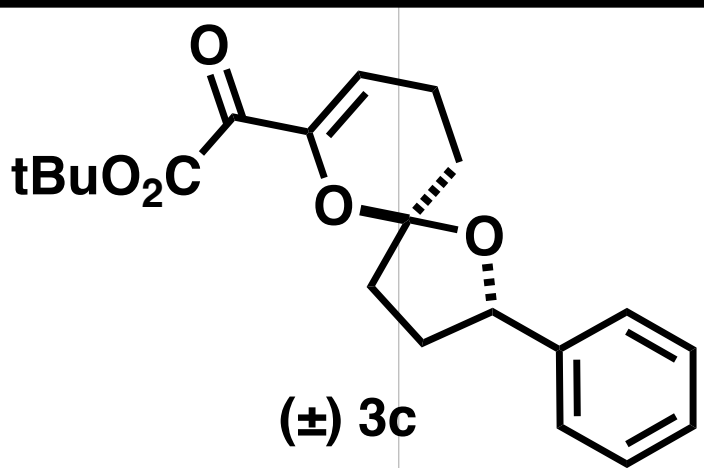

(±) 3c

(^{13}C NMR, $CDCl_3$, 126 MHz)

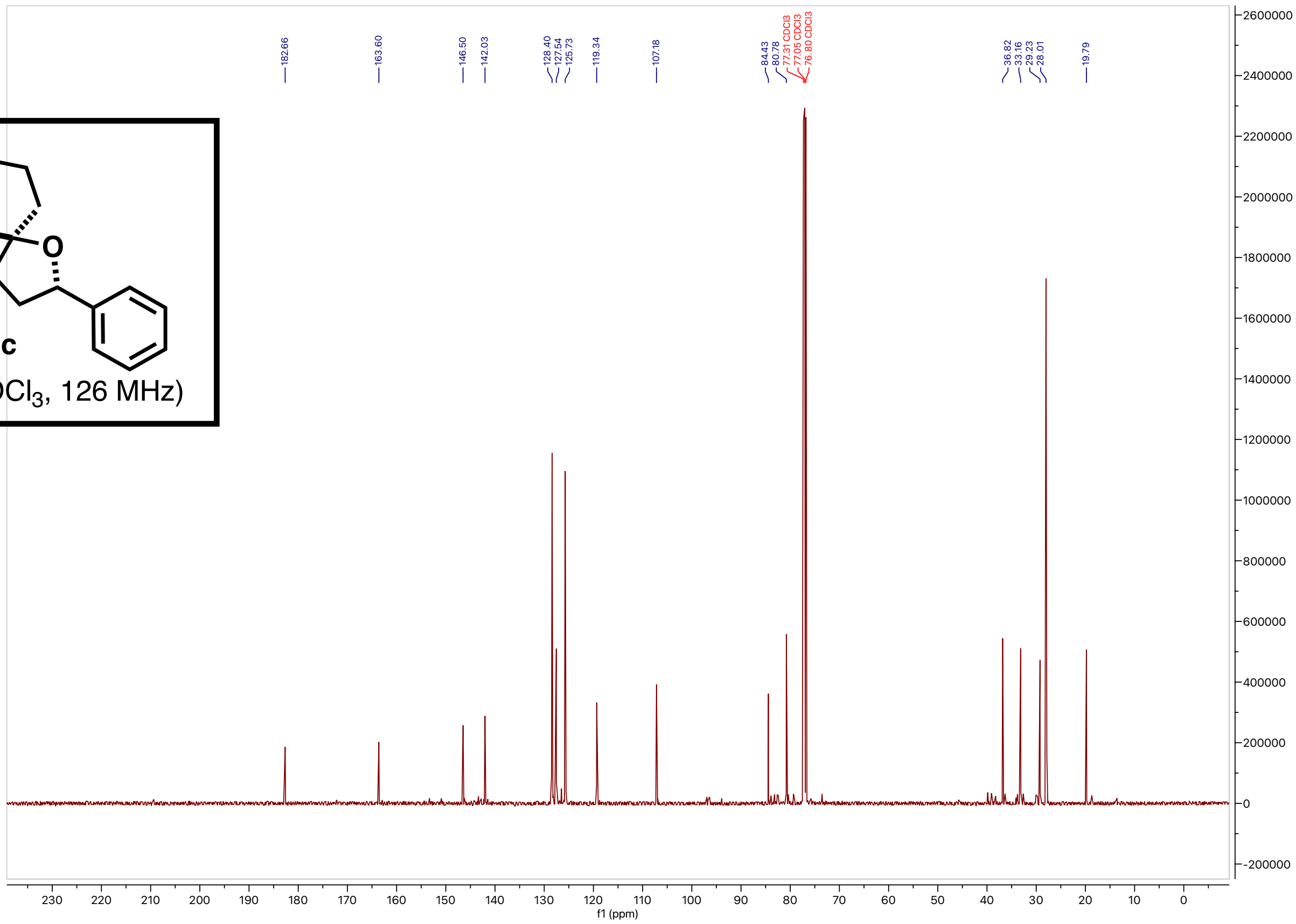

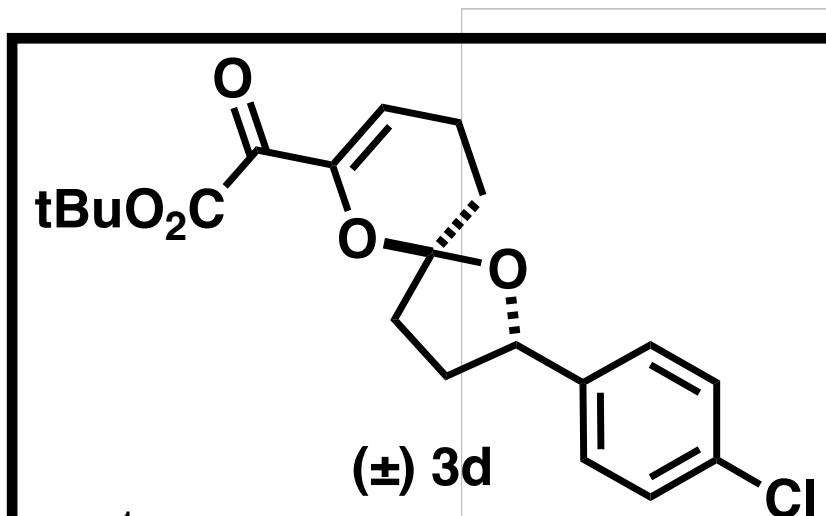

(±) 3d

(^{1}H NMR, $CDCl_3$, 500 MHz)

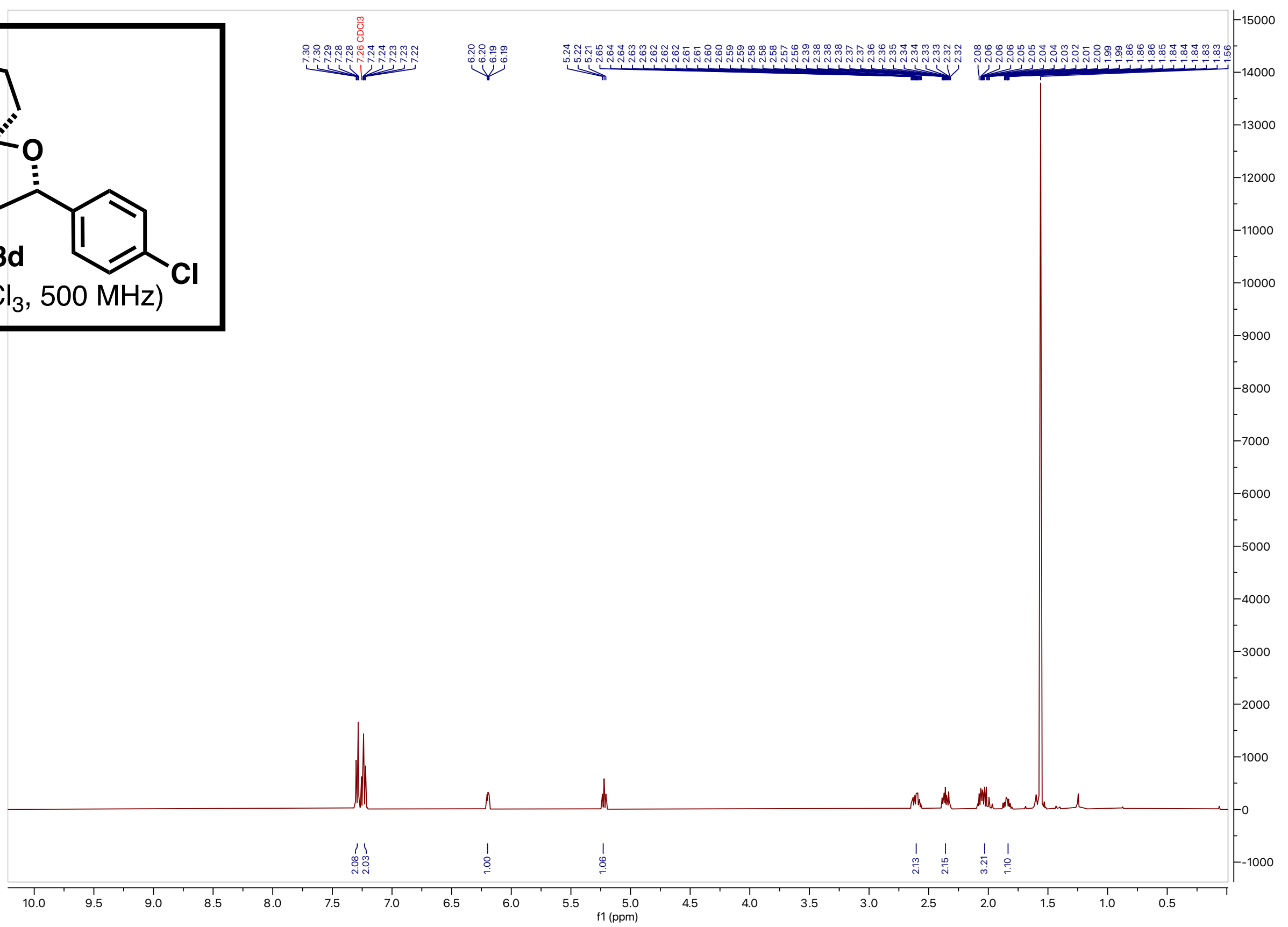

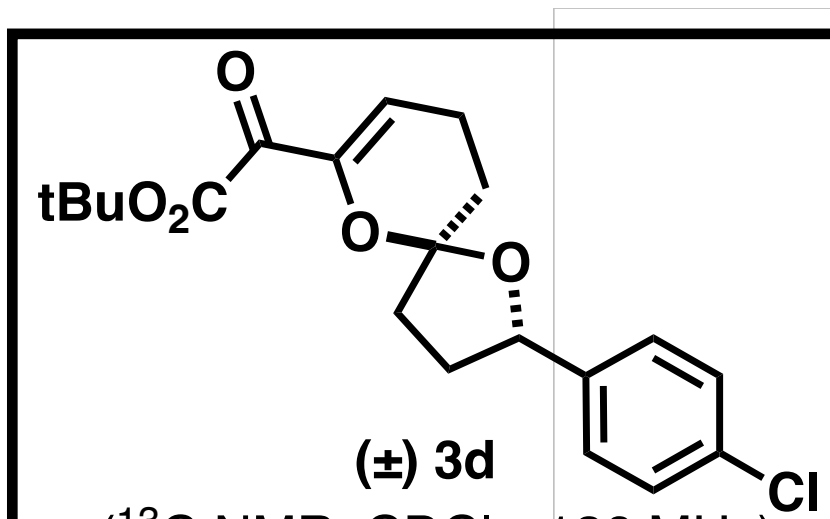

(±) 3d
(^{13}C NMR, $CDCl_3$, 126 MHz)

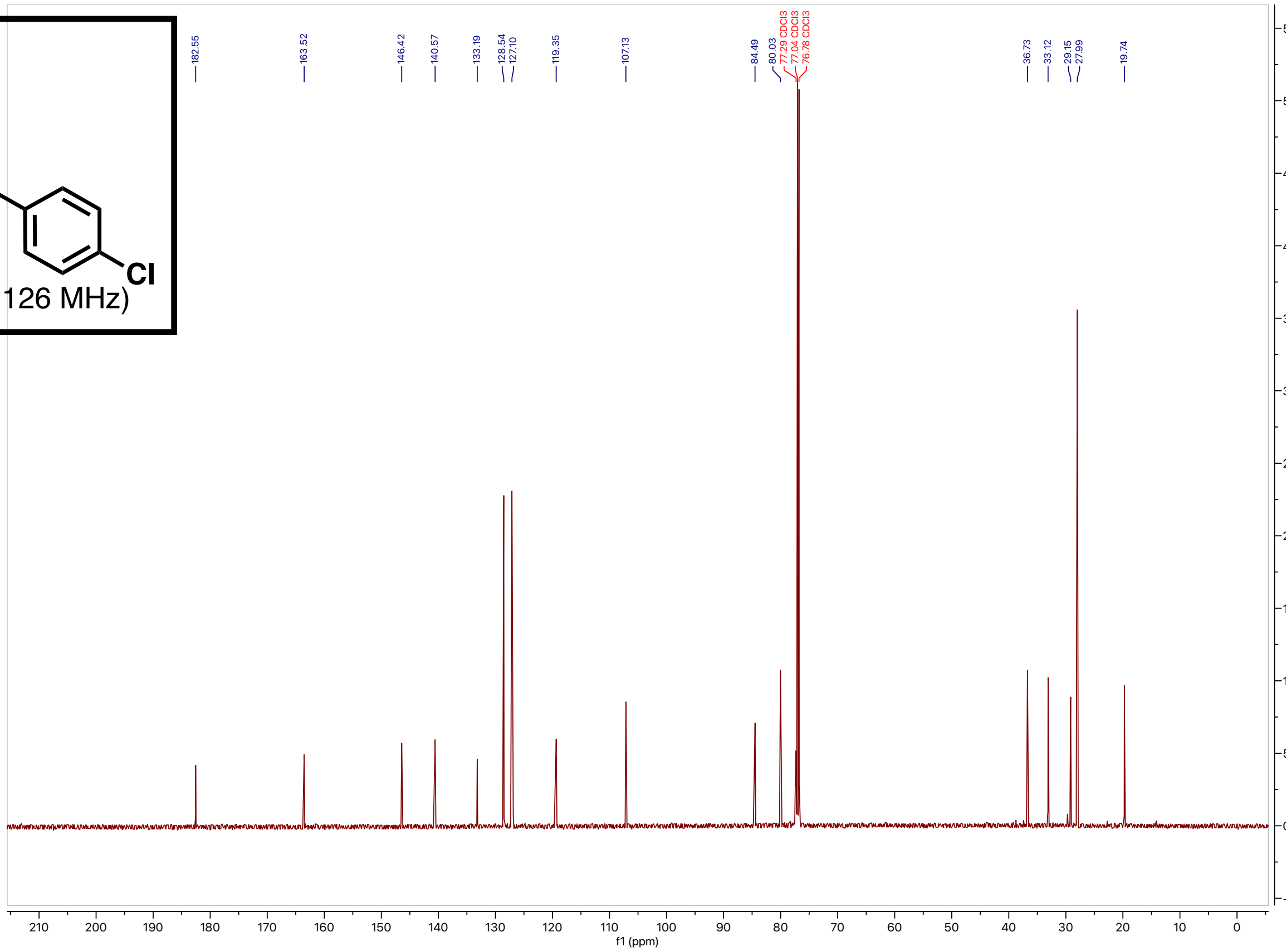

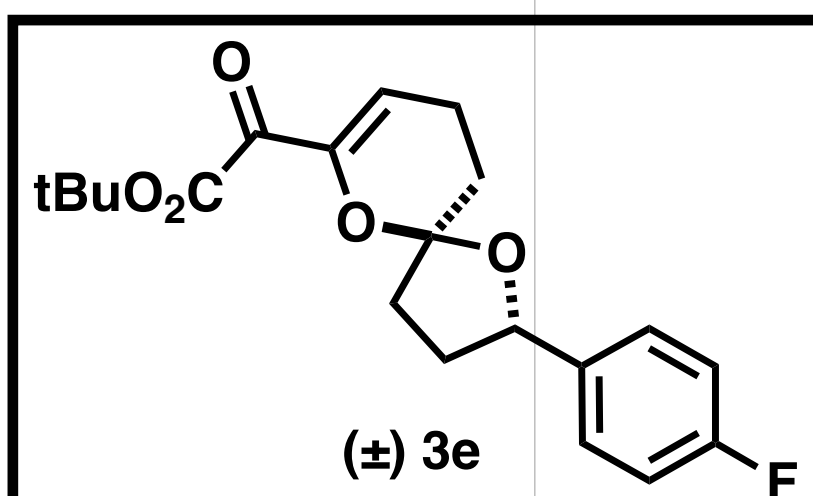

(±) 3e
(^{1}H NMR, $CDCl_3$, 500 MHz)

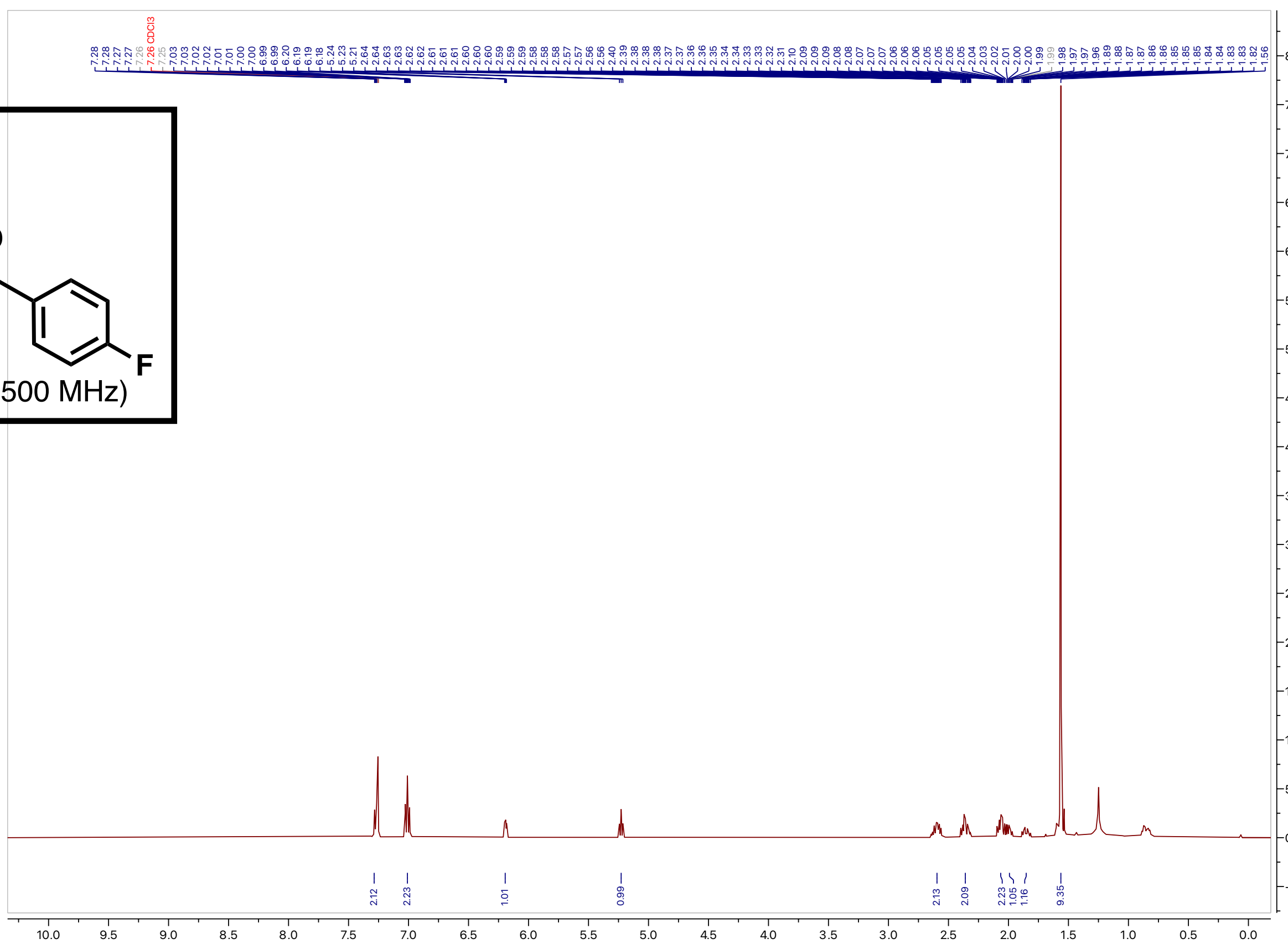

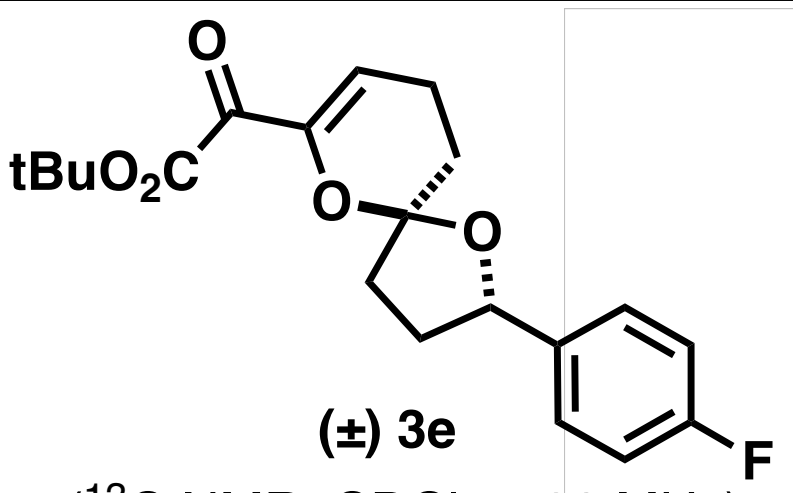

(±) 3e

(^{13}C NMR, $CDCl_3$, 126 MHz)

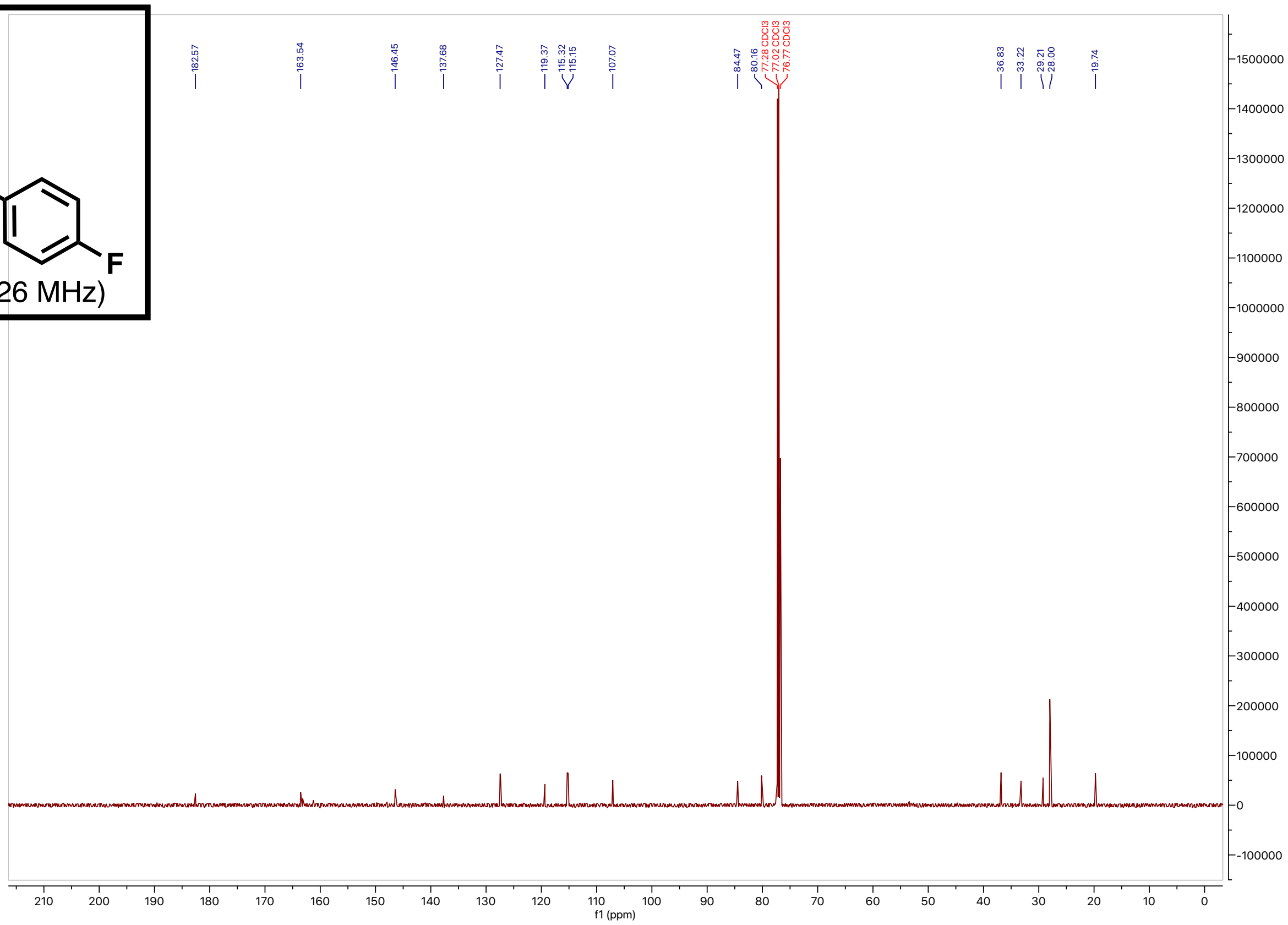

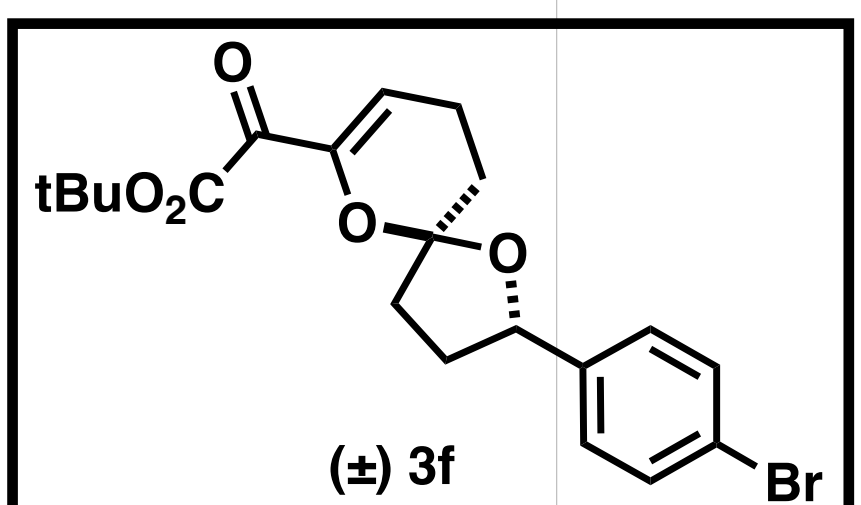

(±) 3f

(^{1}H NMR, $CDCl_3$, 500 MHz)

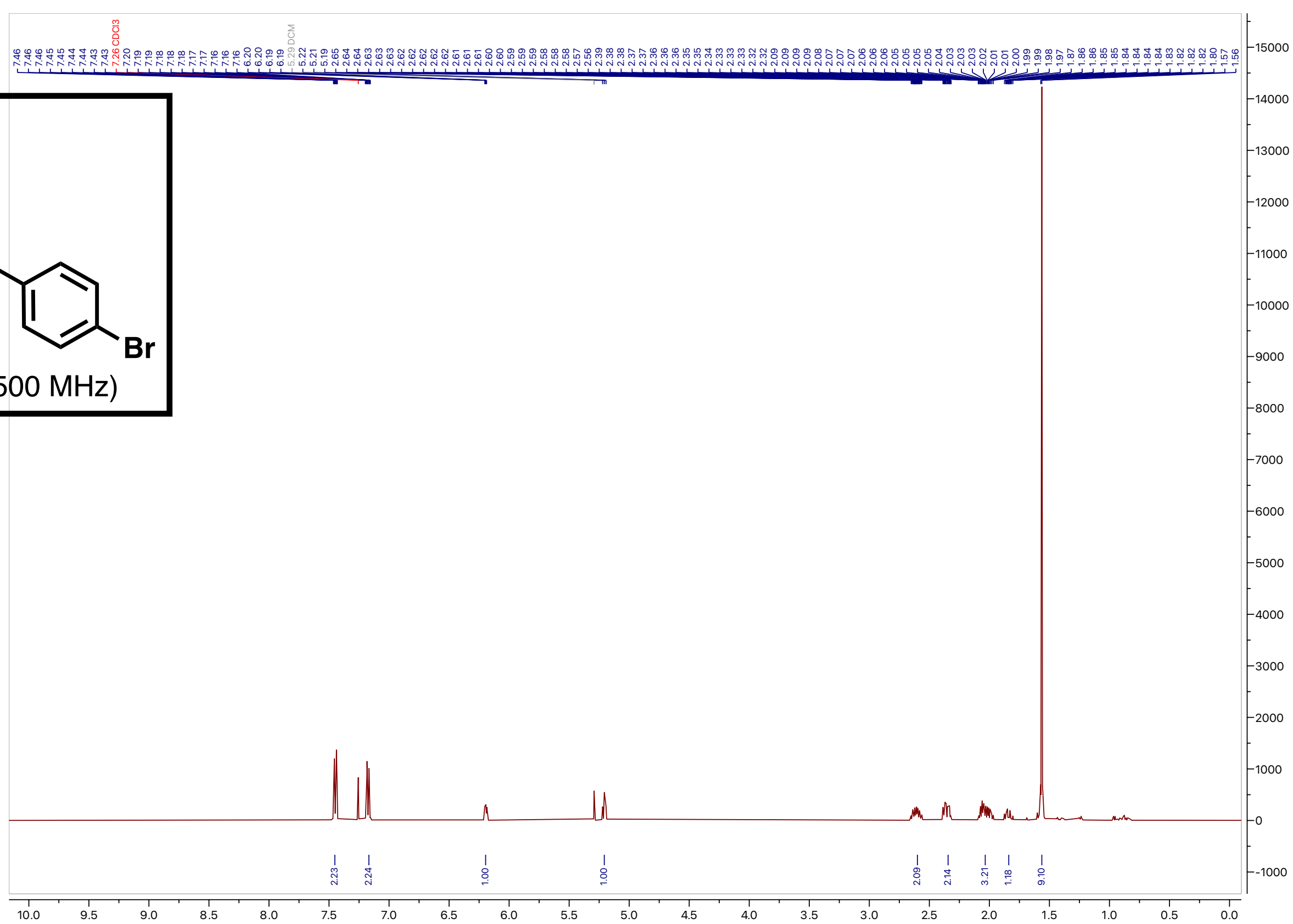

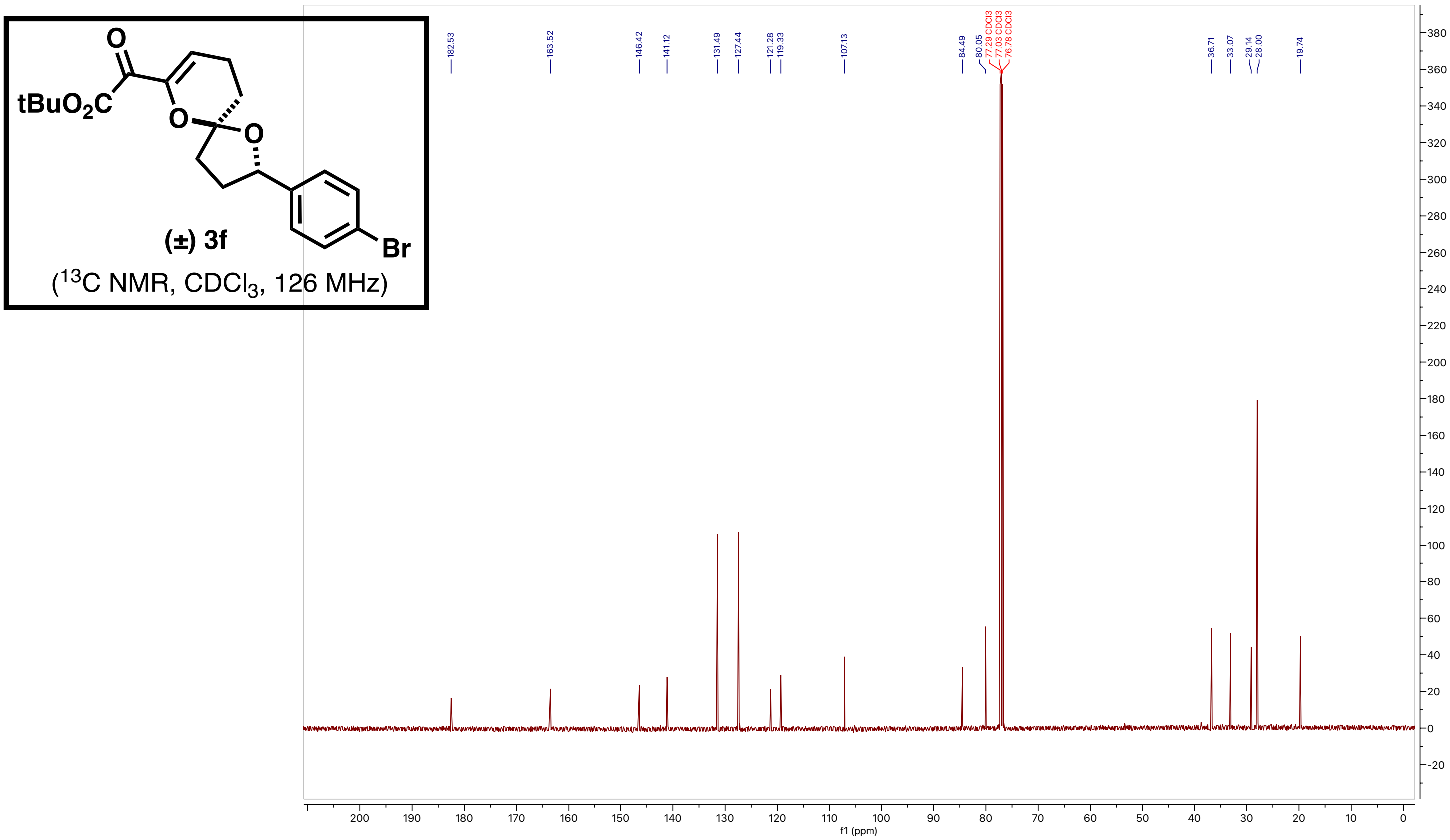
tBuO2C
O
Br
(±) 3f
(13C NMR, CDCl3, 126 MHz)
182.53
163.52
146.42
141.12
131.49
127.44
121.28
119.33
107.13
84.49
80.05
77.29 CDCl3
77.03 CDCl3
76.78 CDCl3
36.71
33.07
29.14
28.00
19.74
f1 (ppm)

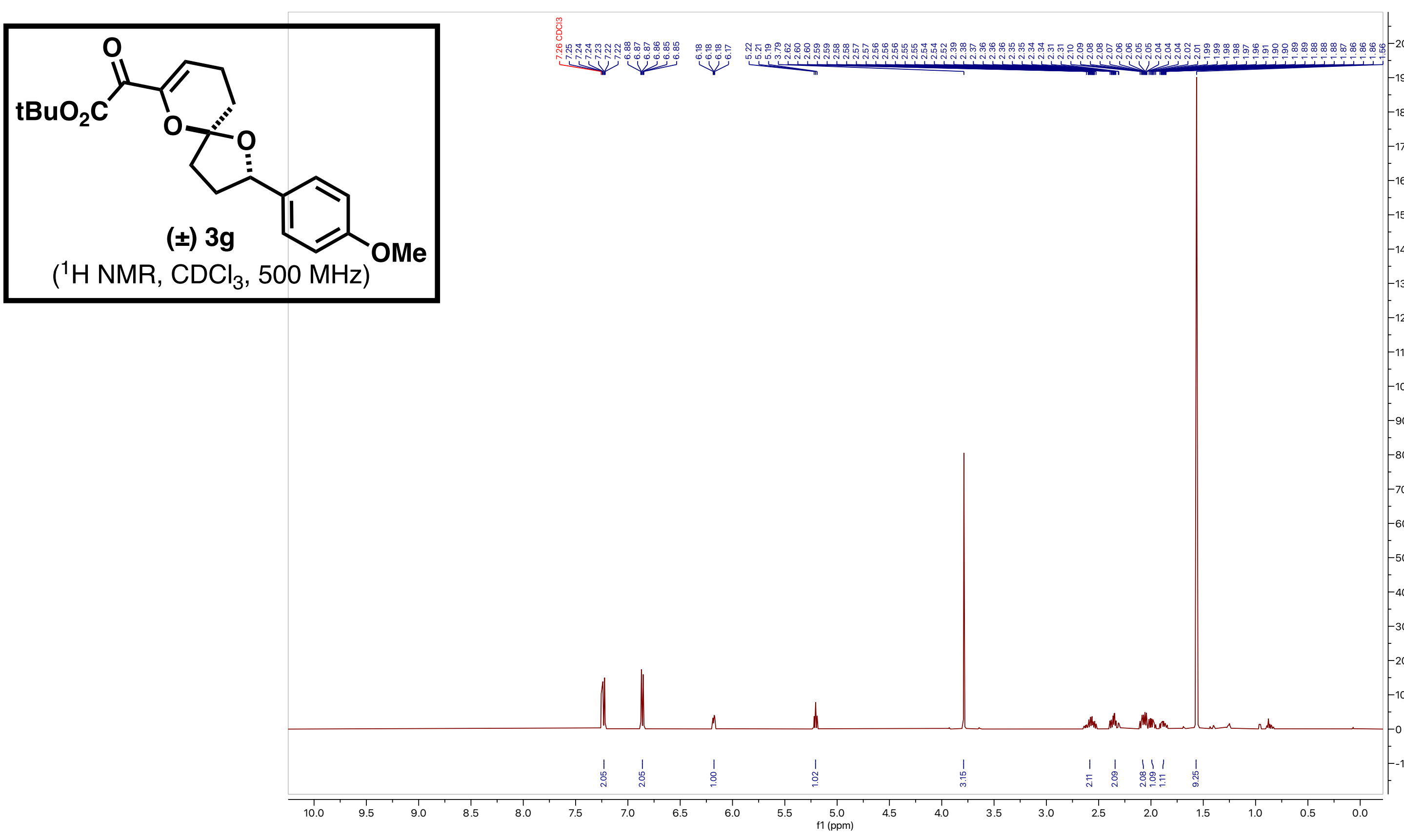
tBuO2C
O
OMe
(±) 3g
(1H NMR, CDCl3, 500 MHz)
7.26 CDCl3
2.05
2.05
1.00
1.02
3.15
2.11
2.09
2.08
1.09
1.11
9.25
f1 (ppm)

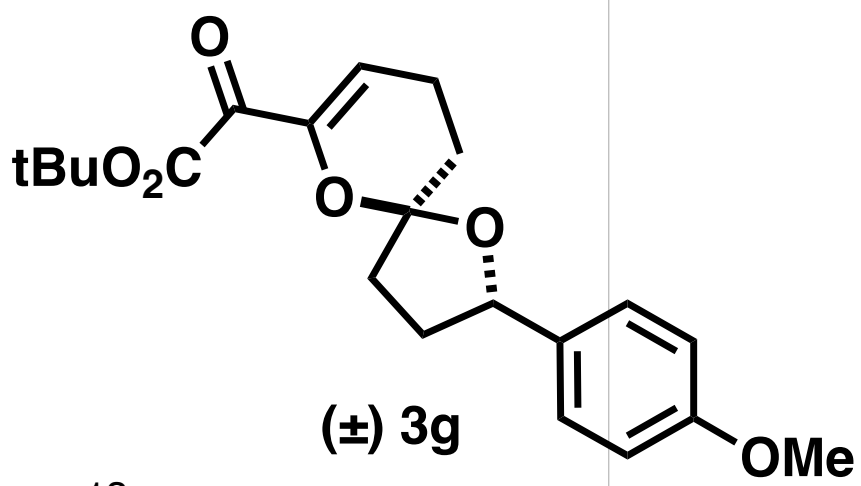

(±) 3g

(^{13}C NMR, $CDCl_3$, 126 MHz)

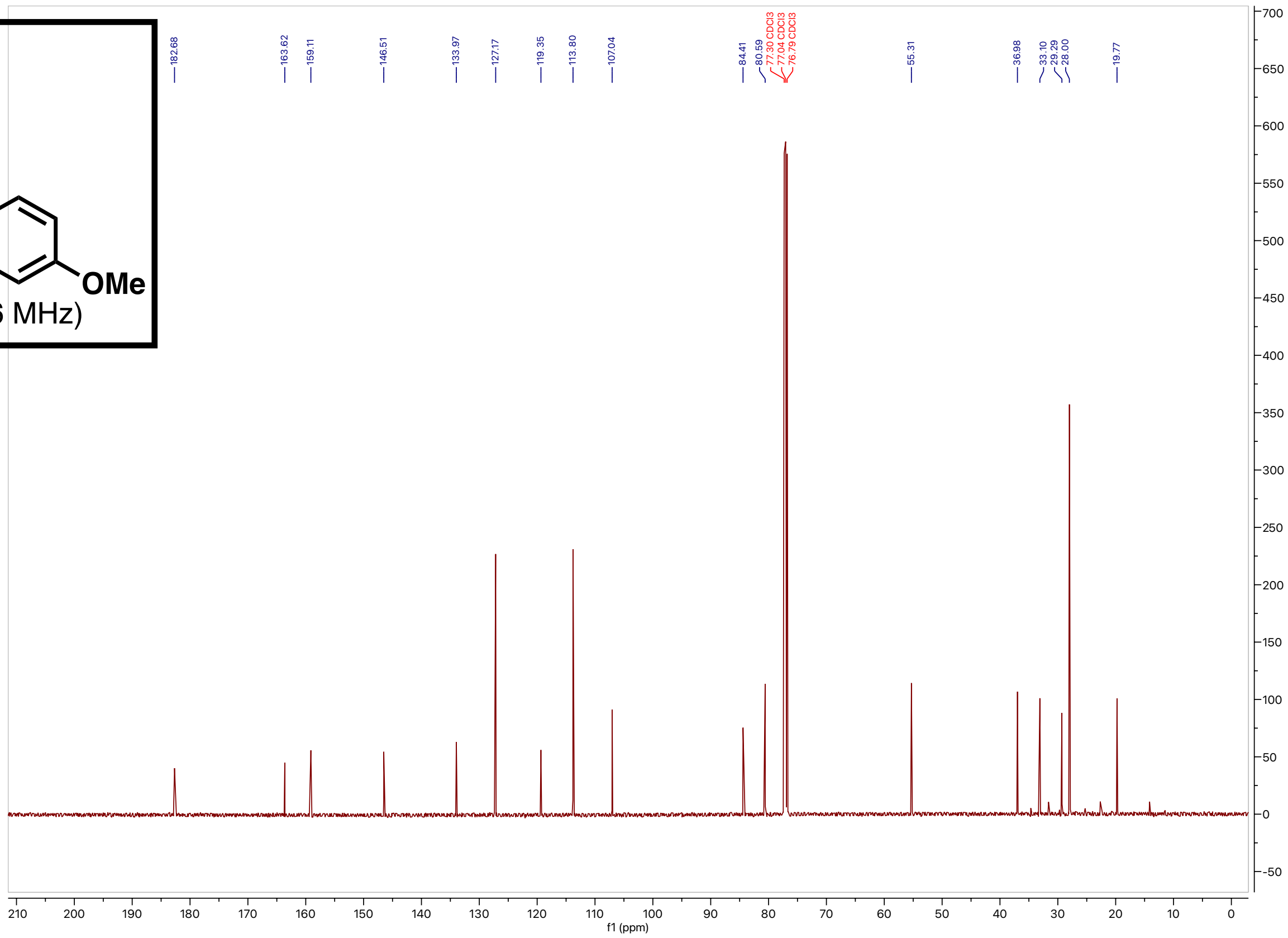

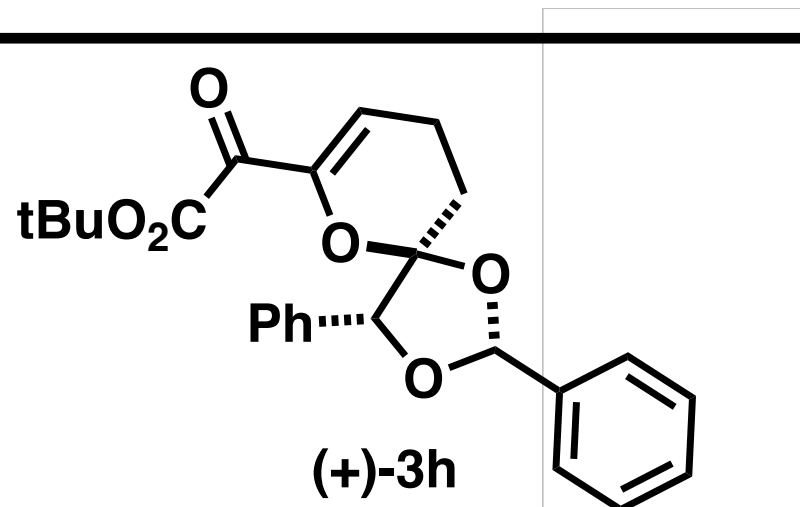

(+)-3h

(^{1}H NMR, $CDCl_3$, 500 MHz)

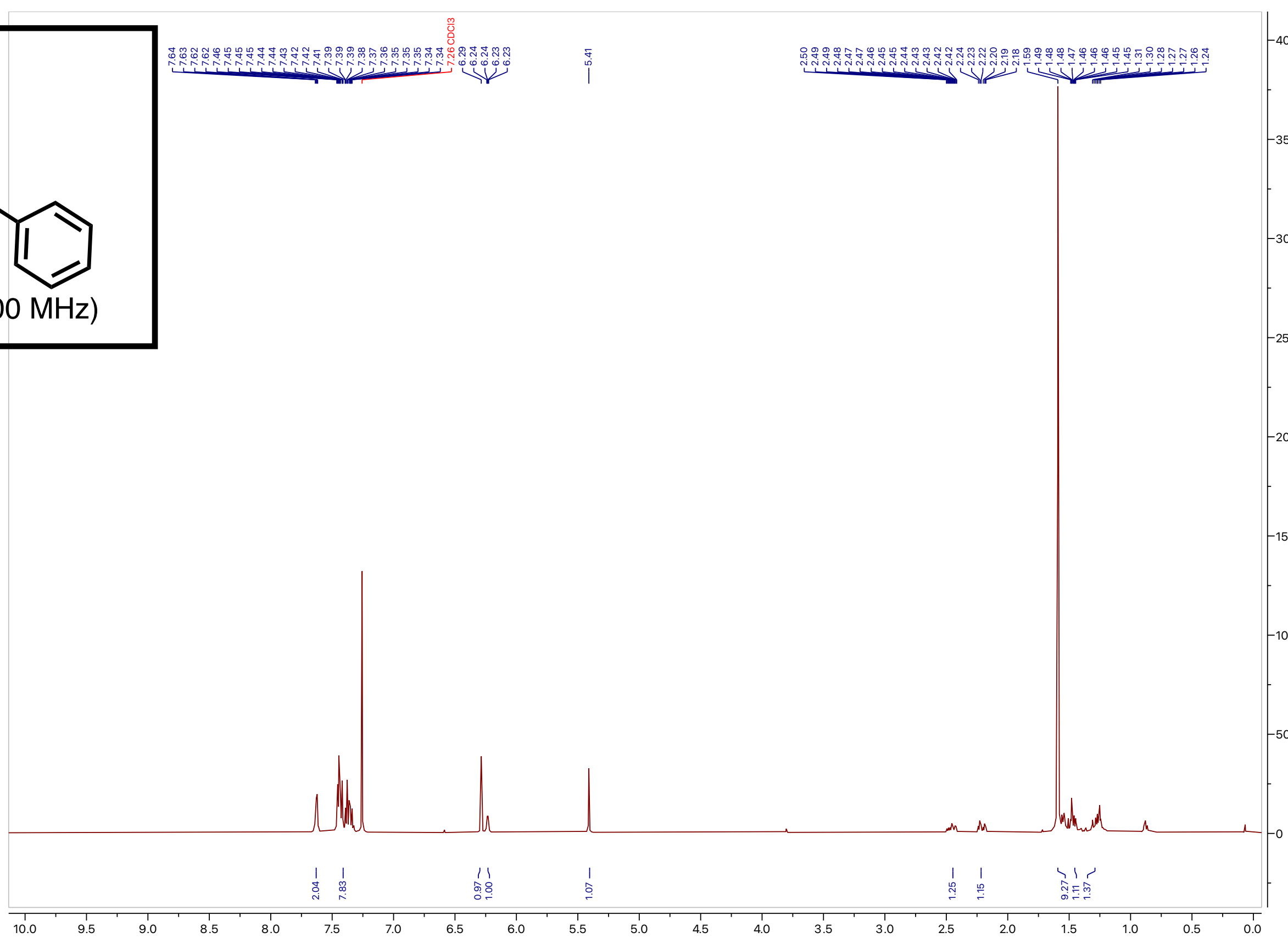

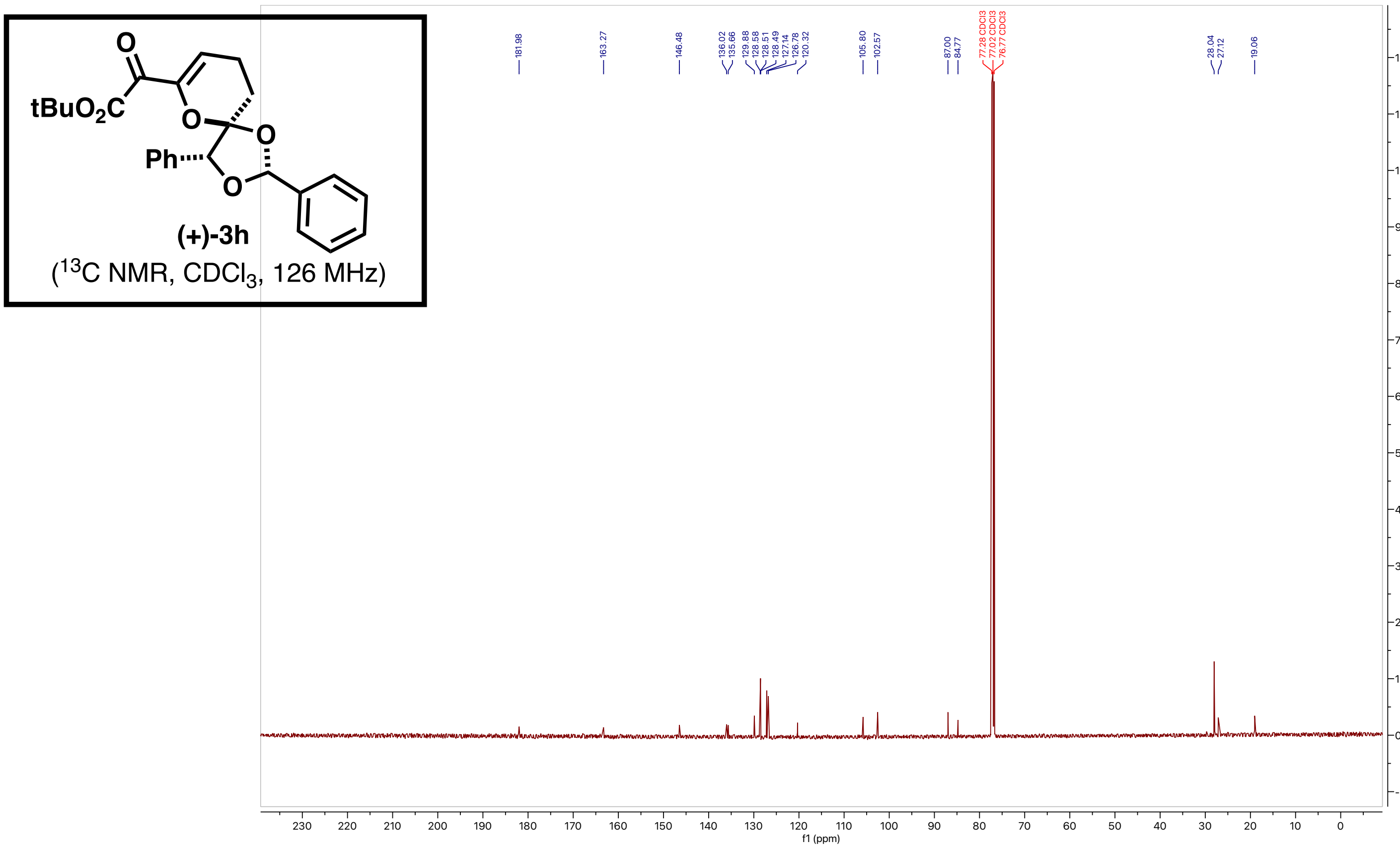
tBuO2C
Ph
(+)-3h
(13C NMR, CDCl3, 126 MHz)
181.98
163.27
146.48
136.02
135.66
129.88
128.58
128.51
128.49
127.14
126.78
120.32
105.80
102.57
87.00
84.77
77.28 CDCl3
77.02 CDCl3
76.77 CDCl3
28.04
27.12
19.06
f1 (ppm)

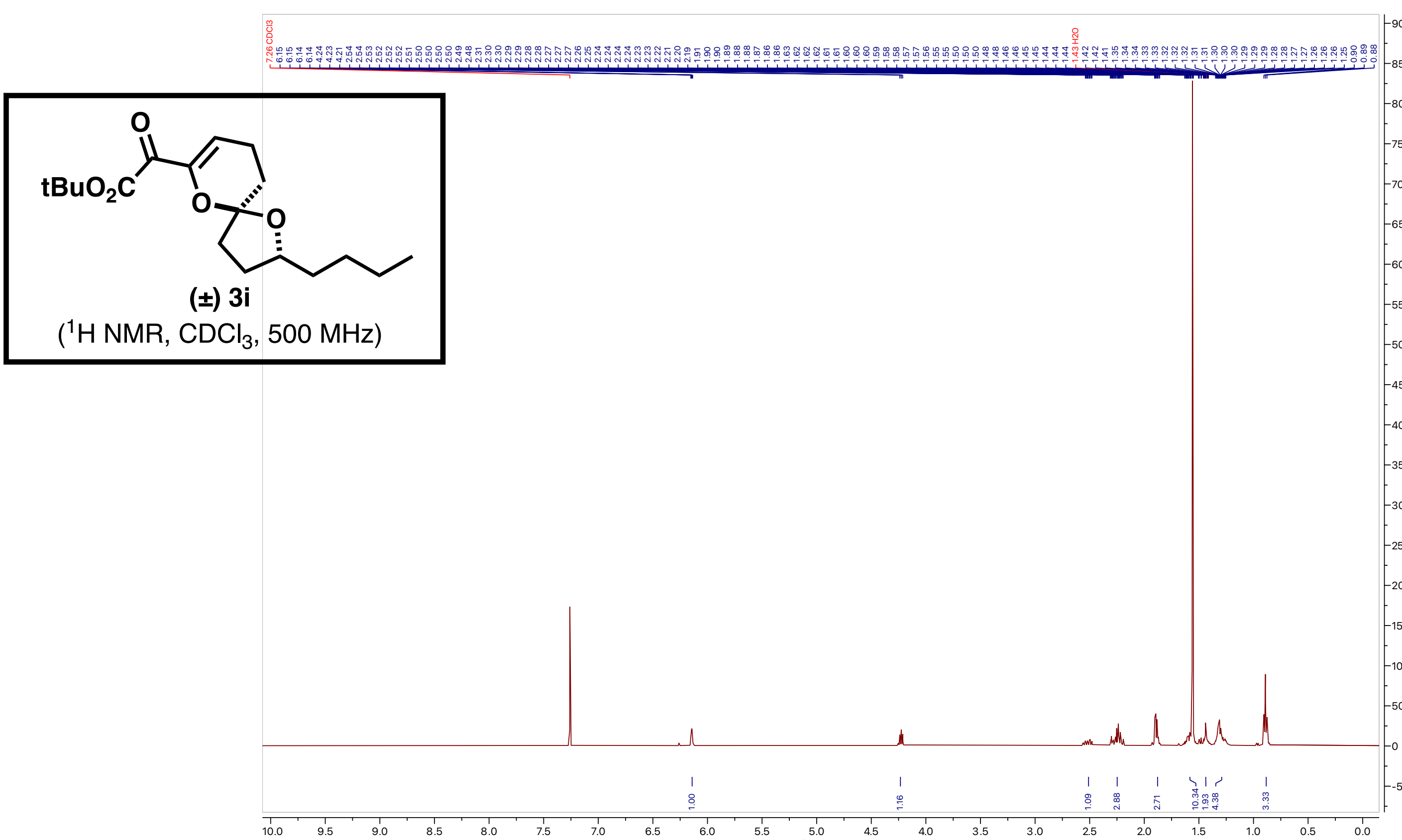
tBuO2C
(±) 3i
(1H NMR, CDCl3, 500 MHz)
7.26 CDCl3
1.43 H2O
1.00
1.16
1.09
2.88
2.71
10.34
1.93
4.38
3.33
f1 (ppm)

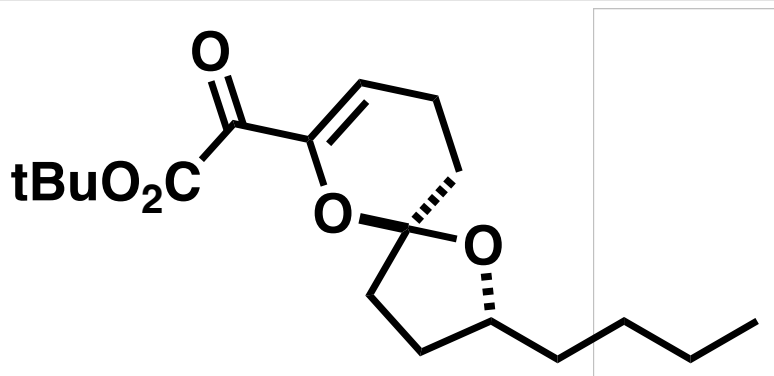

(±) 3i

(^{13}C NMR, $CDCl_3$, 126 MHz)

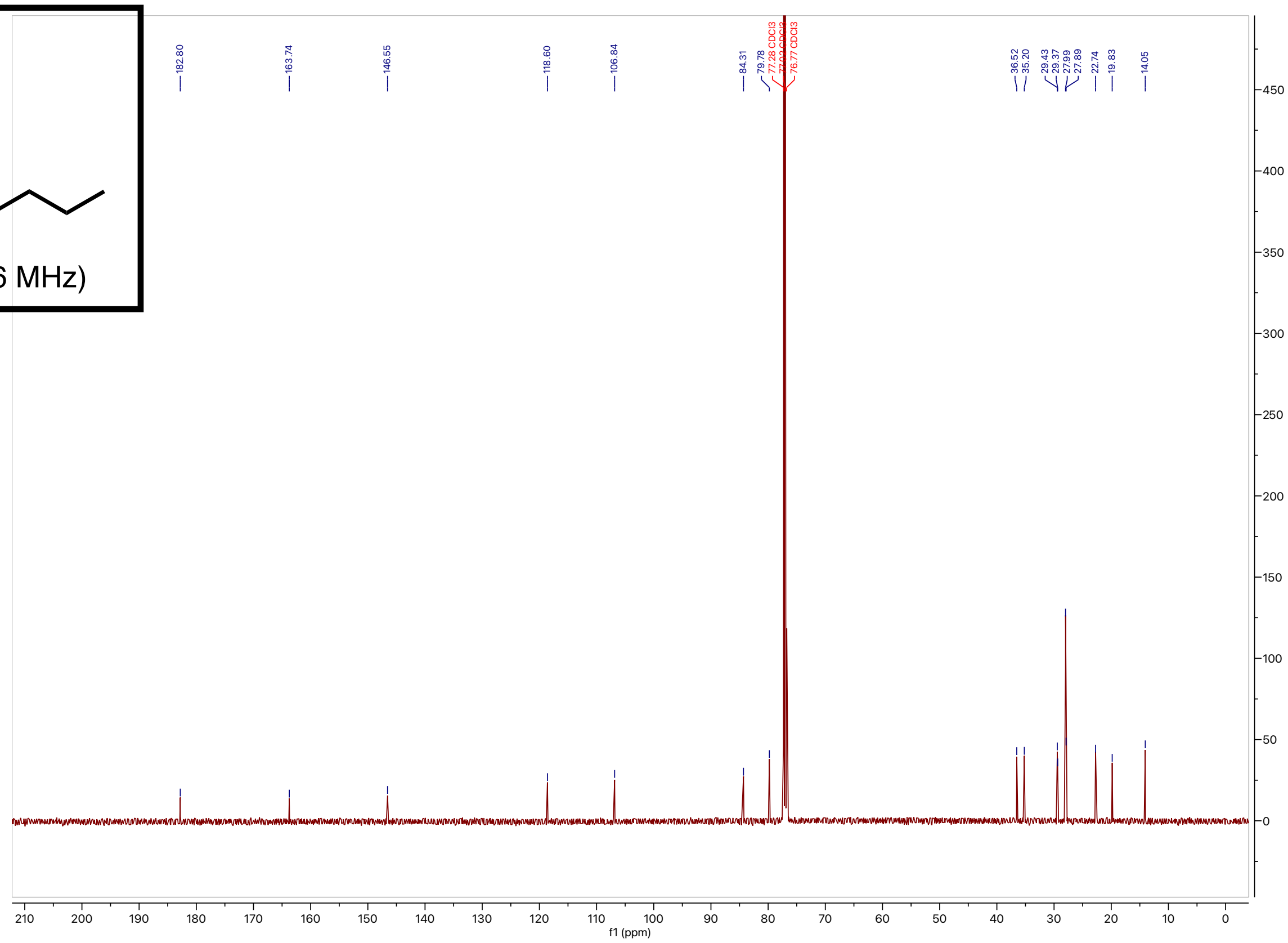

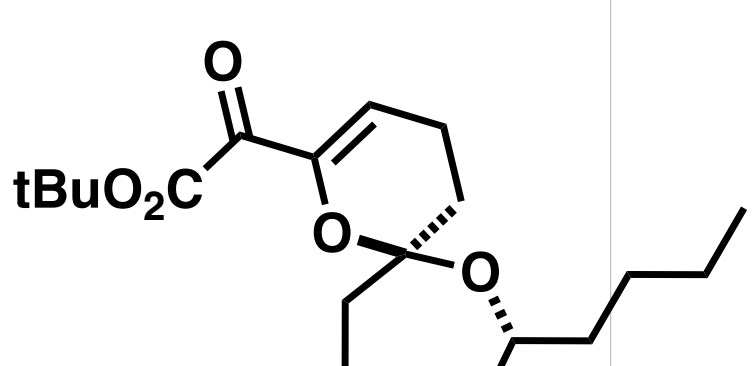

(±) 3j

(^{1}H NMR, $CDCl_3$, 500 MHz)

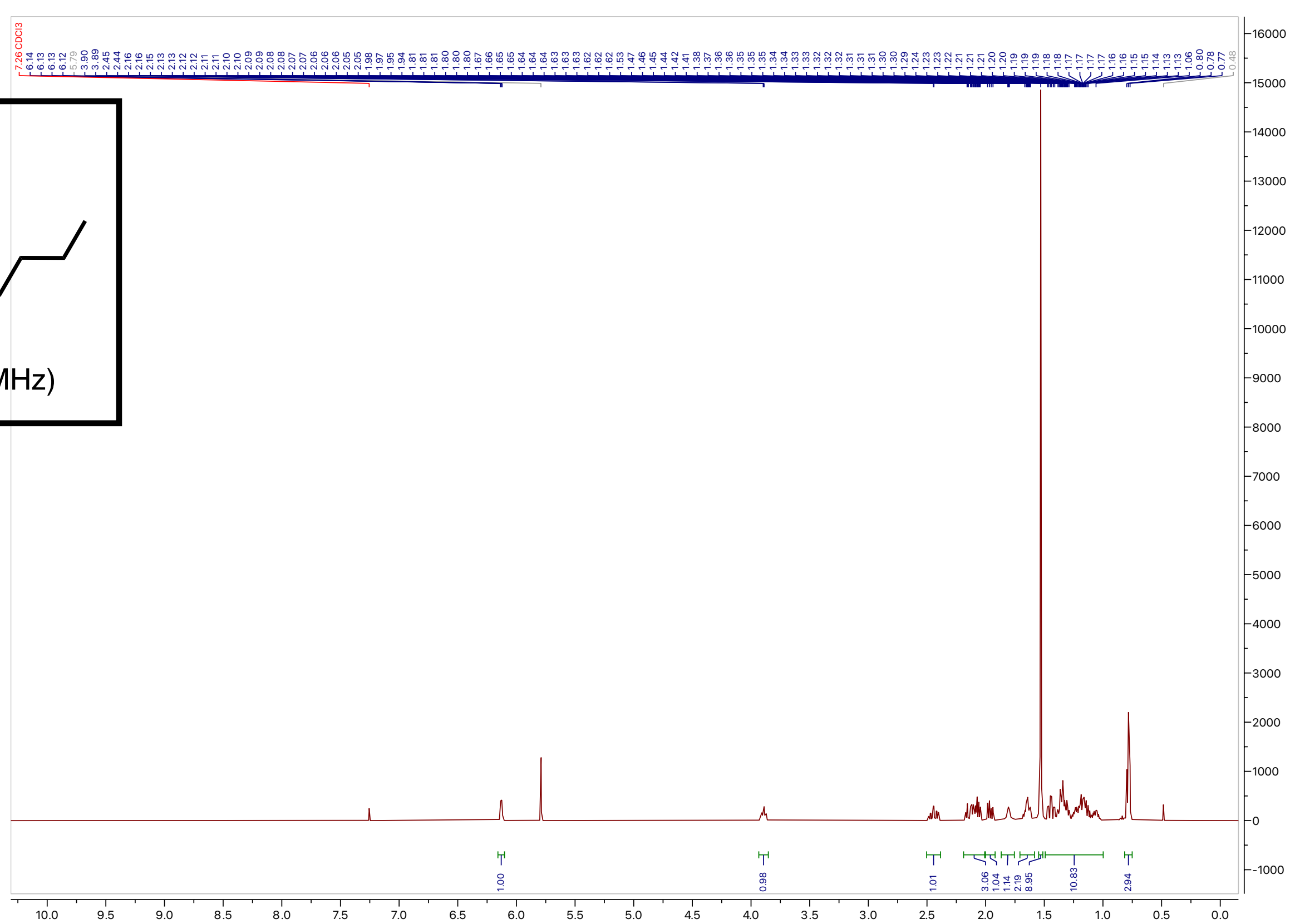

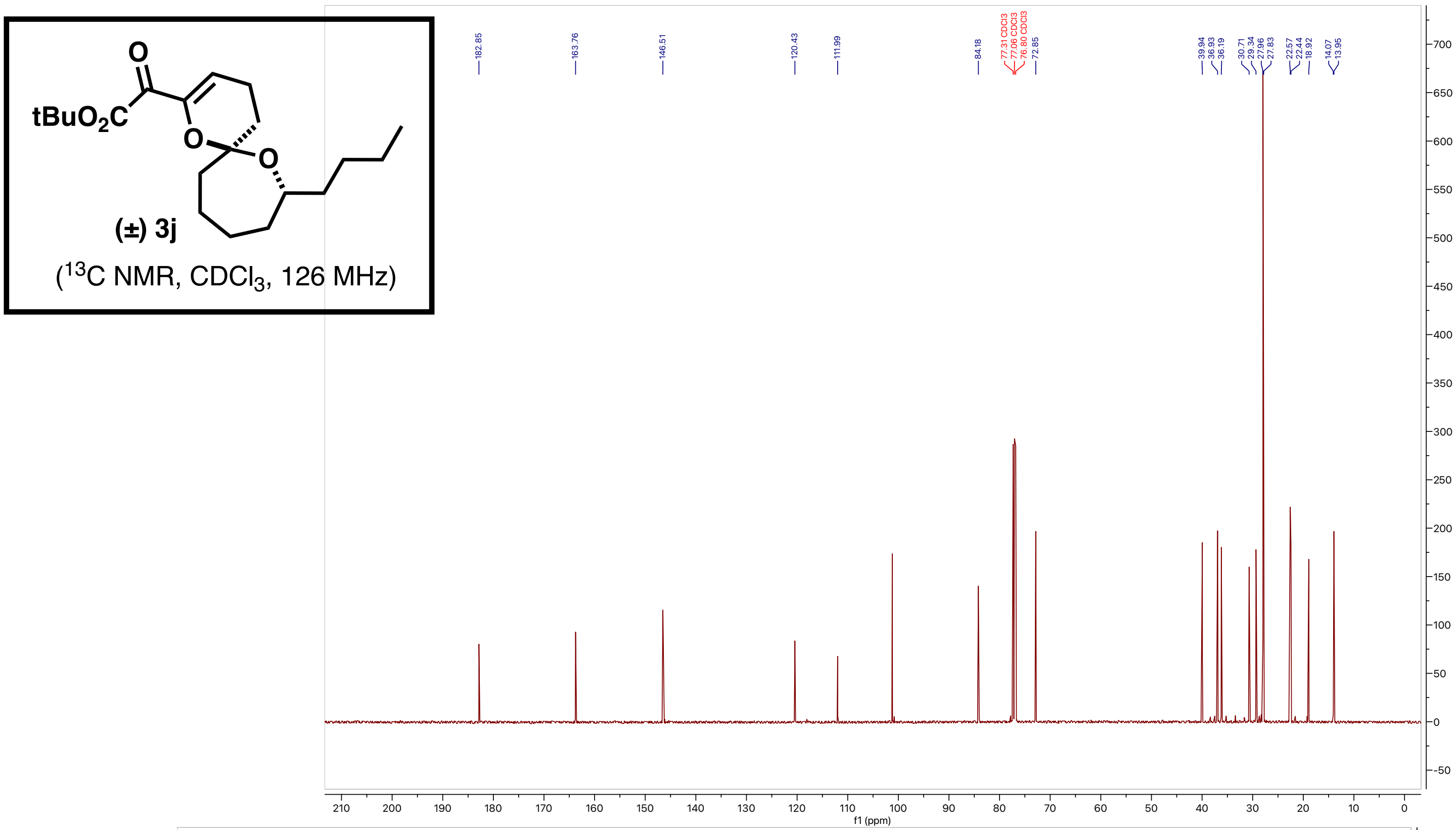
tBuO2C
O
O
O
(±) 3j
(13C NMR, CDCl3, 126 MHz)
f1 (ppm)

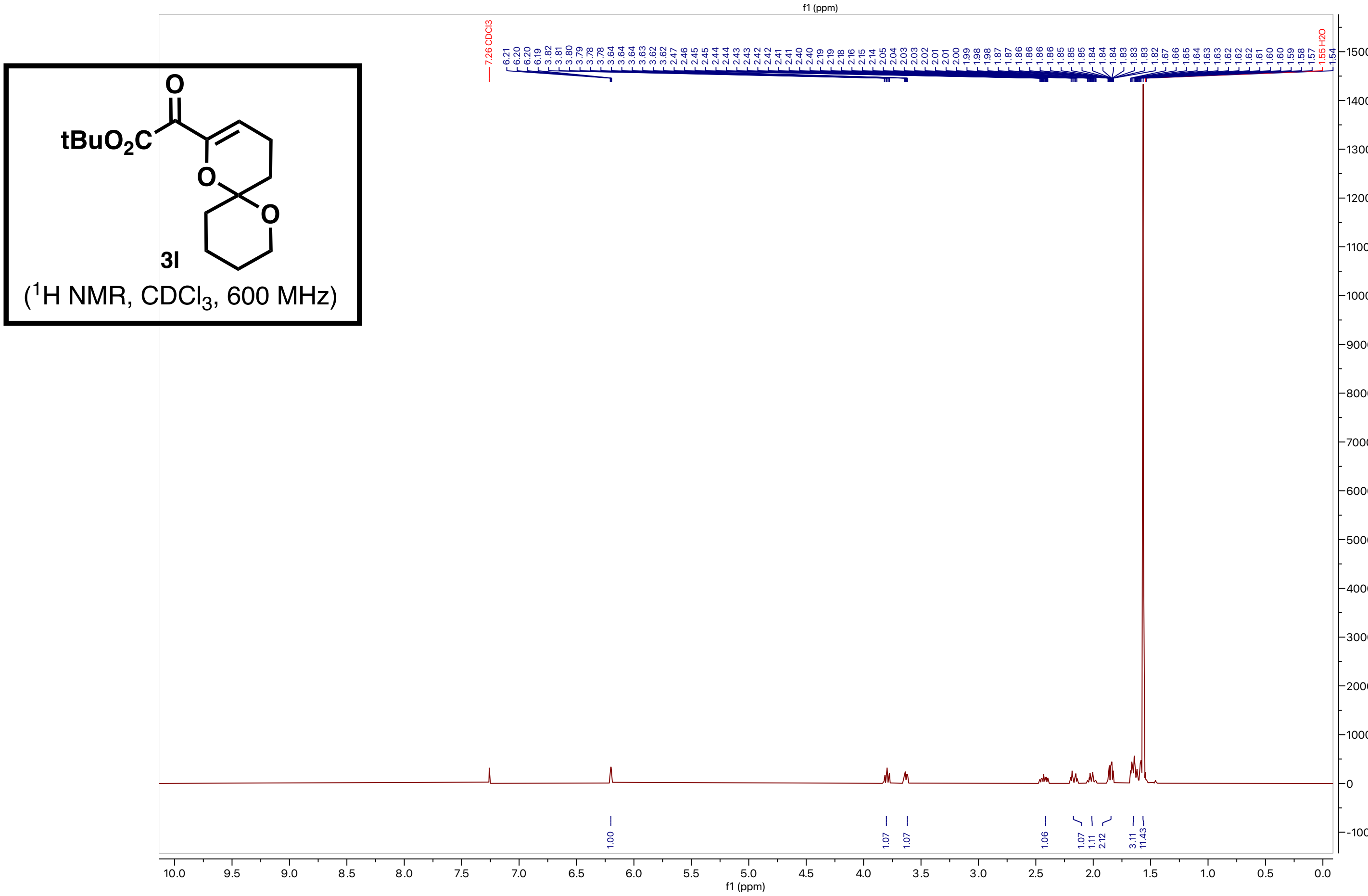
tBuO2C
O
O
O
3l
(1H NMR, CDCl3, 600 MHz)
f1 (ppm)

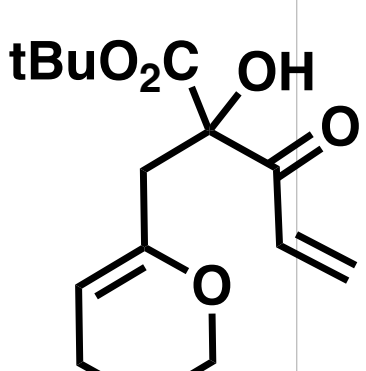

5l

(^{1}H NMR, $CDCl_3$, 500 MHz)

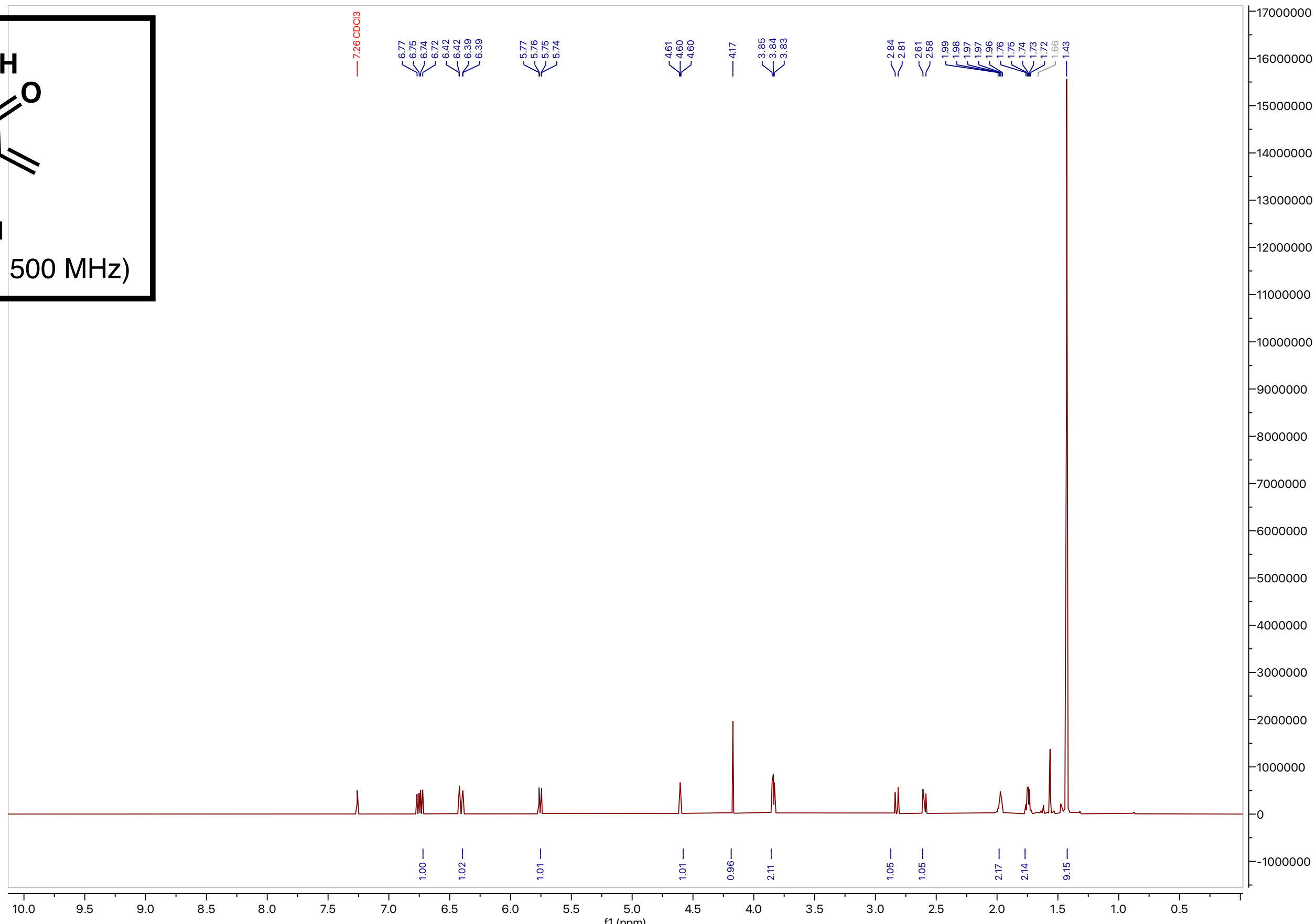

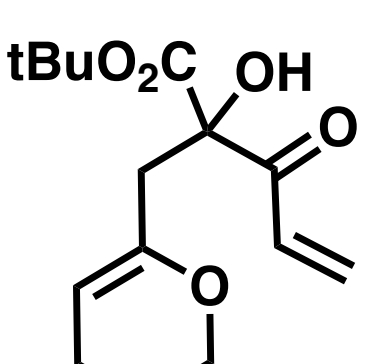

5l

(^{13}C NMR, $CDCl_3$,126 MHz)

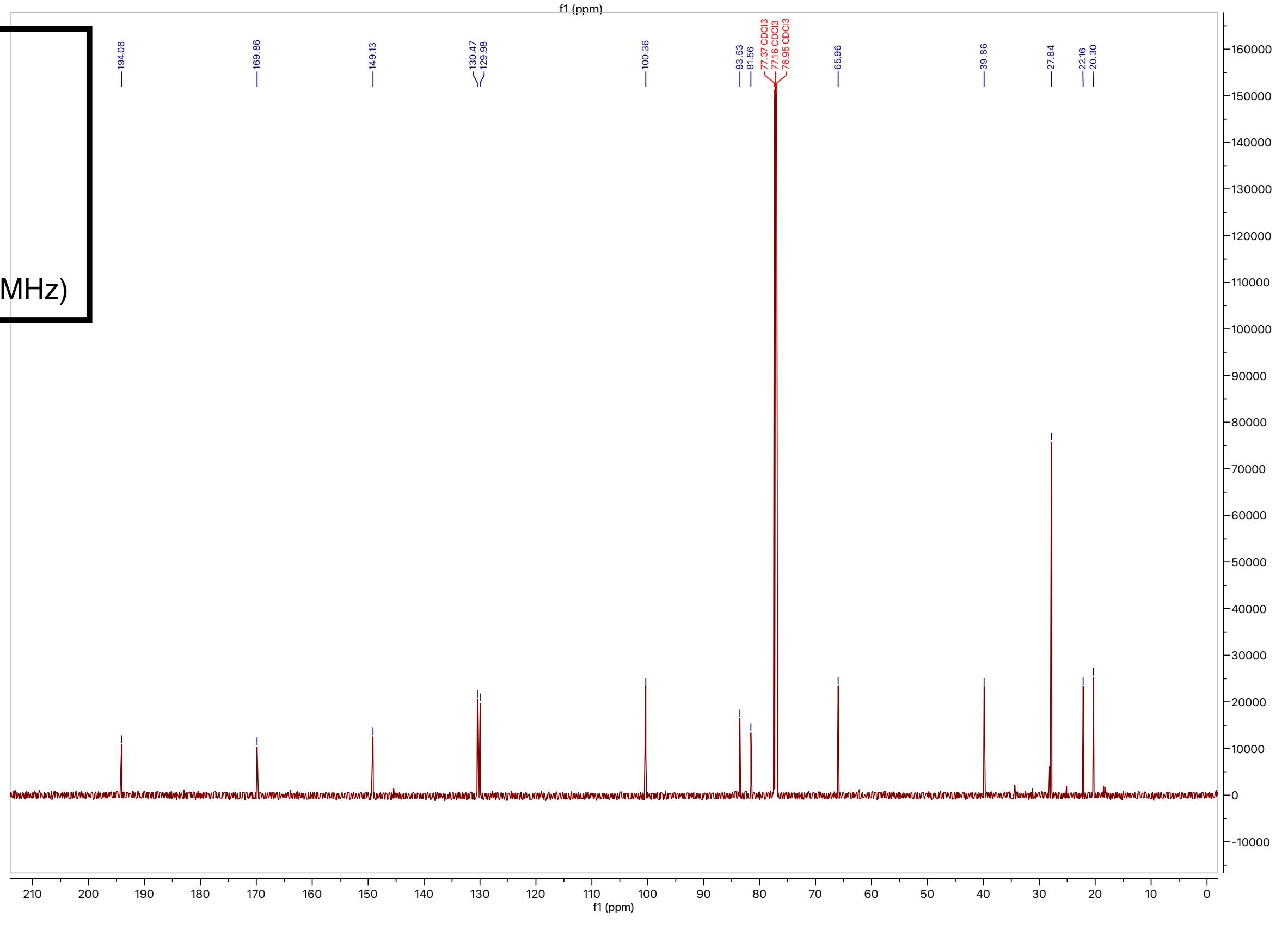

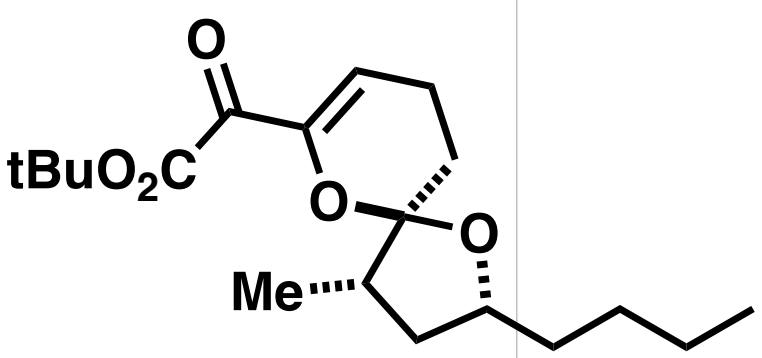

(±) 3n

(^{1}H NMR, $CDCl_3$, 500 MHz, 85.5% purity)

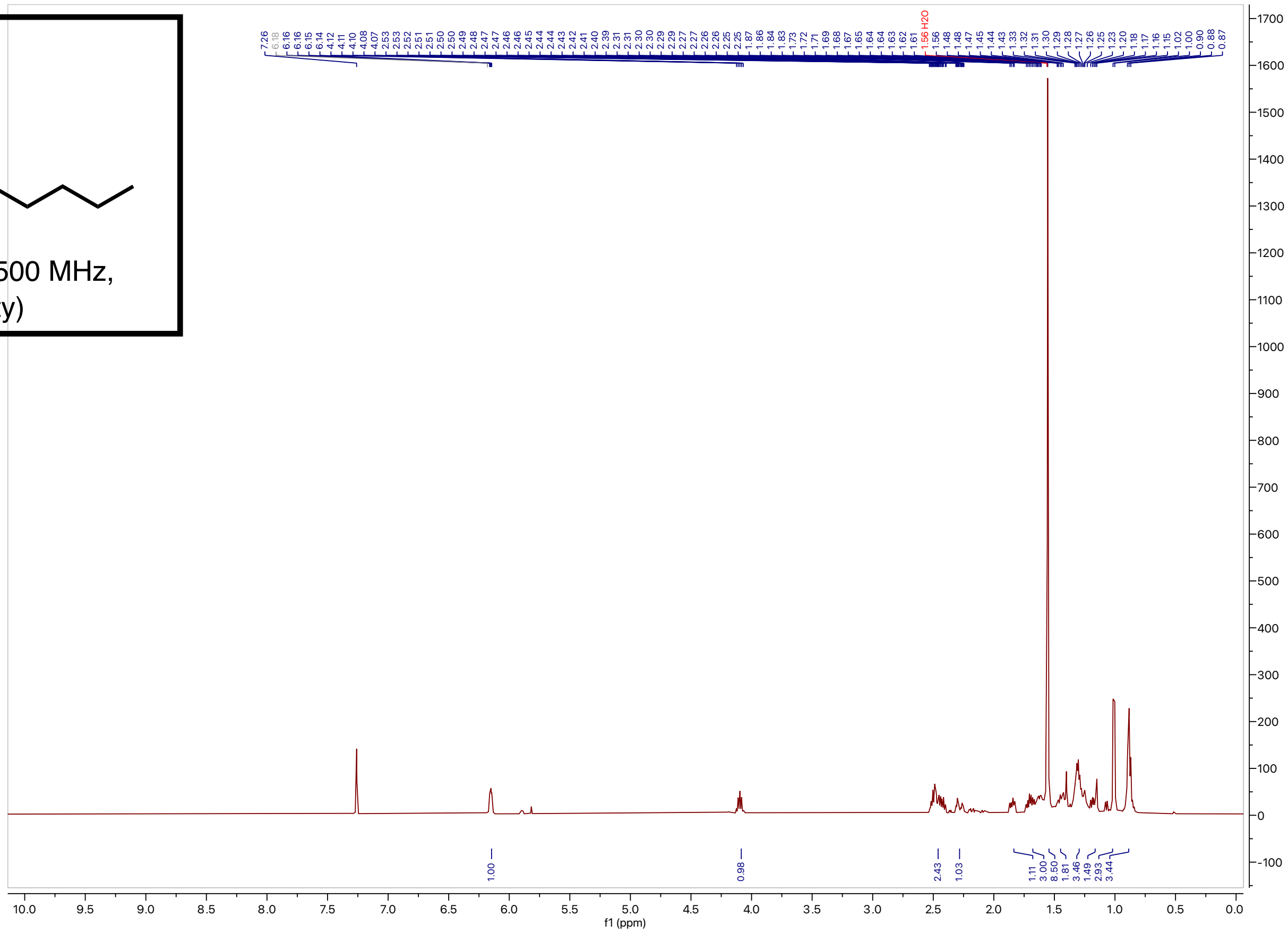

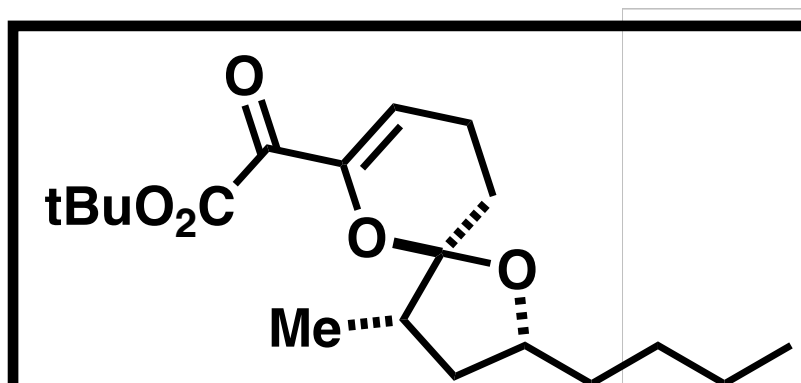

(±) 3n

(^{13}C NMR, $CDCl_3$, 126 MHz, 85.5% purity)

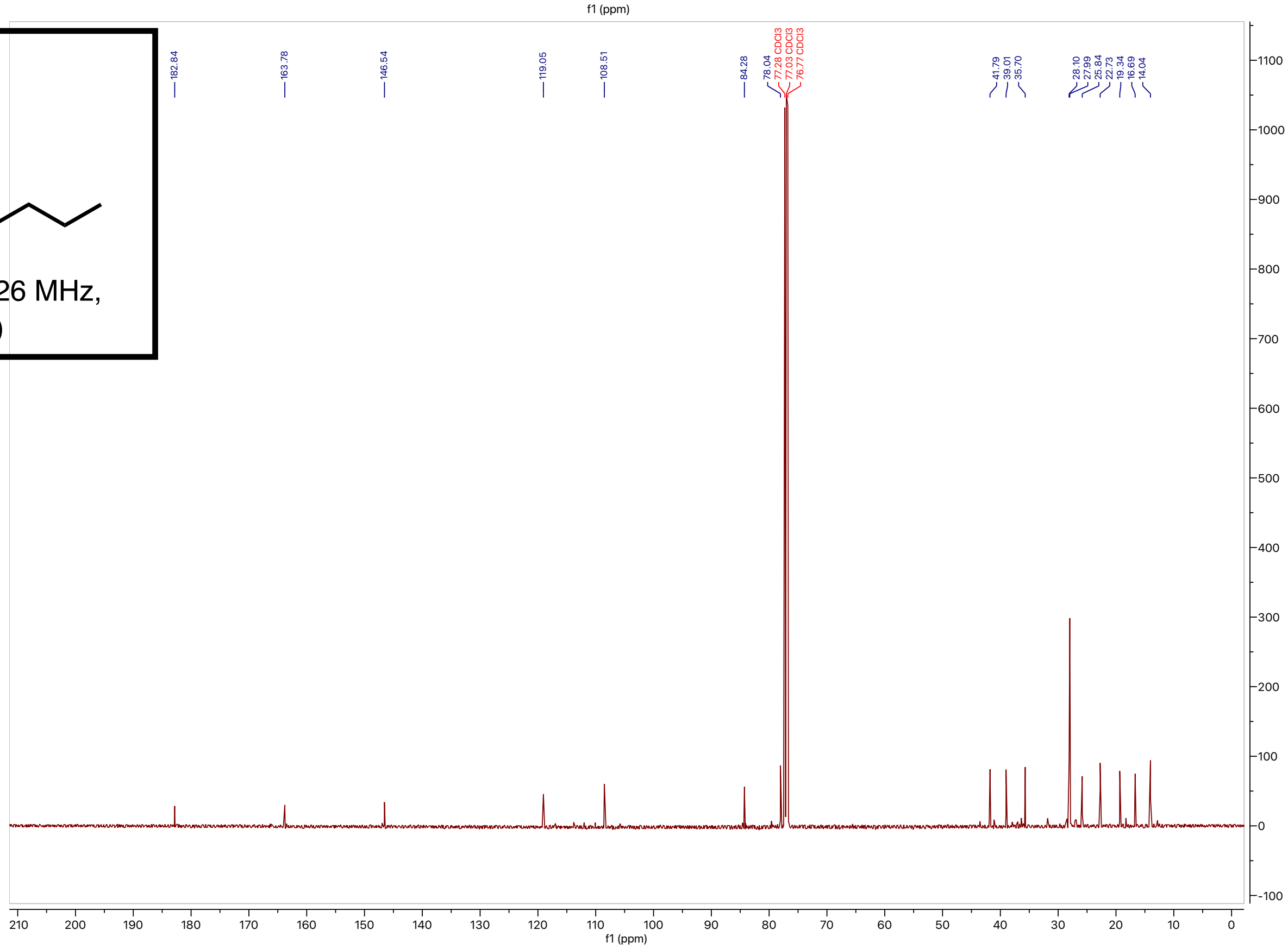

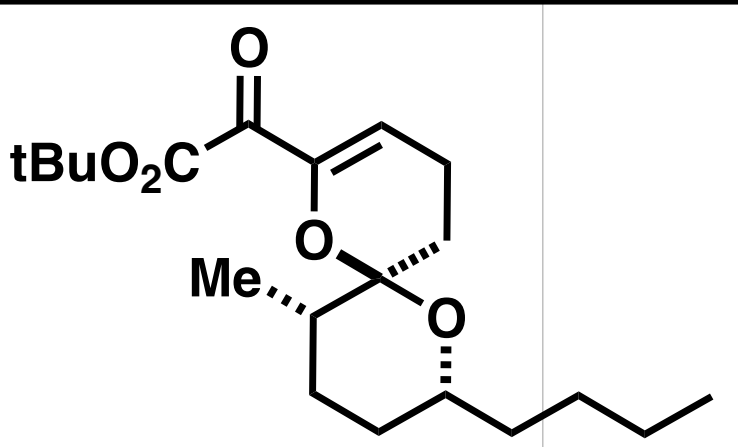

(±) 3o

(^1H NMR, $CDCl_3$, 500 MHz, 89% purity)

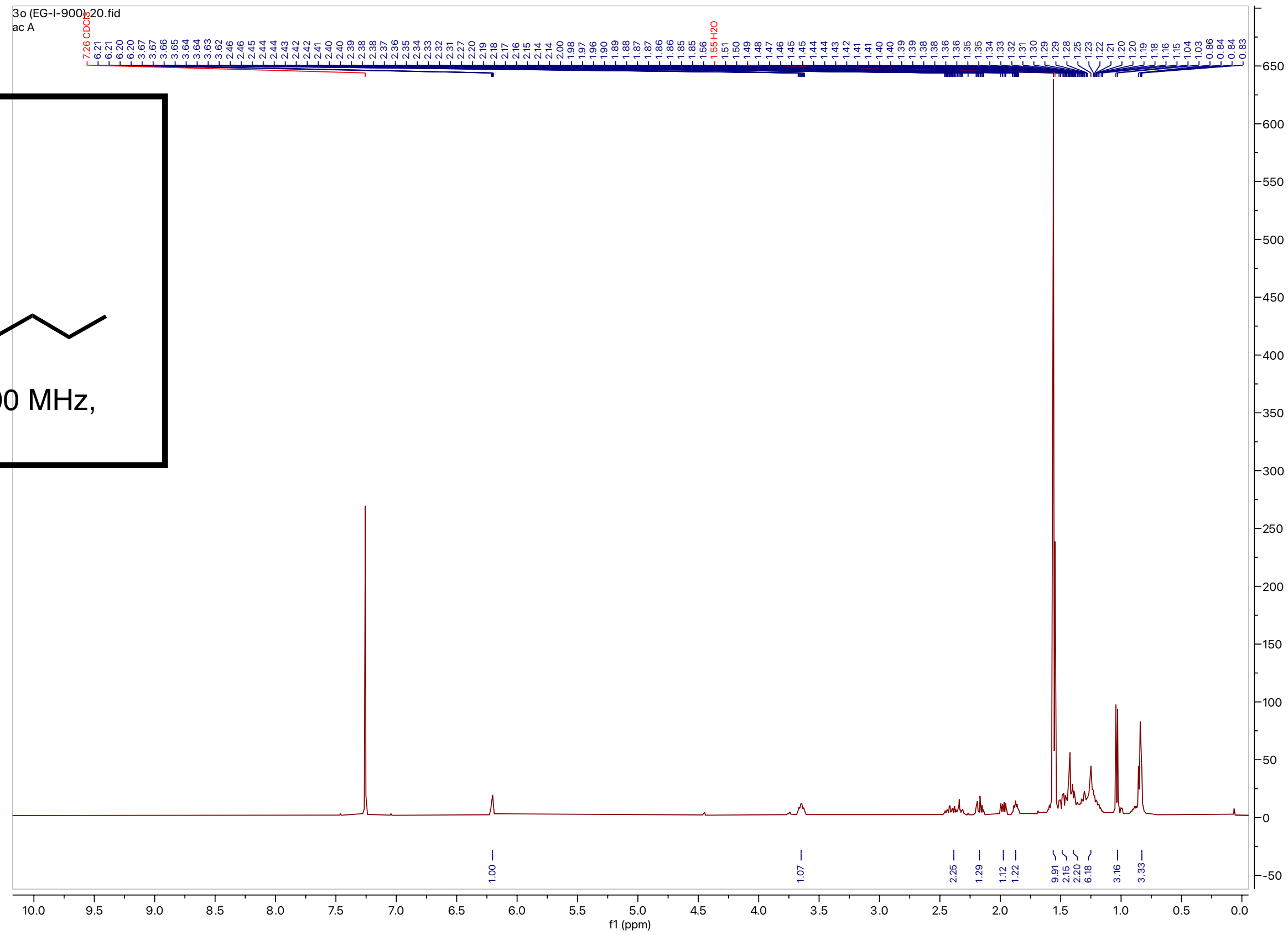

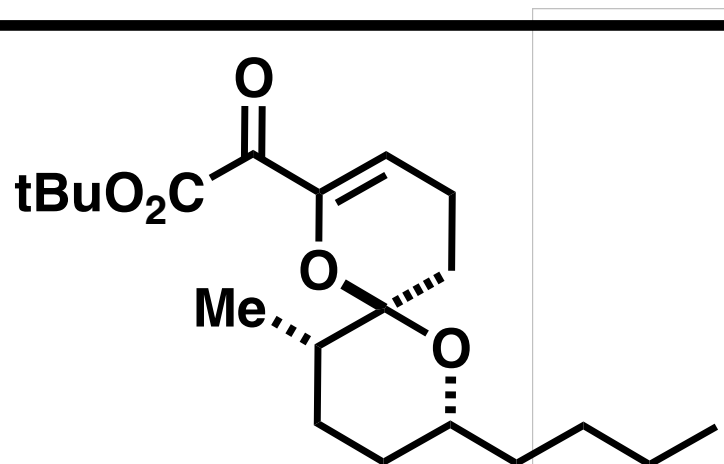

(±) 3o

(^{13}C NMR, $CDCl_3$, 126 MHz, 89% purity)

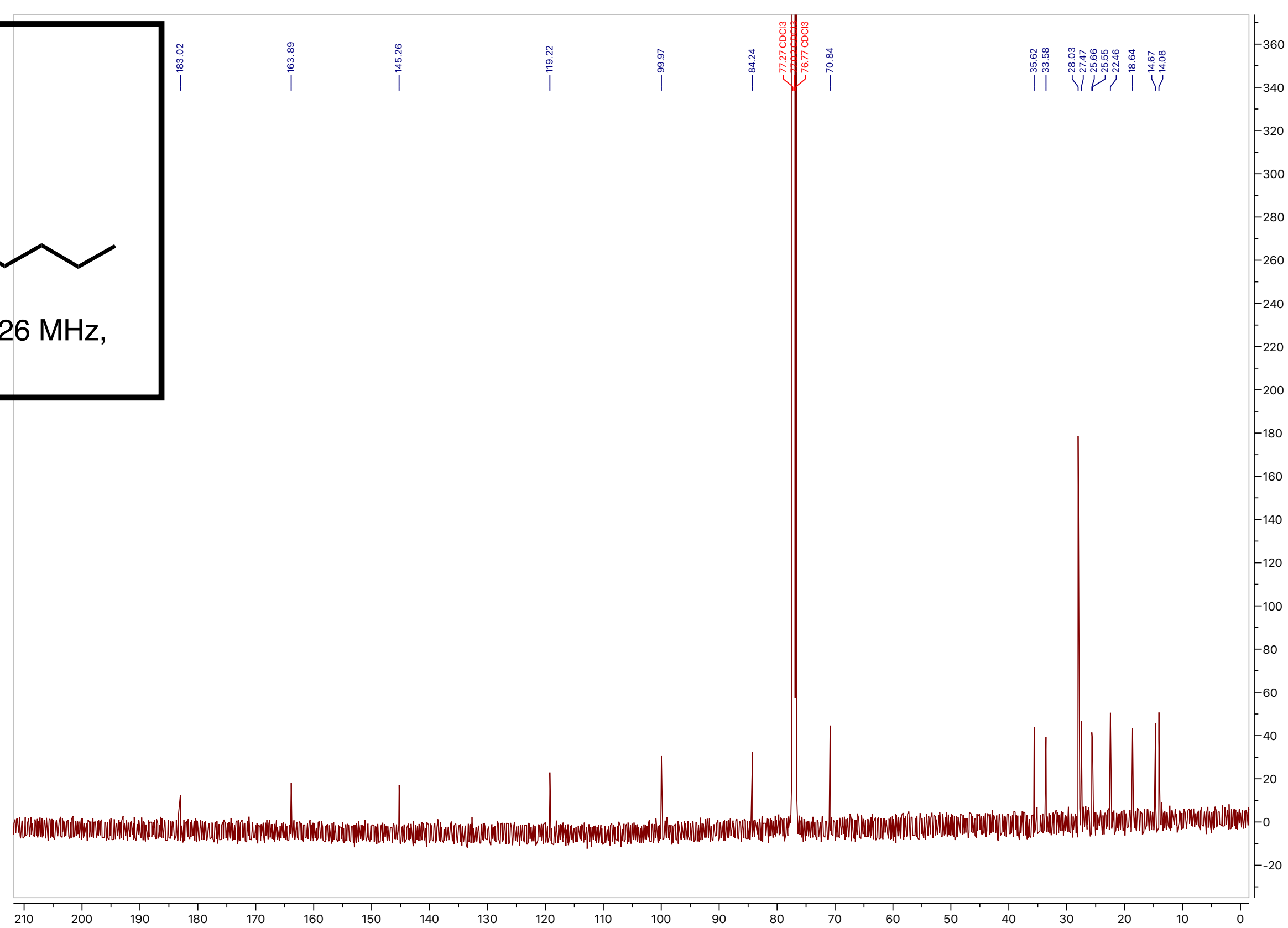

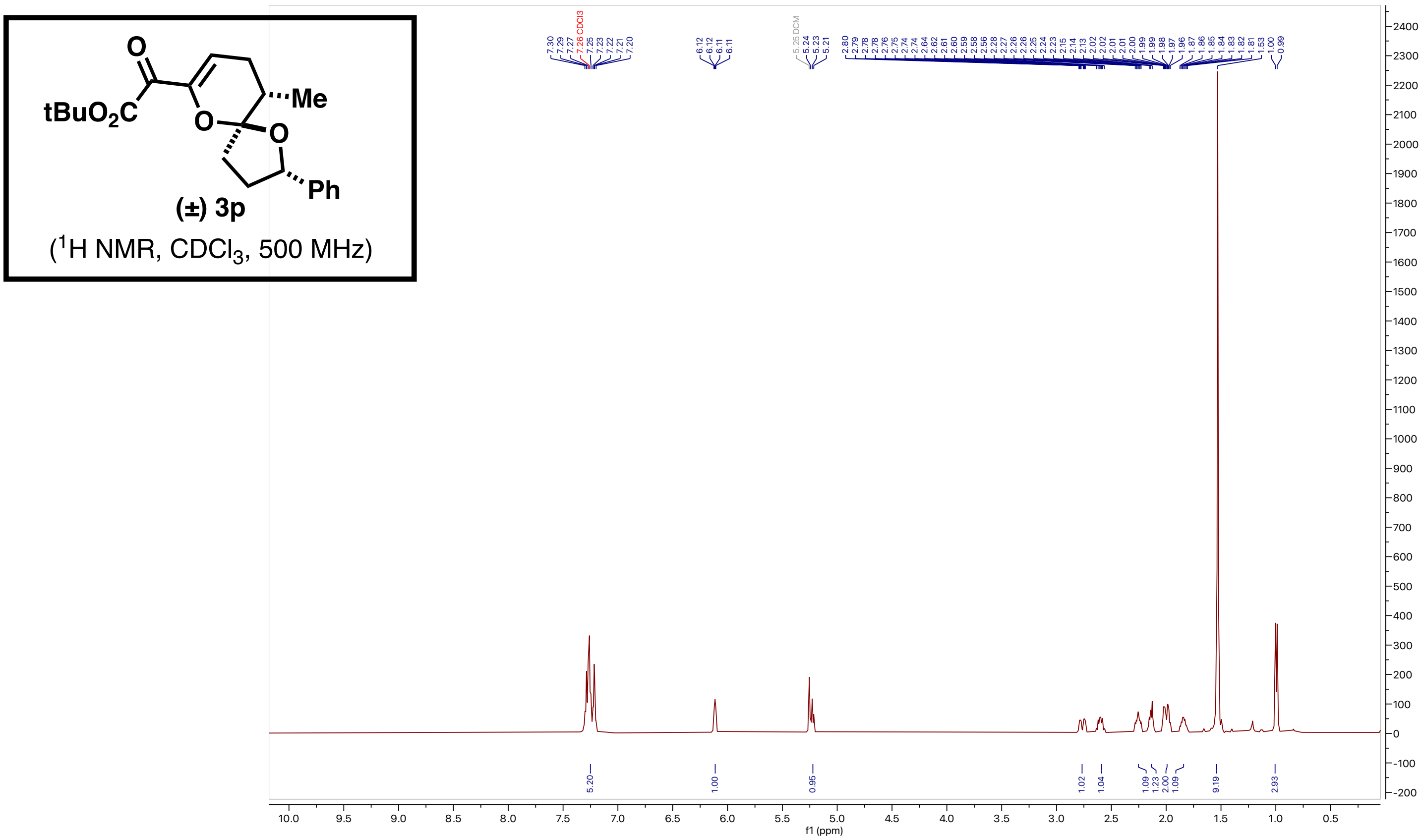
tBuO$_2$C
O
Me
O
O
Ph
(±) 3p
(^{1}H NMR, CDCl$_3$, 500 MHz)
7.30 7.29 7.27 7.26 CDCl3 7.25 7.23 7.22 7.21 7.20
6.12 6.12 6.11 6.11
5.25 DCM 5.24 5.23 5.21
2.80 2.79 2.78 2.76 2.75 2.74 2.74 2.64 2.62 2.61 2.60 2.59 2.58 2.56 2.28 2.27 2.26 2.26 2.25 2.24 2.23 2.15 2.14 2.13 2.02 2.02 2.01 2.01 2.00 1.99 1.99 1.98 1.97 1.96 1.87 1.86 1.85 1.84 1.83 1.82 1.81 1.53 1.00 0.99
5.20
1.00
0.95
1.02
1.04
1.09
1.23
2.00
1.09
9.19
2.93
f1 (ppm)

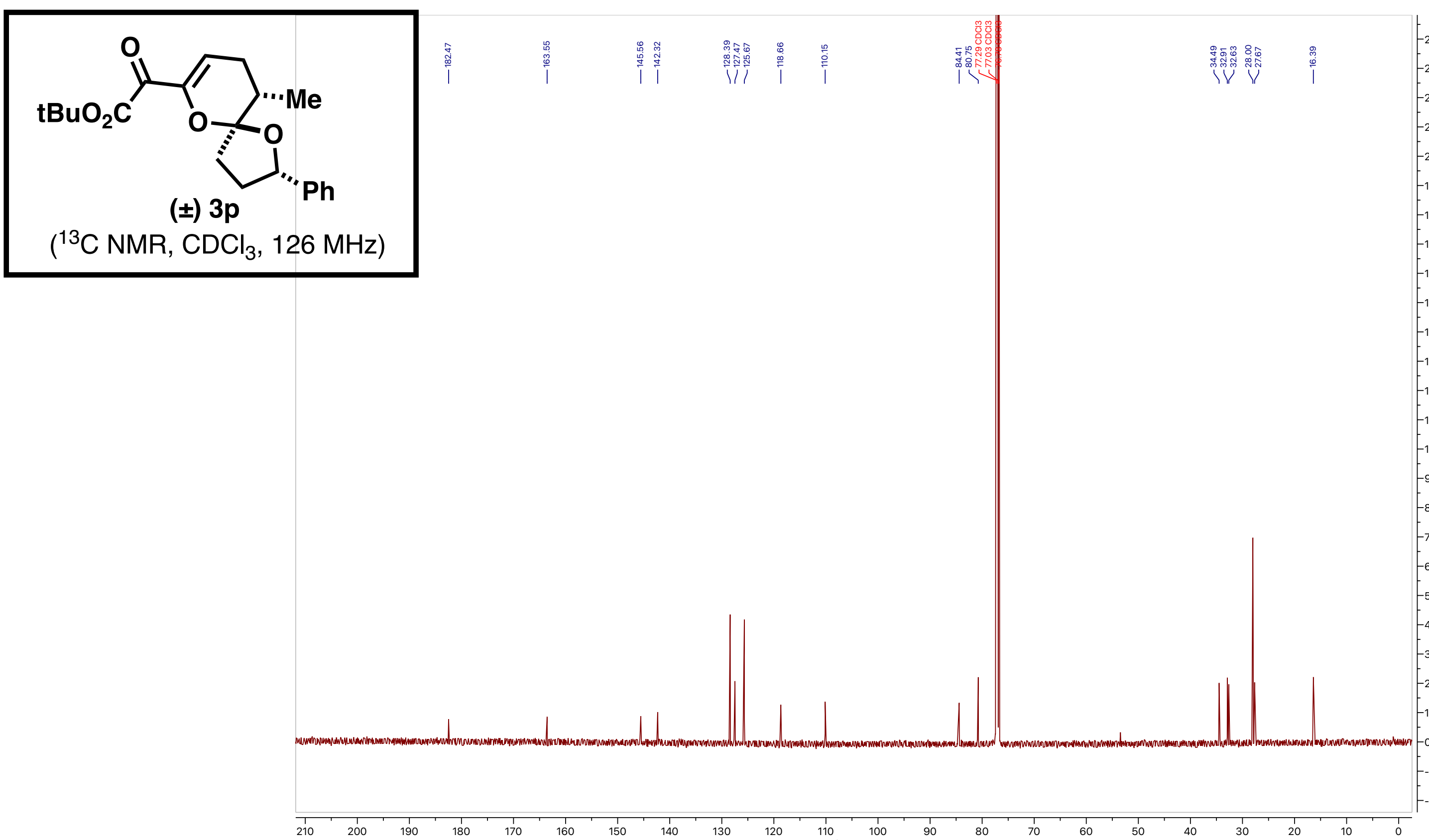
tBuO$_2$C
O
Me
O
O
Ph
(±) 3p
(^{13}C NMR, CDCl$_3$, 126 MHz)
182.47
163.55
145.56
142.32
128.39 127.47 125.67
118.66
110.15
84.41 80.75 77.29 CDCl3 77.03 CDCl3
34.49 32.91 32.63
28.00 27.67
16.39
f1 (ppm)

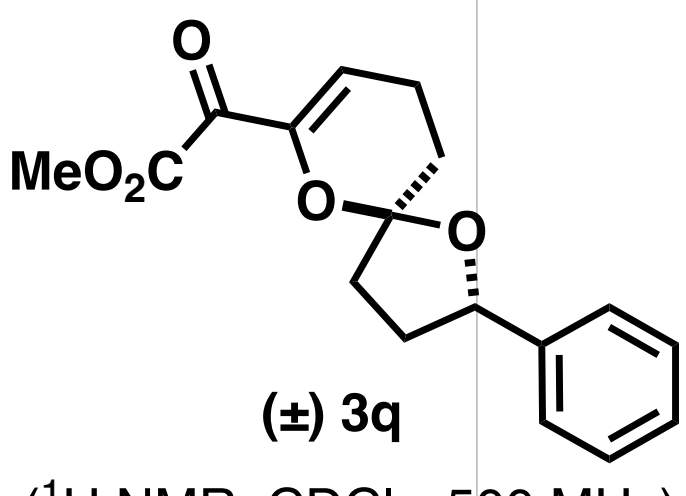

(±) 3q

(^1H NMR, $CDCl_3$, 500 MHz)

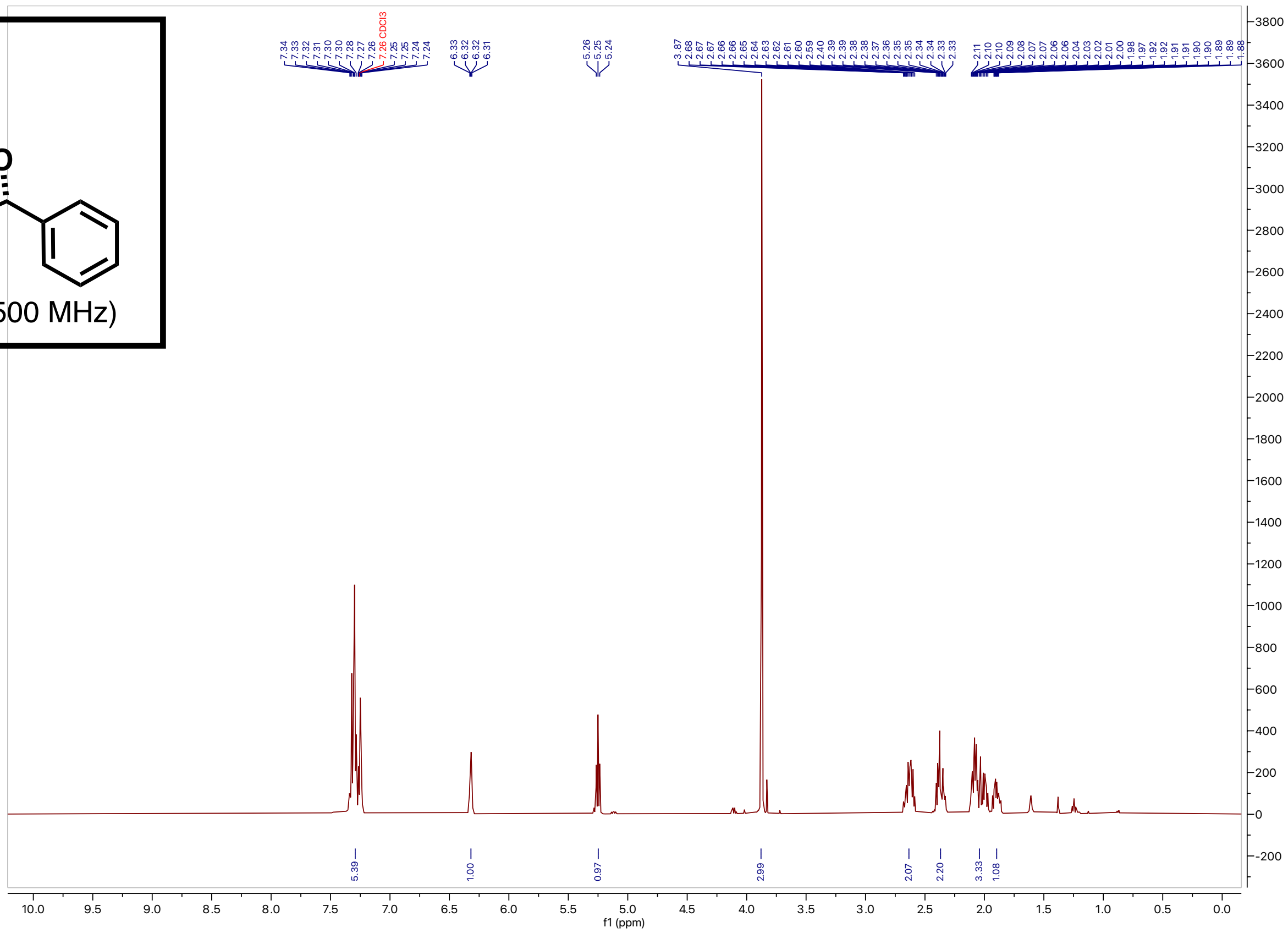

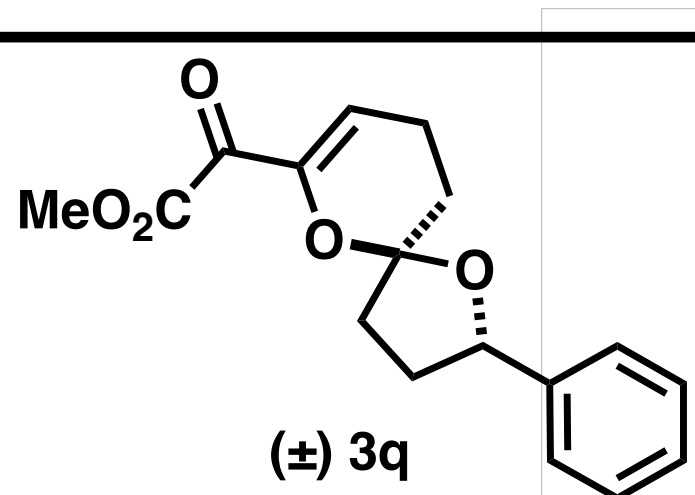

(±) 3q

(^{13}C NMR, $CDCl_3$, 126 MHz)

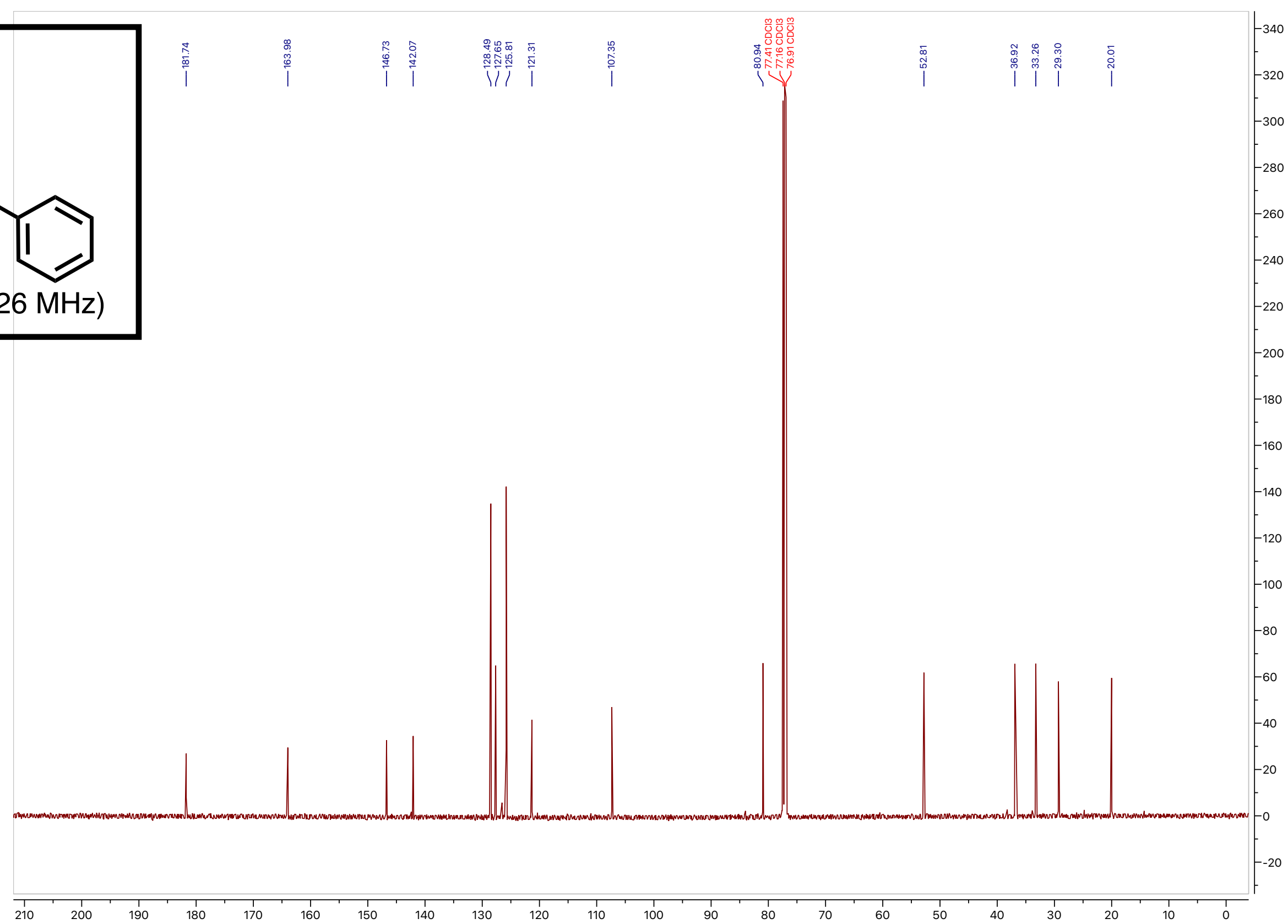

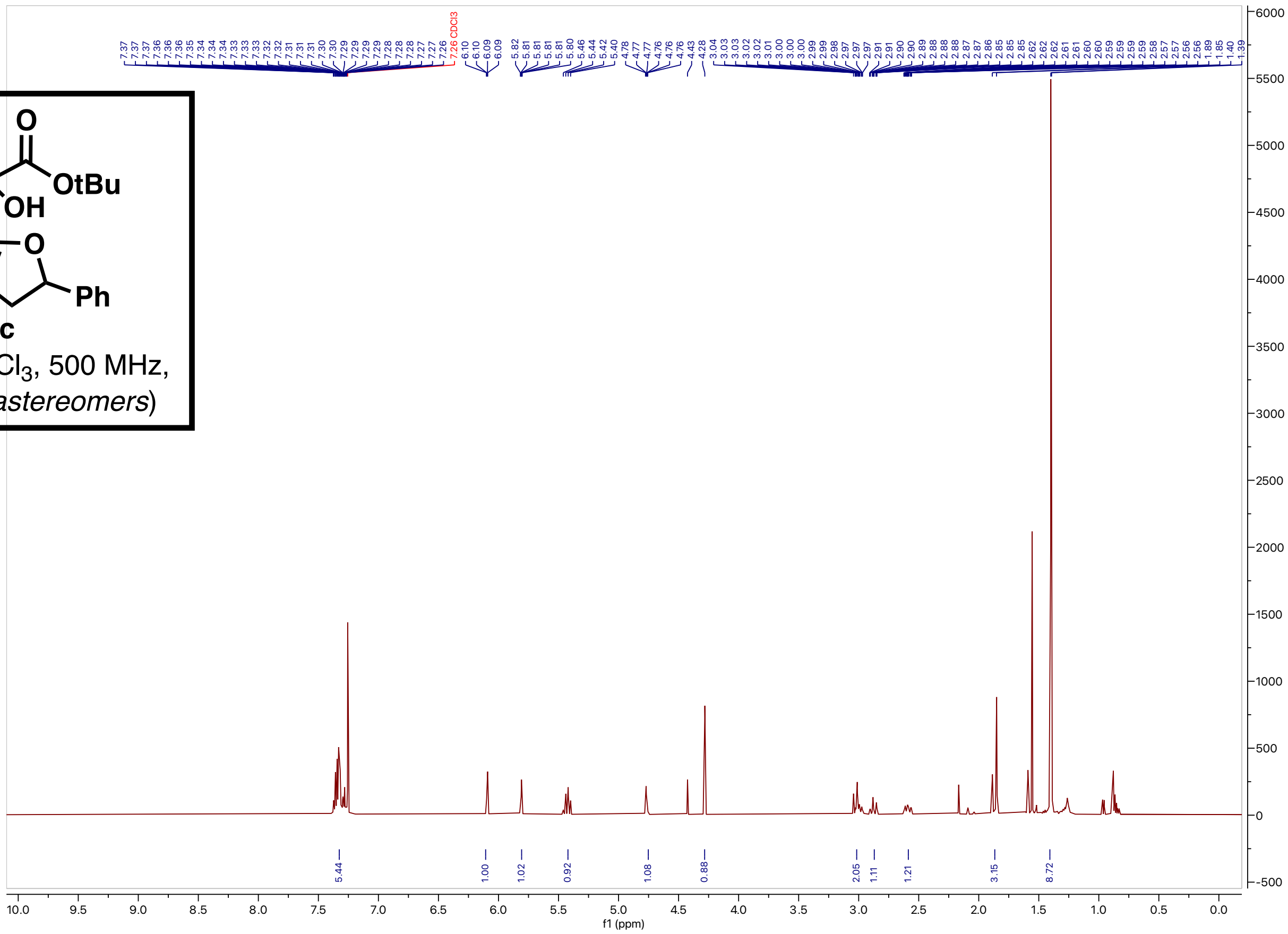

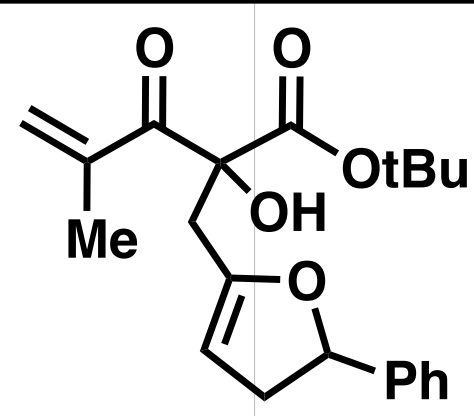

5c

(^{1}H NMR, $CDCl_3$, 500 MHz, *mixture of diastereomers*)

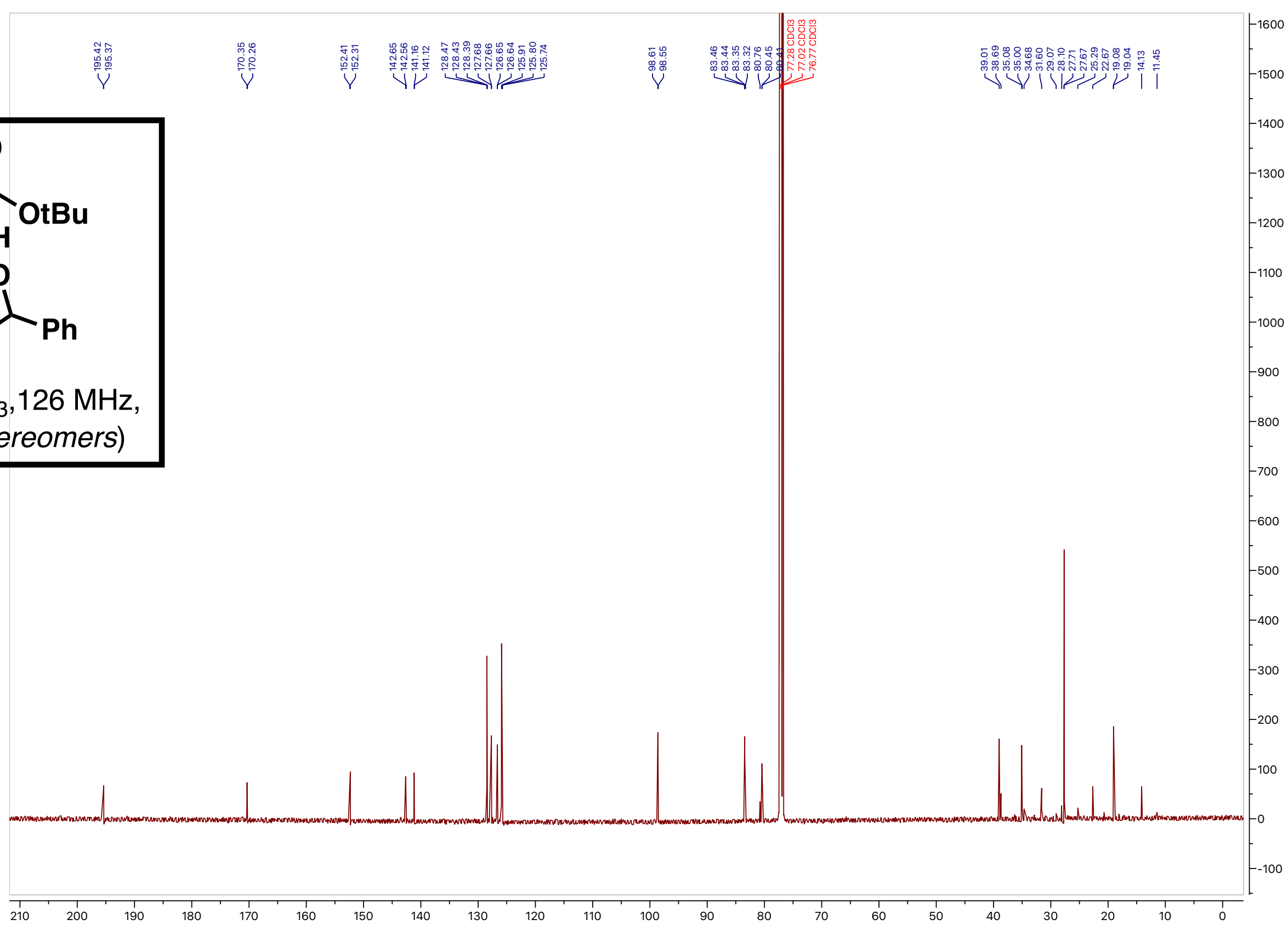

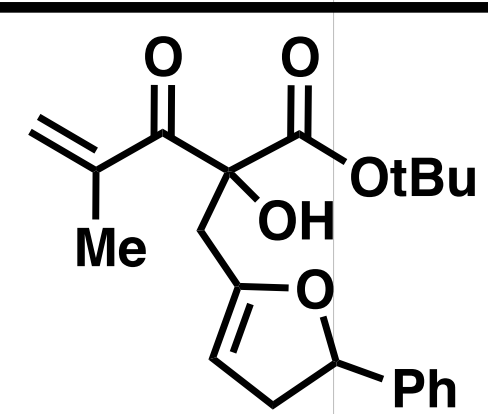

5c

(^{13}C NMR, $CDCl_3$,126 MHz, *mixture of diastereomers*)

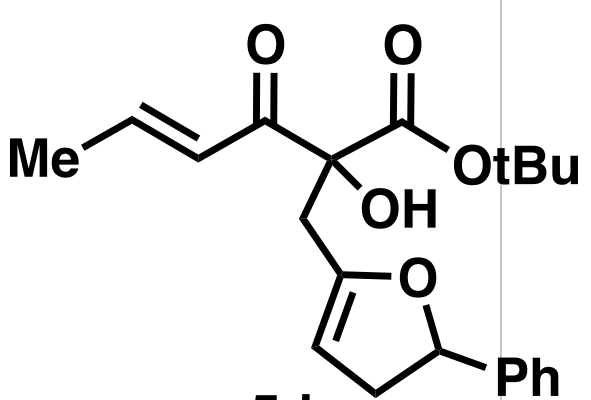

5d

(^{1}H NMR, $CDCl_3$, 500 MHz, *mixture of diastereomers*)

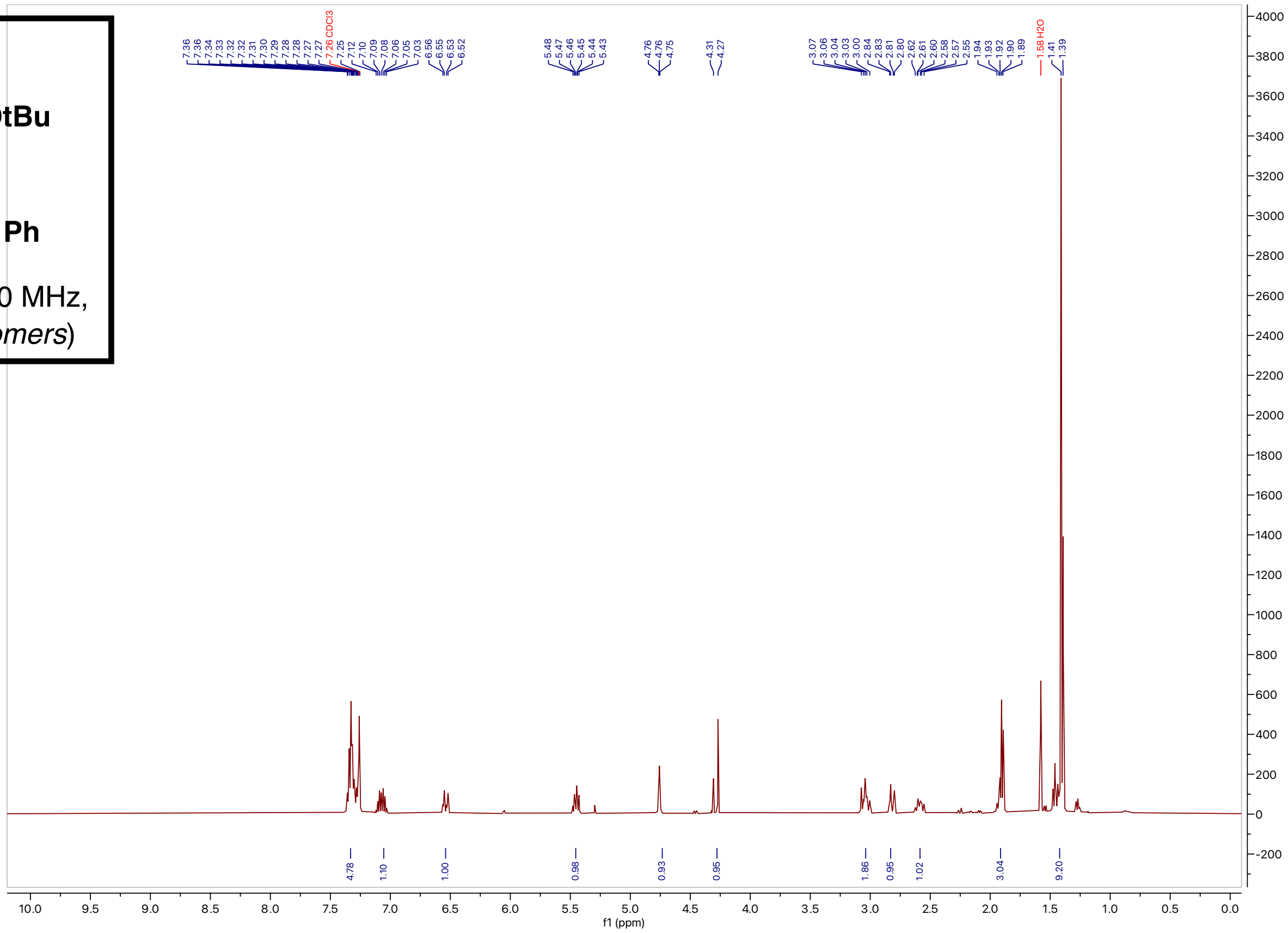

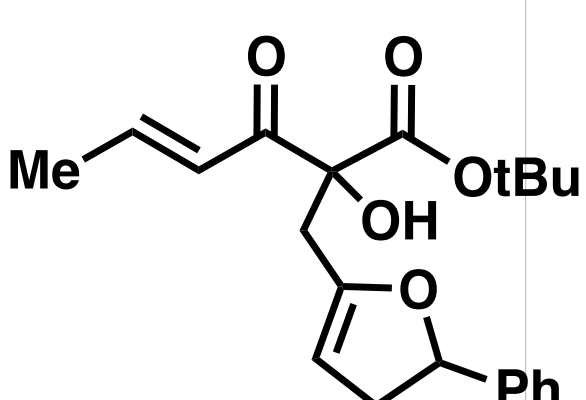

5d

(^{13}C NMR, $CDCl_3$,126 MHz, *mixture of diastereomers*)

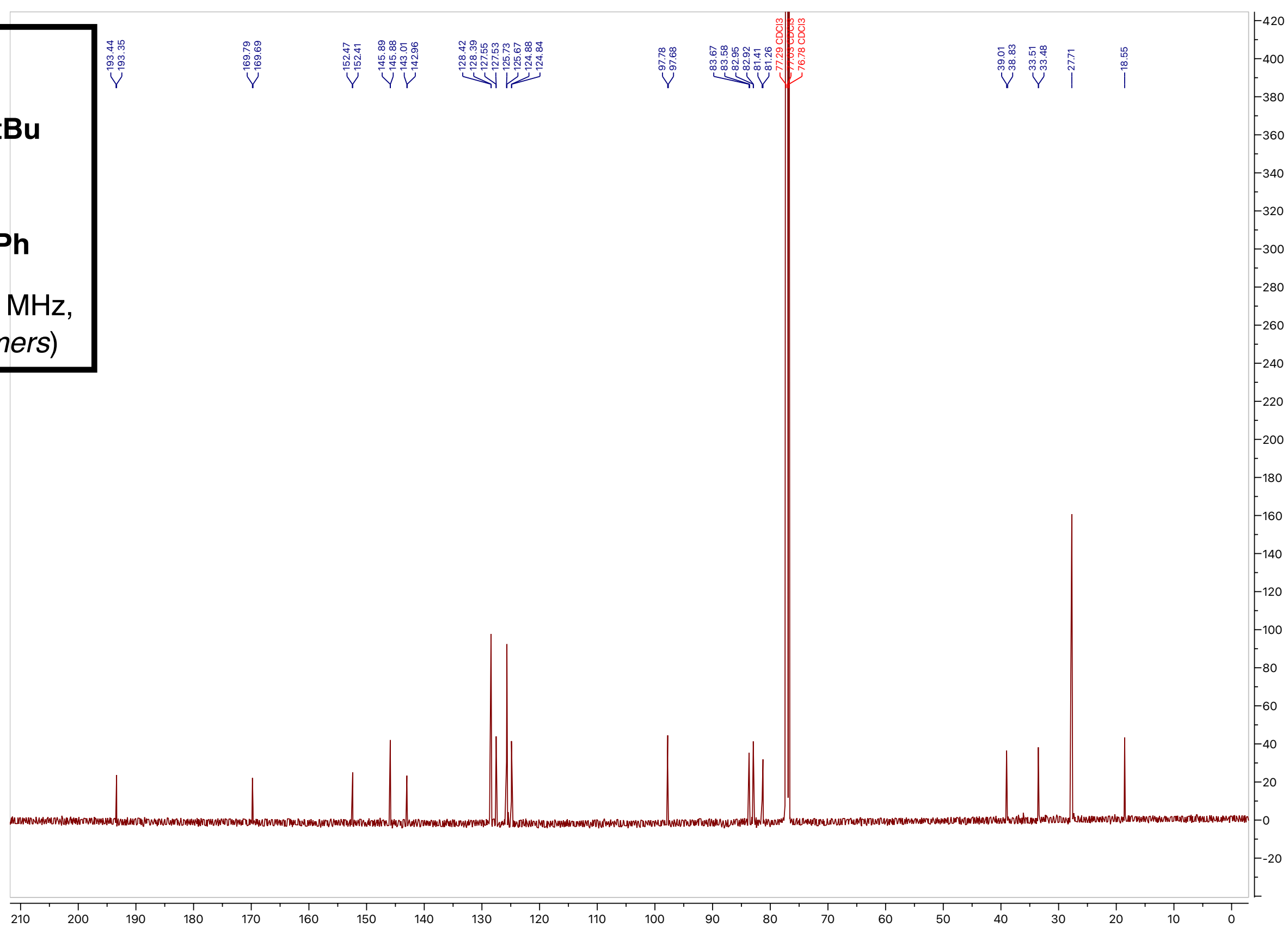

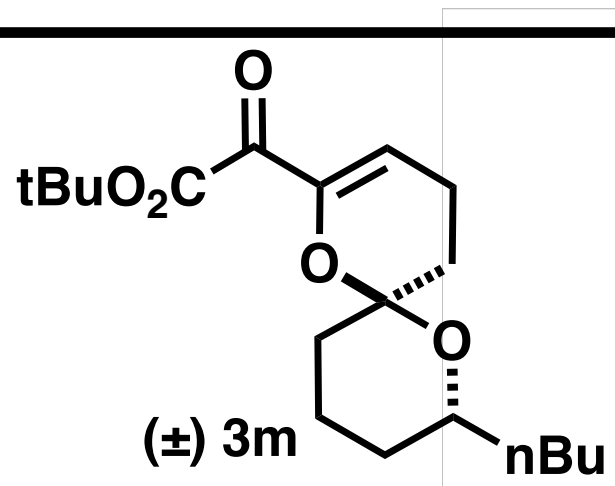

(±) 3m

(^{1}H NMR, $CDCl_3$, 500 MHz)

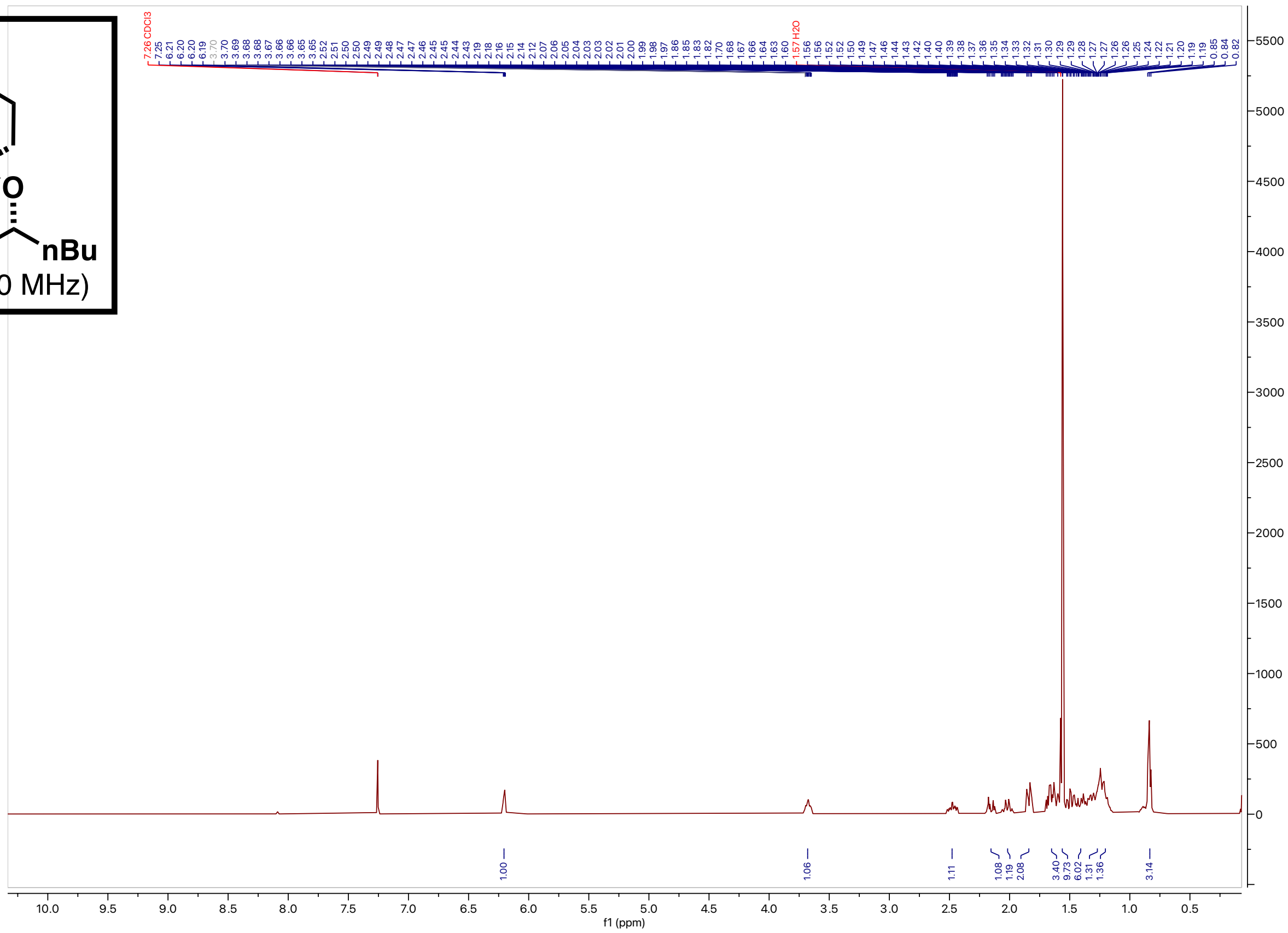

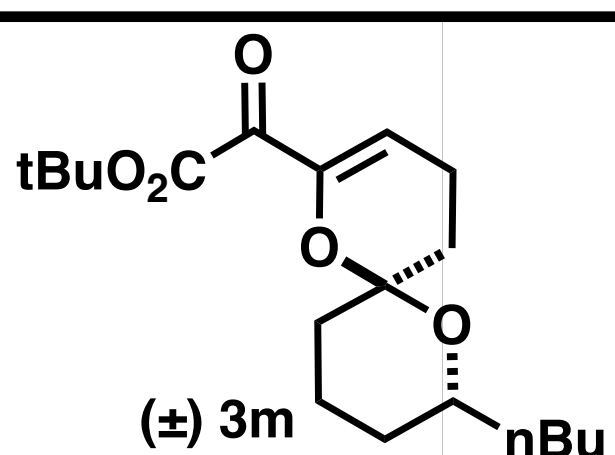

(±) 3m

(^{13}C NMR, $CDCl_3$, 126 MHz)

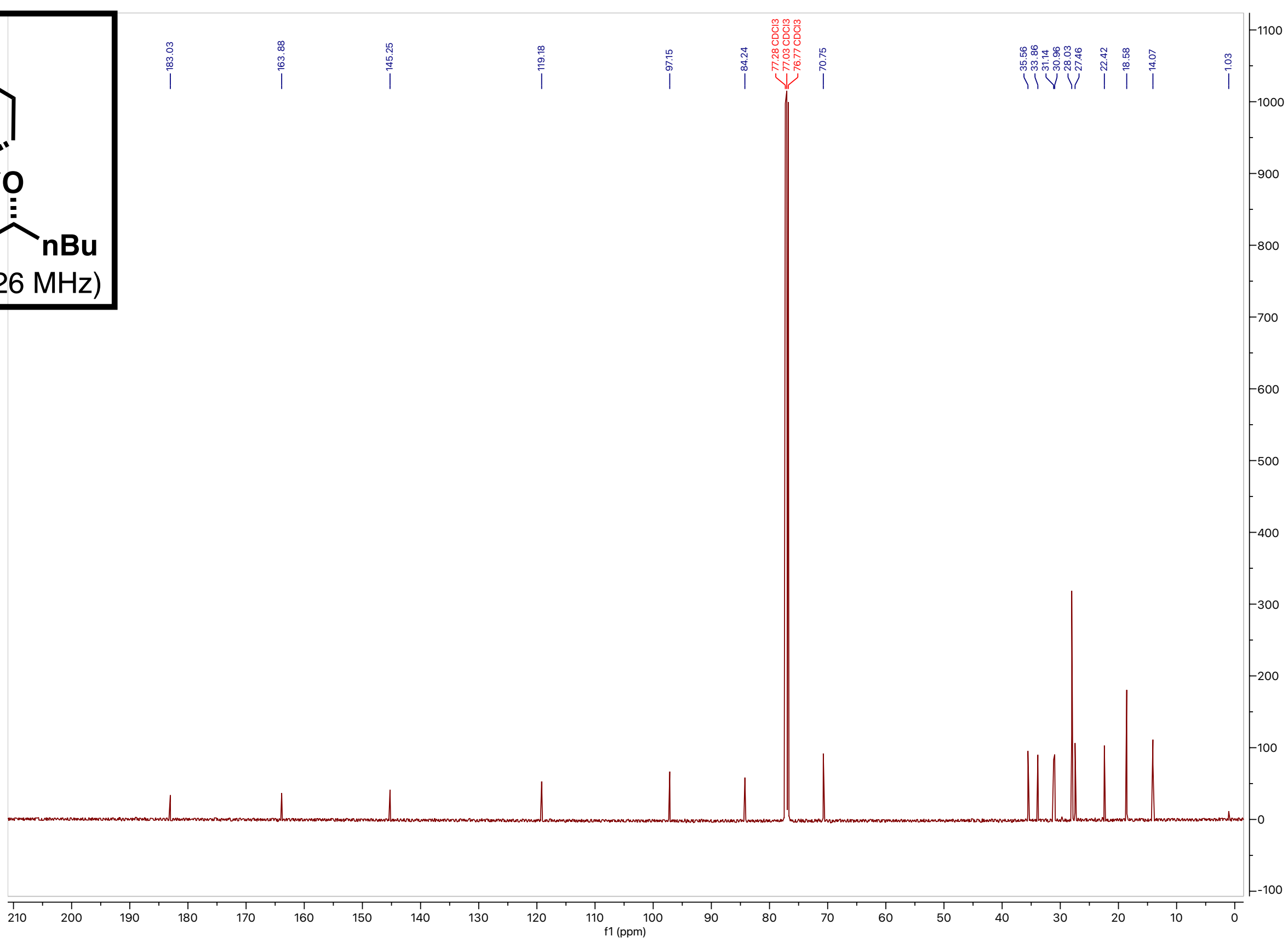

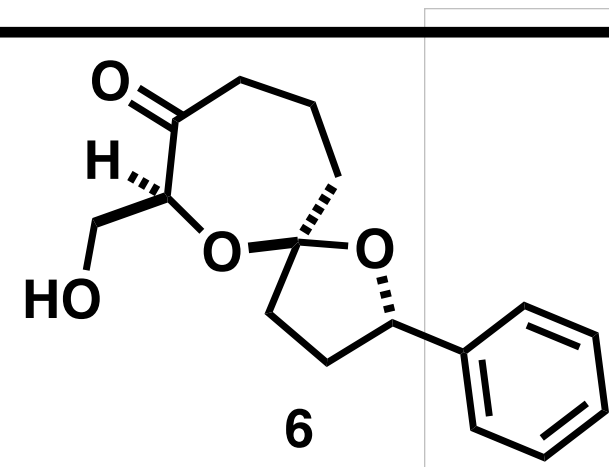

6

(^{1}H NMR, $CDCl_3$, 500 MHz)

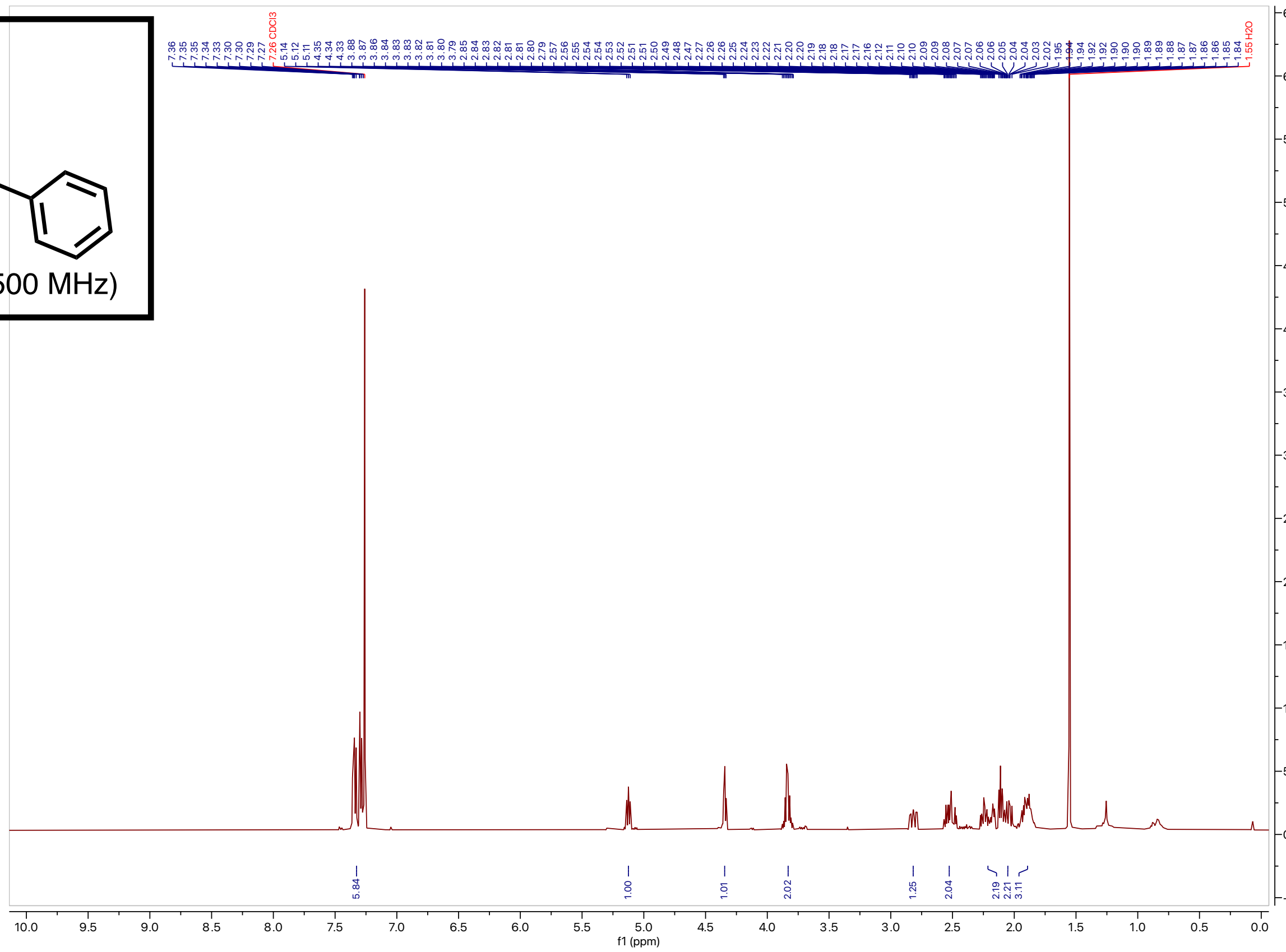

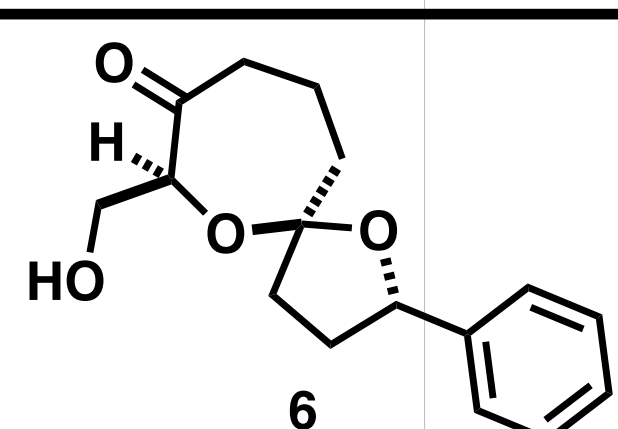

6

(^{13}C NMR, $CDCl_3$, 126 MHz)

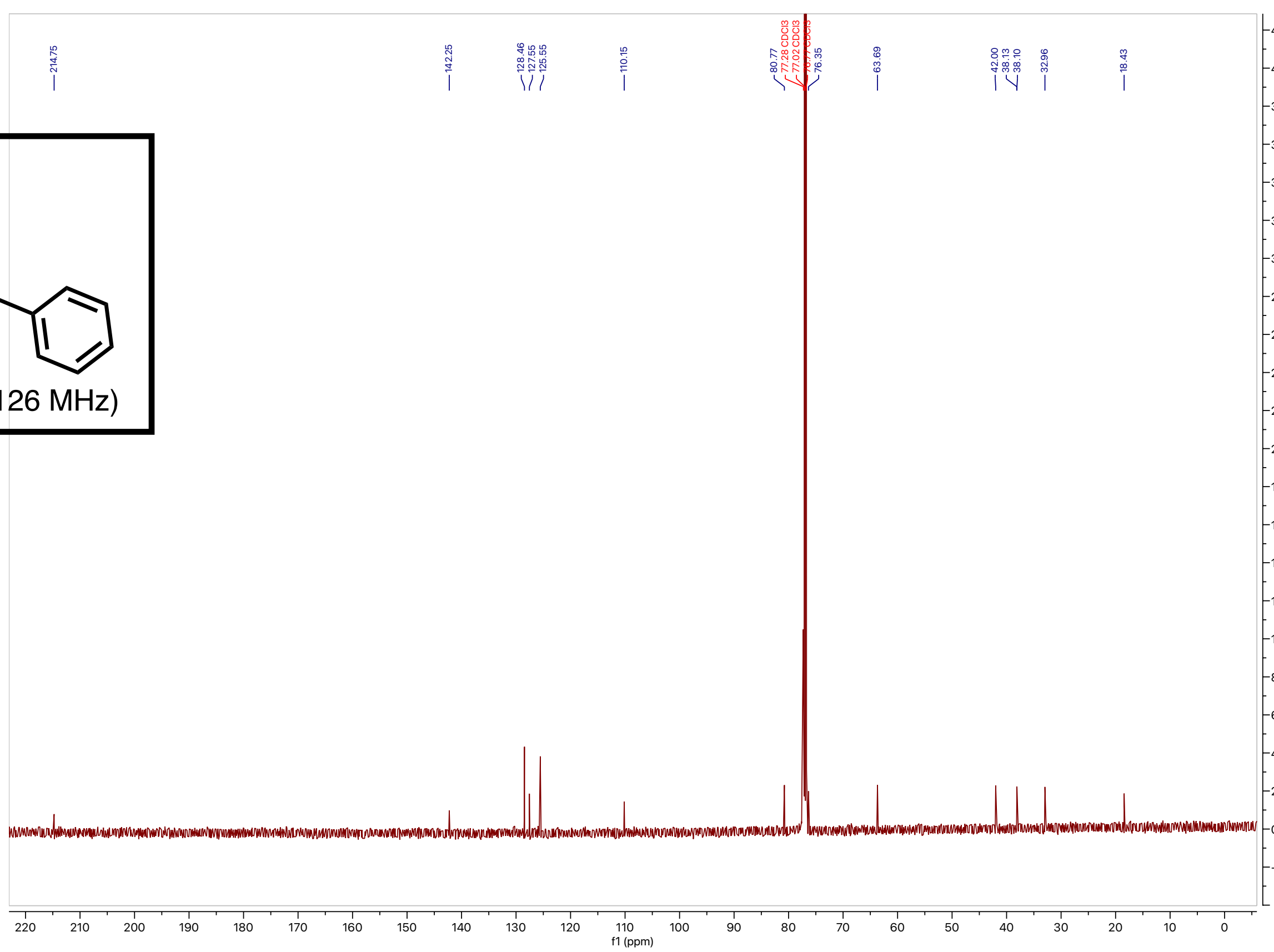

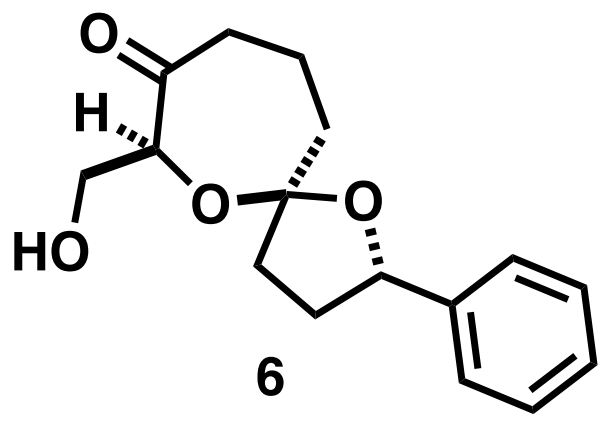

6

(^{1}H-^{13}C HSQC, $CDCl_3$)

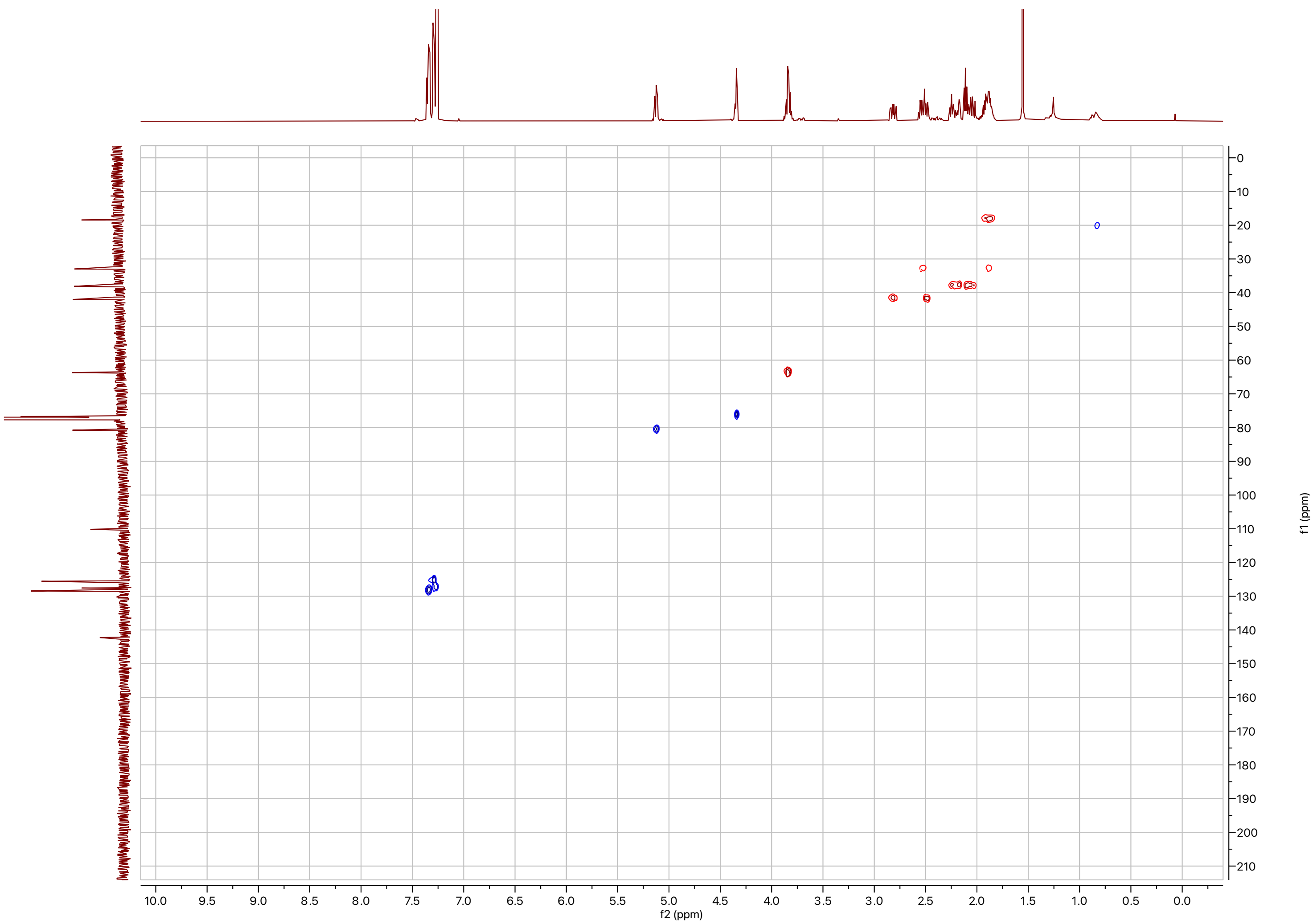

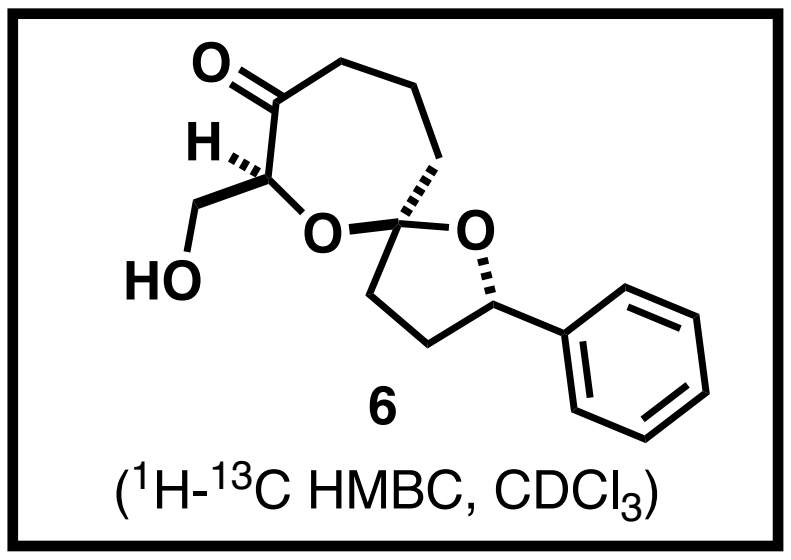
O
H
HO
O
O
6
(^{1}H-^{13}C HMBC, CDCl$_3$)

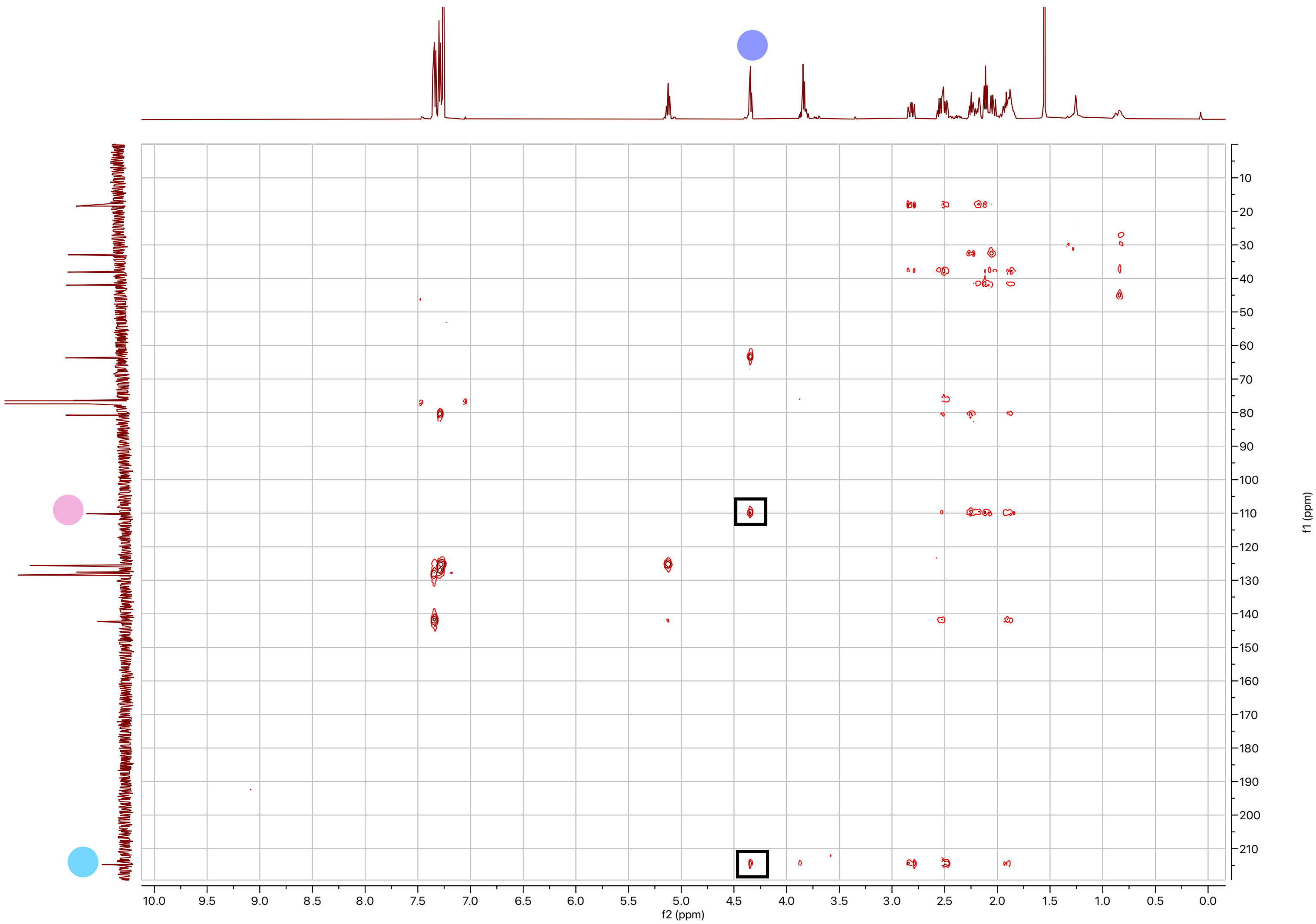
f2 (ppm)
f1 (ppm)

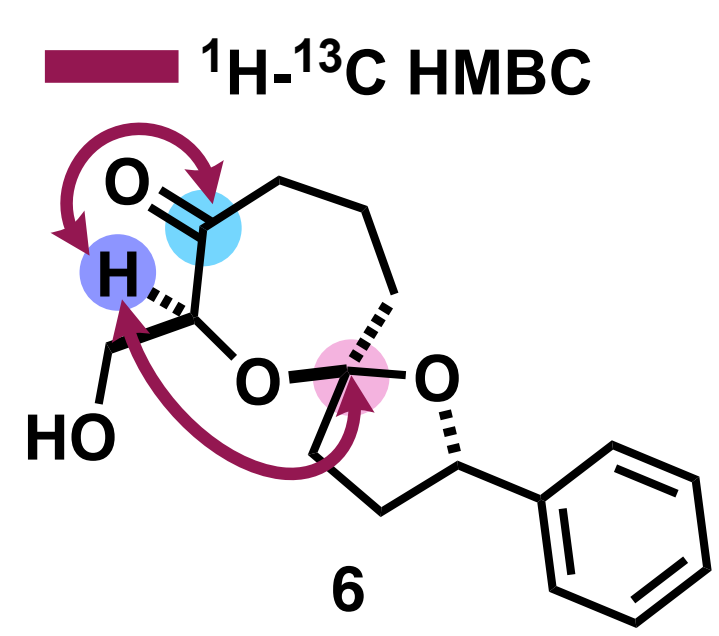
^{1}H-^{13}C HMBC
O
H
HO
O
O
6

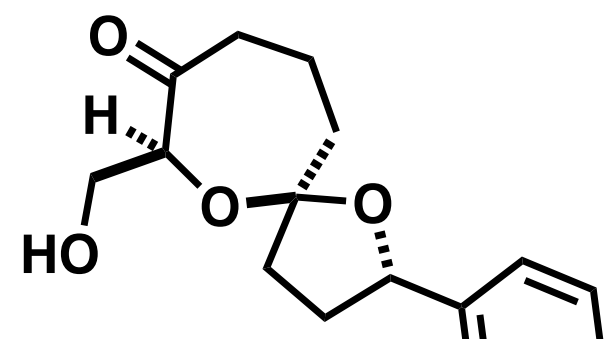

6

(^{1}H-^{1}H NOSEY, $CDCl_3$)

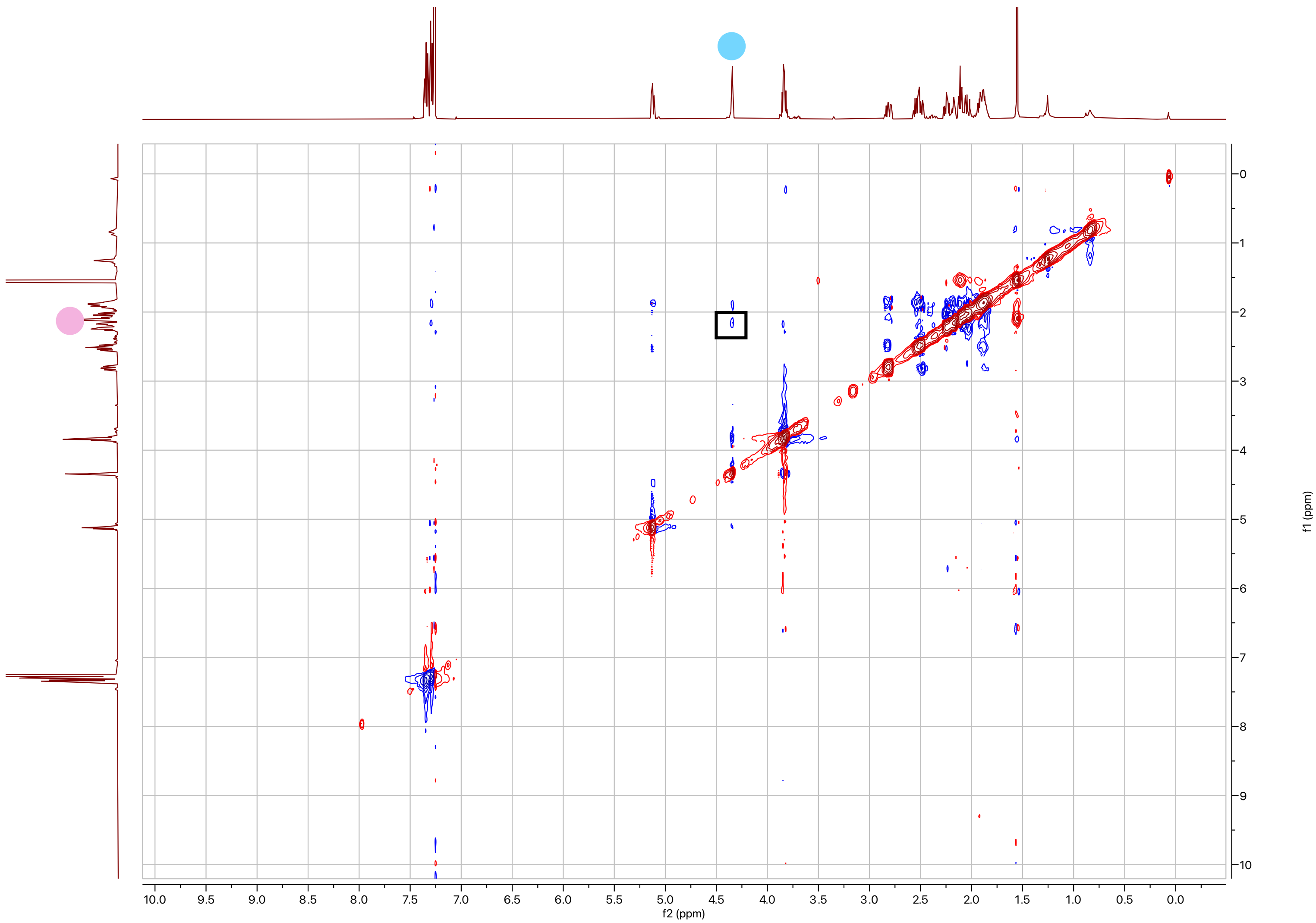

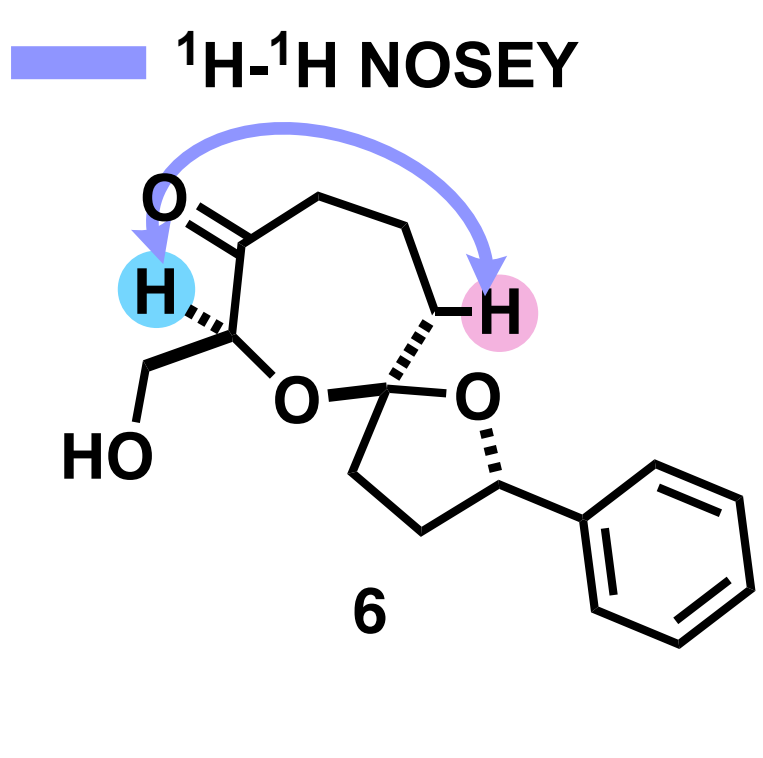

6

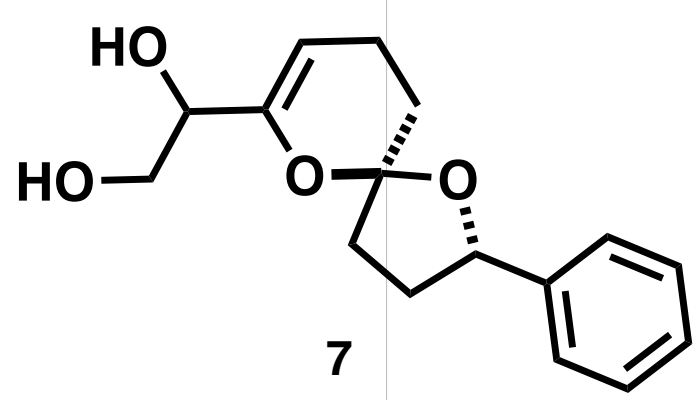

7

(^{1}H NMR, $CDCl_3$, 500 MHz)

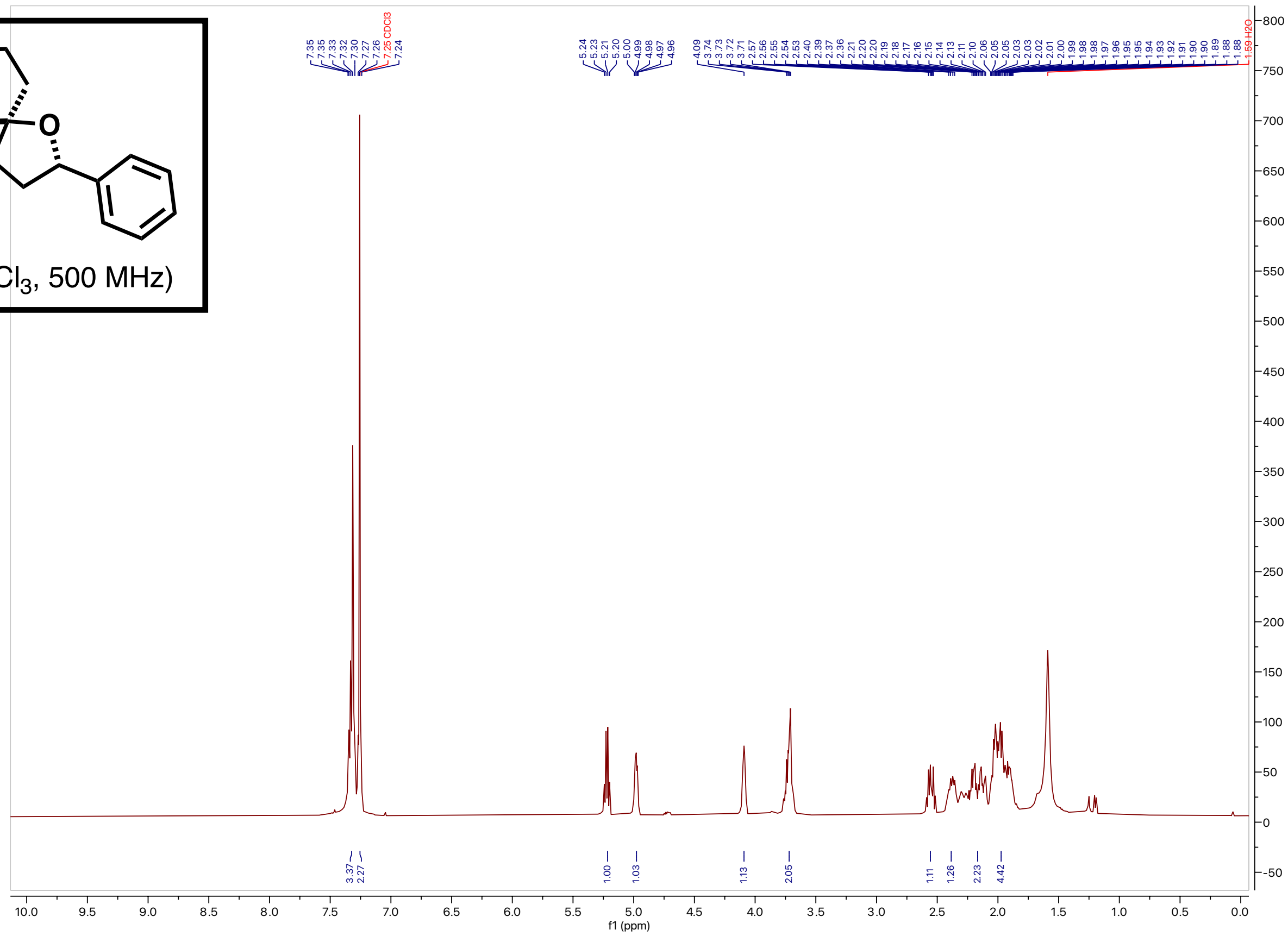

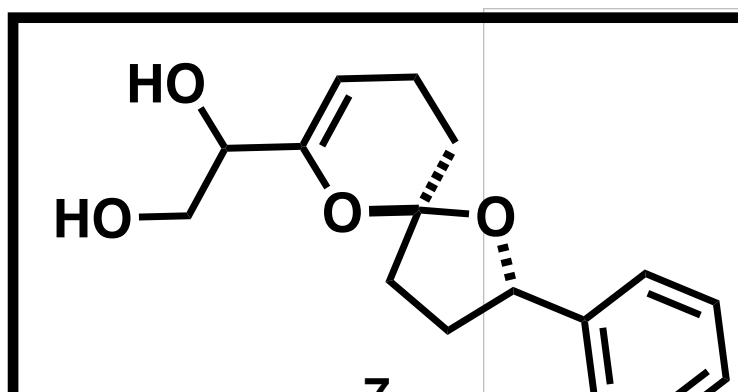

7

(^{13}C NMR, $CDCl_3$, 126 MHz)

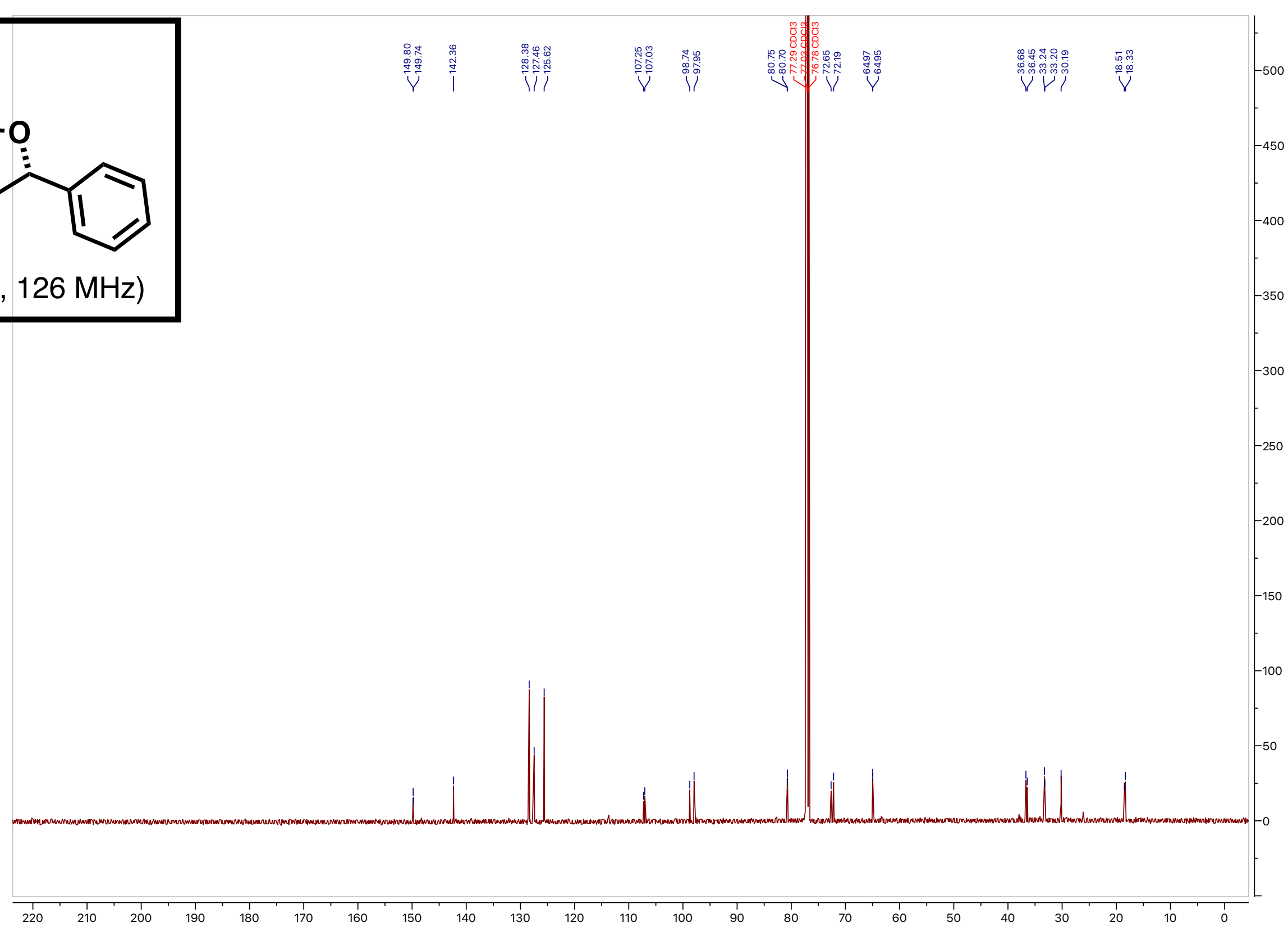

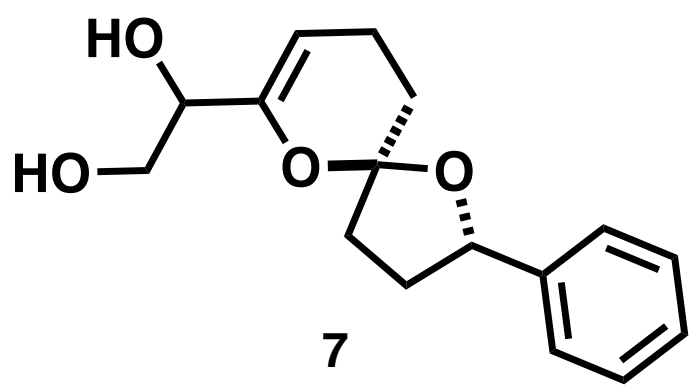

7

(^{1}H-^{13}C HSQC, $CDCl_3$)

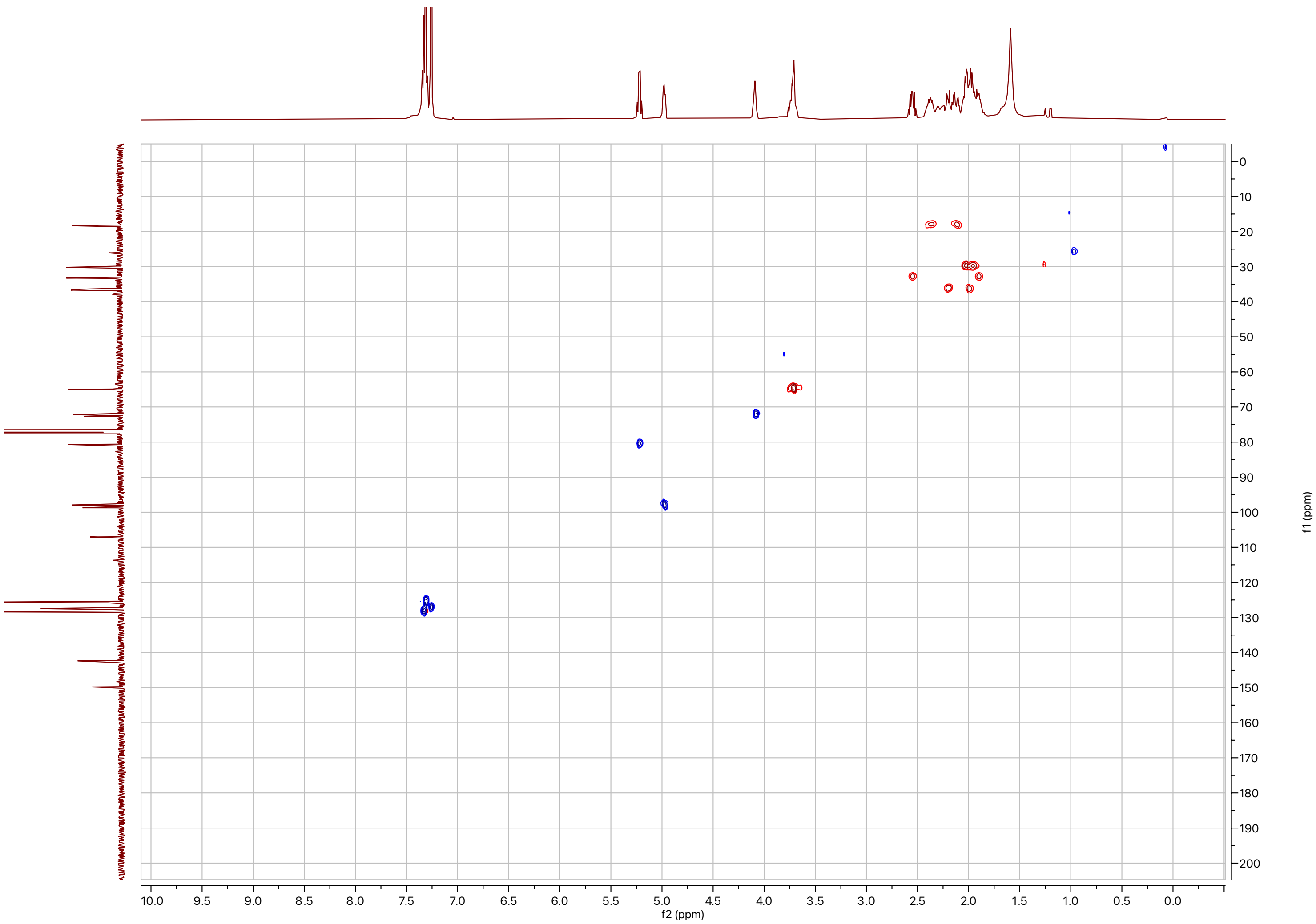

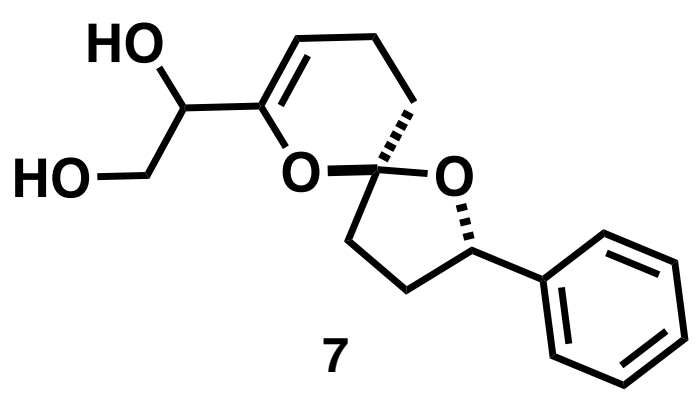

7

(^{1}H-^{13}C HMBC, $CDCl_3$)

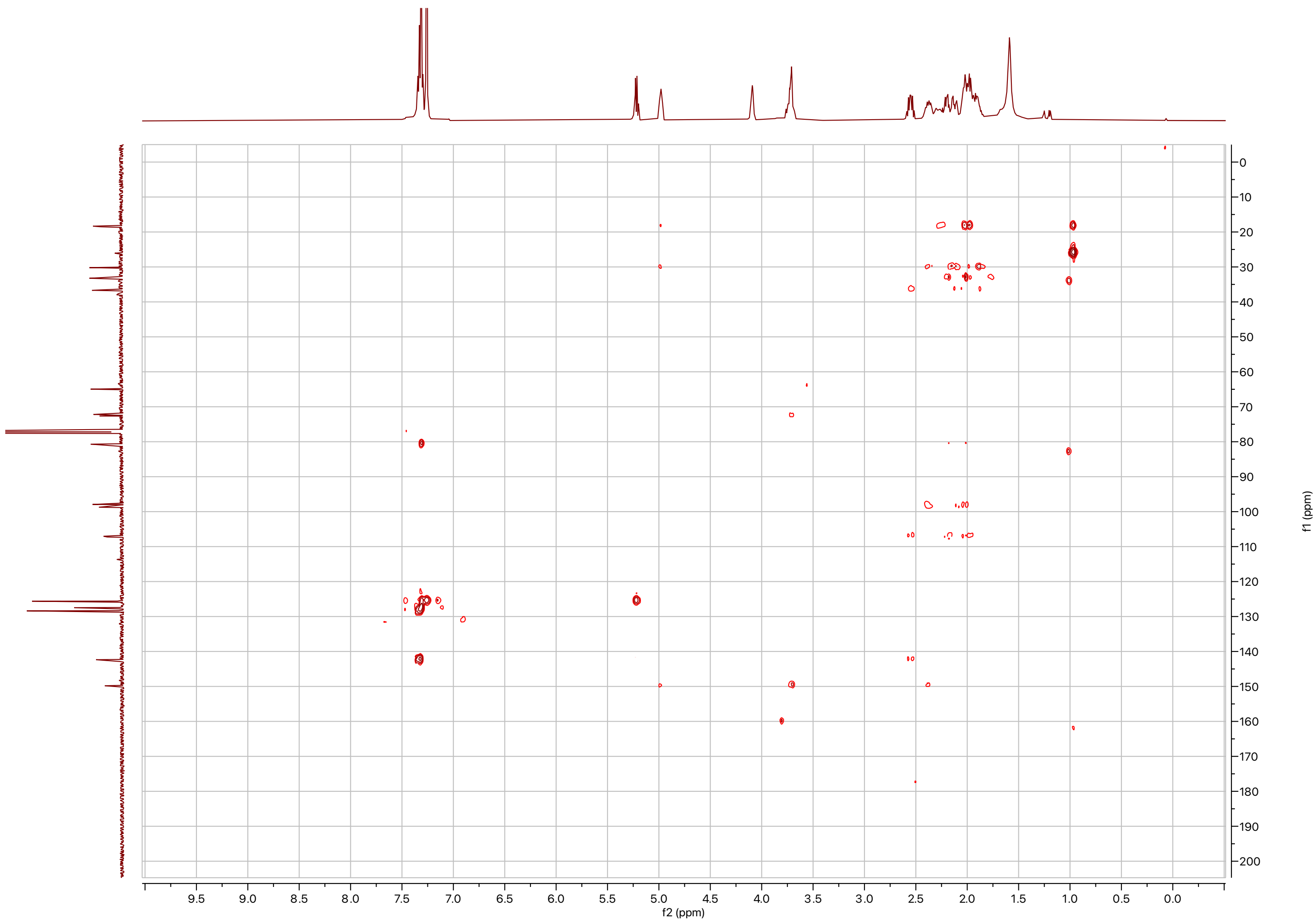

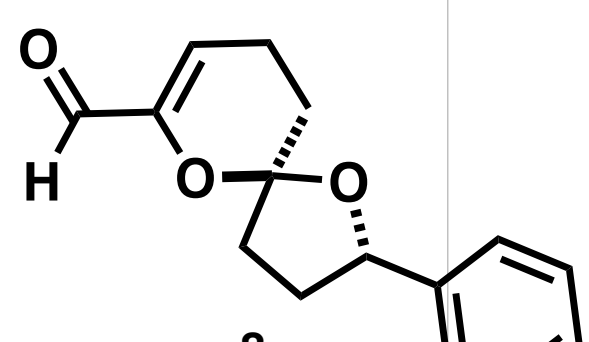

8

(^{1}H NMR, $CDCl_3$, 500 MHz)

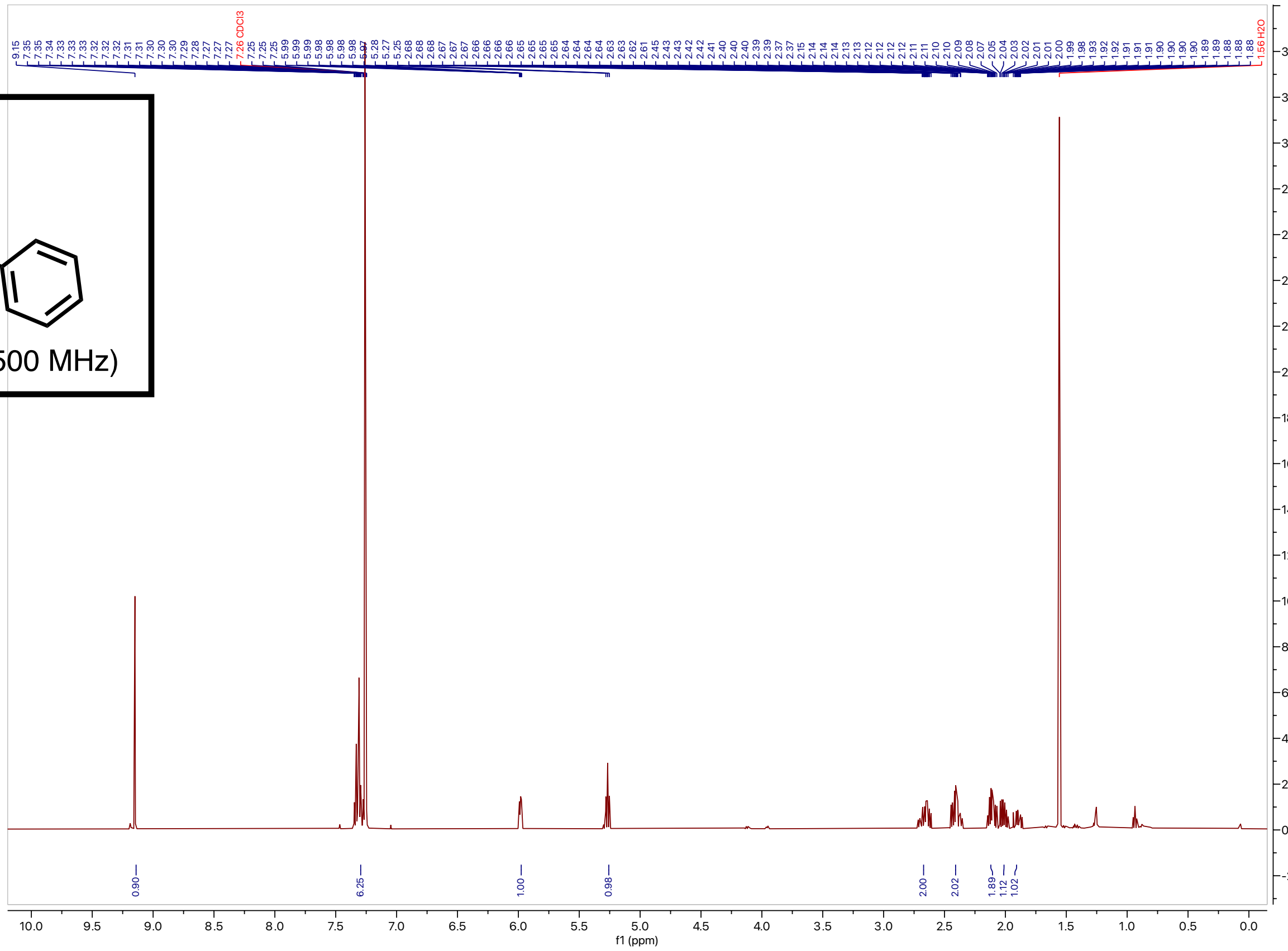

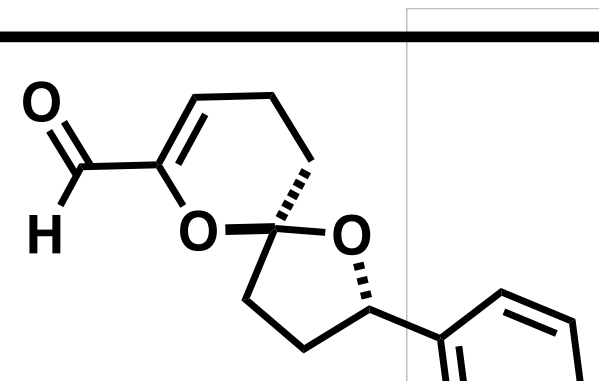

8

(^{13}C NMR, $CDCl_3$, 126 MHz)

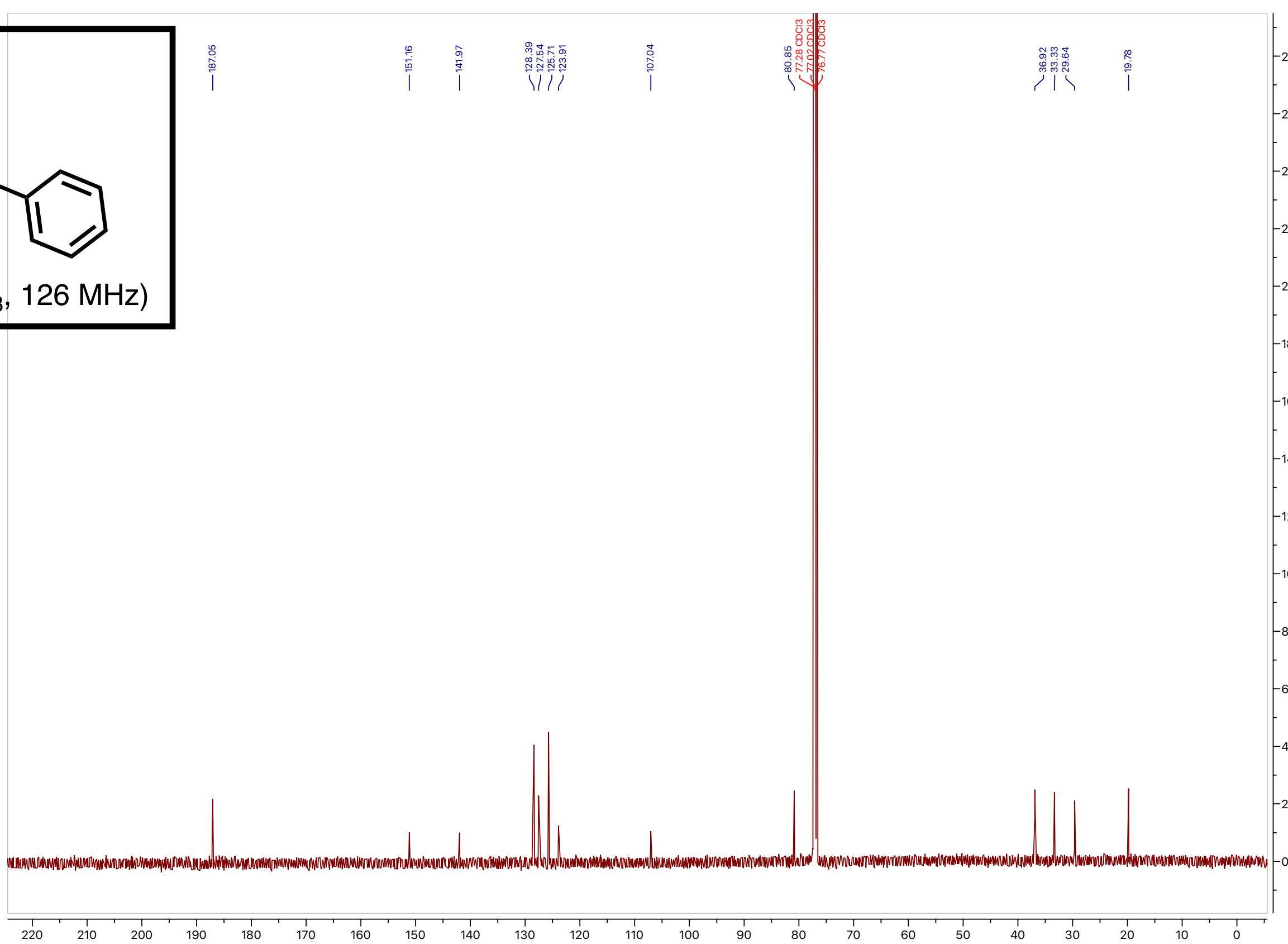

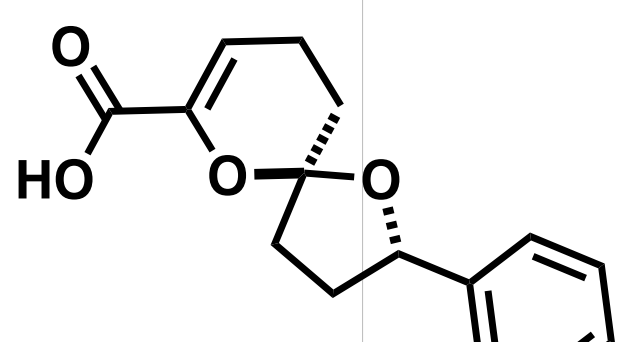

9

(^{1}H NMR, $CDCl_3$, 500 MHz)

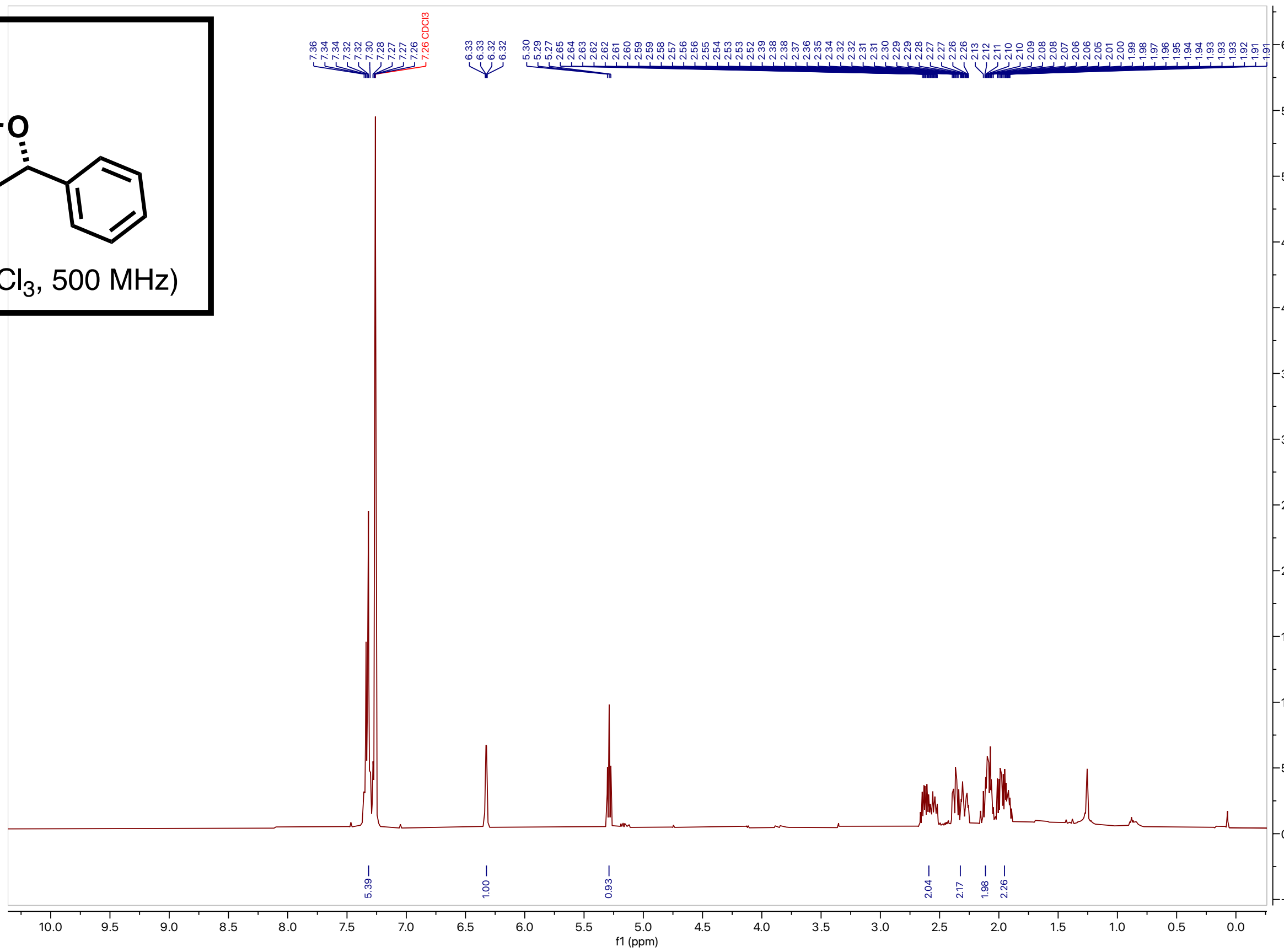

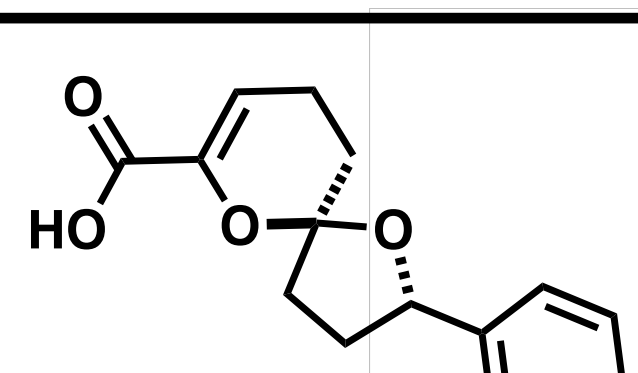

9

(^{13}C NMR, $CDCl_3$, 126 MHz)

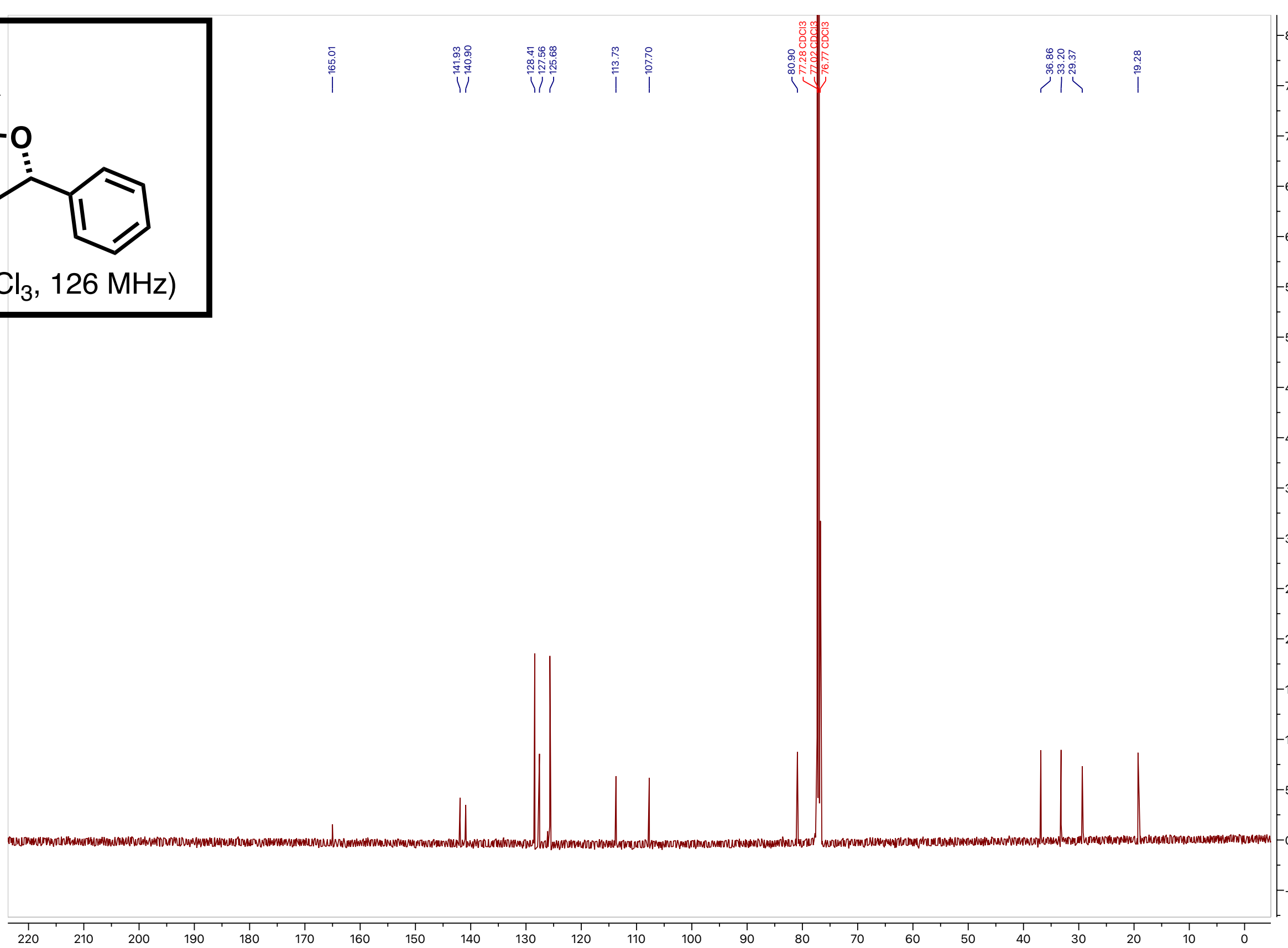

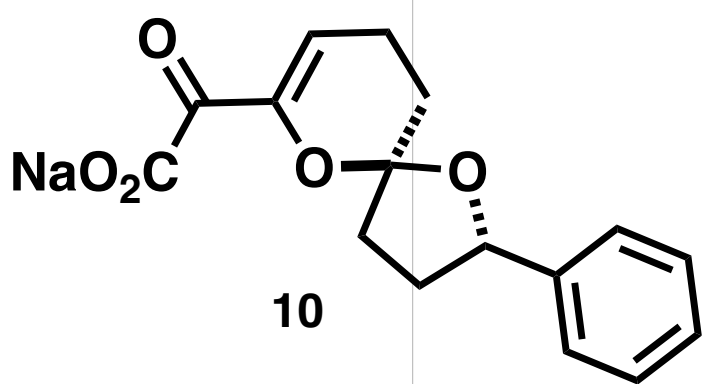

(^{1}H NMR, DMSO-d6, 500 MHz, crude, not isolated)

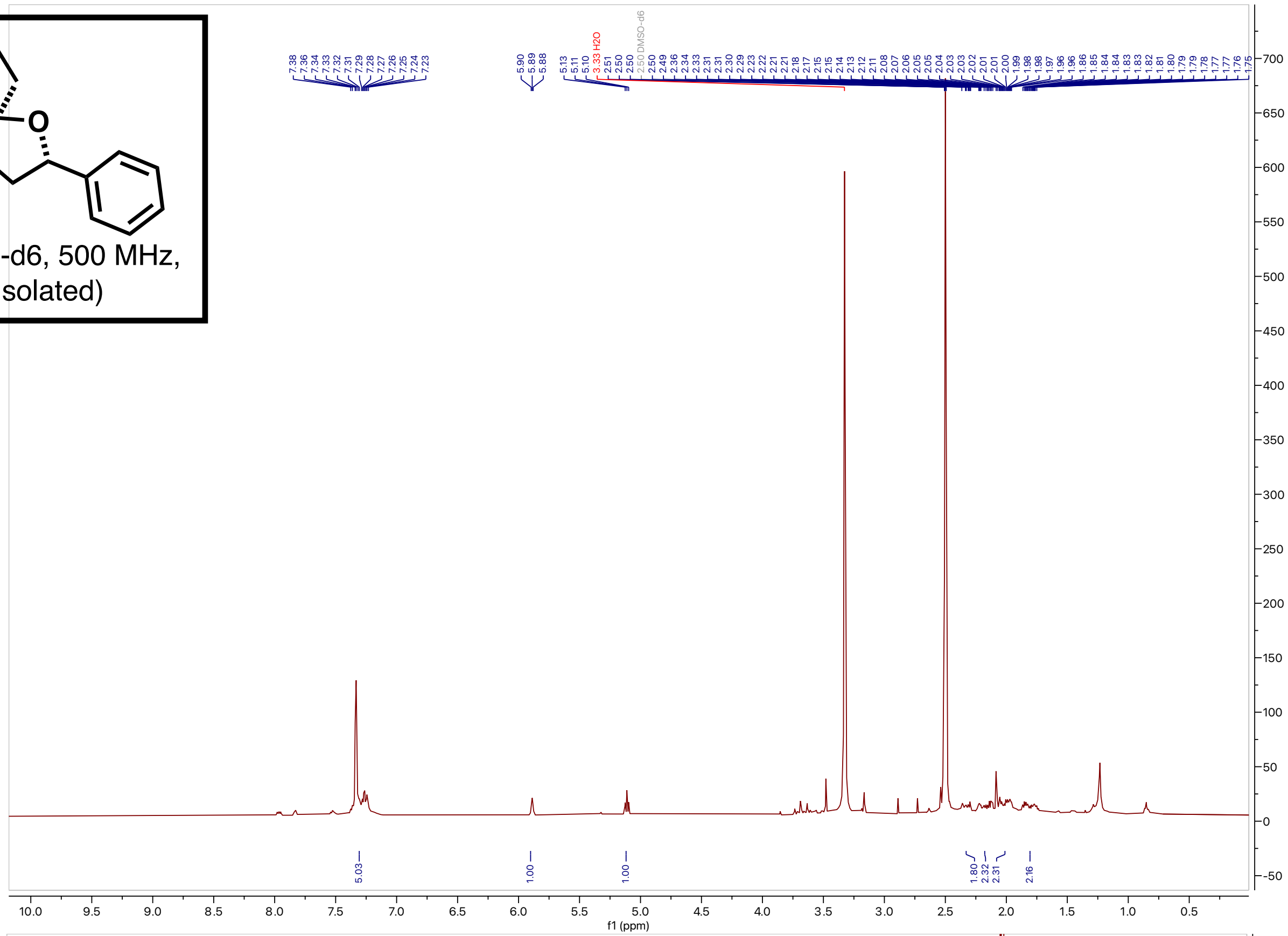

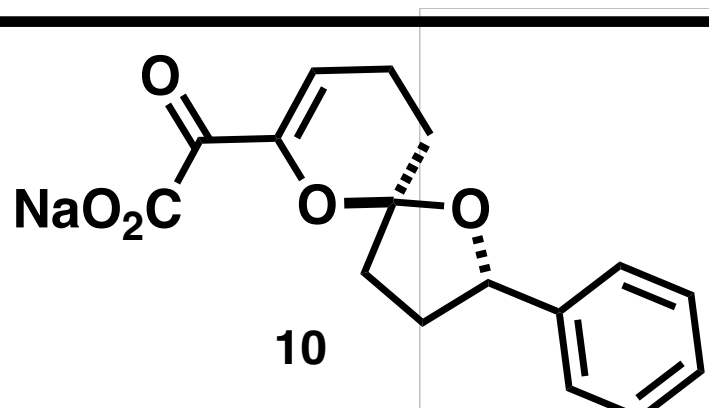

(^{13}C NMR, DMSO-d6, 126 MHz, crude, not isolated)

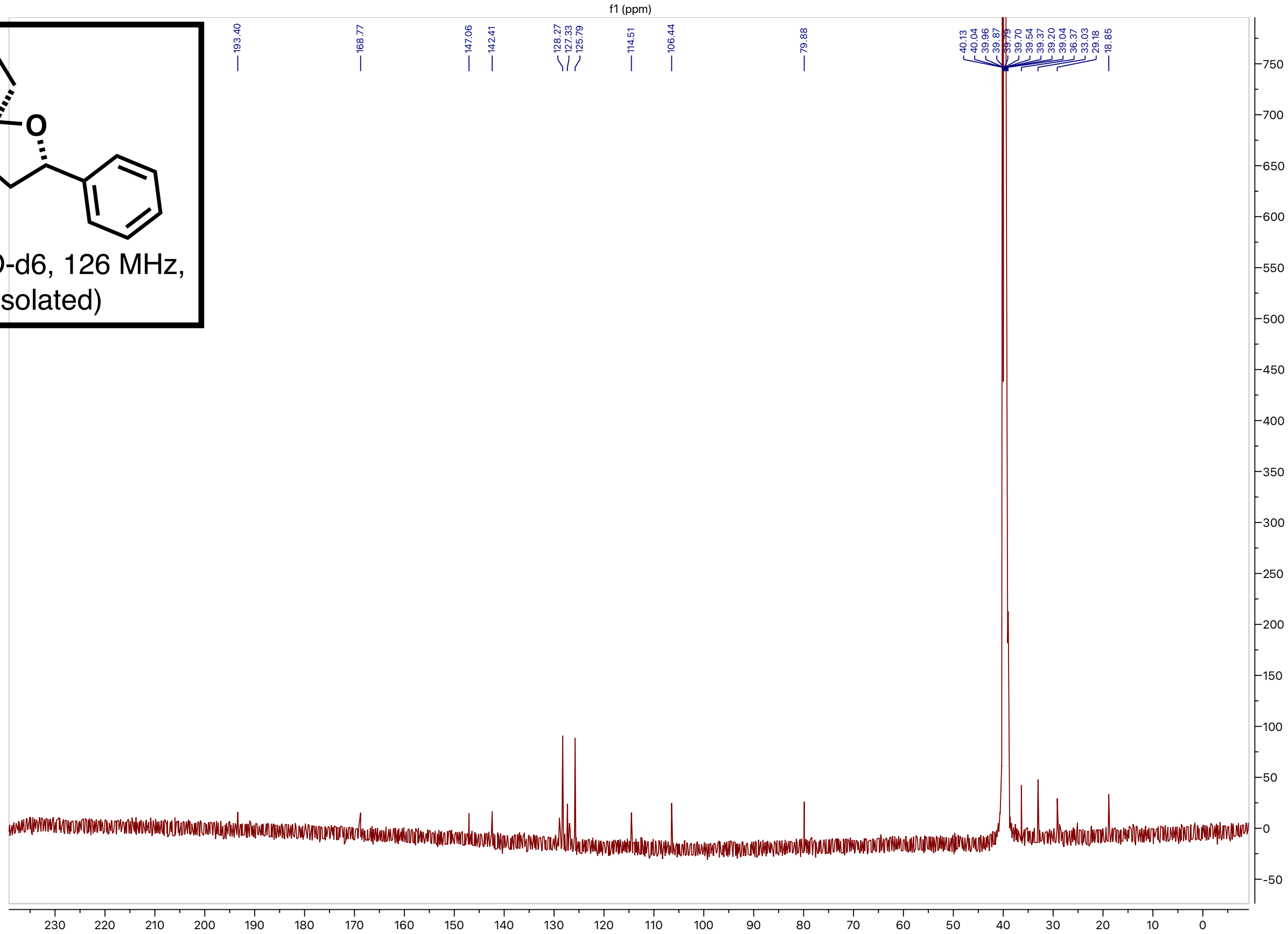

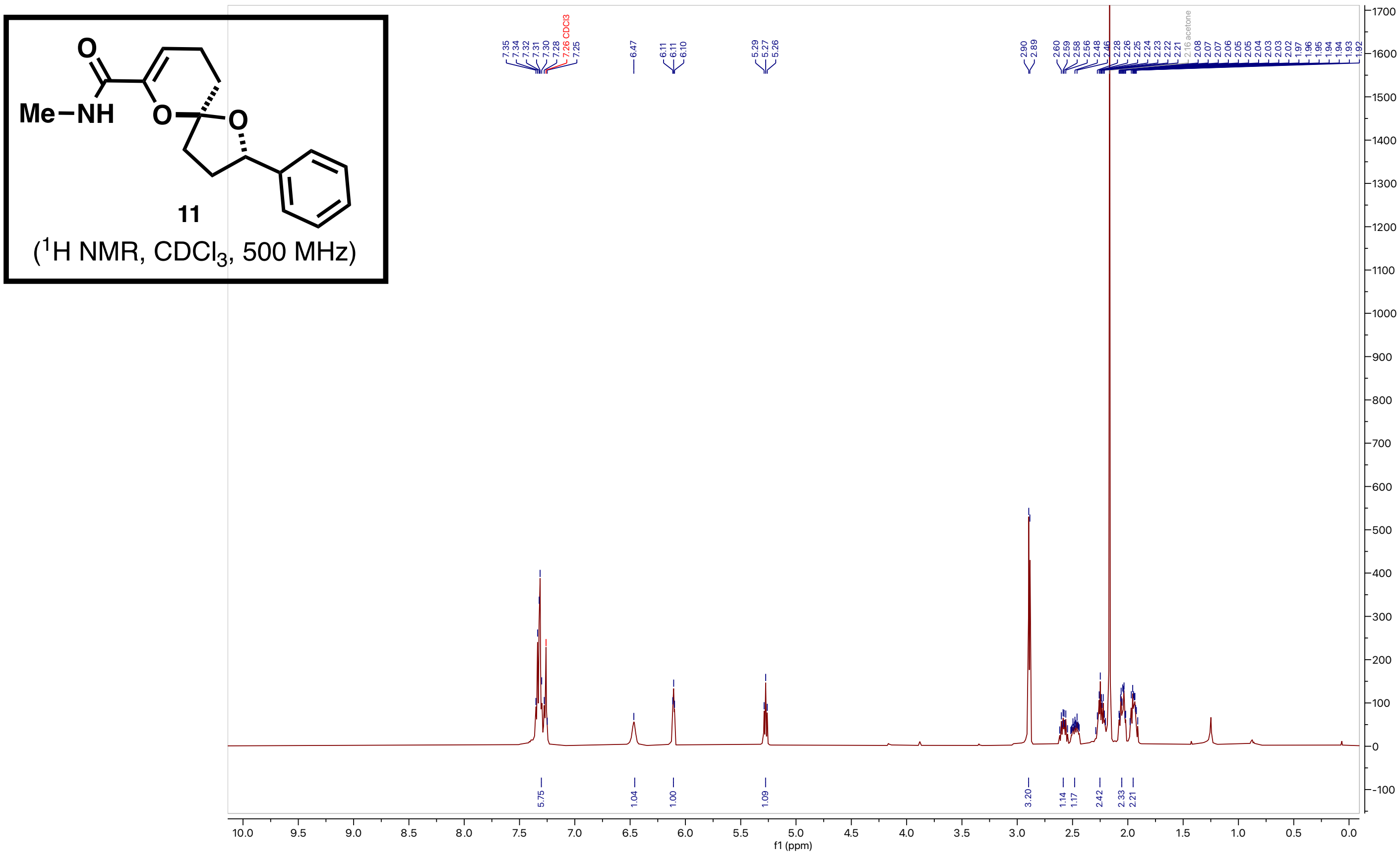
O
Me−NH
O
O
11
(¹H NMR, CDCl₃, 500 MHz)
f1 (ppm)

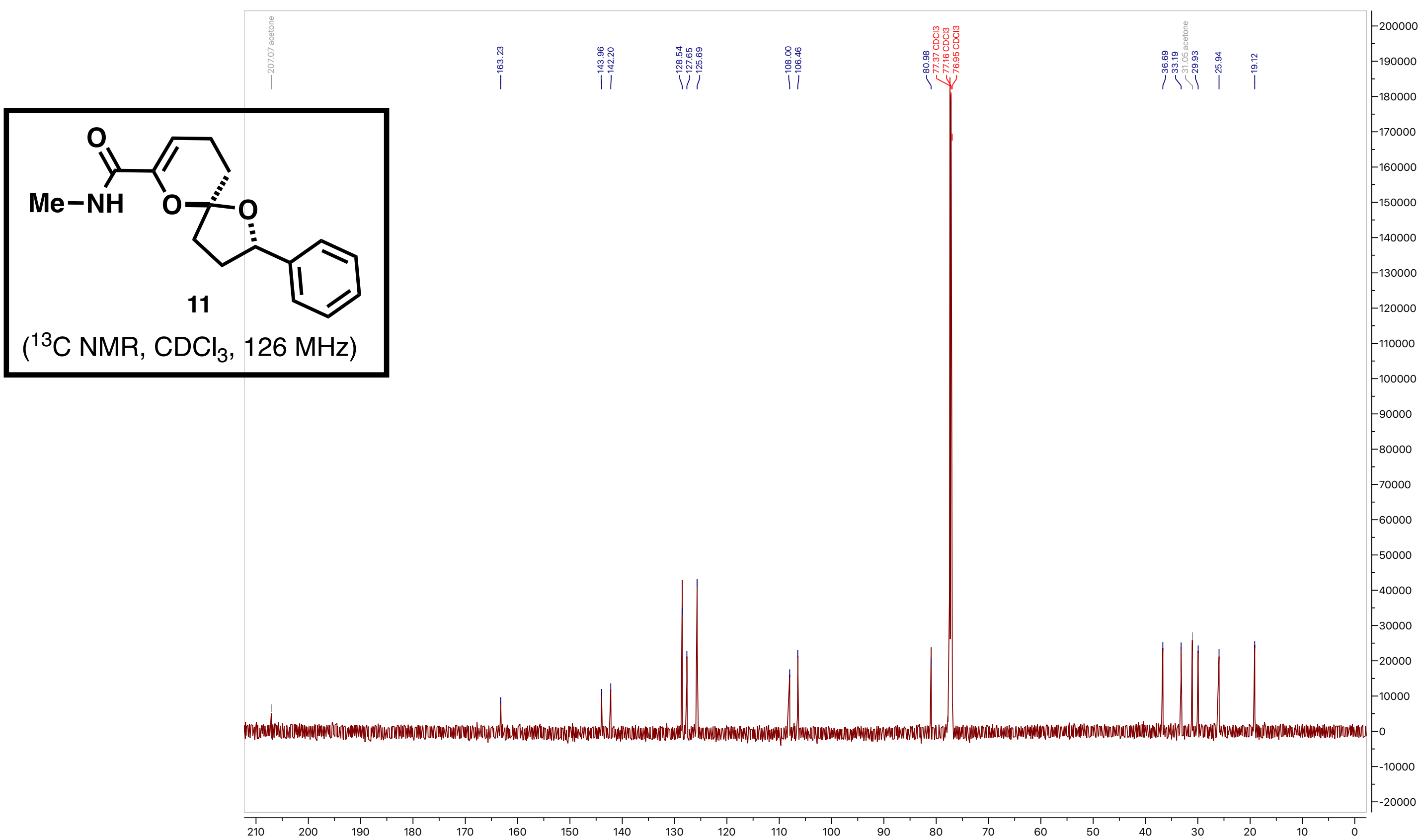
O
Me−NH
O
O
11
(¹³C NMR, CDCl₃, 126 MHz)
f1 (ppm)

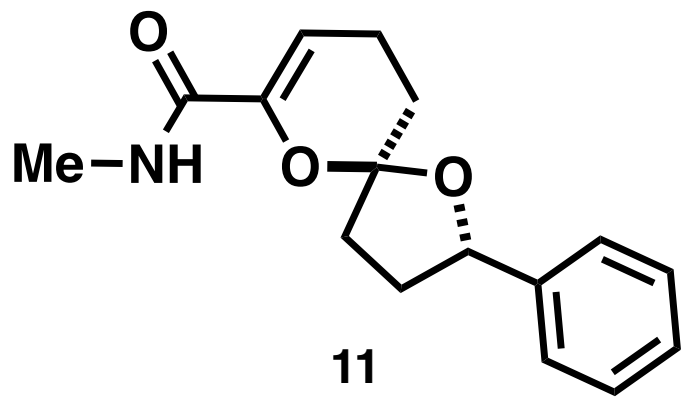

11

(^{1}H-^{13}C HSQC, $CDCl_3$)

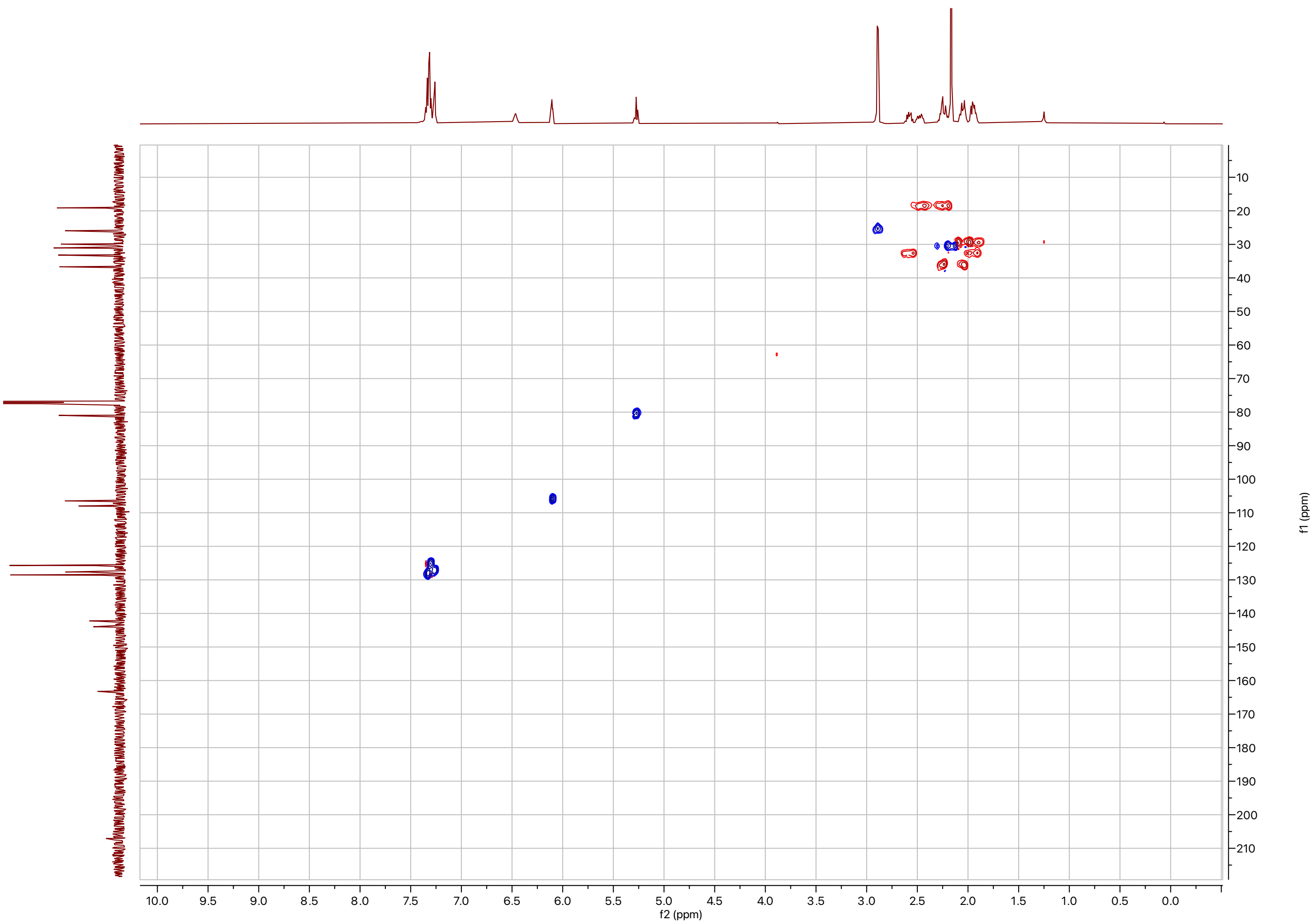

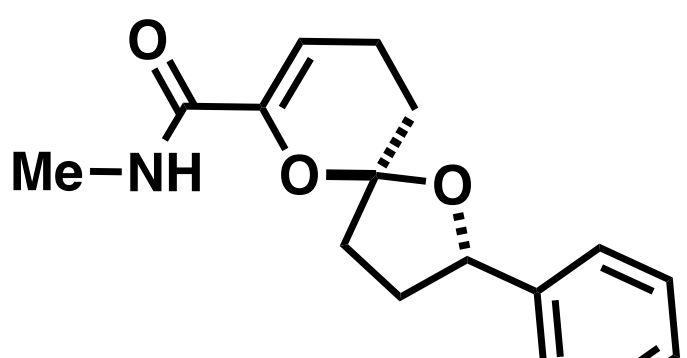

11

(^{1}H-^{13}C HMBC, $CDCl_3$)

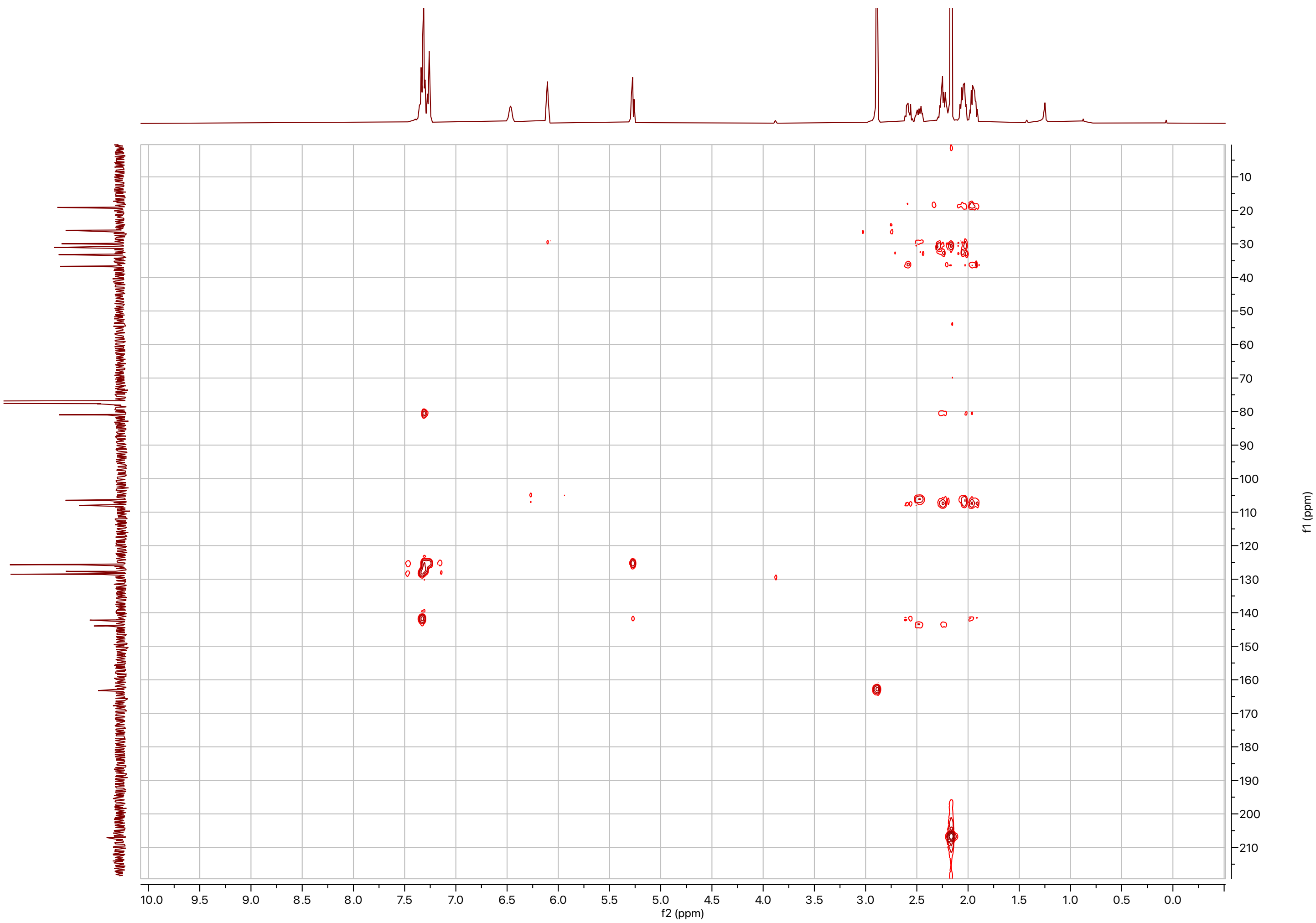

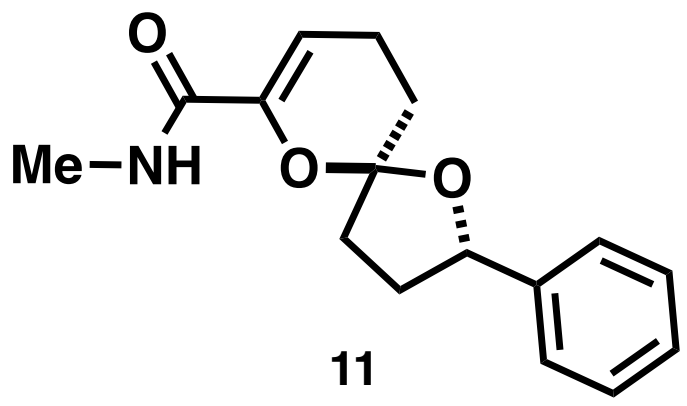

11

(^{1}H-^{1}H NOSEY, $CDCl_3$)

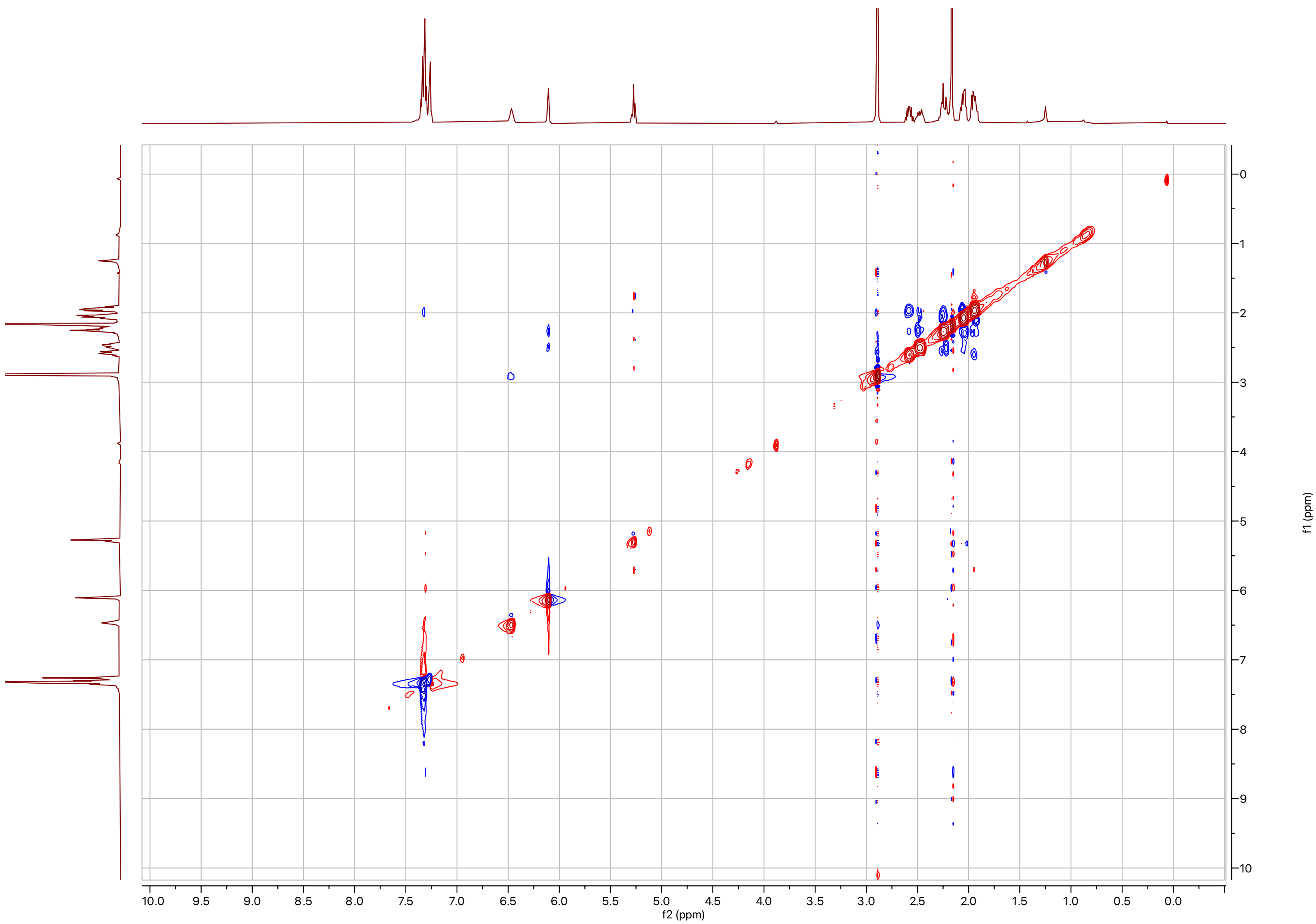

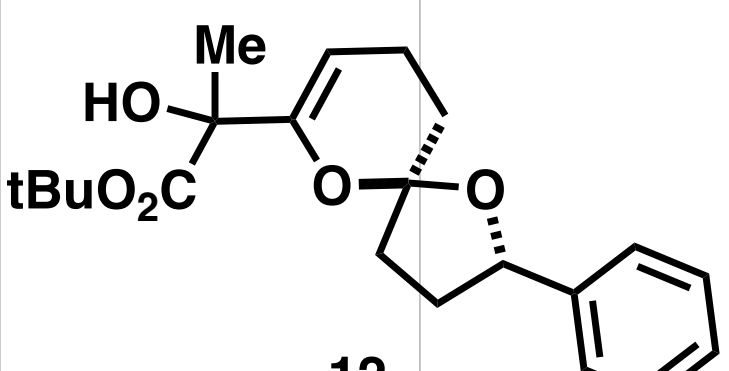

12

(^{1}H NMR, $CDCl_3$, 500 MHz, 82% purity)

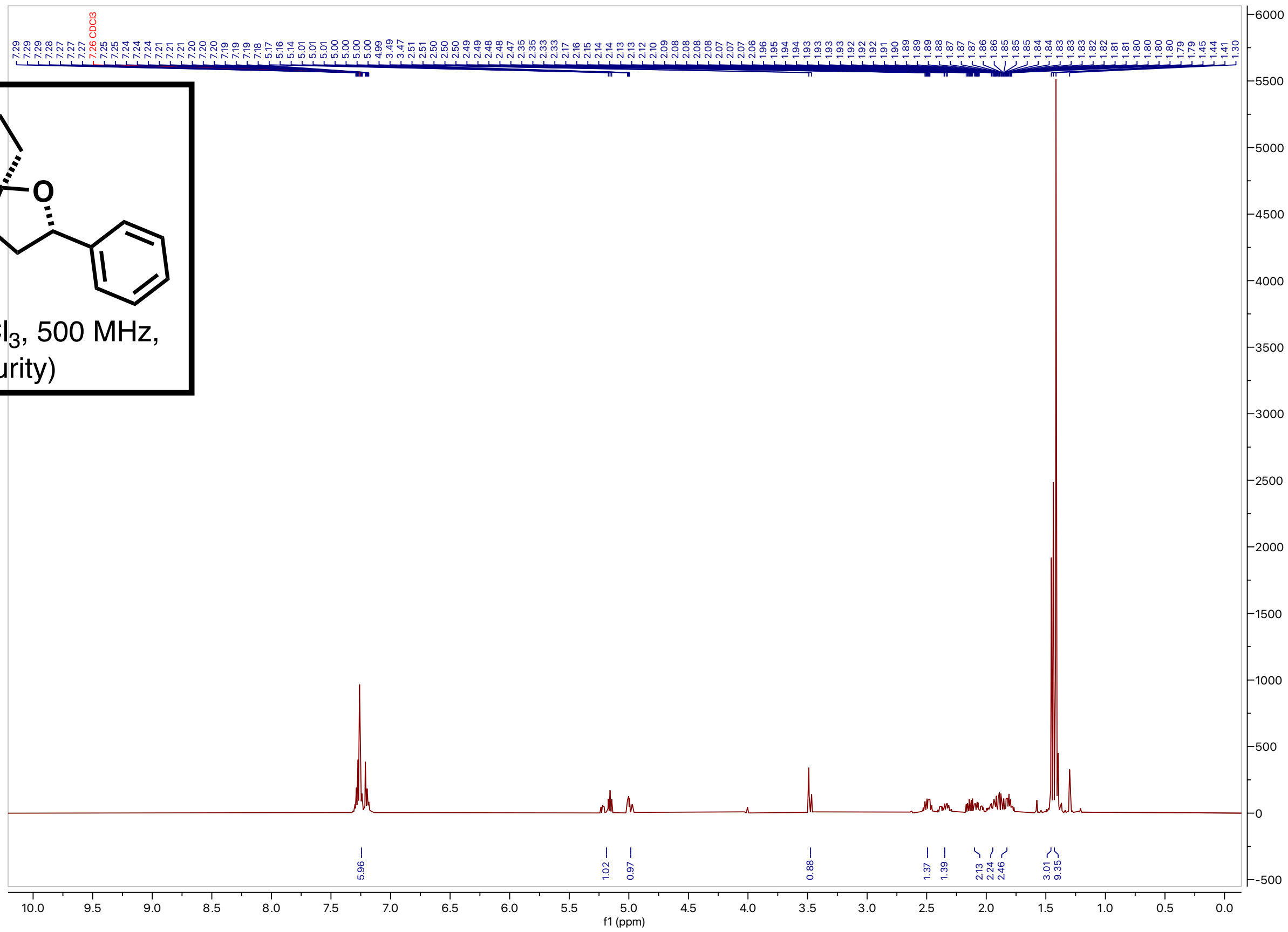

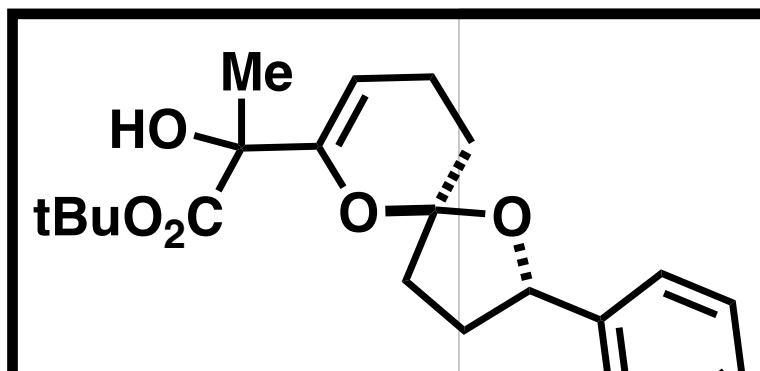

12

(^{13}C NMR, $CDCl_3$, 126 MHz, 82% purity)

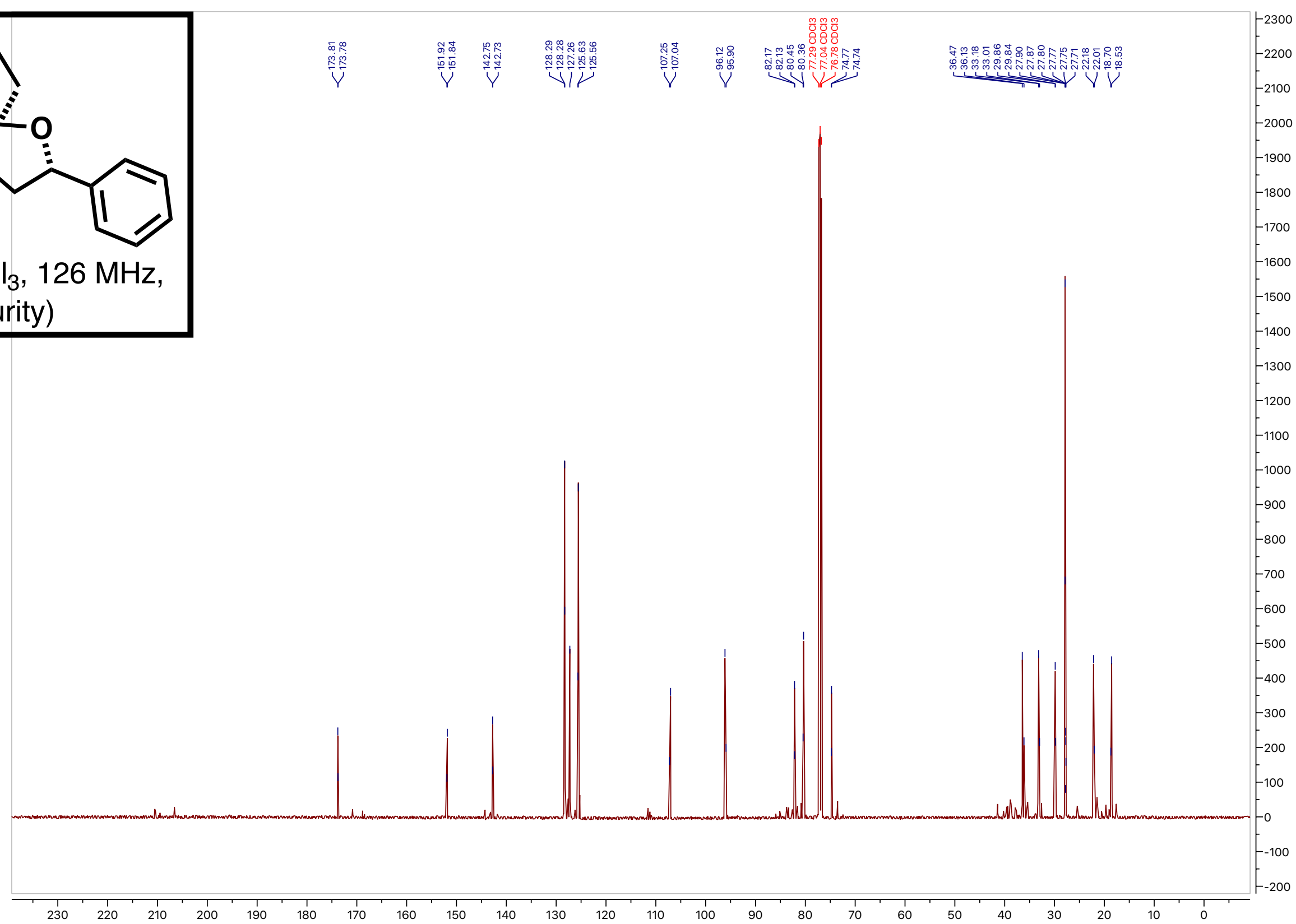

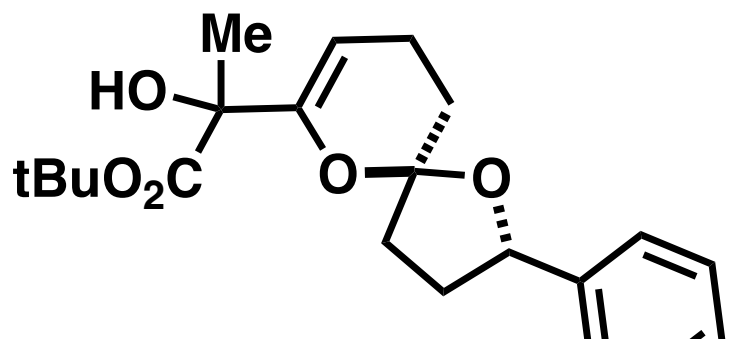

12

(^{1}H-^{13}C HSQC, $CDCl_3$,
82% purity)

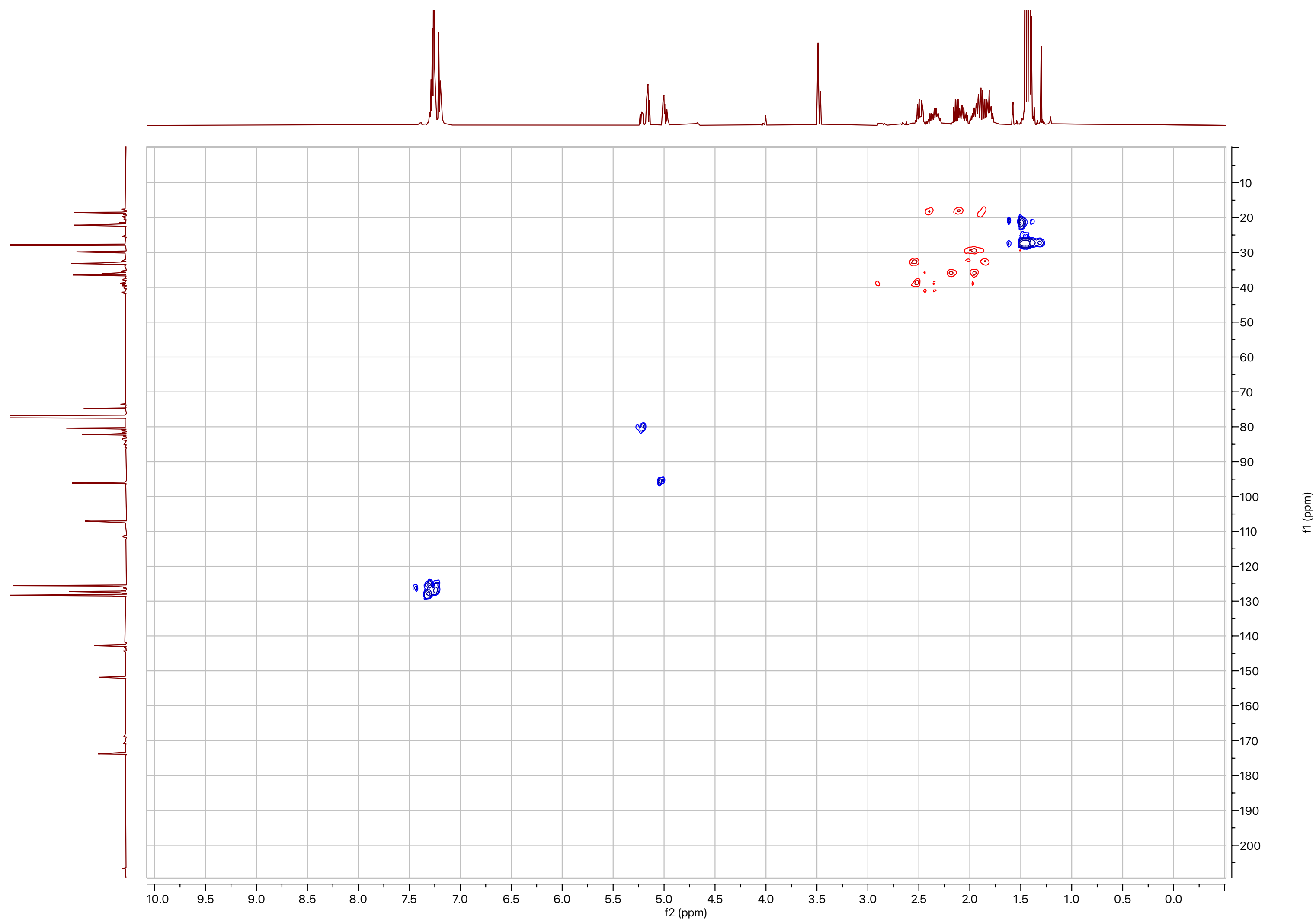

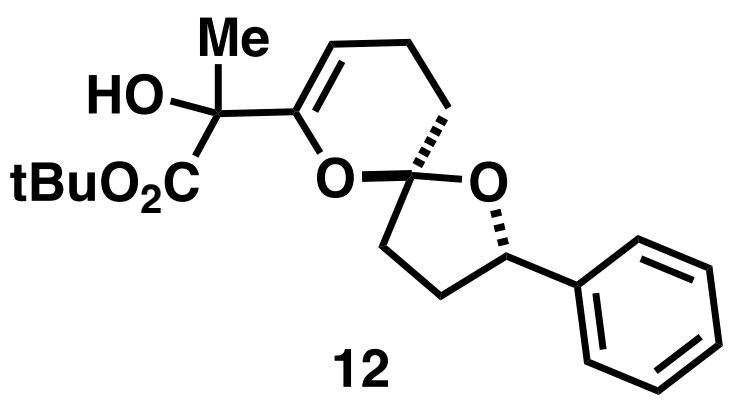

12

(^{1}H-^{13}C HMBC, $CDCl_3$,
82% purity)

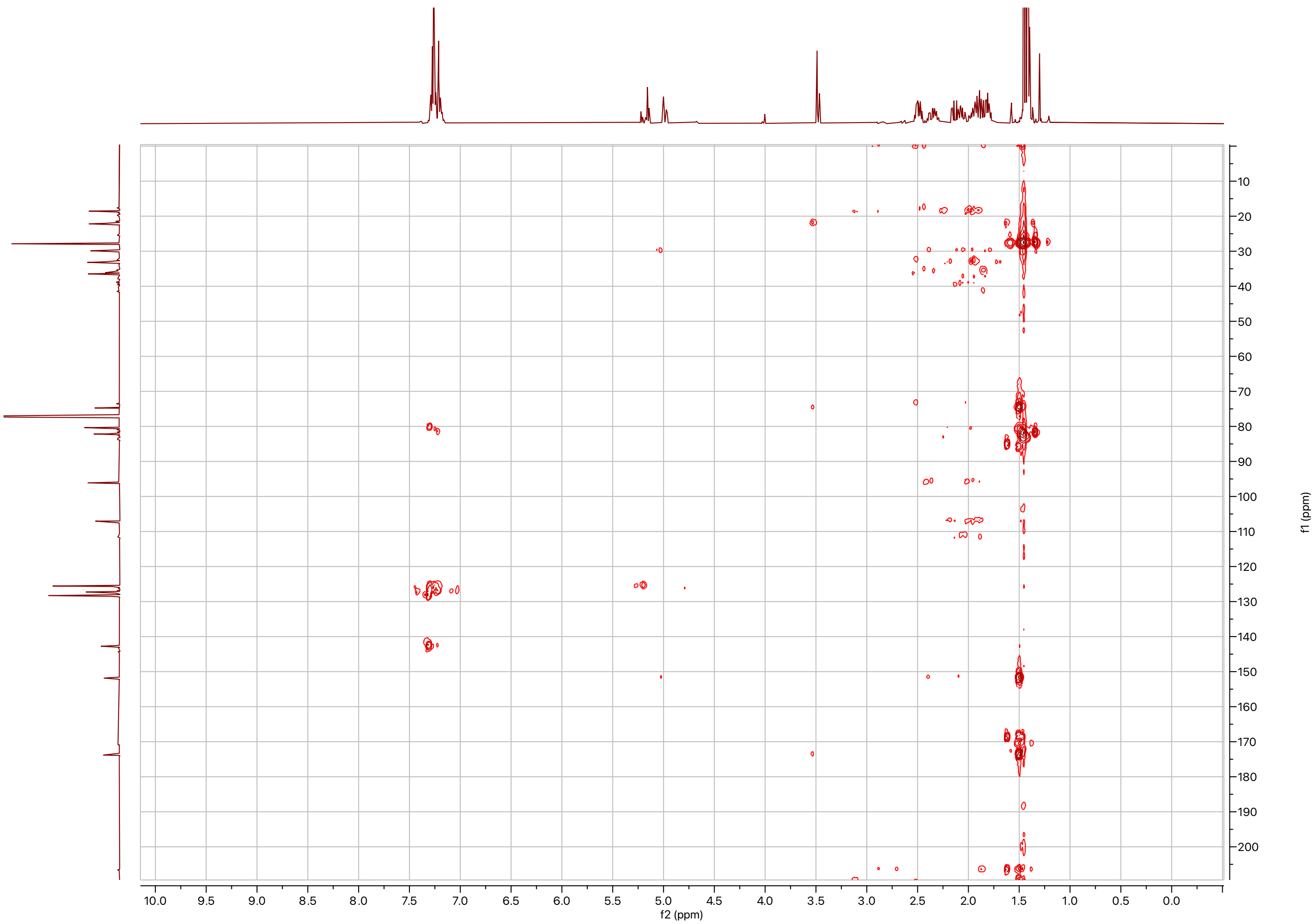

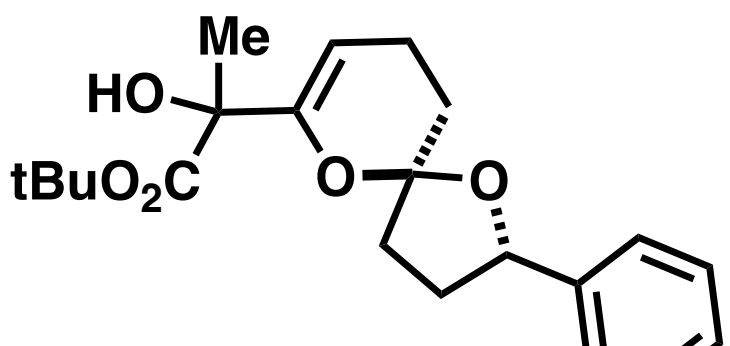

12

(^{1}H-^{1}H NOSEY, $CDCl_3$,
82% purity)

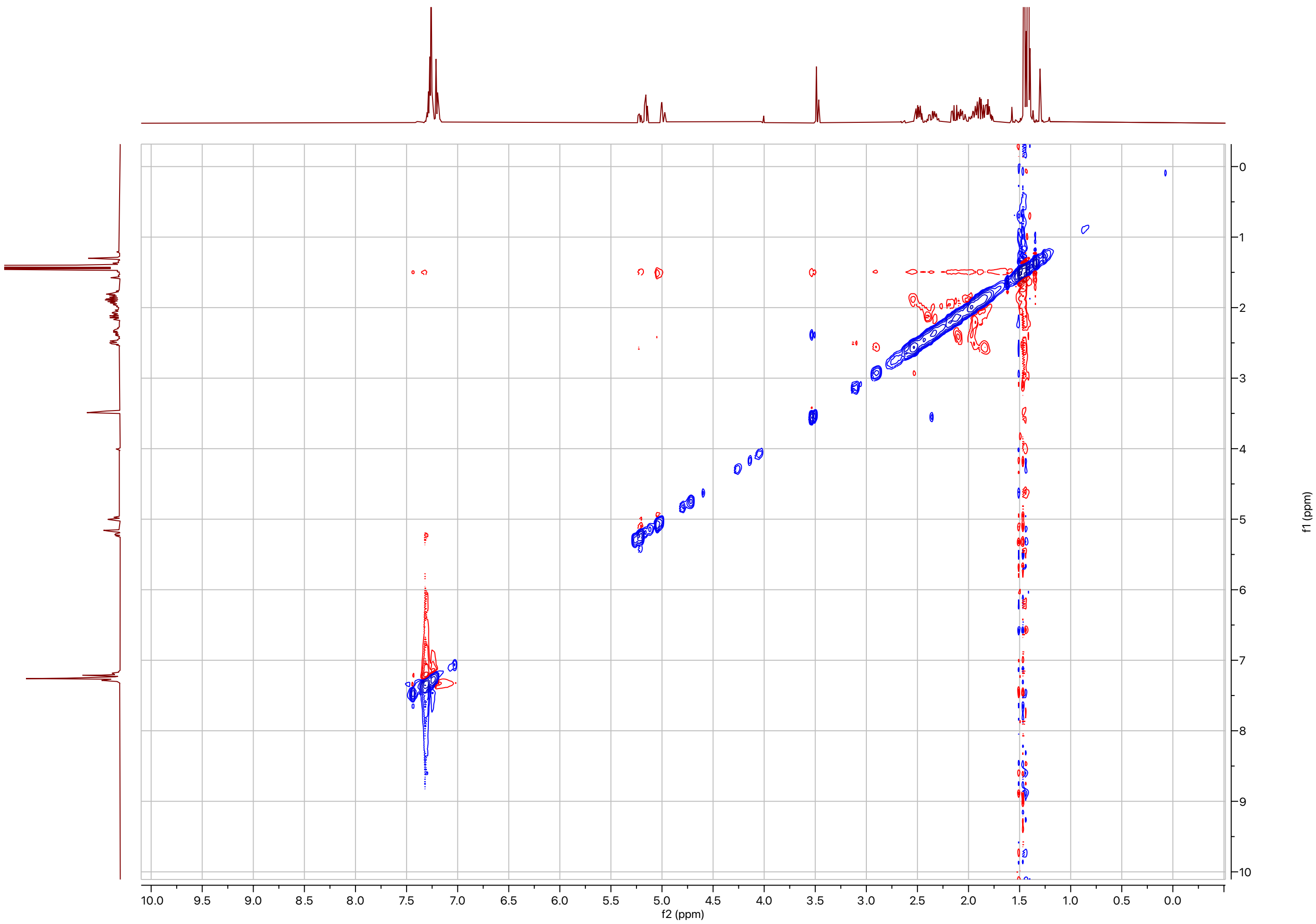

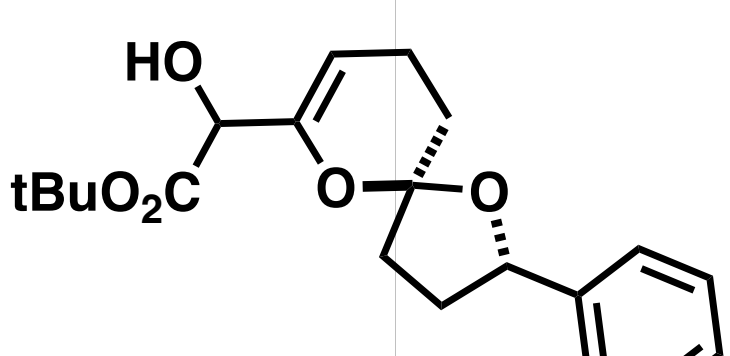

13
(^{1}H NMR, $CDCl_3$, 300 MHz, *mixture of diastereomers*)

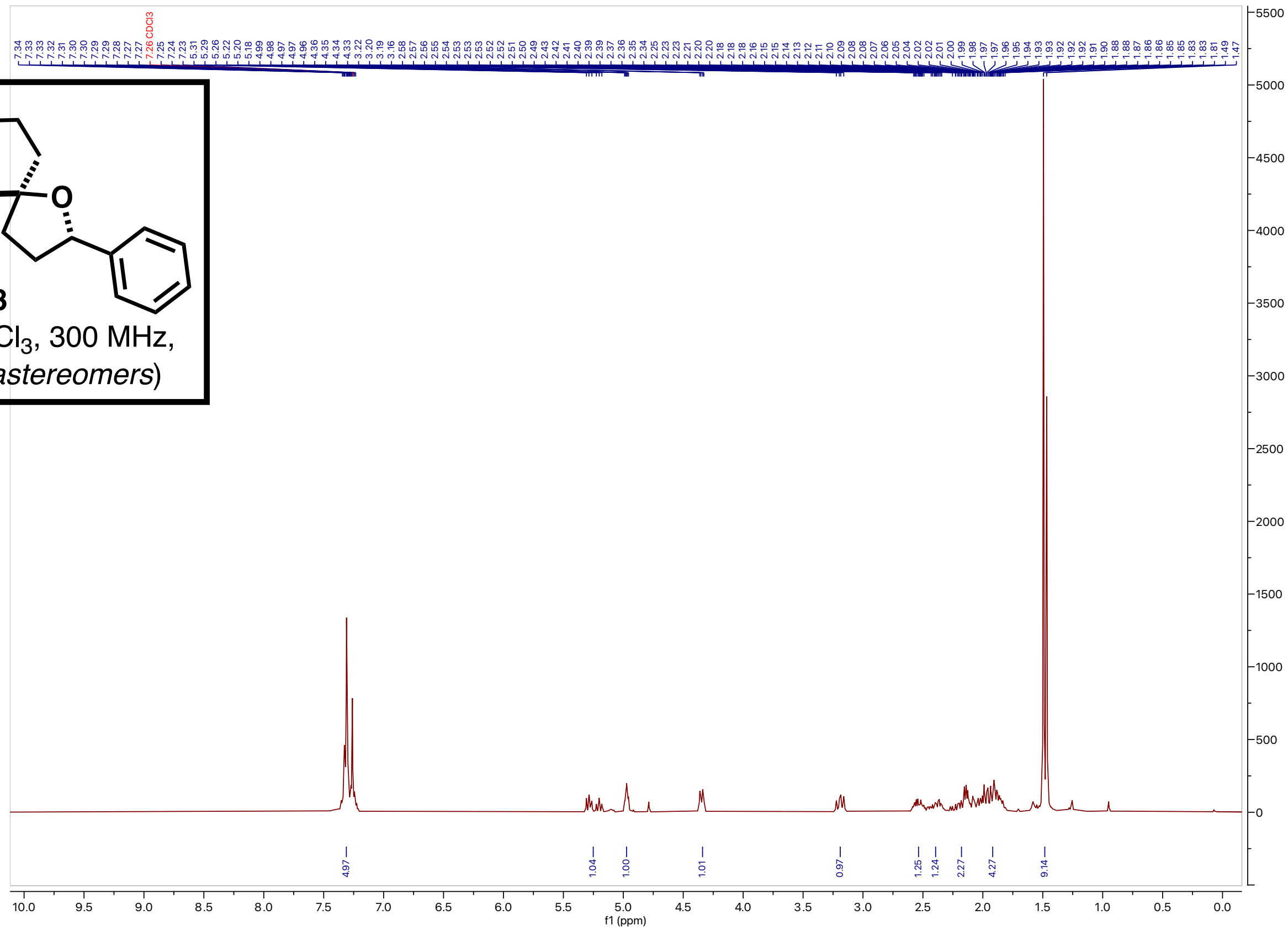

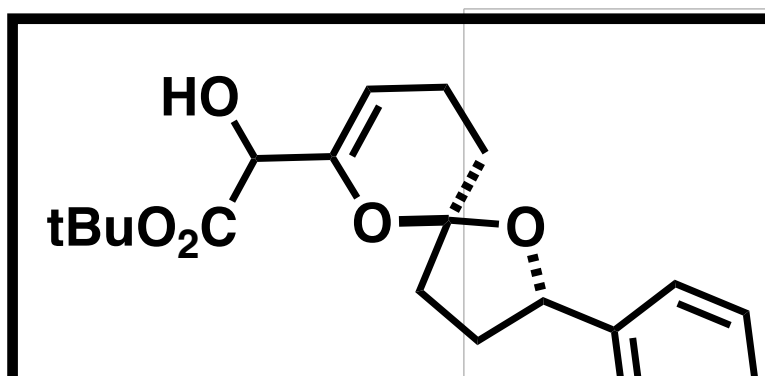

13
(^{1}H NMR, $CDCl_3$, 300 MHz, *mixture of diastereomers*)

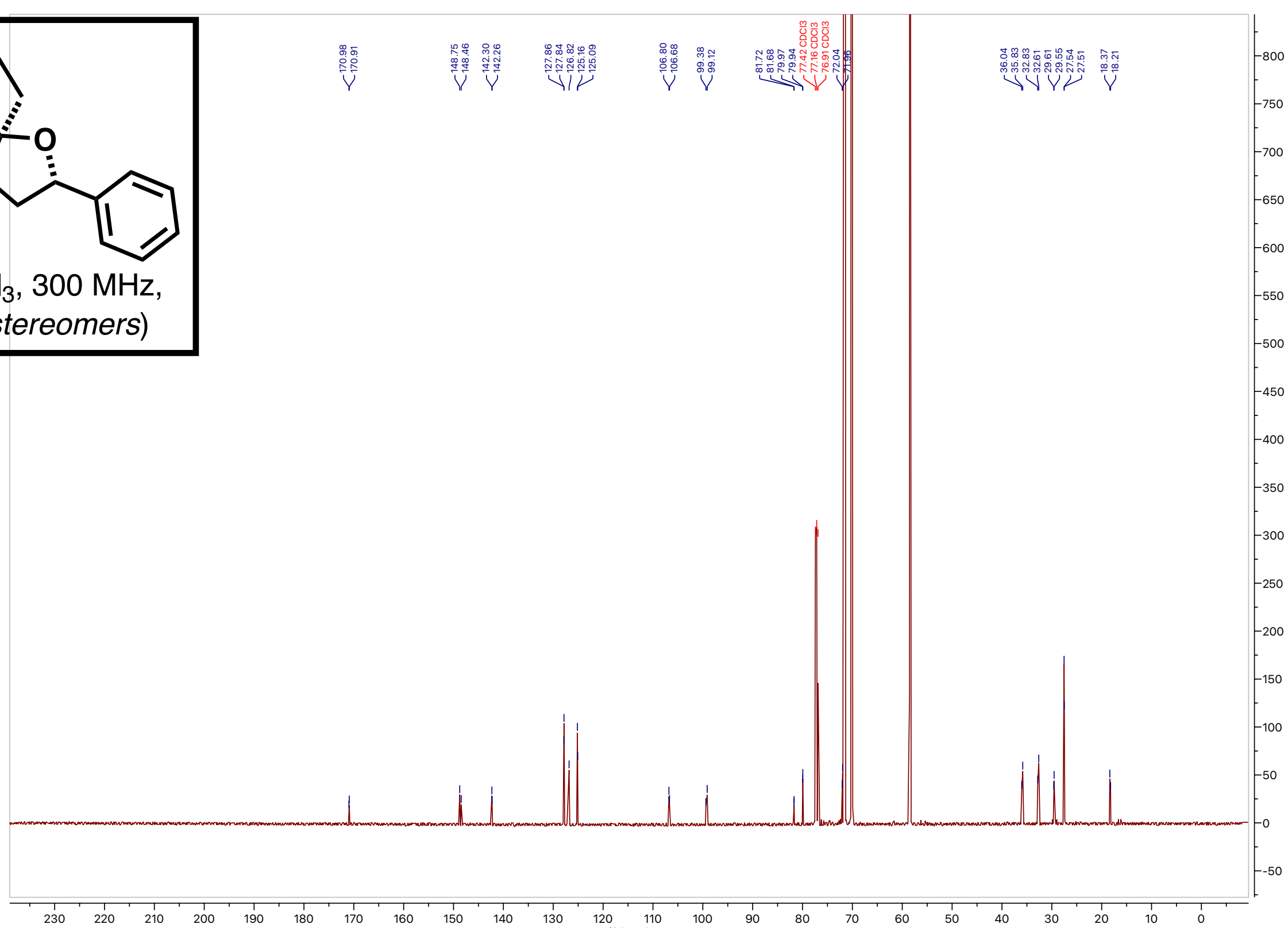

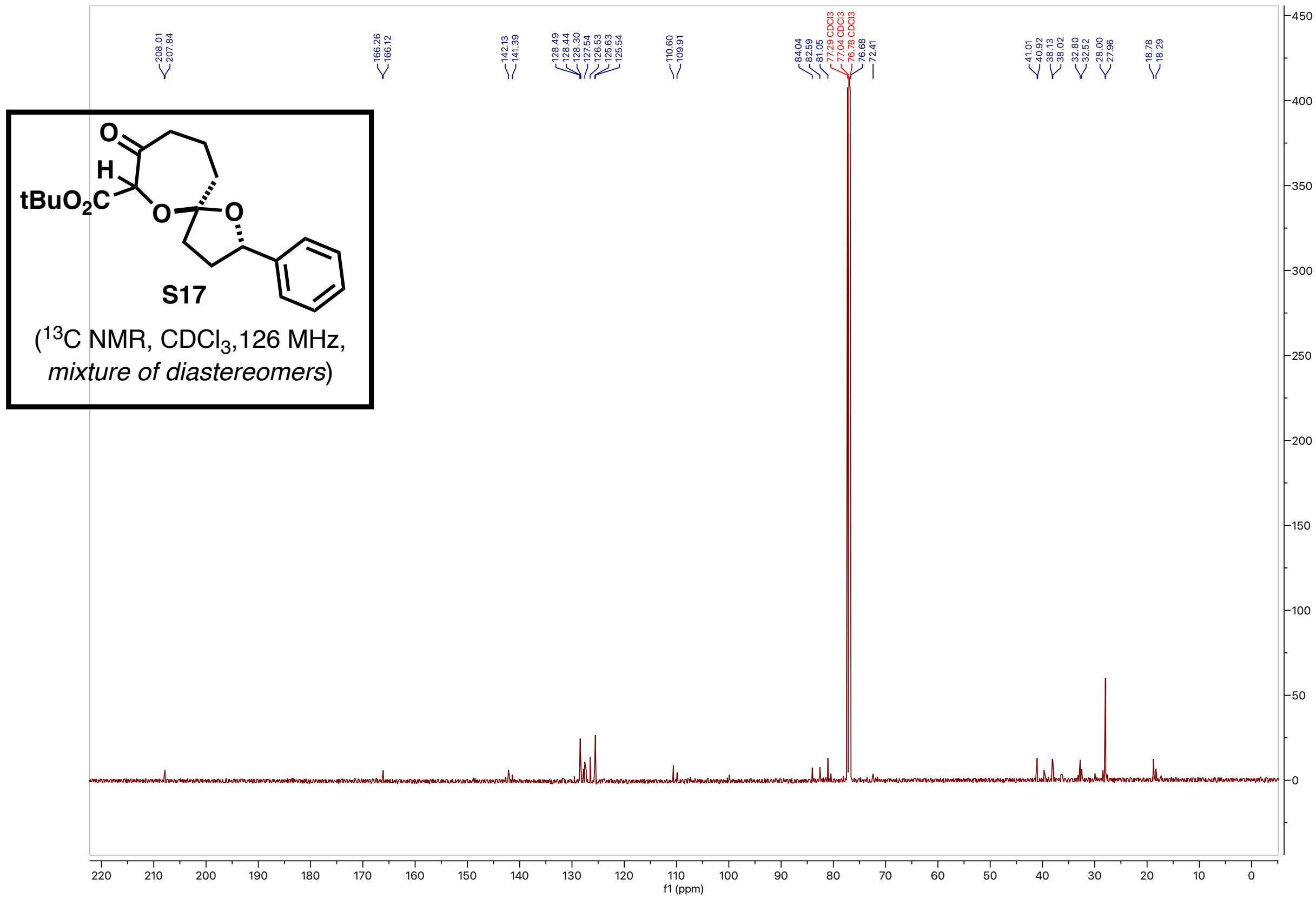
tBuO2C
S17
(13C NMR, CDCl3,126 MHz,
mixture of diastereomers)
f1 (ppm)

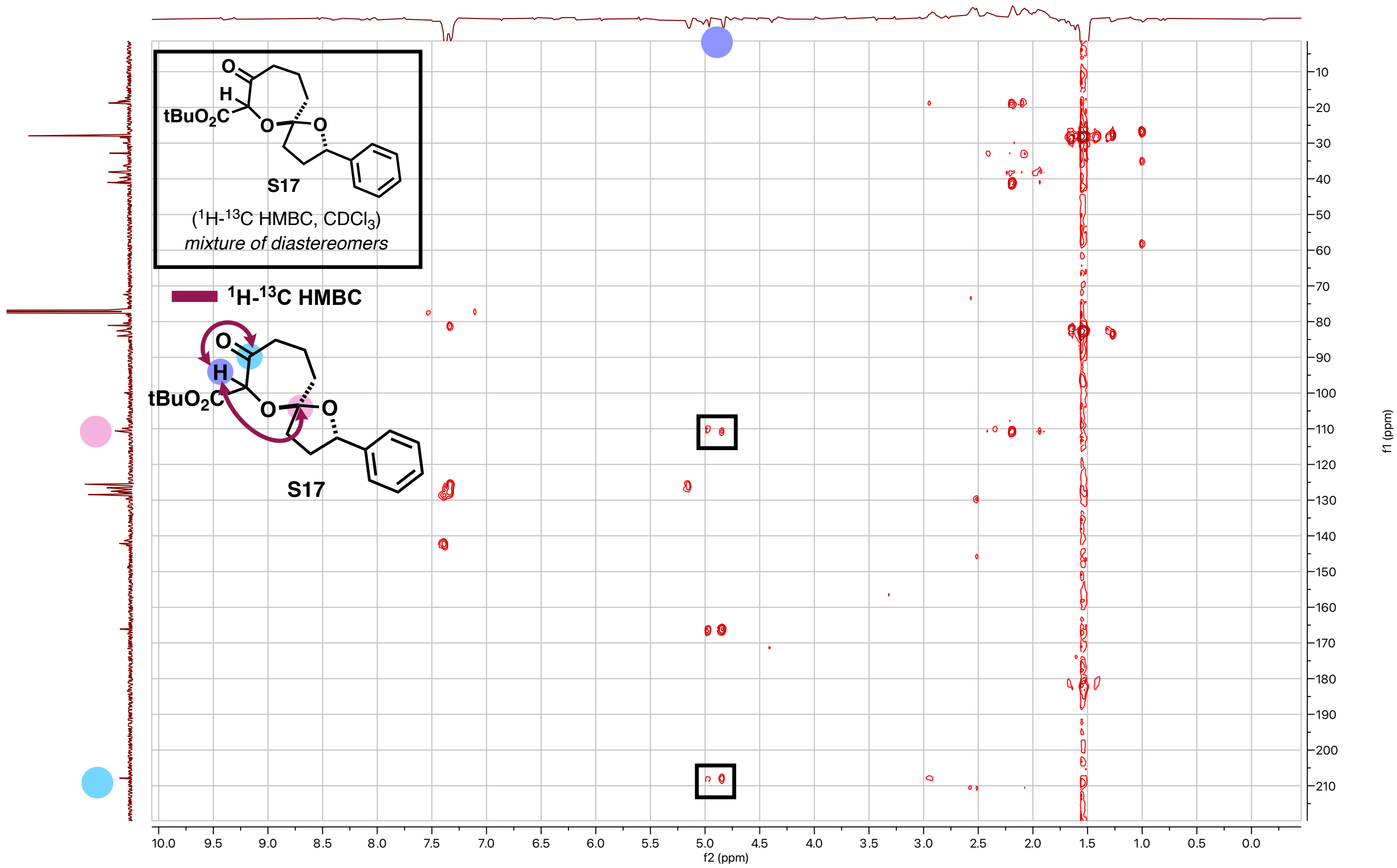
tBuO2C
S17
(1H-13C HMBC, CDCl3)
mixture of diastereomers
1H-13C HMBC
f2 (ppm)
f1 (ppm)

S19

(^{1}H NMR, $CDCl_3$, 600 MHz)
crude aliquot in THF

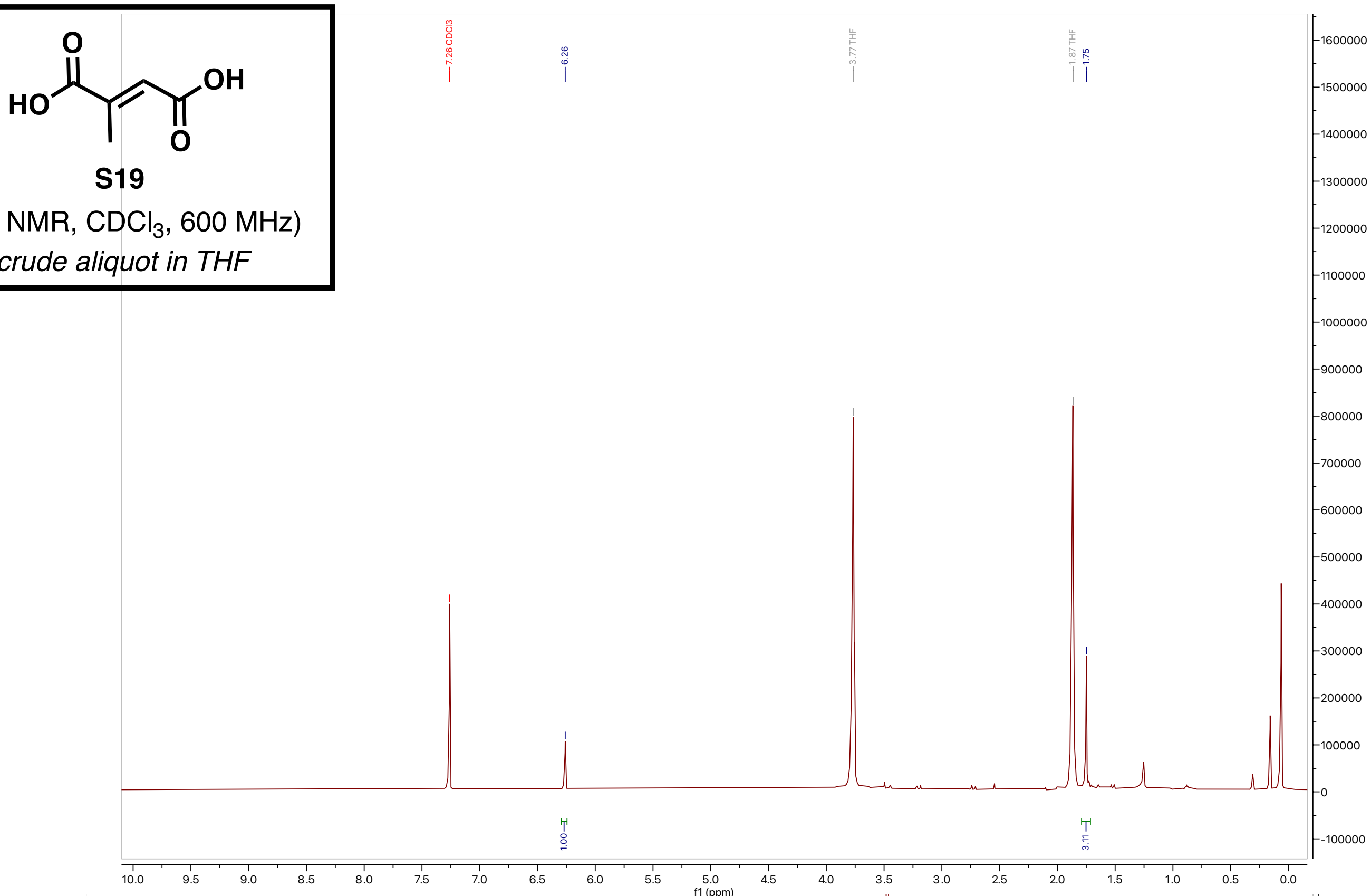

S19

(^{13}C NMR, $CDCl_3$, 126 MHz)
crude aliquot in THF

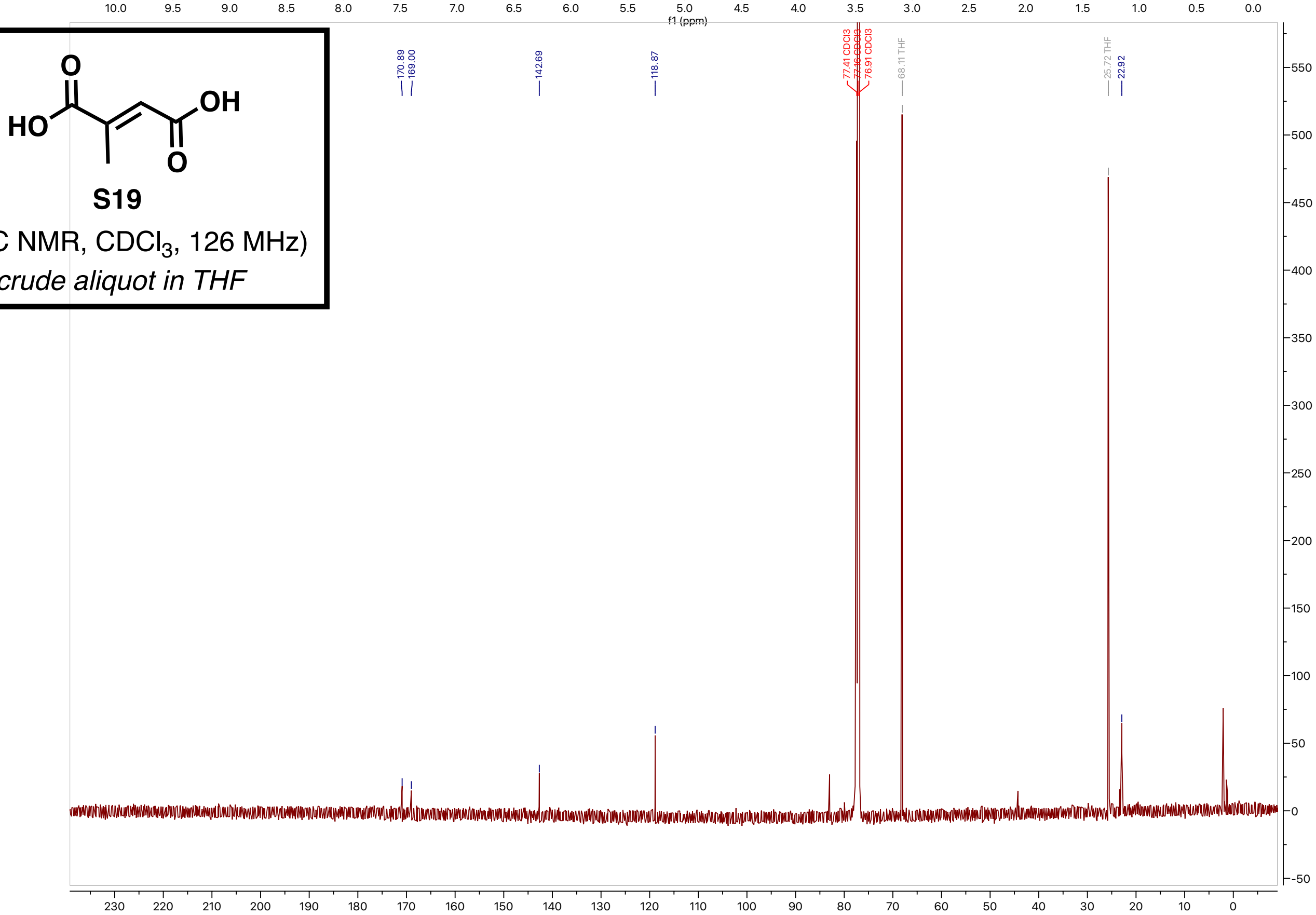

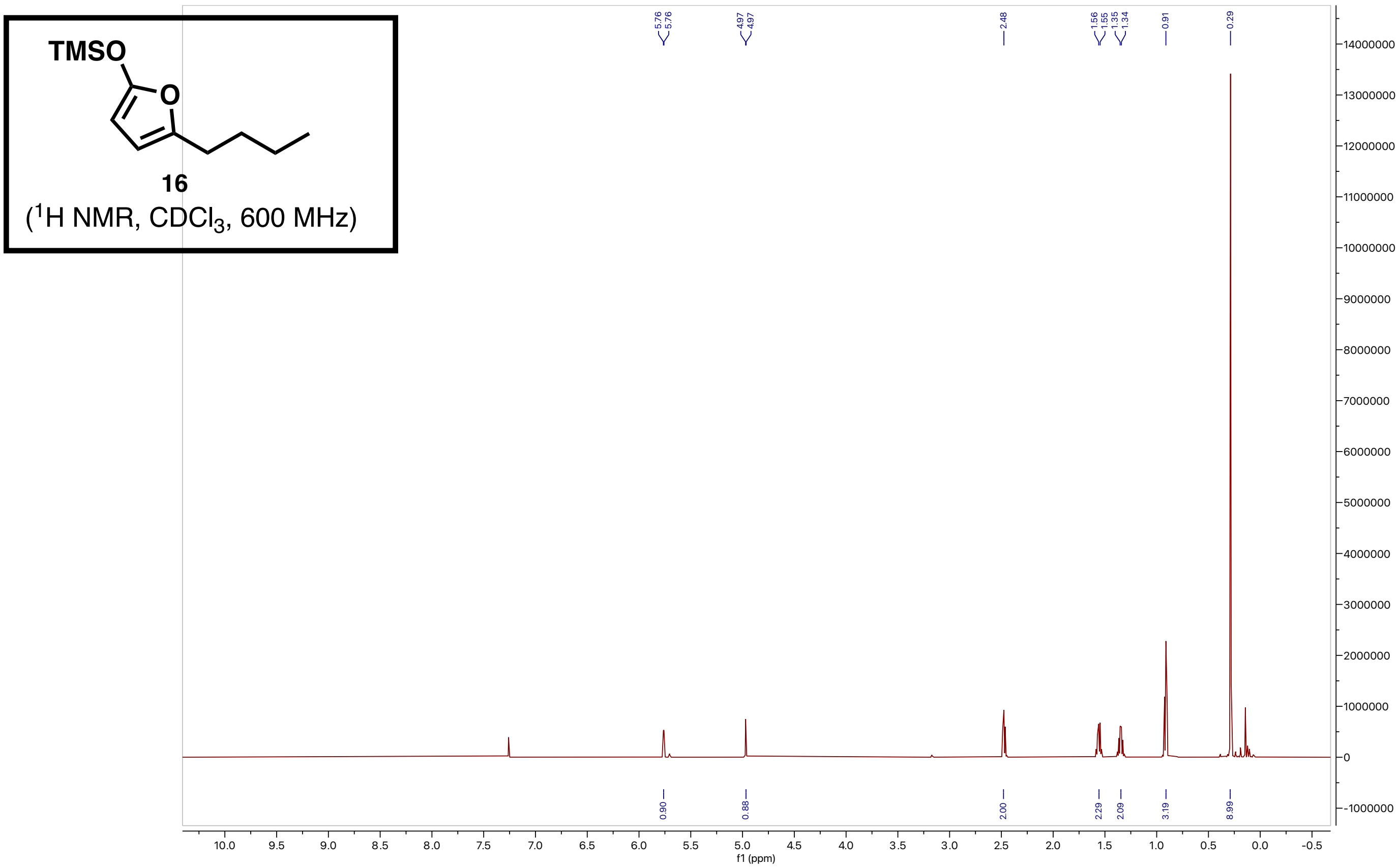

16
(^{1}H NMR, $CDCl_3$, 600 MHz)

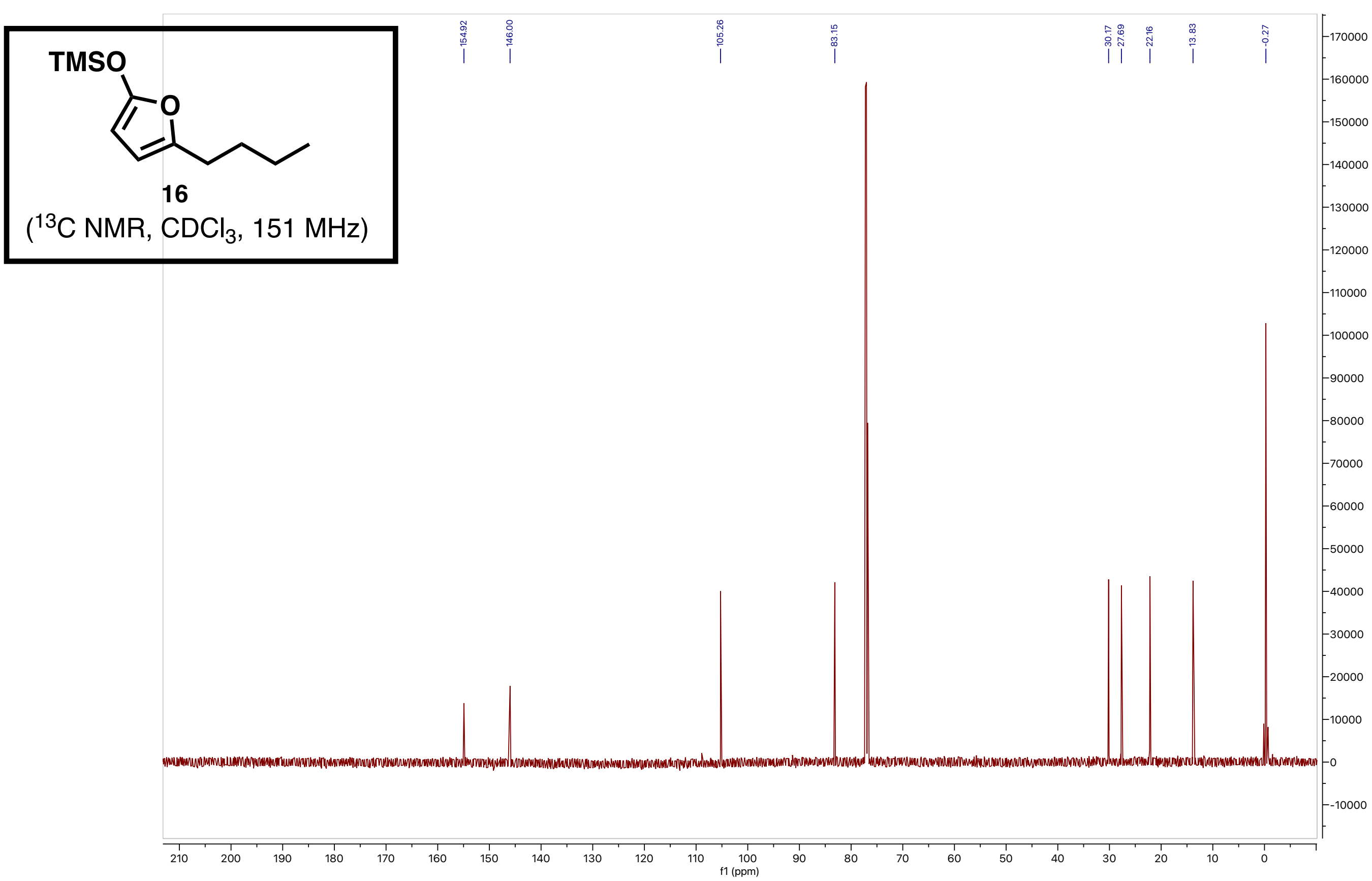

16
(^{13}C NMR, $CDCl_3$, 151 MHz)

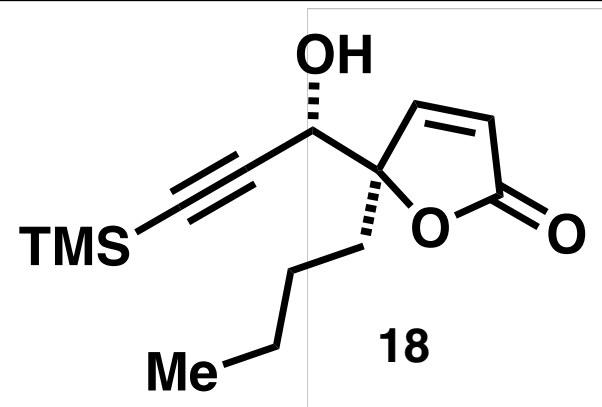

18

(^{1}H NMR, $CDCl_3$, 500 MHz)

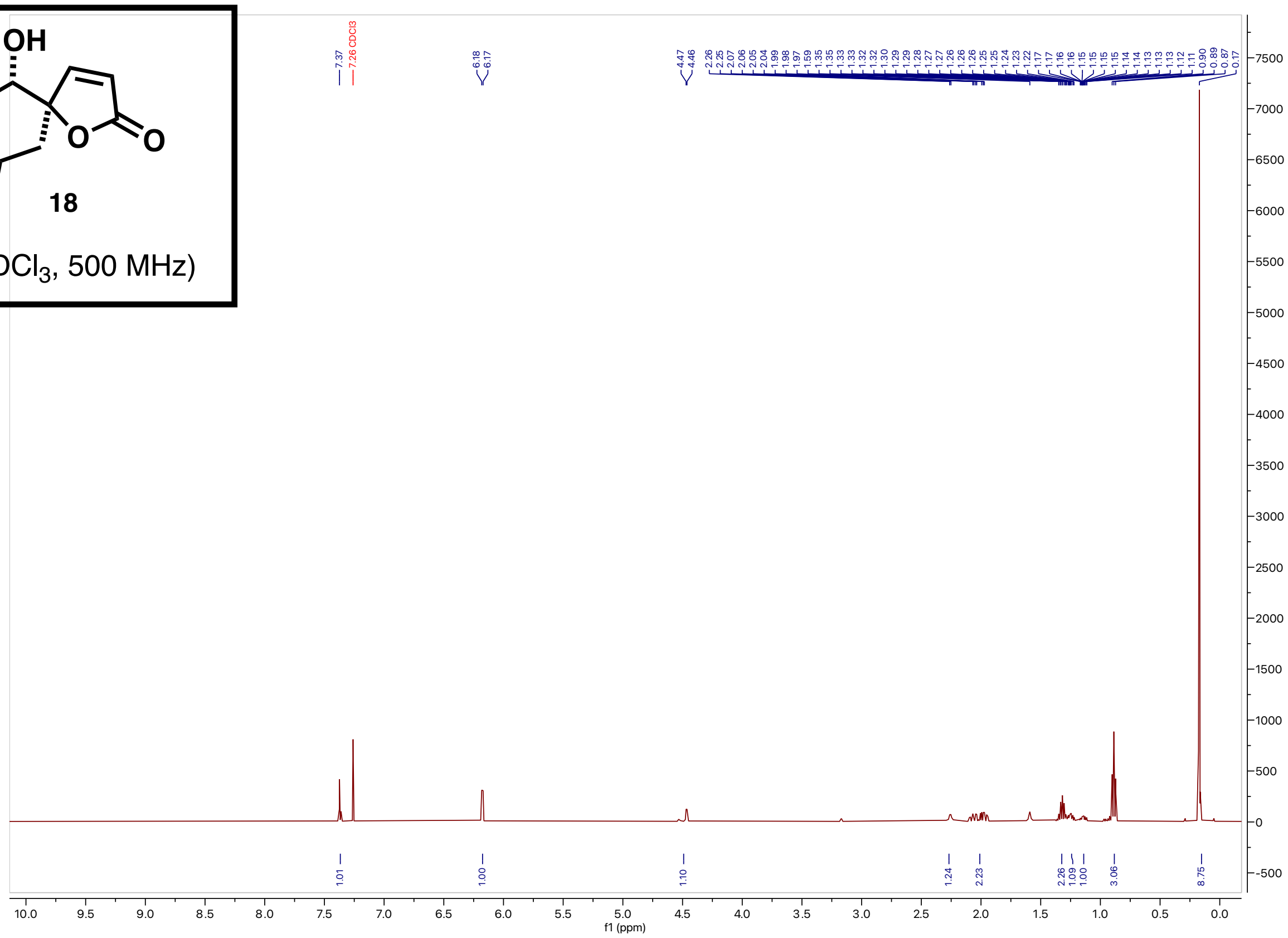

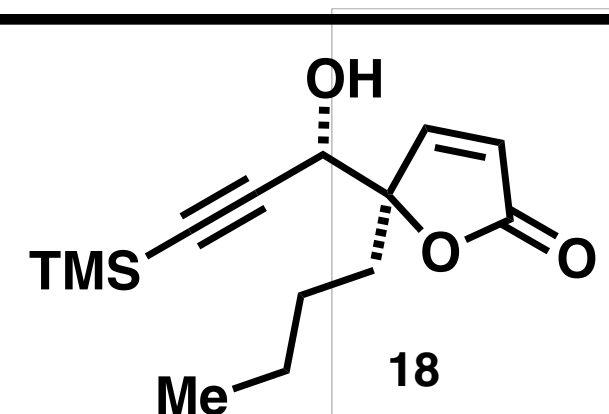

18

(^{13}C NMR, $CDCl_3$, 126 MHz)

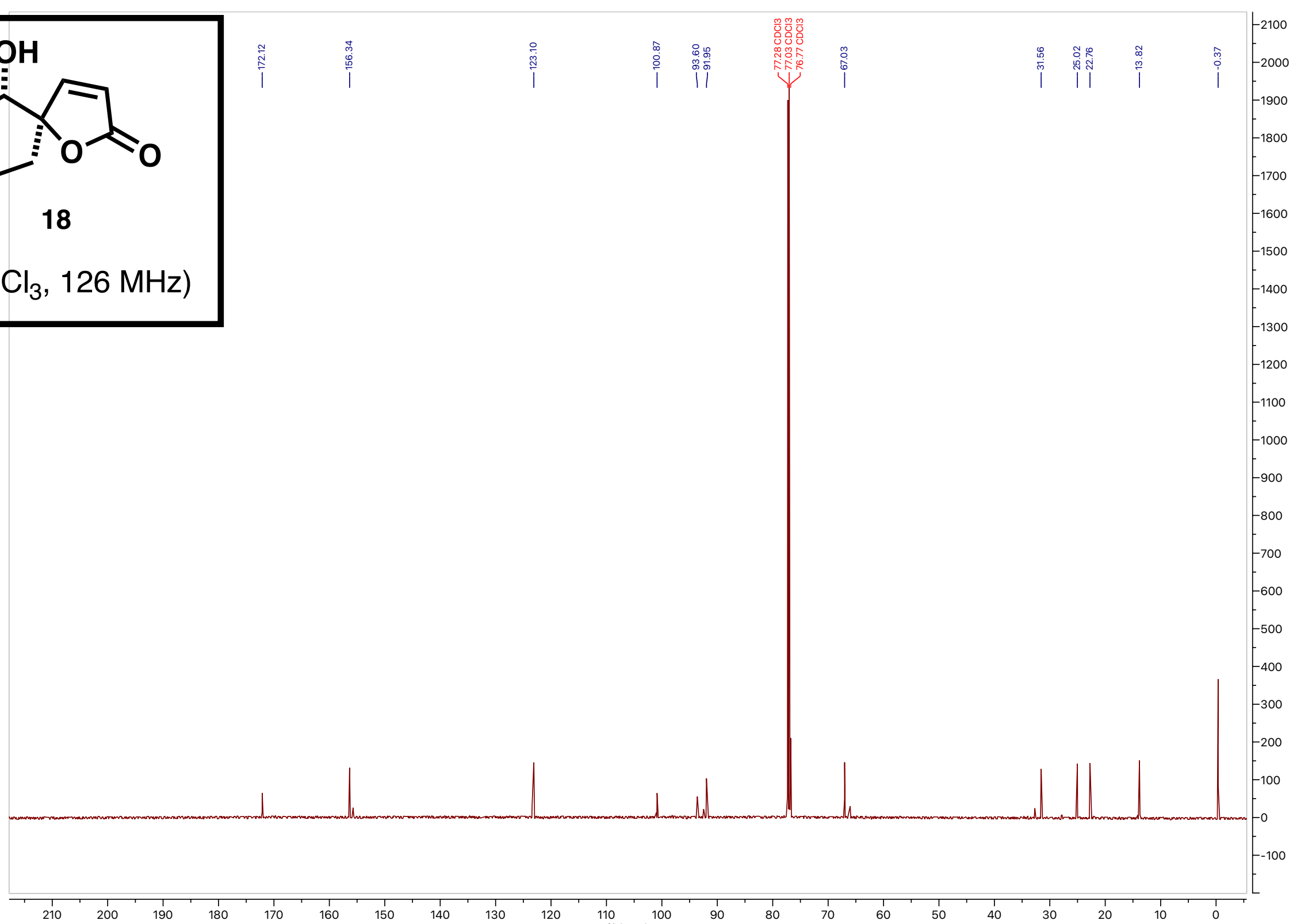

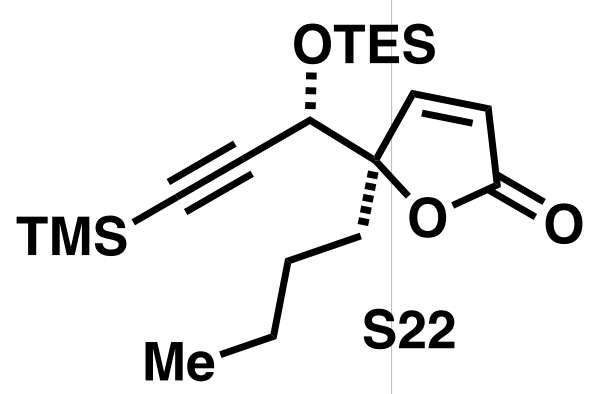

S22

(^{1}H NMR, $CDCl_3$, 500 MHz)

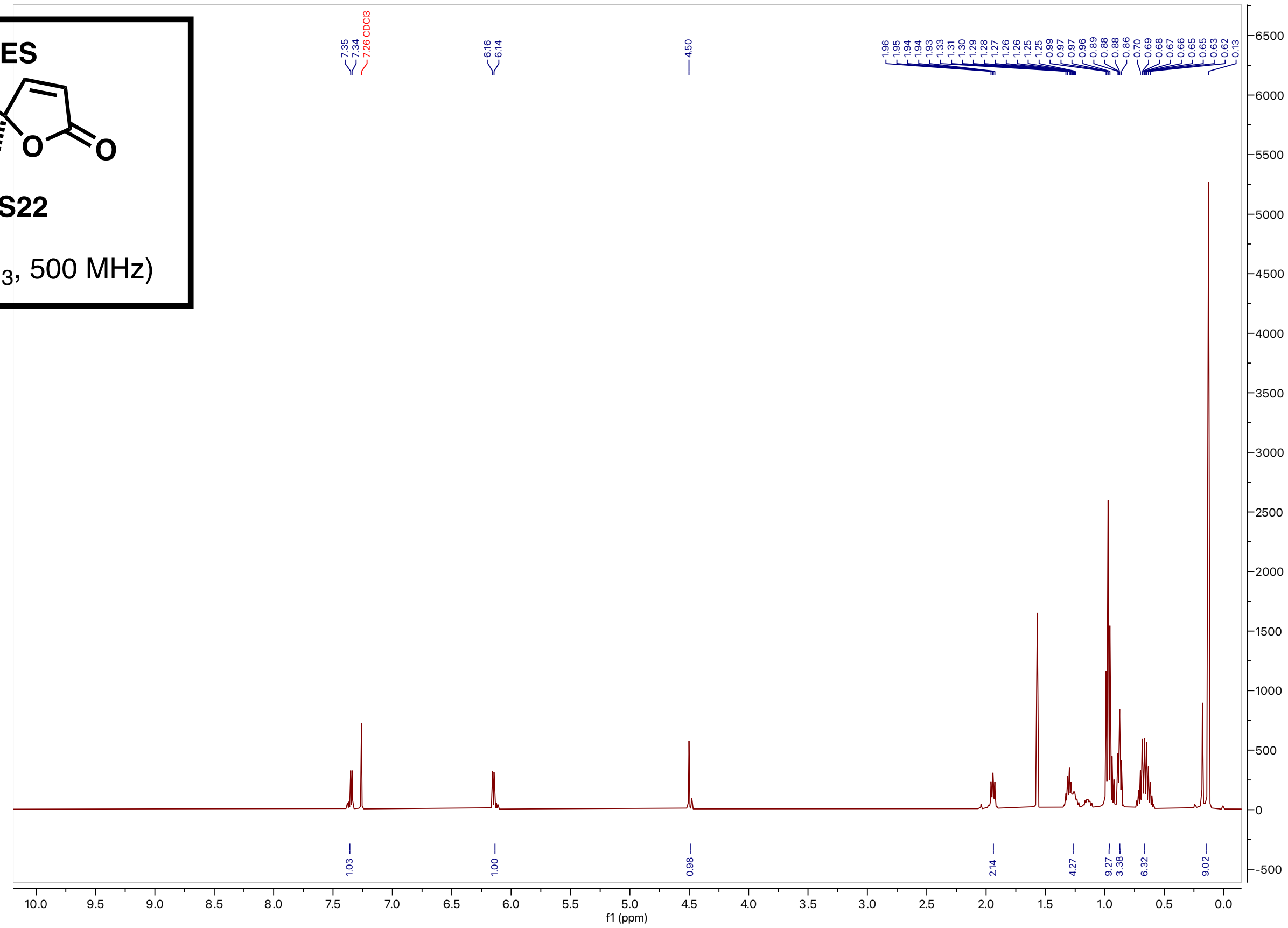

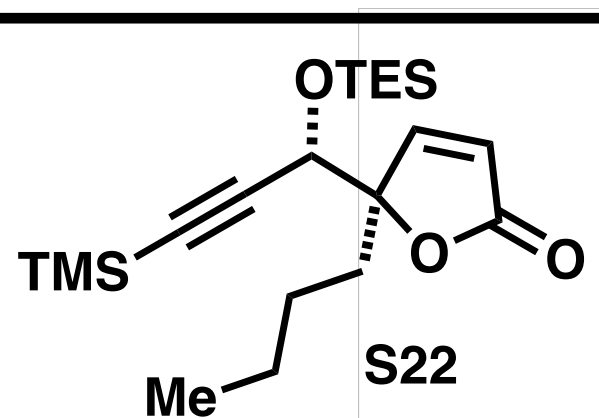

S22

(^{13}C NMR, $CDCl_3$, 126 MHz)

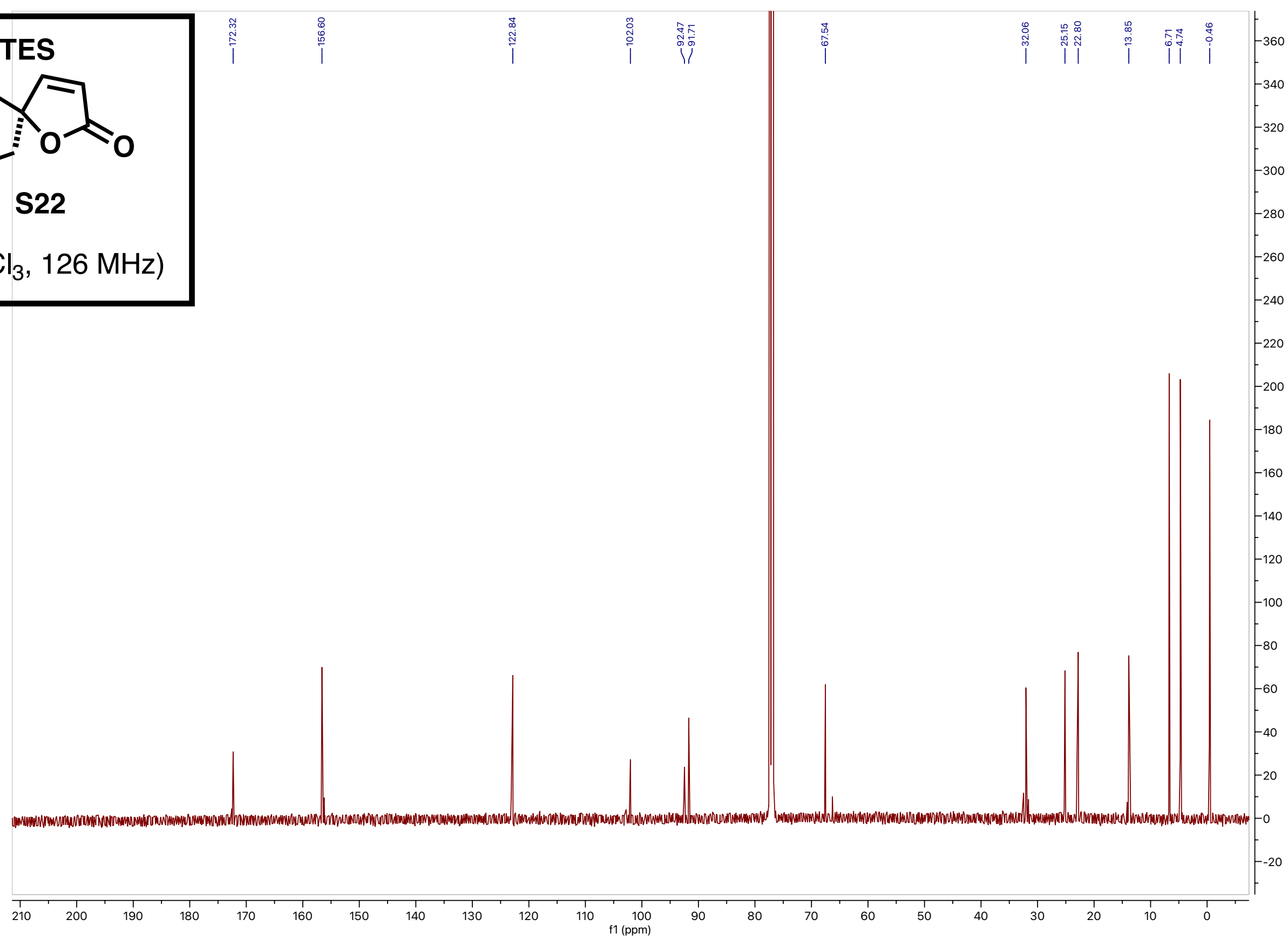

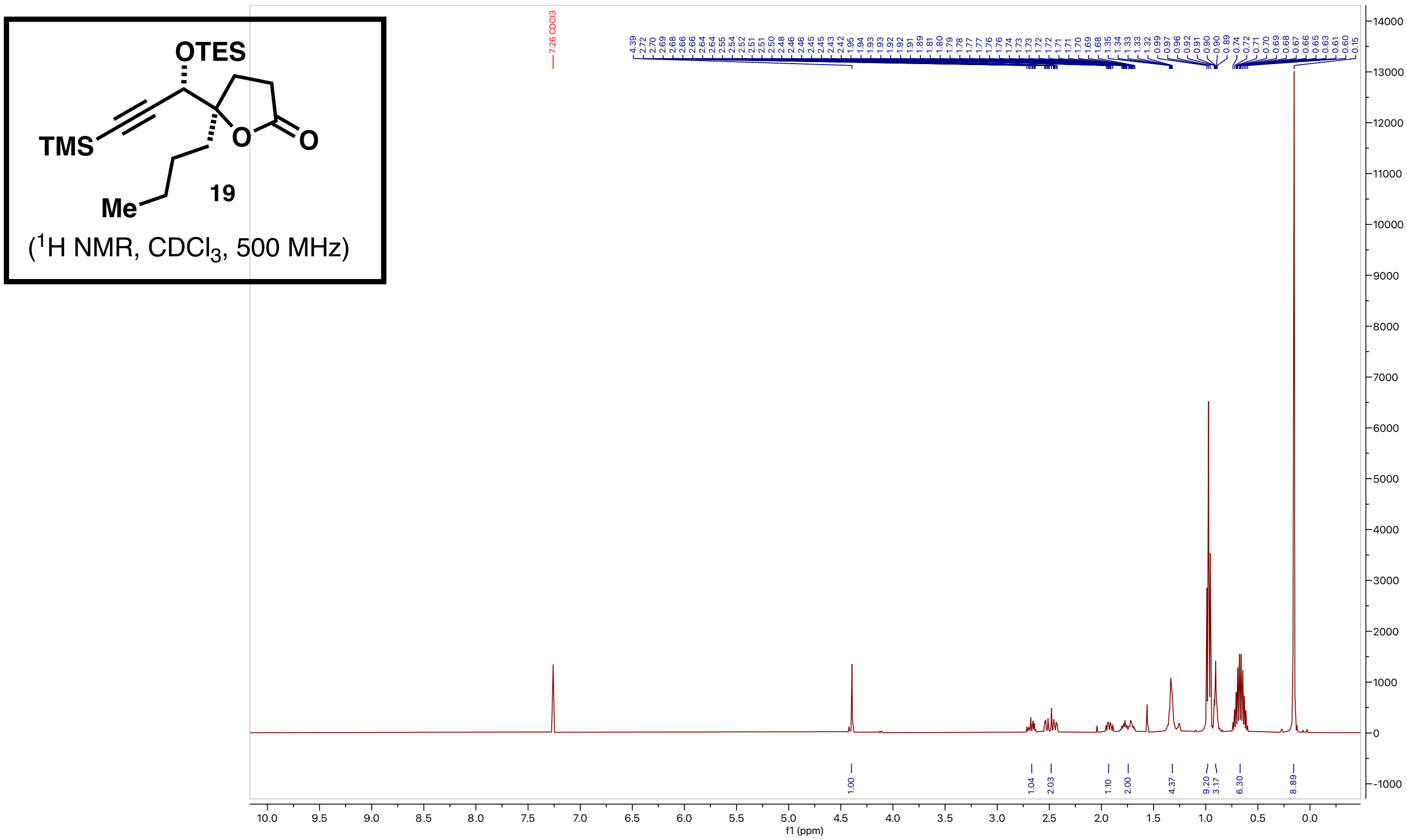
OTES
TMS
O
O
Me
19
(¹H NMR, CDCl₃, 500 MHz)
f1 (ppm)

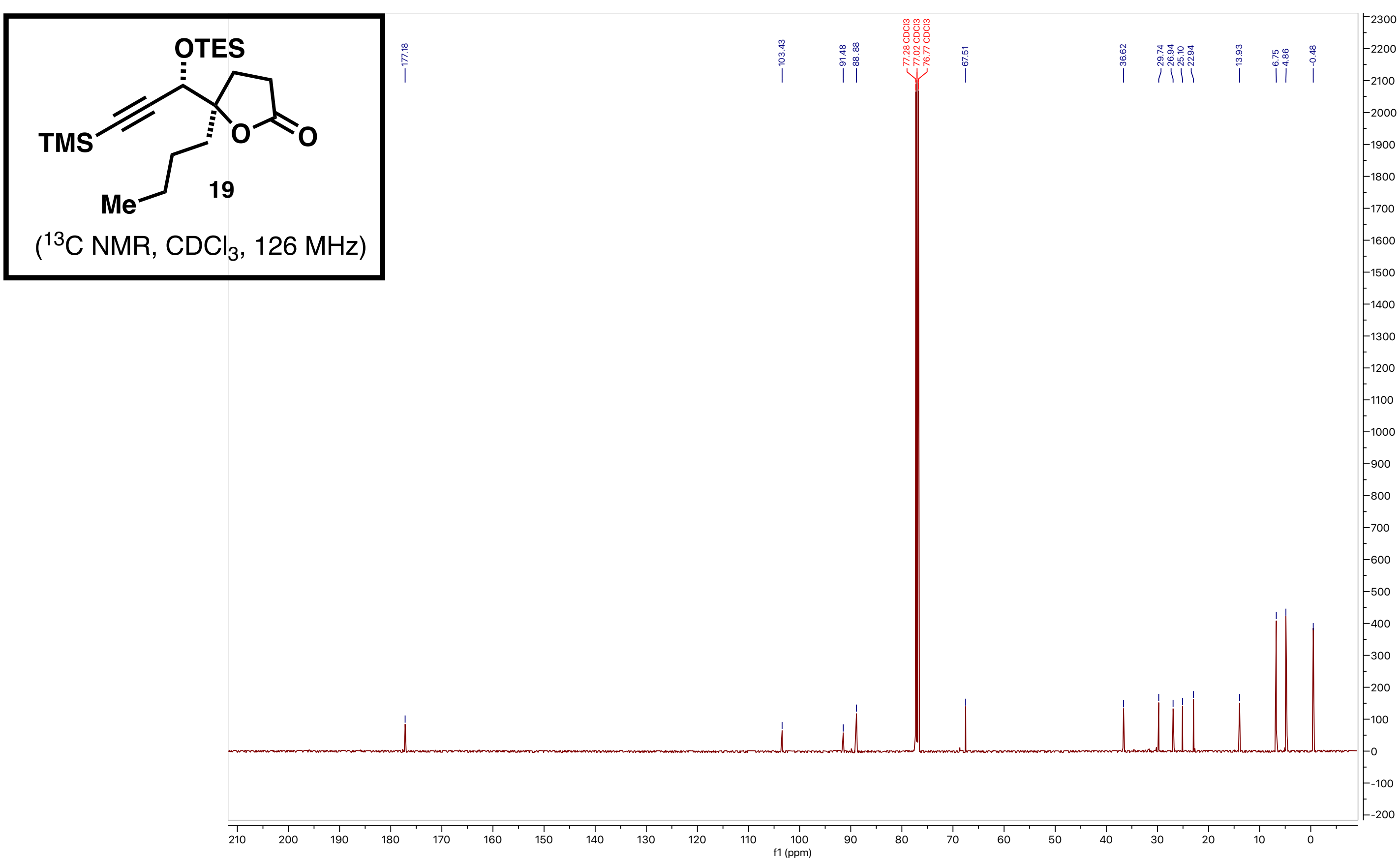
OTES
TMS
O
O
Me
19
(¹³C NMR, CDCl₃, 126 MHz)
f1 (ppm)

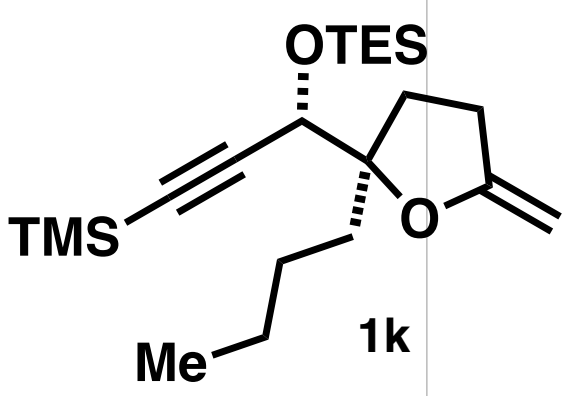

1k

(^{1}H NMR, $CDCl_3$, 500 MHz, crude, not isolated)

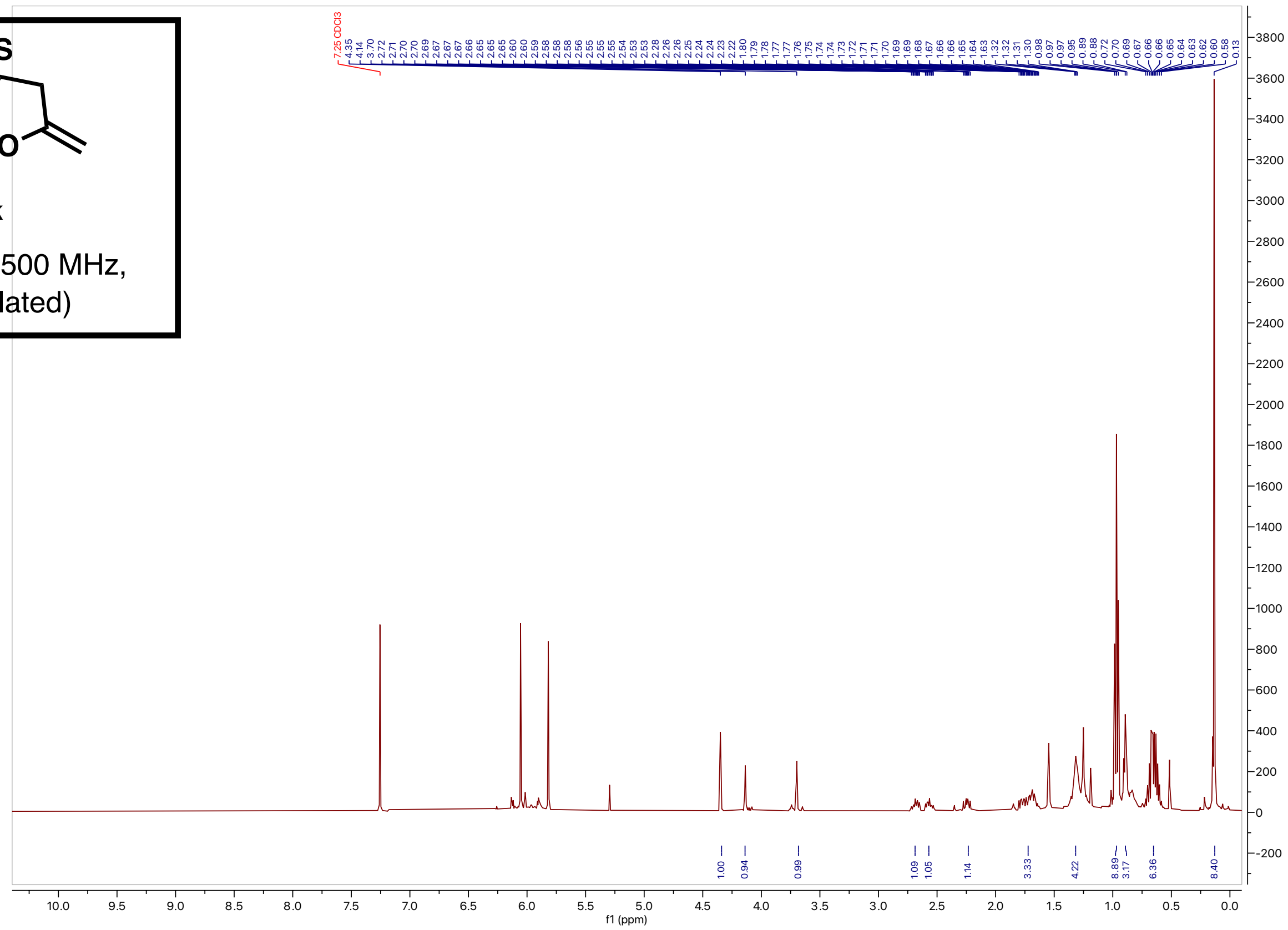

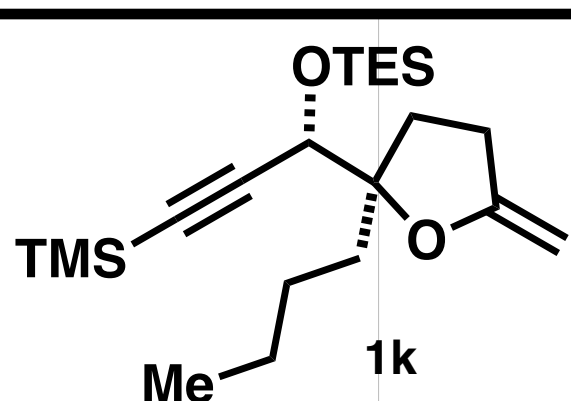

1k

(^{13}C NMR, $CDCl_3$, 151 MHz, crude, not isolated)

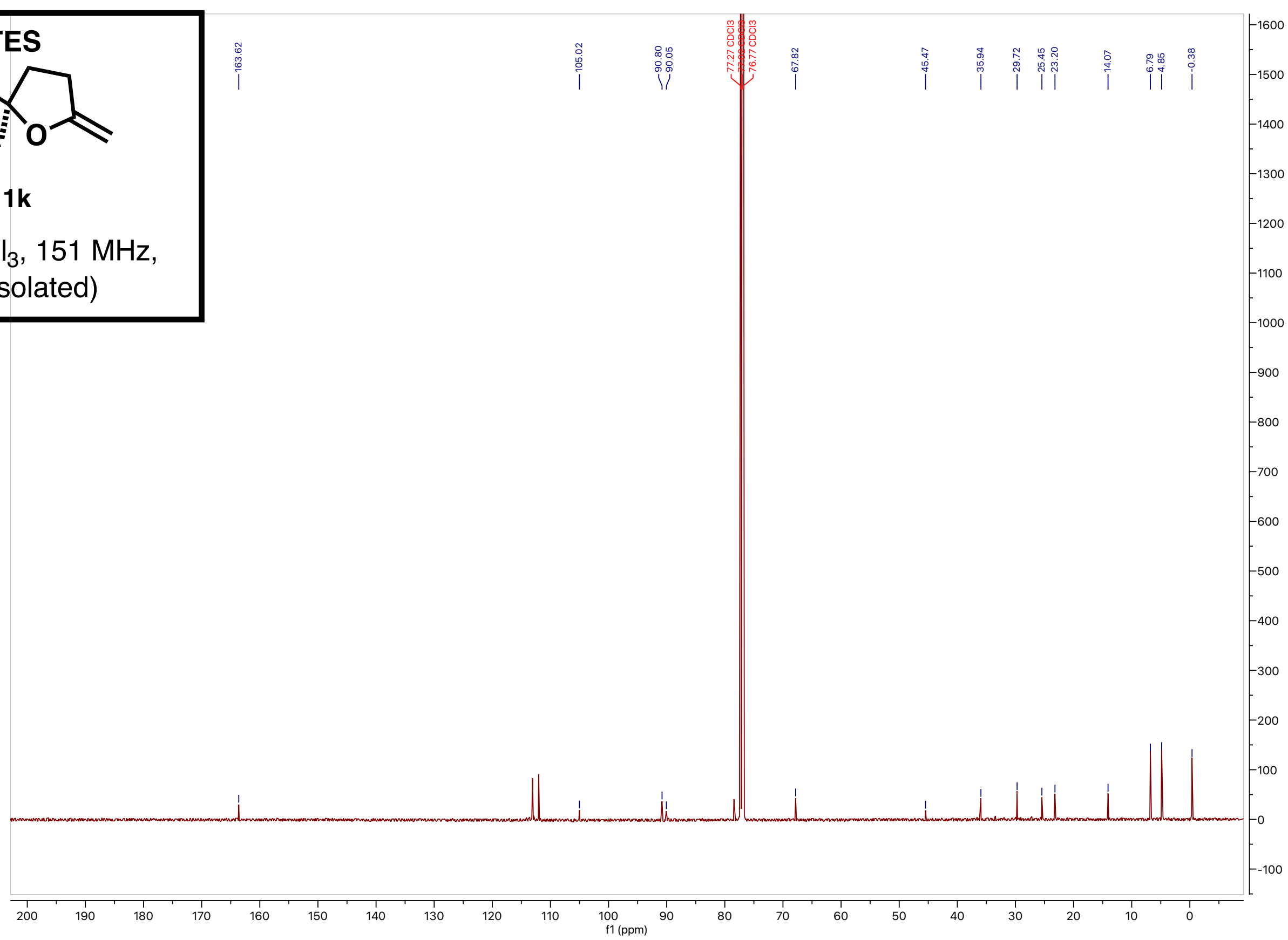

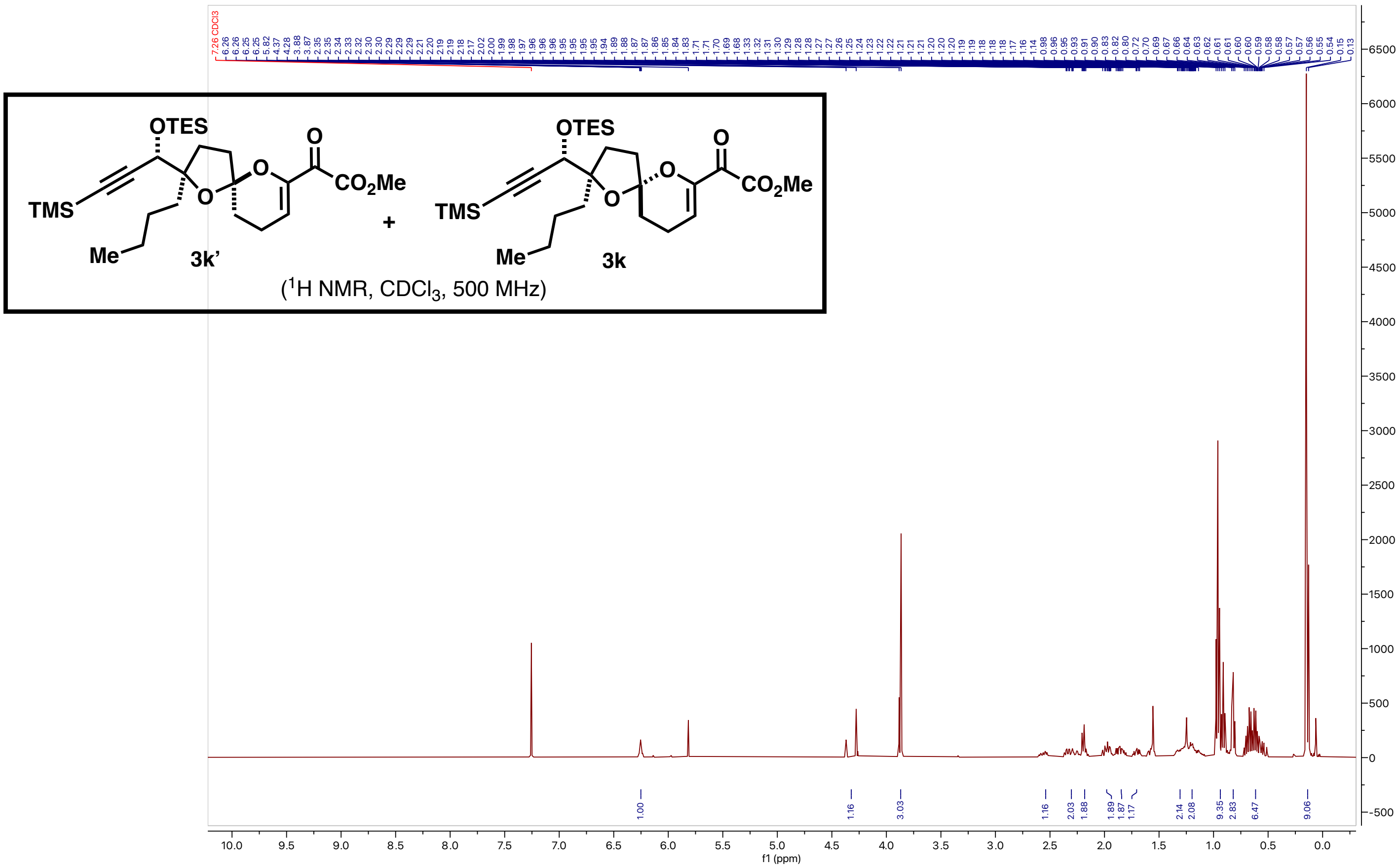
OTES
O
CO2Me
TMS
Me
3k'
+
3k
(1H NMR, CDCl3, 500 MHz)
f1 (ppm)

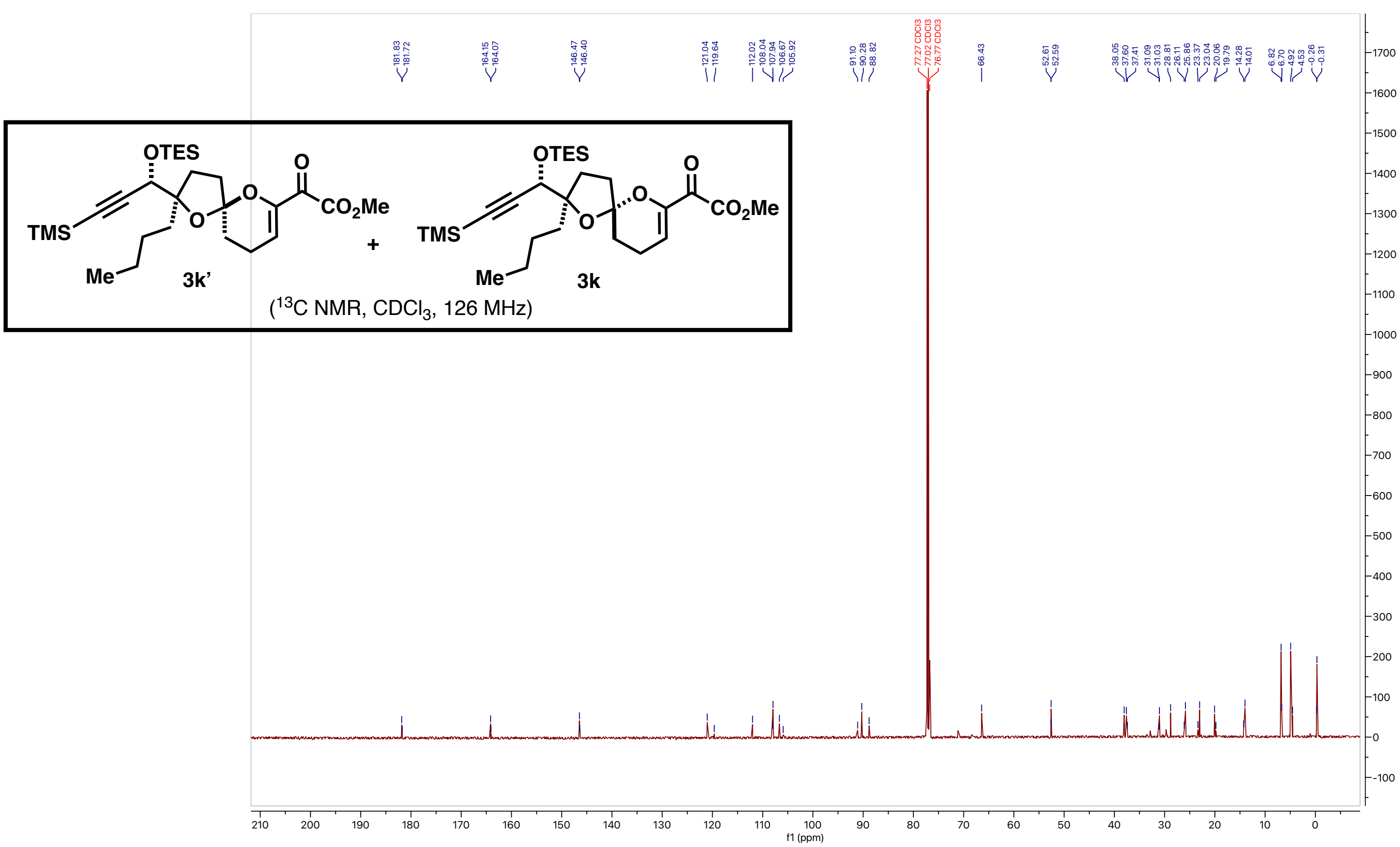
OTES
O
CO2Me
TMS
Me
3k'
+
3k
(13C NMR, CDCl3, 126 MHz)
f1 (ppm)

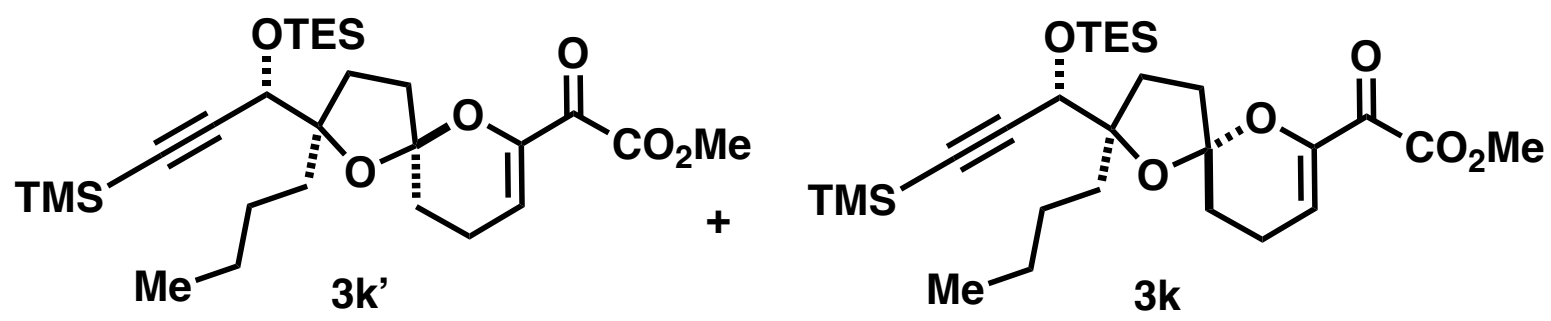

(^{1}H-^{13}C HSQC, $CDCl_3$)

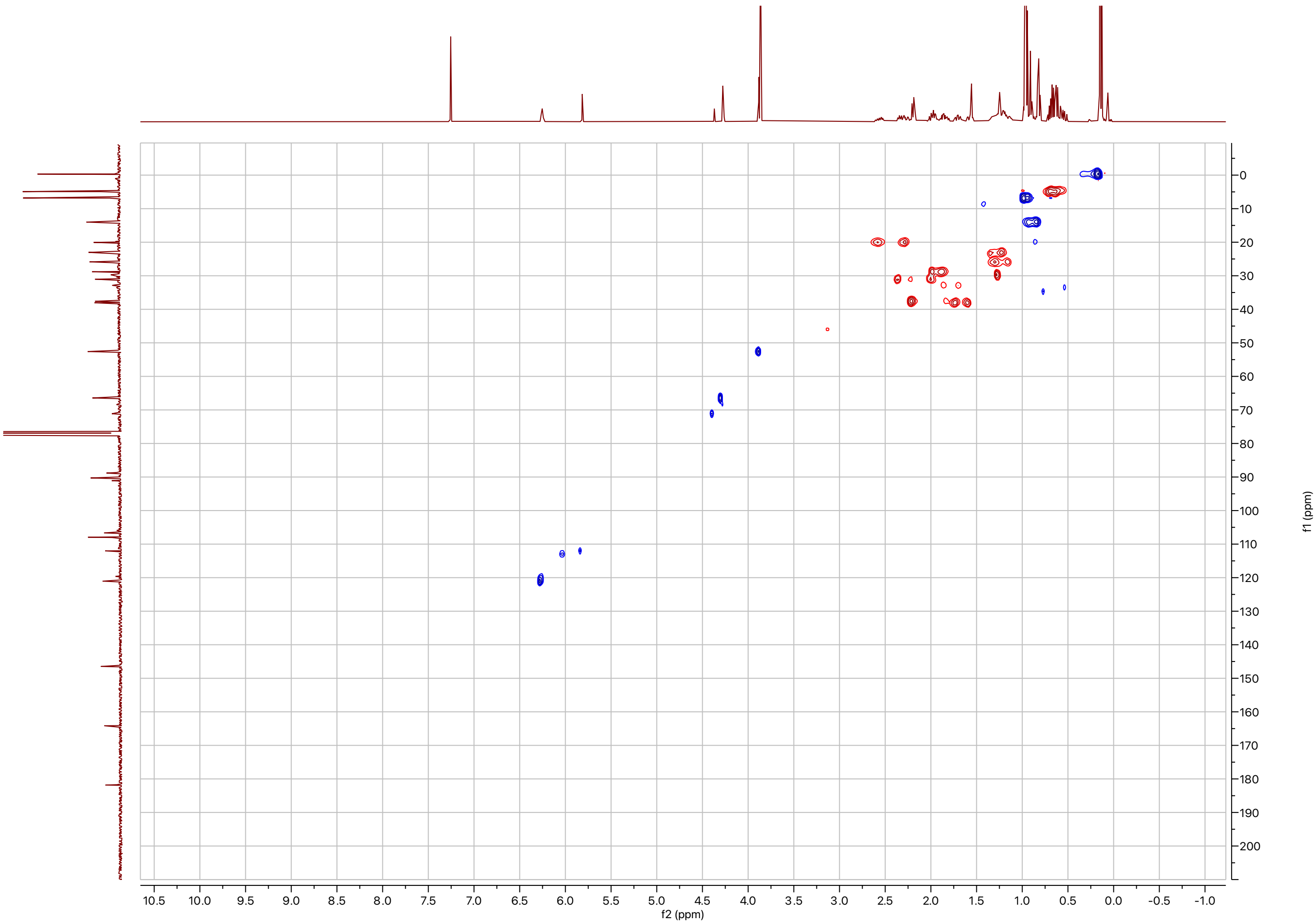

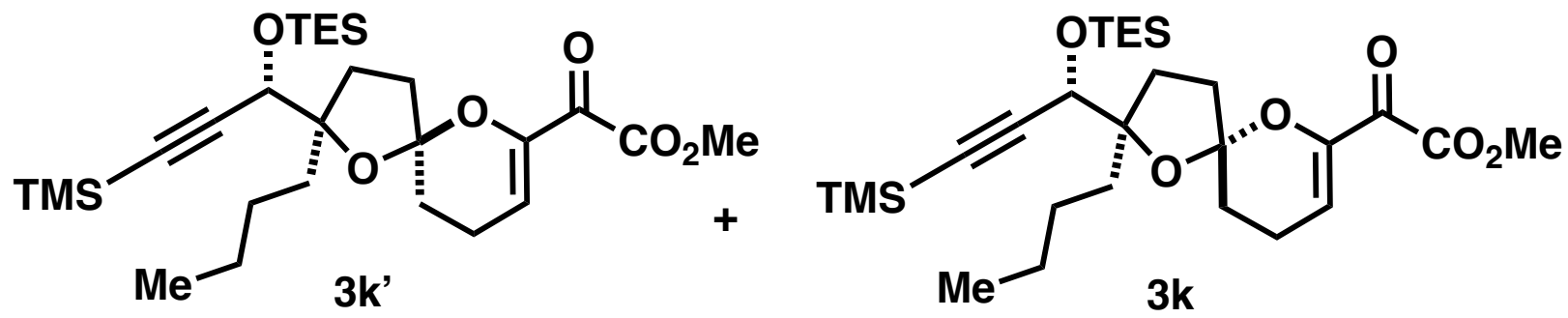

(^{1}H-^{13}C HMBC, $CDCl_3$)

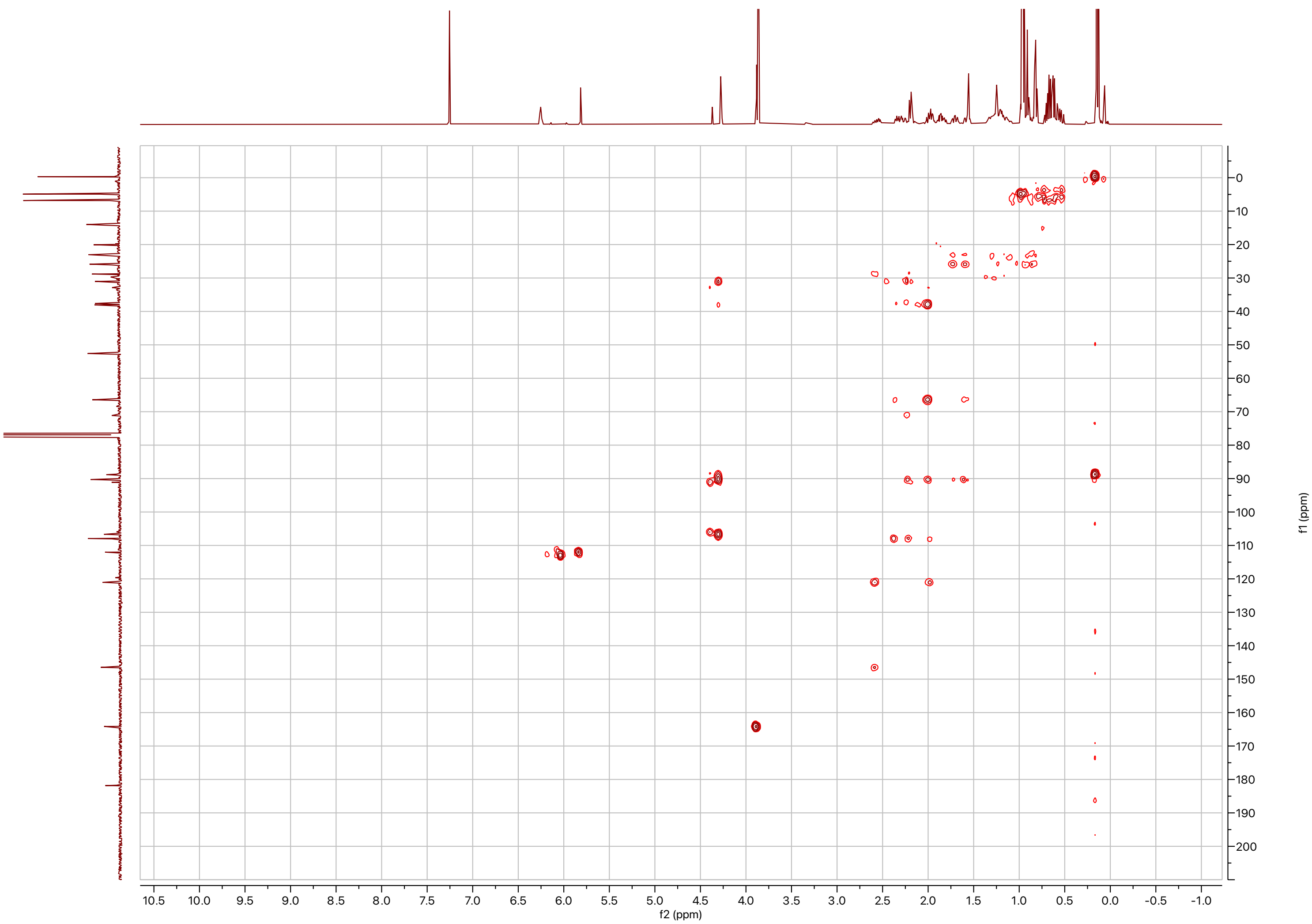

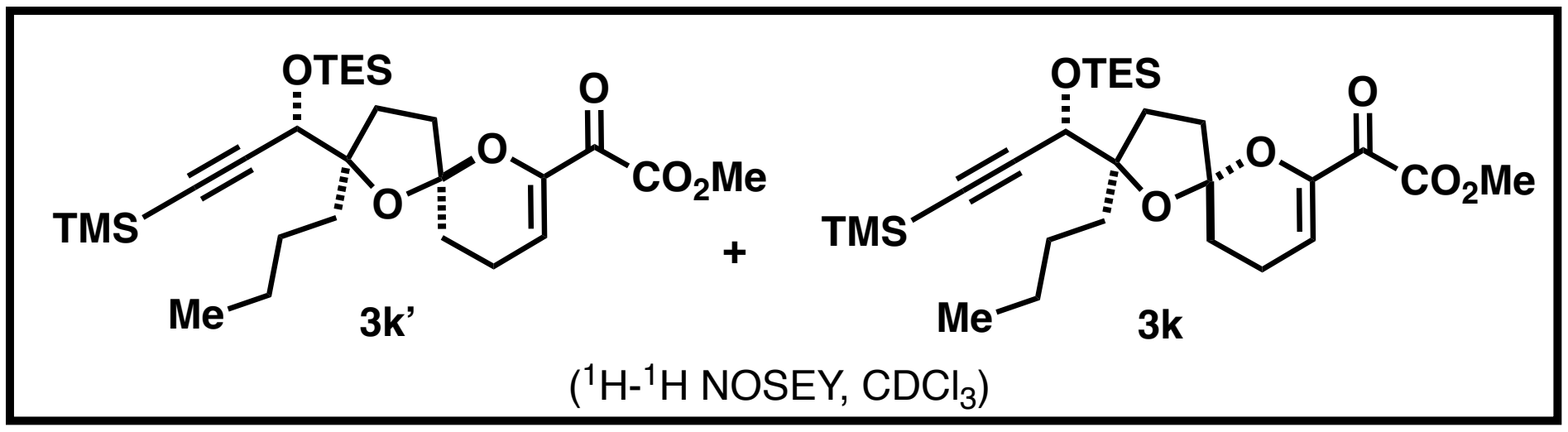
OTES
O
CO2Me
TMS
O
O
Me
3k'
+
OTES
O
CO2Me
TMS
O
O
Me
3k
(1H-1H NOSEY, CDCl3)

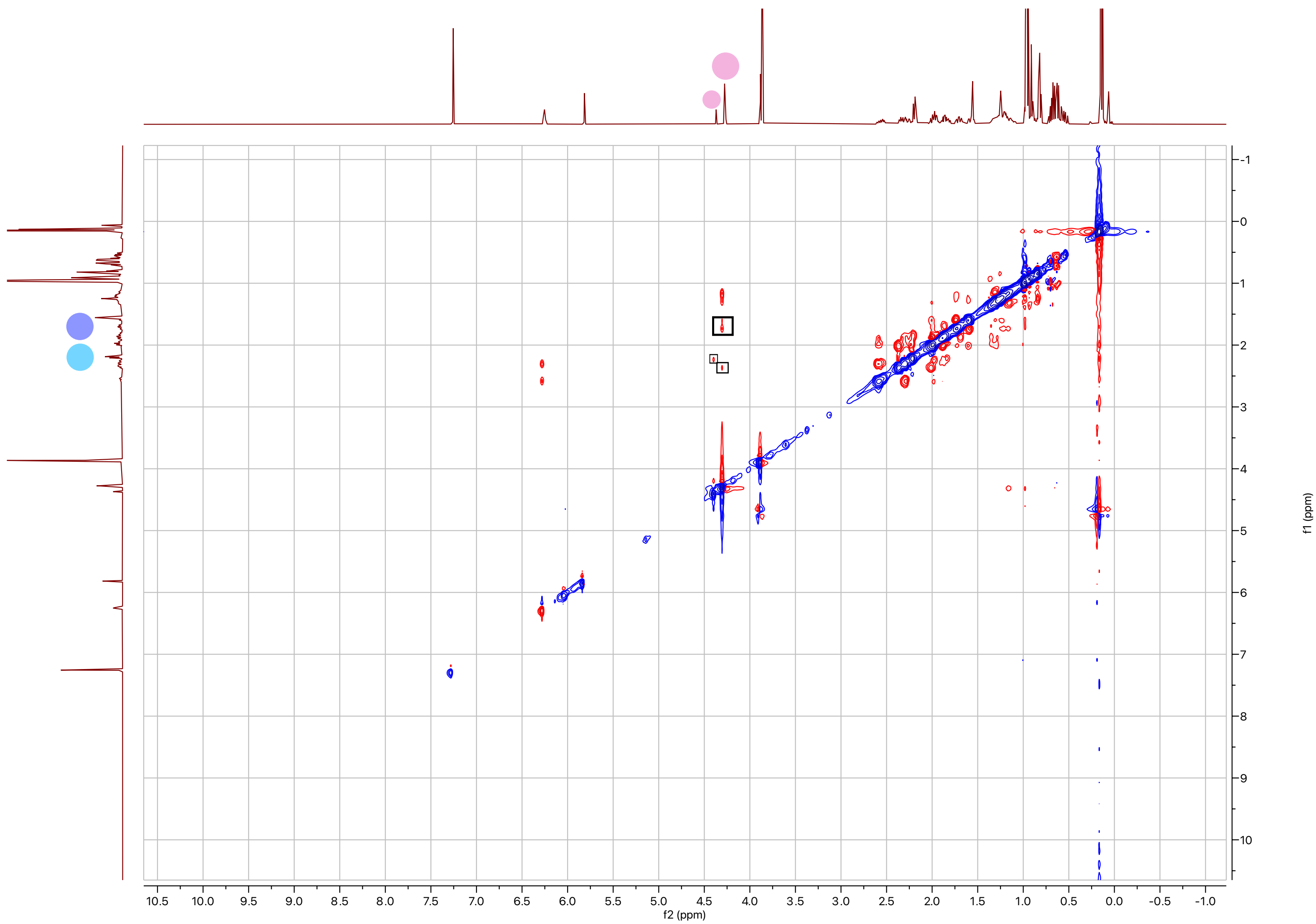
f2 (ppm)
f1 (ppm)

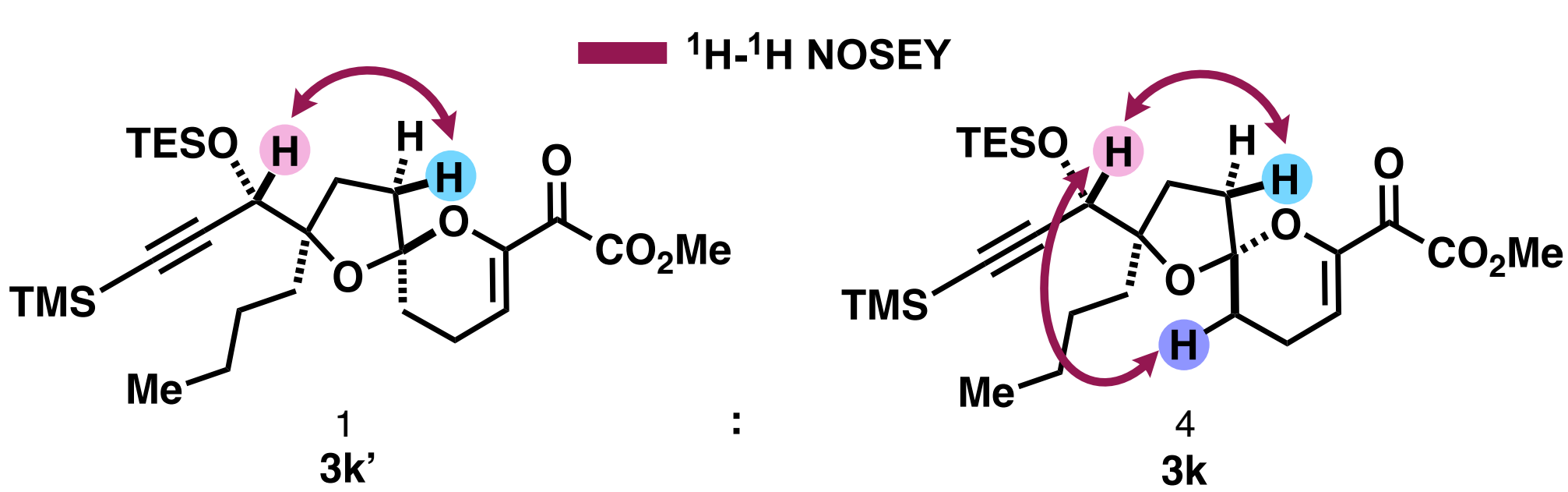
1H-1H NOSEY
TESO
H
H
H
O
CO2Me
TMS
O
O
Me
1
3k'
:
TESO
H
H
H
O
CO2Me
TMS
O
O
H
Me
4
3k

5. Crystallographic data

Structure Tables

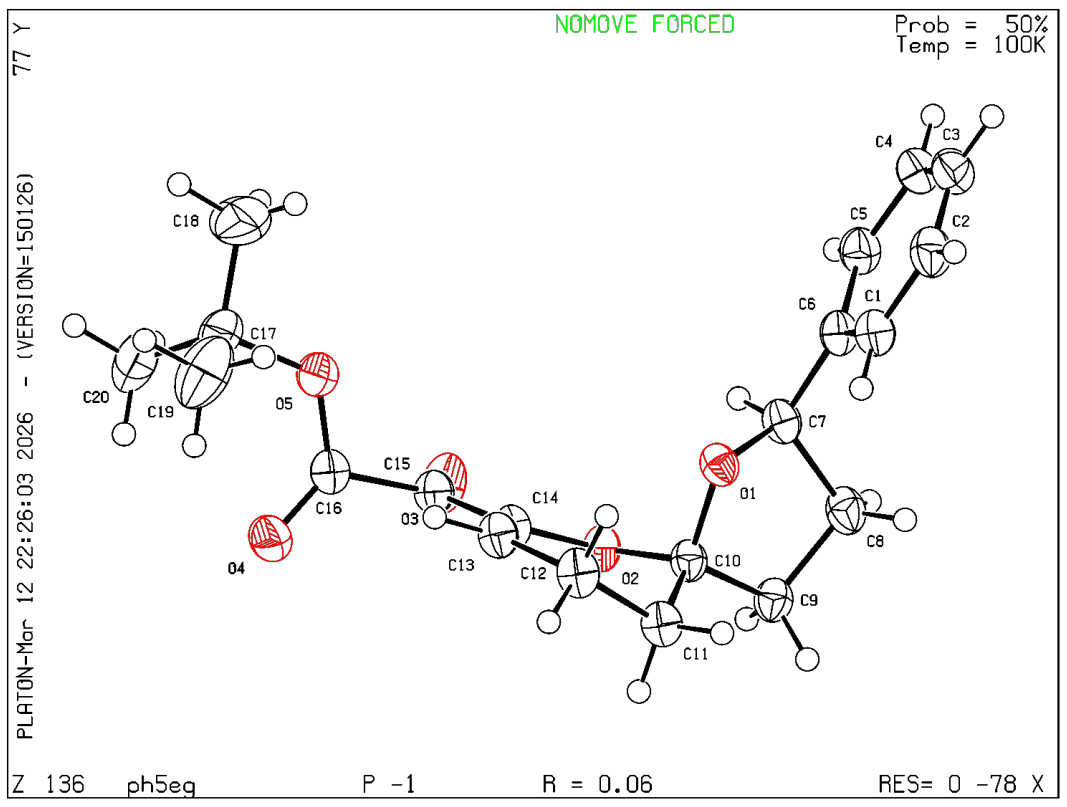

A colorless, block-shaped crystal was mounted on a MiTeGen Micromesh mount with Paratone N oil. Data for ph5eg were collected from a shock-cooled single crystal at 100(2) K on a Bruker three-axis goniometer with a sealed X-ray tube using a multi-layer optics as monochromator and a Bruker Apex-II CCD detector. The diffractometer was equipped with an Oxford Cryostream 700 low temperature device and used CuK_{α} radiation (λ = 1.54178 Å). All data were integrated with SAINT V8.40B and a multi-scan absorption correction using SADABS 2016/2 was applied.[1,2] The structure was solved by direct methods with SHELXT 2018/2 and refined by full-matrix least-squares methods against F^2 using SHELXL-2019/2.[3,4] All non-hydrogen atoms were refined with anisotropic displacement parameters. All hydrogen atoms were refined isotropic on calculated positions using a riding model with their U_{iso} values constrained to 1.5 times the U_{eq} of their pivot atoms for terminal sp^3 carbon atoms and 1.2 times for all other carbon atoms. This report and the CIF file were generated using FinalCif.[5]

Refinement Details:

All non-hydrogen atoms were refined as anisotropic ellipsoids without restraints or constraints. All hydrogen atoms were refined using a riding model.

Table 1. Crystal data and structure refinement for ph5eg

CCDC number	2537372
Empirical formula	$C_{20}H_{24}O_5$
Formula weight	344.39
Temperature [K]	100(2)
Crystal system	triclinic
Space group (number)	$P\overline{1}$ (2)
a [Å]	6.230(3)
b [Å]	12.340(6)
c [Å]	12.975(7)
α [°]	75.951(15)
β [°]	83.360(14)
γ [°]	80.687(12)
Volume [Å^3]	951.8(8)
Z	2
ρ_{calc} [gcm^{-3}]	1.202
μ [mm^{-1}]	0.700
F(000)	368
Crystal size [mm^3]	0.050×0.400×0.400
Crystal colour	colorless
Crystal shape	block
Radiation	CuK_{α} (λ=1.54178 Å)
2θ range [°]	7.05 to 147.92 (0.80 Å)
Index ranges	−7 ≤ h ≤ 7<br>−13 ≤ k ≤ 14<br>−16 ≤ l ≤ 16
Reflections collected	27672
Independent reflections	3674<br>R_{int} = 0.0663<br>R_{sigma} = 0.0351
Completeness to θ = 67.679°	96.6 %
Data / Restraints / Parameters	3674 / 0 / 230
Absorption correction T_{min}/T_{max} (method)	0.6096 / 0.7538 (multi-scan)
Goodness-of-fit on F^2	1.152
Final R indexes [I≥2σ(I)]	R_1 = 0.0560<br>wR_2 = 0.1561
Final R indexes [all data]	R_1 = 0.0641<br>wR_2 = 0.1631
Largest peak/hole [eÅ^{-3}]	0.23/−0.31
Extinction coefficient	0.0047(10)

Table 1. Atomic coordinates and U_{eq} [Å^2] for ph5eg

Atom	*x*	*y*	*z*	U_{eq}
O1	0.3897(2)	0.74806(11)	0.12567(11)	0.0295(3)
O2	0.3120(2)	0.57409(11)	0.11233(11)	0.0292(3)
O3	0.1340(2)	0.40630(14)	0.25366(13)	0.0440(4)
O4	0.5624(3)	0.24151(13)	0.31096(13)	0.0423(4)
O5	0.4316(3)	0.34734(13)	0.43061(12)	0.0428(4)
C1	0.3434(3)	0.97011(17)	0.15846(17)	0.0340(4)
H1	0.473519	0.946703	0.118995	0.041
C2	0.3297(4)	1.06417(18)	0.20074(18)	0.0382(5)
H2	0.451245	1.104211	0.191085	0.046
C3	0.1391(4)	1.10018(18)	0.25716(18)	0.0406(5)
H3	0.129715	1.165221	0.285268	0.049
C4	−0.0375(4)	1.04087(18)	0.27234(18)	0.0398(5)
H4	−0.168517	1.065470	0.310494	0.048
C5	−0.0223(3)	0.94545(18)	0.23161(17)	0.0353(5)
H5	−0.142473	0.904194	0.243527	0.042
C6	0.1663(3)	0.90944(16)	0.17367(16)	0.0311(4)
C7	0.1732(3)	0.80999(16)	0.12500(16)	0.0302(4)
H7	0.067824	0.759599	0.167078	0.036
C8	0.1301(3)	0.83810(17)	0.00736(17)	0.0354(5)
H8A	0.192590	0.906720	−0.031973	0.043
H8B	−0.028324	0.849614	−0.001492	0.043
C9	0.2452(3)	0.73390(16)	−0.03107(16)	0.0309(4)
H9A	0.326423	0.755785	−0.101444	0.037
H9B	0.139259	0.684107	−0.036328	0.037
C10	0.4020(3)	0.67541(15)	0.05550(15)	0.0281(4)
C11	0.6394(3)	0.64658(17)	0.01907(16)	0.0321(4)
H11A	0.698062	0.716108	−0.020859	0.039
H11B	0.652359	0.594485	−0.029262	0.039
C12	0.7716(3)	0.59117(17)	0.11509(17)	0.0343(5)
H12A	0.915897	0.555084	0.090666	0.041
H12B	0.795509	0.649329	0.151743	0.041
C13	0.6537(3)	0.50506(16)	0.19043(16)	0.0311(4)
H13	0.730905	0.451540	0.244023	0.037
C14	0.4416(3)	0.50050(15)	0.18518(15)	0.0278(4)
C15	0.3255(3)	0.41404(16)	0.25640(16)	0.0305(4)
C16	0.4589(3)	0.32368(16)	0.33590(16)	0.0304(4)
C17	0.5358(4)	0.2699(2)	0.52429(18)	0.0494(6)
C18	0.4428(8)	0.3282(3)	0.6134(3)	0.1016(16)
H18A	0.493718	0.401845	0.599757	0.152
H18B	0.491578	0.281698	0.681403	0.152
H18C	0.283059	0.338642	0.616488	0.152
C19	0.7802(5)	0.2639(3)	0.5040(3)	0.0717(9)
H19A	0.830106	0.227857	0.444396	0.108
H19B	0.849489	0.219665	0.568053	0.108
H19C	0.820003	0.340323	0.486536	0.108
C20	0.4625(5)	0.1557(2)	0.5438(2)	0.0615(8)
H20A	0.302877	0.164435	0.547133	0.092
H20B	0.512780	0.108891	0.611459	0.092
H20C	0.524263	0.119386	0.485618	0.092

U_{eq} is defined as 1/3 of the trace of the orthogonalized U_{ij} tensor.

Table 2. Anisotropic displacement parameters [Å^2] for ph5eg. The anisotropic displacement factor exponent takes the form: $-2\pi^2[\, h^2(a^*)^2U_{11} + k^2(b^*)^2U_{22} + \ldots + 2hka^*b^*U_{12} \,]$

Atom	U_{11}	U_{22}	U_{33}	U_{23}	U_{13}	U_{12}
O1	0.0271(7)	0.0271(7)	0.0353(7)	−0.0084(5)	−0.0085(5)	−0.0006(5)
O2	0.0244(6)	0.0266(7)	0.0355(7)	−0.0021(5)	−0.0073(5)	−0.0044(5)
O3	0.0268(8)	0.0469(10)	0.0521(10)	0.0060(7)	−0.0099(6)	−0.0087(6)
O4	0.0464(9)	0.0364(9)	0.0418(8)	−0.0102(6)	−0.0085(7)	0.0065(6)
O5	0.0536(10)	0.0380(9)	0.0335(8)	−0.0078(6)	−0.0119(7)	0.0087(7)
C1	0.0333(10)	0.0296(11)	0.0384(11)	−0.0053(8)	−0.0045(8)	−0.0049(8)
C2	0.0410(12)	0.0305(11)	0.0439(12)	−0.0050(8)	−0.0092(9)	−0.0083(8)
C3	0.0506(13)	0.0306(12)	0.0417(12)	−0.0107(8)	−0.0084(9)	−0.0018(9)
C4	0.0427(12)	0.0336(12)	0.0407(11)	−0.0089(8)	−0.0037(9)	0.0030(8)
C5	0.0324(10)	0.0324(11)	0.0390(11)	−0.0051(8)	−0.0038(8)	−0.0020(8)
C6	0.0285(10)	0.0278(10)	0.0352(10)	−0.0036(7)	−0.0054(7)	−0.0022(7)
C7	0.0250(9)	0.0276(10)	0.0374(10)	−0.0062(7)	−0.0041(7)	−0.0026(7)
C8	0.0355(11)	0.0299(11)	0.0414(11)	−0.0075(8)	−0.0140(8)	0.0005(8)
C9	0.0314(10)	0.0292(10)	0.0319(10)	−0.0039(7)	−0.0064(8)	−0.0058(7)
C10	0.0295(9)	0.0237(10)	0.0314(9)	−0.0046(7)	−0.0045(7)	−0.0057(7)
C11	0.0268(9)	0.0316(11)	0.0374(10)	−0.0061(8)	−0.0037(8)	−0.0045(7)
C12	0.0244(9)	0.0334(11)	0.0443(12)	−0.0052(8)	−0.0053(8)	−0.0054(7)
C13	0.0257(9)	0.0304(11)	0.0372(10)	−0.0058(8)	−0.0074(7)	−0.0030(7)
C14	0.0266(9)	0.0232(10)	0.0332(10)	−0.0053(7)	−0.0053(7)	−0.0021(7)
C15	0.0267(9)	0.0304(11)	0.0337(10)	−0.0055(7)	−0.0054(7)	−0.0031(7)
C16	0.0270(9)	0.0287(10)	0.0351(10)	−0.0048(7)	−0.0037(7)	−0.0054(7)
C17	0.0602(15)	0.0490(14)	0.0326(11)	−0.0041(9)	−0.0147(10)	0.0108(11)
C18	0.159(4)	0.092(3)	0.0436(17)	−0.0272(16)	−0.038(2)	0.050(3)
C19	0.0633(19)	0.087(2)	0.0598(18)	0.0086(15)	−0.0322(14)	−0.0167(15)
C20	0.0621(17)	0.0602(18)	0.0460(15)	0.0138(12)	−0.0022(12)	−0.0024(13)

Table 3. Bond lengths and angles for ph5eg

Atom–Atom	Length [Å]
O1–C10	1.413(2)
O1–C7	1.438(2)
O2–C14	1.375(2)
O2–C10	1.451(2)
O3–C15	1.217(2)
O4–C16	1.203(2)
O5–C16	1.317(3)
O5–C17	1.493(3)
C1–C2	1.386(3)
C1–C6	1.401(3)
C2–C3	1.389(3)
C3–C4	1.388(3)
C4–C5	1.389(3)
C5–C6	1.390(3)
C6–C7	1.503(3)
C7–C8	1.526(3)
C8–C9	1.532(3)
C9–C10	1.539(3)
C10–C11	1.512(3)

C11–C12	1.530(3)
C12–C13	1.485(3)
C13–C14	1.342(3)
C14–C15	1.460(3)
C15–C16	1.529(3)
C17–C20	1.506(4)
C17–C19	1.508(4)
C17–C18	1.517(4)

**Atom–Atom–Atom**	**Angle [°]**
C10–O1–C7	107.18(14)
C14–O2–C10	115.45(14)
C16–O5–C17	121.64(17)
C2–C1–C6	120.3(2)
C1–C2–C3	120.3(2)
C4–C3–C2	119.8(2)
C3–C4–C5	119.8(2)
C4–C5–C6	121.0(2)
C5–C6–C1	118.76(19)
C5–C6–C7	119.75(18)
C1–C6–C7	121.44(18)
O1–C7–C6	109.82(15)
O1–C7–C8	102.79(15)
C6–C7–C8	115.73(16)
C7–C8–C9	103.40(15)
C8–C9–C10	103.62(16)
O1–C10–O2	108.60(15)
O1–C10–C11	108.13(15)
O2–C10–C11	110.35(15)
O1–C10–C9	106.82(15)
O2–C10–C9	105.38(14)
C11–C10–C9	117.25(16)
C10–C11–C12	110.24(17)
C13–C12–C11	110.16(16)
C14–C13–C12	122.07(18)
C13–C14–O2	124.35(18)
C13–C14–C15	122.65(18)
O2–C14–C15	112.99(16)
O3–C15–C14	124.72(18)
O3–C15–C16	118.19(18)
C14–C15–C16	117.01(16)
O4–C16–O5	127.85(19)
O4–C16–C15	121.44(18)
O5–C16–C15	110.62(16)
O5–C17–C20	109.8(2)
O5–C17–C19	108.9(2)
C20–C17–C19	112.1(2)
O5–C17–C18	101.6(2)
C20–C17–C18	110.7(3)
C19–C17–C18	113.2(3)

Table 4. Torsion angles for ph5eg

**Atom–Atom–Atom–Atom**	**Torsion Angle [°]**
C6–C1–C2–C3	−1.0(3)
C1–C2–C3–C4	0.8(3)
C2–C3–C4–C5	0.4(3)
C3–C4–C5–C6	−1.4(3)
C4–C5–C6–C1	1.1(3)
C4–C5–C6–C7	−176.33(19)
C2–C1–C6–C5	0.1(3)
C2–C1–C6–C7	177.47(19)
C10–O1–C7–C6	−164.88(15)
C10–O1–C7–C8	−41.17(18)
C5–C6–C7–O1	−146.48(18)
C1–C6–C7–O1	36.1(2)
C5–C6–C7–C8	97.7(2)
C1–C6–C7–C8	−79.6(2)
O1–C7–C8–C9	35.47(19)
C6–C7–C8–C9	155.16(16)
C7–C8–C9–C10	−17.80(19)
C7–O1–C10–O2	−83.34(17)
C7–O1–C10–C11	156.90(15)
C7–O1–C10–C9	29.87(19)
C14–O2–C10–O1	−73.91(18)
C14–O2–C10–C11	44.5(2)
C14–O2–C10–C9	171.93(15)
C8–C9–C10–O1	−6.2(2)
C8–C9–C10–O2	109.21(17)
C8–C9–C10–C11	−127.61(18)
O1–C10–C11–C12	58.2(2)
O2–C10–C11–C12	−60.4(2)
C9–C10–C11–C12	178.97(16)
C10–C11–C12–C13	44.7(2)
C11–C12–C13–C14	−15.4(3)
C12–C13–C14–O2	−0.9(3)

C12–C13–C14–C15	177.89(18)
C10–O2–C14–C13	−14.1(3)
C10–O2–C14–C15	166.95(15)
C13–C14–C15–O3	−179.3(2)
O2–C14–C15–O3	−0.3(3)
C13–C14–C15–C16	−2.6(3)
O2–C14–C15–C16	176.33(16)
C17–O5–C16–O4	0.8(3)
C17–O5–C16–C15	177.19(19)
O3–C15–C16–O4	92.9(3)
C14–C15–C16–O4	−84.0(2)
O3–C15–C16–O5	−83.7(2)
C14–C15–C16–O5	99.4(2)
C16–O5–C17–C20	−58.5(3)
C16–O5–C17–C19	64.6(3)
C16–O5–C17–C18	−175.7(3)

Bibliography

[1] Bruker, *SAINT*, *V8.40B*, Bruker AXS SE, Karlsruhe, Germany.

[2] L. Krause, R. Herbst-Irmer, G. M. Sheldrick, D. Stalke, *J. Appl. Cryst.* **2015**, *48*, 3–10, doi:10.1107/S1600576714022985.

[3] G. M. Sheldrick, *Acta Cryst.* **2015**, *A71*, 3–8, doi:10.1107/S2053273314026370.

[4] G. M. Sheldrick, *Acta Cryst.* **2015**, *C71*, 3–8, doi:10.1107/S2053229614024218.

[5] D. Kratzert, *FinalCif*, *V156*, https://dkratzert.de/finalcif.html.

Structure Tables

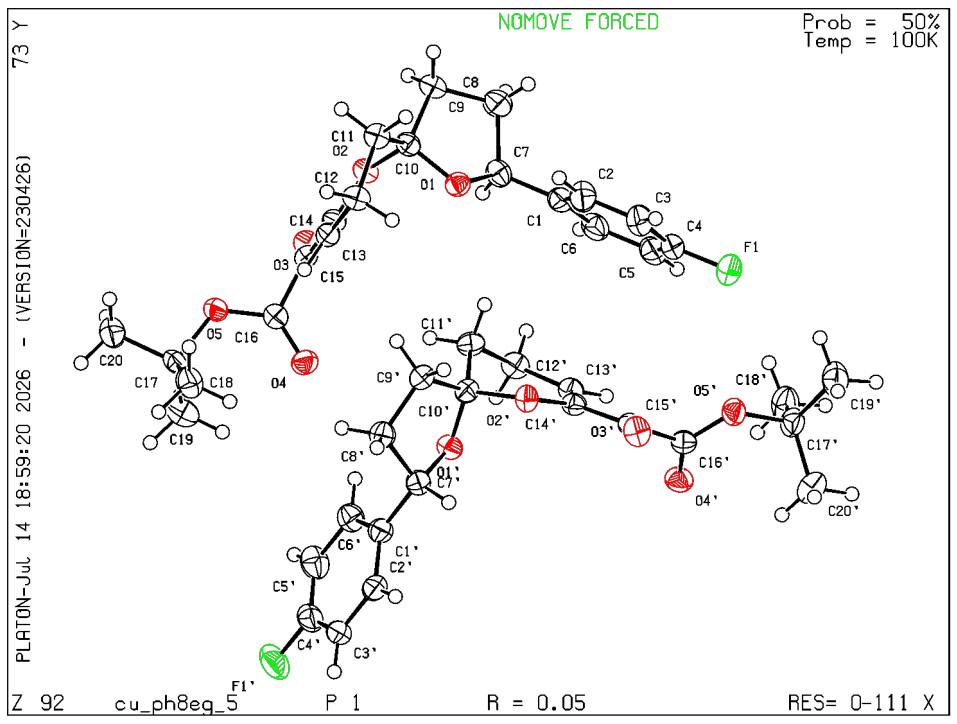

A colourless, plate-shaped crystal was mounted on a MiTeGen Micromesh mount with Paratone N oil with perfluoroether oil. Data for ph8eg were collected from a shock-cooled single crystal at 100(2) K on a Bruker three-circle diffractometer with a sealed microsource X-ray tube using Quazar multi-layer optics as monochromator and a Bruker Apex-II CCD detector. The diffractometer was equipped with an Oxford Cryostream 700 low temperature device and used CuK_α radiation (λ = 1.54178 Å). All data were integrated with SAINT V8.42, and a multi-scan absorption correction using TWINABS Bruker was applied.[1,2] The structure was solved by direct methods with SHELXT and refined by full-matrix least-squares methods against F^2 using SHELXL-2025/1.[3,4] All non-hydrogen atoms were refined with anisotropic displacement parameters. All hydrogen atoms were refined isotropic on calculated positions using a riding model with their U_{iso} values constrained to 1.5 times the U_{eq} of their pivot atoms for terminal sp^3 carbon atoms and 1.2 times for all other carbon atoms. Crystallographic data for the structures reported in this paper have been deposited with the Cambridge Crystallographic Data Centre.[5] CCDC 2573303 contain the supplementary crystallographic data for this paper. These data can be obtained free of charge from The Cambridge Crystallographic Data Centre via www.ccdc.cam.ac.uk/structures. This report and the CIF file were generated using FinalCif.[6]

Refinement Details: The structure was refined as a two-component non-merohedral twin on the reflections of the major component. All non-hydrogen atoms were refined as anisotropic ellipsoids without restraints or constraints. All hydrogen atoms were refined using a riding model. Two molecules of the same absolute configuration were found in the P1 asymmetric unit, and the Flack tests show likely inversion twinning in addition to the non-merohedral twinning that was modeled.

Table 5. Crystal data and structure refinement for ph8eg

CCDC number	2573303
Empirical formula	$C_{20}H_{23}FO_5$
Formula weight	362.38
Temperature [K]	100(2)
Crystal system	triclinic
Space group (number)	$P1$ (1)
a [Å]	6.1741(3)
b [Å]	10.1847(5)
c [Å]	15.3003(7)
α [°]	86.428(3)
β [°]	81.139(3)
γ [°]	85.049(3)
Volume [Å^3]	945.90(8)
Z	2
ρ_{calc} [gcm^{-3}]	1.272
μ [mm^{-1}]	0.810
F(000)	384
Crystal size [mm^3]	0.043×0.115×0.183
Crystal colour	colourless
Crystal shape	plate
Radiation	CuK_α (λ=1.54178 Å)
2θ range [°]	5.85 to 140.34 (0.82 Å)
Index ranges	−7 ≤ h ≤ 7<br>−12 ≤ k ≤ 12<br>−18 ≤ l ≤ 18
Reflections collected	7460
Independent reflections	7460<br>R_{int} = 0.089<br>R_{sigma} = 0.0487
Completeness to θ = 67.679°	96.2 %
Data / Restraints / Parameters	7460 / 3 / 477
Absorption correction T_{min}/T_{max} (method)	0.6558 / 0.7533 (multi-scan)
Goodness-of-fit on F^2	1.069
Final R indexes [I≥2σ(I)]	R_1 = 0.0537<br>wR_2 = 0.1361
Final R indexes [all data]	R_1 = 0.0643<br>wR_2 = 0.1434
Largest peak/hole [eÅ^{-3}]	0.21/-0.19
Extinction coefficient	0.0089(15)
Flack X parameter	0.46(17)

Table 6. Atomic coordinates and U_{eq} [Å^2] for ph8eg

Atom	*x*	*y*	*z*	U_{eq}
F1	0.6584(8)	0.5224(4)	0.1059(3)	0.0663(12)
O1	0.3855(5)	0.8506(3)	0.4422(2)	0.0297(7)
O2	0.1462(5)	0.9022(3)	0.5704(2)	0.0283(7)
O3	−0.0778(5)	0.7438(4)	0.6952(2)	0.0324(8)
O4	0.3298(6)	0.5560(3)	0.7393(2)	0.0349(8)
O5	0.2005(6)	0.7110(3)	0.8392(2)	0.0303(7)
C1	0.3454(9)	0.7383(5)	0.3129(4)	0.0353(11)
C2	0.5548(10)	0.7664(6)	0.2716(4)	0.0417(13)
H2	0.624762	0.836088	0.291421	0.050
C3	0.6622(11)	0.6933(6)	0.2019(4)	0.0479(14)
H3	0.805221	0.711531	0.173587	0.058
C4	0.5545(12)	0.5939(6)	0.1753(4)	0.0481(15)
C5	0.3486(12)	0.5627(6)	0.2132(4)	0.0498(15)
H5	0.279445	0.493465	0.192469	0.060
C6	0.2440(10)	0.6371(6)	0.2839(4)	0.0408(13)
H6	0.101585	0.617572	0.312292	0.049
C7	0.2279(9)	0.8186(5)	0.3883(3)	0.0337(11)
H7	0.113137	0.766690	0.424877	0.040
C8	0.1245(11)	0.9542(6)	0.3601(4)	0.0475(15)
H8A	0.210275	0.990848	0.305367	0.057
H8B	−0.028991	0.948467	0.350165	0.057
C9	0.1332(8)	1.0381(5)	0.4385(3)	0.0319(11)
H9A	−0.014701	1.053938	0.473373	0.038
H9B	0.191571	1.124081	0.418195	0.038
C10	0.2883(8)	0.9559(5)	0.4936(3)	0.0283(10)
C11	0.4682(8)	1.0267(5)	0.5237(3)	0.0302(10)
H11A	0.401466	1.099430	0.561502	0.036
H11B	0.561068	1.065358	0.471476	0.036
C12	0.6099(8)	0.9302(5)	0.5757(3)	0.0305(10)
H12A	0.711820	0.874650	0.534114	0.037
H12B	0.698874	0.979871	0.608783	0.037
C13	0.4691(8)	0.8446(5)	0.6387(3)	0.0280(10)
H13	0.531346	0.795572	0.684817	0.034
C14	0.2584(8)	0.8346(5)	0.6324(3)	0.0261(10)
C15	0.1187(8)	0.7500(4)	0.6944(3)	0.0262(10)
C16	0.2336(8)	0.6603(5)	0.7601(3)	0.0280(10)
C17	0.2909(8)	0.6405(5)	0.9160(3)	0.0326(11)
C18	0.5408(9)	0.6294(7)	0.8956(4)	0.0421(13)
H18A	0.588624	0.567836	0.848392	0.063
H18B	0.602240	0.596726	0.948944	0.063
H18C	0.592422	0.716346	0.876347	0.063
C19	0.1957(11)	0.5077(6)	0.9349(4)	0.0471(14)
H19A	0.035871	0.519038	0.936704	0.071
H19B	0.230004	0.469819	0.992049	0.071
H19C	0.259728	0.448363	0.888025	0.071
C20	0.2077(11)	0.7339(7)	0.9898(4)	0.0474(14)
H20A	0.267585	0.819739	0.974349	0.071
H20B	0.255071	0.697046	1.045082	0.071
H20C	0.046726	0.744986	0.997420	0.071

F1'	0.5745(8)	−0.0373(4)	0.9174(2)	0.0634(11)
O1'	0.6542(5)	0.3261(3)	0.5711(2)	0.0300(7)
O2'	0.8279(5)	0.4098(3)	0.4353(2)	0.0291(7)
O3'	1.0756(6)	0.3044(4)	0.2917(2)	0.0362(8)
O4'	0.6926(6)	0.1026(3)	0.2757(3)	0.0368(8)
O5'	0.8340(6)	0.2378(3)	0.1619(2)	0.0351(8)
C1'	0.7595(8)	0.2093(5)	0.7010(3)	0.0315(11)
C2'	0.9120(10)	0.1145(5)	0.7305(4)	0.0371(11)
H2'	1.058775	0.107238	0.700395	0.044
C3'	0.8510(11)	0.0305(6)	0.8037(4)	0.0457(15)
H3'	0.953683	−0.034111	0.824700	0.055
C4'	0.6356(12)	0.0447(6)	0.8447(4)	0.0454(14)
C5'	0.4815(11)	0.1363(6)	0.8179(4)	0.0453(14)
H5'	0.335053	0.142601	0.848328	0.054
C6'	0.5446(9)	0.2206(6)	0.7443(4)	0.0378(12)
H6'	0.440823	0.285188	0.724086	0.045
C7'	0.8310(8)	0.3013(5)	0.6232(3)	0.0302(10)
H7'	0.963520	0.259745	0.586005	0.036
C8'	0.8775(9)	0.4390(5)	0.6460(4)	0.0341(11)
H8'A	1.033954	0.442211	0.652605	0.041
H8'B	0.784340	0.466057	0.701581	0.041
C9'	0.8200(8)	0.5278(5)	0.5667(3)	0.0300(10)
H9'A	0.731328	0.608973	0.586774	0.036
H9'B	0.954960	0.552819	0.527667	0.036
C10'	0.6879(8)	0.4437(5)	0.5185(3)	0.0285(10)
C11'	0.4670(8)	0.5052(5)	0.4999(4)	0.0315(11)
H11C	0.487414	0.585760	0.460819	0.038
H11D	0.375057	0.530765	0.556060	0.038
C12'	0.3520(8)	0.4069(5)	0.4553(3)	0.0329(11)
H12C	0.291836	0.339722	0.499982	0.040
H12D	0.228009	0.453531	0.429127	0.040
C13'	0.5082(8)	0.3416(5)	0.3854(3)	0.0296(10)
H13'	0.451808	0.294355	0.343354	0.036
C14'	0.7243(8)	0.3463(5)	0.3788(3)	0.0273(10)
C15'	0.8811(8)	0.2891(5)	0.3055(3)	0.0297(10)
C16'	0.7858(8)	0.1978(5)	0.2465(3)	0.0287(10)
C17'	0.7696(10)	0.1628(5)	0.0908(4)	0.0380(12)
C18'	0.5220(10)	0.1644(7)	0.1023(4)	0.0494(15)
H18D	0.457820	0.255563	0.106863	0.074
H18E	0.469460	0.112521	0.156301	0.074
H18F	0.478176	0.126119	0.051072	0.074
C19'	0.8568(13)	0.2423(7)	0.0079(4)	0.0577(18)
H19D	0.820536	0.202249	−0.044117	0.087
H19E	1.016742	0.243102	0.003257	0.087
H19F	0.789455	0.332927	0.010848	0.087
C20'	0.8834(11)	0.0258(6)	0.0919(4)	0.0480(14)
H20D	0.849326	−0.021545	0.042448	0.072
H20E	0.832119	−0.021683	0.147892	0.072
H20F	1.042620	0.031637	0.085921	0.072

U_{eq} is defined as 1/3 of the trace of the orthogonalized U_{ij} tensor.

Table 7. Anisotropic displacement parameters [Å^2] for ph8eg. The anisotropic displacement factor exponent takes the form: $-2\pi^2[\, h^2(a^*)^2U_{11} + k^2(b^*)^2U_{22} + \ldots + 2hka^*b^*U_{12}\,]$

Atom	U_{11}	U_{22}	U_{33}	U_{23}	U_{13}	U_{12}
F1	0.116(4)	0.044(2)	0.035(2)	−0.0098(16)	−0.002(2)	0.008(2)
O1	0.0283(18)	0.0292(17)	0.0318(18)	−0.0020(14)	−0.0053(13)	−0.0010(12)
O2	0.0233(18)	0.0322(17)	0.0285(17)	0.0037(14)	−0.0035(12)	−0.0023(12)
O3	0.027(2)	0.0353(19)	0.0352(19)	0.0019(15)	−0.0053(14)	−0.0048(13)
O4	0.036(2)	0.0313(19)	0.038(2)	−0.0042(15)	−0.0104(15)	0.0035(14)
O5	0.0317(19)	0.0307(18)	0.0278(17)	0.0011(14)	−0.0054(13)	0.0028(13)
C1	0.045(3)	0.031(3)	0.032(3)	0.000(2)	−0.012(2)	−0.003(2)
C2	0.047(3)	0.044(3)	0.035(3)	−0.004(2)	−0.003(2)	−0.010(2)
C3	0.059(4)	0.048(3)	0.034(3)	−0.002(3)	0.000(3)	−0.003(3)
C4	0.083(5)	0.033(3)	0.026(3)	−0.001(2)	−0.007(3)	0.006(3)
C5	0.083(5)	0.036(3)	0.034(3)	0.001(2)	−0.019(3)	−0.010(3)
C6	0.056(4)	0.035(3)	0.035(3)	0.004(2)	−0.015(2)	−0.014(2)
C7	0.033(3)	0.039(3)	0.031(3)	0.000(2)	−0.009(2)	−0.006(2)
C8	0.056(4)	0.046(3)	0.044(3)	−0.004(3)	−0.026(3)	0.010(3)
C9	0.030(3)	0.032(3)	0.033(3)	0.006(2)	−0.0065(19)	−0.0023(18)
C10	0.028(3)	0.030(2)	0.027(2)	0.0023(19)	−0.0028(18)	−0.0038(18)
C11	0.025(3)	0.032(3)	0.033(3)	0.000(2)	−0.0030(19)	−0.0057(18)
C12	0.023(3)	0.033(3)	0.036(3)	0.003(2)	−0.0051(19)	−0.0036(18)
C13	0.026(3)	0.029(2)	0.030(3)	−0.0034(19)	−0.0069(18)	−0.0005(17)
C14	0.024(3)	0.027(2)	0.027(2)	0.0010(19)	−0.0053(18)	−0.0011(17)
C15	0.026(3)	0.024(2)	0.029(2)	−0.0040(19)	−0.0035(17)	−0.0006(17)
C16	0.024(2)	0.031(2)	0.030(3)	−0.0004(19)	−0.0060(18)	−0.0050(17)
C17	0.037(3)	0.037(3)	0.024(2)	0.004(2)	−0.0099(19)	0.003(2)
C18	0.038(3)	0.054(4)	0.035(3)	−0.002(2)	−0.008(2)	−0.003(2)
C19	0.047(4)	0.047(3)	0.046(3)	0.011(3)	−0.008(3)	−0.007(3)

C20	0.056(4)	0.053(4)	0.031(3)	−0.003(3)	−0.004(2)	0.007(3)
F1'	0.106(3)	0.053(2)	0.0343(19)	0.0108(17)	−0.0136(19)	−0.029(2)
O1'	0.0297(18)	0.0302(18)	0.0315(19)	0.0014(14)	−0.0085(13)	−0.0057(13)
O2'	0.0237(18)	0.0369(18)	0.0275(17)	−0.0052(14)	−0.0042(12)	−0.0031(13)
O3'	0.027(2)	0.047(2)	0.035(2)	−0.0024(16)	−0.0047(14)	−0.0060(14)
O4'	0.044(2)	0.030(2)	0.038(2)	0.0036(15)	−0.0088(15)	−0.0085(15)
O5'	0.045(2)	0.0321(19)	0.0288(19)	−0.0038(15)	−0.0039(15)	−0.0096(15)
C1'	0.035(3)	0.029(3)	0.032(3)	−0.002(2)	−0.008(2)	−0.0036(19)
C2'	0.044(3)	0.033(3)	0.035(3)	−0.008(2)	−0.011(2)	0.005(2)
C3'	0.078(5)	0.029(3)	0.032(3)	−0.001(2)	−0.019(3)	0.003(3)
C4'	0.078(5)	0.030(3)	0.031(3)	0.003(2)	−0.011(3)	−0.019(3)
C5'	0.047(3)	0.051(3)	0.038(3)	0.001(3)	−0.002(2)	−0.018(3)
C6'	0.037(3)	0.041(3)	0.035(3)	0.000(2)	−0.004(2)	−0.004(2)
C7'	0.027(3)	0.033(3)	0.030(3)	−0.002(2)	−0.0039(18)	−0.0004(19)
C8'	0.035(3)	0.034(3)	0.035(3)	−0.003(2)	−0.010(2)	−0.0056(19)
C9'	0.029(3)	0.028(2)	0.033(3)	−0.004(2)	−0.0039(19)	−0.0023(18)
C10'	0.028(3)	0.027(2)	0.030(2)	−0.0028(19)	−0.0021(18)	−0.0022(17)
C11'	0.024(3)	0.032(3)	0.039(3)	−0.001(2)	−0.0053(19)	0.0024(18)
C12'	0.022(2)	0.039(3)	0.037(3)	0.002(2)	−0.0038(19)	−0.0020(18)
C13'	0.028(3)	0.030(2)	0.032(3)	0.001(2)	−0.0081(19)	−0.0045(18)
C14'	0.028(3)	0.025(2)	0.030(3)	0.0011(19)	−0.0072(19)	−0.0035(17)
C15'	0.028(3)	0.030(3)	0.031(3)	0.001(2)	−0.0072(19)	−0.0019(18)
C16'	0.026(3)	0.028(2)	0.032(3)	0.000(2)	−0.0055(19)	−0.0020(17)
C17'	0.047(3)	0.035(3)	0.033(3)	−0.008(2)	−0.005(2)	−0.009(2)
C18'	0.045(4)	0.064(4)	0.044(3)	−0.015(3)	−0.016(3)	−0.002(3)
C19'	0.088(5)	0.051(4)	0.035(3)	−0.007(3)	−0.003(3)	−0.023(3)
C20'	0.052(4)	0.042(3)	0.051(4)	−0.015(3)	−0.004(3)	0.000(3)

Table 8. Bond lengths and angles for ph8eg

Atom–Atom	Length [Å]
F1–C4	1.369(7)
O1–C10	1.406(6)
O1–C7	1.438(6)
O2–C14	1.375(6)
O2–C10	1.462(5)
O3–C15	1.218(6)
O4–C16	1.208(6)
O5–C16	1.325(6)
O5–C17	1.495(5)
C1–C6	1.381(7)
C1–C2	1.393(8)
C1–C7	1.511(7)
C2–C3	1.388(8)
C3–C4	1.373(9)
C4–C5	1.368(10)
C5–C6	1.401(9)
C7–C8	1.536(8)
C8–C9	1.525(8)
C9–C10	1.532(6)
C10–C11	1.518(7)
C11–C12	1.528(7)
C12–C13	1.487(7)
C13–C14	1.332(7)
C14–C15	1.470(6)
C15–C16	1.530(7)
C17–C19	1.515(8)
C17–C20	1.521(8)
C17–C18	1.522(8)
F1'–C4'	1.374(6)
O1'–C10'	1.413(6)
O1'–C7'	1.444(6)
O2'–C14'	1.373(6)
O2'–C10'	1.467(6)
O3'–C15'	1.209(6)
O4'–C16'	1.197(6)
O5'–C16'	1.329(6)
O5'–C17'	1.488(6)
C1'–C6'	1.387(7)
C1'–C2'	1.394(7)
C1'–C7'	1.503(7)
C2'–C3'	1.391(8)
C3'–C4'	1.380(9)
C4'–C5'	1.365(10)
C5'–C6'	1.399(8)
C7'–C8'	1.527(7)
C8'–C9'	1.535(7)
C9'–C10'	1.525(7)
C10'–C11'	1.512(7)
C11'–C12'	1.526(7)
C12'–C13'	1.475(7)
C13'–C14'	1.327(7)
C14'–C15'	1.478(7)
C15'–C16'	1.546(7)
C17'–C20'	1.507(8)
C17'–C18'	1.510(9)
C17'–C19'	1.514(8)

Atom–Atom–Atom	Angle [°]
C10–O1–C7	107.2(4)
C14–O2–C10	114.0(4)
C16–O5–C17	121.2(4)
C6–C1–C2	119.6(5)
C6–C1–C7	119.9(5)
C2–C1–C7	120.6(5)
C3–C2–C1	120.6(5)
C4–C3–C2	117.7(6)
C5–C4–F1	118.0(6)
C5–C4–C3	124.0(6)
F1–C4–C3	118.0(6)
C4–C5–C6	117.3(6)
C1–C6–C5	120.8(6)
O1–C7–C1	108.9(4)
O1–C7–C8	103.4(4)
C1–C7–C8	114.8(5)
C9–C8–C7	103.5(4)
C8–C9–C10	104.4(4)
O1–C10–O2	108.4(4)
O1–C10–C11	108.9(4)
O2–C10–C11	110.0(4)
O1–C10–C9	106.8(4)
O2–C10–C9	105.4(4)
C11–C10–C9	116.9(4)
C10–C11–C12	110.0(4)
C13–C12–C11	110.4(4)
C14–C13–C12	122.1(4)
C13–C14–O2	125.1(4)
C13–C14–C15	122.3(4)
O2–C14–C15	112.6(4)
O3–C15–C14	124.9(4)
O3–C15–C16	118.5(4)

C14–C15–C16	116.6(4)
O4–C16–O5	127.7(4)
O4–C16–C15	121.6(4)
O5–C16–C15	110.6(4)
O5–C17–C19	109.6(4)
O5–C17–C20	101.6(4)
C19–C17–C20	111.6(5)
O5–C17–C18	108.8(4)
C19–C17–C18	112.9(5)
C20–C17–C18	111.7(5)
C10'–O1'–C7'	108.4(4)
C14'–O2'–C10'	114.1(4)
C16'–O5'–C17'	120.7(4)
C6'–C1'–C2'	120.3(5)
C6'–C1'–C7'	120.3(5)
C2'–C1'–C7'	119.5(5)
C3'–C2'–C1'	120.5(6)
C4'–C3'–C2'	117.4(5)
C5'–C4'–F1'	118.4(6)
C5'–C4'–C3'	123.8(5)
F1'–C4'–C3'	117.8(6)
C4'–C5'–C6'	118.3(6)
C1'–C6'–C5'	119.7(5)
O1'–C7'–C1'	109.0(4)
O1'–C7'–C8'	103.7(4)
C1'–C7'–C8'	115.5(4)
C7'–C8'–C9'	103.9(4)

C10'–C9'–C8'	104.6(4)
O1'–C10'–O2'	108.3(4)
O1'–C10'–C11'	108.4(4)
O2'–C10'–C11'	110.0(4)
O1'–C10'–C9'	107.2(4)
O2'–C10'–C9'	106.5(4)
C11'–C10'–C9'	116.2(4)
C10'–C11'–C12'	109.8(4)
C13'–C12'–C11'	110.2(4)
C14'–C13'–C12'	122.6(5)
C13'–C14'–O2'	124.8(4)
C13'–C14'–C15'	123.0(4)
O2'–C14'–C15'	112.1(4)
O3'–C15'–C14'	124.8(4)
O3'–C15'–C16'	119.1(4)
C14'–C15'–C16'	116.0(4)
O4'–C16'–O5'	127.3(5)
O4'–C16'–C15'	122.8(5)
O5'–C16'–C15'	109.9(4)
O5'–C17'–C20'	109.4(5)
O5'–C17'–C18'	109.2(5)
C20'–C17'–C18'	113.5(5)
O5'–C17'–C19'	102.3(4)
C20'–C17'–C19'	110.9(5)
C18'–C17'–C19'	110.9(6)

Bibliography

[1] Bruker, *SAINT*, *V8.42*, Bruker AXS SE, Karlsruhe, Germany.

[2] L. Krause, R. Herbst-Irmer, G. M. Sheldrick, D. Stalke, *J. Appl. Cryst.* **2015**, *48*, 3–10, doi:10.1107/S1600576714022985.

[3] G. M. Sheldrick, *Acta Cryst.* **2015**, *A71*, 3–8, doi:10.1107/S2053273314026370.

[4] G. M. Sheldrick, *Acta Cryst.* **2015**, *C71*, 3–8, doi:10.1107/S2053229614024218.

[5] C. R. Groom, I. J. Bruno, M. P. Lightfoot, S. C. Ward, *Acta Cryst.* **2016**, *B72*, 171–179, doi:10.1107/S2052520616003954.

[6] D. Kratzert, *FinalCif*, *V167*, https://dkratzert.de/finalcif.html.

6. Computational Methods

Visualization:

Computed structures are illustrated using CYLView software version 20.0.[1]

Conformational analysis

Conformational analyses were performed for ground state and transition structures using CREST.[2] The length of transition state bonds was frozen during conformational searches. 15 of the lowest conformers generated for each structure were reoptimized without restriction using the DFT methods described below.

DFT Calculations

All DFT calculations were performed using the Gaussian16 program.[3] All conformers were optimized and energies calculated using DFT with M062-X functional, 6-311+g(d,p) basis set and SCRF=SMD solvation model for dichloromethane.[4] All ground state structures were confirmed to be potential energy minima by a frequency calculations that had no imaginary frequencies. Transition state structures were confirmed by IRC analysis and a frequency calculation with only one imaginary frequency corresponding to the reaction coordinate. IRC endpoint structures were reoptimized using the M062-X/6-311+g(d,p)/SMD(CH_2Cl_2) level of theory to verify the stationary structures. Gibbs free energies were corrected using the GoodVibes program.[5]

6.1. Energies of 1i,o,q, 3i,o,q, 5i,o,q Transition States and Intermediates

Table of energies in Hartree

1i	-427.538086
1q	-466.815918
1o	-506.090942
2	-650.828796
3i	-1078.401611
3q	-1117.685669
3o	-1156.959717
5i	-1078.385132
5q	-1117.668701
5o	-1156.940063
Int-5i	-1078.339111
Int-5q	-1117.615723
Int-5o	-1156.887939

TS-3i	-1078.338257
TS-3q	-1117.615234
TS-3o	-1156.891235
TS1-5i	-1078.338257
TS1-5q	-1117.614624
TS1-5o	-1156.885742
TS2-5i	-1078.320190
TS2-5q	-1117.615356
TS2-50	-1156.884399

6.2. Atomic Coordinates of 1i,o,q, 3i,o,q, 5i,o,q, Transition States and Intermediates

Atomic coordinates

1i

C -3.03305100 1.82793600 -0.23609000

C -2.35666900 0.70353800 -0.00483100

C -2.89137800 -0.69807800 0.16396000

O -0.99798300 0.68760500 0.10460900

C -1.65693500 -1.54779900 -0.14324200

C -0.53040700 -0.65650300 0.37072700

C 0.81681000 -0.85207300 -0.29117200

C 1.90832400 0.05485500 0.27181000

C 3.26091400 -0.16131100 -0.40357600

C 4.34914000 0.74385700 0.16682100

H -4.10973600 1.79492600 -0.33579100

H -2.52399000 2.77988600 -0.32475200

H -3.74605700 -0.89032900 -0.48290900

H -3.20425100 -0.84158600 1.20294600

H -1.54658600 -1.68980200 -1.22236200

H -1.66414500 -2.52211100 0.34454000

H -0.43525900 -0.75821000 1.45957000

H	0.69999200	-0.67985800	-1.36743000
H	1.10332100	-1.90215900	-0.16025200
H	2.00795500	-0.12530000	1.34923800
H	1.60643800	1.10104700	0.15436900
H	3.15786400	0.01942100	-1.47919900
H	3.55683600	-1.21030300	-0.29062300
H	4.08386400	1.79761000	0.04196500
H	5.30892200	0.57921200	-0.32875800
H	4.48820800	0.56018600	1.23612900

1q

C	-2.11657700	2.37578300	0.20182900
C	-1.95348200	1.09853200	-0.13848200
C	-3.06315500	0.10704400	-0.35319700
O	-0.68924700	0.62492300	-0.38642800
C	-0.34065200	-0.60236800	0.28818600
C	-2.76837000	-1.20557500	0.37892400
C	-1.36870000	-1.68776300	0.00430000
C	1.06028300	-0.96899800	-0.16519100
C	2.11683900	0.07103200	0.19894000
C	3.52139000	-0.33896300	-0.23927900
C	4.57426000	0.70397600	0.12552600
H	-3.11067500	2.78357600	0.33436900
H	-1.26042100	3.02909300	0.32358700
H	-3.12860100	-0.09659600	-1.42900300
H	-4.01052700	0.54378400	-0.03462500
H	-0.33468300	-0.38649100	1.36721400
H	-3.52037900	-1.95553700	0.12450600

H	-2.82089800	-1.04015800	1.46059100
H	-1.33128200	-1.93991100	-1.06200300
H	-1.09784100	-2.58358800	0.56945400
H	1.04266800	-1.12217000	-1.25077800
H	1.31767900	-1.93100000	0.29193300
H	2.10832900	0.22990500	1.28446600
H	1.85716300	1.02944100	-0.26139000
H	3.52596100	-0.50314100	-1.32256100
H	3.77834300	-1.29890700	0.22244200
H	4.35166800	1.66407400	-0.34886200
H	5.57271100	0.39589100	-0.19416800
H	4.60374400	0.86614800	1.20692000

1o

C	-1.37834700	1.42731200	-0.08222800
C	-2.51132400	0.87523700	0.73922900
C	-1.27395500	2.71728200	-0.41650100
O	-0.44588500	0.56357900	-0.57478300
C	-3.25626800	-0.28711900	0.06103300
C	-2.57414800	-1.64304000	0.24493100
C	-1.19045300	-1.75203800	-0.39330800
C	-0.19261600	-0.69585500	0.07462400
C	1.24223900	-1.06008800	-0.27991600
C	2.26804600	-0.04538400	0.21706400
C	3.70278400	-0.43819400	-0.12985100
C	4.72404600	0.57792200	0.37408300
H	-3.19736800	1.70175500	0.92960600
H	-2.14119600	0.54408400	1.71657300

H	-2.03169400	3.42458000	-0.10788600
H	-0.43993400	3.06778600	-1.01315300
H	-3.37846900	-0.06739800	-1.00556400
H	-4.26074700	-0.34302200	0.48887400
H	-3.21642200	-2.42661800	-0.16765200
H	-2.48525000	-1.84399700	1.31947500
H	-1.26472300	-1.67410200	-1.48412000
H	-0.77376000	-2.73877800	-0.16907200
H	-0.26314200	-0.55660000	1.16046900
H	1.31400400	-1.16602200	-1.36930500
H	1.45730200	-2.04233500	0.15375900
H	2.17482600	0.05784500	1.30557700
H	2.04550400	0.93691100	-0.21174000
H	3.79336900	-0.54291500	-1.21669100
H	3.92187500	-1.42322100	0.29724000
H	5.74438300	0.28405500	0.11605400
H	4.66945700	0.67891600	1.46193400
H	4.53902900	1.56424000	-0.06105900

2

C	3.68559900	-1.12117200	-0.90634300
C	3.62522700	0.07702700	-0.32576600
C	2.41041000	0.58187700	0.32766900
O	2.29740700	1.69677000	0.78297200
C	1.20363600	-0.35083500	0.53298200
C	-0.09514300	0.13657200	-0.14012000
O	1.26698900	-1.34080600	1.20190800
O	-0.03026300	0.84778200	-1.10913100

O	-1.15621000	-0.35353400	0.44842200
C	-2.52182800	-0.06273800	-0.04531300
C	-3.39879200	-0.82583300	0.93328000
C	-2.78073400	1.43465800	0.04024100
C	-2.67546200	-0.60866400	-1.45770700
H	4.59005300	-1.45166600	-1.40431600
H	2.84479700	-1.80745200	-0.91176800
H	4.46558100	0.76410000	-0.33060200
H	-3.24462600	-0.46052700	1.95073600
H	-3.16947800	-1.89303600	0.90154300
H	-4.44783100	-0.68472700	0.66596800
H	-2.56803700	1.79941800	1.04792600
H	-3.83628300	1.61683000	-0.17409000
H	-2.18125800	1.99264400	-0.67826300
H	-2.38967500	-1.66274800	-1.49083700
H	-2.07790100	-0.04947600	-2.17687600
H	-3.72620400	-0.53041100	-1.74633200

3i

O	2.14370000	2.19434900	-2.08979400
C	1.71930300	1.45401700	-1.23642700
C	0.61093700	1.77221800	-0.30747300
C	-0.11249800	2.89106400	-0.39822400
O	0.42171200	0.78575000	0.61685300
C	-1.23022100	3.17107400	0.55569800
O	-1.84845200	0.40190300	0.39013500
C	-0.99669300	-1.54035700	1.47967000
C	-1.88455400	-1.04368800	0.31993900

C	-3.33311900	-1.49139900	0.41381800
C	-4.21439100	-0.92724300	-0.69709100
C	-5.66469300	-1.39587700	-0.59725200
C	-6.53780000	-0.83606800	-1.71689700
C	-0.79462100	-0.28526100	2.33526100
C	-0.85936900	0.80976900	1.28667100
C	-1.15859700	2.22280900	1.75412400
C	2.30797000	0.04293000	-1.08822700
O	1.85983700	-0.89326200	-1.69632600
O	3.33770900	0.06317700	-0.26717000
C	4.09759900	-1.16182300	0.05877700
C	5.15181500	-0.65417000	1.02903500
C	3.17232200	-2.16410600	0.73491200
C	4.74185300	-1.71370200	-1.20534100
H	0.12263800	3.58474600	-1.19713600
H	-2.18375000	3.05095200	0.02962200
H	-1.18503900	4.21177600	0.88609200
H	-0.03915000	-1.89858800	1.10002800
H	-1.47576400	-2.35066600	2.03023000
H	-1.46759200	-1.33827700	-0.64812300
H	-3.34893900	-2.58665000	0.37629800
H	-3.72591700	-1.19523700	1.39493900
H	-3.80278500	-1.22606100	-1.66922900
H	-4.18191800	0.16680300	-0.66591600
H	-6.07346100	-1.09489200	0.37379500
H	-5.69245600	-2.49102600	-0.62098500
H	-6.16274900	-1.14787000	-2.69597000

H	-6.54403000	0.25757200	-1.69622600
H	-7.57173500	-1.17875100	-1.62982100
H	0.14460900	-0.26868800	2.88692500
H	-1.62288400	-0.14066200	3.03254900
H	-0.35754700	2.52719600	2.43308600
H	-2.09689900	2.21852700	2.31016600
H	5.78097000	0.09931700	0.55035600
H	5.78327000	-1.48564800	1.34837300
H	4.68050400	-0.21348600	1.91018200
H	2.42688600	-2.55528800	0.04317800
H	2.66874400	-1.69879800	1.58588600
H	3.77281100	-2.99798300	1.10629000
H	5.31490200	-0.93231800	-1.71035800
H	4.00062500	-2.11698600	-1.89438000
H	5.42916100	-2.51547800	-0.92528100

3q

C	1.67504900	-0.54179100	1.48521200
C	0.56759500	0.23351000	2.21785000
O	0.87630100	1.01562500	3.08411000
O	2.15917300	-1.54880100	1.92831800
O	2.00829300	0.10867000	0.38688700
C	3.13458900	-0.31184000	-0.47238500
C	4.42219400	-0.30461500	0.33956700
C	2.83012000	-1.67047900	-1.08415400
C	3.15500000	0.76688000	-1.54306700
C	-0.82066600	-0.05251900	1.79186200
C	-1.85833000	0.68938600	2.18856000

O	-0.89309400	-1.11151200	0.93165000
C	-3.24601400	0.40203900	1.71297500
C	-3.30670700	-0.96856000	1.03836900
C	-2.09616100	-1.17075000	0.13678300
C	-2.07054500	-2.51921300	-0.56554100
C	-0.91776800	-2.59411300	-1.56584500
C	-0.95355100	-1.38157400	-2.49916800
O	-2.08874600	-0.10711200	-0.77479800
C	-0.98151200	-0.09015700	-1.69216200
C	-1.14936600	1.15871800	-2.54340400
C	-1.25798800	2.44973100	-1.72747800
C	-0.00224700	2.79187800	-0.92637000
C	-0.18400100	4.04915400	-0.08049000
H	5.26568500	-0.42584500	-0.34396600
H	4.44653500	-1.11473900	1.06760700
H	4.53683400	0.64978600	0.85920400
H	3.64512800	-1.93818100	-1.76113200
H	1.90604100	-1.62225200	-1.66484200
H	2.74198500	-2.44666300	-0.32409500
H	3.33451300	1.74792900	-1.09766300
H	2.20088500	0.78933200	-2.07614700
H	3.95163600	0.55412000	-2.25901600
H	-1.66093300	1.52218500	2.85382500
H	-3.54879200	1.18874800	1.01340100
H	-3.94258700	0.44721500	2.55412700
H	-4.21301200	-1.07217300	0.44008500
H	-3.29826000	-1.76488800	1.78737900

H	-3.02761800	-2.62714700	-1.08501700
H	-2.00192500	-3.30864200	0.18612600
H	-0.98081700	-3.52281600	-2.13743200
H	0.02914600	-2.61221400	-1.01878700
H	-1.84888600	-1.42212100	-3.13105800
H	-0.08096100	-1.37177800	-3.15968000
H	-0.05624700	-0.01901200	-1.10629100
H	-2.04675400	1.03544700	-3.15974100
H	-0.29456400	1.21776900	-3.22741500
H	-2.10919200	2.36629000	-1.04380900
H	-1.48078600	3.27962900	-2.40726900
H	0.83924400	2.92737600	-1.61584900
H	0.26657400	1.95558200	-0.27294400
H	-0.43604700	4.91114700	-0.70559900
H	0.72381600	4.29246300	0.47723100
H	-0.99420800	3.91439200	0.64274500

3o

C	-1.99062400	0.12135500	-1.55919200
C	-1.37288600	1.52517800	-1.44576200
O	-2.27001600	-0.37104400	-2.61988100
O	-2.08167300	2.50002100	-1.50711900
C	0.10083800	1.55216000	-1.29247700
O	0.63008100	0.29397000	-1.31284700
C	0.79385200	2.68391500	-1.14461100
C	2.28243200	2.66083300	-1.00119300
C	2.82346600	1.31241900	-1.47384700
C	2.00229000	0.16951300	-0.88261400

C	2.43449000	-1.20708900	-1.38462000
C	3.60559000	-1.82019900	-0.61125200
C	3.15880600	-2.56031400	0.65329100
C	2.61263000	-1.66177000	1.76200500
C	1.50724300	-0.70220300	1.33065700
O	2.06860200	0.33041300	0.50363100
C	0.85202900	-0.02417900	2.52782000
C	-0.18099500	1.02697700	2.13405200
C	-1.03204300	1.50830800	3.30626600
C	-2.06442300	2.54518600	2.87131400
O	-2.17003100	-0.38173300	-0.35485200
C	-2.68117300	-1.74712800	-0.13210900
C	-2.63996600	-1.86882900	1.38326800
C	-4.10648800	-1.84947800	-0.65519500
C	-1.74045200	-2.75131700	-0.78301000
H	0.24249900	3.61691300	-1.12256400
H	2.54614800	2.83653900	0.04765400
H	2.72547000	3.47541500	-1.57926400
H	3.86783400	1.17835900	-1.18700200
H	2.75537500	1.24429900	-2.56262400
H	2.68220300	-1.09870400	-2.44329200
H	1.57115800	-1.87786500	-1.33411700
H	4.33888700	-1.04672400	-0.35701000
H	4.11496700	-2.53109500	-1.26677900
H	4.00106000	-3.13334500	1.05243600
H	2.39118100	-3.29077500	0.36979200
H	3.42122700	-1.05078900	2.17987400

H	2.23059100	-2.28722400	2.57547700
H	0.73124100	-1.22725400	0.75825300
H	1.63248900	0.42752000	3.15259200
H	0.37607100	-0.80598900	3.13116200
H	-0.83900000	0.61207300	1.36170300
H	0.32840400	1.88118600	1.67732400
H	-0.38325200	1.93132100	4.08184000
H	-1.54112400	0.65024700	3.76061900
H	-2.73576800	2.12771500	2.11435100
H	-1.57607800	3.42035400	2.43234400
H	-2.67499000	2.88697500	3.71075700
H	-3.00616000	-2.85297800	1.68255900
H	-3.26898900	-1.10441600	1.84556900
H	-1.61539900	-1.75007700	1.74624700
H	-4.14027900	-1.79132000	-1.74280900
H	-4.72302500	-1.05219200	-0.23321900
H	-4.52652000	-2.80900300	-0.34439900
H	-0.71921700	-2.59858500	-0.42445400
H	-1.75455500	-2.67476800	-1.86991400
H	-2.05584200	-3.75884800	-0.50132100

5i

C	-0.76298100	-1.66932600	-1.40038100
C	0.61477200	-1.25030300	-1.78874800
C	-1.08665100	-1.28082600	0.05064500
O	-0.39272000	-2.06382300	0.98505800
C	-0.75917000	0.21118100	0.27300700
O	0.10232500	0.58754900	1.02268000

O	-1.54534200	0.96597300	-0.47899600
C	-2.58164400	-1.49082500	0.36468300
O	-3.36587700	-1.81311800	-0.49409100
C	-2.96372600	-1.25801300	1.78478600
C	-4.22454100	-1.40709200	2.18305000
C	1.05358400	-0.54260600	-2.82634100
O	1.61188800	-1.64992200	-0.92857500
C	2.55683600	-0.43994200	-2.75193500
C	2.83289700	-0.95424500	-1.32341900
C	3.09746600	0.17236600	-0.33822300
C	3.28257400	-0.29149800	1.10216900
C	3.50918300	0.87347900	2.06289700
C	3.66240100	0.41476500	3.51022700
C	-1.45222300	2.43626000	-0.46874300
C	-1.83656600	2.95072100	0.91174800
C	-0.05596600	2.87676900	-0.89081800
C	-2.48377200	2.84393200	-1.50897800
H	-0.86648300	-2.75633400	-1.47135900
H	-1.48169200	-1.21026500	-2.07775600
H	0.55374000	-1.93561400	0.81955800
H	-2.17722800	-0.96022100	2.46961800
H	-4.99465200	-1.71647600	1.48329100
H	-4.51643700	-1.22774200	3.21154600
H	0.42728200	-0.14327800	-3.61075800
H	2.92054300	0.58400800	-2.87126000
H	3.03955700	-1.05589000	-3.51668600
H	3.63076300	-1.69742200	-1.28694600

H	2.26124000	0.87987500	-0.39484300
H	3.99593200	0.70125900	-0.67701000
H	4.13048900	-0.98576600	1.15893800
H	2.39390400	-0.84240400	1.42543700
H	2.66202600	1.56340600	1.98285600
H	4.40103900	1.43057600	1.75426400
H	3.82663700	1.25885900	4.18450100
H	4.51020100	-0.26848200	3.61627500
H	2.76494800	-0.11300100	3.84591000
H	-1.89669200	4.04111500	0.87805700
H	-1.10052400	2.66837200	1.66437400
H	-2.81590900	2.56114300	1.20097400
H	0.68355500	2.67487000	-0.11709200
H	-0.07415600	3.95219600	-1.08347700
H	0.23956600	2.36804300	-1.81240600
H	-2.52537900	3.93282100	-1.57600700
H	-3.47273200	2.47163400	-1.23304200
H	-2.21657700	2.44264800	-2.48933300

5q

C	0.43617300	0.44884900	-1.80422000
C	-0.55020700	-0.63611600	-1.48996400
C	0.89163200	1.17156100	-0.52320000
O	-0.10119500	2.00423500	0.01771700
C	1.30868700	0.11366700	0.51947700
O	0.66124000	-0.12355500	1.50434600
O	2.43349600	-0.47790400	0.14473200

C	2.09591600	2.09324900	-0.79523100
O	2.63291800	2.12899500	-1.87550100
C	2.53364000	2.91458600	0.36770400
C	3.56796500	3.74481400	0.26328200
C	-0.37102600	-1.93580500	-1.73939100
O	-1.64350800	-0.13100100	-0.83854200
C	-1.32849000	-2.98754200	-1.24990800
C	-2.23752500	-2.38998000	-0.17651300
C	-2.74977900	-1.02869300	-0.62276400
C	-3.66233900	-0.36040000	0.38922000
C	-4.20672400	0.98832400	-0.08103800
C	-5.02995500	1.71573000	0.98444500
C	-6.27254100	0.94586900	1.42868100
C	3.03463600	-1.57081600	0.92804700
C	3.37911100	-1.07424800	2.32658800
C	2.08945100	-2.76491900	0.94370800
C	4.29805800	-1.89064300	0.14452700
H	-0.00914200	1.20406000	-2.45833200
H	1.30369700	0.01994100	-2.30375600
H	-0.88024700	1.45020100	0.17788800
H	1.98084700	2.80045700	1.29397800
H	4.10106100	3.85043900	-0.67644500
H	3.90434700	4.33714100	1.10659100
H	0.52624700	-2.22700300	-2.27330300
H	-0.76519900	-3.83183100	-0.84176000
H	-1.92665900	-3.38464800	-2.07762400
H	-1.68018300	-2.25750300	0.75724200

H	-3.08914000	-3.04220100	0.02868800
H	-3.26544900	-1.13178600	-1.58778500
H	-3.10394100	-0.23448800	1.32589200
H	-4.48578100	-1.05105800	0.59431200
H	-4.82498400	0.83221700	-0.97383300
H	-3.37157300	1.62634900	-0.38539400
H	-5.33290300	2.69005300	0.58909400
H	-4.39368700	1.91724000	1.85363300
H	-6.89249800	0.67873900	0.56700500
H	-6.88428100	1.54564800	2.10692400
H	-6.01138700	0.02288800	1.95195800
H	3.94446200	-0.14053400	2.26570700
H	4.00607200	-1.82156700	2.81862300
H	2.48795400	-0.91447600	2.93188400
H	1.16241800	-2.53740800	1.47007200
H	2.58359600	-3.59574500	1.45343800
H	1.85683700	-3.07681600	-0.07719200
H	4.82601200	-2.71755200	0.62373600
H	4.96003400	-1.02221900	0.11514300
H	4.04976300	-2.17961100	-0.87909200

5o

C	0.02288700	0.60737000	1.32593200
C	0.64137600	-0.71428300	0.96820600
C	-0.65198100	1.25062400	0.10156600
O	0.27126000	1.74461100	-0.83373400
C	-1.56799800	0.20446600	-0.56882400
O	-1.33862300	-0.27528000	-1.64678500

O	-2.59121700	-0.07711300	0.22496200
C	-1.51534800	2.46197000	0.50412500
O	-1.69051300	2.75757900	1.66117100
C	-2.10811000	3.21631300	-0.63556100
C	-2.87831000	4.27959900	-0.42037200
C	-3.60867800	-1.07547500	-0.14353600
C	-4.53091700	-1.07581400	1.06564600
C	-4.34751000	-0.60749000	-1.39031000
C	-2.95684900	-2.44045900	-0.32512000
C	0.29289200	-1.87822300	1.52399100
C	0.70977700	-3.25775500	1.07136100
C	1.01987300	-3.32945100	-0.42842800
C	2.38563900	-2.74419600	-0.79751200
C	2.69811000	-1.45210600	-0.05436700
O	1.55655000	-0.56863300	-0.04725800
C	3.84835000	-0.67707300	-0.67491100
C	4.27006900	0.54482700	0.13614800
C	5.41705400	1.31791100	-0.51170000
C	5.83197700	2.53850600	0.30499400
H	0.78366100	1.31044800	1.67831500
H	-0.71863400	0.46799600	2.11080600
H	0.87113700	1.01547600	-1.05337400
H	-1.89056800	2.85865600	-1.63619100
H	-3.07492500	4.62635800	0.58935000
H	-3.32655200	4.82784300	-1.24110700
H	-5.34377600	-1.78721200	0.90667200
H	-4.95914800	-0.08303400	1.22047300

H	-3.98145200	-1.36668000	1.96396800
H	-3.70616100	-0.63049800	-2.27087100
H	-4.72453300	0.40826200	-1.24584200
H	-5.20011600	-1.26829000	-1.56403700
H	-2.32332800	-2.47168500	-1.21063200
H	-3.74434900	-3.19038600	-0.43333300
H	-2.35890800	-2.69443000	0.55391500
H	-0.41841400	-1.81519000	2.34141100
H	-0.11851400	-3.93536600	1.29671300
H	1.56349100	-3.62641900	1.65417200
H	0.23569100	-2.78654500	-0.96565400
H	0.97545100	-4.36883300	-0.76363800
H	2.43127600	-2.56333100	-1.87646000
H	3.17994400	-3.45817000	-0.55513200
H	2.92835200	-1.67318800	0.99465900
H	3.55210500	-0.37453500	-1.68675300
H	4.69537900	-1.36365100	-0.78393000
H	4.57056800	0.22576000	1.14187000
H	3.40985600	1.21170900	0.26128200
H	5.11477000	1.63293800	-1.51654400
H	6.27627200	0.65005300	-0.63957900
H	4.99386000	3.23153600	0.42182700
H	6.65136700	3.08123400	-0.17247300
H	6.16272900	2.24431100	1.30531000

TS1-5i

C	-1.02145200	-1.65711900	-1.27828000
C	0.28156500	-1.64046600	-0.74649800

C	-2.01537000	-1.23992200	0.15937000
O	-1.98929600	-2.13011500	1.07381600
C	-1.41267500	0.14472200	0.53011500
O	-0.67107000	0.30442000	1.46604300
O	-1.79008800	1.07796400	-0.33720100
C	-3.42354800	-1.08296900	-0.48878600
O	-3.70925600	-1.49615500	-1.58929800
C	-4.46774600	-0.46265000	0.37415900
C	-4.31010200	-0.16259100	1.66165600
C	0.85156800	-2.63807400	0.18210900
O	1.02980900	-0.58890000	-0.86801600
C	2.28082000	-2.13609200	0.40345700
C	2.17907100	-0.65047500	0.06156200
C	3.37450500	-0.02221500	-0.61452200
C	4.59030400	-0.00632700	0.31154200
C	5.79775900	0.67808500	-0.32582000
C	7.01259600	0.68843400	0.59726400
C	-1.37879700	2.48199900	-0.20400900
C	-2.09676900	3.15658500	-1.36327000
C	-1.87129700	3.03711600	1.12701000
C	0.13208600	2.59574100	-0.36510100
H	-1.41480000	-2.64576600	-1.48752300
H	-1.22068700	-0.91078600	-2.04101900
H	0.19708000	-2.57520000	1.07123700
H	-5.40457000	-0.26744300	-0.14029000
H	-3.39256100	-0.38429100	2.19737700
H	-5.11795200	0.29345000	2.22405900

H	0.76183700	-3.65297000	-0.20402200
H	2.97177600	-2.62967500	-0.28463000
H	2.62967500	-2.28657000	1.42329200
H	1.85884500	-0.06580100	0.92805600
H	3.11940300	1.00099600	-0.90834500
H	3.60196500	-0.58536500	-1.52640200
H	4.86072100	-1.03299300	0.58416100
H	4.33093200	0.50921500	1.24398400
H	5.52799000	1.70527300	-0.59349900
H	6.04833700	0.16606600	-1.26113900
H	7.86697400	1.18108000	0.12733700
H	7.31409400	-0.33038100	0.85685600
H	6.79035700	1.21801900	1.52808000
H	-1.78304500	2.71908000	-2.31369900
H	-1.85959800	4.22242000	-1.37318800
H	-3.17813800	3.03895700	-1.26147300
H	-2.93987300	2.83813000	1.24459800
H	-1.72159100	4.11947800	1.13412600
H	-1.33248300	2.60581000	1.96972200
H	0.65939300	2.10990400	0.45592400
H	0.40490700	3.65404000	-0.37868300
H	0.44509800	2.14492500	-1.30940600

TS1-5q

C	1.13318300	-1.52962800	1.39713300
C	-0.06255600	-1.77870900	0.72224200
C	2.29480100	-1.01766700	-0.05830600
O	2.48923500	-1.95878400	-0.86944300

C	1.47211300	0.19782500	-0.54673800
O	0.72738000	0.14651600	-1.49145400
O	1.68981600	1.25240900	0.22950600
C	3.55576000	-0.60555400	0.75390300
O	3.74957800	-0.90265500	1.90988000
C	4.60285100	0.11851700	-0.02043800
C	4.56254400	0.33872400	-1.33277300
C	-0.22180200	-3.02371000	-0.07506800
O	-0.96627900	-0.83574900	0.74681300
C	-1.36407500	-2.98547300	-1.08283700
C	-2.55787800	-2.26320000	-0.47116100
C	-2.16143800	-0.84824800	-0.10549800
C	-3.20818100	-0.06785700	0.66731000
C	-4.48161200	0.17281800	-0.14235500
C	-5.44713300	1.11920400	0.56892400
C	-6.73462800	1.33721600	-0.22036700
C	1.07043200	2.55895800	-0.02886500
C	1.67592000	3.43980100	1.05365700
C	1.47424800	3.05478700	-1.41200300
C	-0.43876200	2.45092200	0.14244700
H	1.71487400	-2.39925500	1.67843600
H	1.14184500	-0.69731700	2.09212500
H	0.75778300	-3.18809100	-0.54751000
H	5.43655800	0.46409100	0.58436000
H	3.75226300	-0.02260100	-1.95829400
H	5.36536900	0.87540800	-1.82682000
H	-0.35395000	-3.83662400	0.64947300

H	-1.62754500	-4.00141900	-1.37898000
H	-1.04166700	-2.45292700	-1.98210800
H	-2.91278800	-2.78690900	0.42459500
H	-3.38627100	-2.21106600	-1.17991600
H	-1.86431900	-0.30091500	-1.00586000
H	-2.77269200	0.89380800	0.95525600
H	-3.44319600	-0.60997200	1.59012700
H	-4.99049400	-0.77768700	-0.33432600
H	-4.21853100	0.59298700	-1.12119300
H	-4.94898800	2.08008400	0.73689400
H	-5.68460500	0.71293500	1.55822200
H	-7.41075500	2.02093500	0.29825100
H	-7.26442100	0.39234300	-0.37136000
H	-6.52080000	1.76081800	-1.20590100
H	1.43159700	3.05020000	2.04442600
H	1.27895900	4.45340400	0.96811400
H	2.76272200	3.47878100	0.94890000
H	2.56065700	3.00643200	-1.52552900
H	1.16545000	4.09772600	-1.51588200
H	1.00556100	2.47235000	-2.20421400
H	-0.88371900	1.81911000	-0.62664200
H	-0.87580000	3.45030500	0.07219300
H	-0.67366300	2.03678800	1.12549100

TS1-5o

C	-0.84214100	-1.78082700	-1.05375300
C	0.42949900	-1.85761700	-0.47711200
C	-1.91419000	-1.17636800	0.38512400

O	-1.92779900	-1.96047300	1.37267700
C	-1.27093400	0.21776700	0.59343500
O	-0.38728000	0.42833000	1.38261400
O	-1.84547300	1.11239500	-0.20662900
C	-3.29411900	-1.08380700	-0.32538300
O	-3.55053600	-1.55342900	-1.40795400
C	-4.33282700	-0.45202700	0.54693500
C	-5.57788000	-0.27989300	0.11363000
C	-1.57326500	2.55392300	-0.11394600
C	-2.01185300	3.05354100	1.25625800
C	-0.10308400	2.83855600	-0.38902300
C	-2.45232900	3.13421900	-1.21116200
C	0.77071900	-2.93686700	0.48928300
C	1.94937100	-3.81688600	0.02831900
C	3.31296000	-3.17304100	0.26133400
C	3.50839400	-1.84685800	-0.46935100
C	2.54452500	-0.73879200	-0.06143900
O	1.26342300	-0.89645000	-0.75656400
C	3.02804600	0.64798000	-0.45418300
C	4.05846500	1.20819300	0.52563800
C	4.64123300	2.54864000	0.07141400
C	3.58922700	3.64543300	-0.08775200
H	-1.36197100	-2.72294500	-1.17983100
H	-0.96917500	-1.05693100	-1.85260100
H	1.00247400	-2.47096200	1.45541900
H	-4.01989700	-0.13023800	1.53494100
H	-5.86852200	-0.61000500	-0.87891000

H	-6.33313600	0.19025300	0.73341000
H	-1.40349200	2.62315000	2.05206500
H	-3.06190800	2.80676500	1.43173600
H	-1.90453200	4.14039000	1.28824000
H	0.02791200	3.91600800	-0.51819200
H	0.21192600	2.34151500	-1.31053100
H	0.52706100	2.50607000	0.43463300
H	-2.14676600	2.75224300	-2.18795700
H	-2.36259900	4.22242700	-1.21698900
H	-3.49824700	2.86895200	-1.04095600
H	-0.12706400	-3.53462800	0.62874900
H	1.82371300	-4.06884900	-1.02980400
H	1.89355300	-4.75345100	0.58685400
H	4.09523700	-3.86963200	-0.05159900
H	3.44660000	-3.00688100	1.33636600
H	3.42248300	-1.98164800	-1.55382700
H	4.52113500	-1.48252200	-0.27726800
H	2.32983000	-0.76614600	1.01140000
H	2.15845000	1.30538100	-0.48362600
H	3.44572100	0.61011700	-1.46787100
H	4.87534000	0.49250600	0.66313100
H	3.58111400	1.33448000	1.50500000
H	5.17050000	2.40785800	-0.87754900
H	5.38991300	2.87043900	0.80136200
H	4.05765900	4.60682000	-0.31192100
H	3.00785000	3.76227400	0.83227300
H	2.89124900	3.42259600	-0.89891500

Int-5i

C	-0.99990400	-1.69767300	-1.21823500
C	0.24486700	-2.02900400	-0.62811400
C	-1.92372100	-1.06925600	0.12716900
O	-2.11551000	-1.91597700	1.07642100
C	-1.08173700	0.18546200	0.49474400
O	-0.30764900	0.20848100	1.41789900
O	-1.30206100	1.17515700	-0.36610300
C	-3.25901700	-0.65838800	-0.56166100
O	-3.58453200	-1.03200200	-1.66628500
C	-4.19777000	0.15910800	0.25736900
C	-4.02041100	0.46410800	1.54088100
C	0.47519200	-3.14980400	0.30080600
O	1.24759900	-1.21945300	-0.70013400
C	1.97136400	-3.05631800	0.60440800
C	2.30294100	-1.60283600	0.27328100
C	3.65223000	-1.35845900	-0.36111000
C	3.96069400	0.11164300	-0.64840800
C	4.18521700	0.95576000	0.60602100
C	4.54244000	2.39927000	0.26286100
C	-0.64780400	2.48294200	-0.24344400
C	0.84969200	2.32680600	-0.47407100
C	-1.28658300	3.28921400	-1.36433800
C	-0.97212700	3.10189900	1.11119500
H	-1.59815300	-2.56093100	-1.49209000
H	-0.94716000	-0.92986900	-1.98546900
H	-0.19458300	-2.91346200	1.15082100

H	-5.07173300	0.50329300	-0.28873100
H	-3.17024700	0.09706600	2.10697000
H	-4.74708800	1.07278200	2.06865100
H	0.13123100	-4.09772900	-0.11208000
H	2.53772600	-3.72616500	-0.04663400
H	2.20997800	-3.28517300	1.64122700
H	2.11743400	-0.94891300	1.12804400
H	3.70110500	-1.95034900	-1.28091900
H	4.40301700	-1.77011100	0.32300400
H	3.14945000	0.54656900	-1.24325900
H	4.86093200	0.16083300	-1.26962600
H	4.98953300	0.50693200	1.19960300
H	3.28962100	0.94423600	1.23580700
H	4.68146000	3.00300100	1.16276700
H	3.75274300	2.86317100	-0.33493800
H	5.46836100	2.44308600	-0.31789100
H	1.31230600	3.31682800	-0.46319500
H	1.03518400	1.86928100	-1.44914800
H	1.31086900	1.71522800	0.30212400
H	-0.85702000	4.29290400	-1.38733500
H	-2.36448700	3.37293200	-1.20672200
H	-1.10851200	2.80980900	-2.32950900
H	-0.46300800	2.58761400	1.92500500
H	-2.05073400	3.07332300	1.28825100
H	-0.65438100	4.14731900	1.10477700

Int-5q

C	0.70839500	-1.39081200	1.37922000

C	-0.37291100	-1.87517600	0.55347100
C	1.96902100	-1.02482600	0.35086100
O	2.43019500	-2.05456100	-0.30950300
C	1.36233800	0.10022200	-0.53566900
O	0.83623500	-0.11757200	-1.59855000
O	1.45693700	1.28969700	0.05502300
C	3.06720500	-0.44743800	1.28801400
O	3.01645400	-0.53738600	2.49617400
C	4.27109300	0.14698500	0.64026000
C	4.50779500	0.15498500	-0.66940400
C	-0.24562400	-3.18252900	-0.10788200
O	-1.34880500	-1.06710200	0.35496500
C	-1.28244300	-3.45682900	-1.18840600
C	-2.61872400	-2.86146800	-0.76321900
C	-2.47948100	-1.36928100	-0.55434800
C	-3.69746100	-0.71129100	0.06146900
C	-3.56843200	0.79826400	0.23980300
C	-4.85162500	1.44034900	0.76174800
C	-4.70155700	2.94689000	0.95367500
C	1.02193800	2.52949000	-0.59899800
C	-0.48907900	2.50804000	-0.78301600
C	1.42979600	3.60169500	0.40036400
C	1.77247300	2.71137600	-1.91296900
H	1.13514200	-2.18155700	1.99331800
H	0.43041600	-0.51558700	1.96286400
H	0.80718800	-3.16267800	-0.46379500
H	4.96722800	0.59943700	1.34135200

H	3.83610100	-0.32499100	-1.37390100
H	5.40290500	0.62220500	-1.06674700
H	-0.25574400	-3.93327700	0.69223800
H	-1.36634700	-4.53052100	-1.35630900
H	-0.96181800	-3.00026600	-2.12870400
H	-2.96881700	-3.32748300	0.16469400
H	-3.38559100	-3.01478900	-1.52507100
H	-2.19763800	-0.87191600	-1.48711200
H	-3.90871300	-1.19288800	1.02280100
H	-4.53972400	-0.93653900	-0.60178000
H	-3.29805800	1.25611900	-0.71933200
H	-2.75030400	1.01500500	0.93343200
H	-5.12877600	0.97224800	1.71264000
H	-5.66935100	1.23576300	0.06208300
H	-5.62521200	3.39886400	1.32265000
H	-4.44253500	3.43544500	0.00973500
H	-3.90805200	3.17025700	1.67274900
H	-0.98028700	2.34440000	0.17873500
H	-0.79338600	1.72717500	-1.47985200
H	-0.81615000	3.47458800	-1.17522500
H	1.14177900	4.58548900	0.02384500
H	2.51152500	3.58716000	0.55344200
H	0.93661700	3.43643200	1.36089600
H	1.57623700	3.71645800	-2.29421900
H	1.45830000	1.98656800	-2.66292000
H	2.84862600	2.61239300	-1.74722000

Int-5o

C	-0.02099400	-1.79224000	-0.94834100
C	1.17551600	-1.50942600	-0.15655400
C	-1.35031100	-1.52430500	-0.09981500
O	-1.54196700	-2.36878600	0.90812600
C	-1.19768800	-0.08503500	0.45583200
O	-0.18169400	0.27417400	1.01088600
O	-2.30024100	0.63793100	0.33232600
C	-2.53127100	-1.60763900	-1.10872700
O	-2.37475800	-1.66948600	-2.31025500
C	-3.88468200	-1.70631600	-0.48068500
C	-4.97553900	-1.84700000	-1.22861200
C	-2.42764700	1.98129700	0.91791100
C	-2.30138600	1.89318400	2.43290000
C	-1.39566800	2.91449100	0.29583800
C	-3.83738100	2.38823900	0.51650300
C	1.52215200	-2.30502200	1.04606400
C	2.77163100	-3.17537600	0.77464000
C	4.08385300	-2.40490300	0.91558600
C	4.24535300	-1.23048900	-0.05074800
C	3.16974100	-0.16113400	0.06879000
O	1.95195700	-0.61846800	-0.63158700
C	3.52099000	1.15504200	-0.59601900
C	2.42319300	2.20868400	-0.46564300
C	2.81289100	3.54116400	-1.10122400
C	1.71227600	4.59026500	-0.96865300
H	-0.05492300	-2.86454300	-1.16100200
H	-0.01204000	-1.21330200	-1.87026700

H	1.71841300	-1.62649000	1.88172900
H	-3.92350900	-1.64674600	0.59913200
H	-4.90170100	-1.91391600	-2.30991500
H	-5.96387300	-1.90129600	-0.78556600
H	-2.55218800	2.86512700	2.86471100
H	-1.28987800	1.62729800	2.73769500
H	-3.00053100	1.15157500	2.82732500
H	-1.44921800	2.86003800	-0.79472800
H	-0.38502400	2.66623200	0.61761800
H	-1.61797700	3.94106300	0.59811200
H	-4.05679200	3.38571300	0.90276400
H	-4.56746600	1.68550600	0.92449400
H	-3.93540100	2.40362900	-0.57138300
H	0.64502300	-2.90268300	1.28818900
H	2.69815100	-3.63151800	-0.21821500
H	2.75518200	-3.99224800	1.49827000
H	4.91849400	-3.09546900	0.77079800
H	4.15822400	-2.02775700	1.94161000
H	4.26935500	-1.57664700	-1.08992400
H	5.20327500	-0.74046200	0.14283700
H	2.88022500	0.00871500	1.10950100
H	3.75385000	0.96845800	-1.65067700
H	4.43866800	1.51633600	-0.12142600
H	2.19834800	2.36295500	0.59689500
H	1.50001300	1.84071300	-0.92611300
H	3.04347300	3.38124100	-2.16019500
H	3.73253200	3.90950300	-0.63334600

H	0.79473500	4.25682100	-1.46173900
H	2.00928500	5.53981200	-1.42041600
H	1.47671600	4.77854500	0.08299700

TS2-5i

C	0.83693000	-1.43850100	1.39143200
C	-0.37539800	-1.84393800	0.72635800
C	1.84339600	-1.09682800	0.11883400
O	1.93968500	-2.07242300	-0.77113200
C	1.25699600	0.21717300	-0.46614500
O	0.61740300	0.25425800	-1.48546000
O	1.52251500	1.25082500	0.32867000
C	3.22523500	-0.82495200	0.77336700
O	3.46993400	-1.11157900	1.92358800
C	4.28491300	-0.24765200	-0.09693500
C	4.17721900	-0.08225000	-1.41359300
C	-0.45036600	-3.02072100	-0.07405600
O	-1.32539100	-0.98572500	0.50858600
C	-1.81374400	-2.94854700	-0.75308000
C	-2.19949200	-1.47323500	-0.58682200
C	-3.63889700	-1.19131100	-0.22750400
C	-3.95309200	0.29557700	-0.08337100
C	-5.42223900	0.55528700	0.24237400
C	-5.72994500	2.04298000	0.38625100
C	1.08582600	2.61525200	0.00618100
C	-0.43628400	2.67806000	0.00025500
C	1.65488900	3.43502700	1.15444400
C	1.69898400	3.05157900	-1.31900800

H	1.32142100	-2.26424900	1.90703200
H	0.73110500	-0.55947400	2.02294500
H	0.54218500	-2.72844200	-0.70471400
H	5.18807200	0.03493200	0.43647400
H	3.29423000	-0.39599000	-1.96084300
H	4.99291600	0.35059400	-1.98306700
H	-0.14411600	-3.94443200	0.41233800
H	-2.53408900	-3.59266900	-0.24249800
H	-1.78459200	-3.22413800	-1.80598200
H	-1.88086900	-0.88233400	-1.44926900
H	-3.88093300	-1.72836500	0.69685000
H	-4.25536000	-1.62785100	-1.02152200
H	-3.69056700	0.81136700	-1.01470600
H	-3.32576800	0.72747800	0.70378600
H	-5.68325900	0.03304100	1.16931100
H	-6.04759300	0.12318700	-0.54655900
H	-5.13448900	2.48806700	1.18856900
H	-6.78406900	2.21365800	0.61715100
H	-5.49787200	2.57989500	-0.53800700
H	-0.85768600	2.09932500	-0.82103500
H	-0.74729700	3.71994500	-0.11013300
H	-0.83126600	2.30079600	0.94689800
H	1.38678100	4.48558100	1.02484400
H	2.74391000	3.35164500	1.17903000
H	1.25373300	3.08655800	2.10885700
H	1.50822800	4.11801700	-1.46156200
H	1.27344200	2.50644600	-2.16064600

H	2.78106100	2.89655700	-1.30145700

TS2-5q

C	0.72550200	-1.35409100	1.37386400
C	-0.38945700	-1.88249600	0.60820000
C	1.89773400	-1.02163600	0.26787300
O	2.21802000	-2.05240500	-0.49695900
C	1.32605800	0.18742300	-0.52080600
O	0.79334300	0.07946500	-1.59562100
O	1.46947300	1.31842000	0.16834200
C	3.12675200	-0.57795300	1.10720400
O	3.18887700	-0.73421100	2.30699000
C	4.28990900	-0.01245900	0.36930400
C	4.40201400	0.02861800	-0.95650400
C	-0.21394200	-3.15214900	-0.04891900
O	-1.36954000	-1.07195300	0.38316200
C	-1.31522400	-3.58194000	-1.00761500
C	-2.63503600	-2.95045100	-0.57611700
C	-2.49391700	-1.44434200	-0.49780500
C	-3.71510700	-0.73559300	0.05268800
C	-3.56925600	0.78109100	0.13128800
C	-4.85632100	1.47476500	0.57107000
C	-4.68627600	2.98806700	0.67234800
C	1.06477400	2.62156800	-0.37184300
C	-0.44718000	2.65423900	-0.54947100
C	1.50156400	3.59136900	0.71605800
C	1.81710800	2.89935400	-1.66792600
H	1.17663800	-2.11555900	2.00704700

H	0.47190700	-0.45795400	1.93670300
H	0.83142000	-2.88813500	-0.52083100
H	5.07228600	0.37824900	1.01402400
H	3.64324200	-0.39248200	-1.60812500
H	5.27931400	0.46476700	-1.42274200
H	0.08847400	-3.90176000	0.68620500
H	-1.39282100	-4.66894500	-1.02473900
H	-1.07249800	-3.25610100	-2.02253600
H	-2.94015800	-3.33436900	0.40381900
H	-3.43380300	-3.17106000	-1.28694900
H	-2.21575300	-1.03289500	-1.47300500
H	-3.94511800	-1.14743300	1.04186500
H	-4.55116400	-0.99467700	-0.60590000
H	-3.26470700	1.16889300	-0.84843100
H	-2.76779200	1.03336900	0.83177600
H	-5.17037400	1.07276300	1.54066100
H	-5.65611400	1.23860100	-0.13928700
H	-5.61370100	3.47716100	0.97946500
H	-4.38652700	3.41109000	-0.29102000
H	-3.91380200	3.24463600	1.40319600
H	-0.93907300	2.41332700	0.39573300
H	-0.77441500	1.94984700	-1.31384500
H	-0.75078000	3.66128100	-0.84723400
H	1.24069600	4.61164000	0.42708300
H	2.58242100	3.53464200	0.86515700
H	1.00373200	3.35679200	1.65963200
H	1.64427800	3.93824800	-1.95934000

H	1.48381500	2.25098600	-2.47722800
H	2.89079400	2.76024800	-1.51579600

TS2-5o

C	1.07047800	-1.34005500	1.30153600
C	-0.17546600	-1.65106700	0.61719900
C	2.16858000	-1.02798900	0.12130800
O	2.31274000	-2.02337700	-0.73800800
C	1.65322700	0.28951600	-0.51841200
O	1.04574600	0.32753100	-1.55733000
O	1.93251300	1.33460100	0.25957600
C	3.50311100	-0.76885100	0.87087300
O	3.65400100	-1.03221600	2.04377400
C	4.64548900	-0.24793000	0.07069600
C	4.64217900	-0.10851800	-1.25323700
C	1.56757900	2.70567800	-0.11200200
C	0.05054900	2.83787100	-0.15146100
C	2.15099600	3.53311600	1.02381600
C	2.22618600	3.07689400	-1.43545600
C	-0.19951800	-2.88288600	-0.14059700
C	-1.27980700	-3.14748800	-1.17626000
C	-2.68920000	-3.26619600	-0.59765100
C	-3.00419500	-2.14143100	0.39708900
C	-2.39980700	-0.79277000	0.02595600
O	-1.02987000	-0.68247000	0.57407500
C	-3.13296500	0.39606900	0.62405400
C	-4.44232000	0.70134800	-0.10145600
C	-5.16525700	1.90612600	0.49895200

C	-6.46521900	2.22675900	-0.23270400
H	1.48074400	-2.21421900	1.80394000
H	0.98520600	-0.48912300	1.97317000
H	0.85934500	-2.71497600	-0.62995000
H	5.51580100	0.01759900	0.66436900
H	3.79155700	-0.40642300	-1.85774500
H	5.51190500	0.28511900	-1.76884300
H	-0.37645600	2.52055000	0.80307700
H	-0.38143400	2.24136600	-0.95489300
H	-0.21118300	3.88655400	-0.31379300
H	1.93140200	4.59027000	0.86061300
H	3.23477500	3.40327000	1.07101200
H	1.71865600	3.22890600	1.97971800
H	1.78728600	2.53532700	-2.27236500
H	3.29772300	2.86405700	-1.39316900
H	2.09629500	4.14847200	-1.60551700
H	0.01708900	-3.72249800	0.52687900
H	-1.01643700	-4.06440300	-1.70772700
H	-1.24609900	-2.34971200	-1.92291000
H	-2.81075400	-4.22893600	-0.09354600
H	-3.40971100	-3.24447400	-1.41977100
H	-2.64130600	-2.39511600	1.39919100
H	-4.08529300	-2.02012000	0.49055800
H	-2.30515800	-0.66062400	-1.05437300
H	-2.47745800	1.26906200	0.56003900
H	-3.32049400	0.20214300	1.68644700
H	-5.11024100	-0.16629300	-0.06986200

H	-4.23073900	0.89699400	-1.15939800
H	-4.49988600	2.77572800	0.46870100
H	-5.37492700	1.70910900	1.55582100
H	-6.27460200	2.45101500	-1.28607300
H	-6.96886300	3.09034000	0.20786200
H	-7.15549200	1.37930800	-0.19160100

TS-3i

O	-0.67526500	0.45231100	-2.39453900
C	-0.82494300	-0.16363400	-1.37323500
C	-0.03349700	-1.40545400	-0.95618400
C	0.82507900	-1.98631200	-1.91546000
O	-0.20534500	-1.77447400	0.21537200
C	1.59449500	-3.07964200	-1.54310400
O	2.58291800	-0.41634200	0.02444400
C	2.48933800	-0.61338800	2.36609500
C	1.98419700	0.22743300	1.19706900
C	2.37214600	1.68806200	1.19123500
C	1.84205700	2.43711200	-0.02996900
C	2.19211800	3.92319600	-0.01028500
C	1.65488200	4.66029900	-1.23392400
C	2.66651600	-2.00136800	1.73773000
C	2.94263000	-1.65660700	0.30188800
C	3.41667300	-2.46602000	-0.69583100
C	-1.83082700	0.31073500	-0.31130100
O	-1.57070600	1.19179700	0.46628600
O	-2.96010000	-0.35620300	-0.43431300
C	-4.10576900	-0.12487400	0.46964800

C	-5.14298100	-1.11013900	-0.04435900
C	-3.69616800	-0.46096000	1.89677800
C	-4.59568000	1.30864100	0.31795100
H	0.94172400	-1.50356400	-2.87702500
H	2.13581400	-3.63523200	-2.30056700
H	1.28286800	-3.65381300	-0.67706400
H	1.78500100	-0.60618700	3.19663800
H	3.45134400	-0.22539100	2.71190700
H	0.90281400	0.11275800	1.08302100
H	1.96745500	2.13503400	2.10660700
H	3.46416800	1.76576600	1.24760300
H	0.75313300	2.31869400	-0.07510000
H	2.24706800	1.98090300	-0.94003400
H	3.28062400	4.03812700	0.04152800
H	1.78620300	4.37648900	0.90093700
H	0.56587500	4.57552100	-1.29095900
H	2.06917600	4.23951300	-2.15465500
H	1.90846500	5.72284400	-1.20664900
H	1.74382200	-2.58667700	1.78487700
H	3.47640600	-2.58780400	2.16998600
H	3.83250200	-3.42562500	-0.41594500
H	3.76750300	-2.00979800	-1.61233300
H	-6.04861600	-1.03490400	0.56080300
H	-4.76342300	-2.13220500	0.01872200
H	-5.39739000	-0.89068600	-1.08356600
H	-3.27213600	-1.46687100	1.94127700
H	-4.58550900	-0.43367200	2.53098400

H	-2.96992000	0.25127500	2.28715200
H	-4.77713800	1.53699300	-0.73516300
H	-3.88192200	2.02533300	0.72239600
H	-5.53952700	1.41398800	0.85816100

TS-3q

C	2.07480100	-1.47475800	-0.12137500
C	0.96058200	-1.64015200	0.92554800
O	1.22521500	-1.69361200	2.09691800
O	2.51546300	-2.42177600	-0.71803300
O	2.45842900	-0.21683200	-0.19679200
C	3.50007000	0.23915100	-1.13575800
C	3.51895700	1.74145600	-0.89834300
C	4.83122400	-0.39441100	-0.75867600
C	3.07562900	-0.08116000	-2.56235300
C	-0.44325800	-1.76648200	0.32246000
C	-1.44989100	-2.28944200	1.16680700
O	-0.54726300	-1.42201400	-0.86328500
C	-2.68851600	-2.63228000	0.64289800
C	-4.12344100	-1.09270800	0.78855200
C	-3.48822800	-0.21372400	-0.04862600
C	-3.63511100	-0.20591700	-1.53424900
C	-3.77977900	1.22973900	-2.07520600
C	-2.87629200	2.20974700	-1.30053500
O	-2.59514900	0.60716300	0.48174300
C	-1.86733700	1.47492500	-0.42886400
C	-1.02183300	2.39131000	0.43200200
C	0.00822800	1.63928400	1.26883300

C	0.94782600	2.56406600	2.03844400
C	1.96690500	1.78317200	2.86436600
H	4.27095300	2.20476700	-1.54034200
H	3.76422500	1.96120300	0.14340300
H	2.54364500	2.17583200	-1.13206200
H	5.05595200	-0.20446300	0.29375300
H	5.62030100	0.05620200	-1.36539400
H	4.82783800	-1.46958200	-0.93665100
H	2.06629800	0.29444500	-2.74886200
H	3.09990500	-1.15191700	-2.76136600
H	3.76149800	0.41687500	-3.25171600
H	-1.22535800	-2.42465100	2.21709000
H	-3.35007800	-3.25850200	1.23092500
H	-2.77637000	-2.74641400	-0.43302600
H	-4.05034500	-0.94884600	1.85918500
H	-4.96980100	-1.64601600	0.40372500
H	-4.47783300	-0.83401100	-1.82053800
H	-2.72325500	-0.66164400	-1.93900500
H	-4.82202400	1.54197700	-1.99376800
H	-3.52618800	1.22351400	-3.13598500
H	-3.47650400	2.84570500	-0.64299100
H	-2.33095000	2.86635200	-1.98098000
H	-1.22146400	0.82454900	-1.03179700
H	-1.68192700	2.98746700	1.07262600
H	-0.51040700	3.08807100	-0.24168000
H	0.60756100	1.00989900	0.60300700
H	-0.50192500	0.96727100	1.96806400

H	0.36293100	3.22110500	2.69210100
H	1.47003000	3.21567300	1.32799200
H	2.66602000	2.44975800	3.37585700
H	2.54307000	1.10750700	2.22566400
H	1.46743700	1.17354100	3.62282700

TS-3o

C	-2.32020100	0.66349200	-0.16487300
C	-1.41104200	0.50733500	-1.39481100
O	-1.99121300	1.31522500	0.79105400
O	-1.35174300	1.37369700	-2.22624000
C	-0.59903800	-0.79133000	-1.40012000
O	-0.70925000	-1.49107600	-0.38325900
C	0.17969100	-1.06636300	-2.54779500
C	0.85711800	-2.27414000	-2.62744200
C	2.79456900	-2.16501100	-1.78876200
C	2.42111700	-1.78270800	-0.52360200
C	2.14914900	-2.77388600	0.56800000
C	3.11876000	-2.64750000	1.75650600
C	2.77315800	-1.49211000	2.69221300
C	2.79460900	-0.11866300	2.02371700
C	1.76468400	0.06575000	0.91032000
O	2.24470200	-0.48544400	-0.34508600
C	1.46346700	1.52667500	0.60674900
C	2.68876300	2.37515300	0.27008400
C	2.30478800	3.79223300	-0.15235700
C	3.52123700	4.65541700	-0.47464900
O	-3.45936100	0.03096000	-0.36422800

C	-4.52017400	-0.00441000	0.66308000
C	-5.01537000	1.40906800	0.93734900
C	-5.60356200	-0.83930000	-0.00049300
C	-3.98868900	-0.69536200	1.91123000
H	0.27434100	-0.30578400	-3.31190900
H	1.30185500	-2.57336200	-3.56991200
H	0.53156800	-3.08678000	-1.98672300
H	3.15904800	-1.41204500	-2.47605400
H	3.09348700	-3.19170600	-1.95314200
H	2.23694500	-3.76538600	0.12205400
H	1.11765300	-2.65884300	0.91358600
H	4.14238000	-2.53823700	1.38184700
H	3.08337800	-3.58429900	2.31797800
H	3.47427400	-1.48869500	3.53147100
H	1.77556800	-1.66446100	3.11287500
H	3.78959500	0.09300400	1.61684000
H	2.59225200	0.64594000	2.77993800
H	0.82988300	-0.45088400	1.15448500
H	0.94398200	1.94456600	1.47450100
H	0.75779400	1.56449000	-0.22885700
H	3.25442600	1.89733600	-0.53754400
H	3.35679800	2.42942500	1.13692100
H	1.72098500	4.26076600	0.64781400
H	1.64687400	3.73927100	-1.02673000
H	4.10528500	4.21659400	-1.28886300
H	4.17805300	4.74521900	0.39552500
H	3.22858800	5.66341200	-0.77837600

H	-5.90776200	1.35131300	1.56507100
H	-5.28726700	1.90339300	0.00148900
H	-4.26573400	2.00728700	1.45381700
H	-5.22525500	-1.83514400	-0.24088600
H	-5.94732800	-0.35982000	-0.91961500
H	-6.45191000	-0.94134100	0.67929300
H	-3.22596500	-0.09780500	2.40955400
H	-3.56922200	-1.67075500	1.65370400
H	-4.81785500	-0.84961200	2.60586300

6.3. References

1. Legault, C. Y.; *CYLview*, *20*; Université de Sherbrooke, **2020**.
2. Pracht, P.; Grimme, S.; Bannwarth, C.; Bohle, F.; Ehlert, S.; Feldmann, G.; Gorges, J.; Müller, M.; Neudecker, T.; Plett, C.; Spicher, S.; Steinbach, P.; Wesołowski, P. A.; Zeller, F. CREST—A Program for the Exploration of Low-energy Molecular Chemical Space. *J. Chem. Phys.* **2024**, *160*, 114110.
3. Frisch, M. J.; Trucks, G. W.; Schlegel, H. B.; Scuseria, G. E.; Robb, M. A.; Cheeseman, J. R.; Scalmani, G.; Barone, V.; Petersson, G. A.; Nakatsuji, H.; Li, X.; Caricato, M.; Marenich, A. V.; Bloino, J.; Janesko, B. G.; Gomperts, R.; Mennucci, B.; Hratchian, H. P.; Ortiz, J. V.; Izmaylov, A. F.; Sonnenberg, J. L.; Williams-Young, D.; Ding, F.; Lipparini, F.; Egidi, F.; Goings, J.; Peng, B.; Petrone, A.; Henderson, T.; Ranasinghe, D.; Zakrzewski, V. G.; Gao, J.; Rega, N.; Zheng, G.; Liang, W.; Hada, M.; Ehara, M.; Toyota, K.; Fukuda, R.; Hasegawa, J.; Ishida, M.; Nakajima, T.; Honda, Y.; Kitao, O.; Nakai, H.; Vreven, T.; Throssell, K.; Montgomery, J. A., Jr.; Peralta, J. E.; Ogliaro, F.; Bearpark, M. J.; Heyd, J. J.; Brothers, E. N.; Kudin, K. N.; Staroverov, V. N.; Keith, T. A.; Kobayashi, R.; Normand, J.; Raghavachari, K.; Rendell, A. P.; Burant, J. C.; Iyengar, S. S.; Tomasi, J.; Cossi, M.; Millam, J. M.; Klene, M.; Adamo, C.; Cammi, R.; Ochterski, J. W.; Martin, R. L.; Morokuma, K.; Farkas, O.; Foresman, J. B.; Fox, D. J. *Gaussian 16*, Revision A.03; Gaussian, Inc.: Wallingford, CT, **2016**.
4. Marenich, A. V.; Cramer, C. J.; Truhlar, D. G. Universal Solvation Model Based on Solute Electron Density and on a Continuum Model of the Solvent Defined by the Bulk Dielectric Constant and Atomic Surface Tensions. *J. Phys. Chem. B* **2009**, *113*, 6378–6396.
5. Luchini, G.; Alegre-Requena, J. V.; Funes-Ardoiz, I.; Paton, R. S. GoodVibes: Automated Thermochemistry for Heterogeneous Computational Chemistry Data. *F1000Res.* **2020**, *9*, 291.
